# Supplementary material for: Loss of adaptive capacity in asthmatic patients revealed by biomarker fluctuation dynamics after rhinovirus challenge
Source: eLife. 2019 Nov 5;8:e47969. doi: 10.7554/eLife.47969 (PMC6877087; doi:10.7554/eLife.47969)
Supplement: Supplementary file 1. [file elife-47969-supp1.zip › RelativeChangeWithinWindowPlots_PDFs/Appendix-figure SS26 IndividualWindowBoundariesRelativeChangeAnalysis FeNO Window Size = 10 AsthmaticParticipants.pdf]

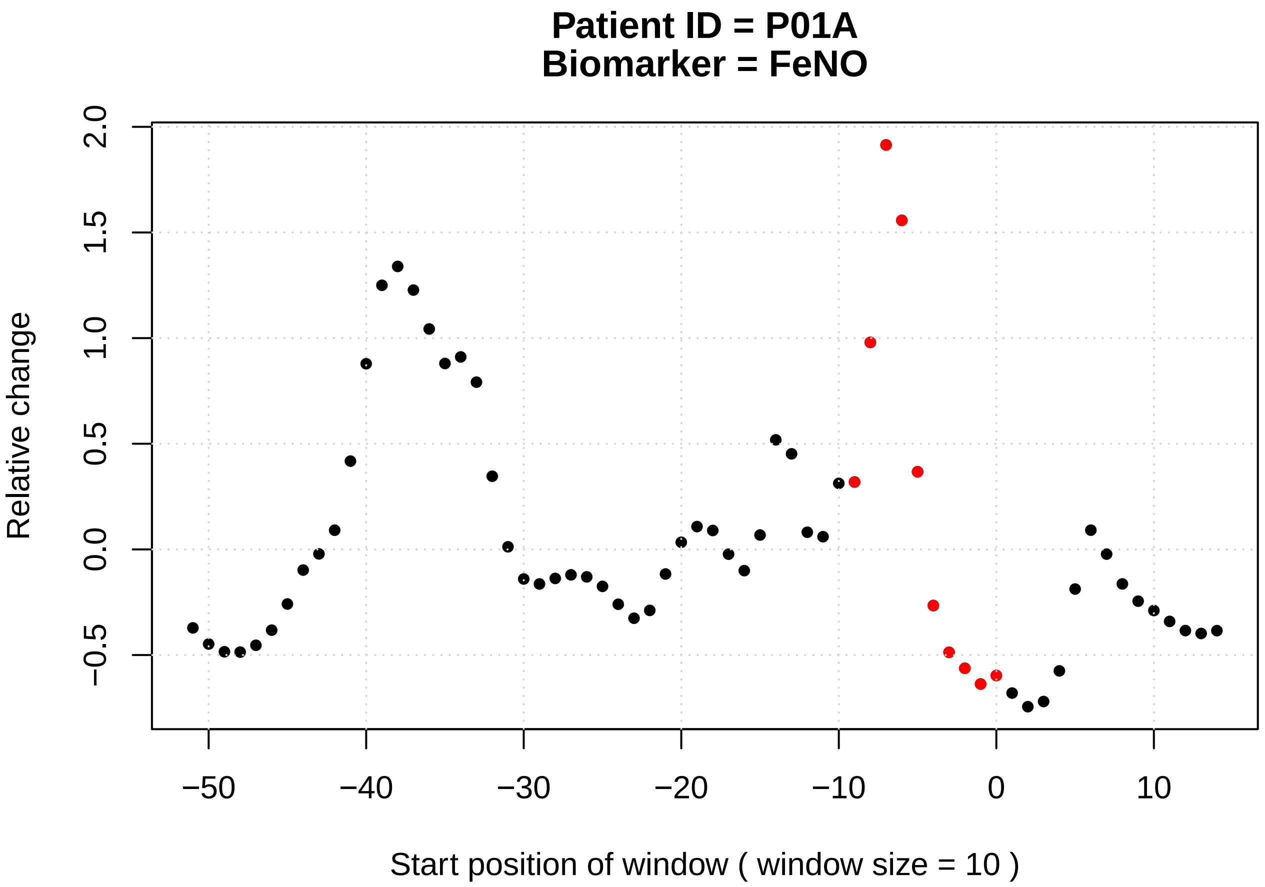
Patient ID = P01A
Biomarker = FeNO
Relative change
2.0
1.5
1.0
0.5
0.0
−0.5
−50
−40
−30
−20
−10
0
10
Start position of window (window size = 10)

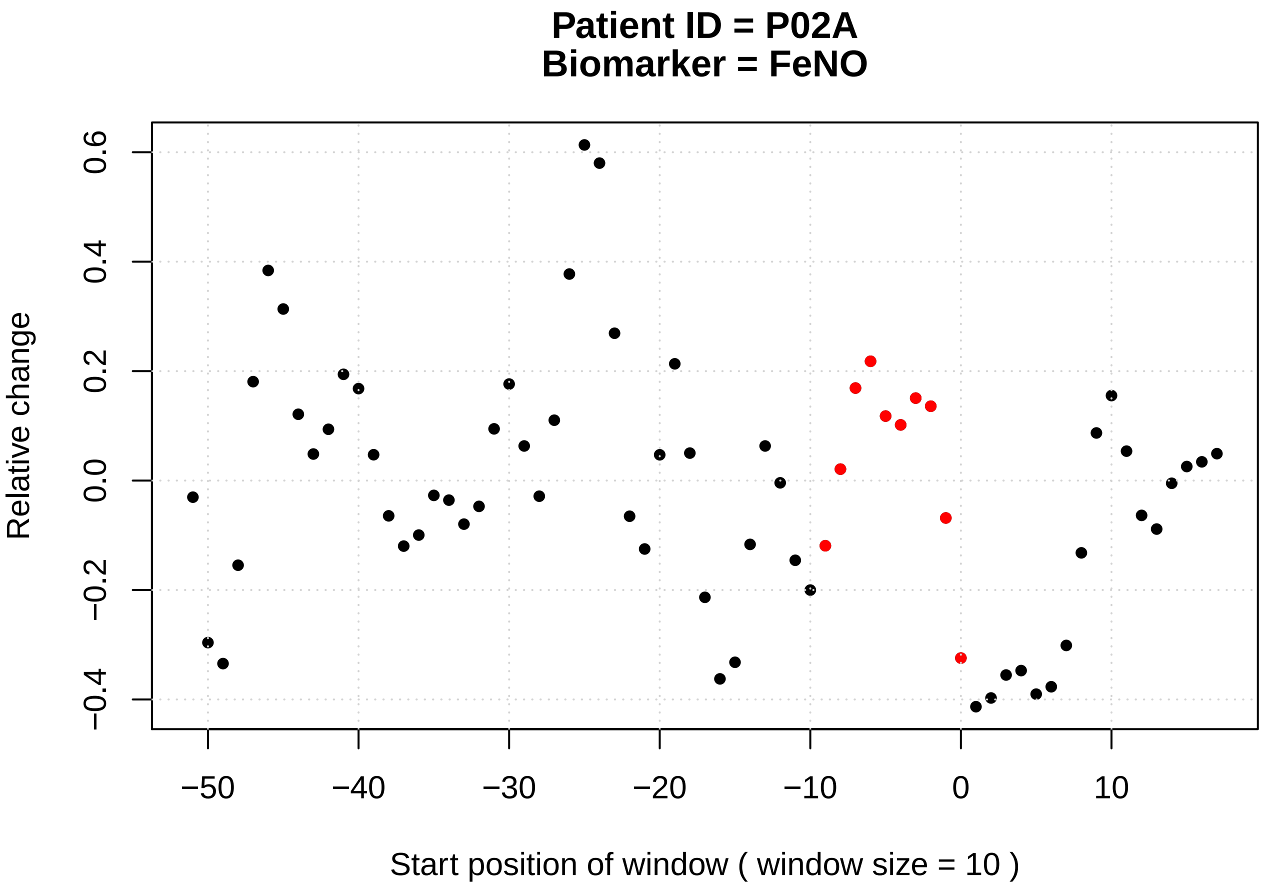

Patient ID = P02A
Biomarker = FeNO
Relative change
0.6
0.4
0.2
0.0
−0.2
−0.4
−50
−40
−30
−20
−10
0
10
Start position of window (window size = 10)

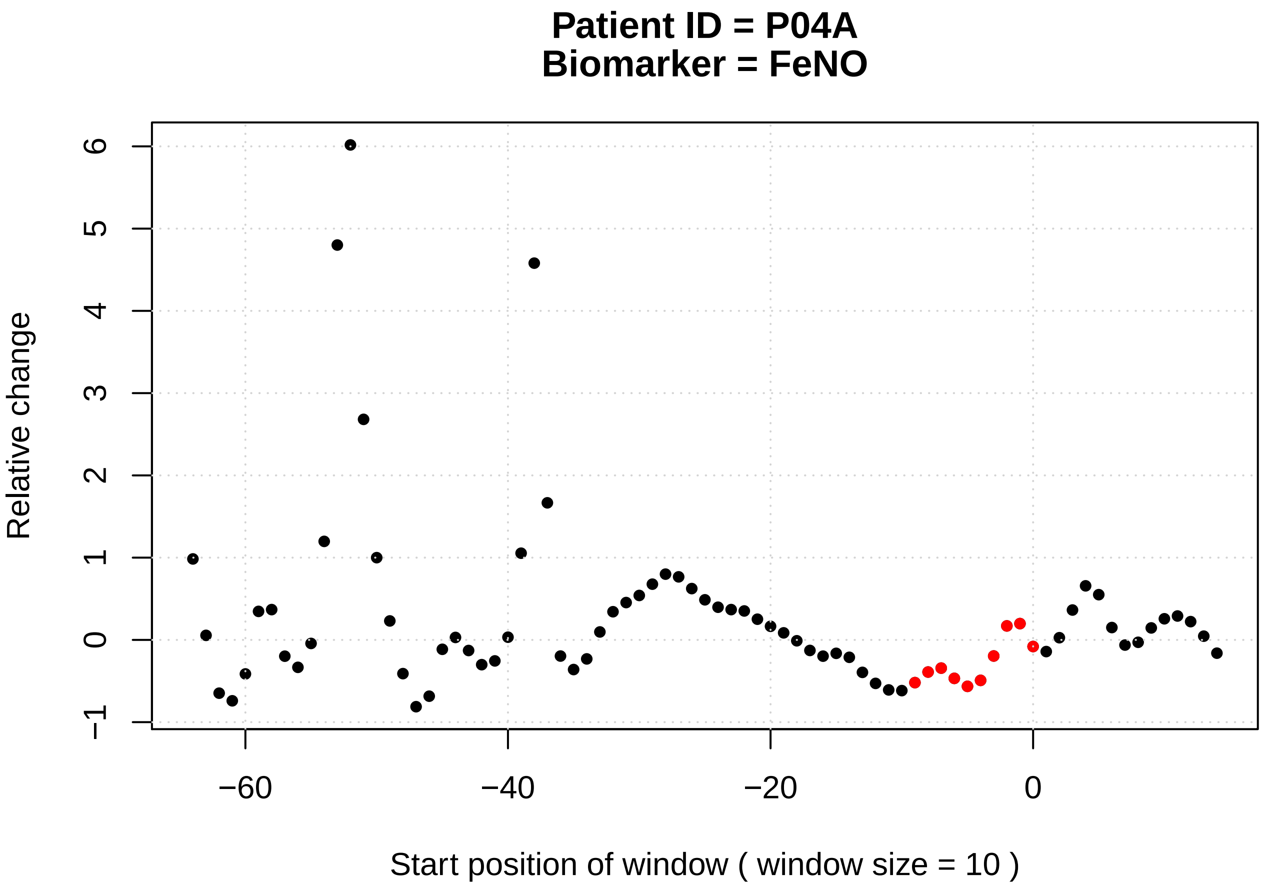

Patient ID = P04A
Biomarker = FeNO
Relative change
−1
0
1
2
3
4
5
6
−60
−40
−20
0
Start position of window (window size = 10)

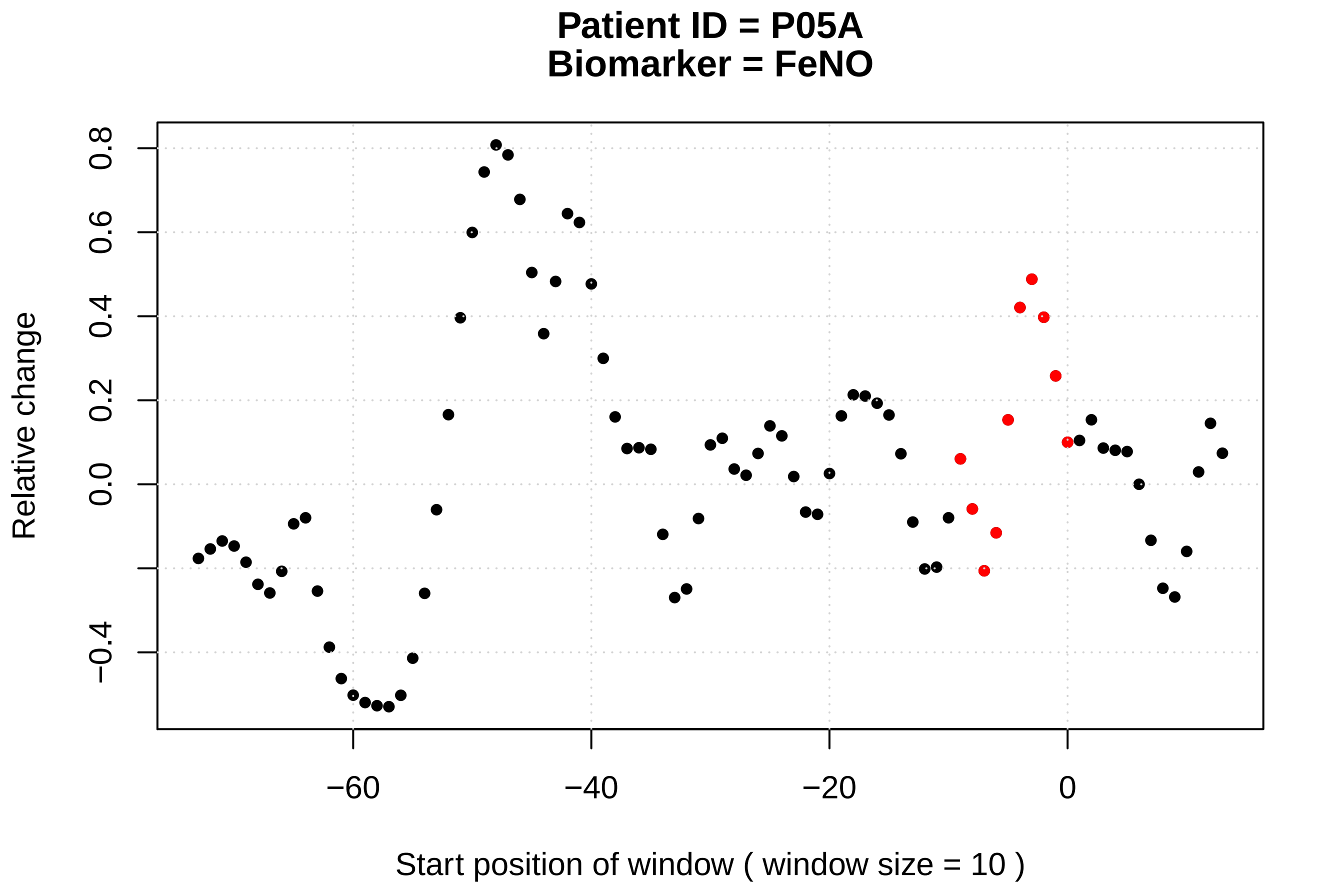
Patient ID = P05A
Biomarker = FeNO
Relative change
0.8
0.6
0.4
0.2
0.0
−0.4
−60
−40
−20
0
Start position of window (window size = 10)

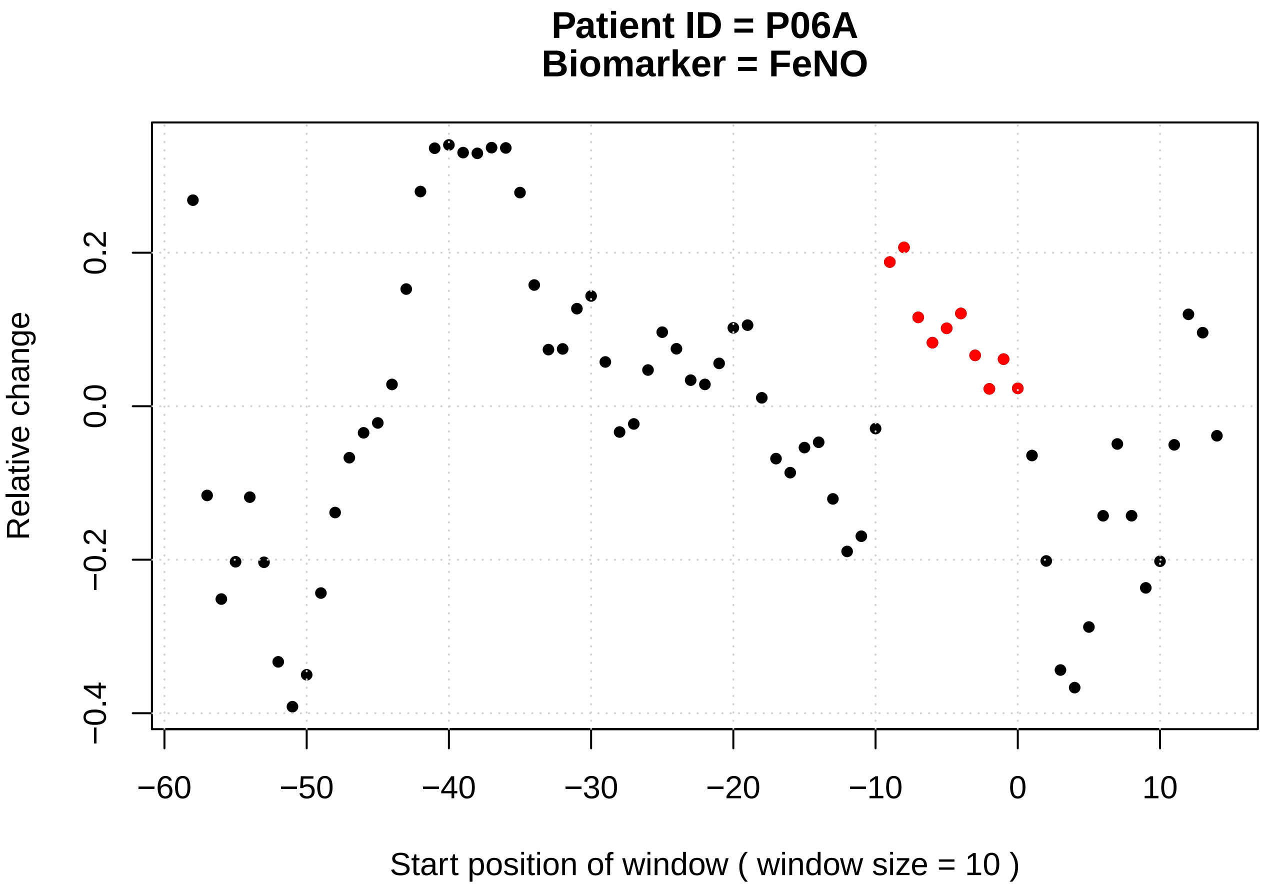

Patient ID = P06A
Biomarker = FeNO
Relative change
0.2
0.0
−0.2
−0.4
−60
−50
−40
−30
−20
−10
0
10
Start position of window (window size = 10)

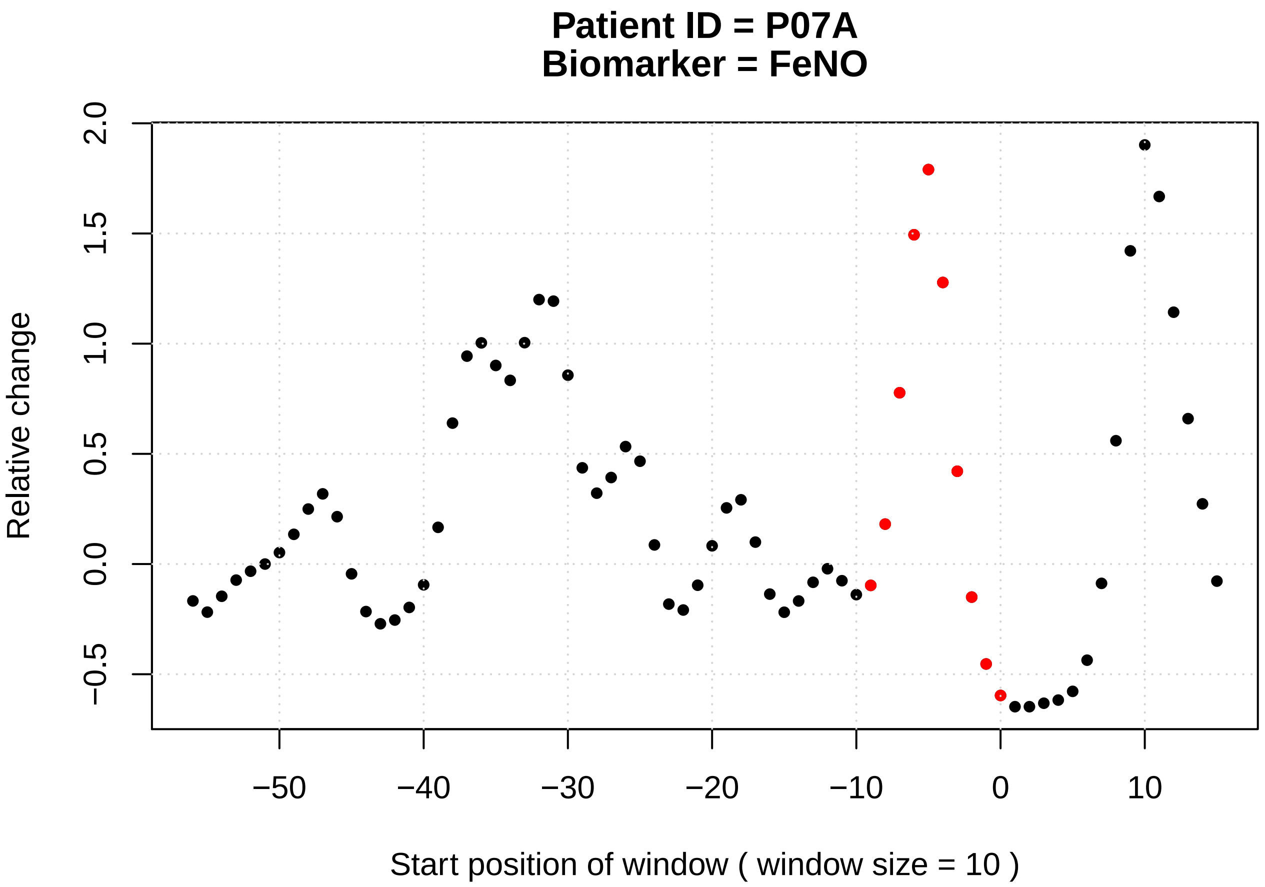
Patient ID = P07A
Biomarker = FeNO
Relative change
2.0
1.5
1.0
0.5
0.0
−0.5
−50
−40
−30
−20
−10
0
10
Start position of window (window size = 10)

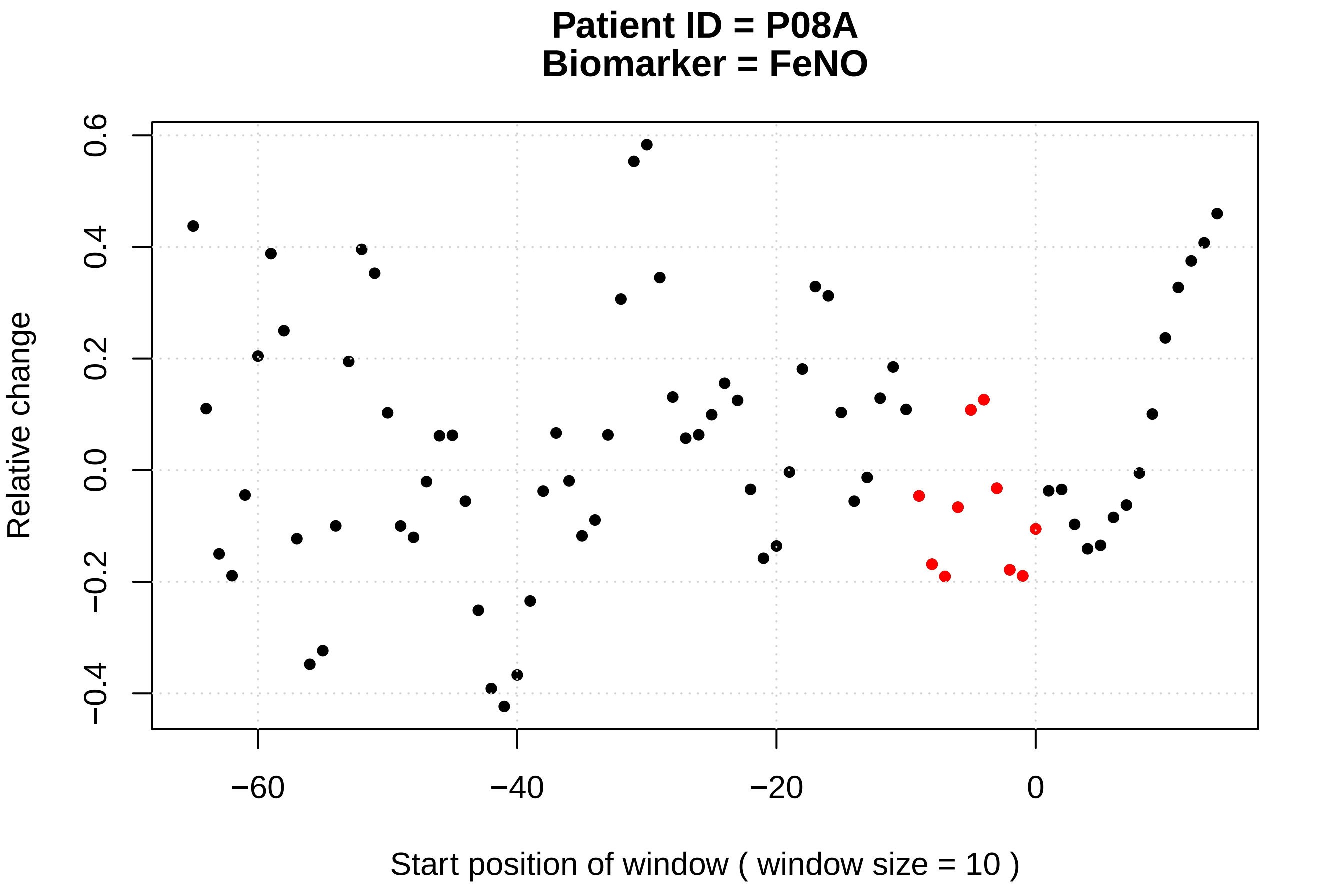
Patient ID = P08A
Biomarker = FeNO
Relative change
0.6
0.4
0.2
0.0
−0.2
−0.4
−60
−40
−20
0
Start position of window (window size = 10)

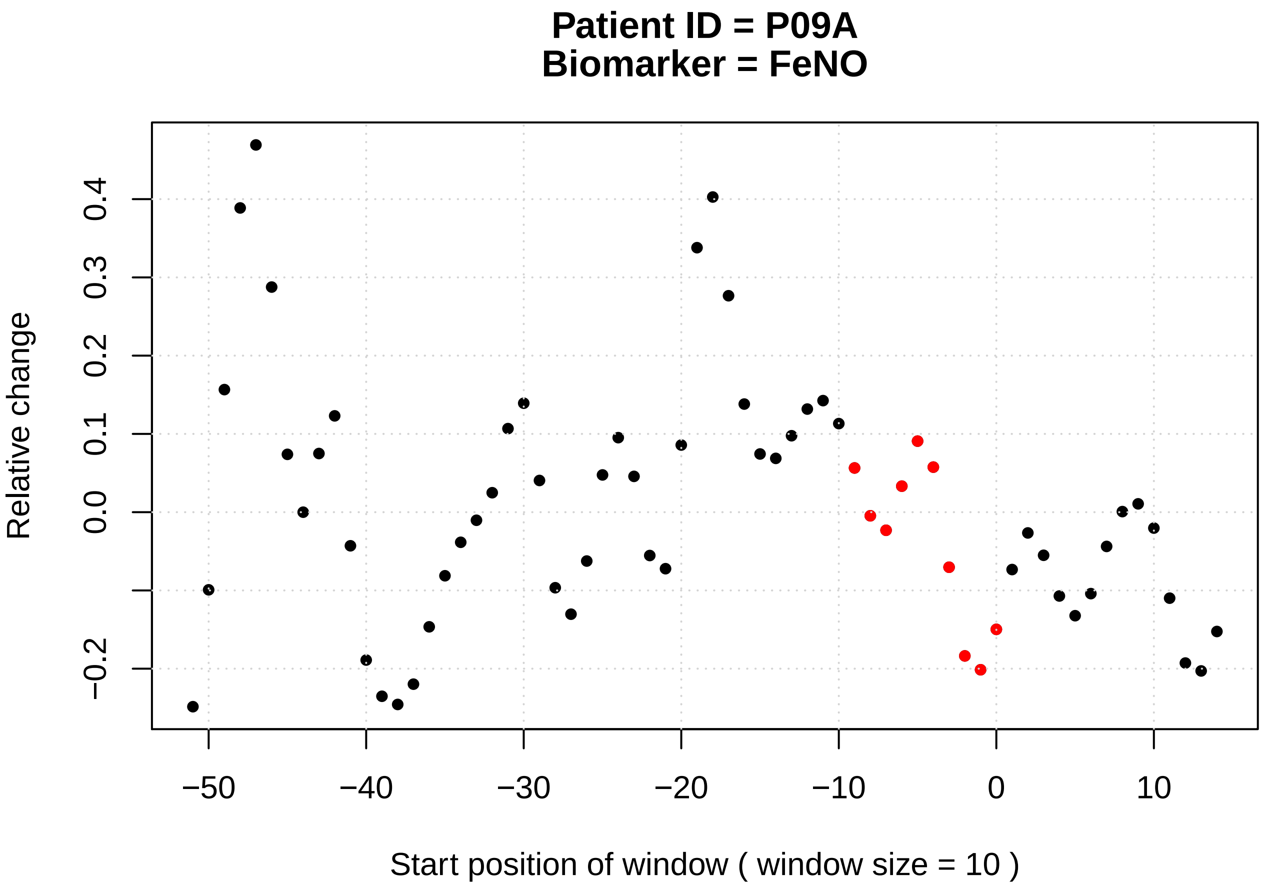

Patient ID = P09A
Biomarker = FeNO
Relative change
0.4
0.3
0.2
0.1
0.0
−0.2
−50
−40
−30
−20
−10
0
10
Start position of window (window size = 10)

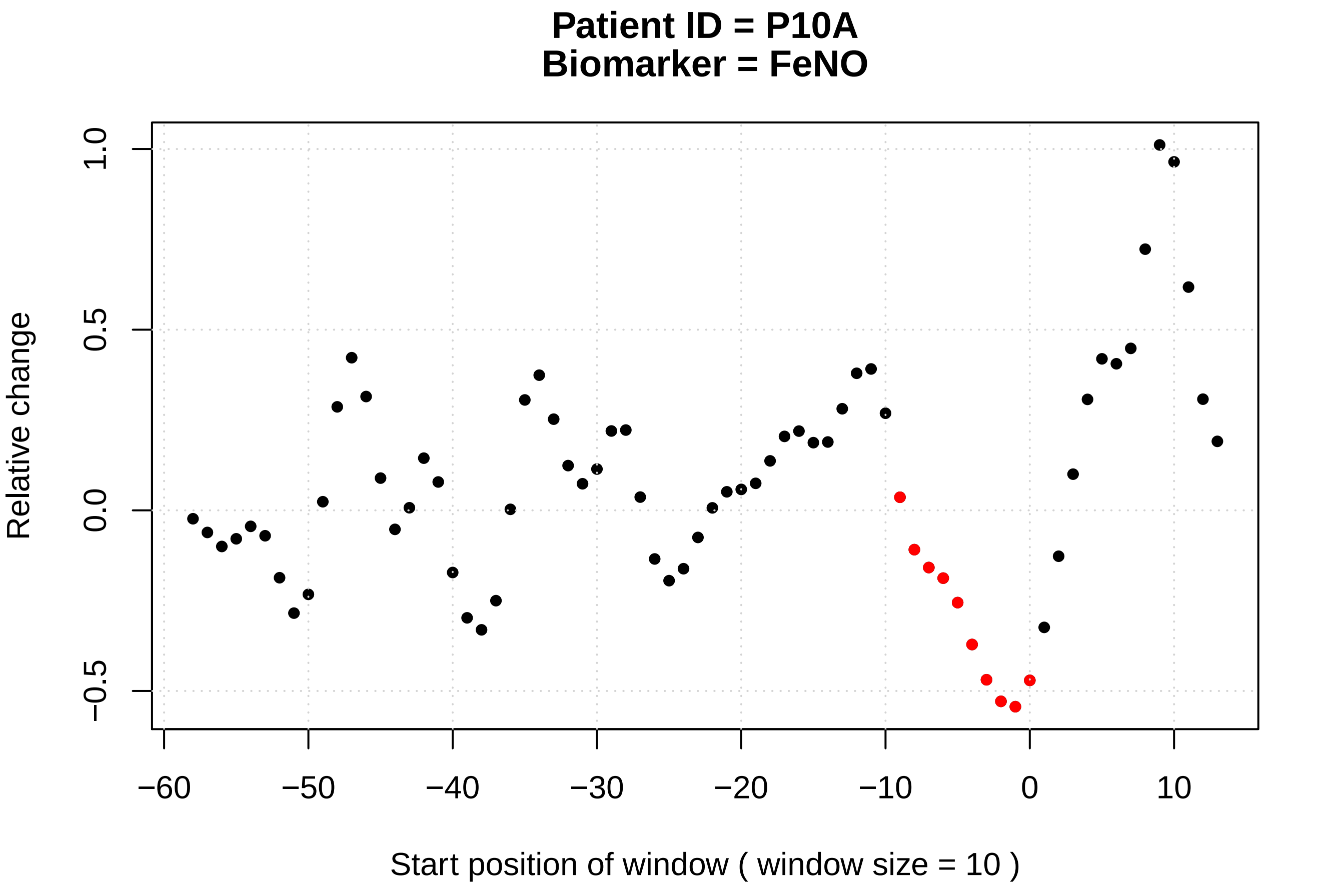

Patient ID = P10A
Biomarker = FeNO
Relative change
1.0
0.5
0.0
−0.5
−60
−50
−40
−30
−20
−10
0
10
Start position of window (window size = 10)

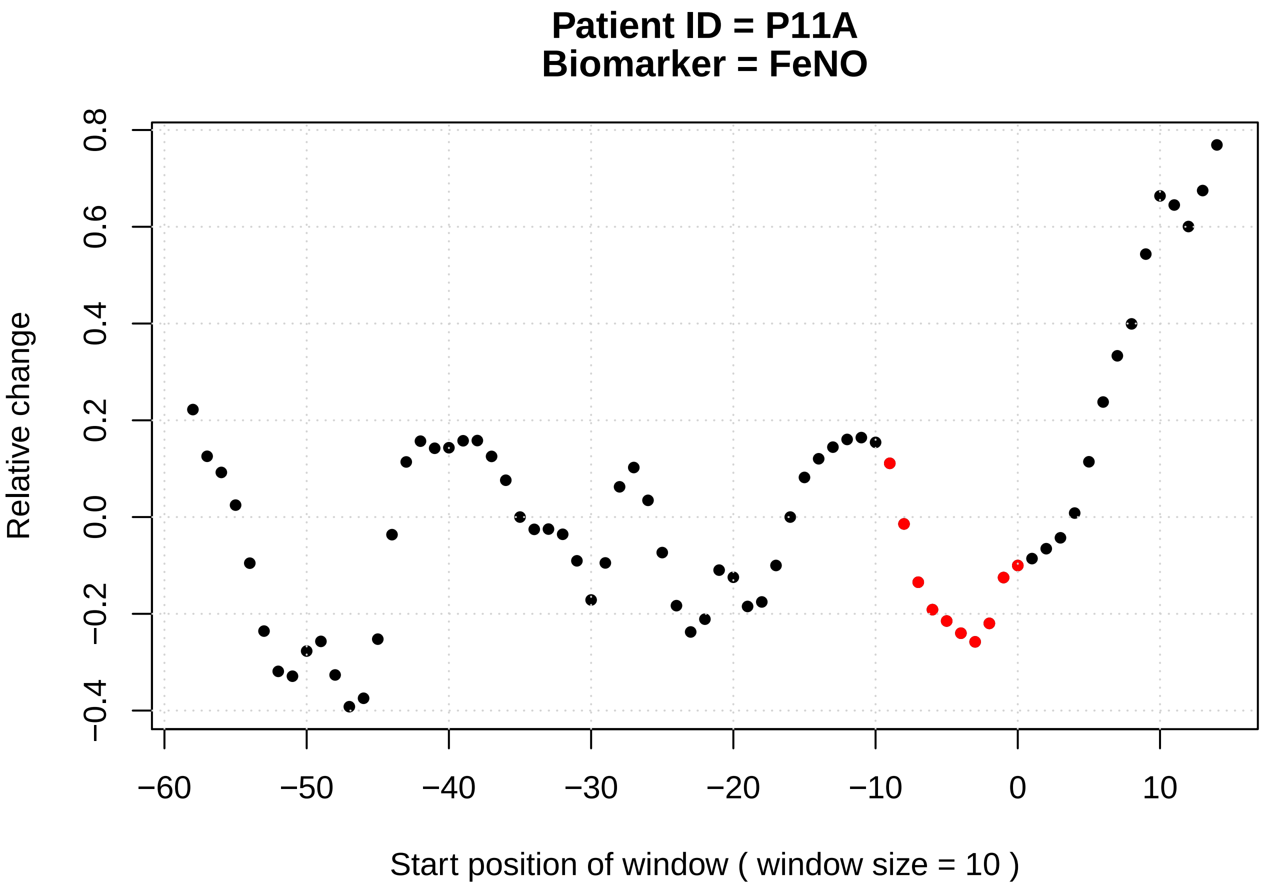
Patient ID = P11A
Biomarker = FeNO
Relative change
−0.4
−0.2
0.0
0.2
0.4
0.6
0.8
−60
−50
−40
−30
−20
−10
0
10
Start position of window (window size = 10)

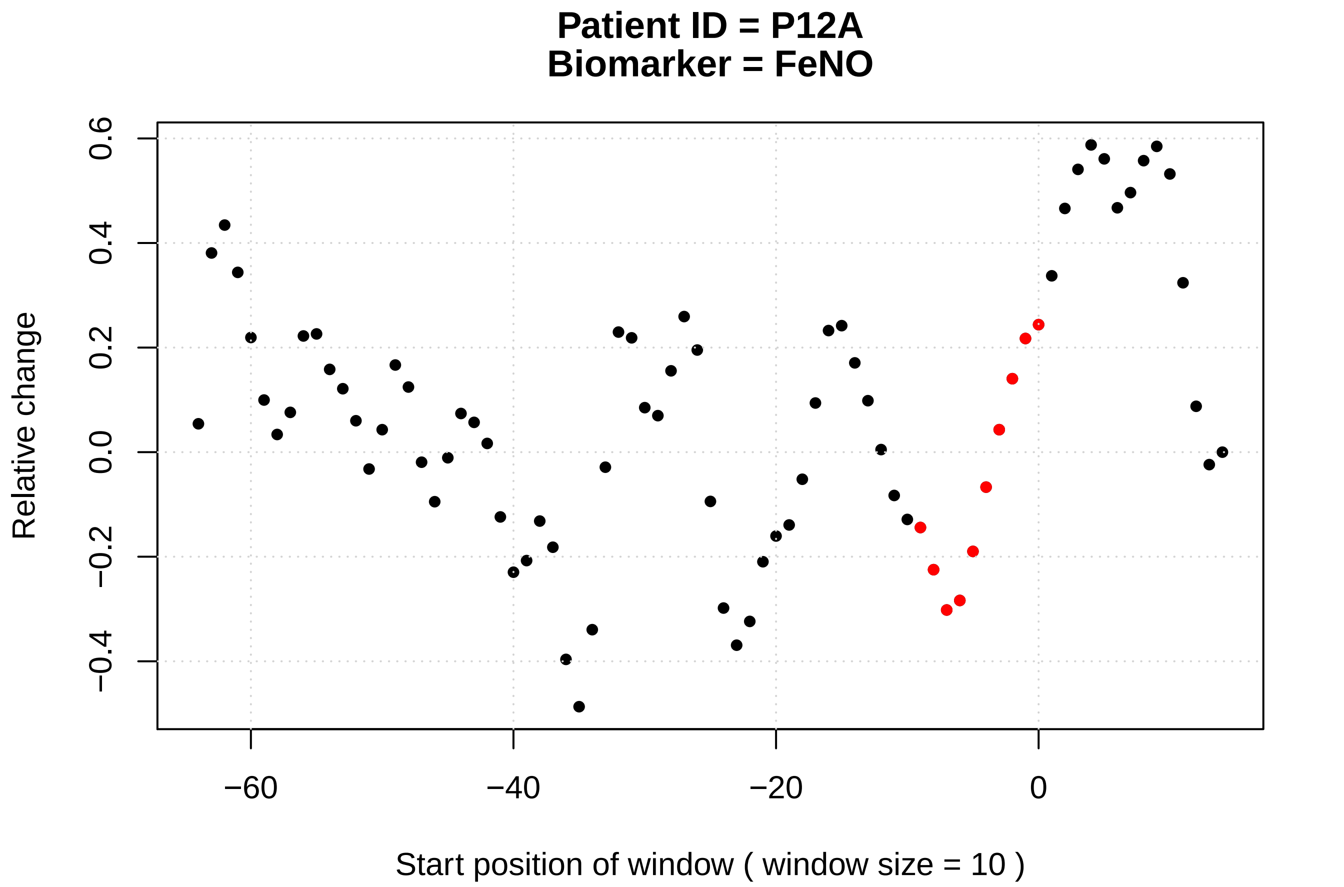

Patient ID = P12A
Biomarker = FeNO
Relative change
Start position of window (window size = 10)
0.6
0.4
0.2
0.0
−0.2
−0.4
−60
−40
−20
0

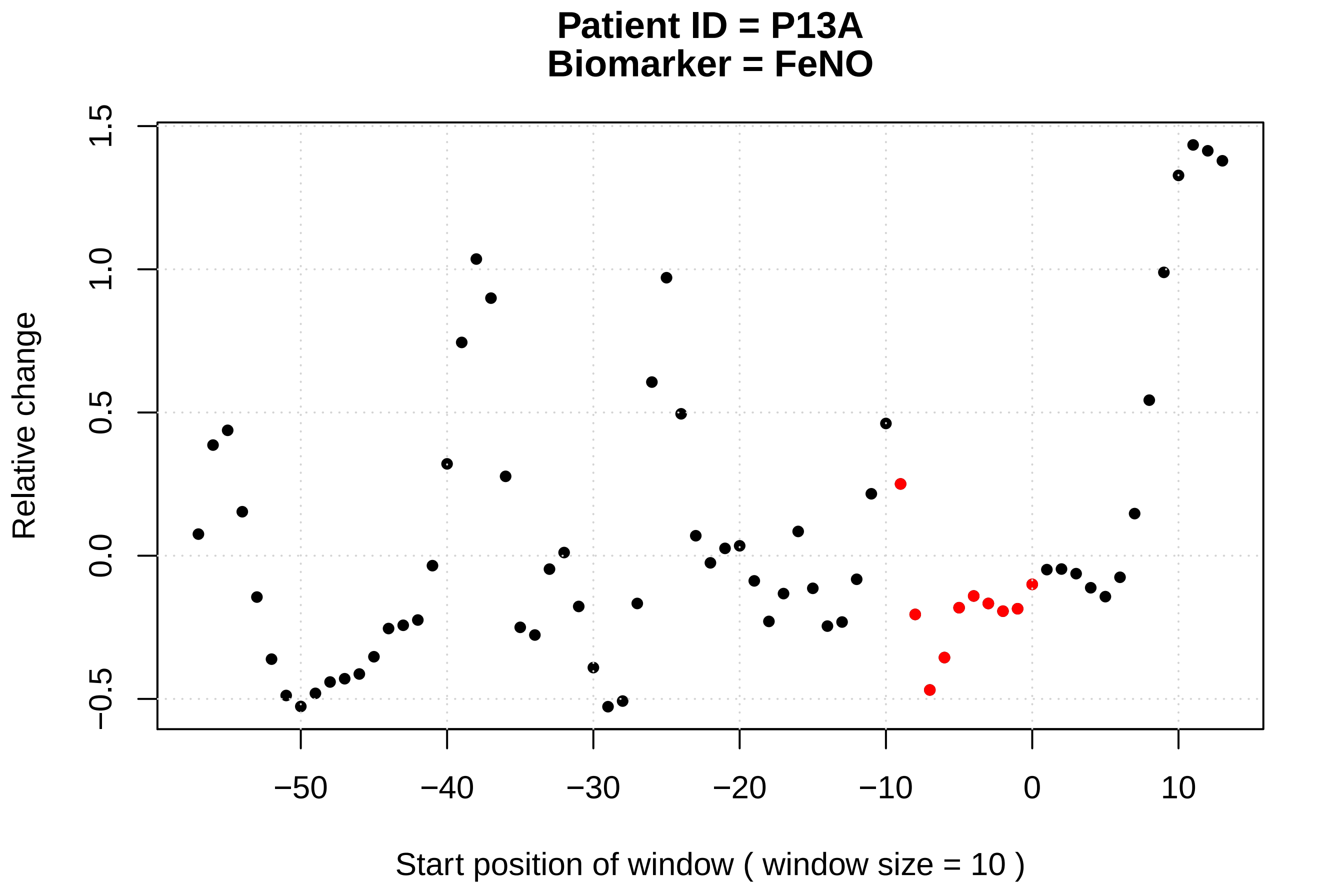
Patient ID = P13A
Biomarker = FeNO
Relative change
Start position of window (window size = 10)
1.5
1.0
0.5
0.0
−0.5
−50
−40
−30
−20
−10
0
10
