# Supplementary material for: Loss of adaptive capacity in asthmatic patients revealed by biomarker fluctuation dynamics after rhinovirus challenge
Source: eLife. 2019 Nov 5;8:e47969. doi: 10.7554/eLife.47969 (PMC6877087; doi:10.7554/eLife.47969)
Supplement: Supplementary file 1. [file elife-47969-supp1.zip › RelativeChangeWithinWindowPlots_PDFs/Appendix-figure SS31 IndividualWindowBoundariesRelativeChangeAnalysis PercentageOfNeutrophils Window Size = 10 HealthyParticipants.pdf]

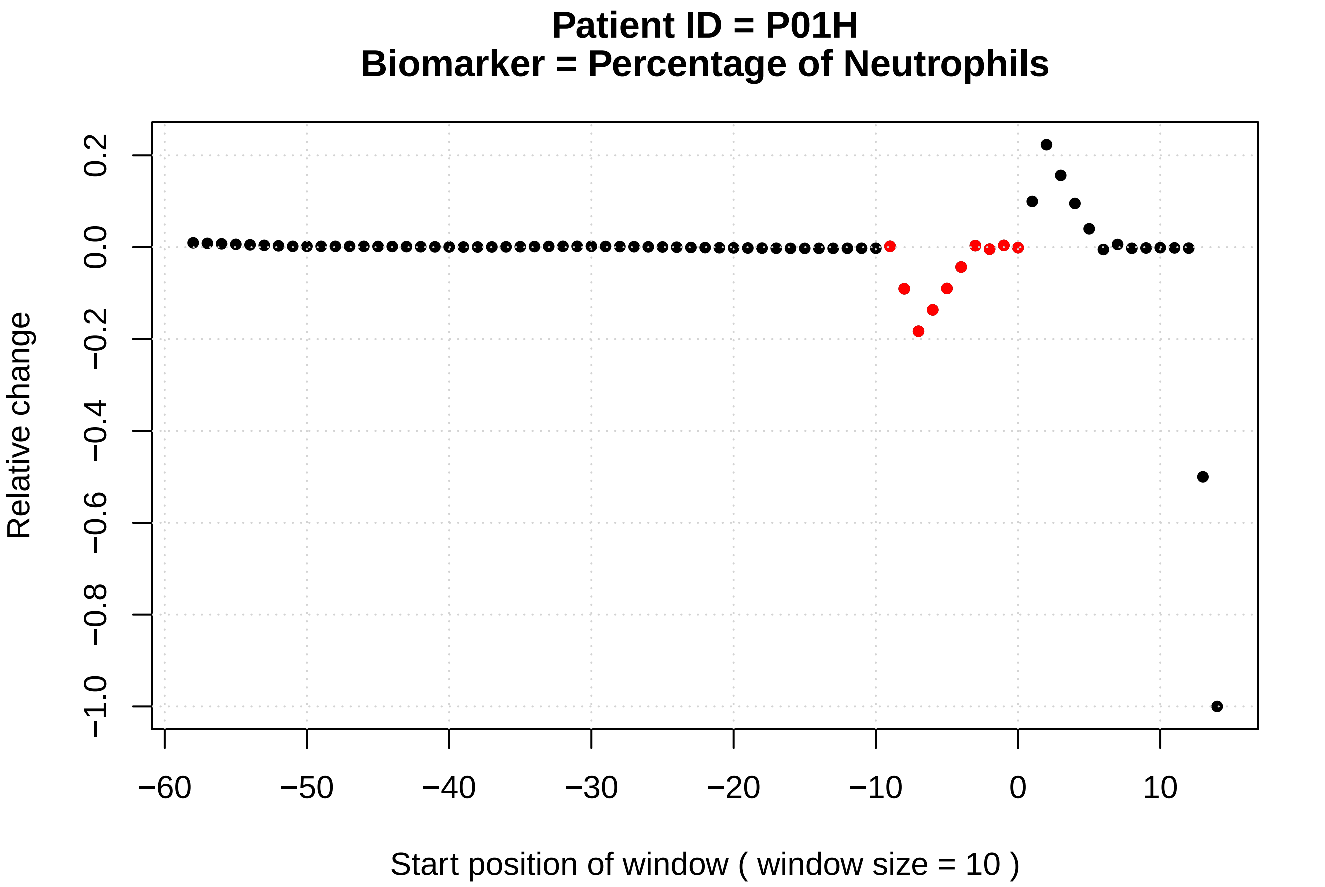
Patient ID = P01H
Biomarker = Percentage of Neutrophils
Relative change
0.2
0.0
−0.2
−0.4
−0.6
−0.8
−1.0
−60
−50
−40
−30
−20
−10
0
10
Start position of window (window size = 10)

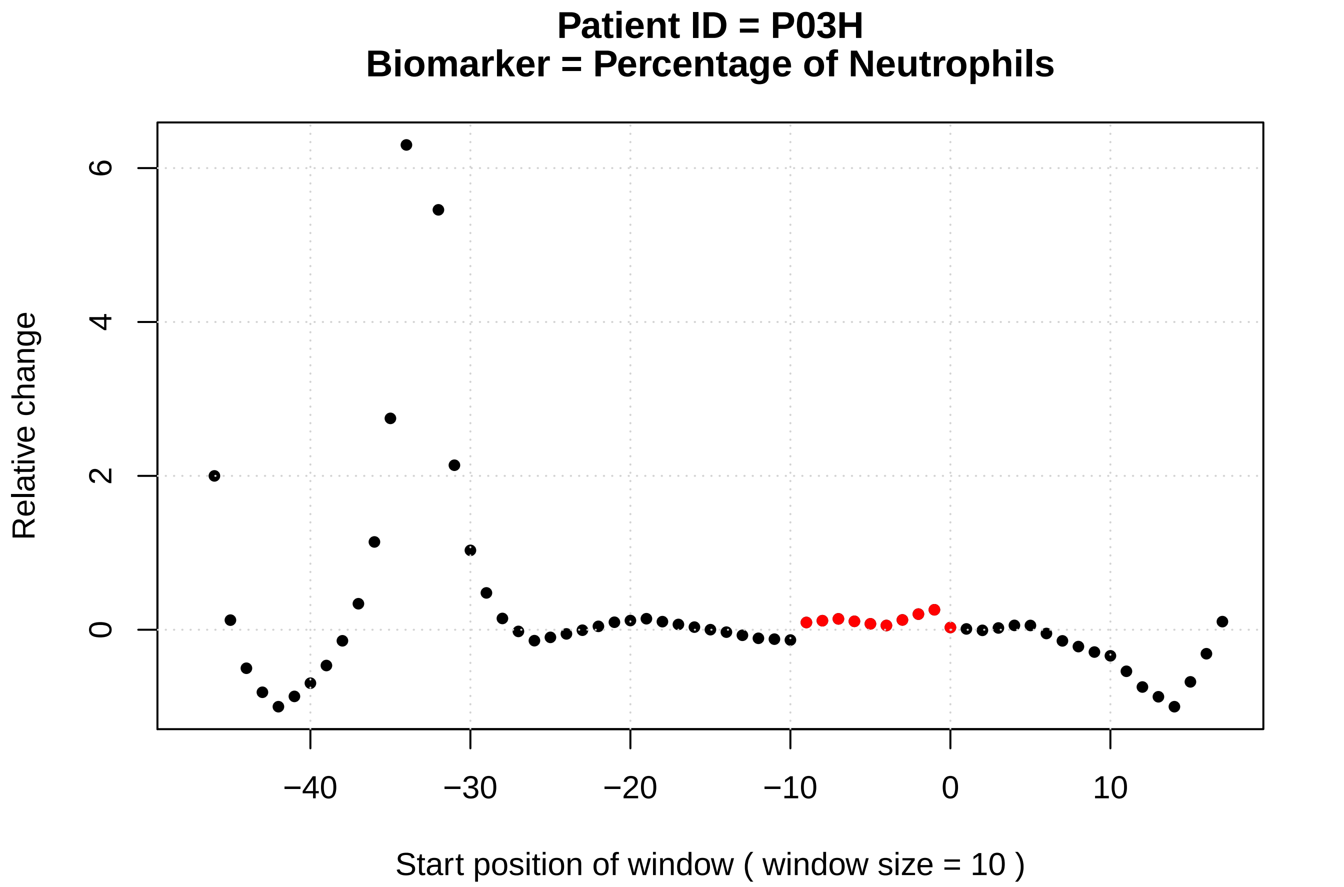
Patient ID = P03H
Biomarker = Percentage of Neutrophils
Relative change
0
2
4
6
−40
−30
−20
−10
0
10
Start position of window (window size = 10)

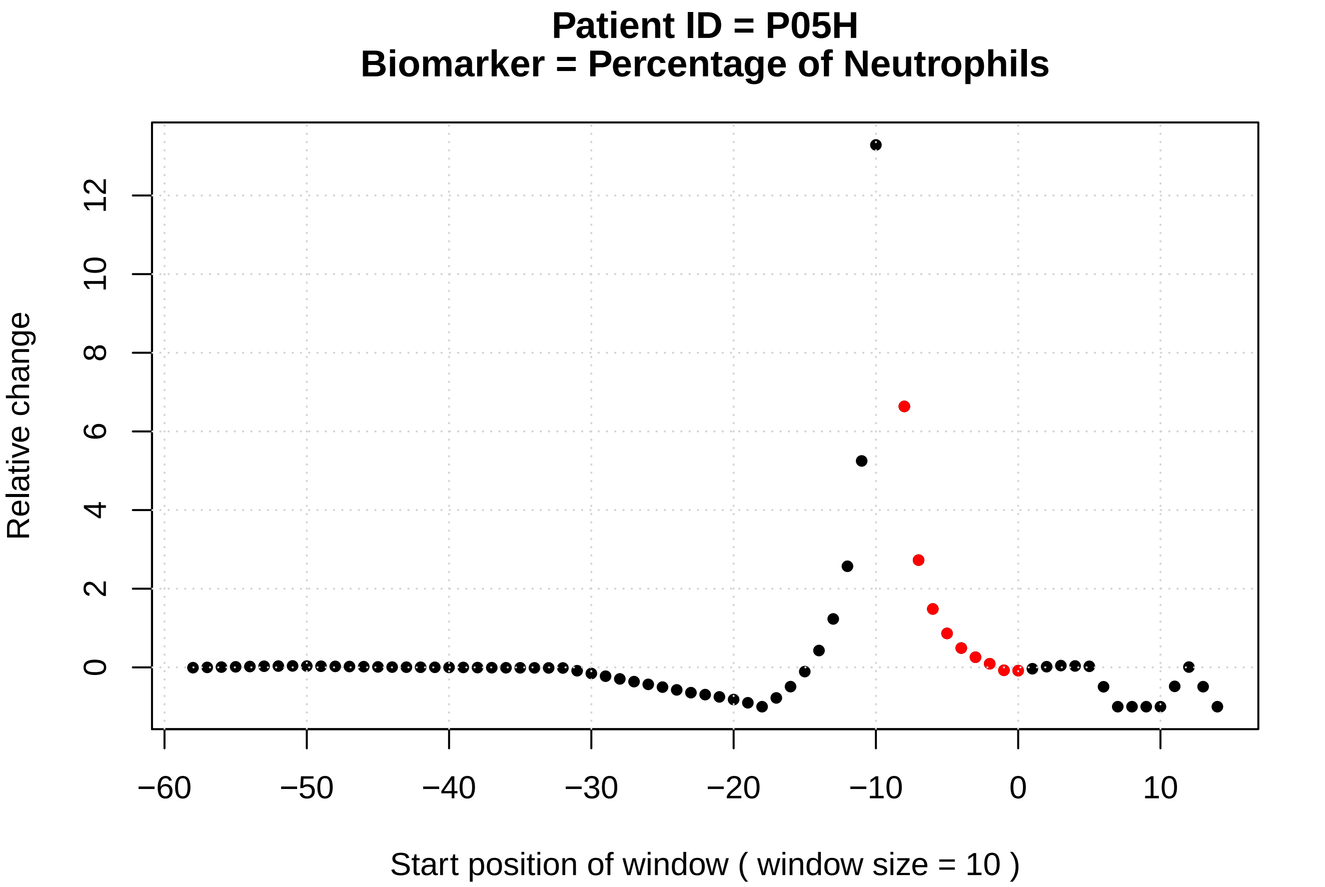
Patient ID = P05H
Biomarker = Percentage of Neutrophils
Relative change
0
2
4
6
8
10
12
−60
−50
−40
−30
−20
−10
0
10
Start position of window (window size = 10)

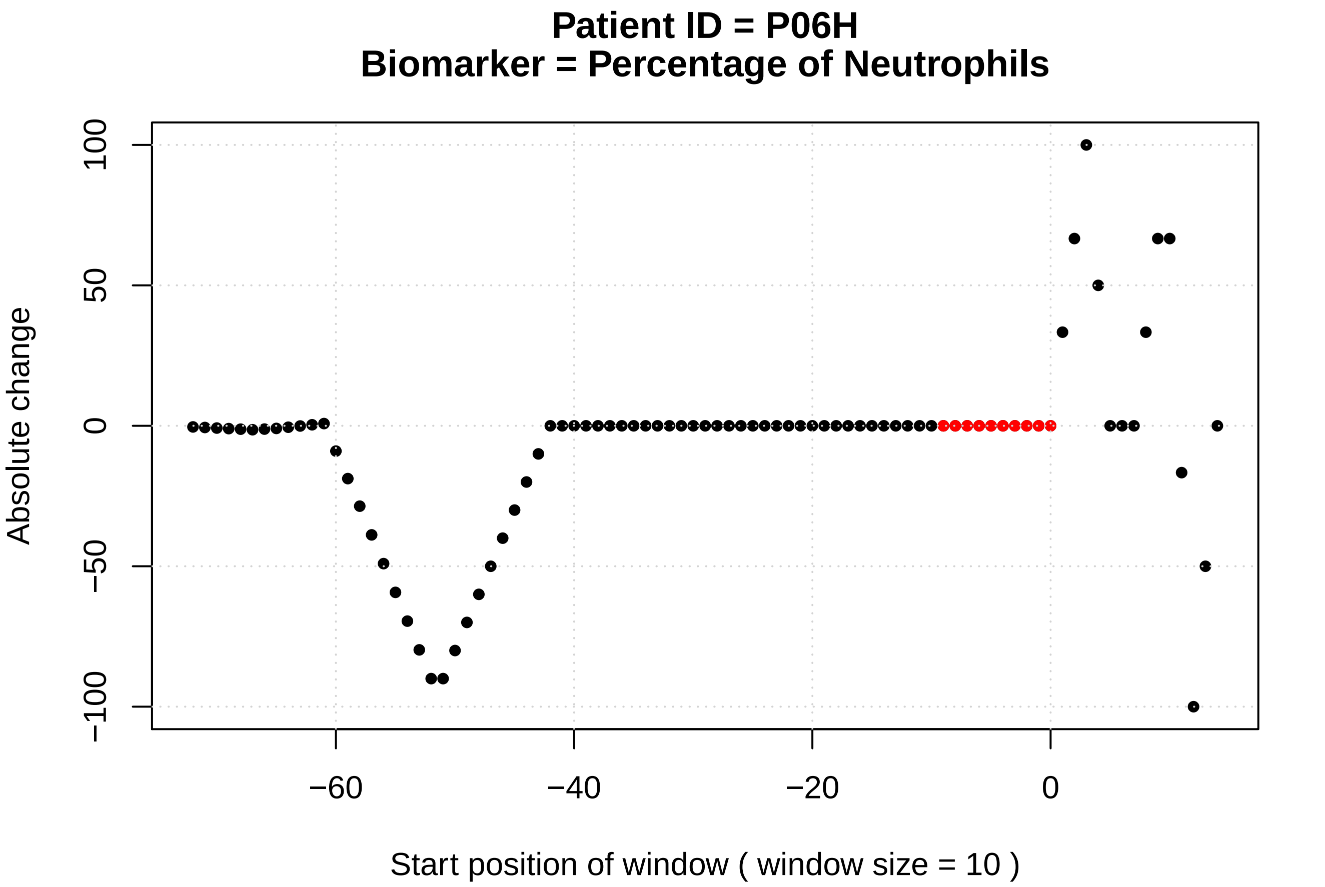

Patient ID = P06H
Biomarker = Percentage of Neutrophils
Absolute change
100
50
0
−50
−100
−60
−40
−20
0
Start position of window (window size = 10)

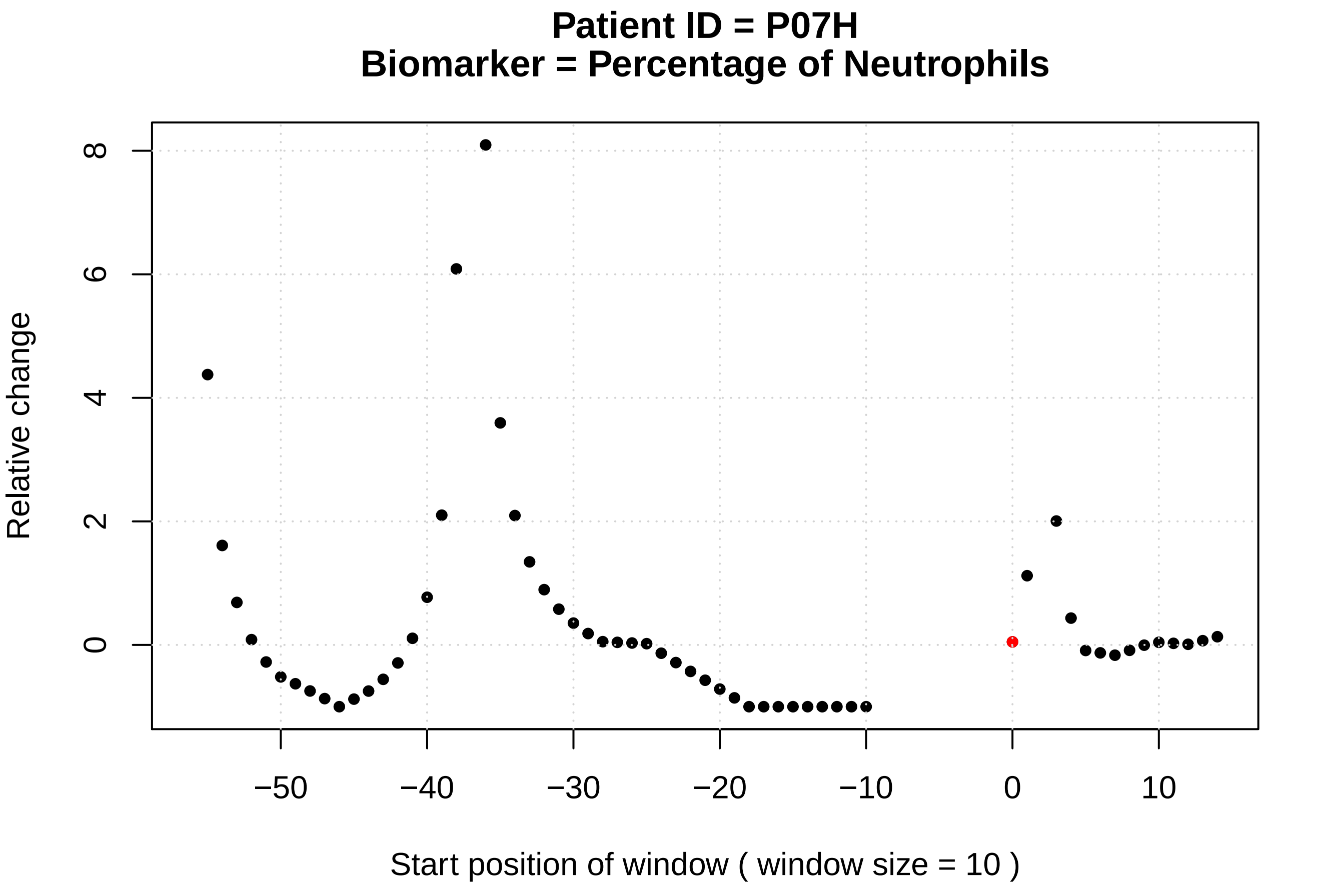
Patient ID = P07H
Biomarker = Percentage of Neutrophils
Relative change
0
2
4
6
8
−50
−40
−30
−20
−10
0
10
Start position of window (window size = 10)

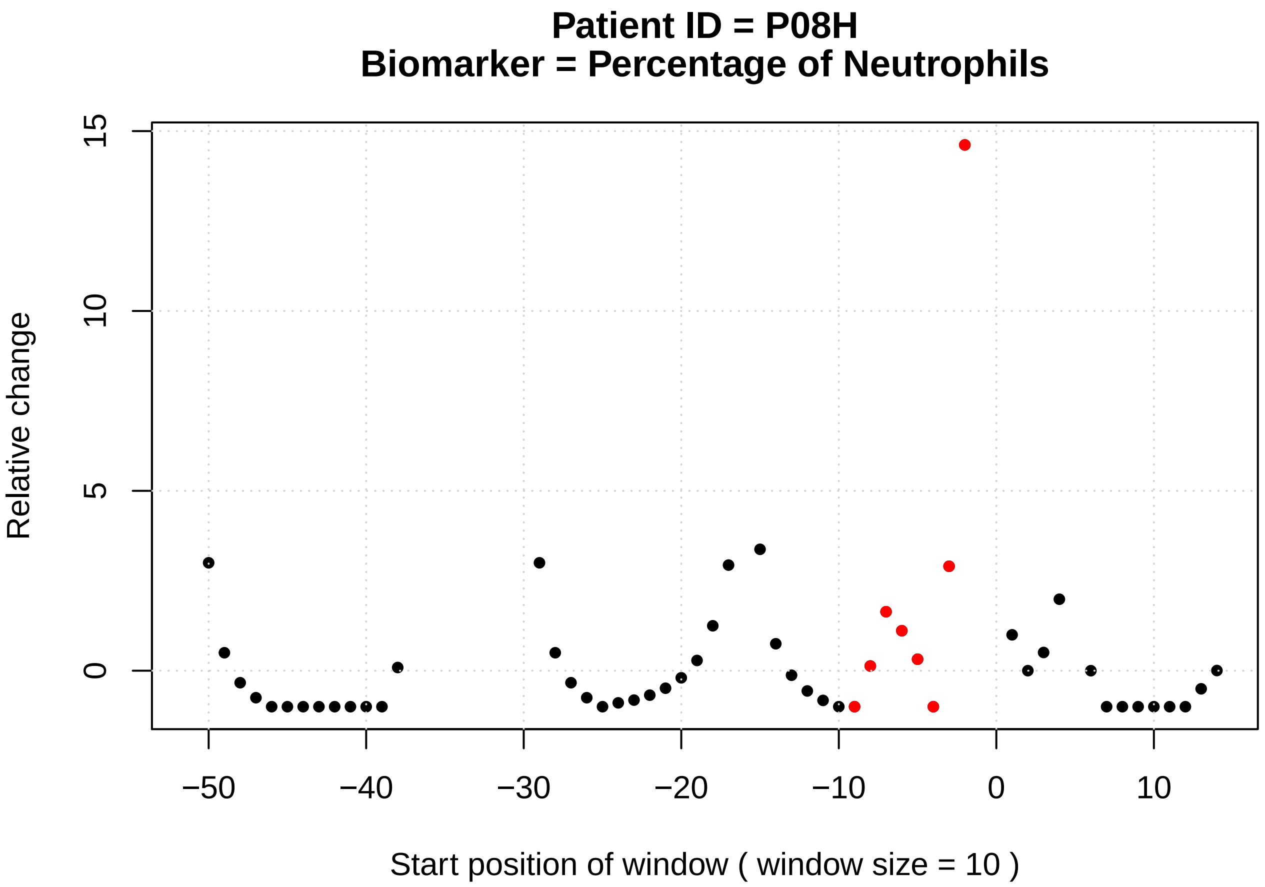

Patient ID = P08H
Biomarker = Percentage of Neutrophils
Relative change
0
5
10
15
−50
−40
−30
−20
−10
0
10
Start position of window (window size = 10)

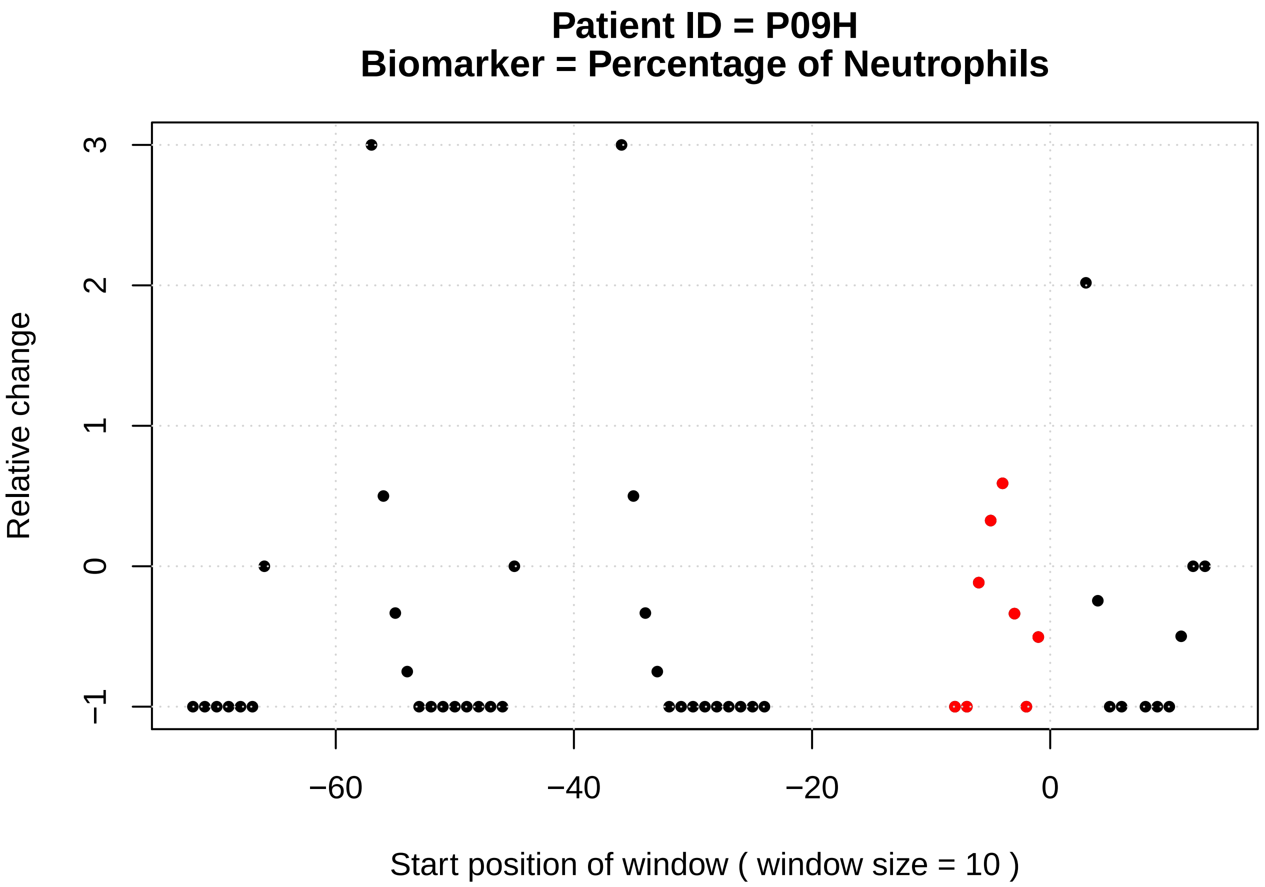
Patient ID = P09H
Biomarker = Percentage of Neutrophils
Relative change
3
2
1
0
−1
−60
−40
−20
0
Start position of window (window size = 10)

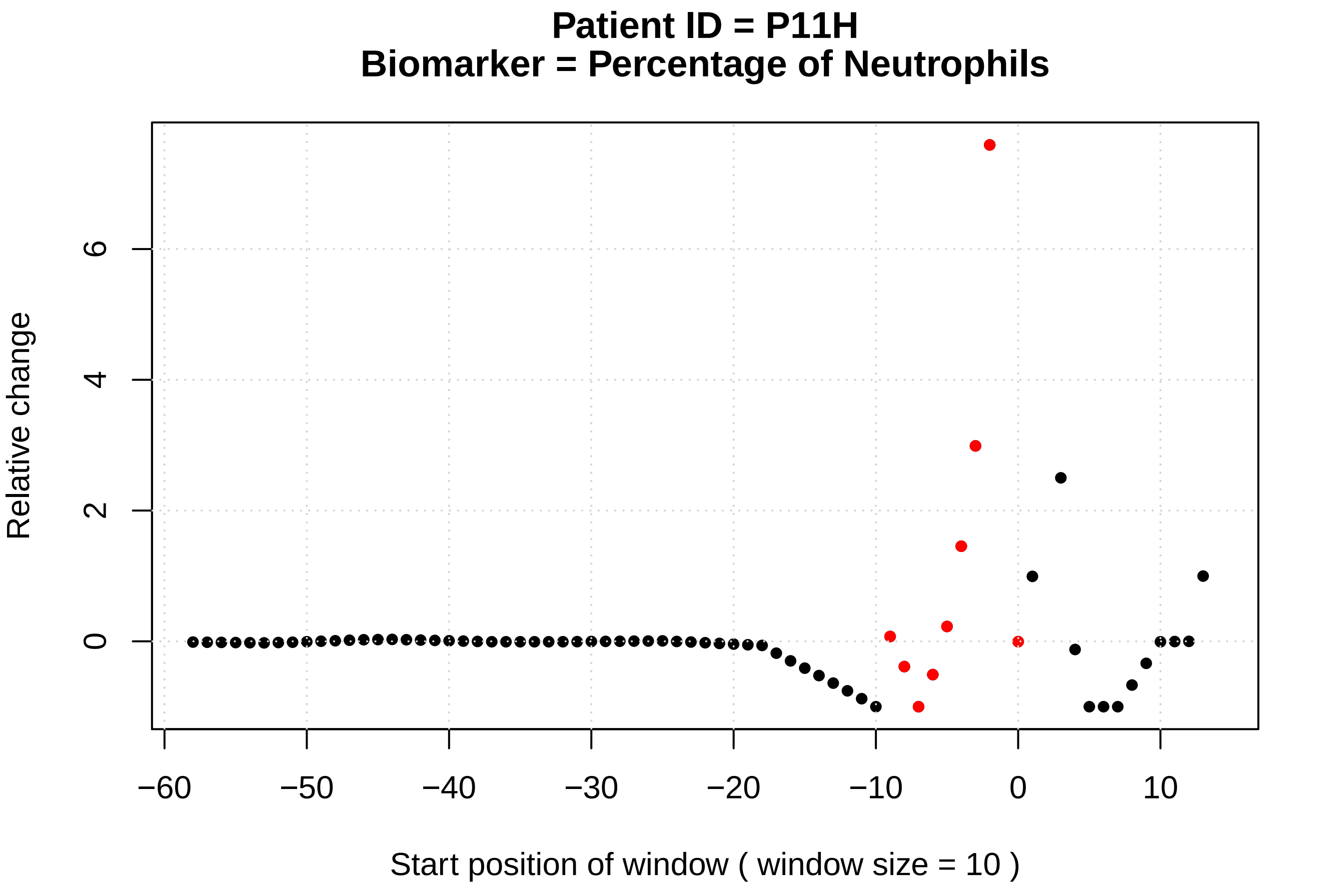

Patient ID = P11H
Biomarker = Percentage of Neutrophils
Relative change
0
2
4
6
−60
−50
−40
−30
−20
−10
0
10
Start position of window (window size = 10)

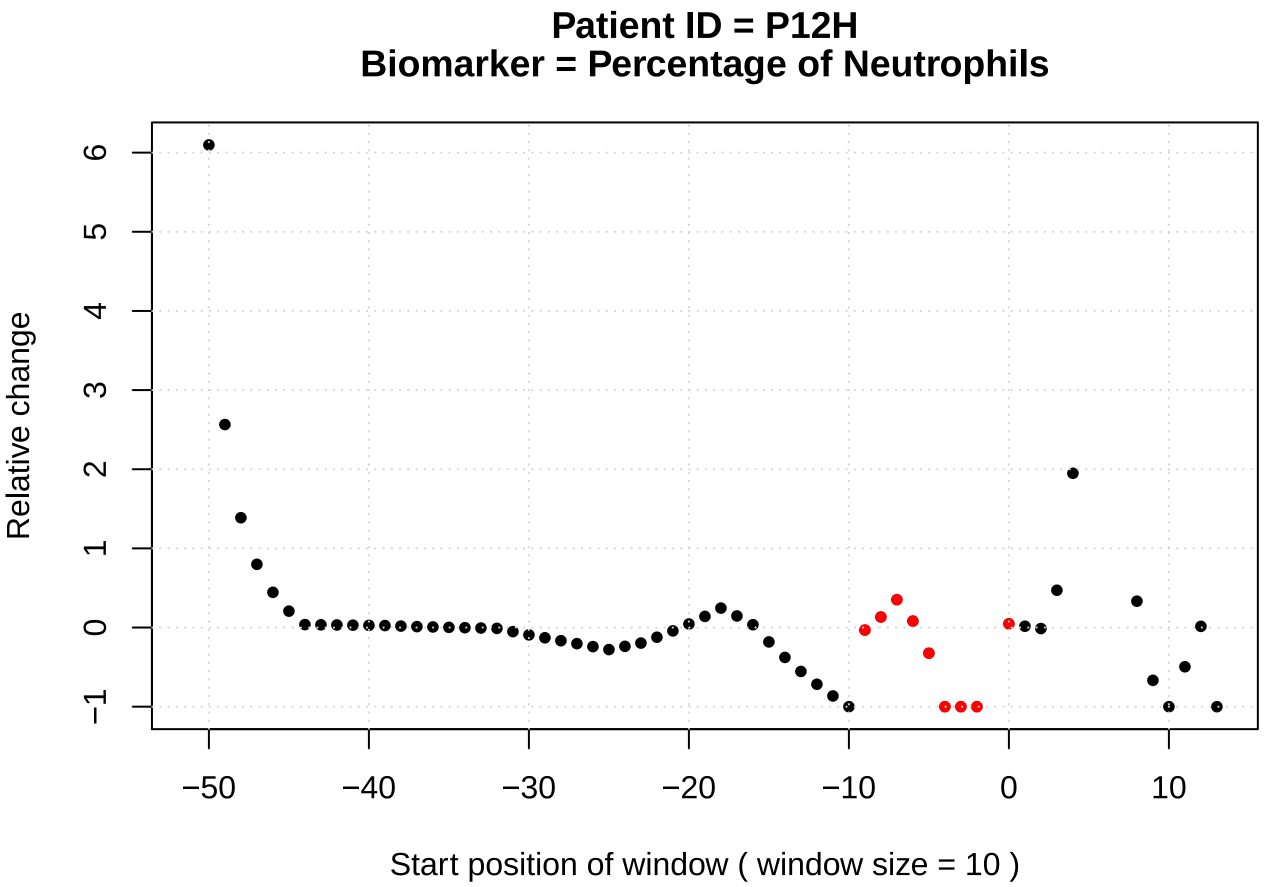

Patient ID = P12H
Biomarker = Percentage of Neutrophils
Relative change
Start position of window (window size = 10)
−1
0
1
2
3
4
5
6
−50
−40
−30
−20
−10
0
10

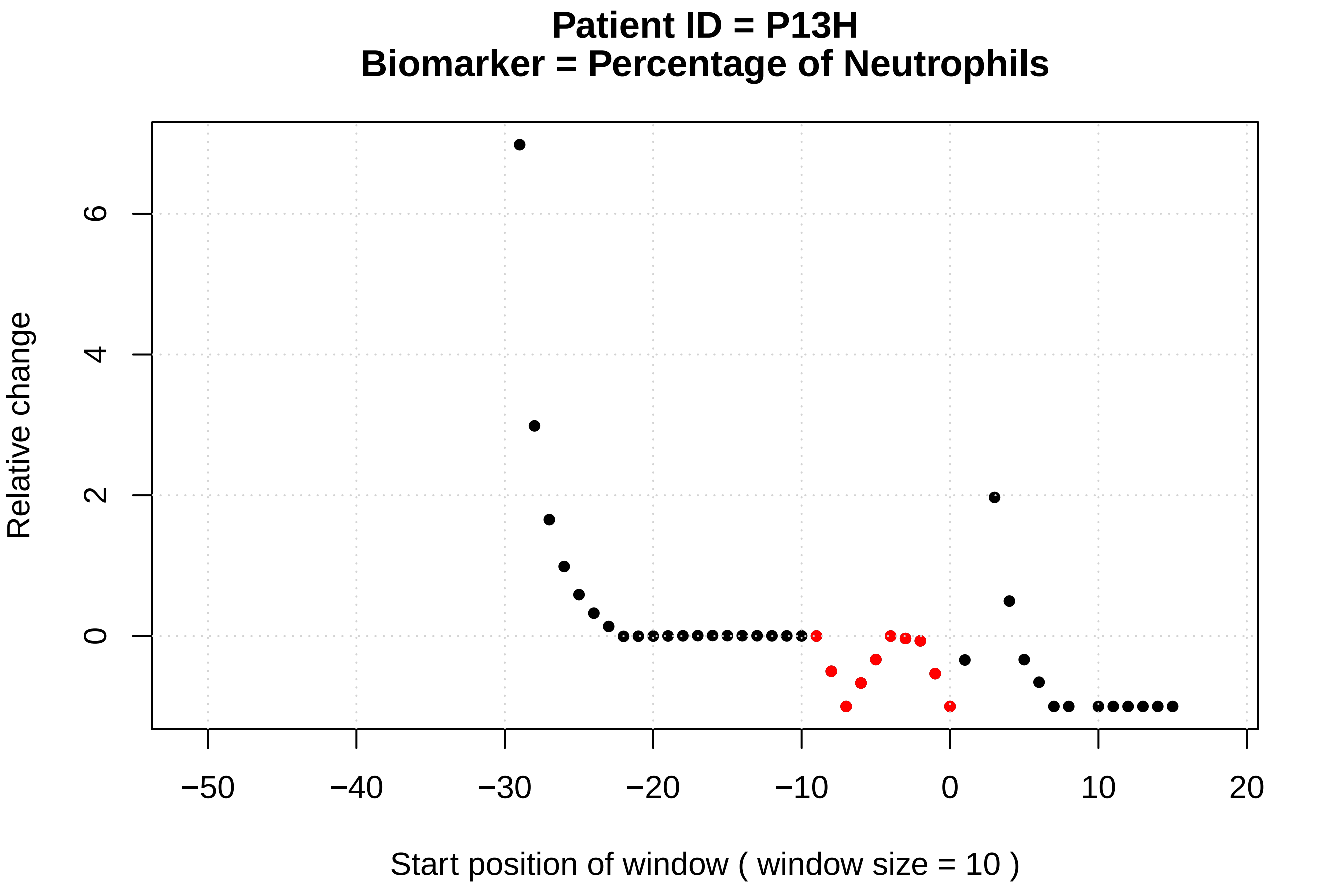

Patient ID = P13H
Biomarker = Percentage of Neutrophils
Relative change
0
2
4
6
−50
−40
−30
−20
−10
0
10
20
Start position of window (window size = 10)

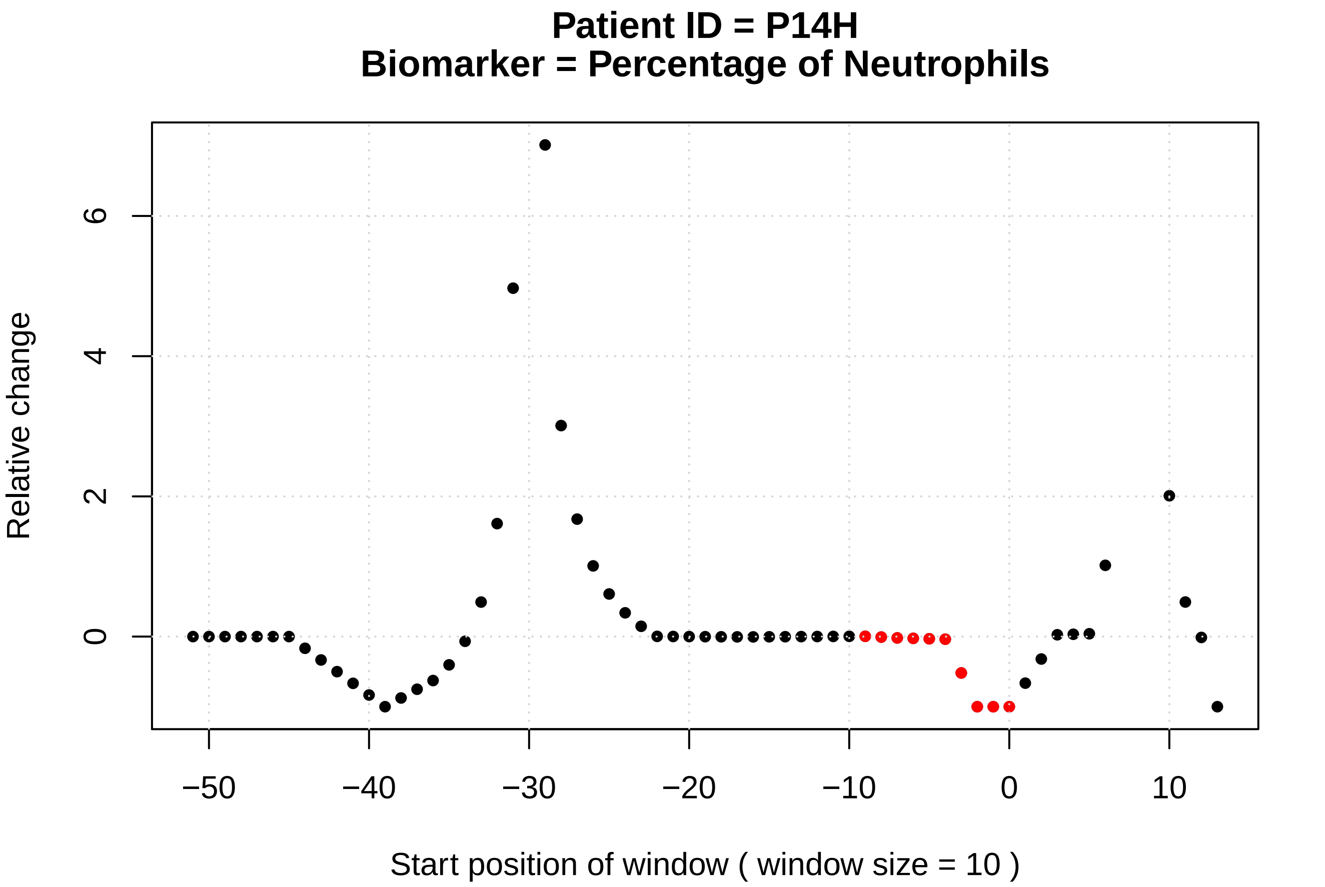
Patient ID = P14H
Biomarker = Percentage of Neutrophils
Relative change
0
2
4
6
−50
−40
−30
−20
−10
0
10
Start position of window (window size = 10)

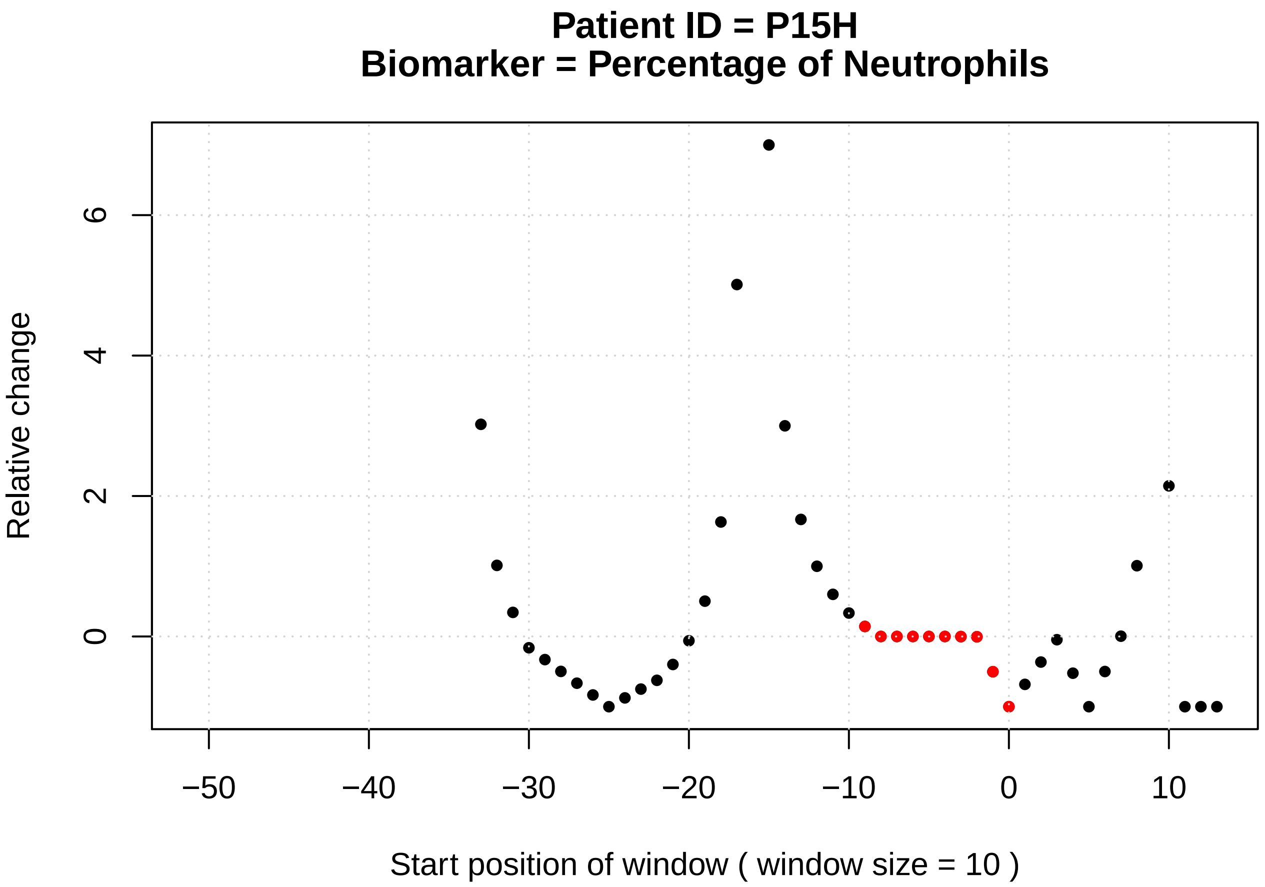
Patient ID = P15H
Biomarker = Percentage of Neutrophils
Relative change
0
2
4
6
−50
−40
−30
−20
−10
0
10
Start position of window (window size = 10)
