# Supplementary material for: Loss of adaptive capacity in asthmatic patients revealed by biomarker fluctuation dynamics after rhinovirus challenge
Source: eLife. 2019 Nov 5;8:e47969. doi: 10.7554/eLife.47969 (PMC6877087; doi:10.7554/eLife.47969)
Supplement: Supplementary file 1. [file elife-47969-supp1.zip › RelativeChangeWithinWindowPlots_PDFs/Appendix-figure SS30 IndividualWindowBoundariesRelativeChangeAnalysis PercentageOfEosinophils Window Size = 10 AsthmaticParticipants.pdf]

Patient ID = P01A
Biomarker = Percentage of Eosinophils

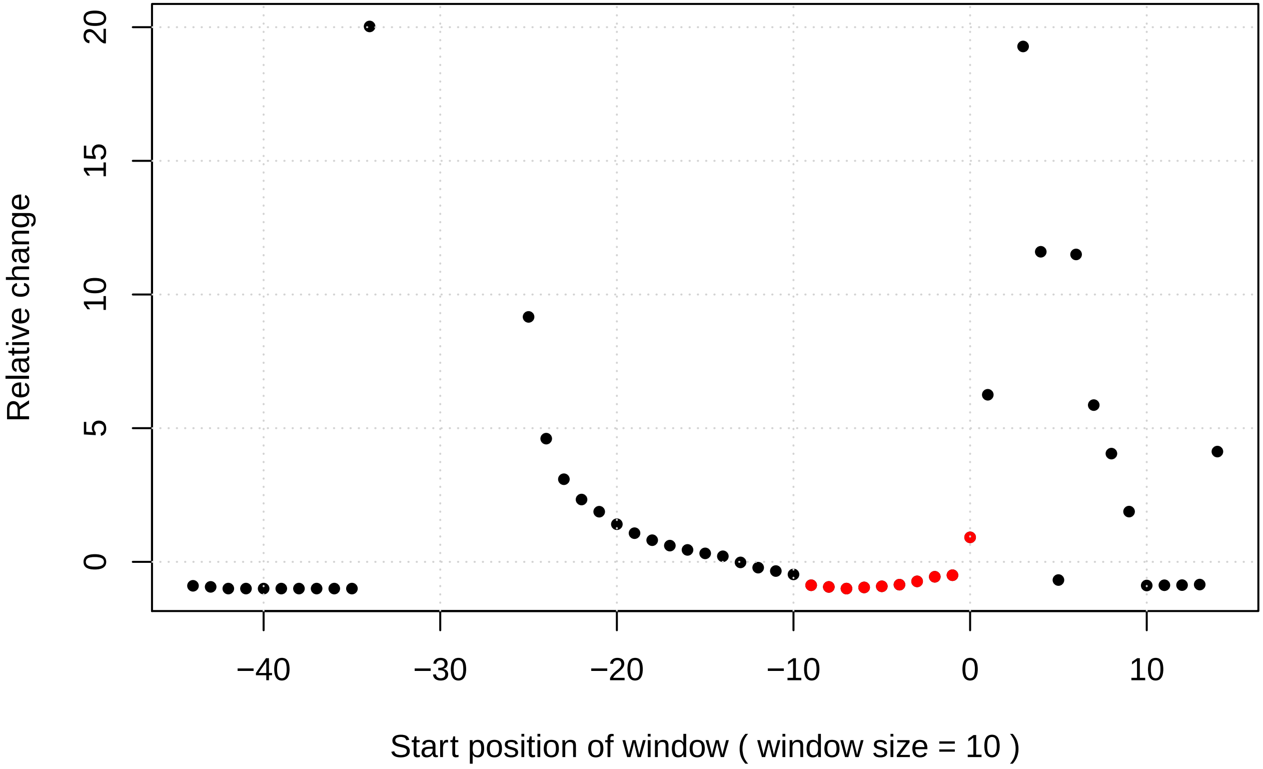

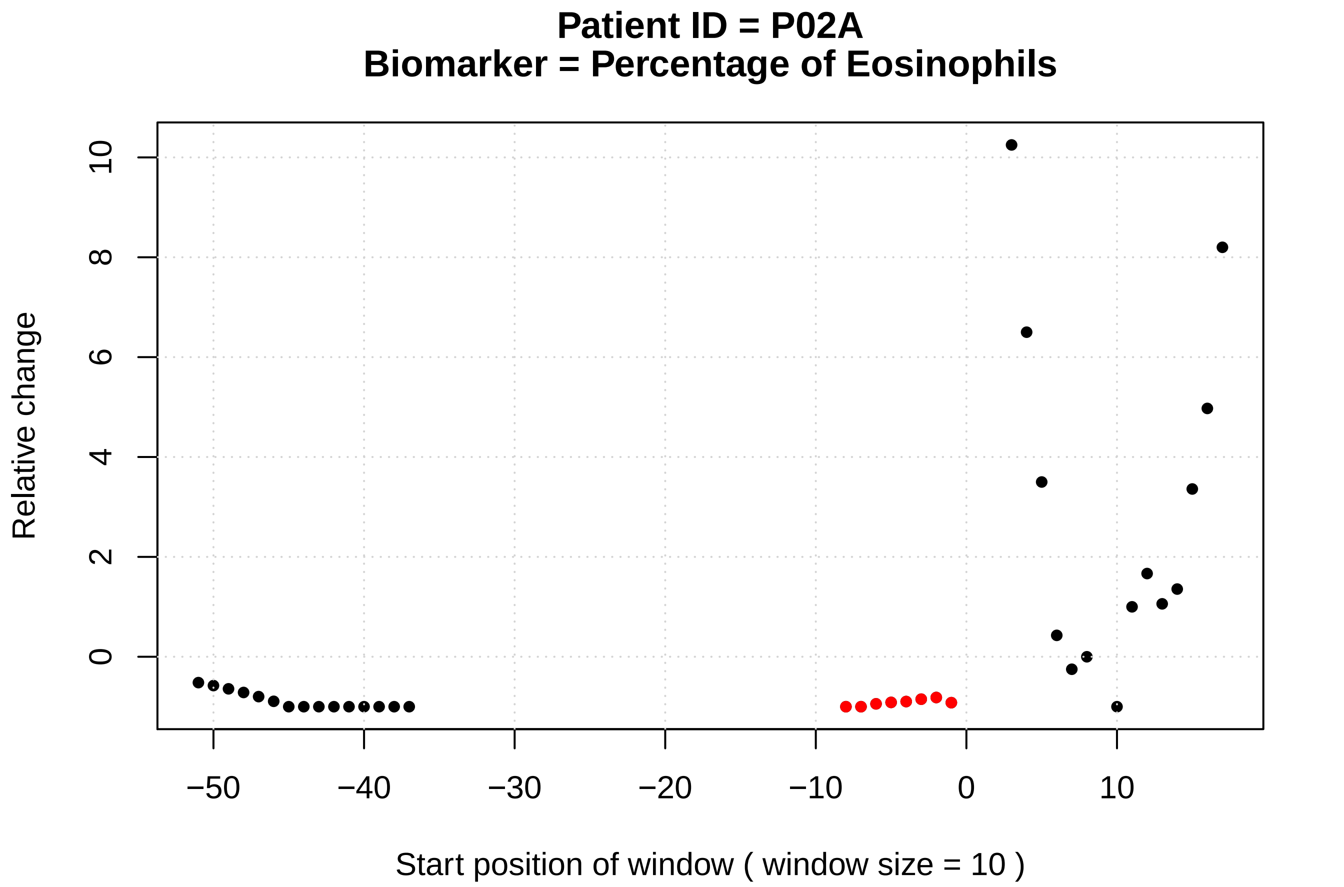
Patient ID = P02A
Biomarker = Percentage of Eosinophils
Relative change
0
2
4
6
8
10
−50
−40
−30
−20
−10
0
10
Start position of window (window size = 10)

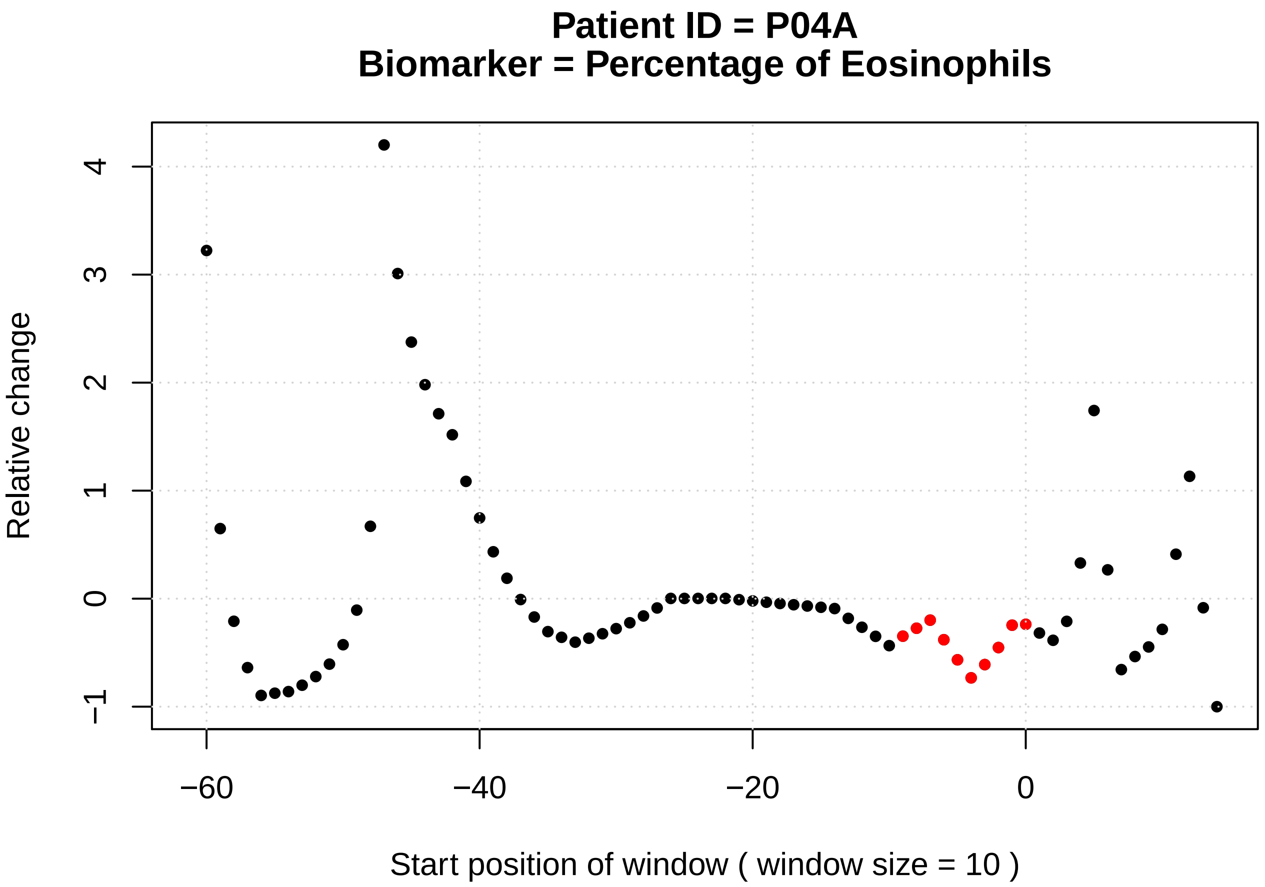
Patient ID = P04A
Biomarker = Percentage of Eosinophils
Relative change
4
3
2
1
0
−1
−60
−40
−20
0
Start position of window (window size = 10)

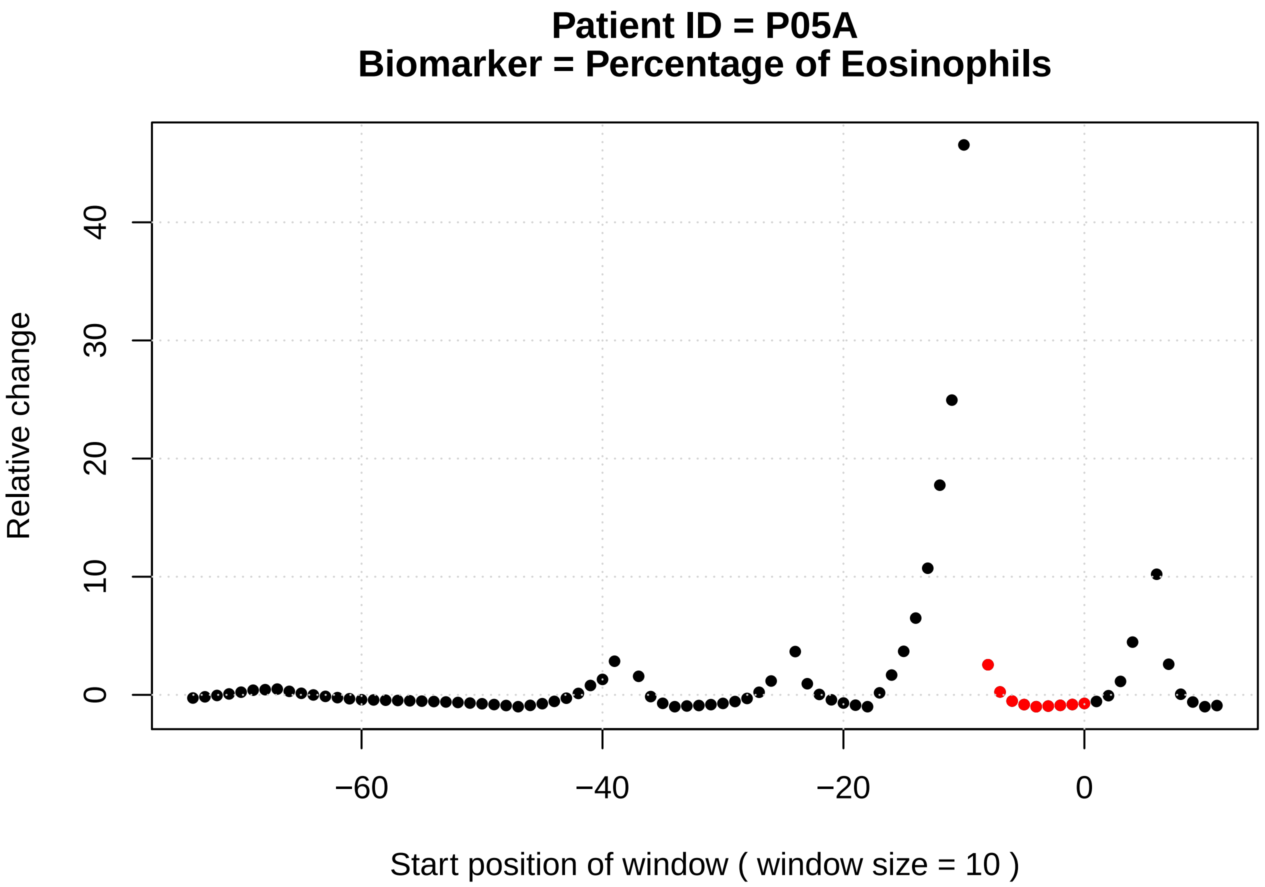
Patient ID = P05A
Biomarker = Percentage of Eosinophils
Relative change
0
10
20
30
40
−60
−40
−20
0
Start position of window (window size = 10)

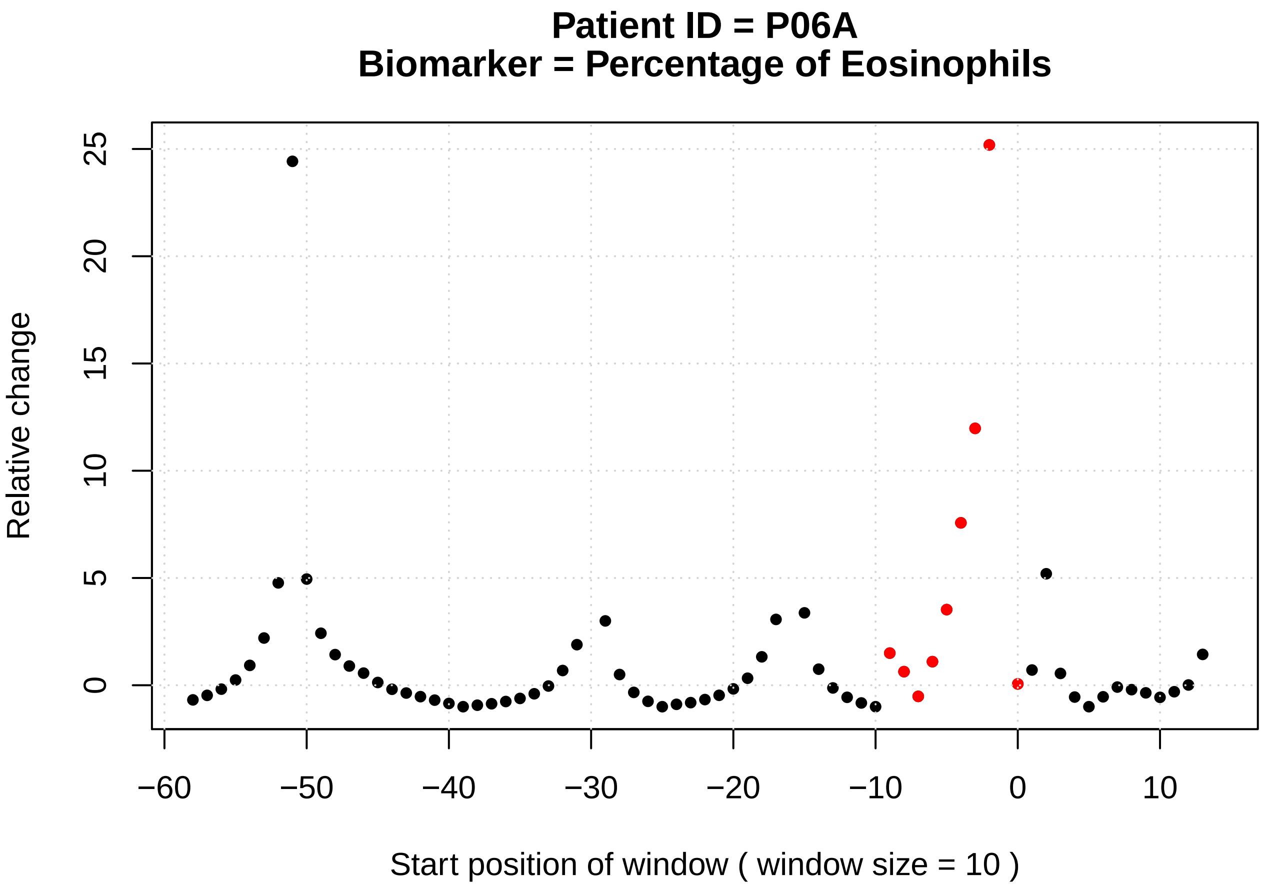
Patient ID = P06A
Biomarker = Percentage of Eosinophils
Relative change
0
5
10
15
20
25
−60
−50
−40
−30
−20
−10
0
10
Start position of window (window size = 10)

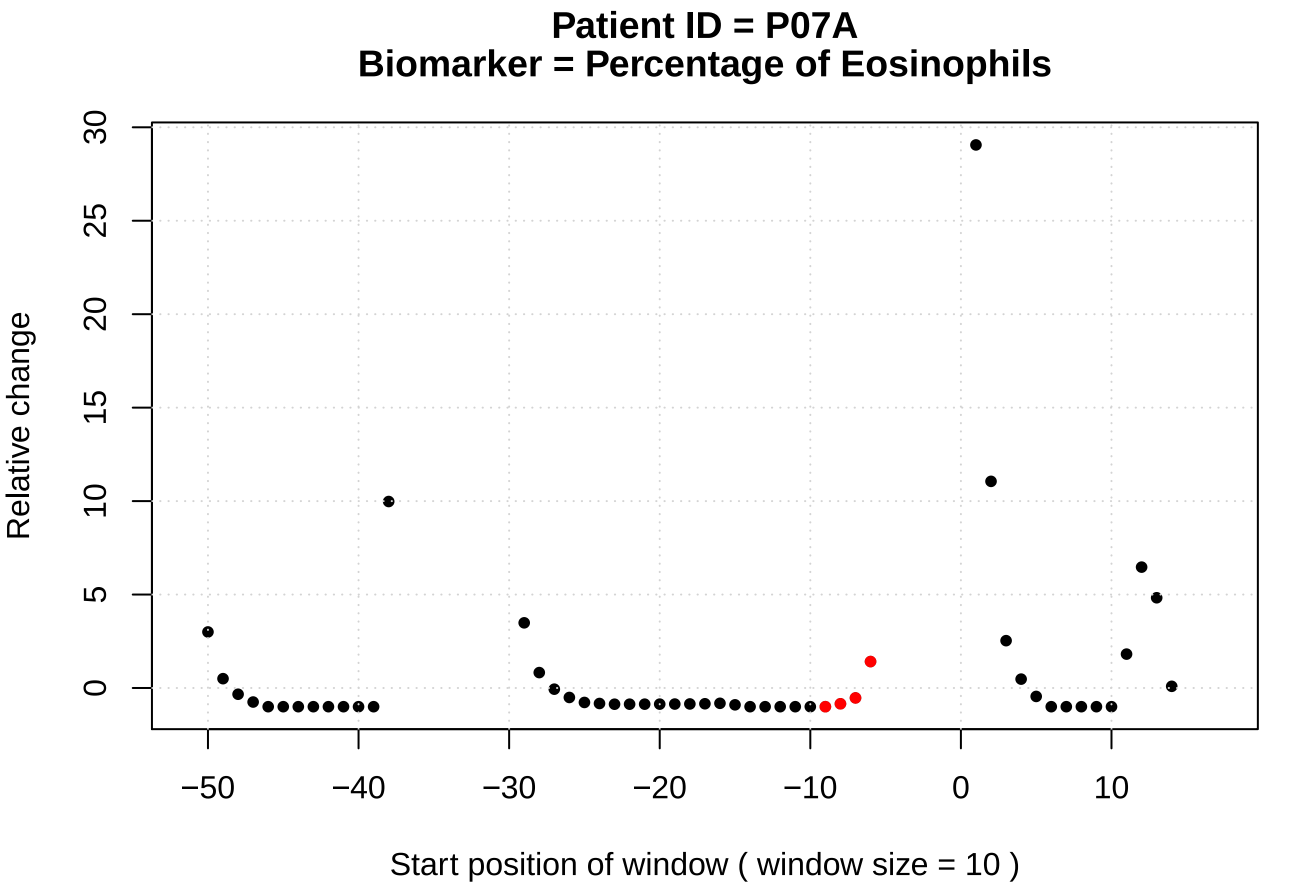
Patient ID = P07A
Biomarker = Percentage of Eosinophils
Relative change
0
5
10
15
20
25
30
−50
−40
−30
−20
−10
0
10
Start position of window (window size = 10)

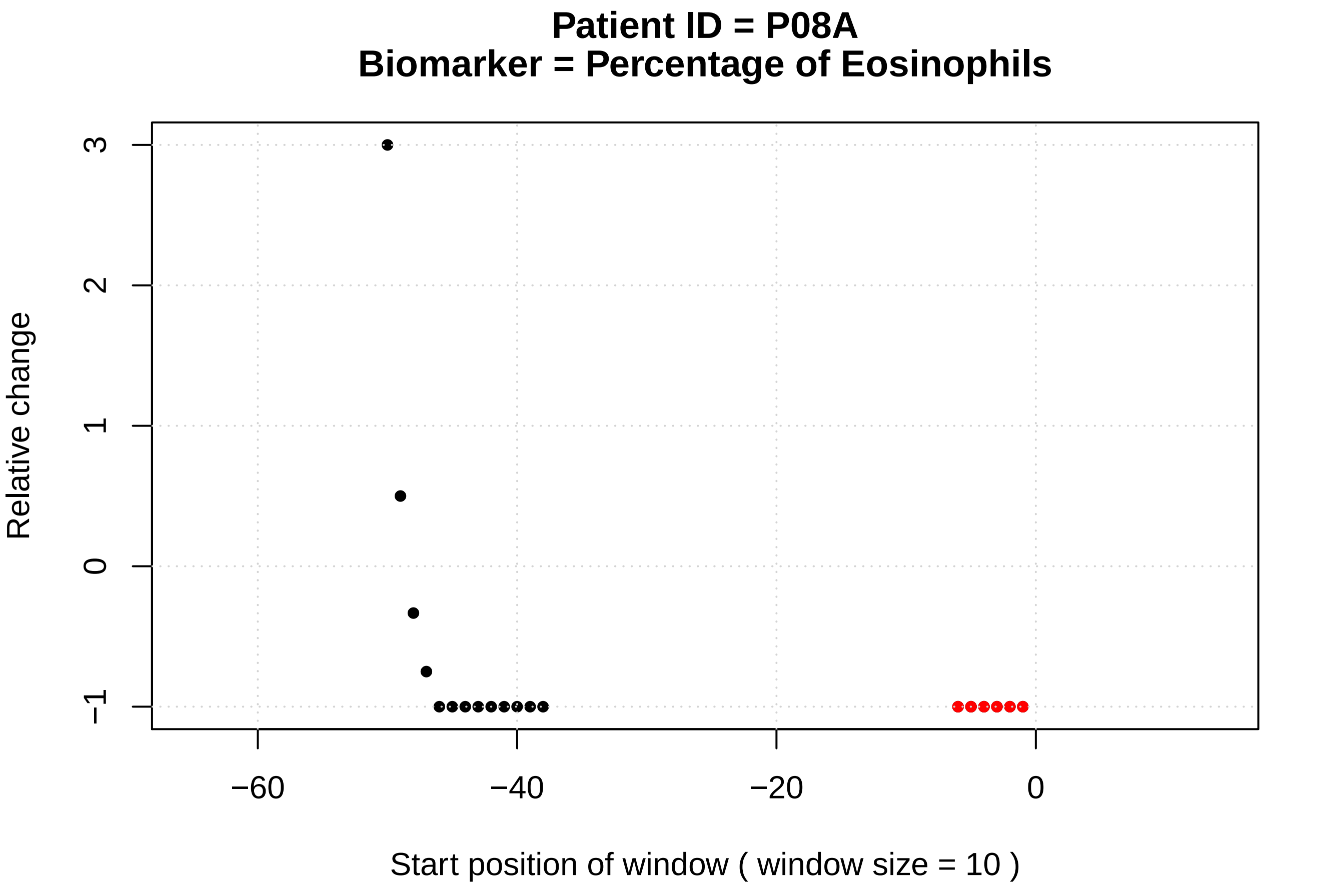
Patient ID = P08A
Biomarker = Percentage of Eosinophils
Relative change
3
2
1
0
−1
−60
−40
−20
0
Start position of window (window size = 10)

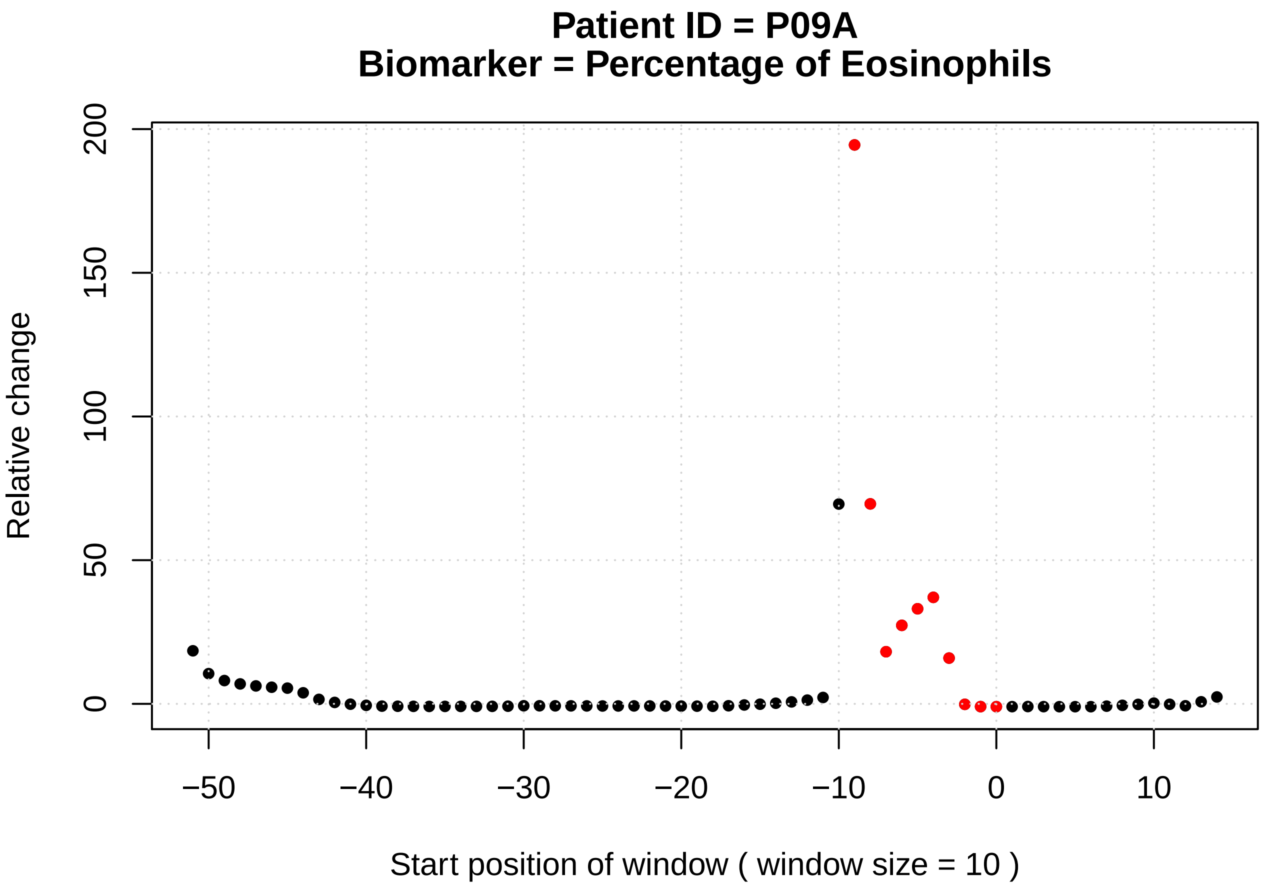
Patient ID = P09A
Biomarker = Percentage of Eosinophils
Relative change
0
50
100
150
200
−50
−40
−30
−20
−10
0
10
Start position of window (window size = 10)

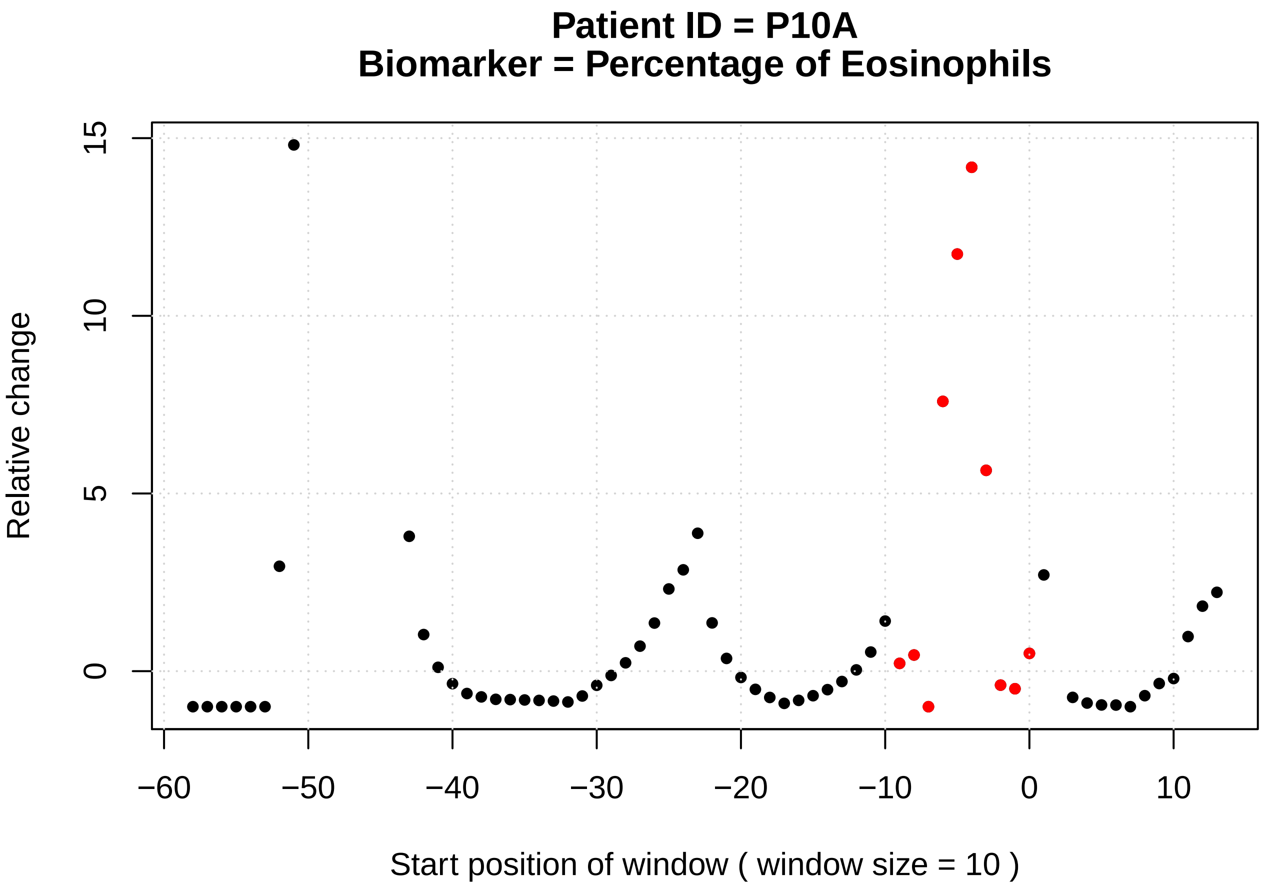
Patient ID = P10A
Biomarker = Percentage of Eosinophils
Relative change
0
5
10
15
−60
−50
−40
−30
−20
−10
0
10
Start position of window (window size = 10)

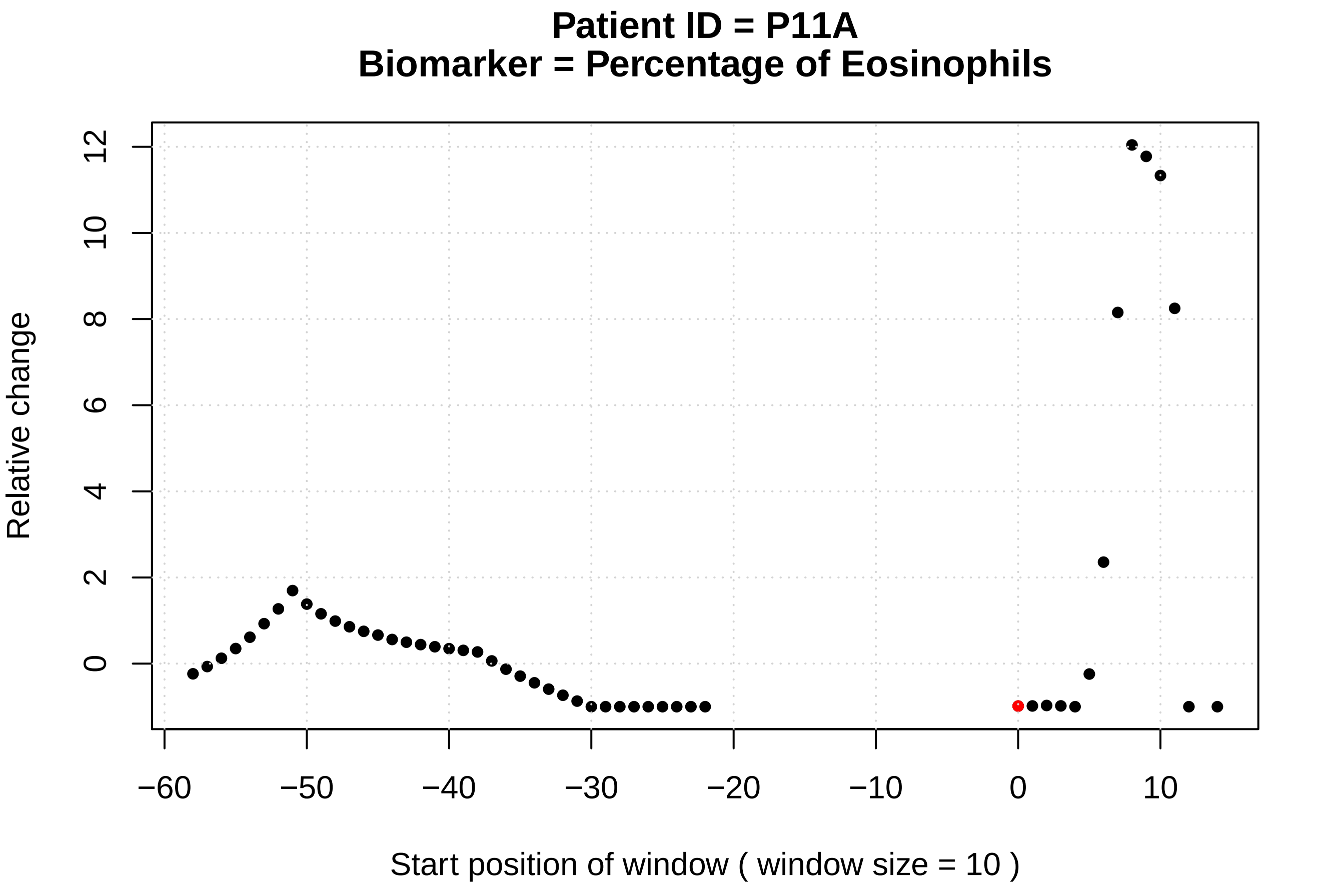
Patient ID = P11A
Biomarker = Percentage of Eosinophils
Relative change
0
2
4
6
8
10
12
−60
−50
−40
−30
−20
−10
0
10
Start position of window (window size = 10)

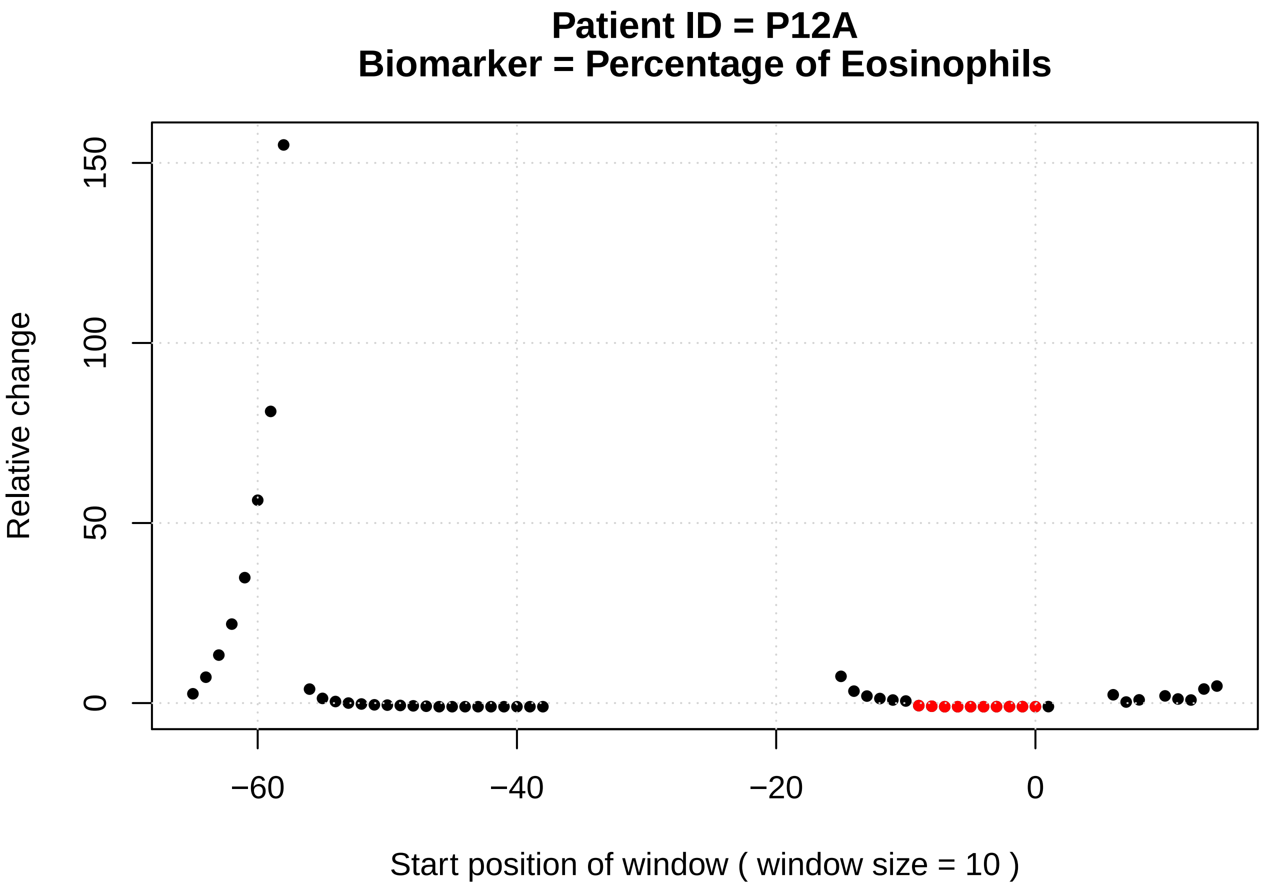
Patient ID = P12A
Biomarker = Percentage of Eosinophils
Relative change
0
50
100
150
−60
−40
−20
0
Start position of window (window size = 10)

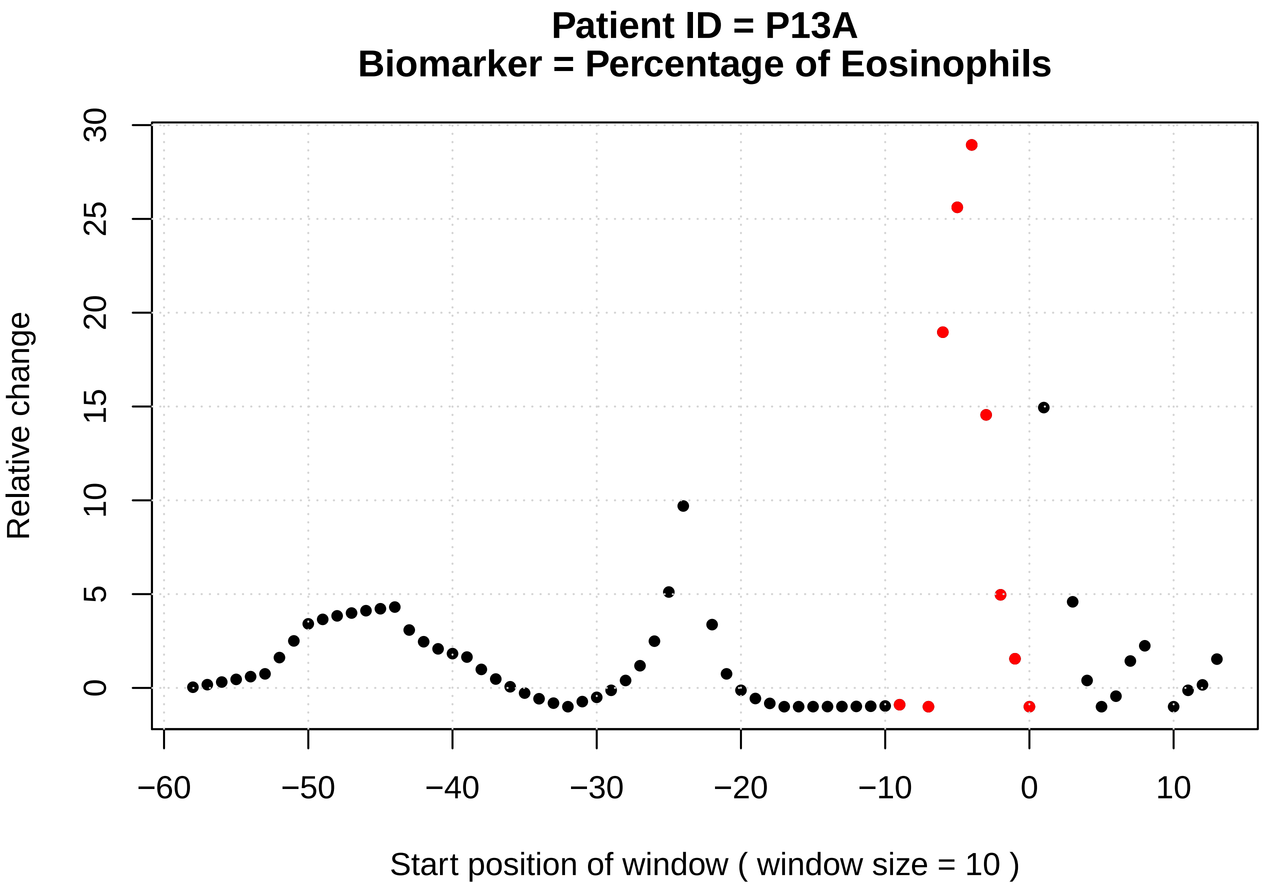

Patient ID = P13A
Biomarker = Percentage of Eosinophils
Relative change
0
5
10
15
20
25
30
−60
−50
−40
−30
−20
−10
0
10
Start position of window (window size = 10)
