# Supplementary material for: Loss of adaptive capacity in asthmatic patients revealed by biomarker fluctuation dynamics after rhinovirus challenge
Source: eLife. 2019 Nov 5;8:e47969. doi: 10.7554/eLife.47969 (PMC6877087; doi:10.7554/eLife.47969)
Supplement: Supplementary file 1. [file elife-47969-supp1.zip › RelativeChangeWithinWindowPlots_PDFs/Appendix-figure SS29 IndividualWindowBoundariesRelativeChangeAnalysis PercentageOfEosinophils Window Size = 10 HealthyParticipants.pdf]

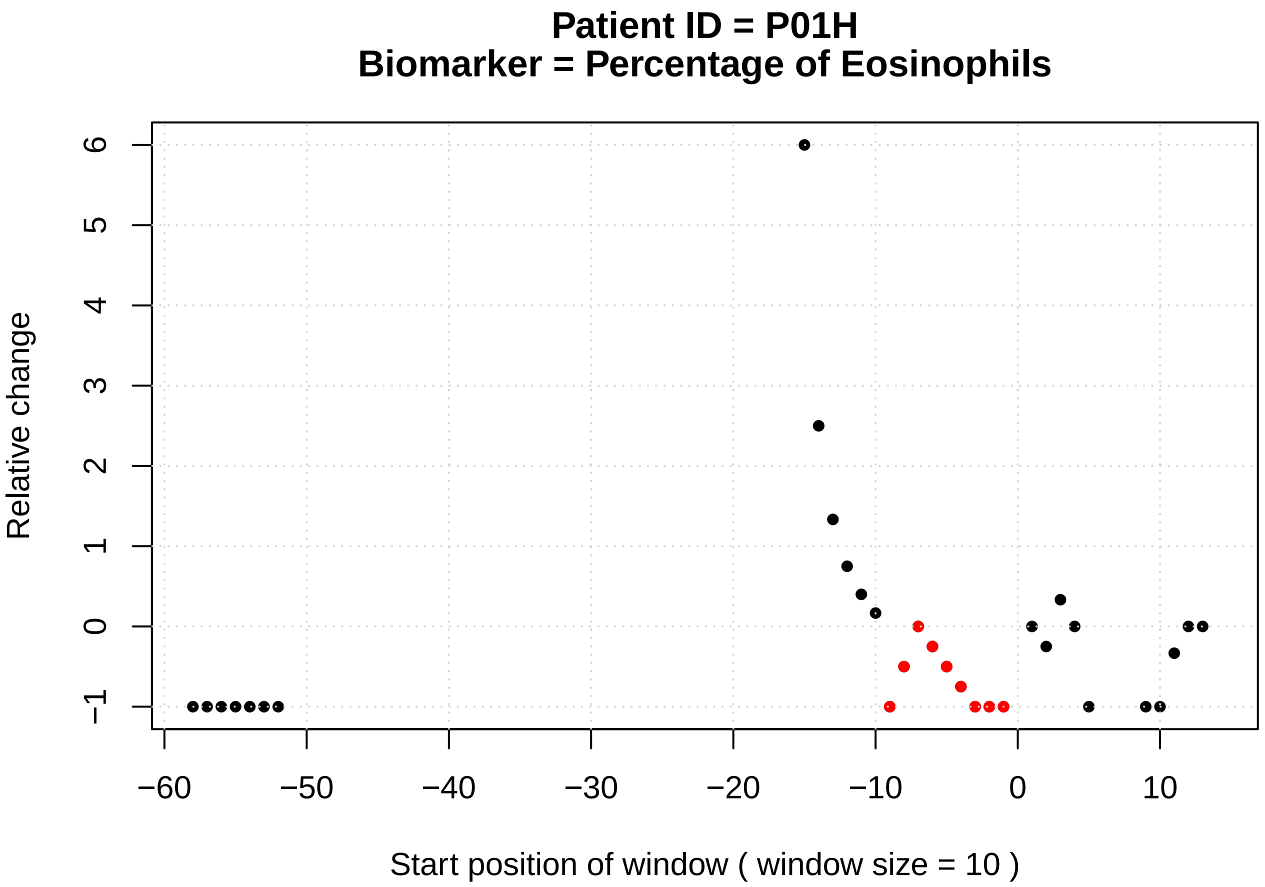
Patient ID = P01H
Biomarker = Percentage of Eosinophils
Relative change
6
5
4
3
2
1
0
−1
−60
−50
−40
−30
−20
−10
0
10
Start position of window ( window size = 10 )

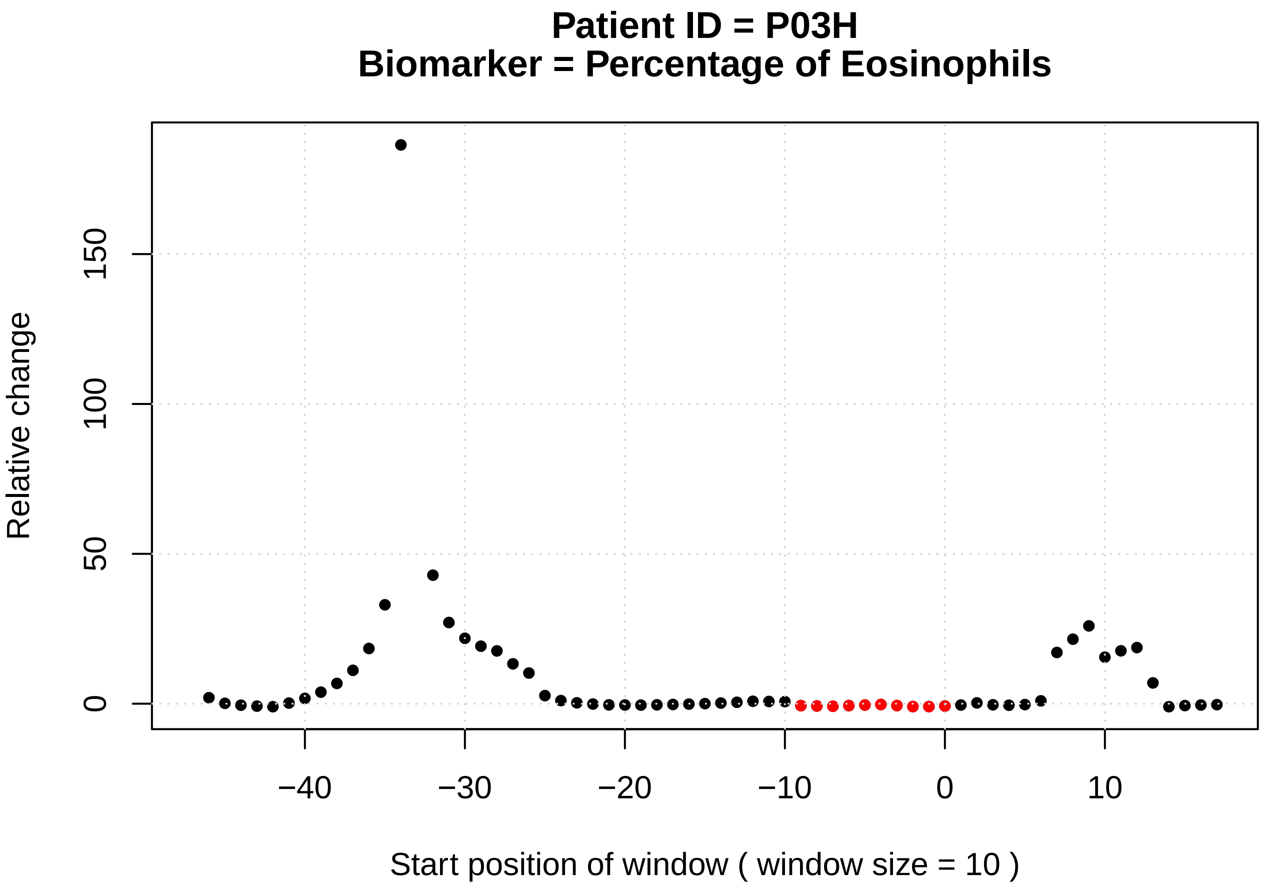

Patient ID = P03H
Biomarker = Percentage of Eosinophils
Relative change
0
50
100
150
−40
−30
−20
−10
0
10
Start position of window ( window size = 10 )

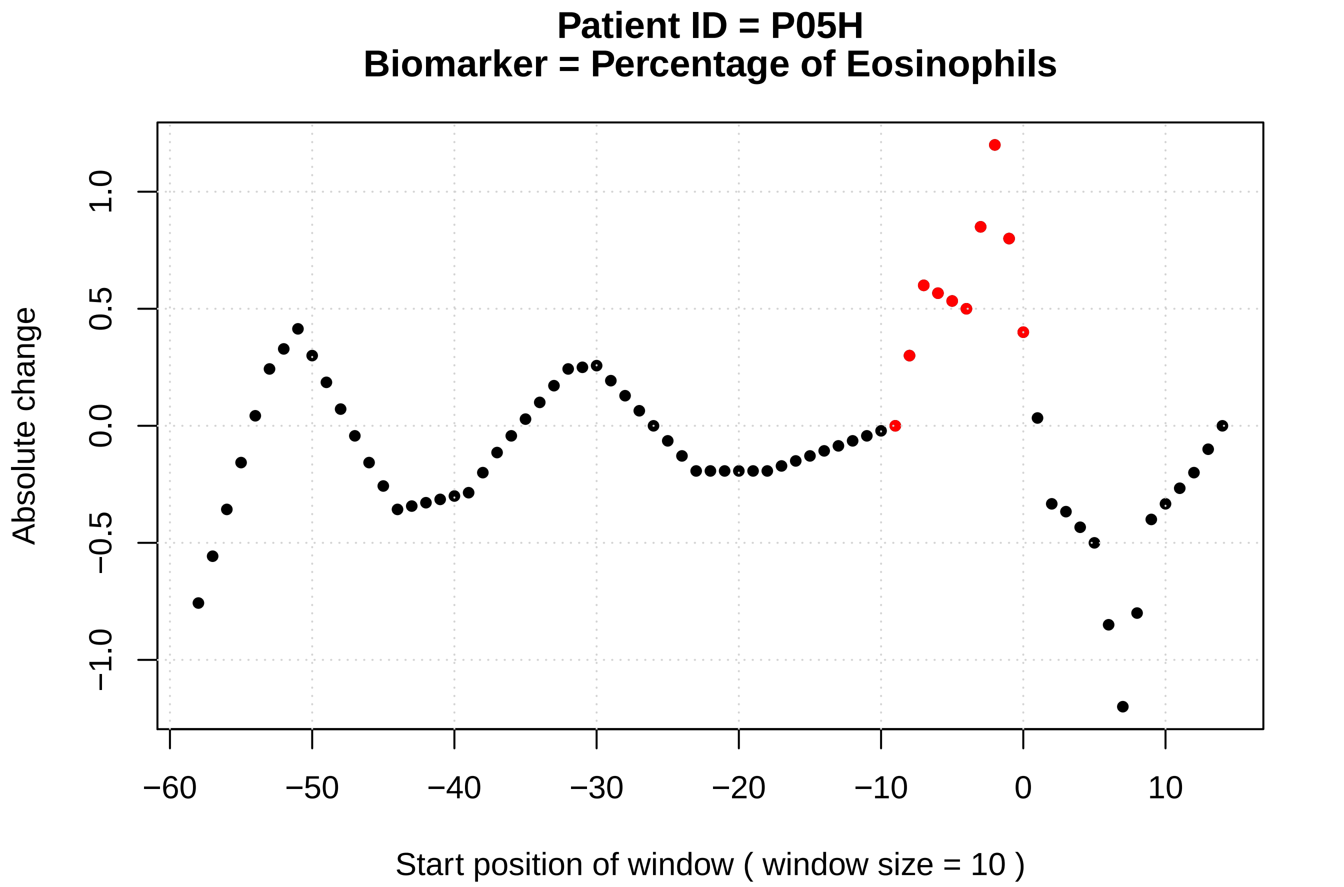

Patient ID = P05H
Biomarker = Percentage of Eosinophils
Absolute change
Start position of window ( window size = 10 )
1.0
0.5
0.0
−0.5
−1.0
−60
−50
−40
−30
−20
−10
0
10

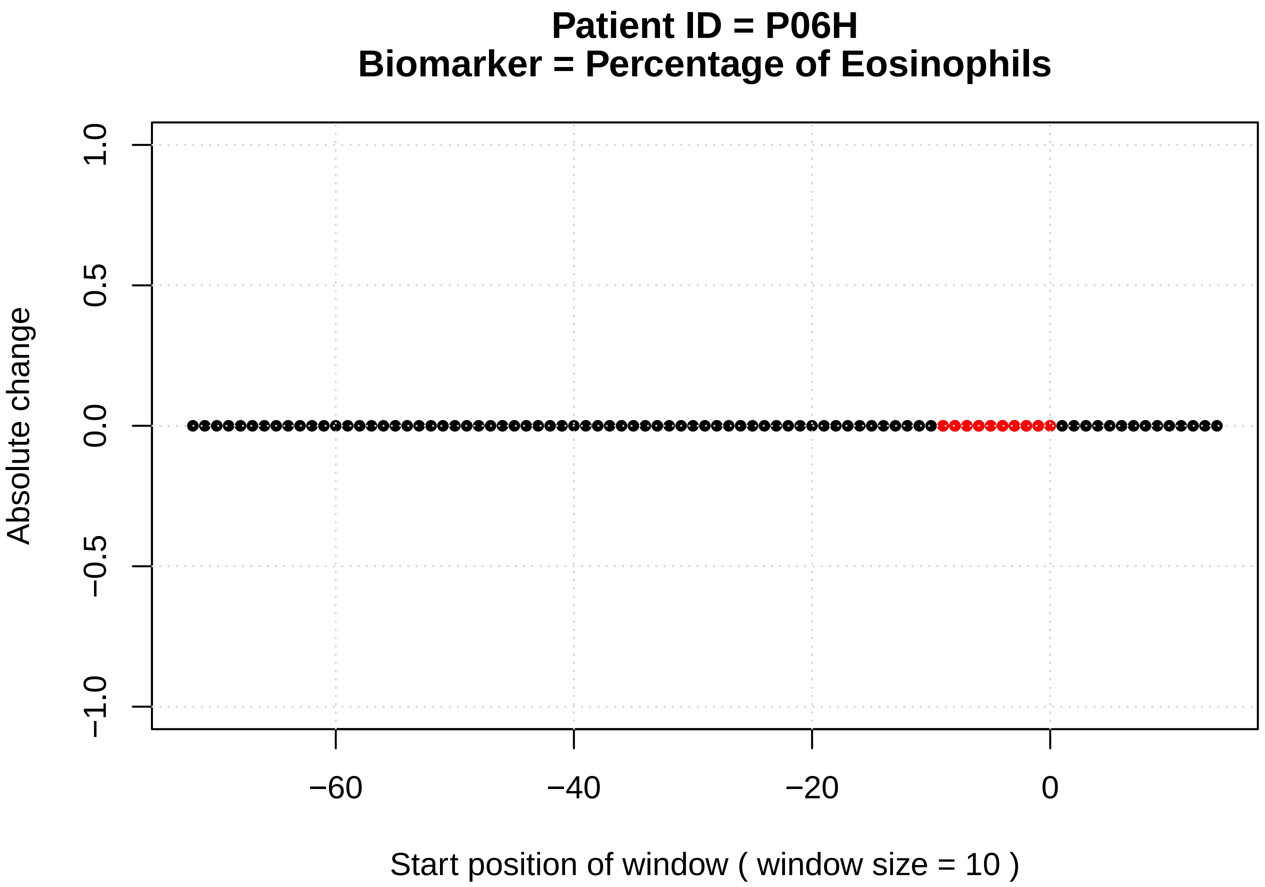
Patient ID = P06H
Biomarker = Percentage of Eosinophils
Absolute change
1.0
0.5
0.0
−0.5
−1.0
−60
−40
−20
0
Start position of window ( window size = 10 )

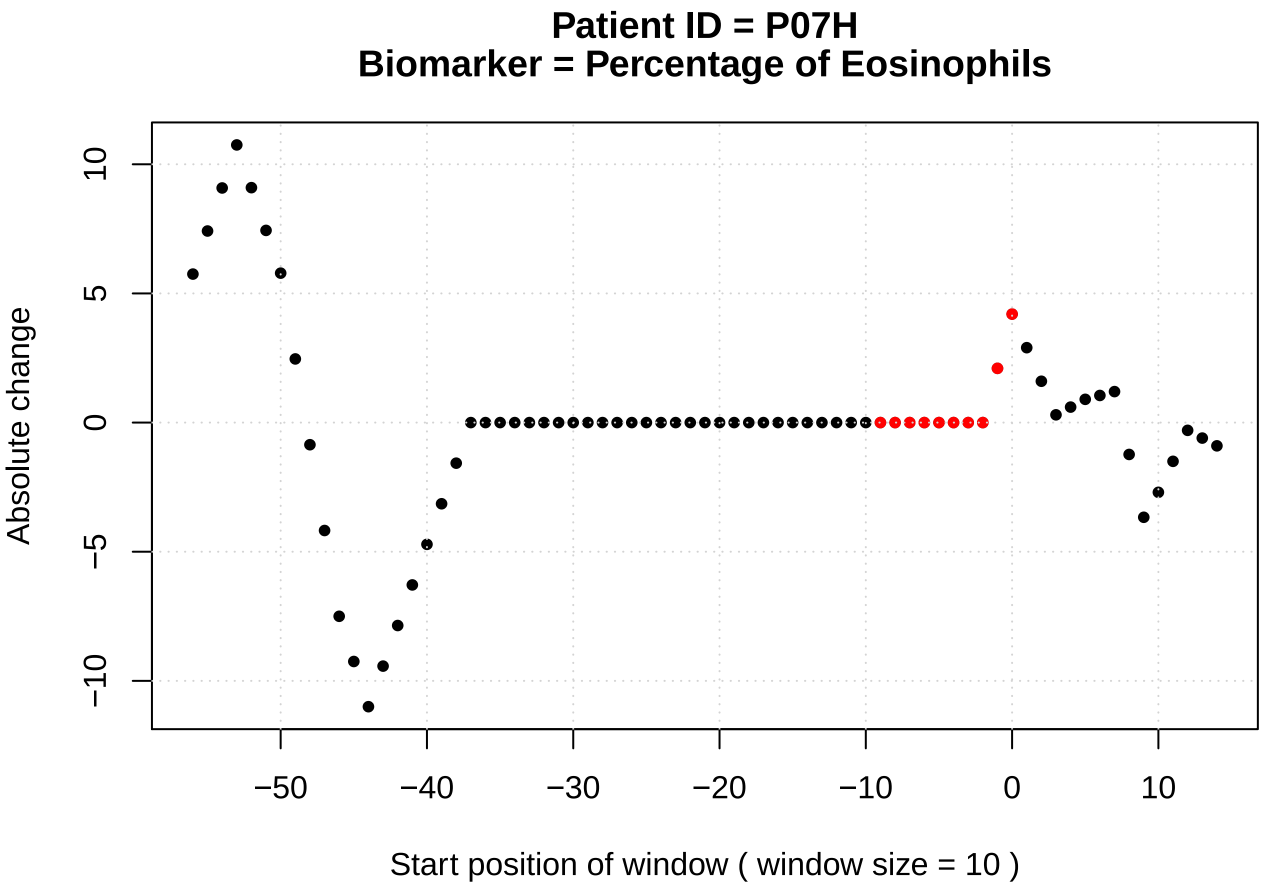
Patient ID = P07H
Biomarker = Percentage of Eosinophils
Absolute change
Start position of window ( window size = 10 )
−50
−40
−30
−20
−10
0
10
−10
−5
0
5
10

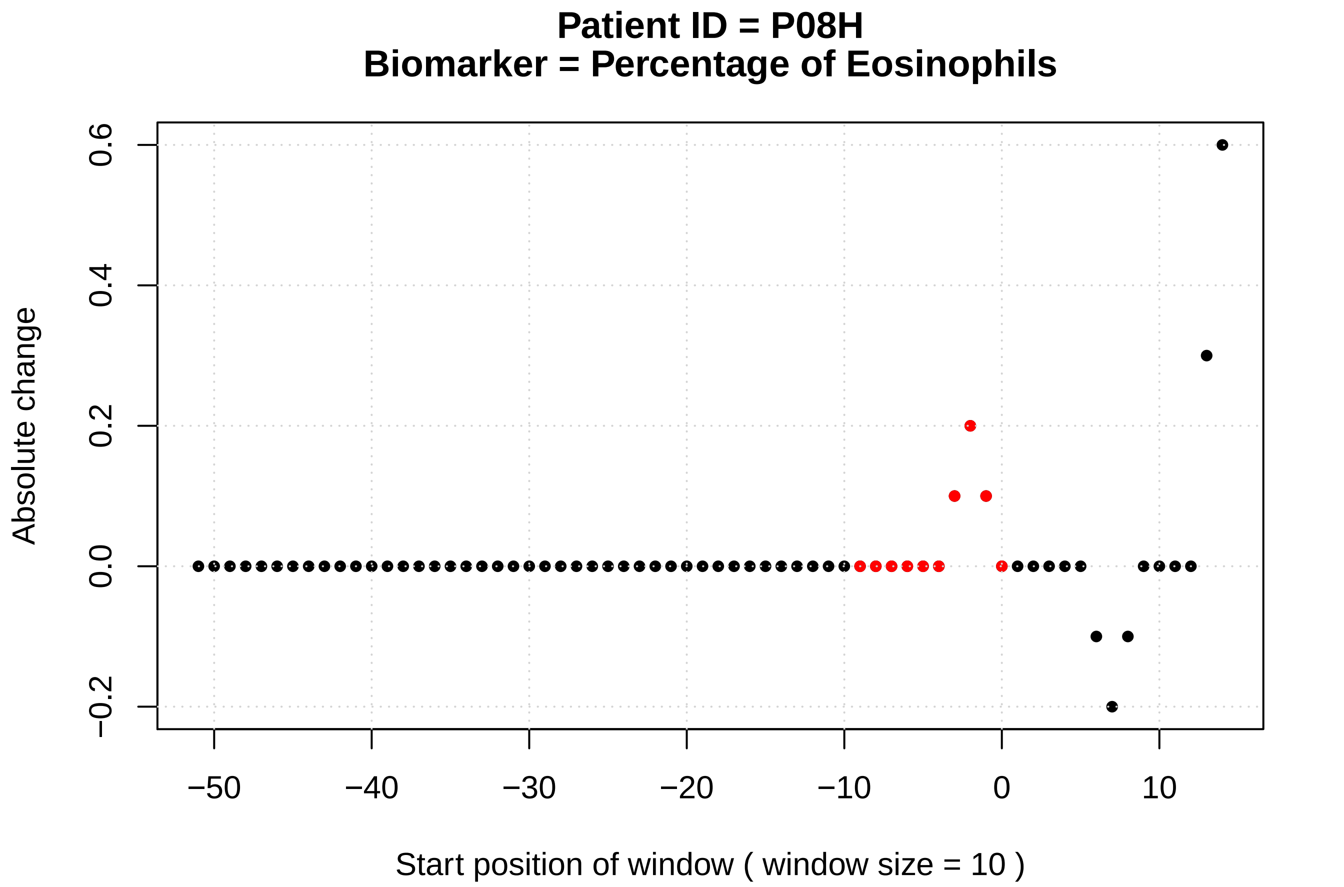

Patient ID = P08H
Biomarker = Percentage of Eosinophils
Absolute change
−0.2
0.0
0.2
0.4
0.6
−50
−40
−30
−20
−10
0
10
Start position of window ( window size = 10 )

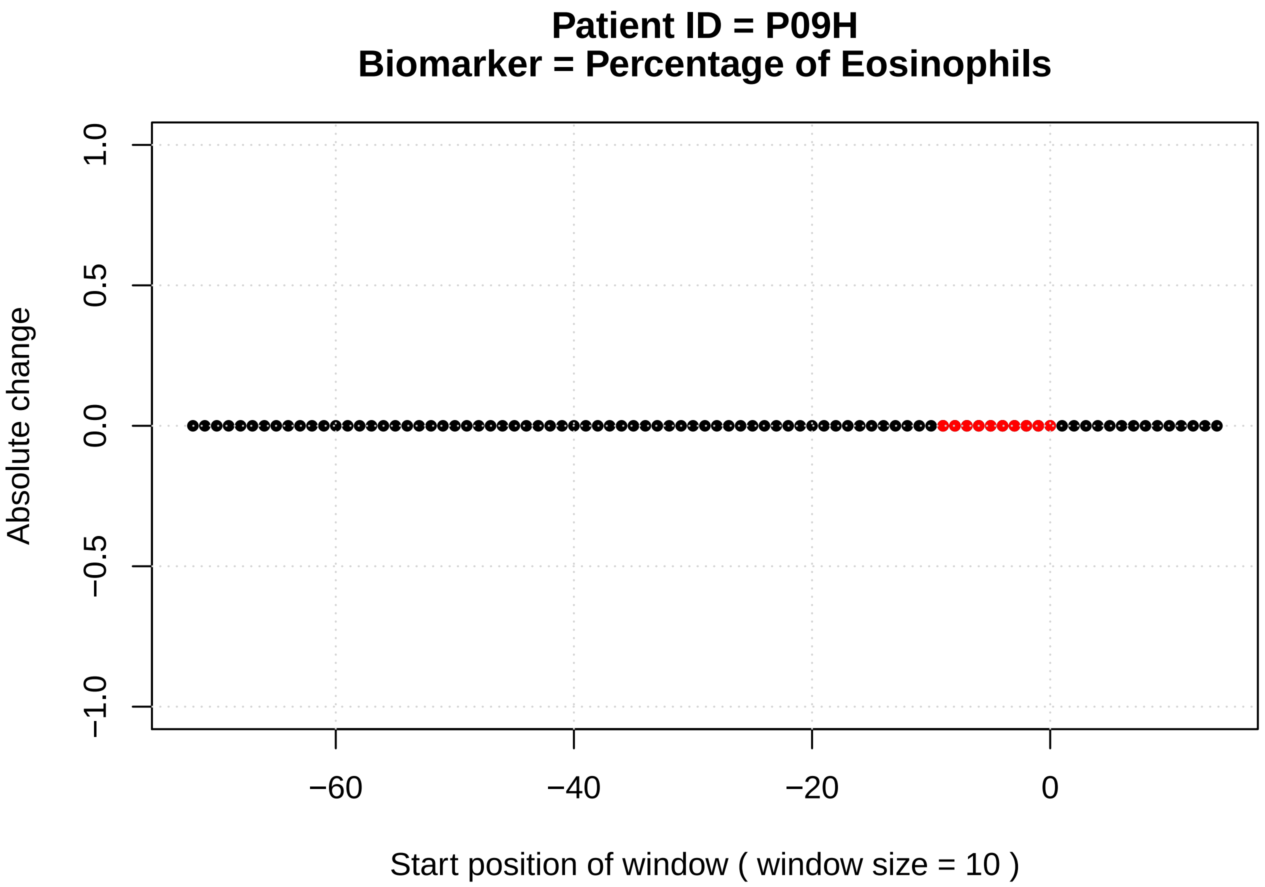

Patient ID = P09H
Biomarker = Percentage of Eosinophils
Absolute change
1.0
0.5
0.0
−0.5
−1.0
−60
−40
−20
0
Start position of window ( window size = 10 )

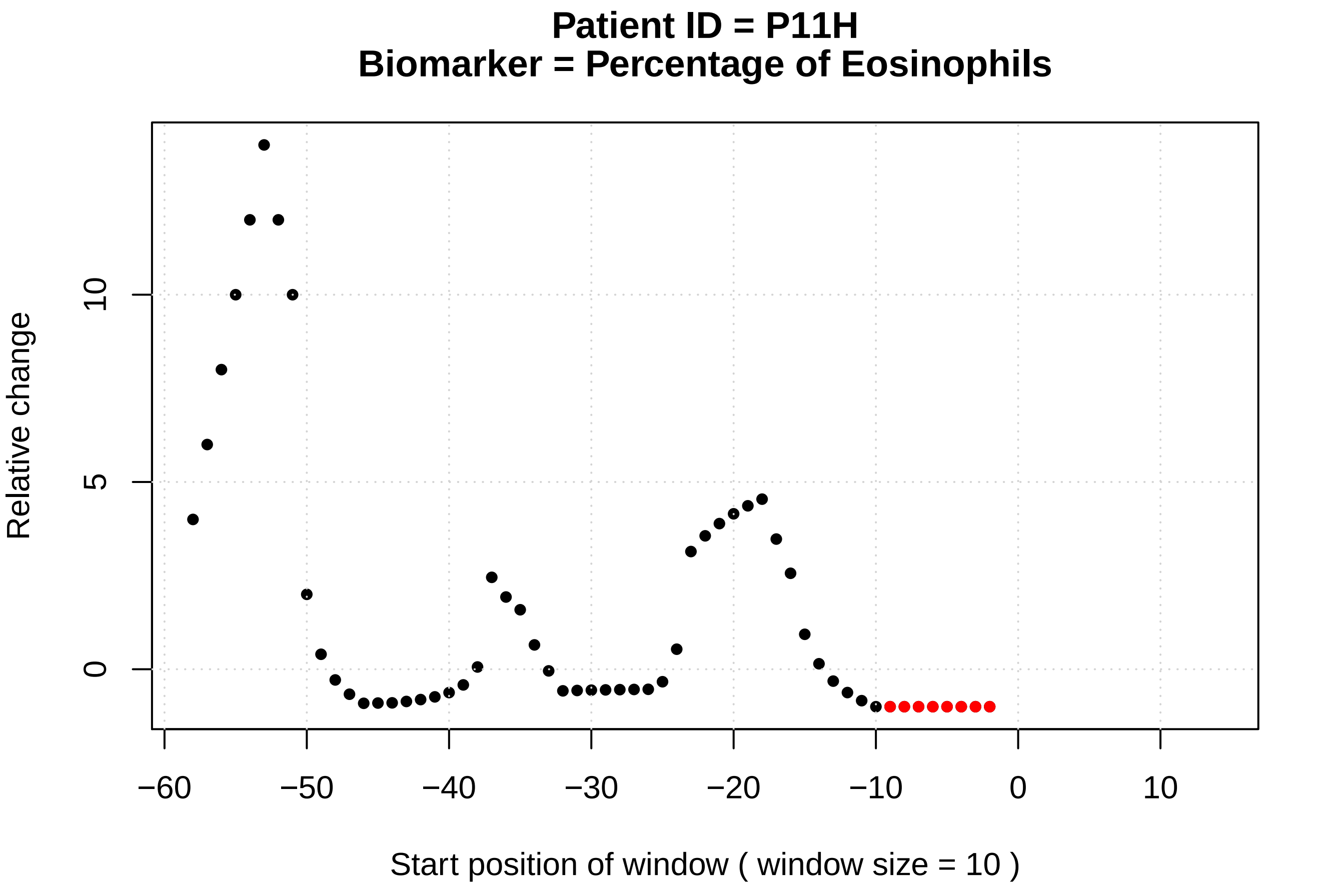
Patient ID = P11H
Biomarker = Percentage of Eosinophils
Relative change
0
5
10
−60
−50
−40
−30
−20
−10
0
10
Start position of window ( window size = 10 )

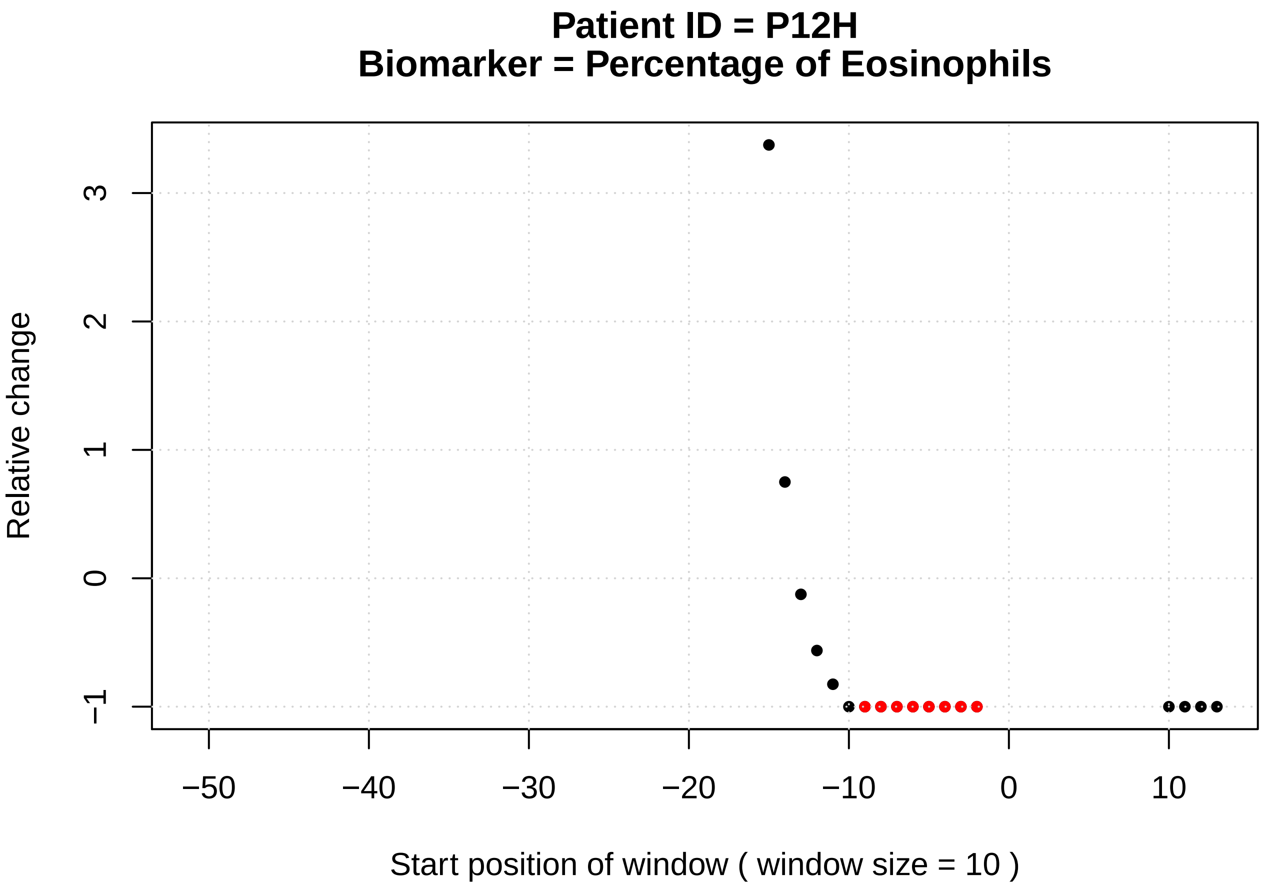
Patient ID = P12H
Biomarker = Percentage of Eosinophils
Relative change
3
2
1
0
−1
−50
−40
−30
−20
−10
0
10
Start position of window ( window size = 10 )

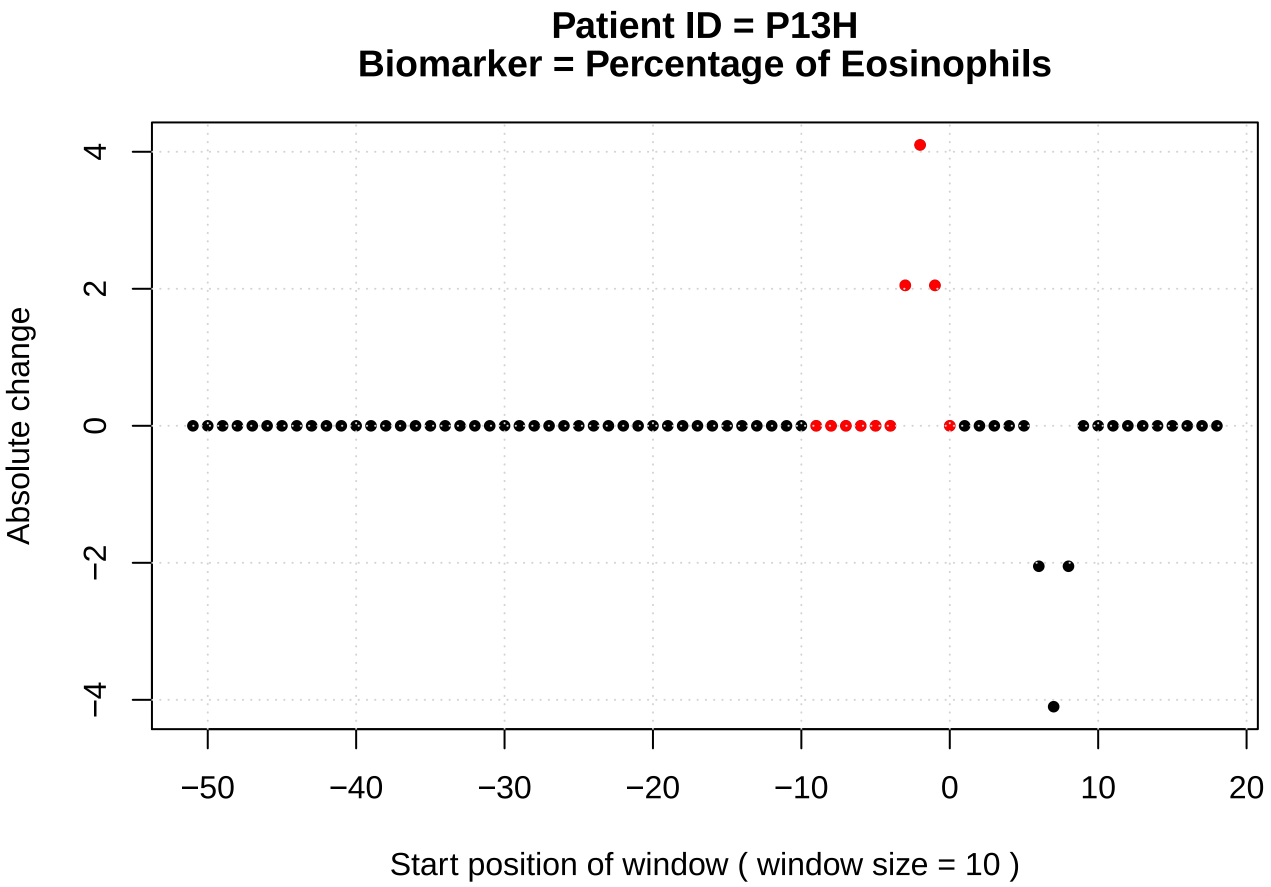
Patient ID = P13H
Biomarker = Percentage of Eosinophils
Absolute change
4
2
0
−2
−4
−50
−40
−30
−20
−10
0
10
20
Start position of window ( window size = 10 )

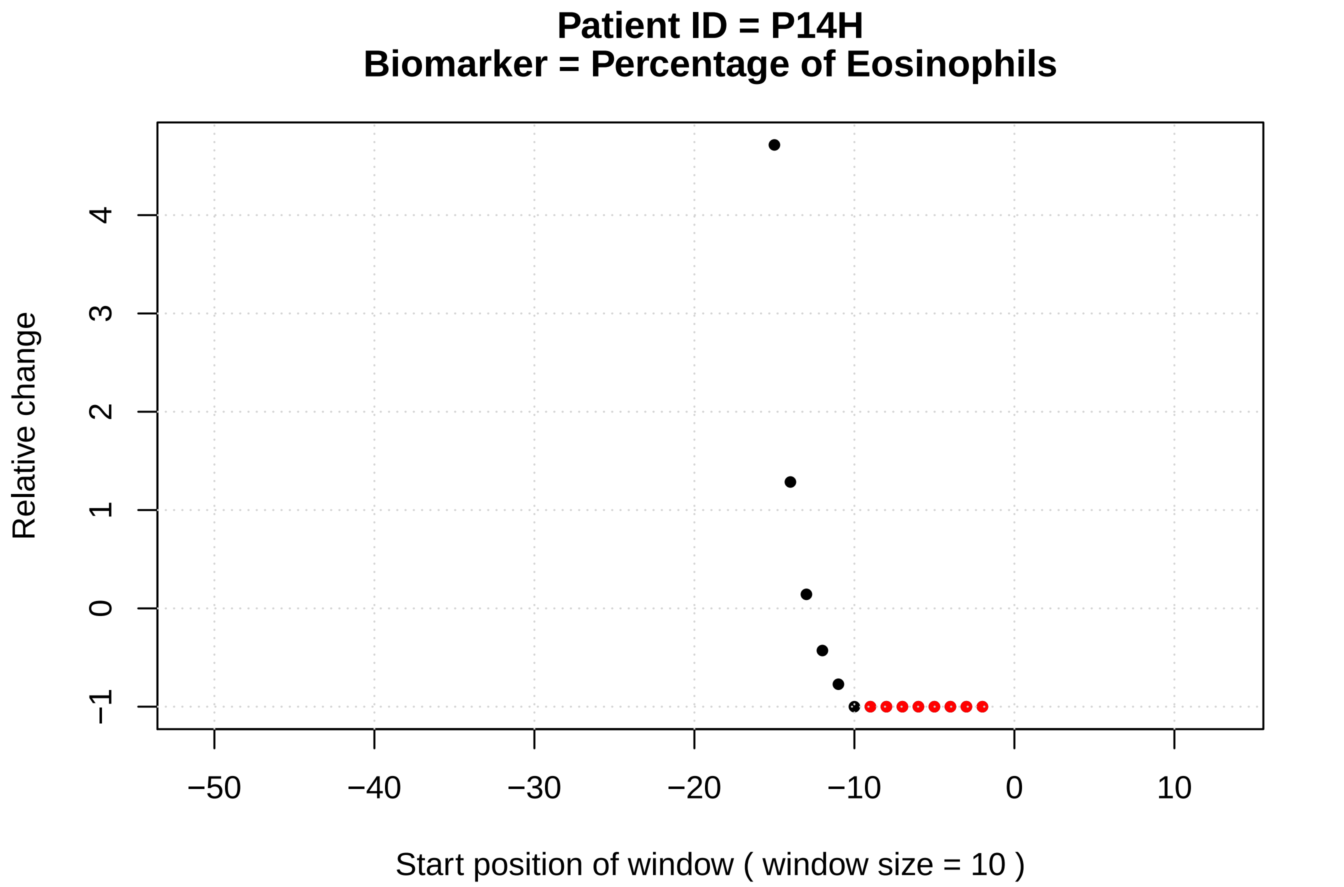

Patient ID = P14H
Biomarker = Percentage of Eosinophils
Relative change
4
3
2
1
0
−1
−50
−40
−30
−20
−10
0
10
Start position of window ( window size = 10 )

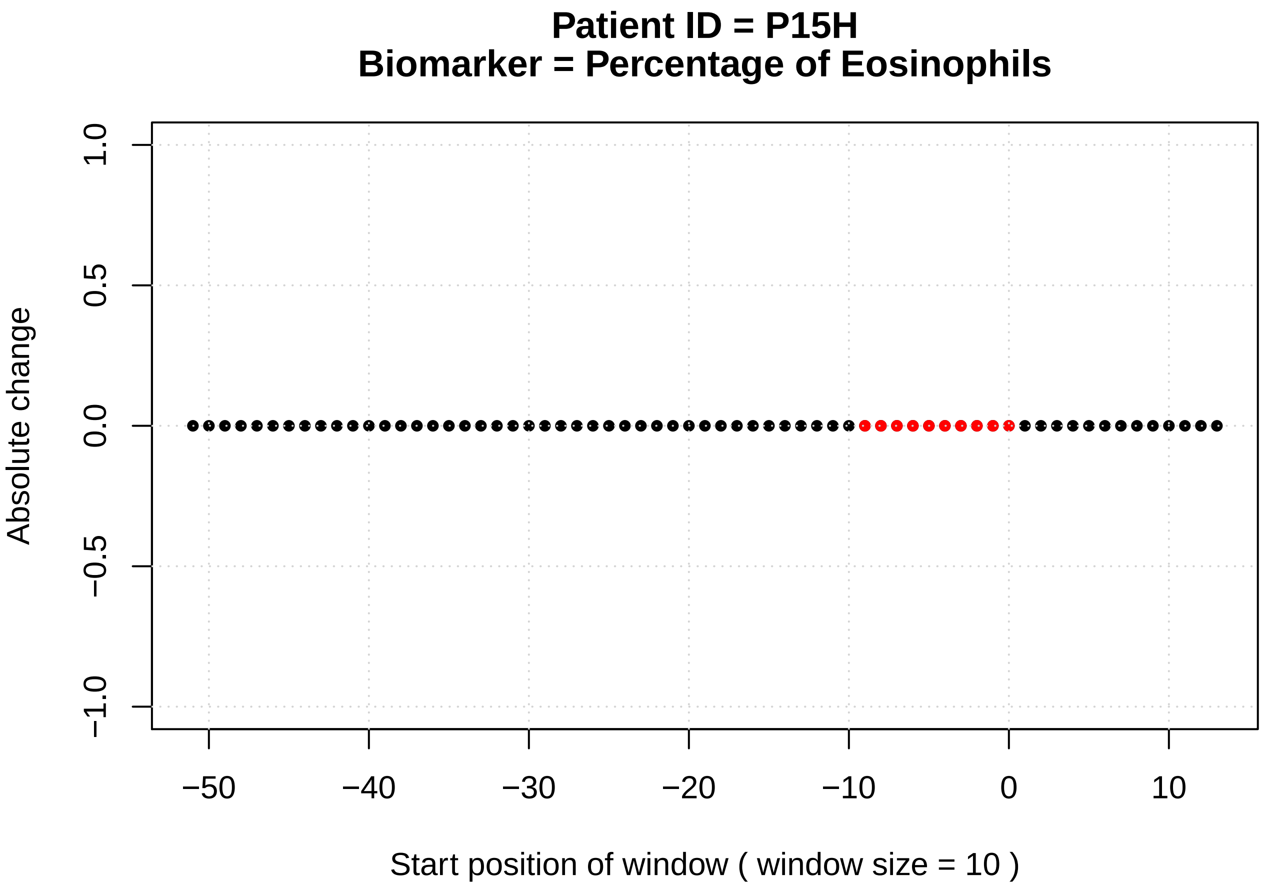
Patient ID = P15H
Biomarker = Percentage of Eosinophils
Absolute change
1.0
0.5
0.0
−0.5
−1.0
−50
−40
−30
−20
−10
0
10
Start position of window ( window size = 10 )
