# Supplementary material for: Loss of adaptive capacity in asthmatic patients revealed by biomarker fluctuation dynamics after rhinovirus challenge
Source: eLife. 2019 Nov 5;8:e47969. doi: 10.7554/eLife.47969 (PMC6877087; doi:10.7554/eLife.47969)
Supplement: Supplementary file 1. [file elife-47969-supp1.zip › RelativeChangeWithinWindowPlots_PDFs/Appendix-figure SS20 IndividualWindowBoundariesRelativeChangeAnalysis NFEV1 Window Size = 10 AsthmaticParticipants.pdf]

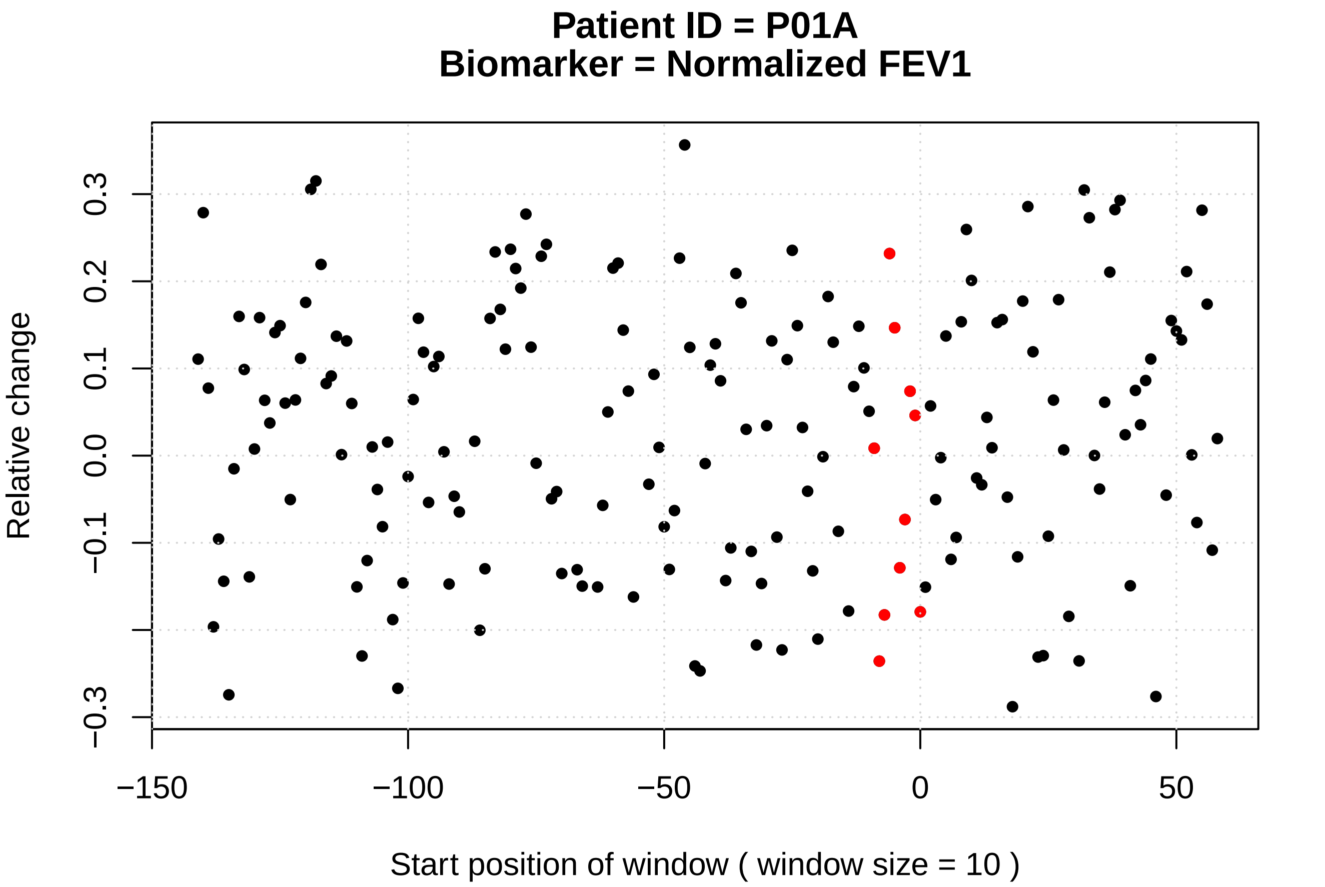
Patient ID = P01A
Biomarker = Normalized FEV1
Relative change
Start position of window (window size = 10)
−150
−100
−50
0
50
0.3
0.2
0.1
0.0
−0.1
−0.3

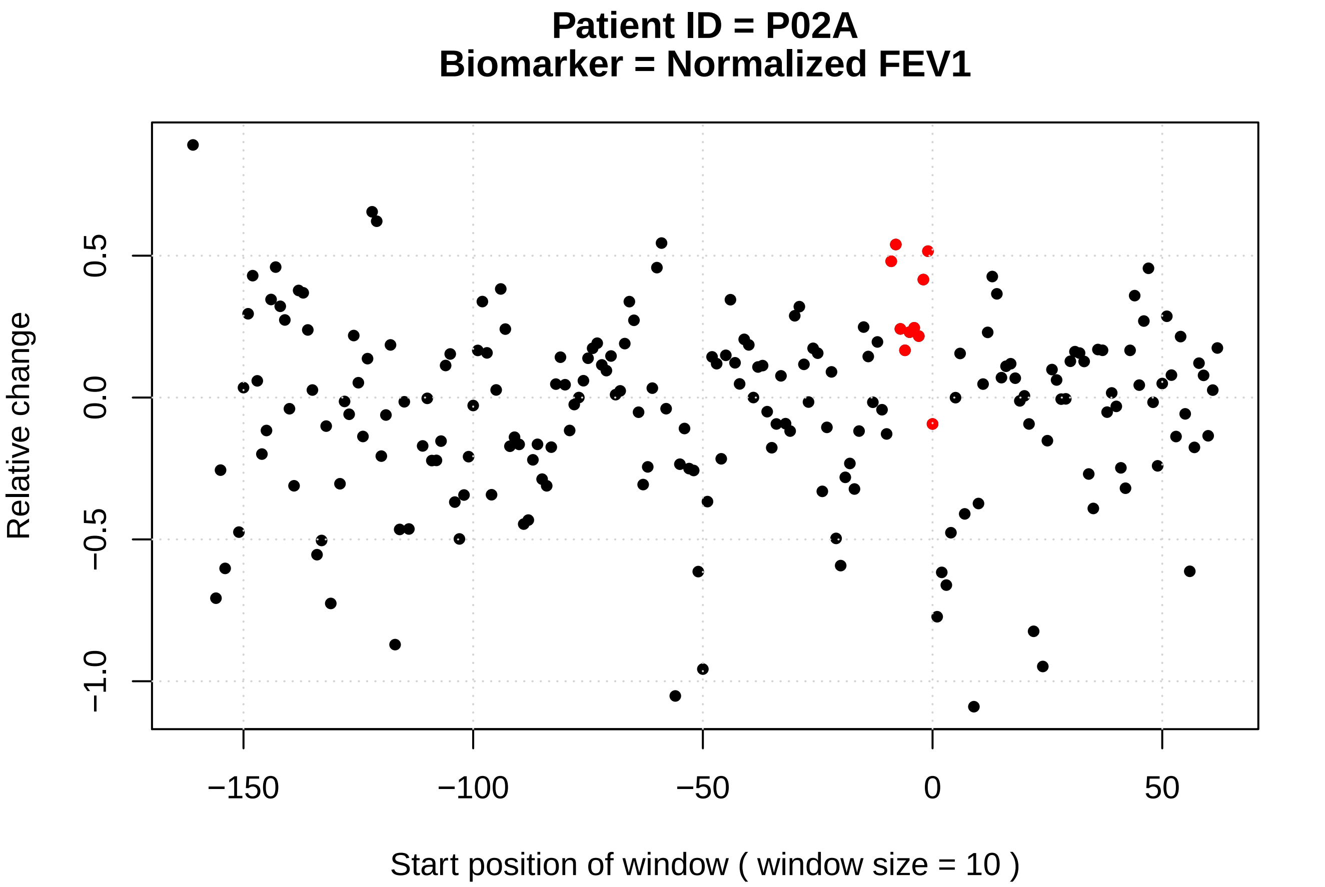

Patient ID = P02A
Biomarker = Normalized FEV1
Relative change
0.5
0.0
−0.5
−1.0
−150
−100
−50
0
50
Start position of window (window size = 10)

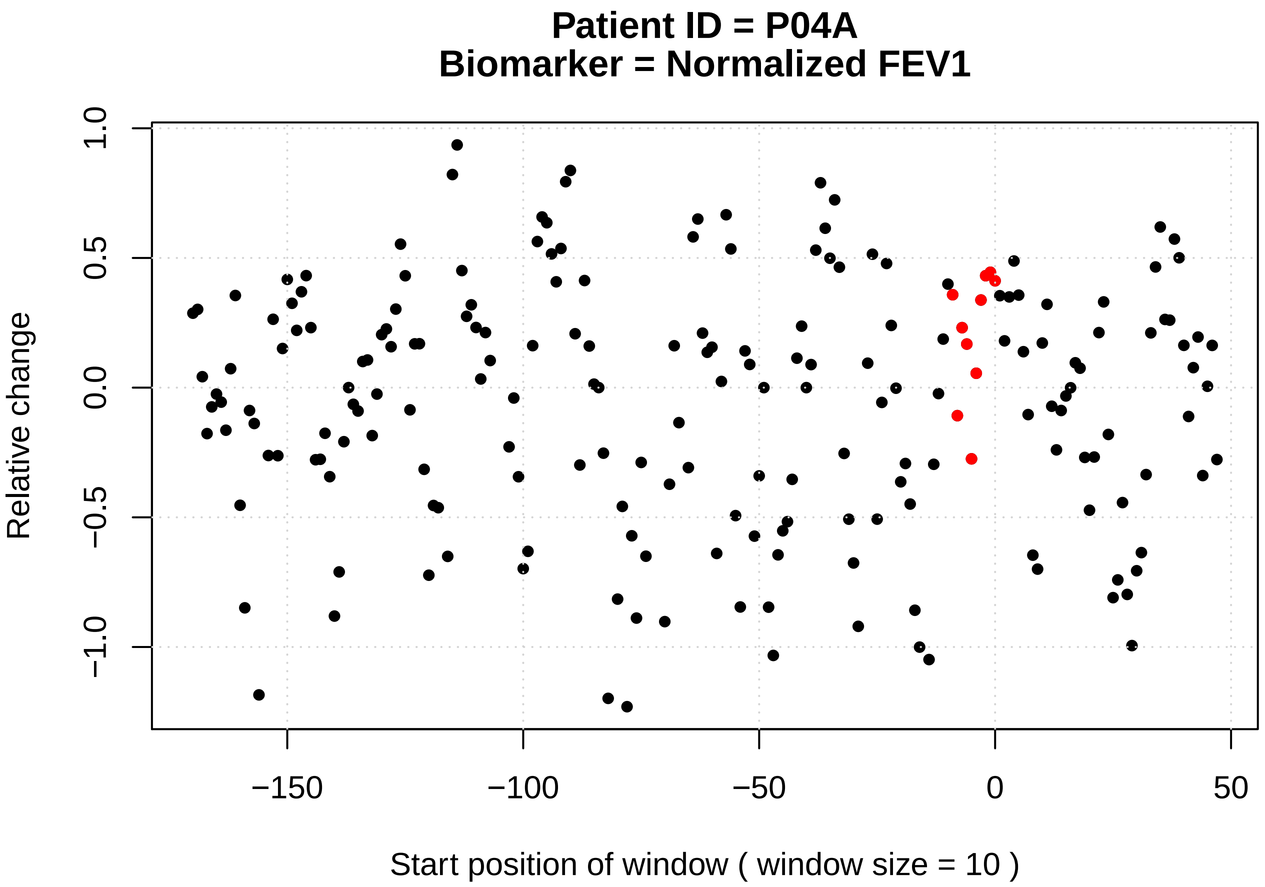

Patient ID = P04A
Biomarker = Normalized FEV1
Relative change
1.0
0.5
0.0
−0.5
−1.0
−150
−100
−50
0
50
Start position of window (window size = 10)

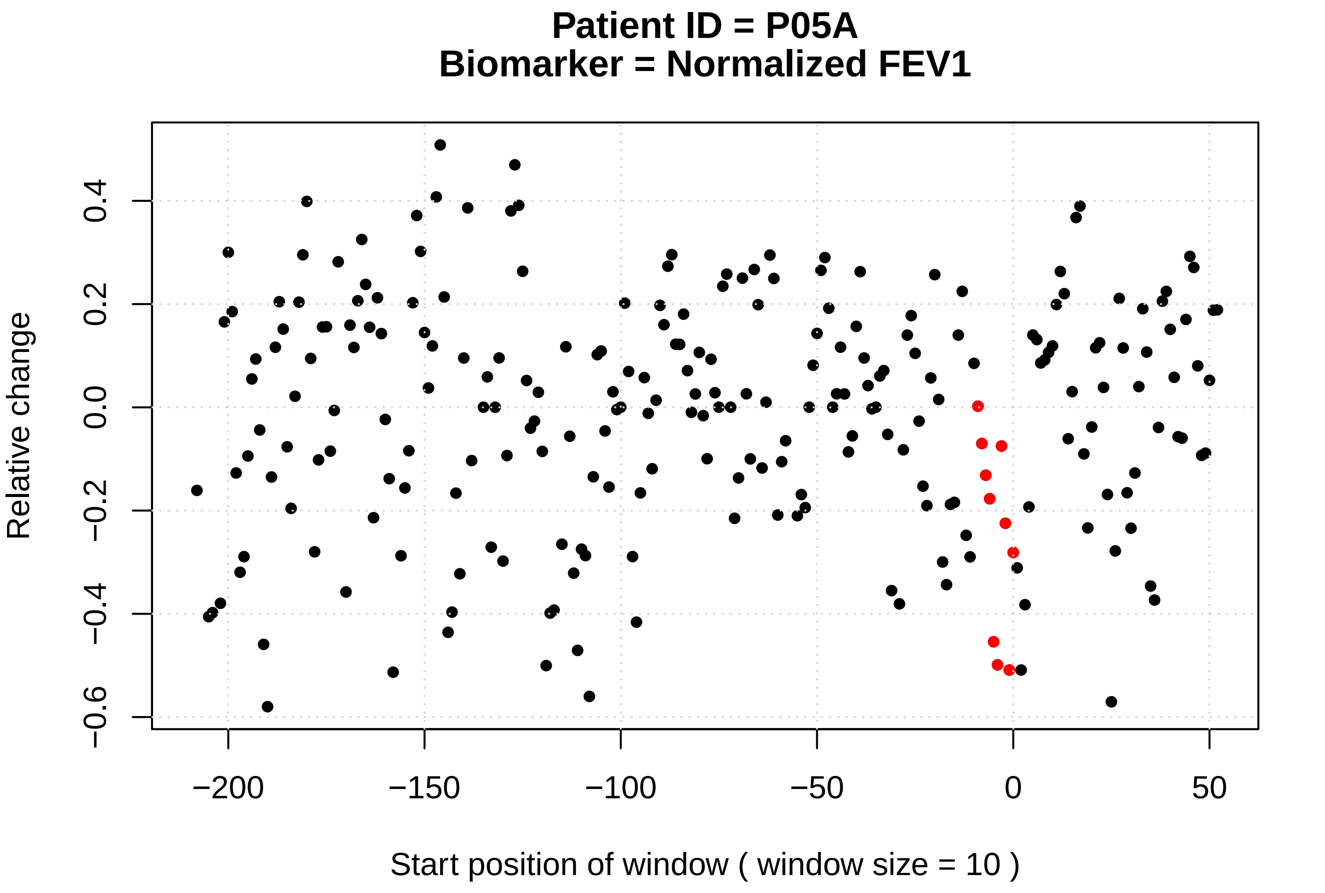

Patient ID = P05A
Biomarker = Normalized FEV1
Relative change
0.4
0.2
0.0
−0.2
−0.4
−0.6
−200
−150
−100
−50
0
50
Start position of window (window size = 10)

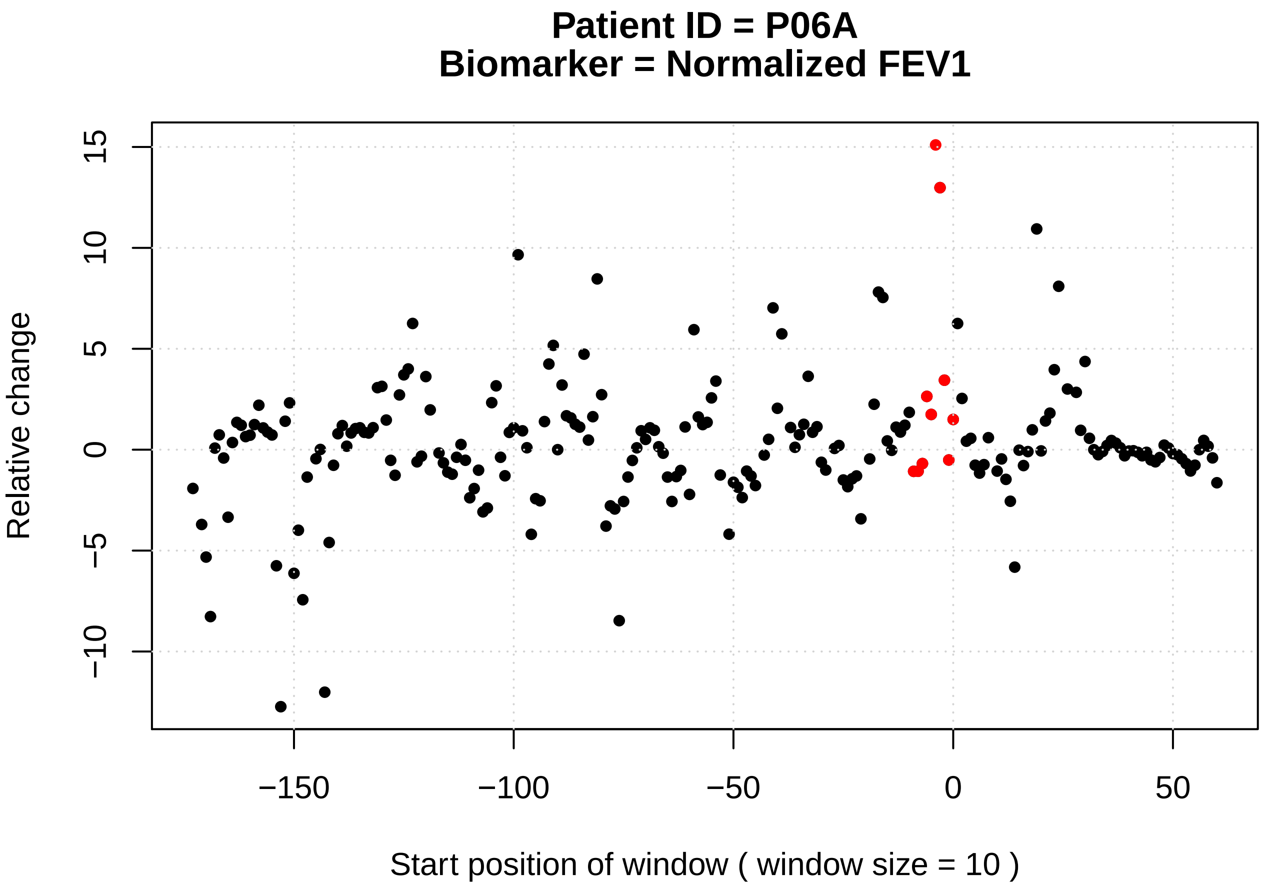

Patient ID = P06A
Biomarker = Normalized FEV1
Relative change
15
10
5
0
−5
−10
−150
−100
−50
0
50
Start position of window (window size = 10)

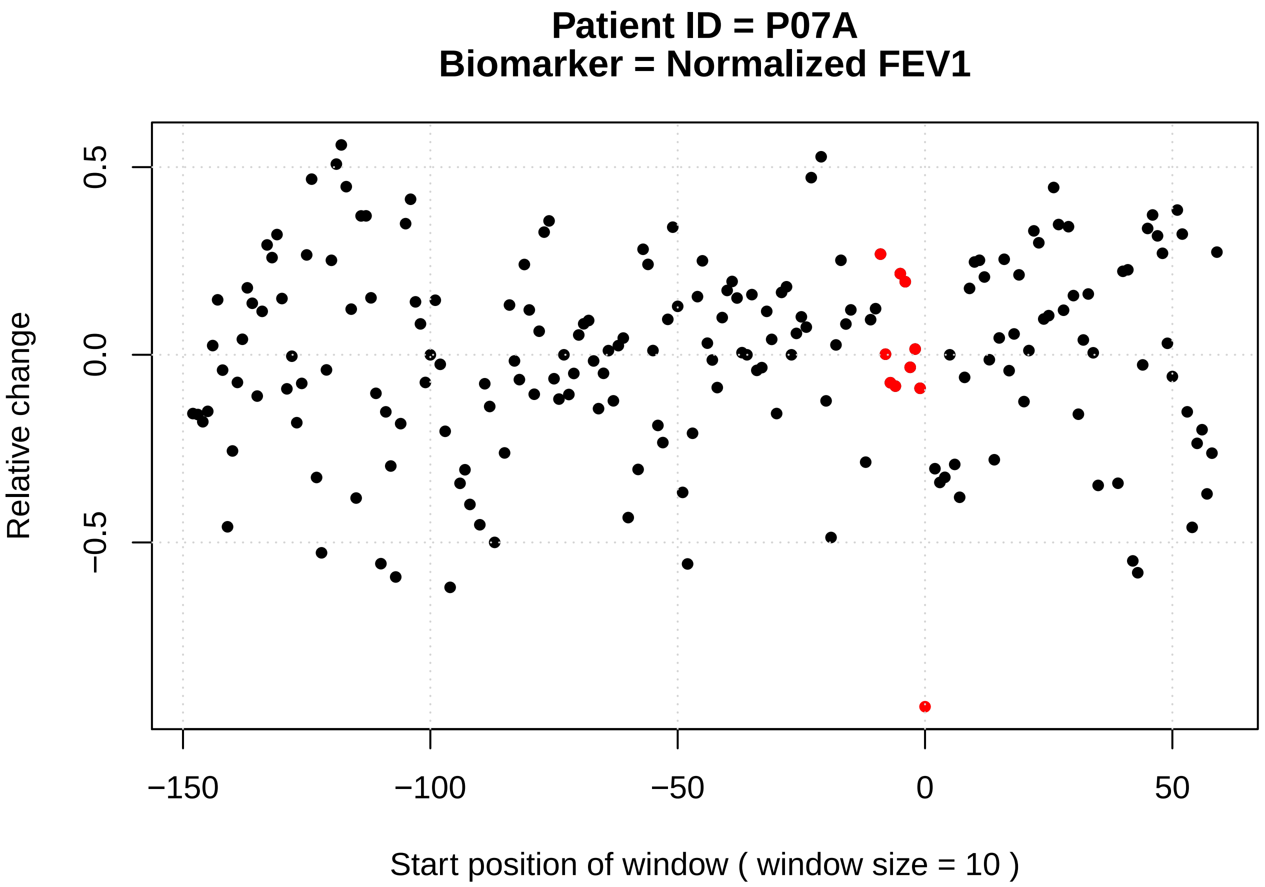

Patient ID = P07A
Biomarker = Normalized FEV1
Relative change
0.5
0.0
−0.5
−150
−100
−50
0
50
Start position of window (window size = 10)

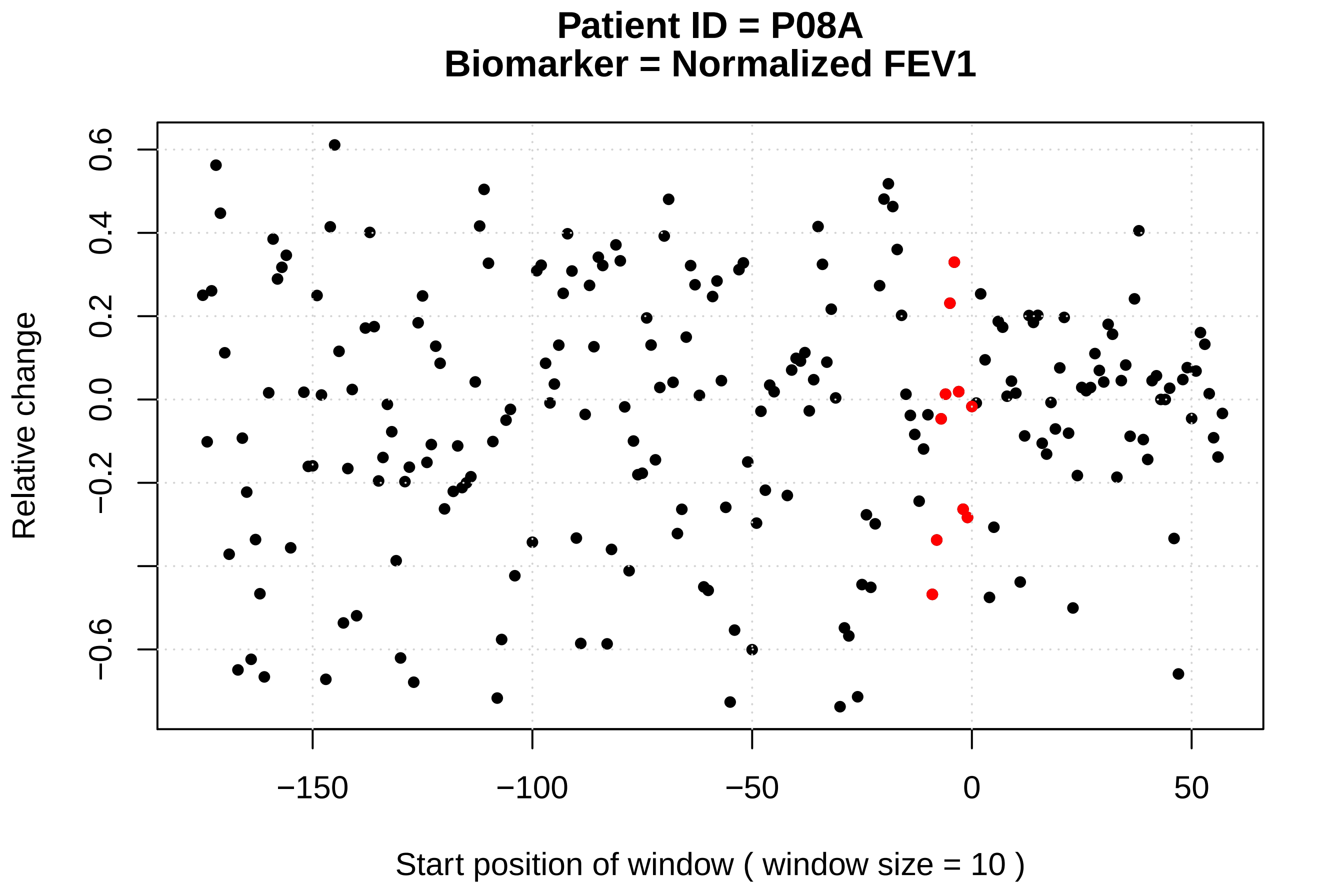

Patient ID = P08A
Biomarker = Normalized FEV1
Relative change
0.6
0.4
0.2
0.0
−0.2
−0.6
−150
−100
−50
0
50
Start position of window (window size = 10)

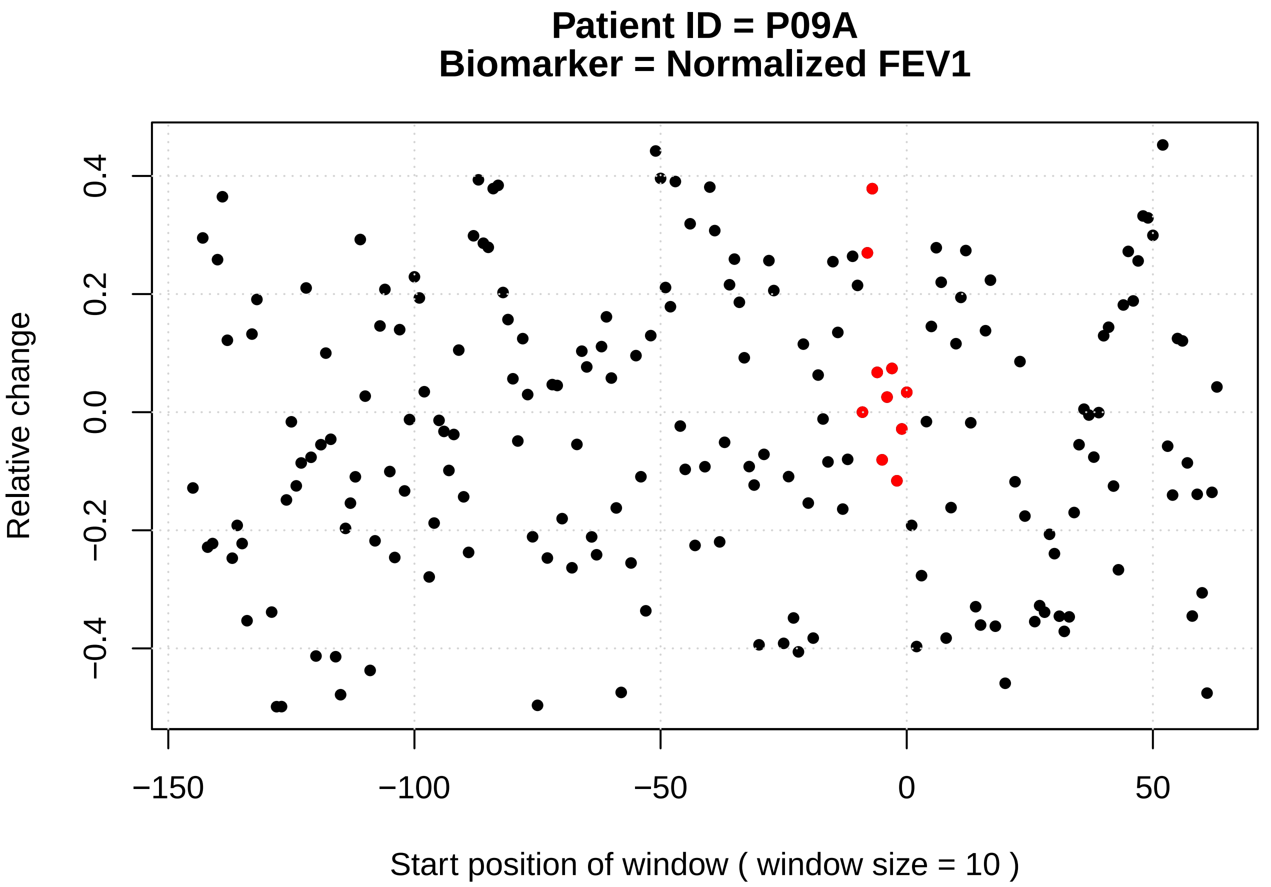

Patient ID = P09A
Biomarker = Normalized FEV1
Relative change
0.4
0.2
0.0
−0.2
−0.4
−150
−100
−50
0
50
Start position of window (window size = 10)

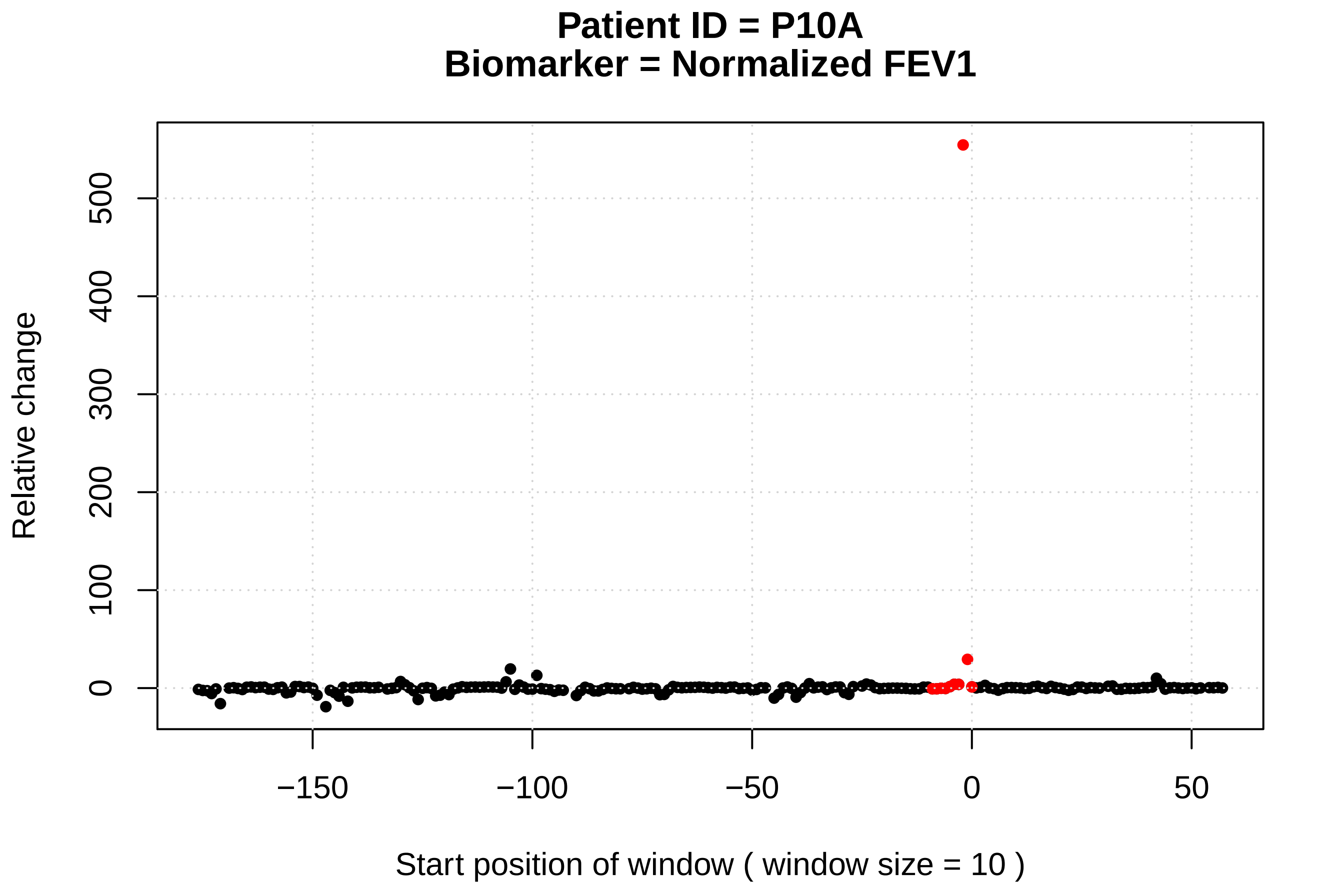

Patient ID = P10A
Biomarker = Normalized FEV1
Relative change
0
100
200
300
400
500
−150
−100
−50
0
50
Start position of window (window size = 10)

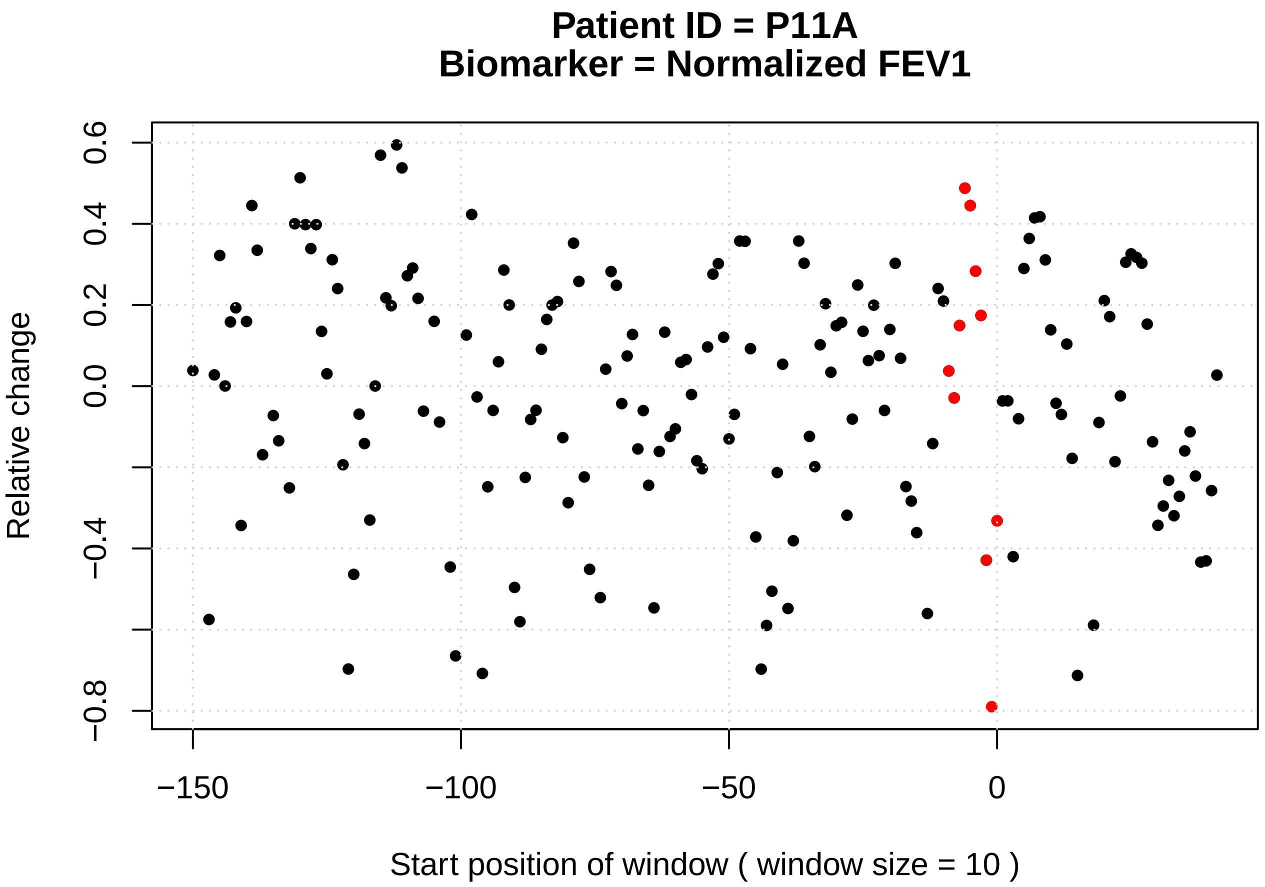

Patient ID = P11A
Biomarker = Normalized FEV1
Relative change
Start position of window (window size = 10)
−150
−100
−50
0
0.6
0.4
0.2
0.0
−0.4
−0.8

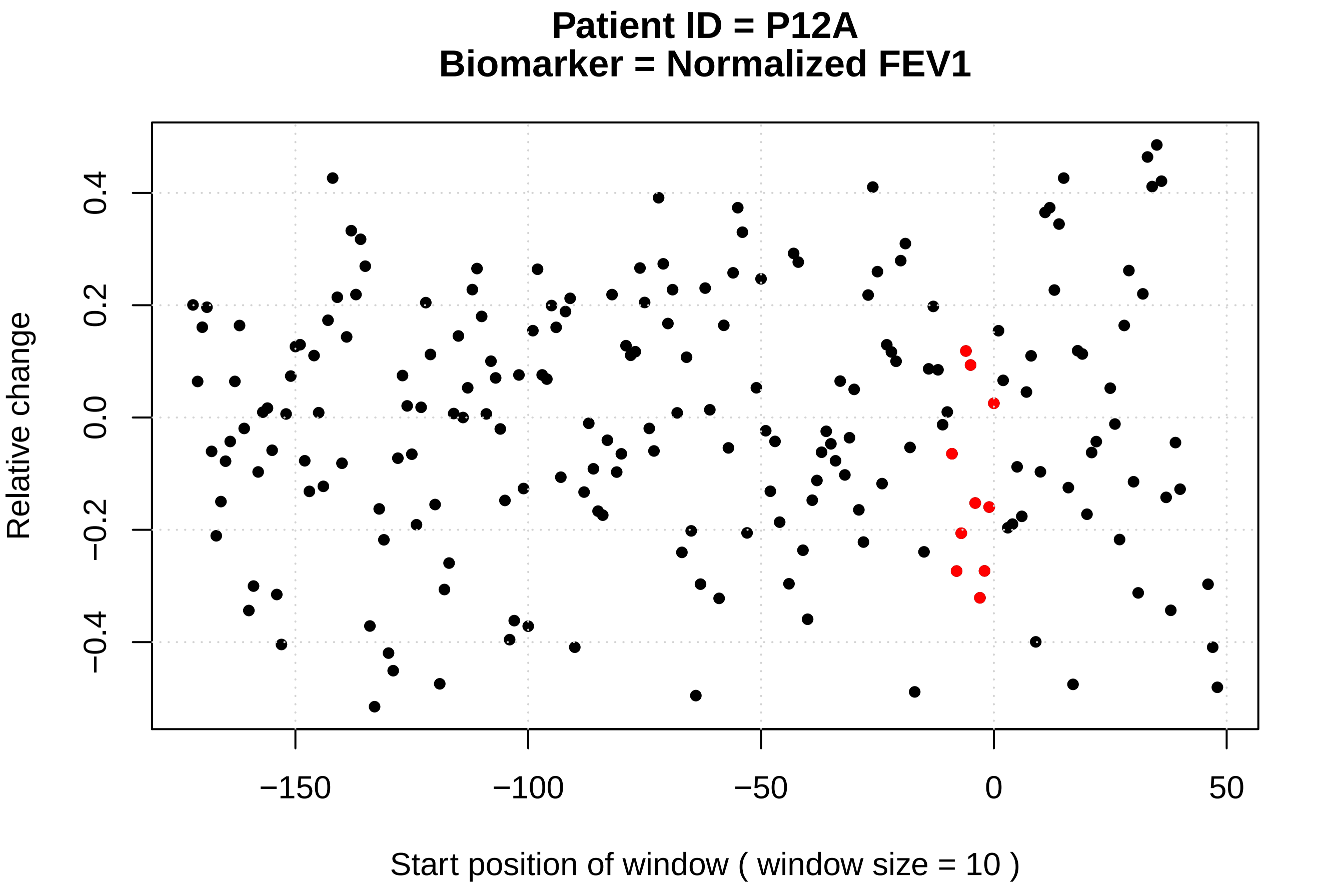

Patient ID = P12A
Biomarker = Normalized FEV1
Relative change
0.4
0.2
0.0
−0.2
−0.4
−150
−100
−50
0
50
Start position of window (window size = 10)

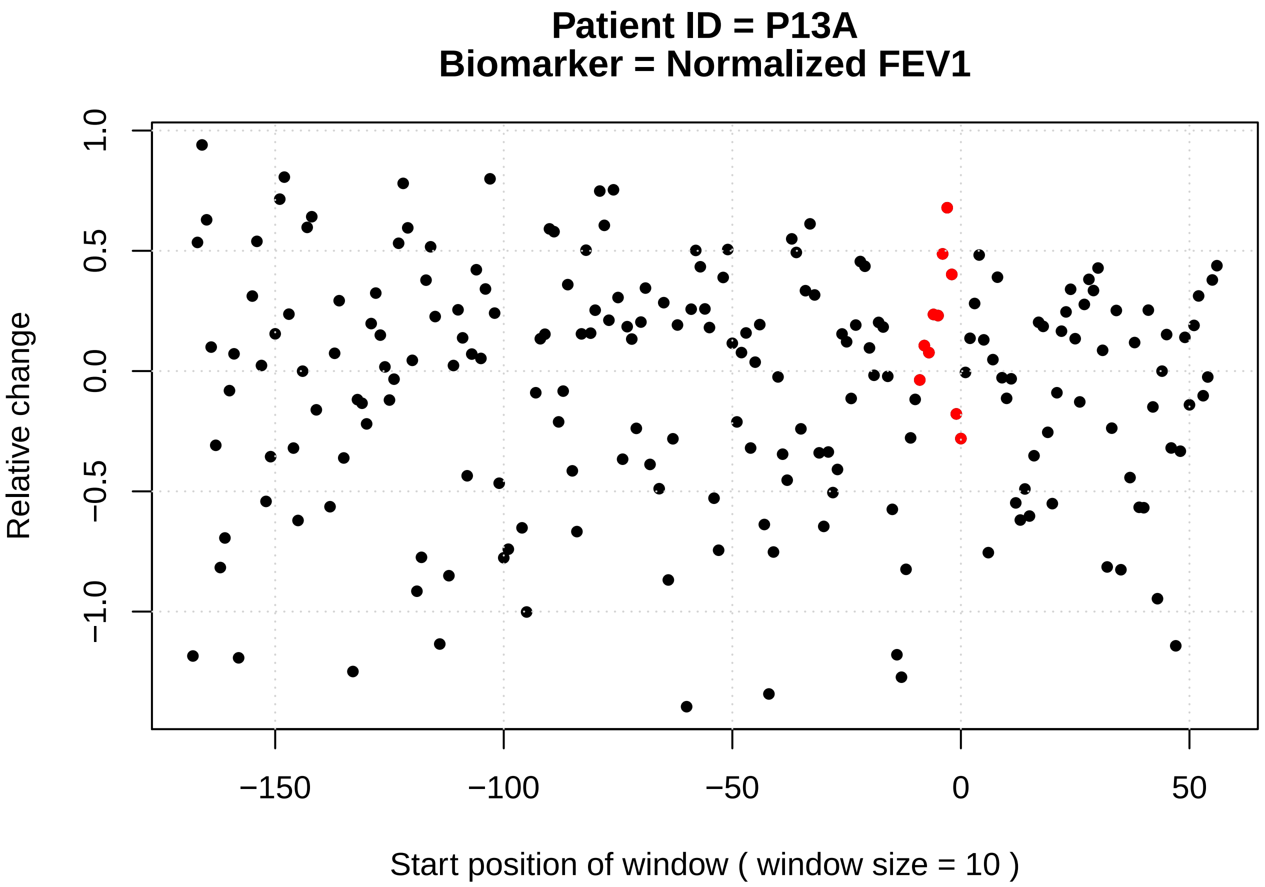

Patient ID = P13A
Biomarker = Normalized FEV1
Relative change
Start position of window (window size = 10)
1.0
0.5
0.0
−0.5
−1.0
−150
−100
−50
0
50
