# Supplementary material for: Loss of adaptive capacity in asthmatic patients revealed by biomarker fluctuation dynamics after rhinovirus challenge
Source: eLife. 2019 Nov 5;8:e47969. doi: 10.7554/eLife.47969 (PMC6877087; doi:10.7554/eLife.47969)

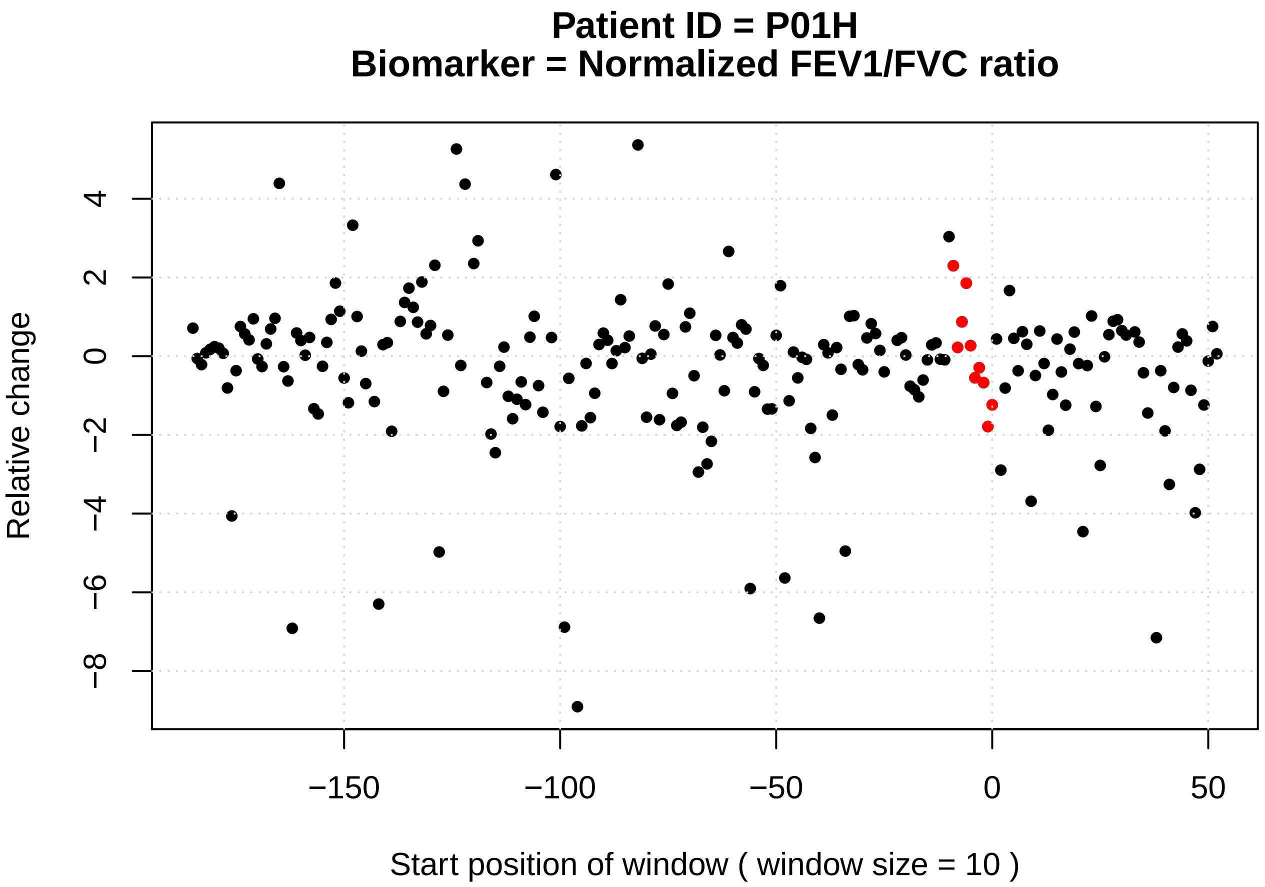
Patient ID = P01H
Biomarker = Normalized FEV1/FVC ratio
Relative change
4
2
0
−2
−4
−6
−8
−150
−100
−50
0
50
Start position of window (window size = 10)

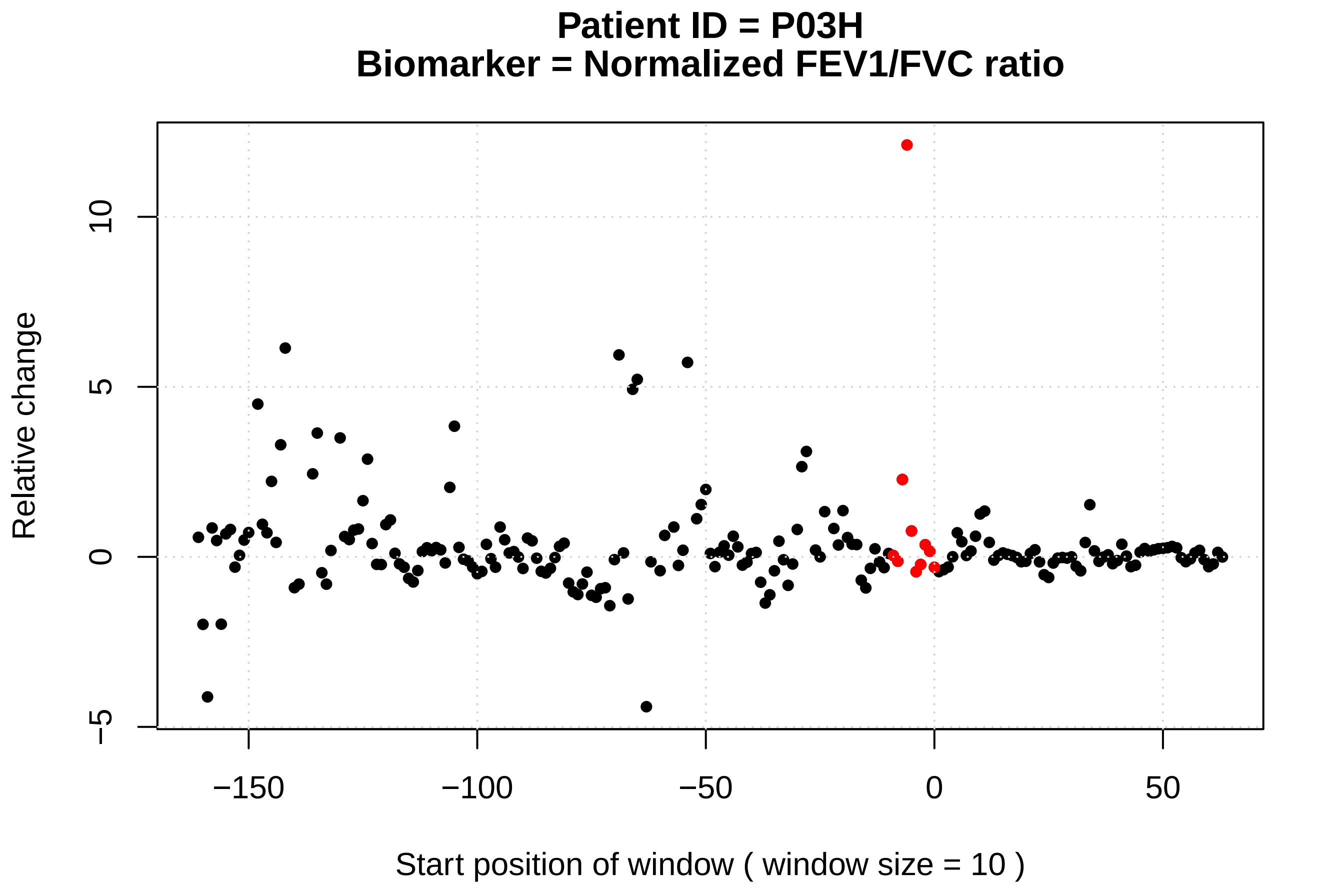
Patient ID = P03H
Biomarker = Normalized FEV1/FVC ratio
Relative change
−5
0
5
10
−150
−100
−50
0
50
Start position of window (window size = 10)

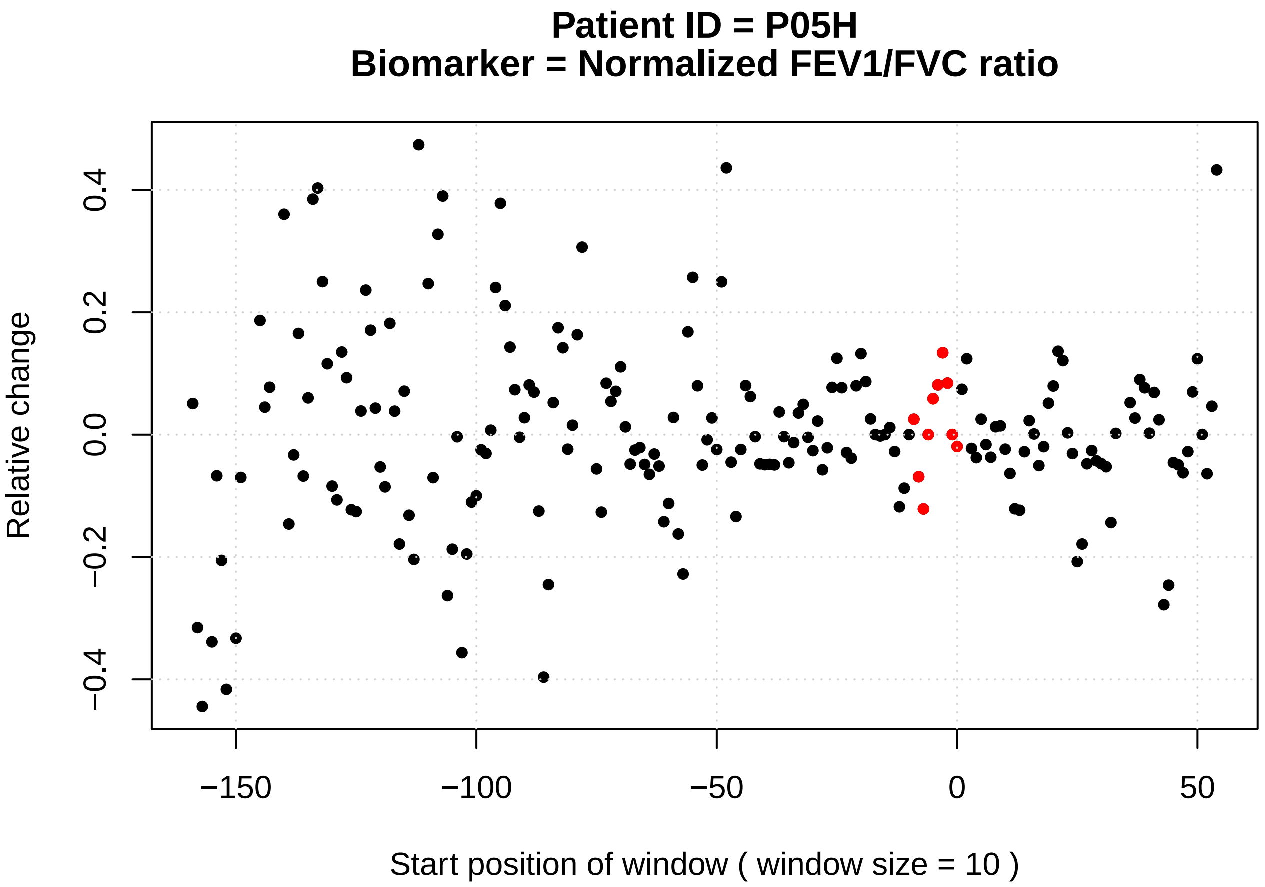
Patient ID = P05H
Biomarker = Normalized FEV1/FVC ratio
Relative change
0.4
0.2
0.0
−0.2
−0.4
−150
−100
−50
0
50
Start position of window (window size = 10)

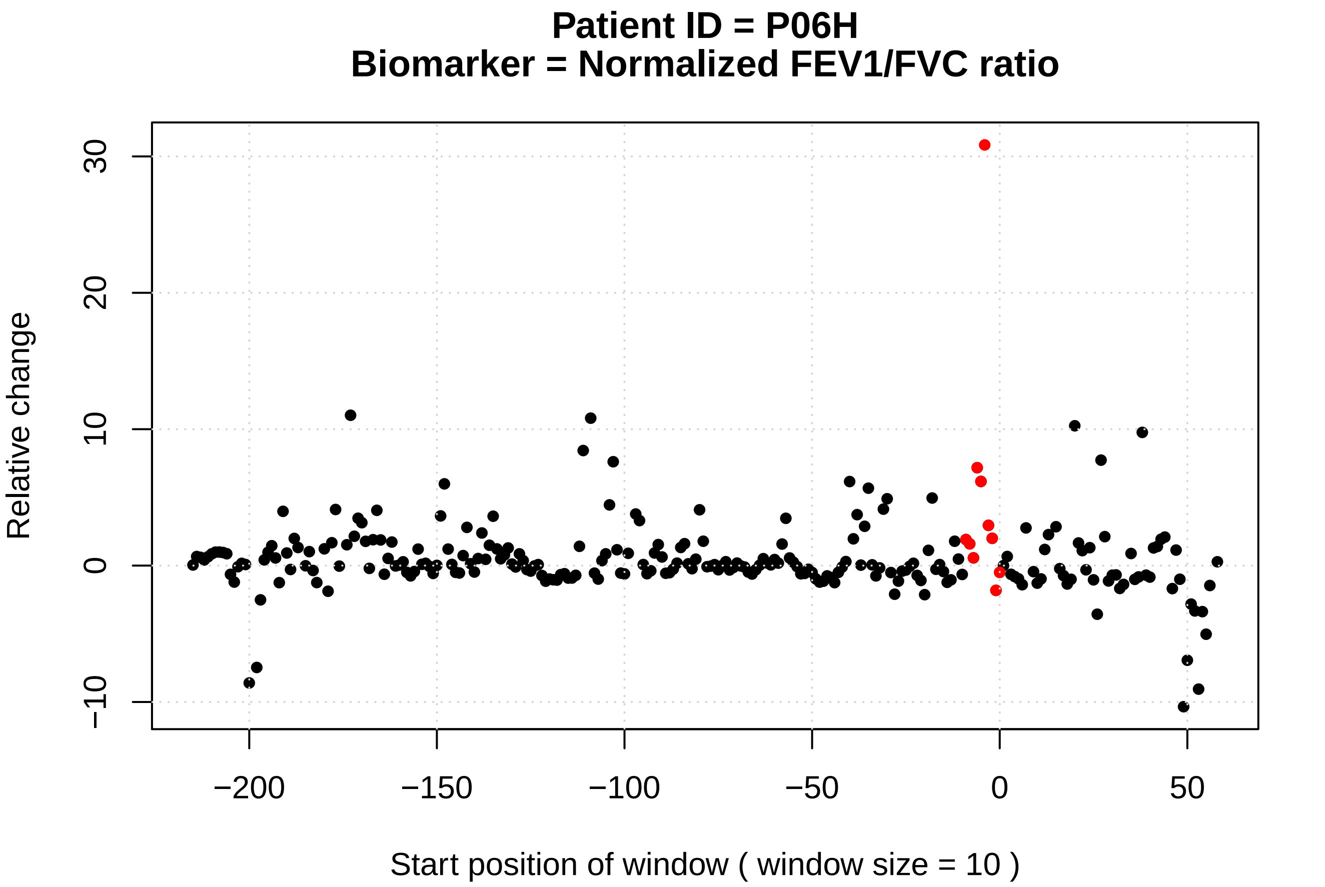
Patient ID = P06H
Biomarker = Normalized FEV1/FVC ratio
Relative change
30
20
10
0
−10
−200
−150
−100
−50
0
50
Start position of window (window size = 10)

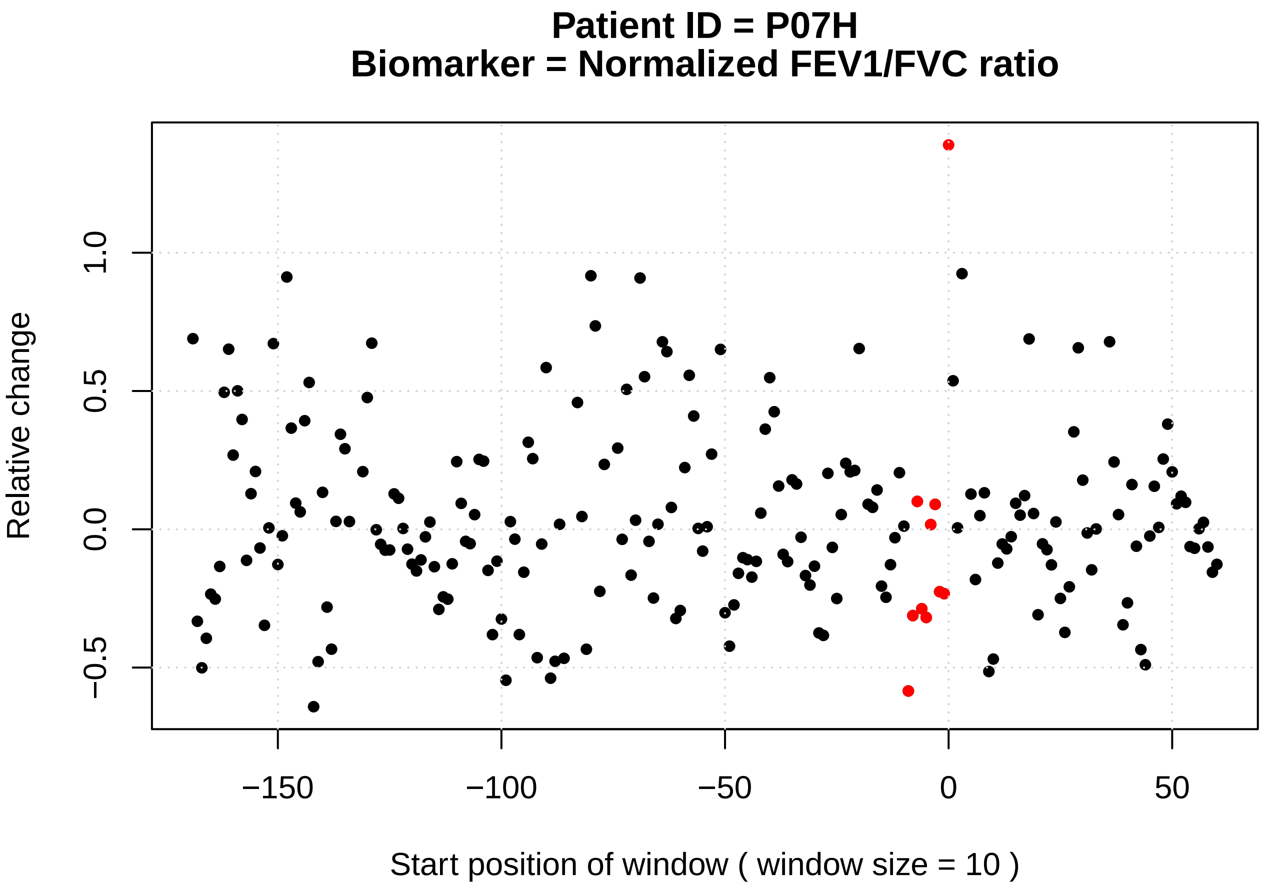
Patient ID = P07H
Biomarker = Normalized FEV1/FVC ratio
Relative change
−0.5
0.0
0.5
1.0
−150
−100
−50
0
50
Start position of window (window size = 10)

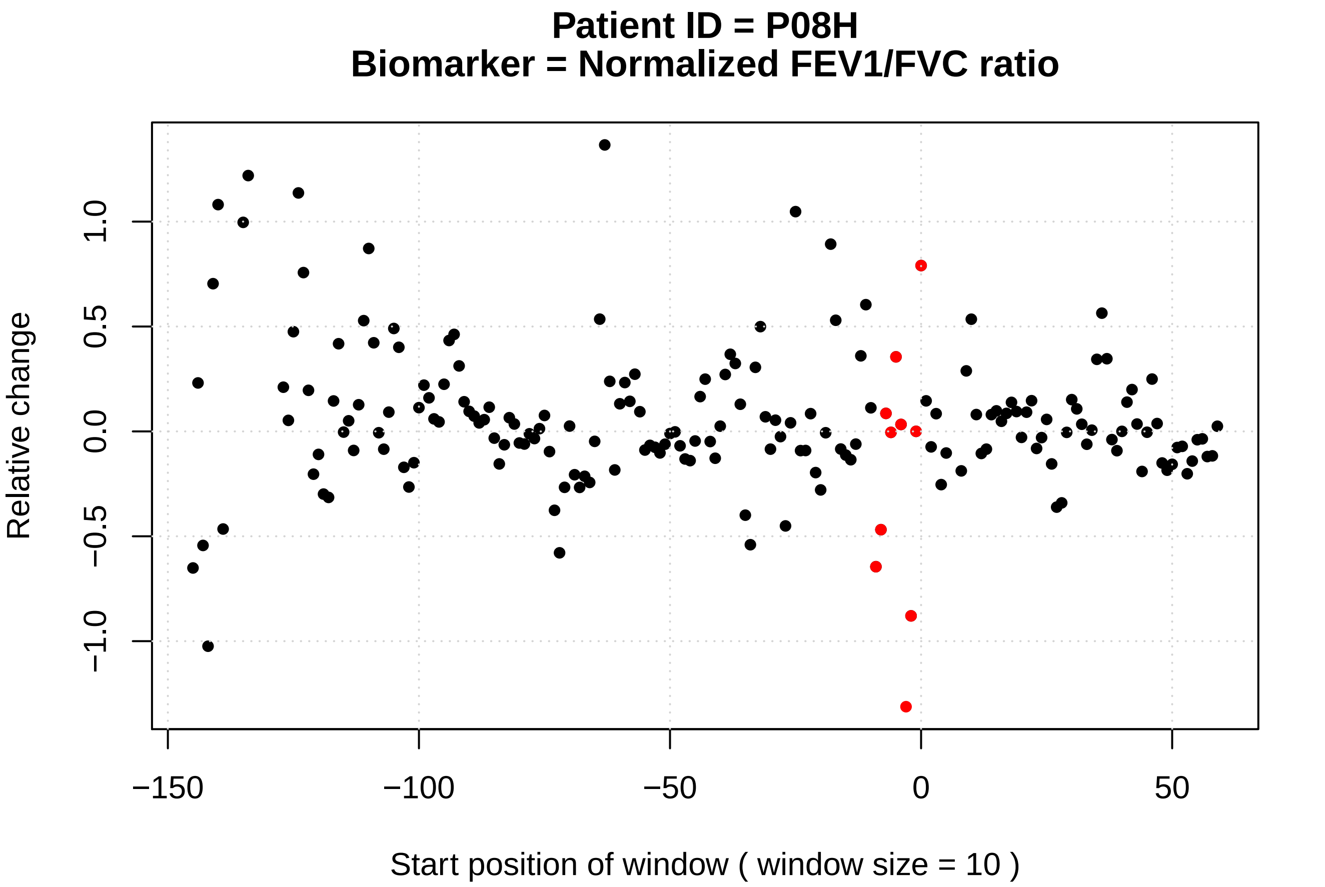

Patient ID = P08H
Biomarker = Normalized FEV1/FVC ratio
Relative change
1.0
0.5
0.0
−0.5
−1.0
−150
−100
−50
0
50
Start position of window (window size = 10)

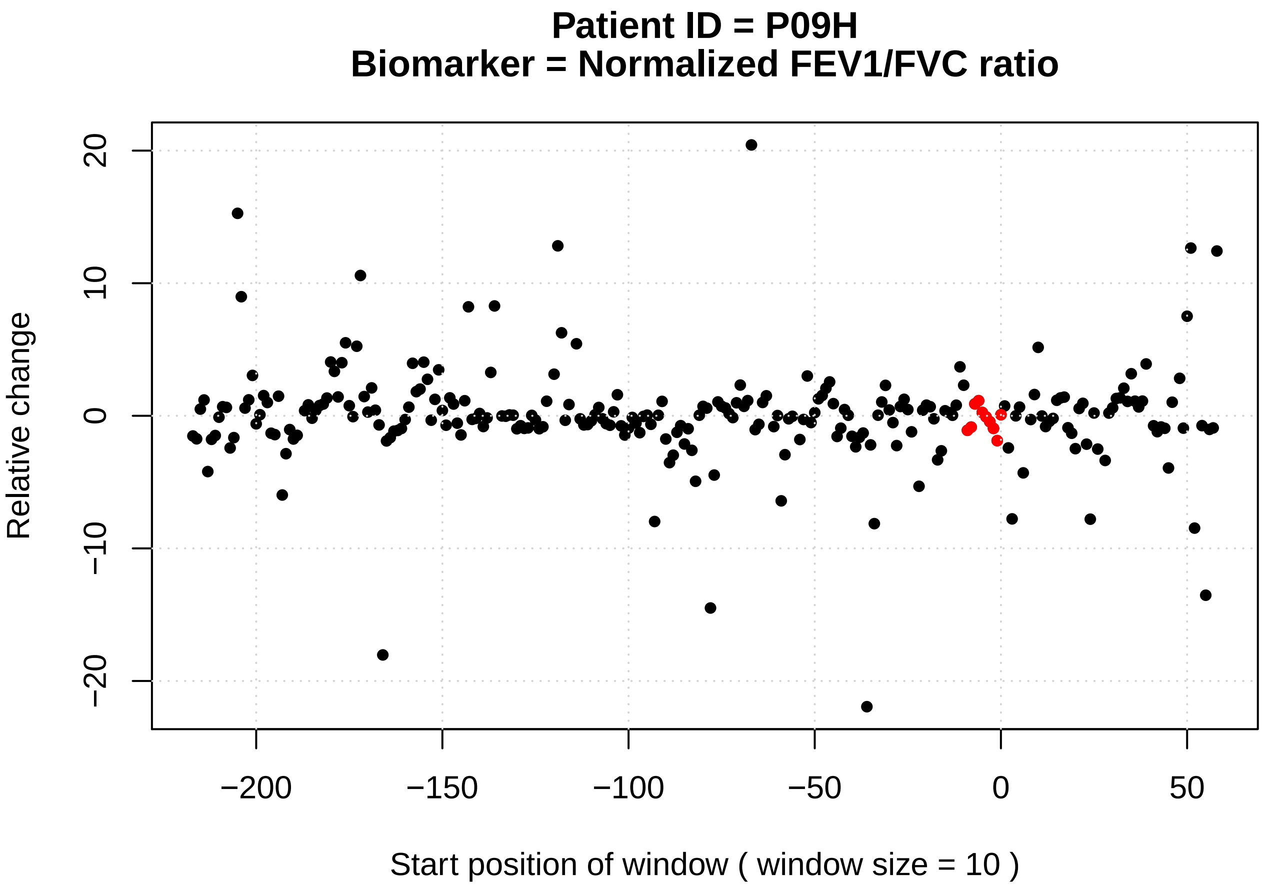

Patient ID = P09H
Biomarker = Normalized FEV1/FVC ratio
Relative change
20
10
0
−10
−20
−200
−150
−100
−50
0
50
Start position of window (window size = 10)

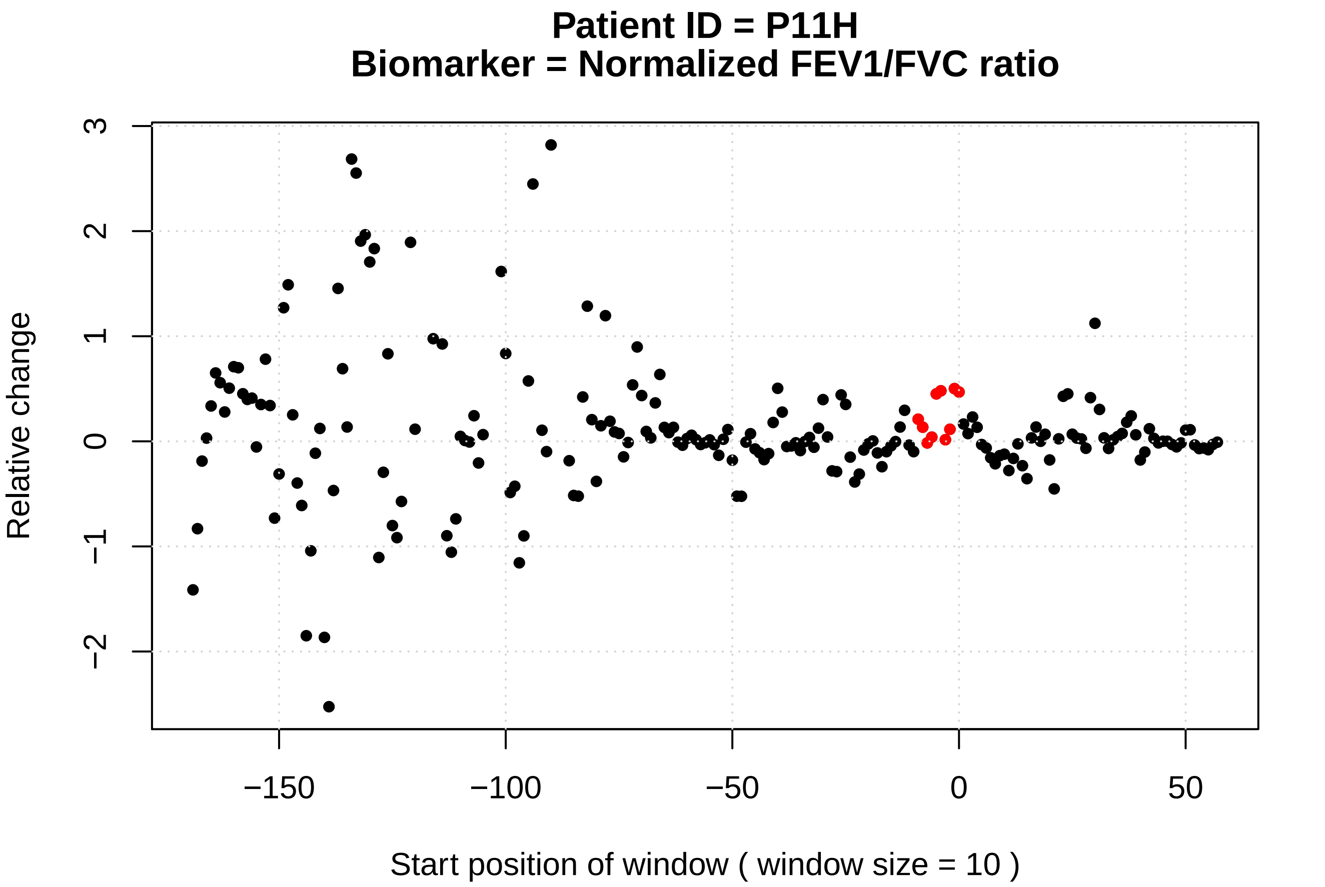
Patient ID = P11H
Biomarker = Normalized FEV1/FVC ratio
Relative change
3
2
1
0
−1
−2
−150
−100
−50
0
50
Start position of window (window size = 10)

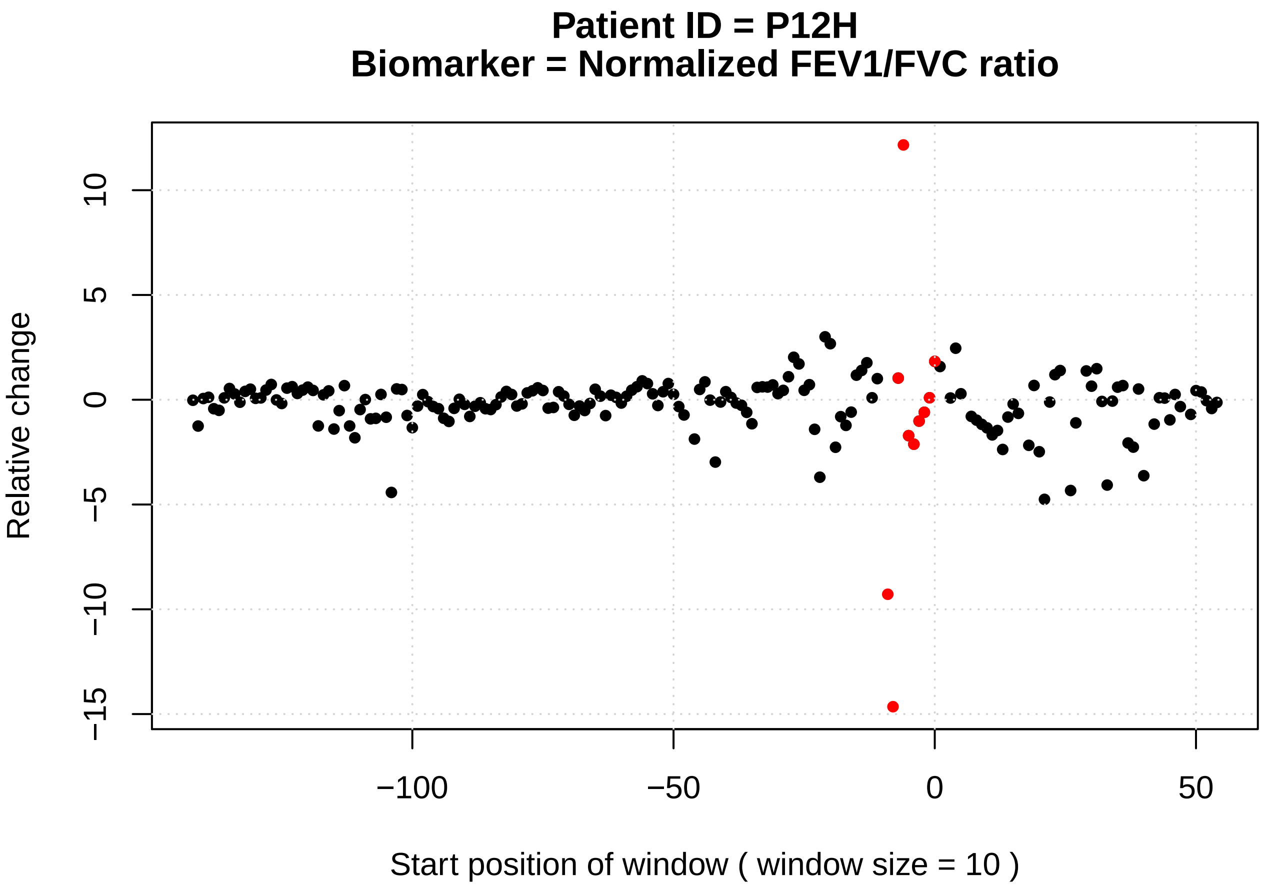

Patient ID = P12H
Biomarker = Normalized FEV1/FVC ratio
Relative change
10
5
0
−5
−10
−15
−100
−50
0
50
Start position of window (window size = 10)

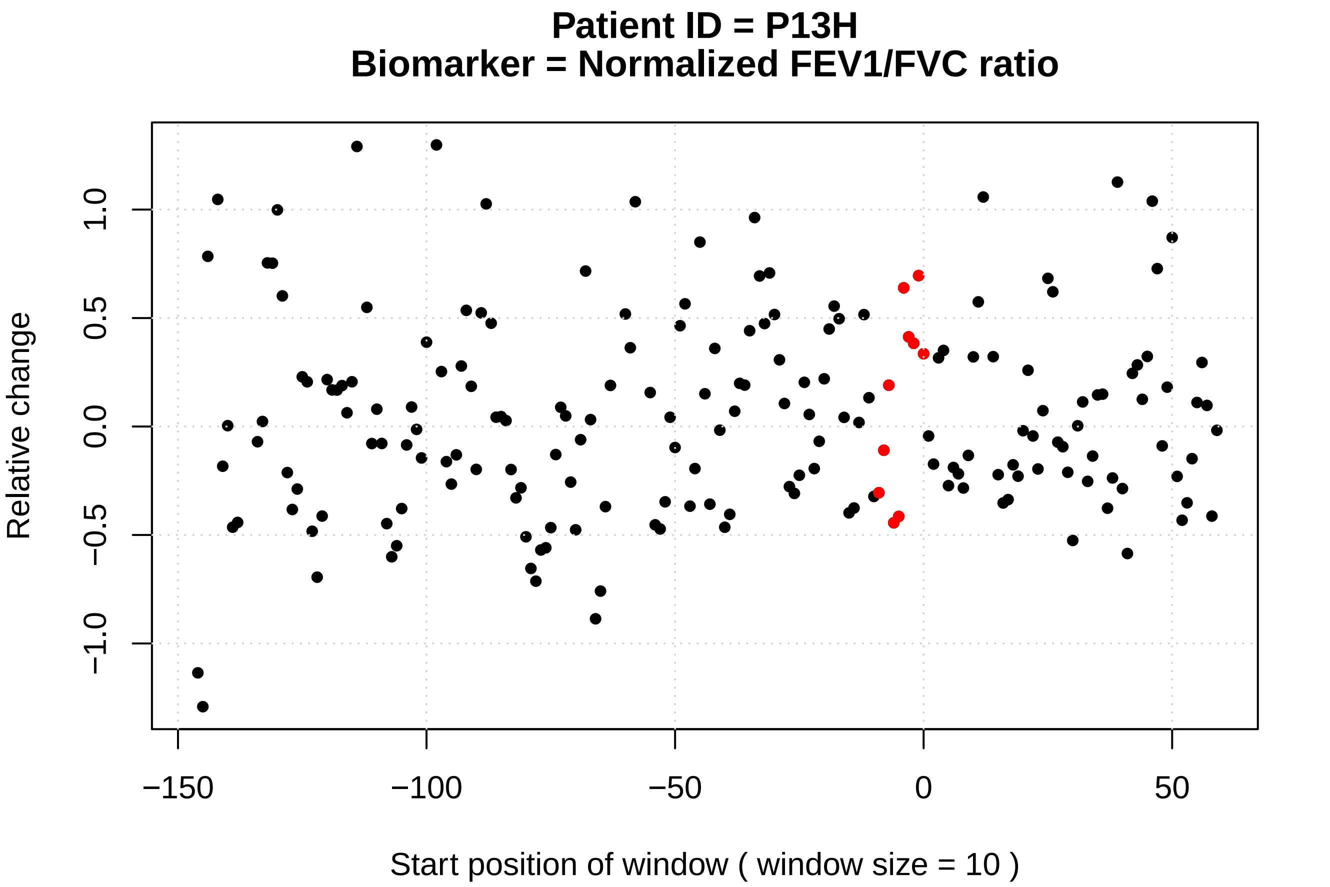

Patient ID = P13H
Biomarker = Normalized FEV1/FVC ratio
Relative change
−1.0
−0.5
0.0
0.5
1.0
−150
−100
−50
0
50
Start position of window (window size = 10)

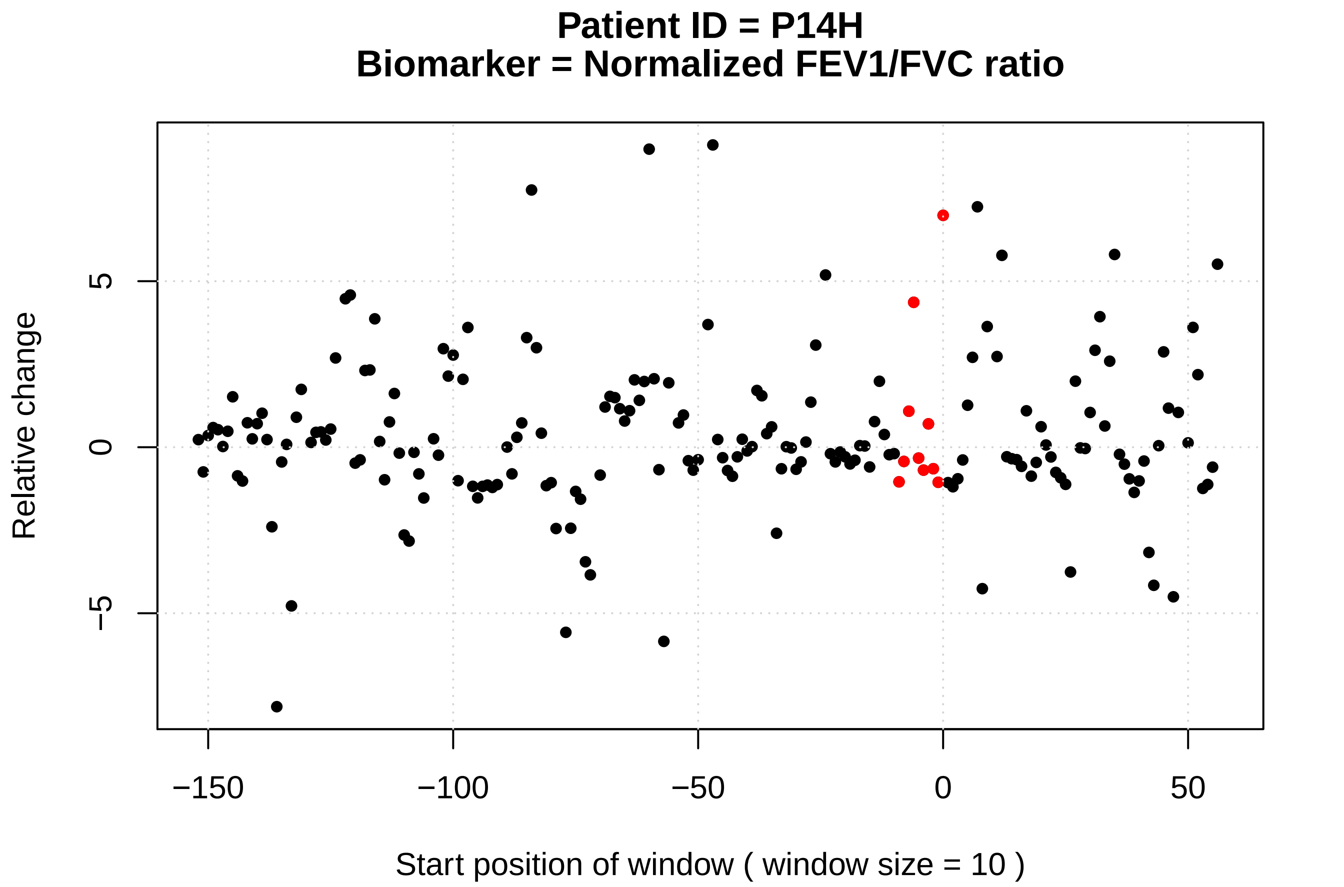
Patient ID = P14H
Biomarker = Normalized FEV1/FVC ratio
Relative change
5
0
−5
−150
−100
−50
0
50
Start position of window (window size = 10)

Patient ID = P15H
Biomarker = Normalized FEV1/FVC ratio

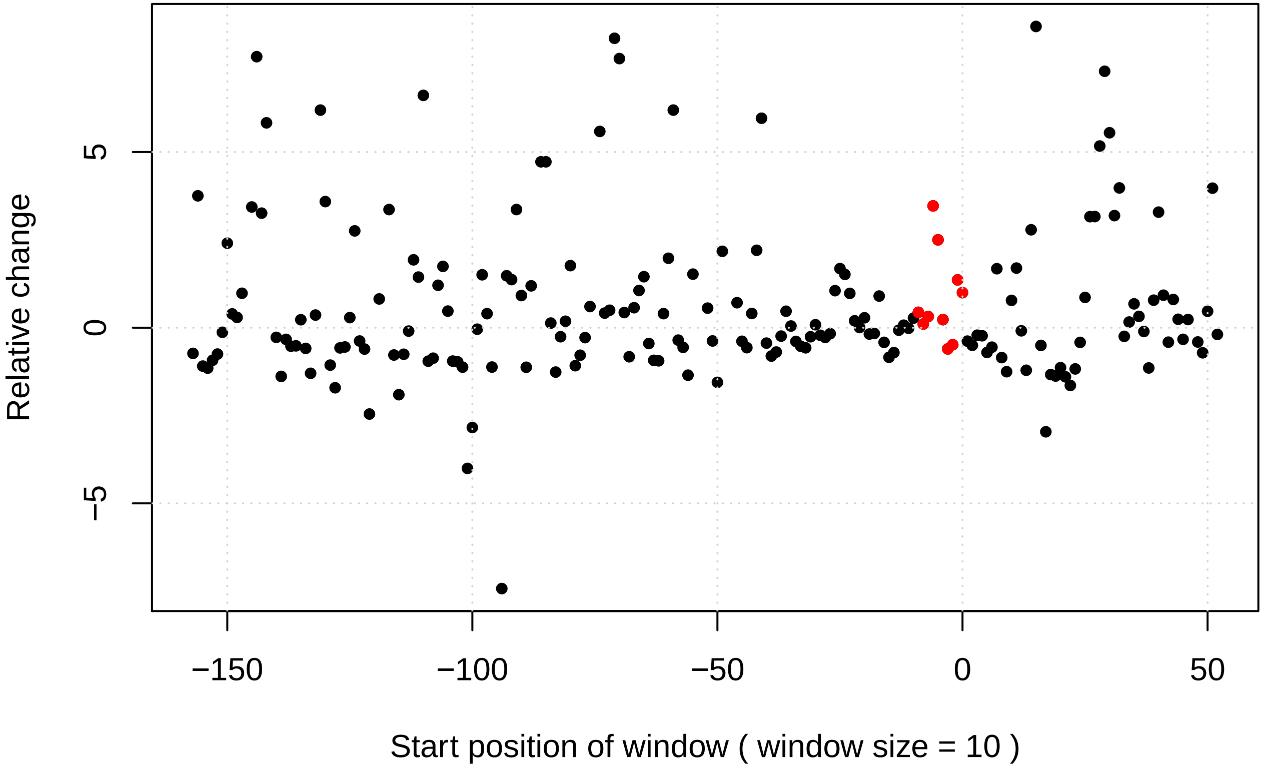

Supplement: Supplementary file 1. [file elife-47969-supp1.zip › RelativeChangeWithinWindowPlots_PDFs/Appendix-figure SS23 IndividualWindowBoundariesRelativeChangeAnalysis NFEV1FVC Window Size = 10 HealthyParticipants.pdf]
