# Supplementary material for: Loss of adaptive capacity in asthmatic patients revealed by biomarker fluctuation dynamics after rhinovirus challenge
Source: eLife. 2019 Nov 5;8:e47969. doi: 10.7554/eLife.47969 (PMC6877087; doi:10.7554/eLife.47969)
Supplement: Supplementary file 1. [file elife-47969-supp1.zip › RelativeChangeWithinWindowPlots_PDFs/Appendix-figure SS19 IndividualWindowBoundariesRelativeChangeAnalysis NFEV1 Window Size = 10 HealthyParticipants.pdf]

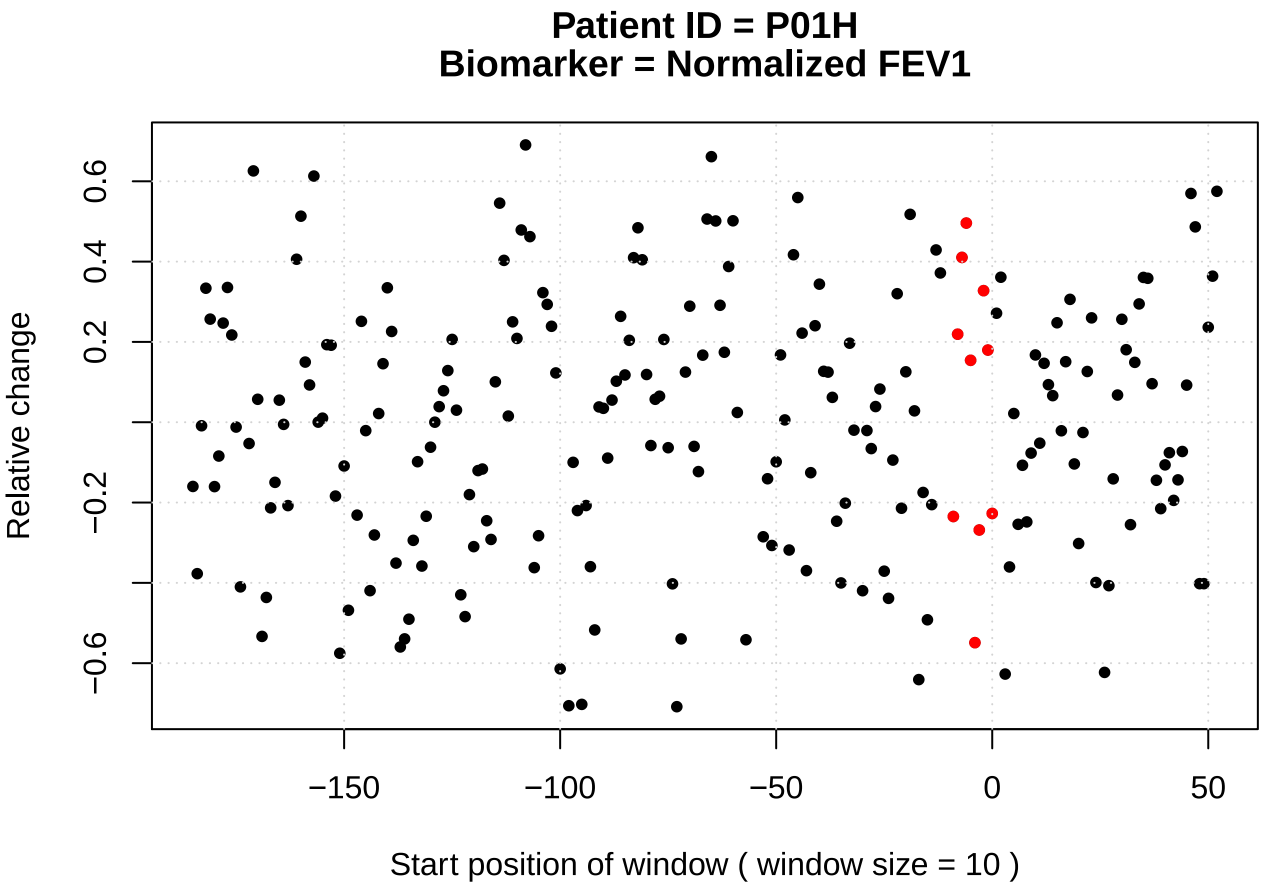
Patient ID = P01H
Biomarker = Normalized FEV1
Relative change
0.6
0.4
0.2
−0.2
−0.6
−150
−100
−50
0
50
Start position of window (window size = 10)

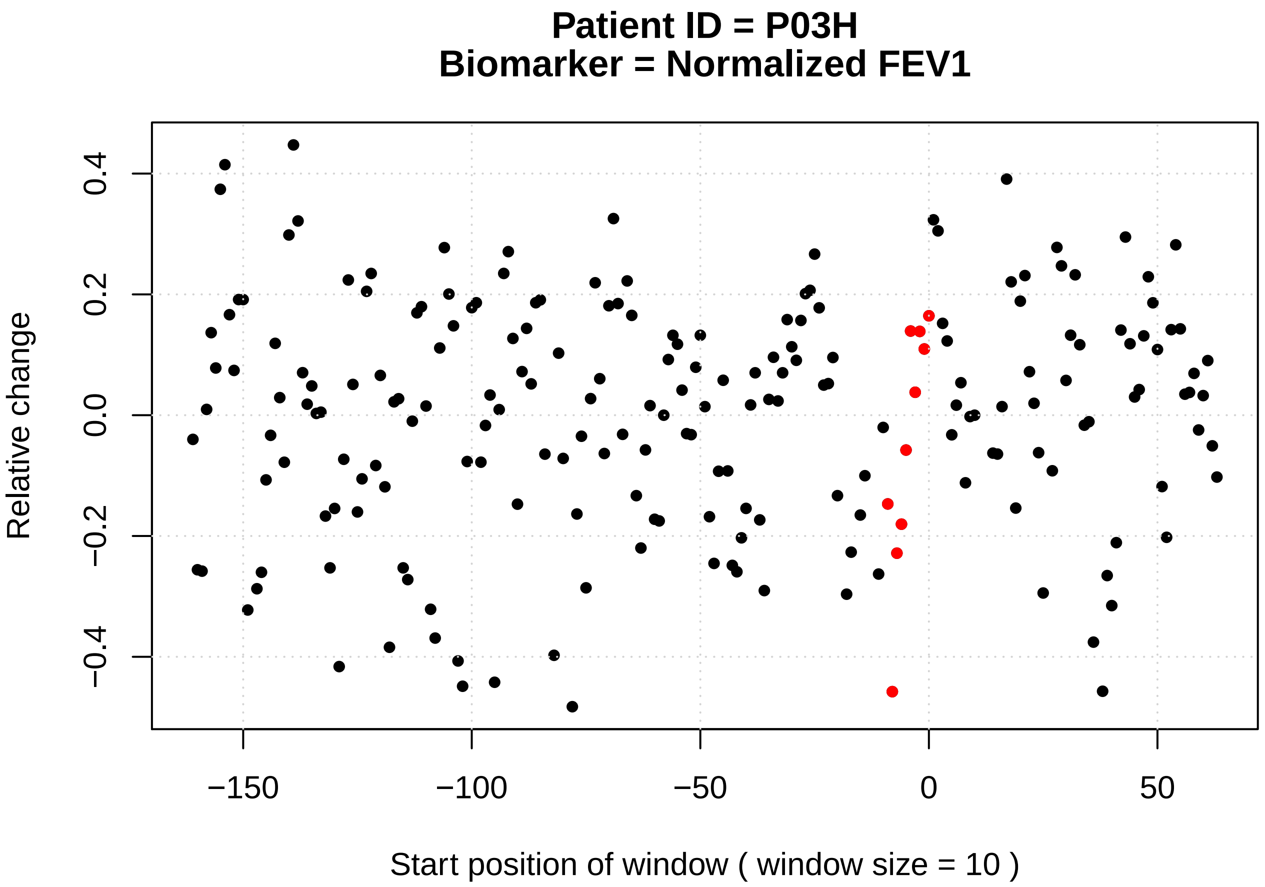
Patient ID = P03H
Biomarker = Normalized FEV1
Relative change
0.4
0.2
0.0
−0.2
−0.4
−150
−100
−50
0
50
Start position of window (window size = 10)

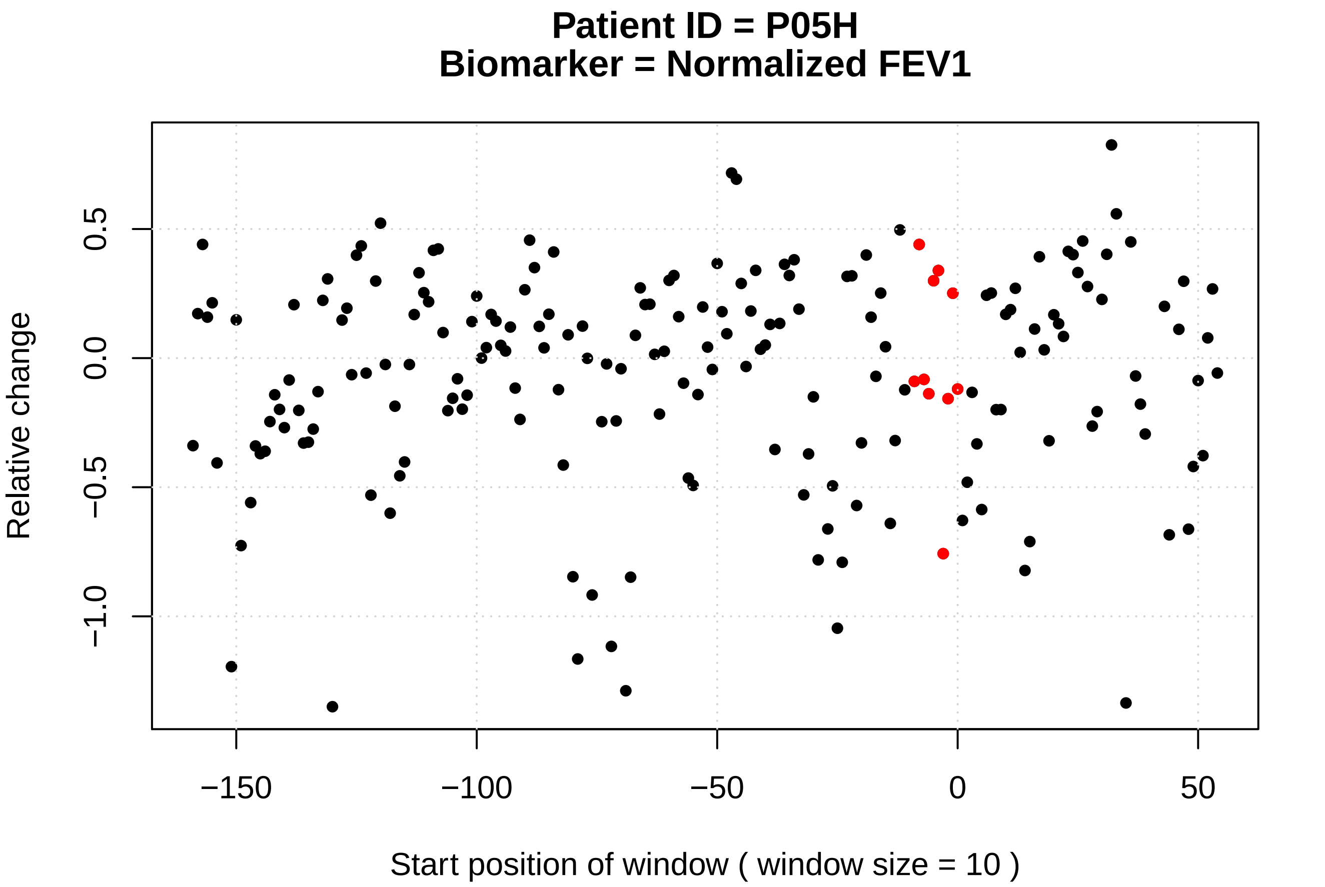

Patient ID = P05H
Biomarker = Normalized FEV1
Relative change
0.5
0.0
−0.5
−1.0
−150
−100
−50
0
50
Start position of window (window size = 10)

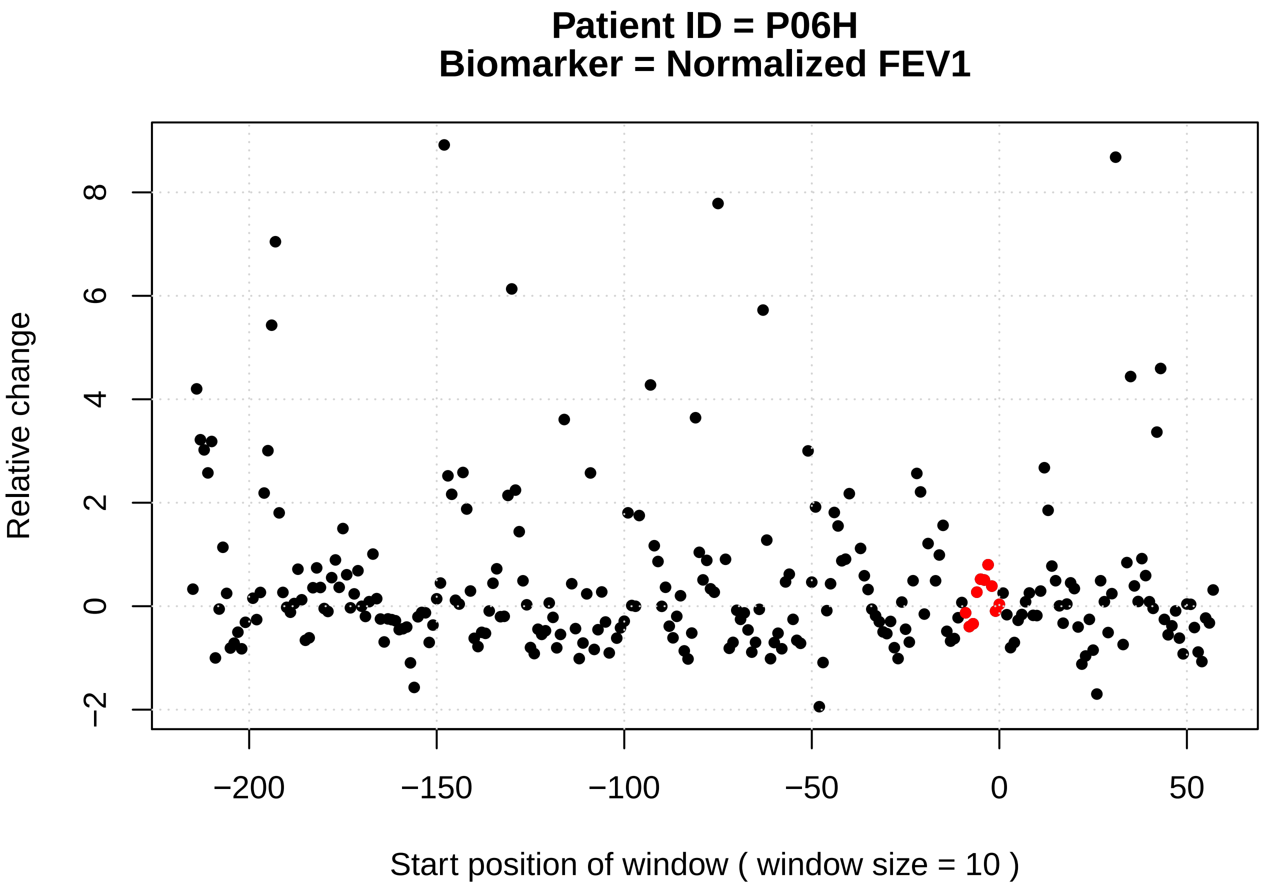
Patient ID = P06H
Biomarker = Normalized FEV1
Relative change
Start position of window (window size = 10)
−2
0
2
4
6
8
−200
−150
−100
−50
0
50

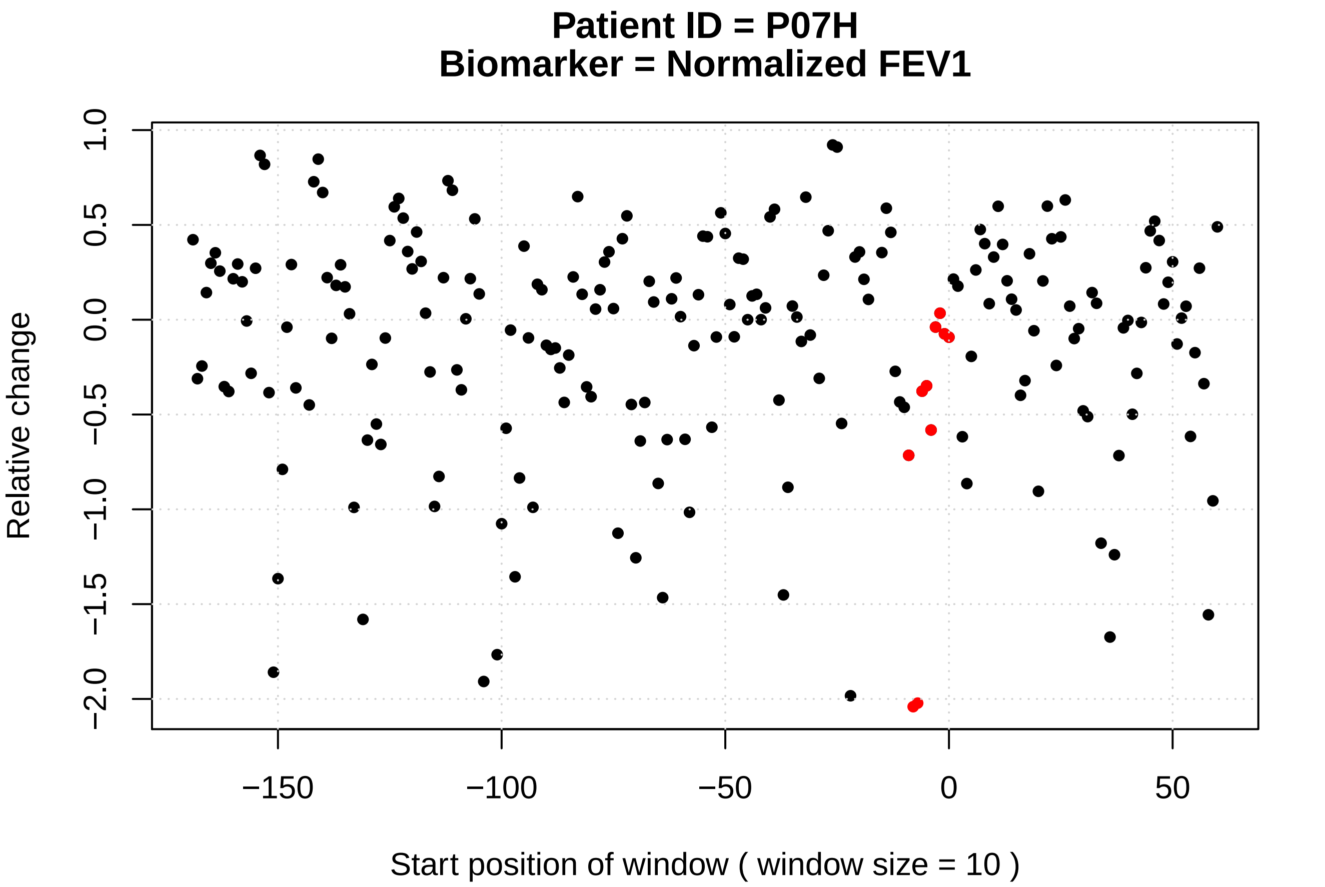

Patient ID = P07H
Biomarker = Normalized FEV1
Relative change
1.0
0.5
0.0
−0.5
−1.0
−1.5
−2.0
−150
−100
−50
0
50
Start position of window (window size = 10)

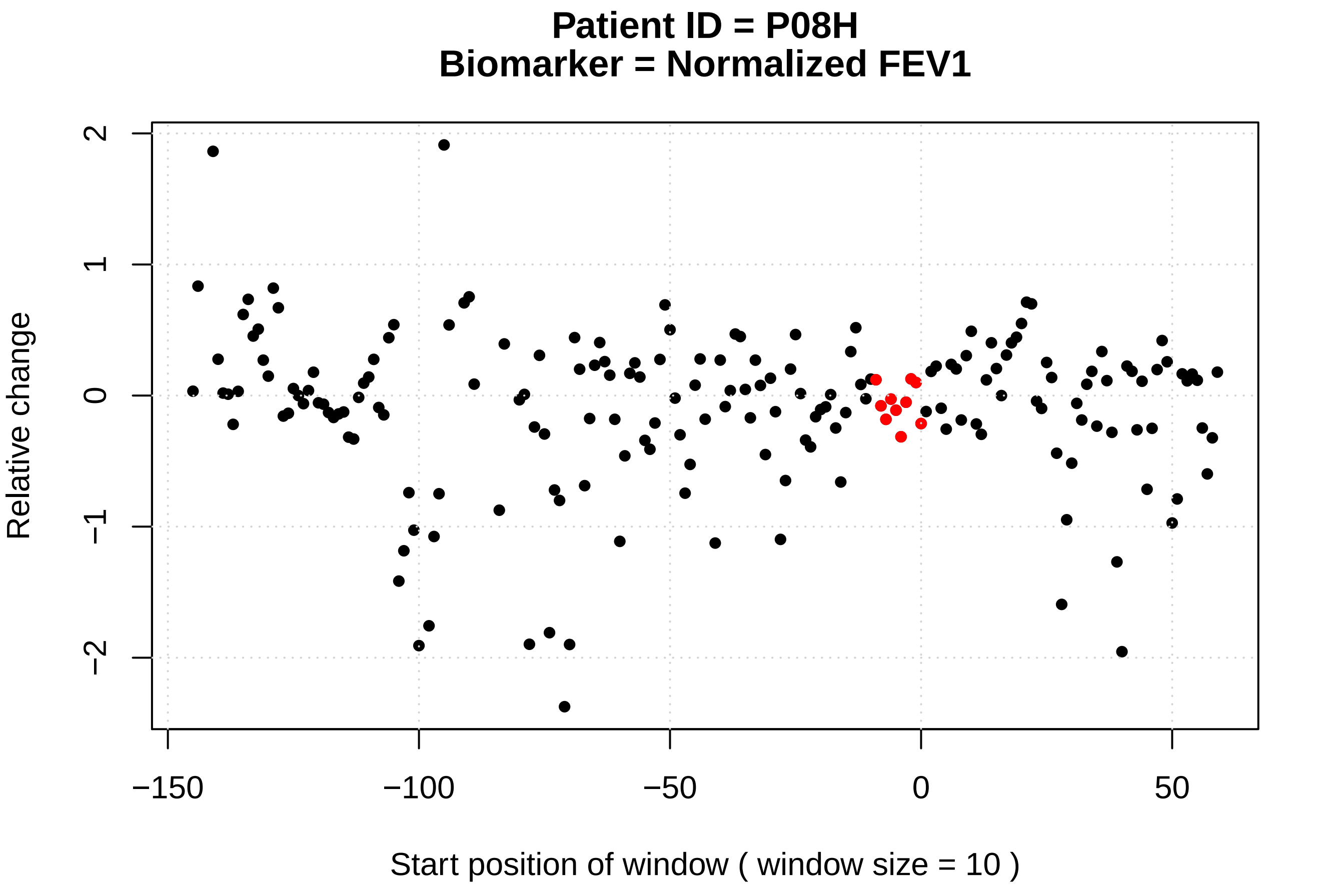

Patient ID = P08H
Biomarker = Normalized FEV1
Relative change
2
1
0
−1
−2
−150
−100
−50
0
50
Start position of window (window size = 10)

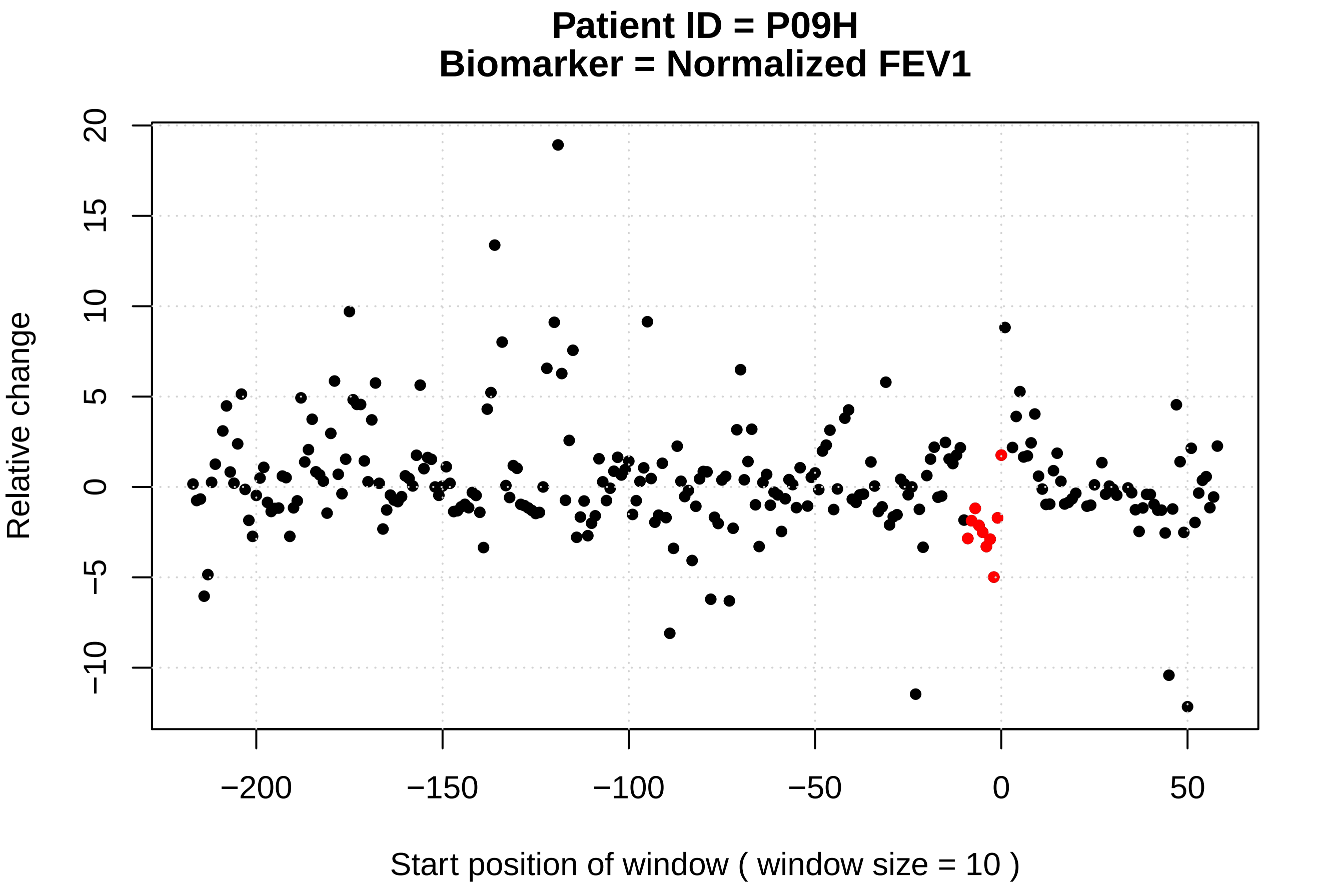
Patient ID = P09H
Biomarker = Normalized FEV1
Relative change
20
15
10
5
0
−5
−10
−200
−150
−100
−50
0
50
Start position of window (window size = 10)

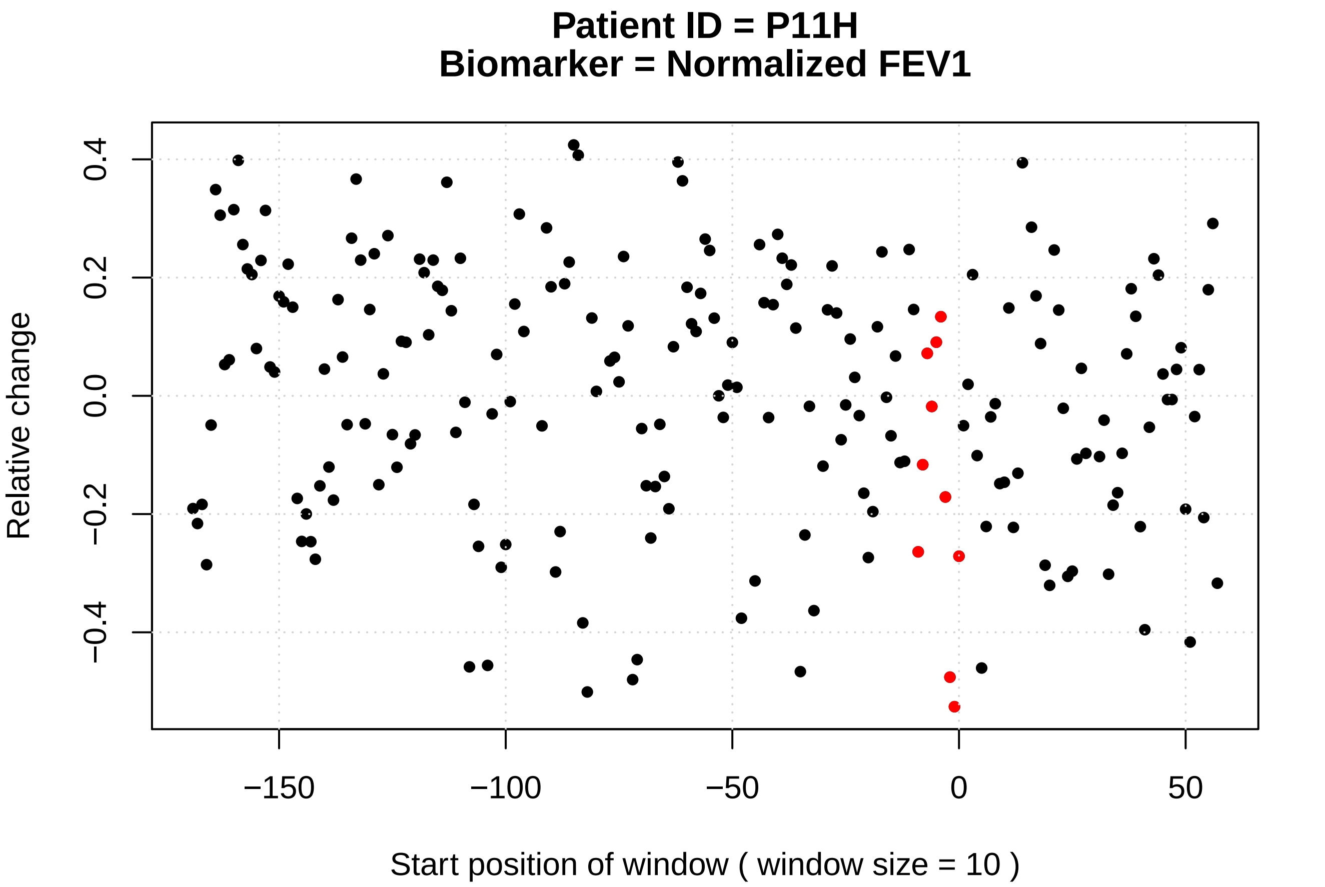
Patient ID = P11H
Biomarker = Normalized FEV1
Relative change
0.4
0.2
0.0
−0.2
−0.4
−150
−100
−50
0
50
Start position of window (window size = 10)

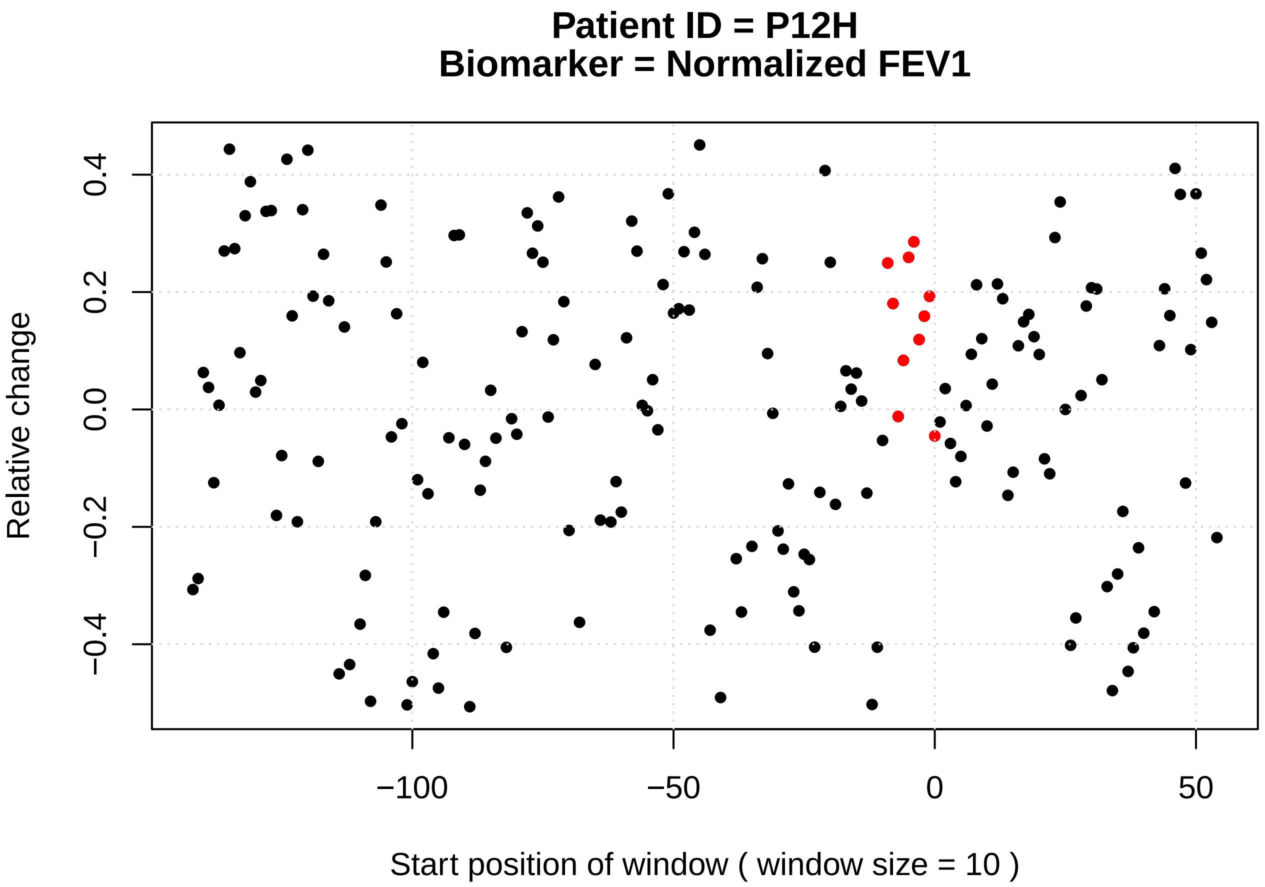
Patient ID = P12H
Biomarker = Normalized FEV1
Relative change
0.4
0.2
0.0
−0.2
−0.4
−100
−50
0
50
Start position of window (window size = 10)

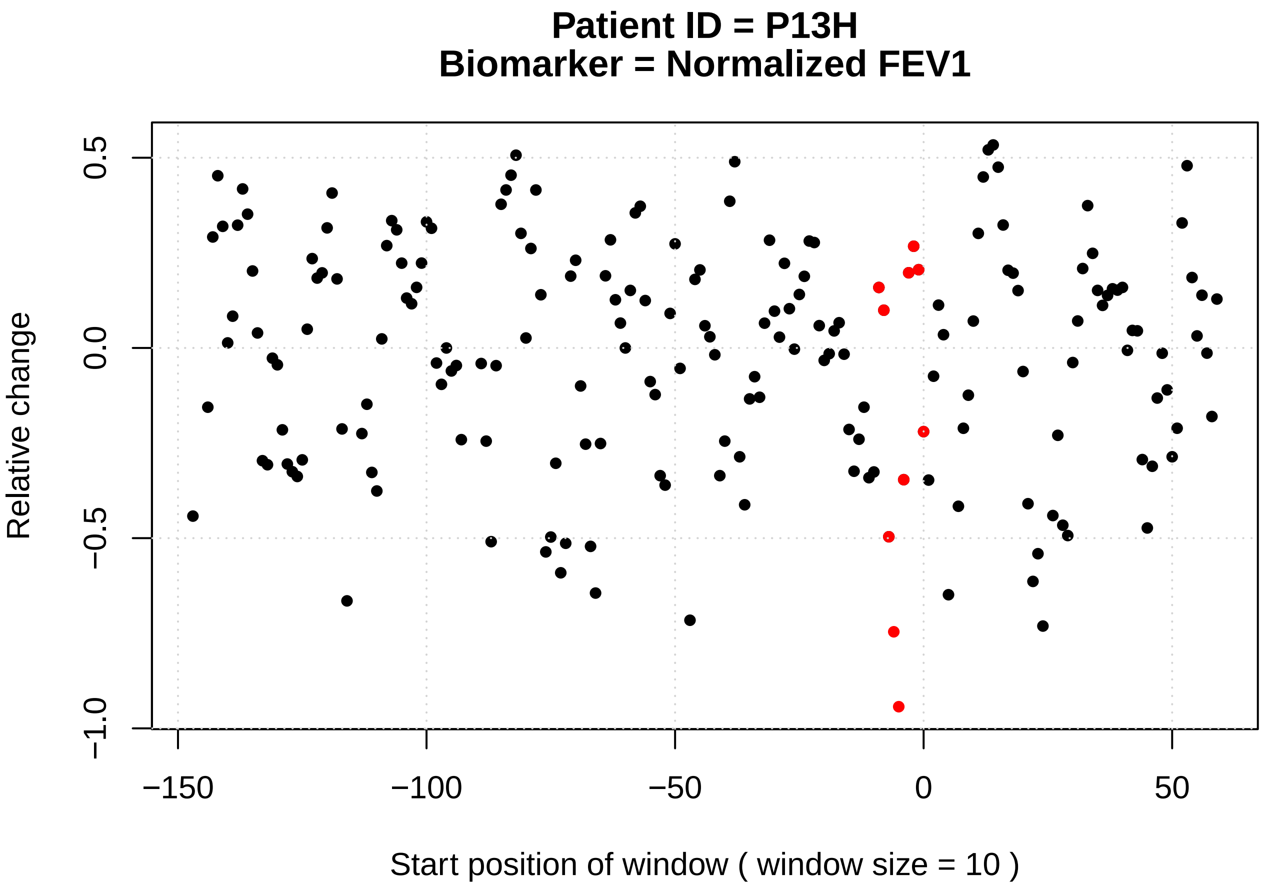
Patient ID = P13H
Biomarker = Normalized FEV1
Relative change
0.5
0.0
−0.5
−1.0
−150
−100
−50
0
50
Start position of window (window size = 10)

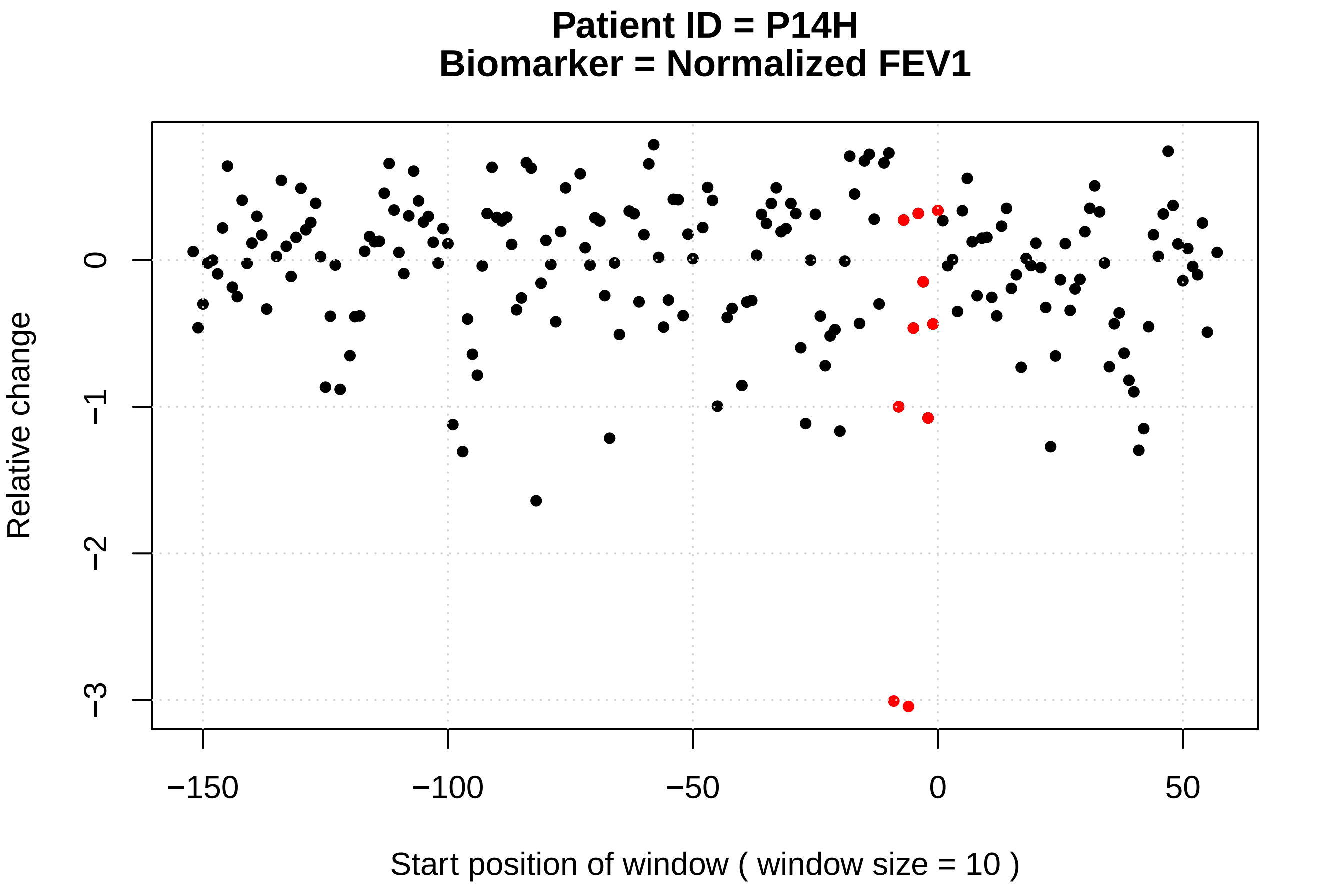
Patient ID = P14H
Biomarker = Normalized FEV1
Relative change
0
−1
−2
−3
−150
−100
−50
0
50
Start position of window (window size = 10)

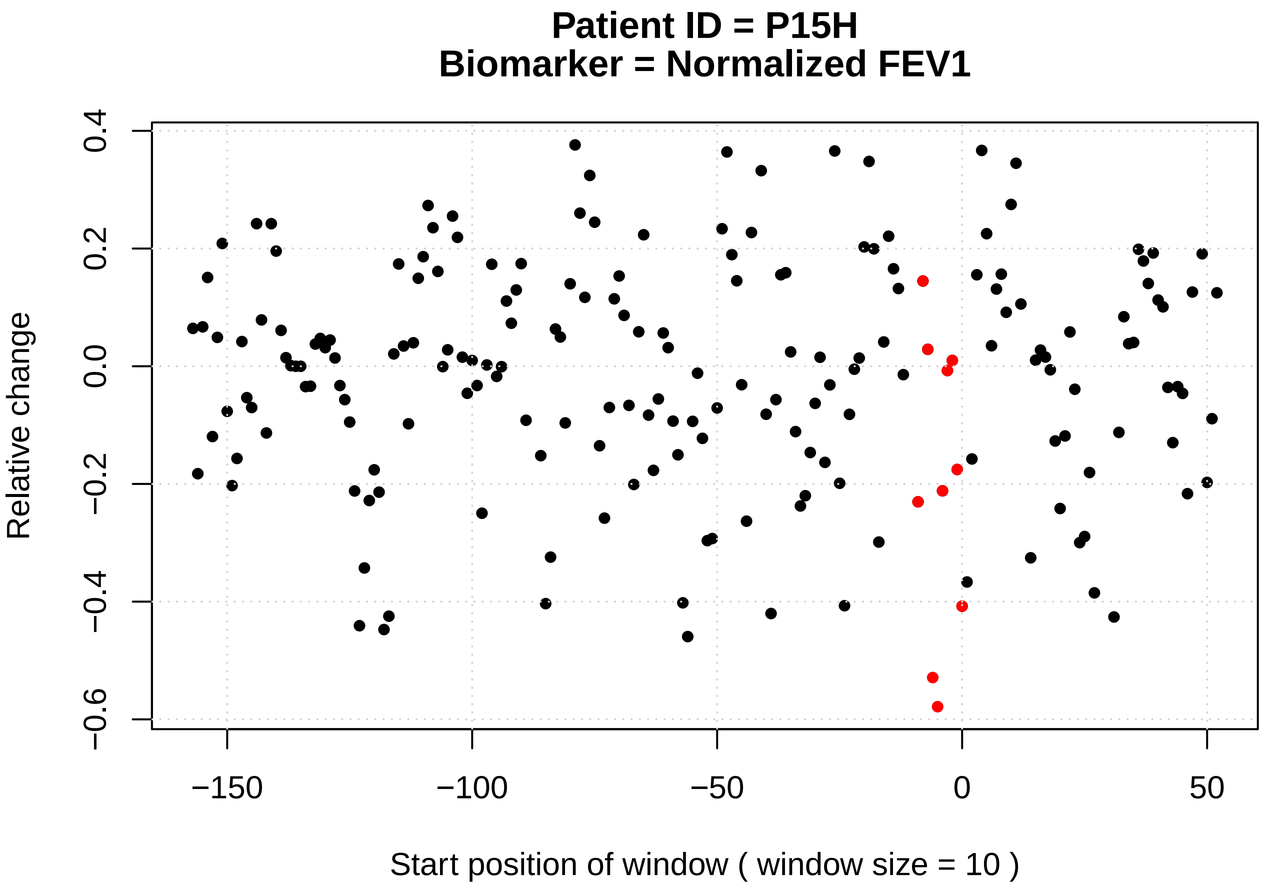

Patient ID = P15H
Biomarker = Normalized FEV1
Relative change
Start position of window (window size = 10)
0.4
0.2
0.0
−0.2
−0.4
−0.6
−150
−100
−50
0
50
