# Supplementary material for: Loss of adaptive capacity in asthmatic patients revealed by biomarker fluctuation dynamics after rhinovirus challenge
Source: eLife. 2019 Nov 5;8:e47969. doi: 10.7554/eLife.47969 (PMC6877087; doi:10.7554/eLife.47969)
Supplement: Supplementary file 1. [file elife-47969-supp1.zip › RelativeChangeWithinWindowPlots_PDFs/Appendix-figure SS27 IndividualWindowBoundariesRelativeChangeAnalysis CellDensity_In10e6_Per_ml Window Size = 10 HealthyParticipants.pdf]

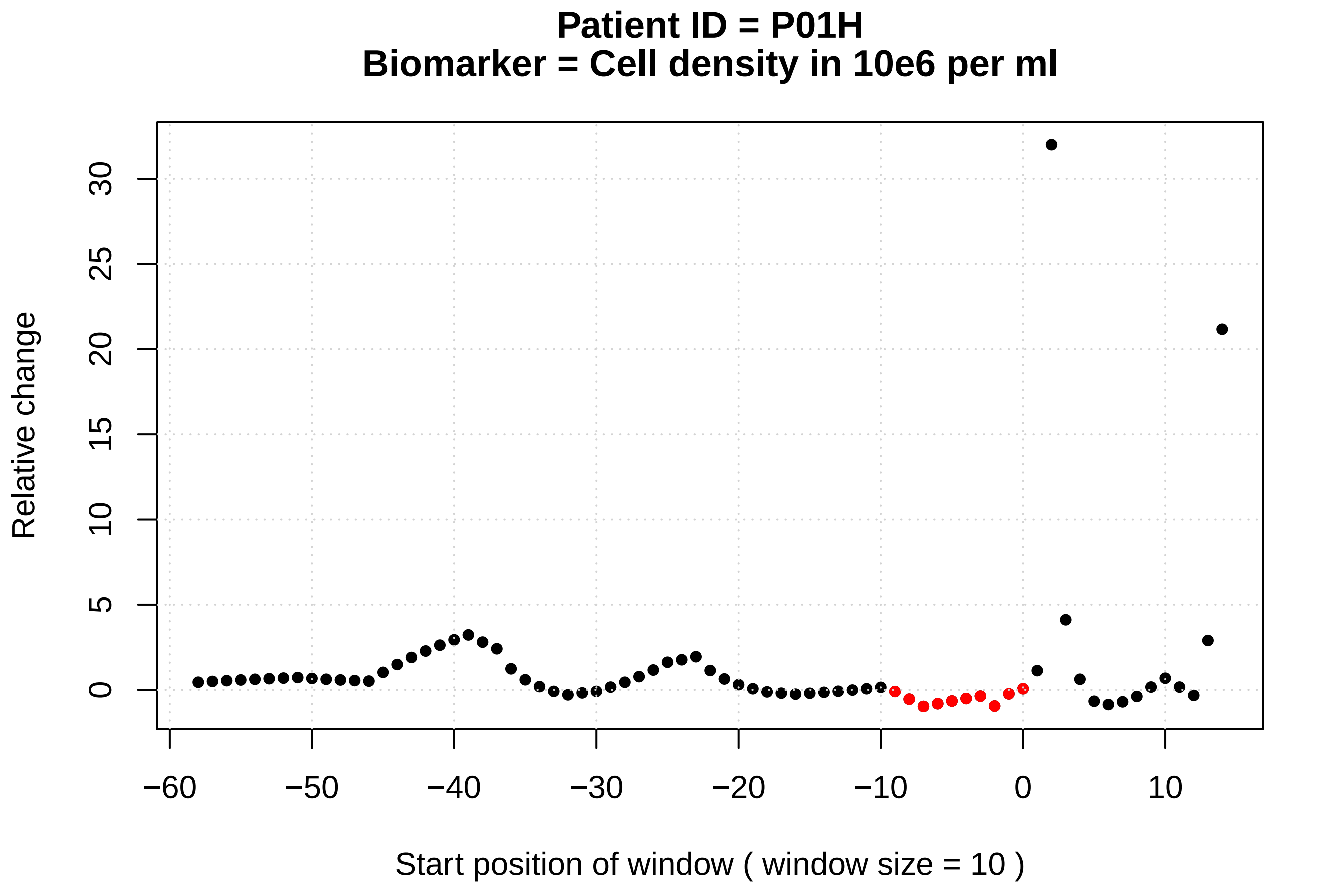

Patient ID = P01H
Biomarker = Cell density in 10e6 per ml
Relative change
0
5
10
15
20
25
30
−60
−50
−40
−30
−20
−10
0
10
Start position of window (window size = 10)

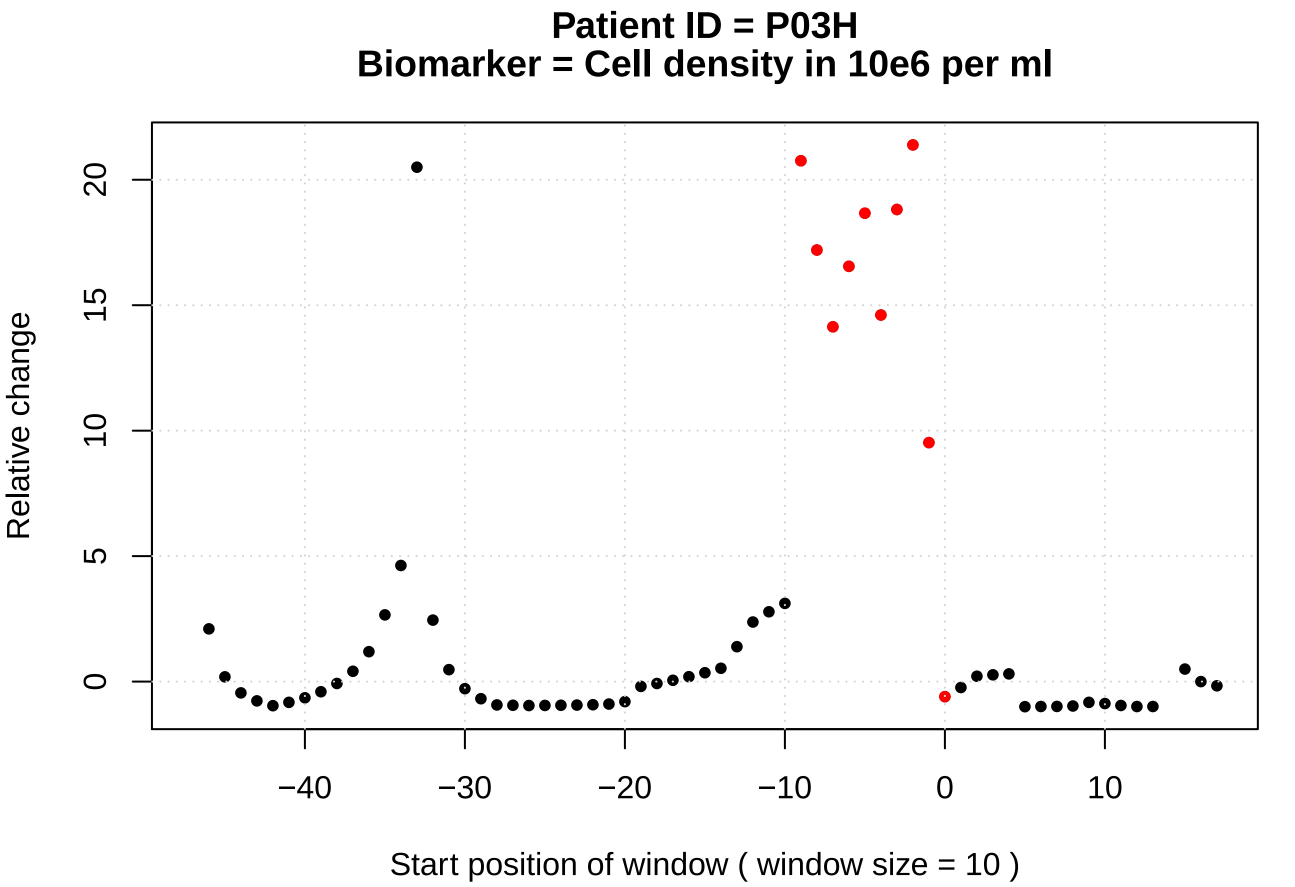
Patient ID = P03H
Biomarker = Cell density in 10e6 per ml
Relative change
0
5
10
15
20
−40
−30
−20
−10
0
10
Start position of window (window size = 10)

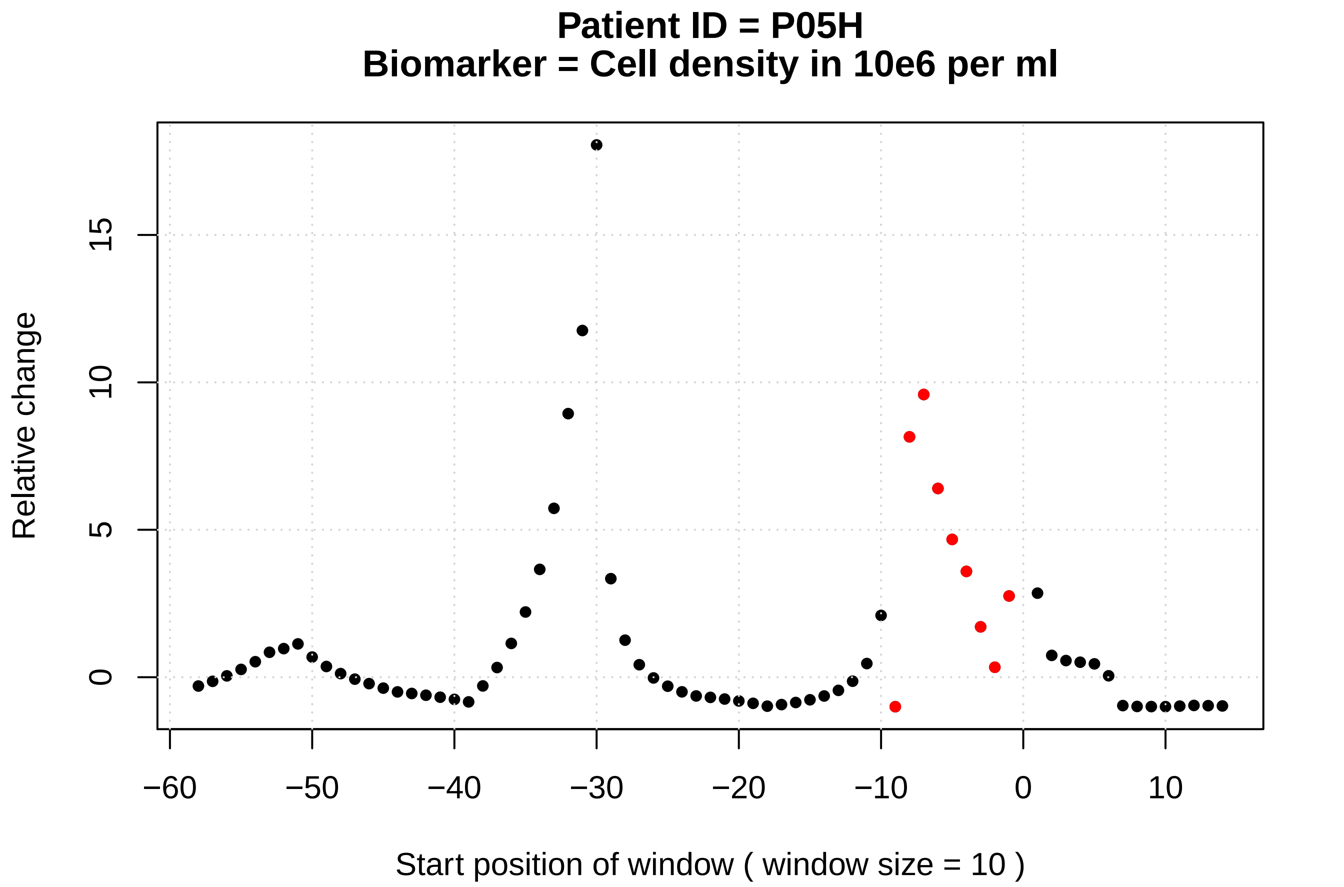
Patient ID = P05H
Biomarker = Cell density in 10e6 per ml
Relative change
0
5
10
15
−60
−50
−40
−30
−20
−10
0
10
Start position of window (window size = 10)

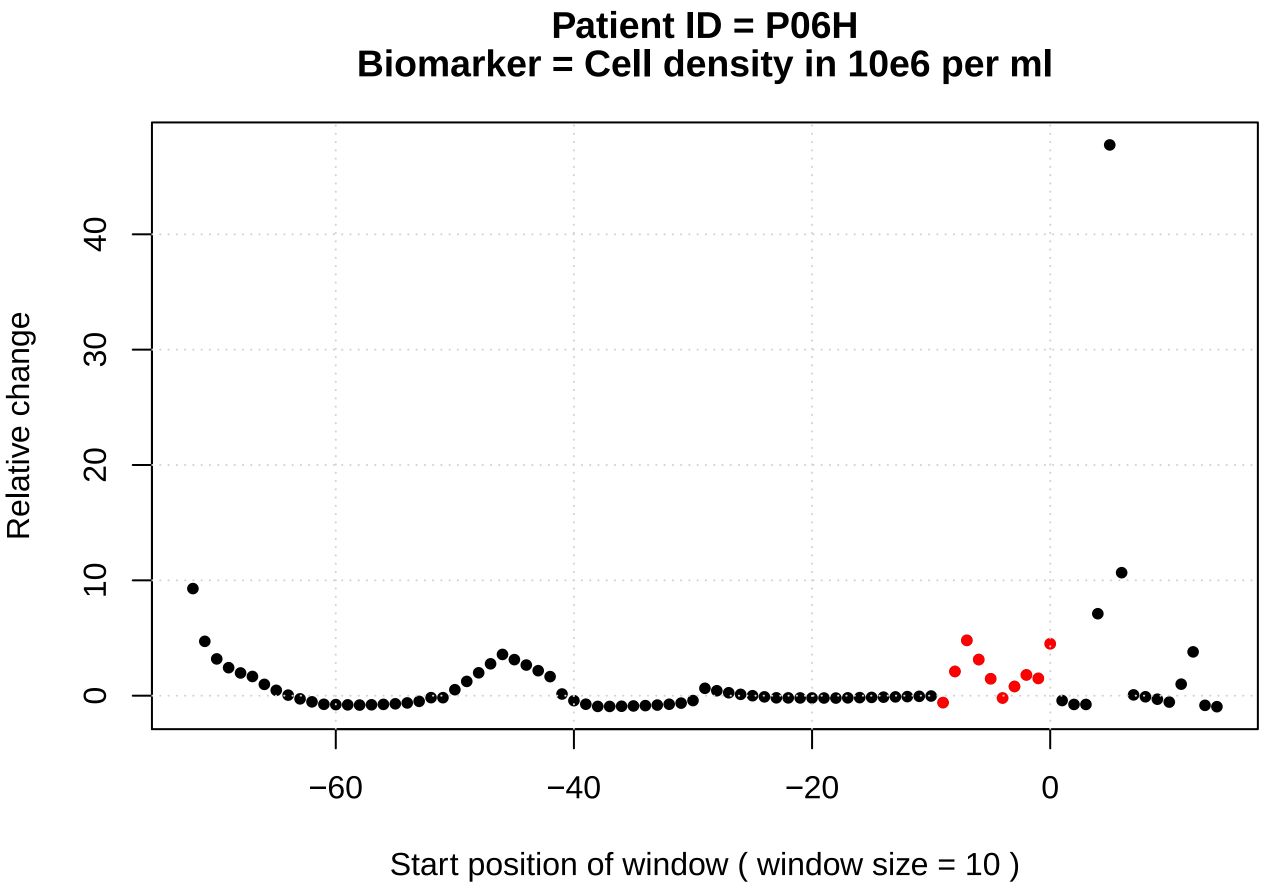

Patient ID = P06H
Biomarker = Cell density in 10e6 per ml
Relative change
0
10
20
30
40
−60
−40
−20
0
Start position of window (window size = 10)

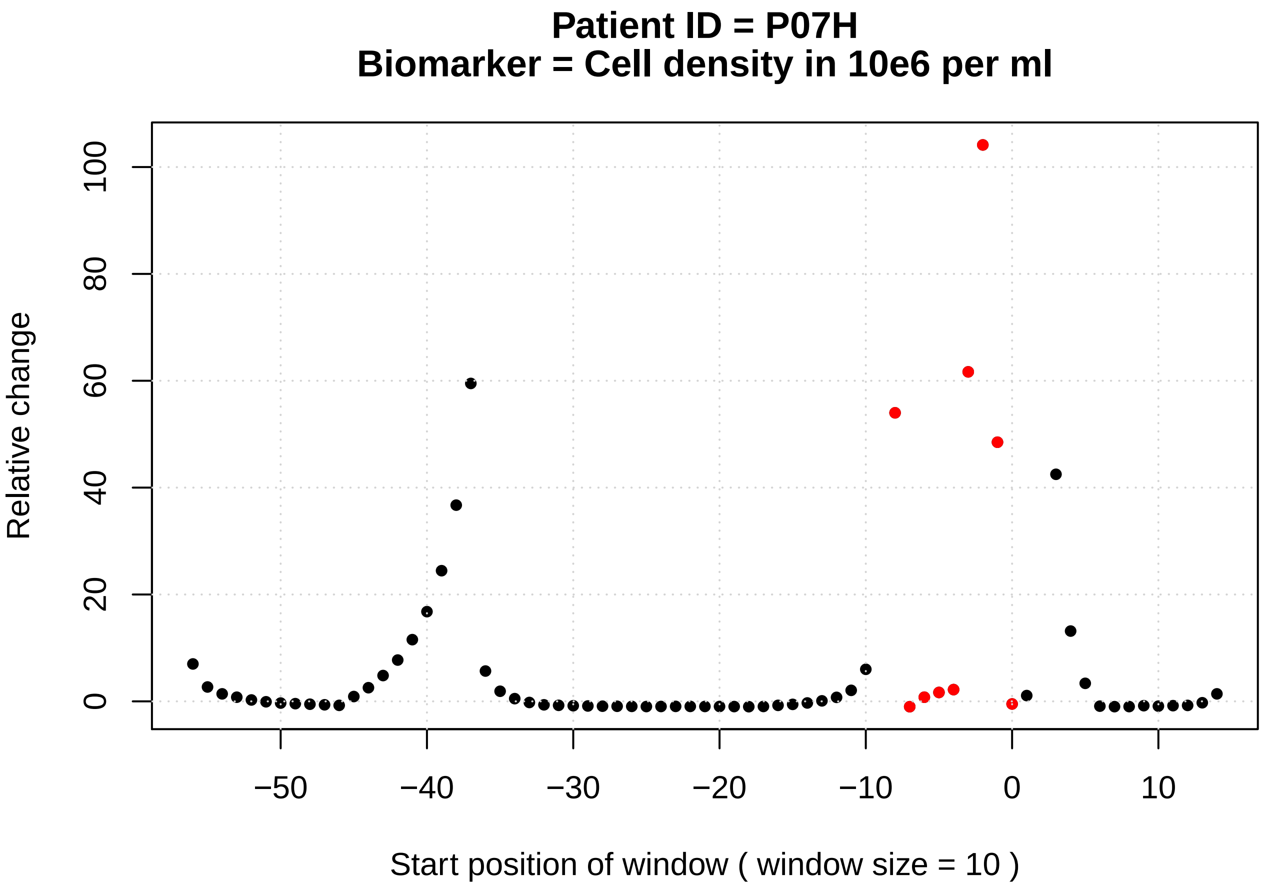
Patient ID = P07H
Biomarker = Cell density in 10e6 per ml
Relative change
0
20
40
60
80
100
−50
−40
−30
−20
−10
0
10
Start position of window (window size = 10)

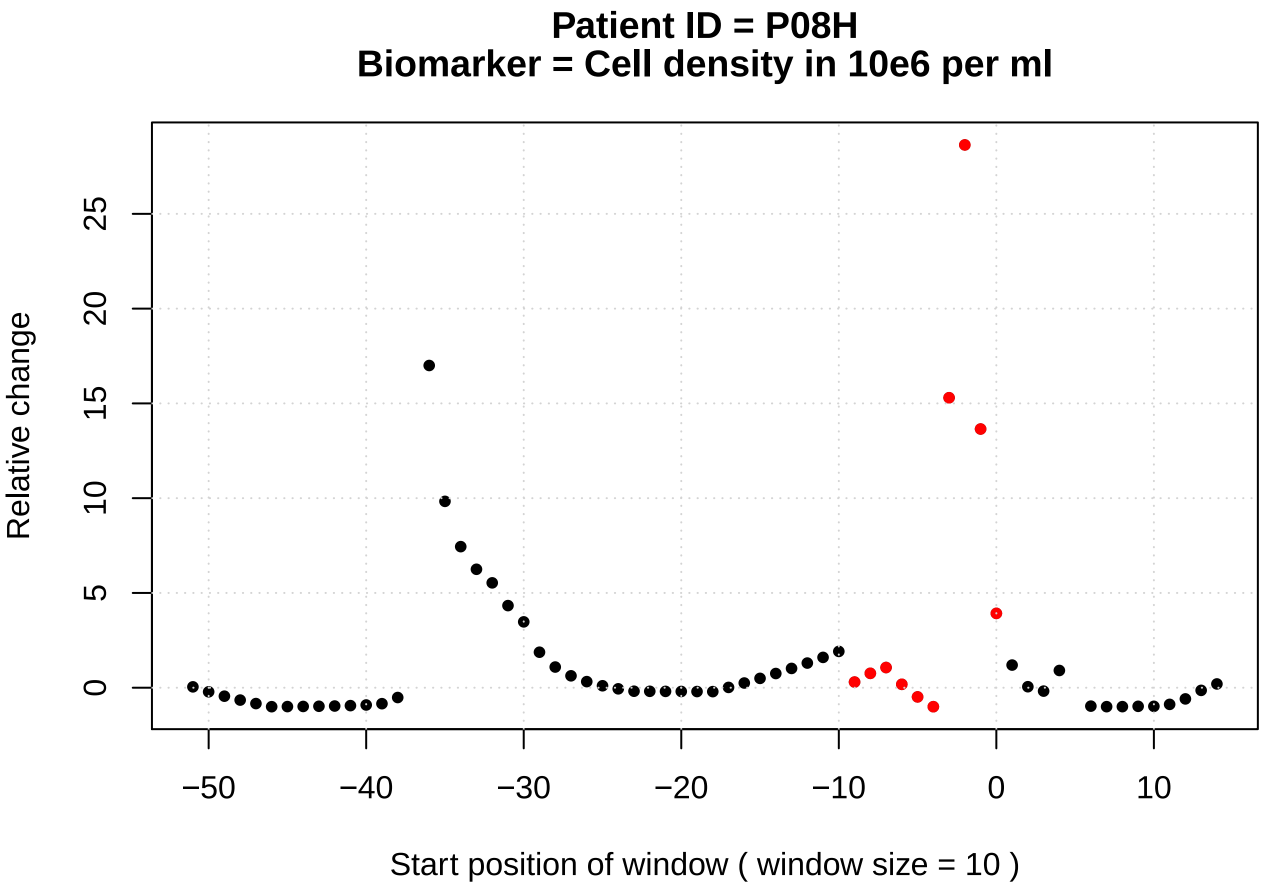

Patient ID = P08H
Biomarker = Cell density in 10e6 per ml
Relative change
Start position of window (window size = 10)
0
5
10
15
20
25
−50
−40
−30
−20
−10
0
10

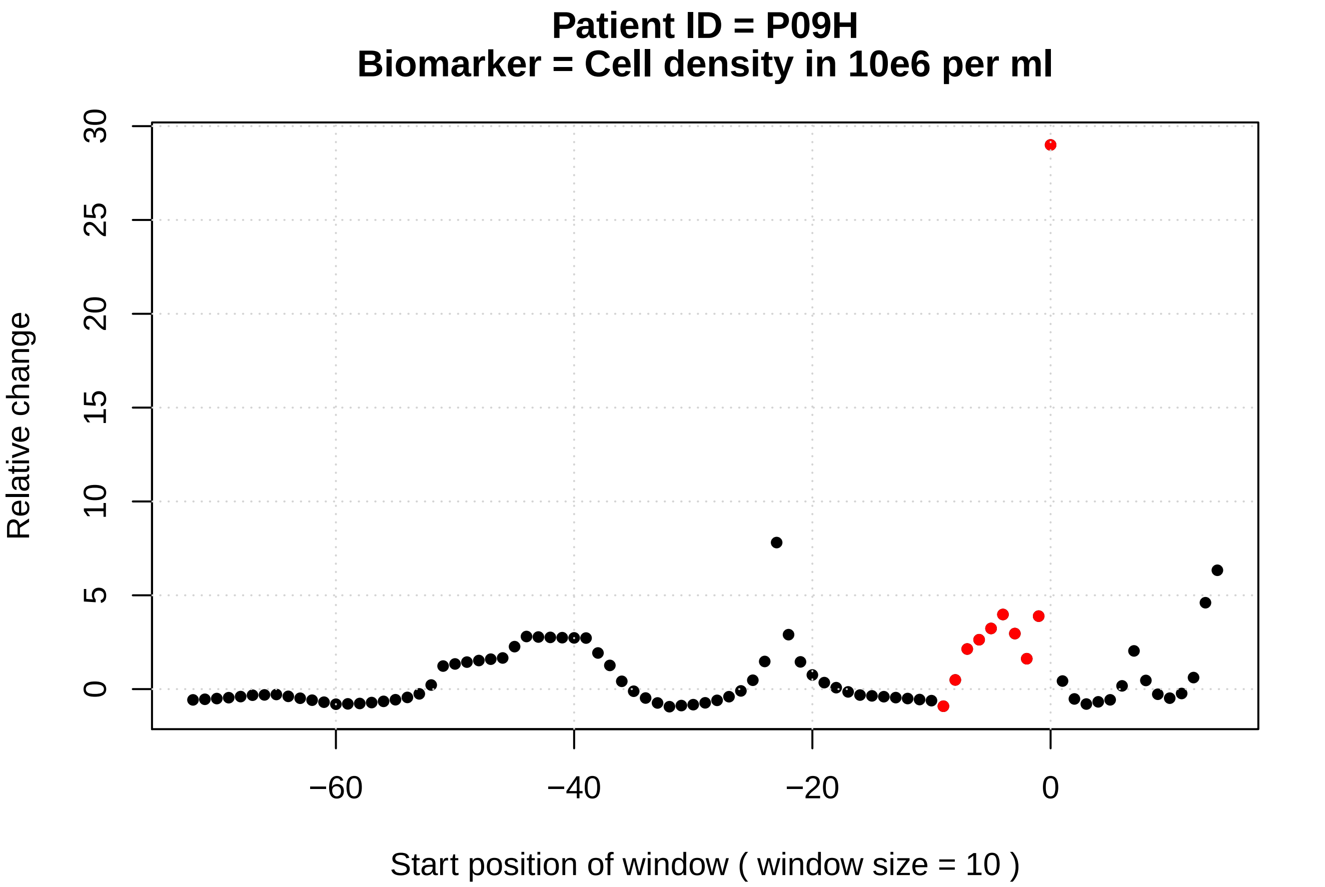
Patient ID = P09H
Biomarker = Cell density in 10e6 per ml
Relative change
0
5
10
15
20
25
30
−60
−40
−20
0
Start position of window (window size = 10)

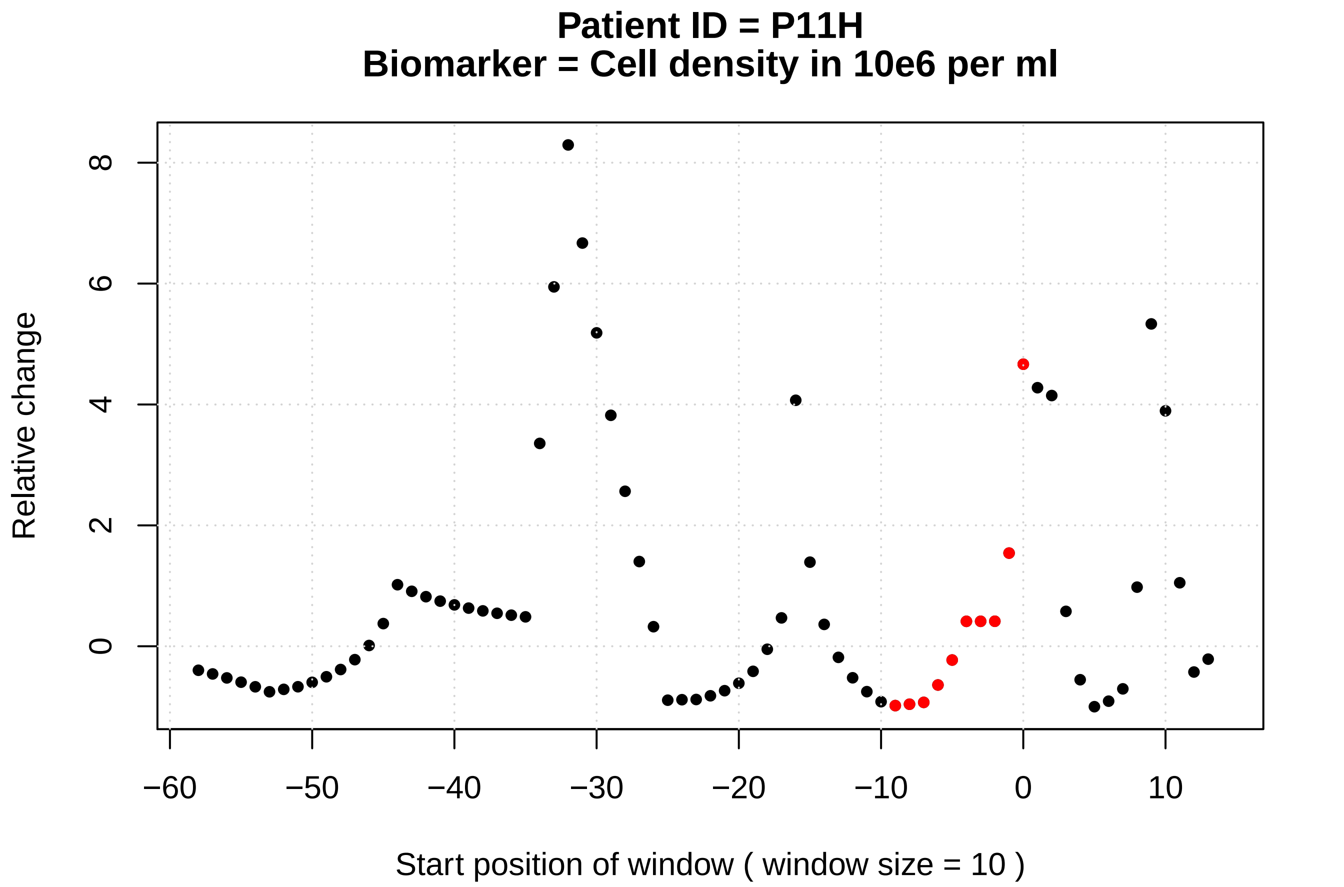
Patient ID = P11H
Biomarker = Cell density in 10e6 per ml
Relative change
0
2
4
6
8
−60
−50
−40
−30
−20
−10
0
10
Start position of window (window size = 10)

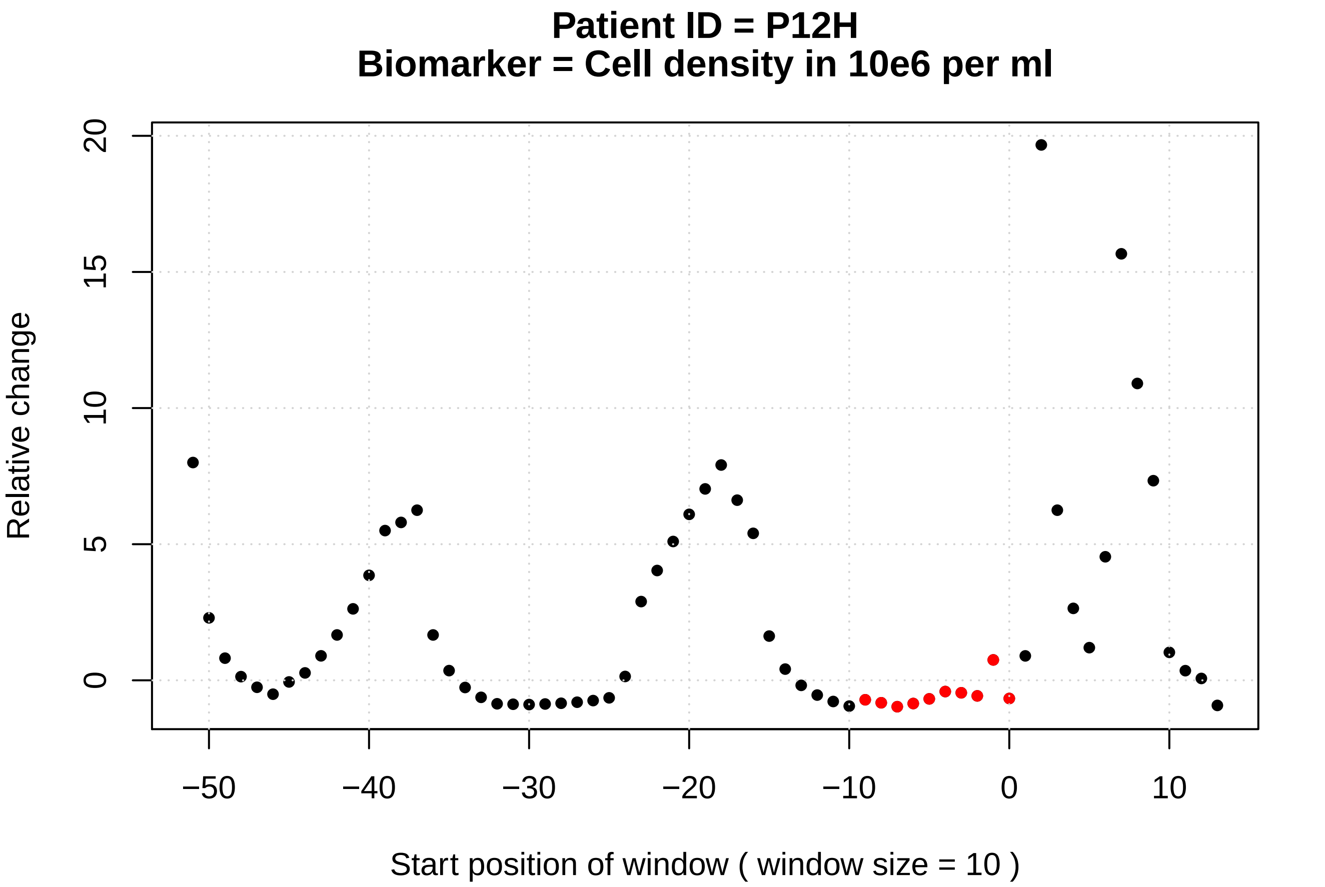
Patient ID = P12H
Biomarker = Cell density in 10e6 per ml
Relative change
0
5
10
15
20
−50
−40
−30
−20
−10
0
10
Start position of window (window size = 10)

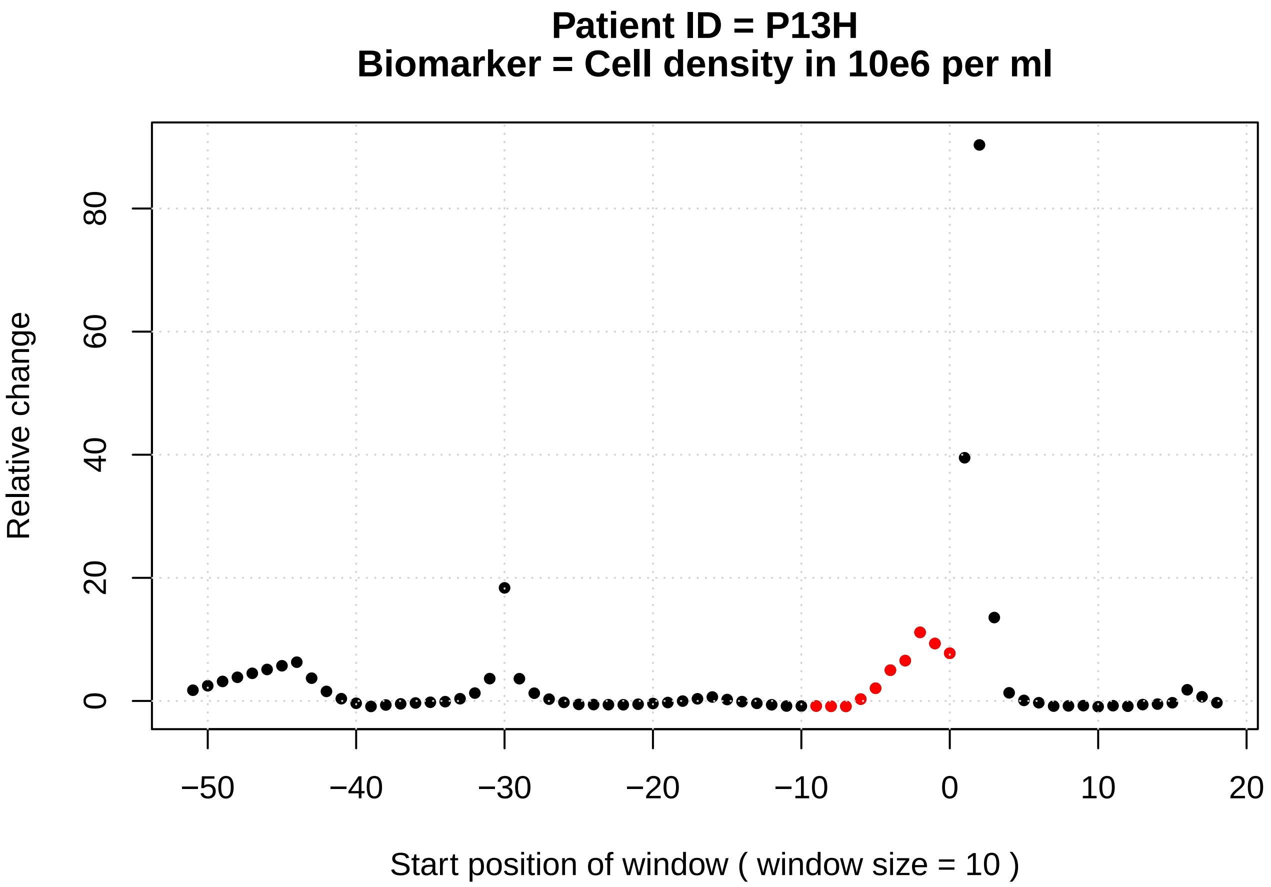
Patient ID = P13H
Biomarker = Cell density in 10e6 per ml
Relative change
0
20
40
60
80
−50
−40
−30
−20
−10
0
10
20
Start position of window (window size = 10)

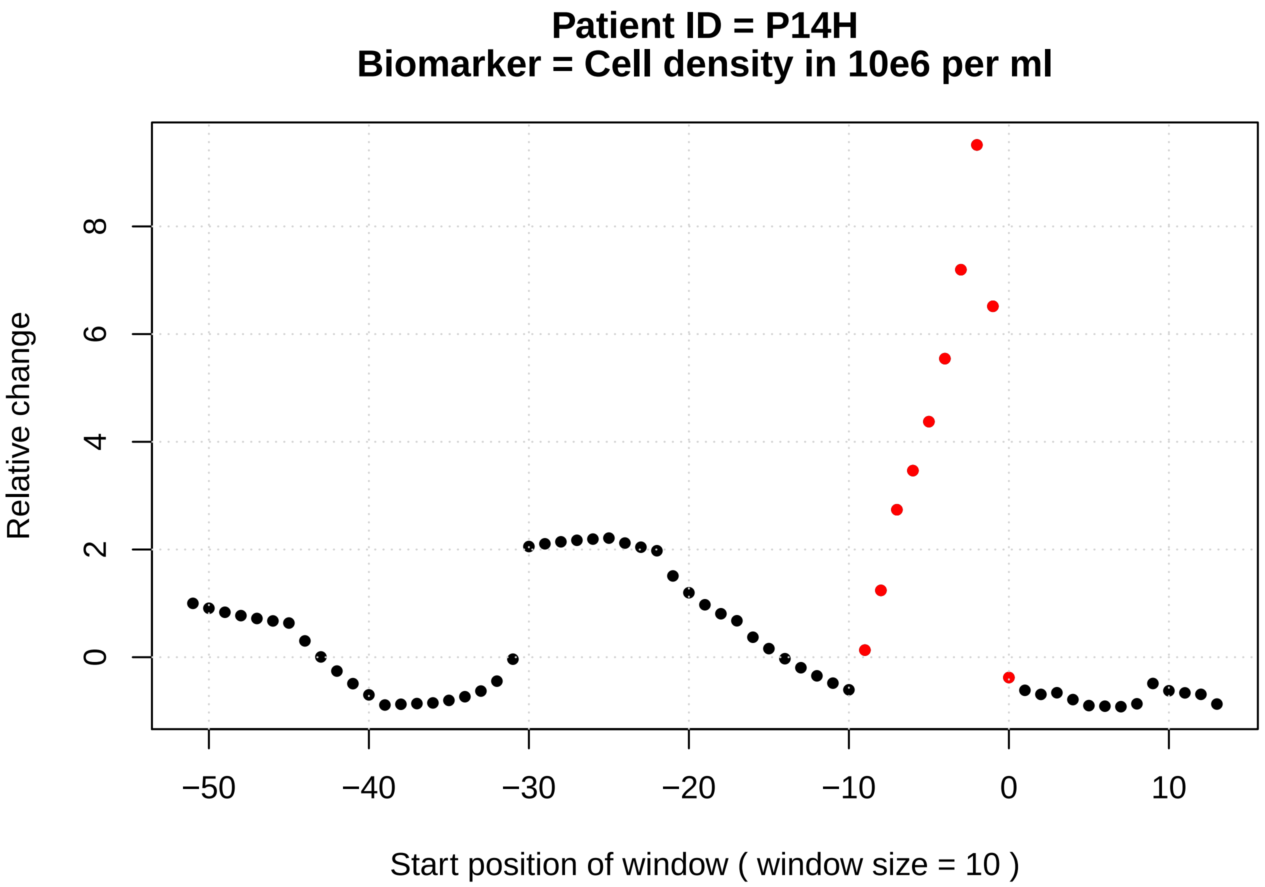
Patient ID = P14H
Biomarker = Cell density in 10e6 per ml
Relative change
0
2
4
6
8
−50
−40
−30
−20
−10
0
10
Start position of window (window size = 10)

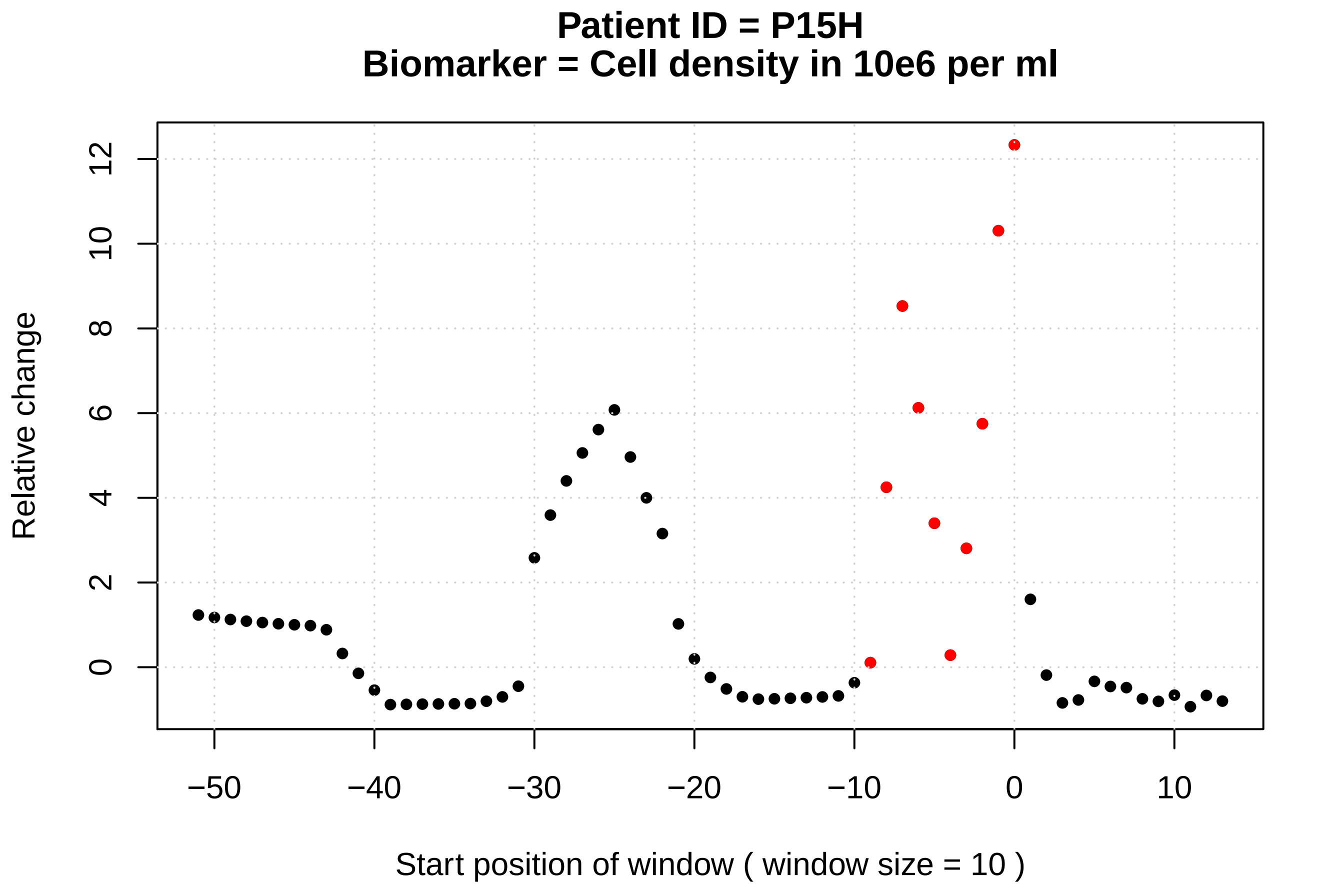
Patient ID = P15H
Biomarker = Cell density in 10e6 per ml
Relative change
0
2
4
6
8
10
12
−50
−40
−30
−20
−10
0
10
Start position of window (window size = 10)
