# Supplementary material for: Loss of adaptive capacity in asthmatic patients revealed by biomarker fluctuation dynamics after rhinovirus challenge
Source: eLife. 2019 Nov 5;8:e47969. doi: 10.7554/eLife.47969 (PMC6877087; doi:10.7554/eLife.47969)
Supplement: Supplementary file 1. [file elife-47969-supp1.zip › RelativeChangeWithinWindowPlots_PDFs/Appendix-figure SS24 IndividualWindowBoundariesRelativeChangeAnalysis NFEV1FVC Window Size = 10 AsthmaticParticipants.pdf]

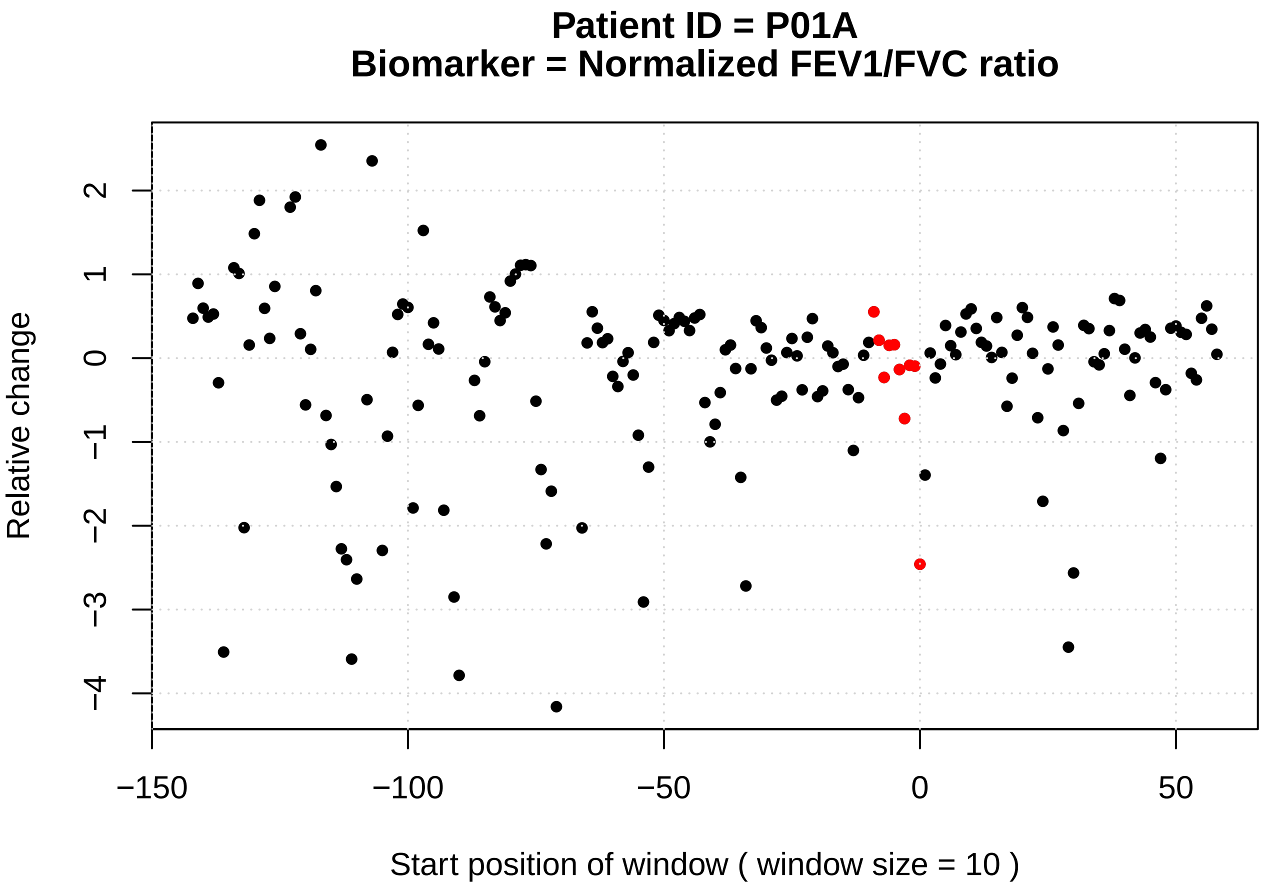
Patient ID = P01A
Biomarker = Normalized FEV1/FVC ratio
Relative change
Start position of window (window size = 10)
−150
−100
−50
0
50
2
1
0
−1
−2
−3
−4

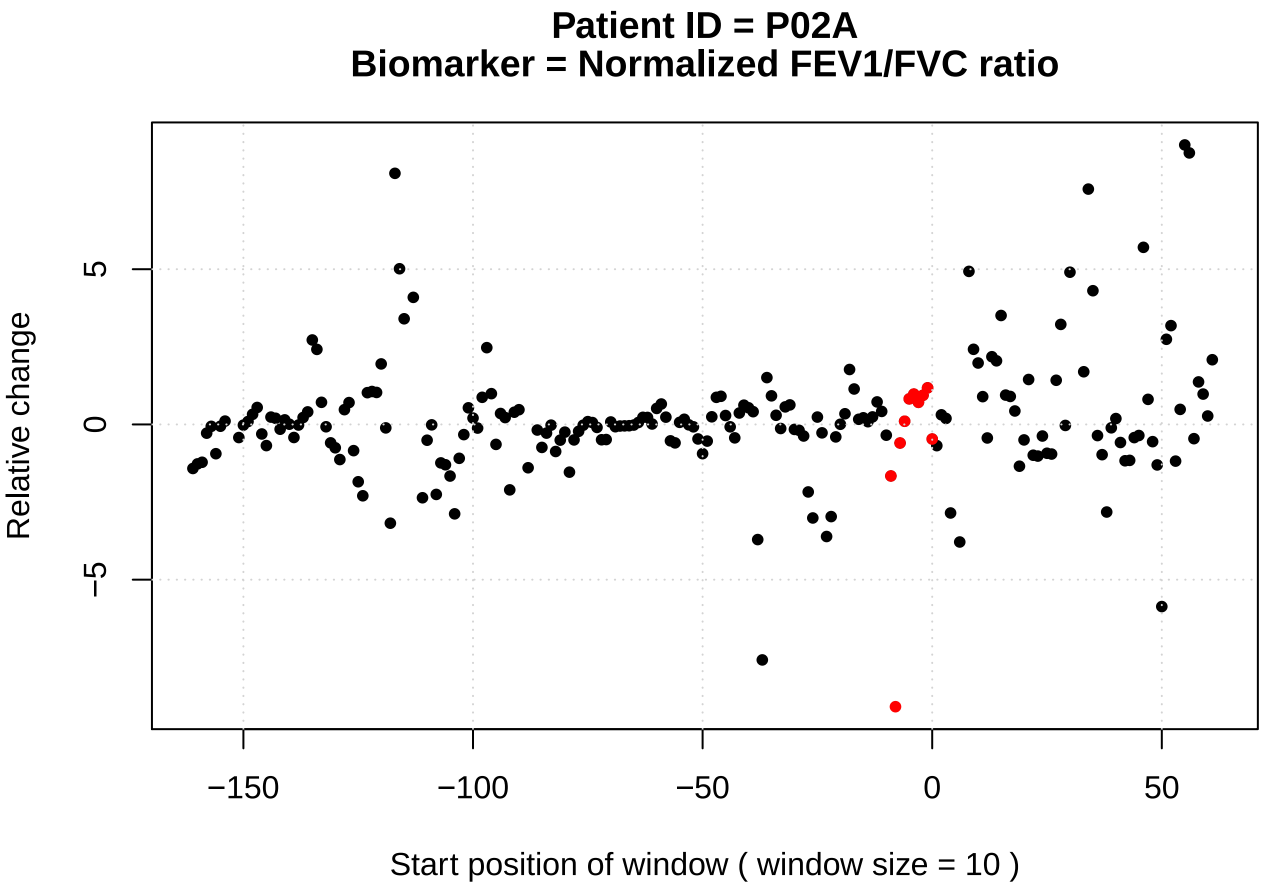
Patient ID = P02A
Biomarker = Normalized FEV1/FVC ratio
Relative change
5
0
−5
−150
−100
−50
0
50
Start position of window (window size = 10)

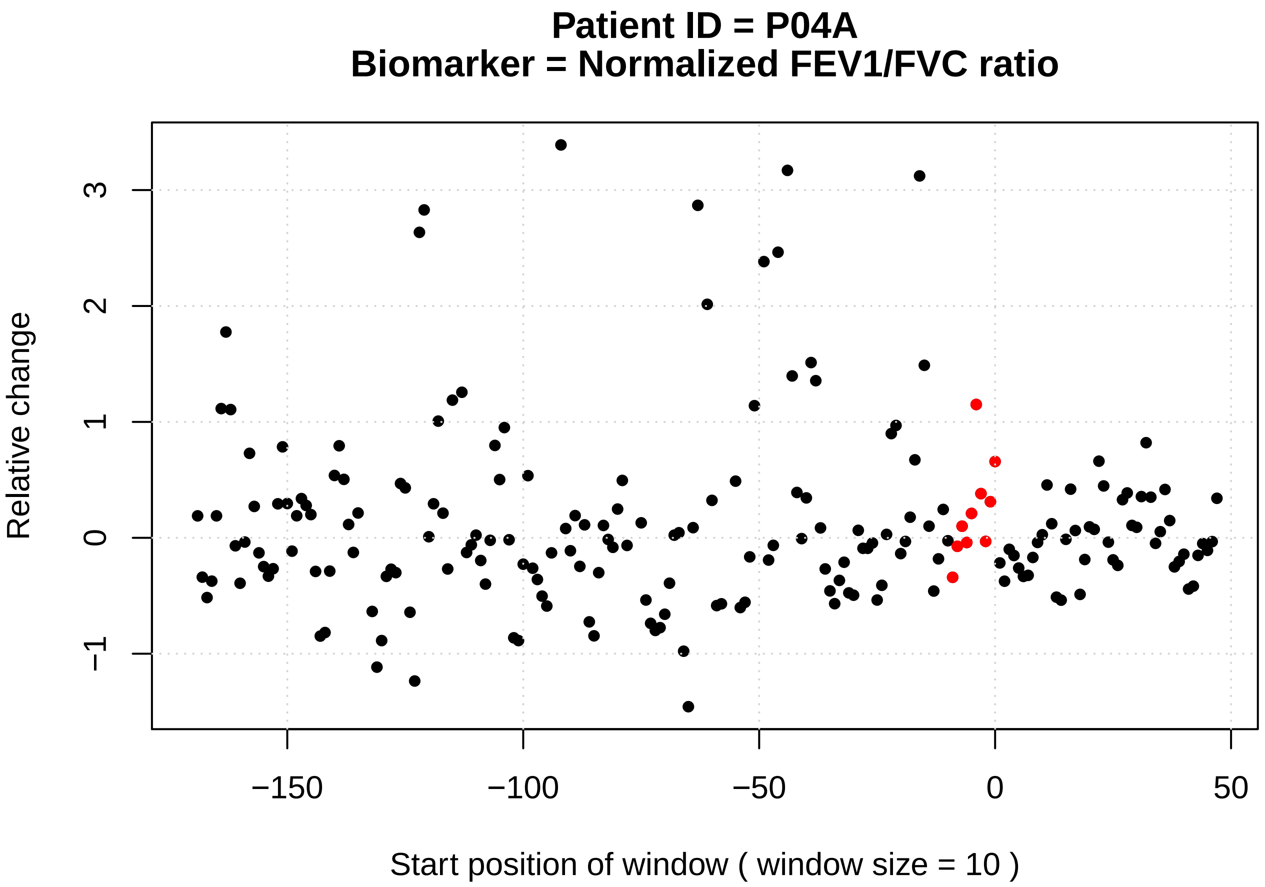

Patient ID = P04A
Biomarker = Normalized FEV1/FVC ratio
Relative change
−1
0
1
2
3
−150
−100
−50
0
50
Start position of window (window size = 10)

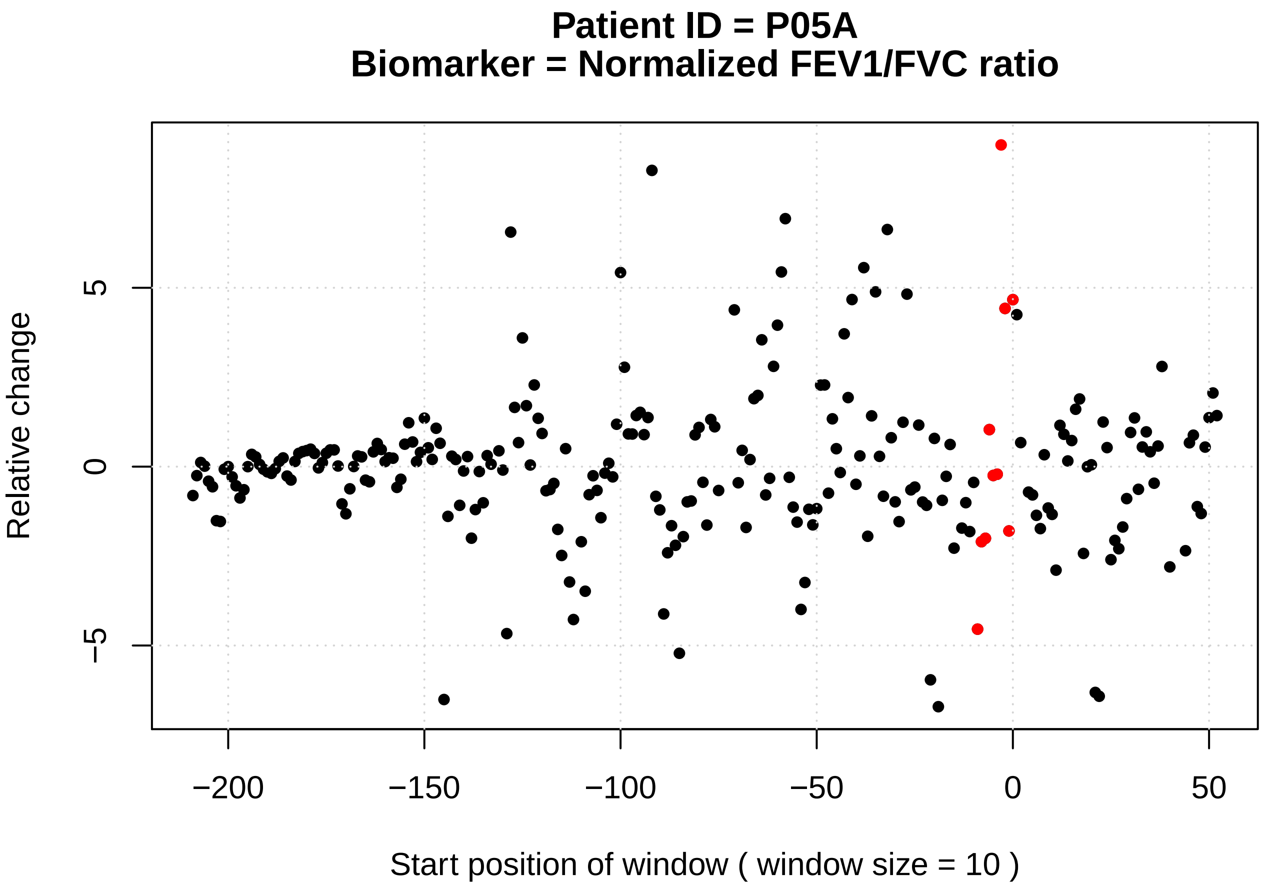
Patient ID = P05A
Biomarker = Normalized FEV1/FVC ratio
Relative change
5
0
−5
−200
−150
−100
−50
0
50
Start position of window (window size = 10)

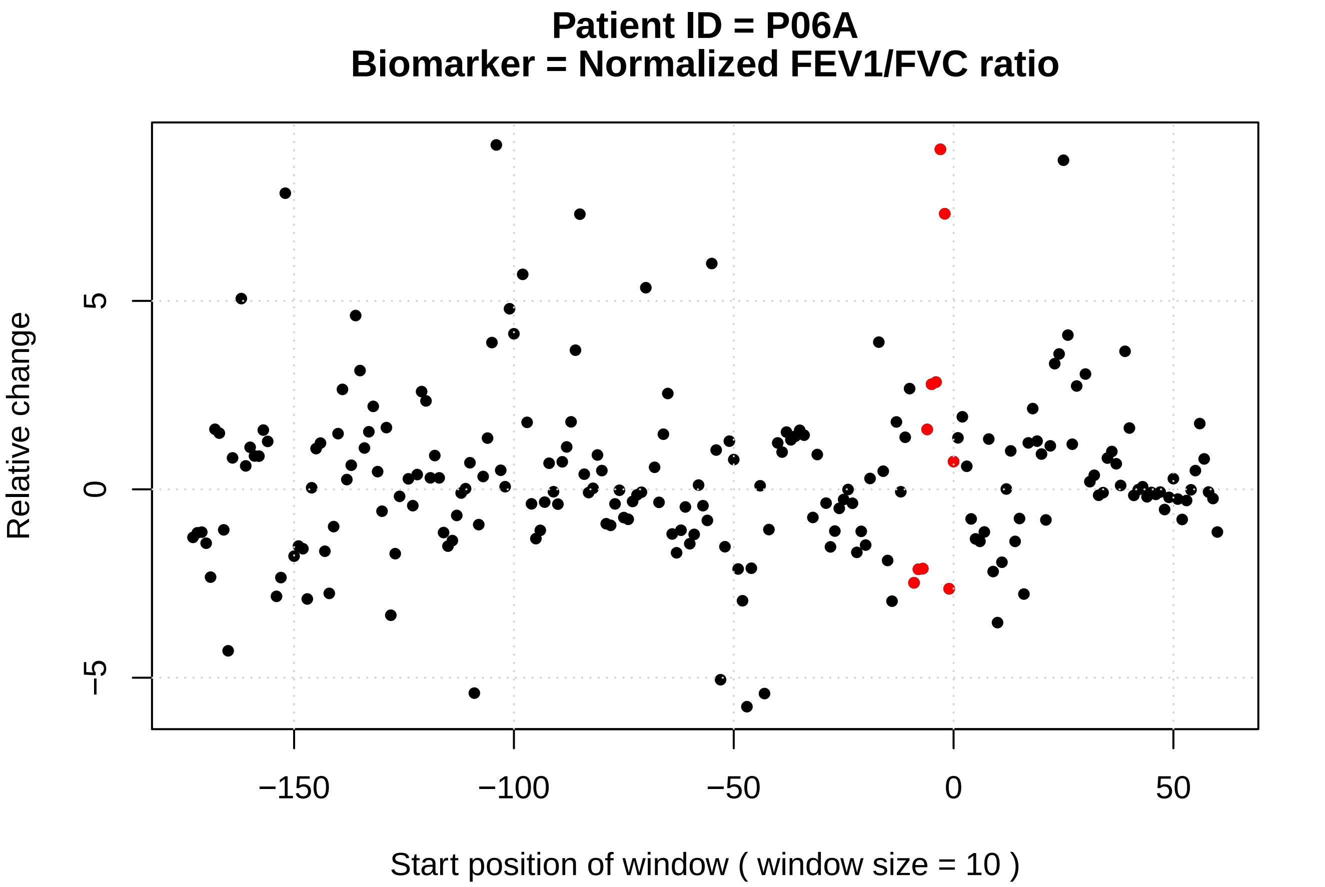
Patient ID = P06A
Biomarker = Normalized FEV1/FVC ratio
Relative change
5
0
−5
−150
−100
−50
0
50
Start position of window (window size = 10)

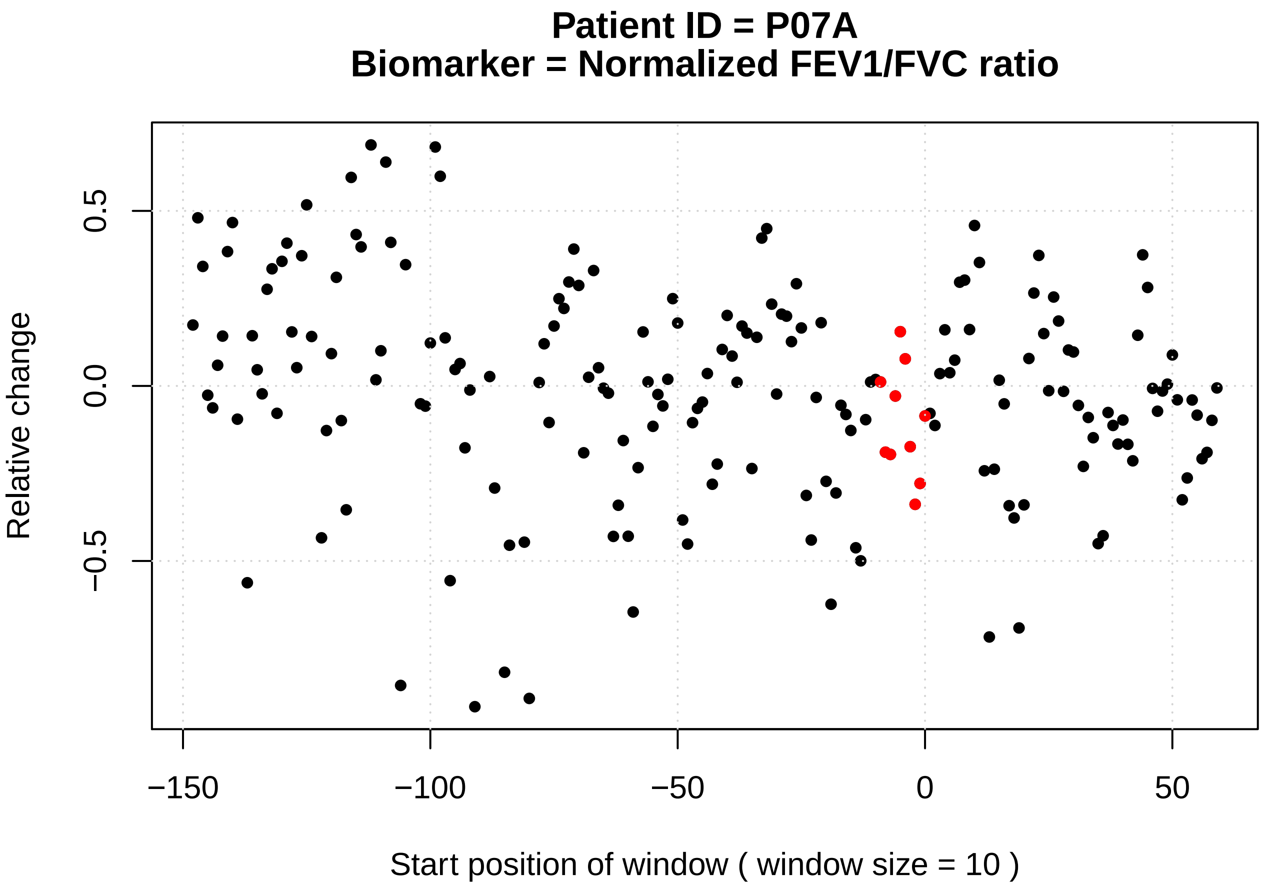
Patient ID = P07A
Biomarker = Normalized FEV1/FVC ratio
Relative change
0.5
0.0
−0.5
−150
−100
−50
0
50
Start position of window (window size = 10)

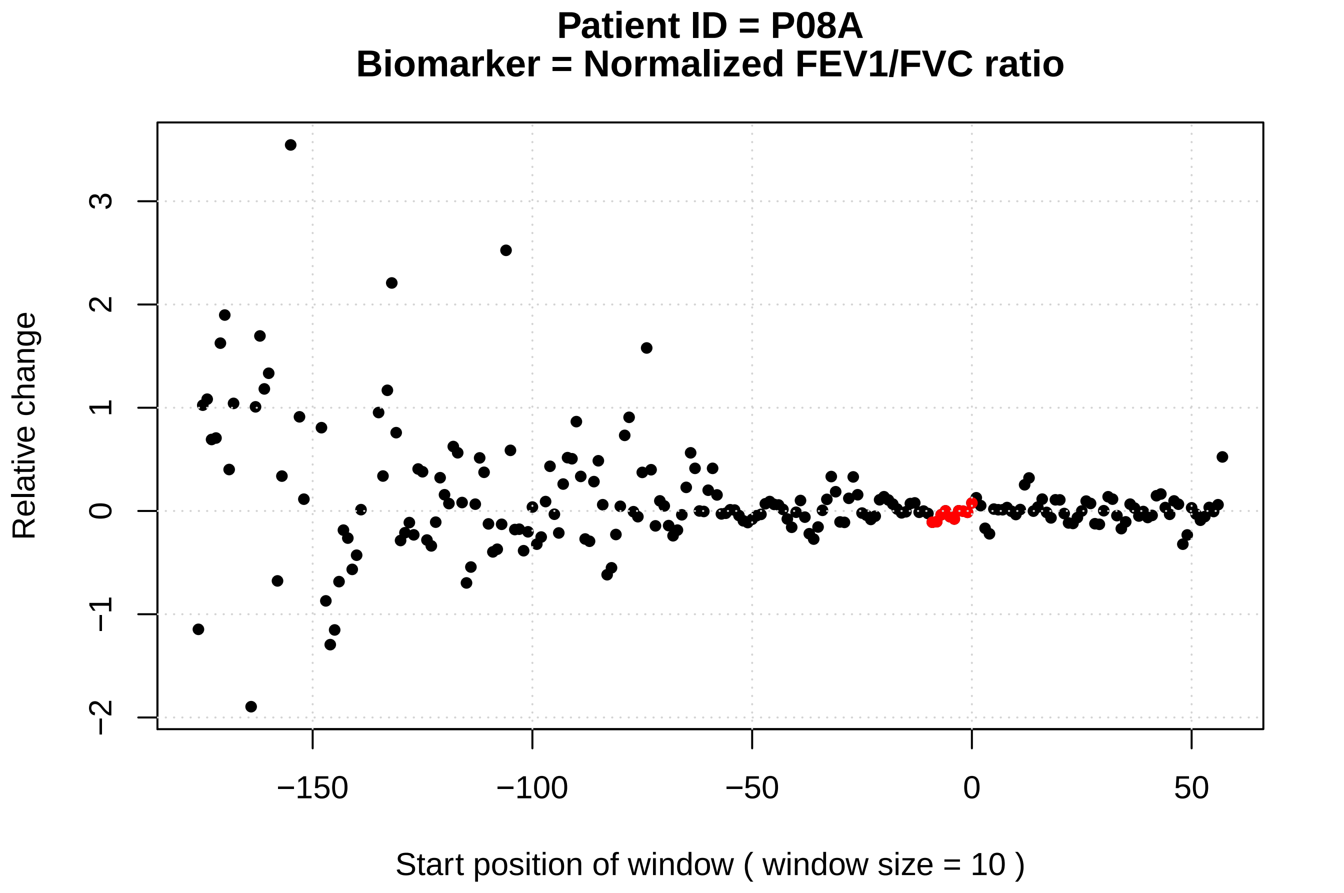

Patient ID = P08A
Biomarker = Normalized FEV1/FVC ratio
Relative change
3
2
1
0
−1
−2
−150
−100
−50
0
50
Start position of window (window size = 10)

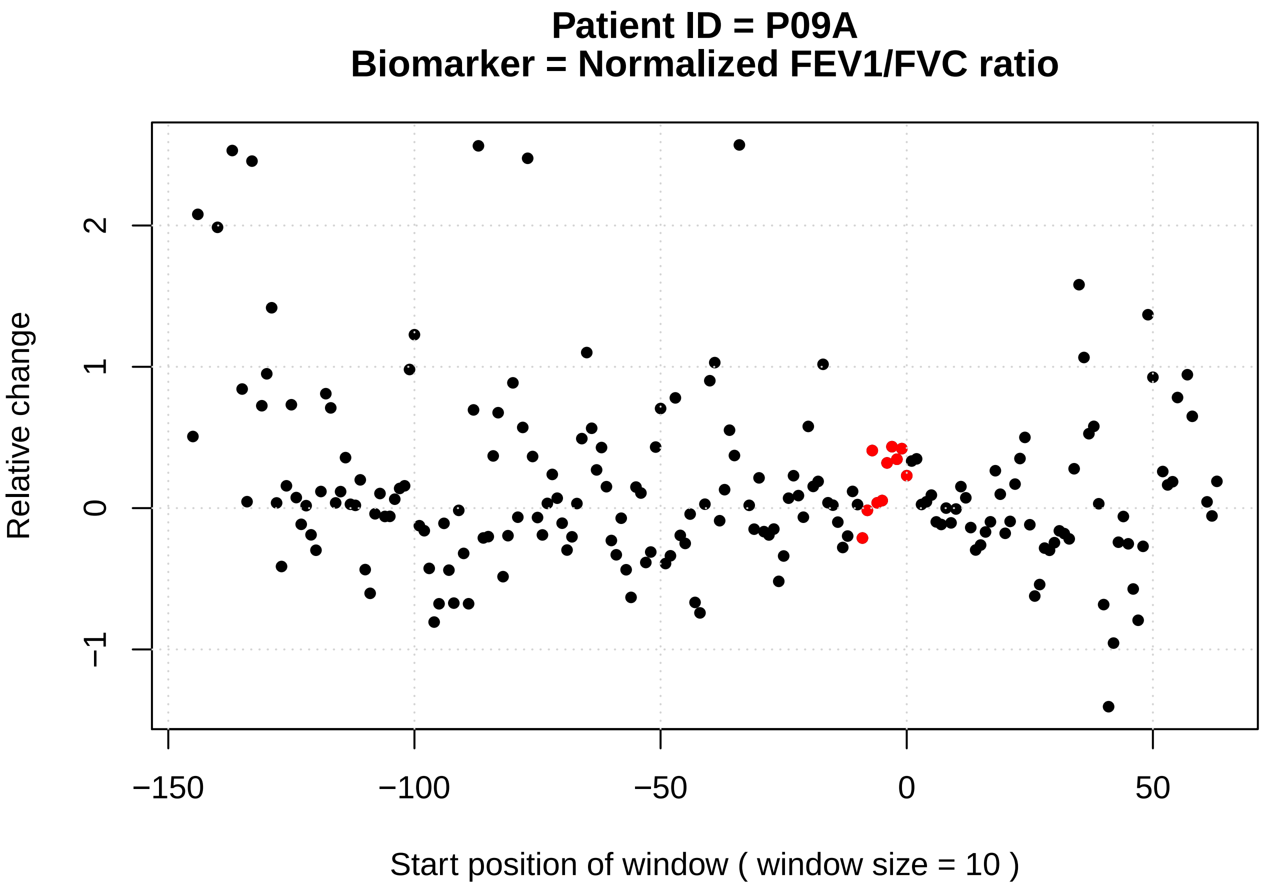

Patient ID = P09A
Biomarker = Normalized FEV1/FVC ratio
Relative change
−1
0
1
2
−150
−100
−50
0
50
Start position of window (window size = 10)

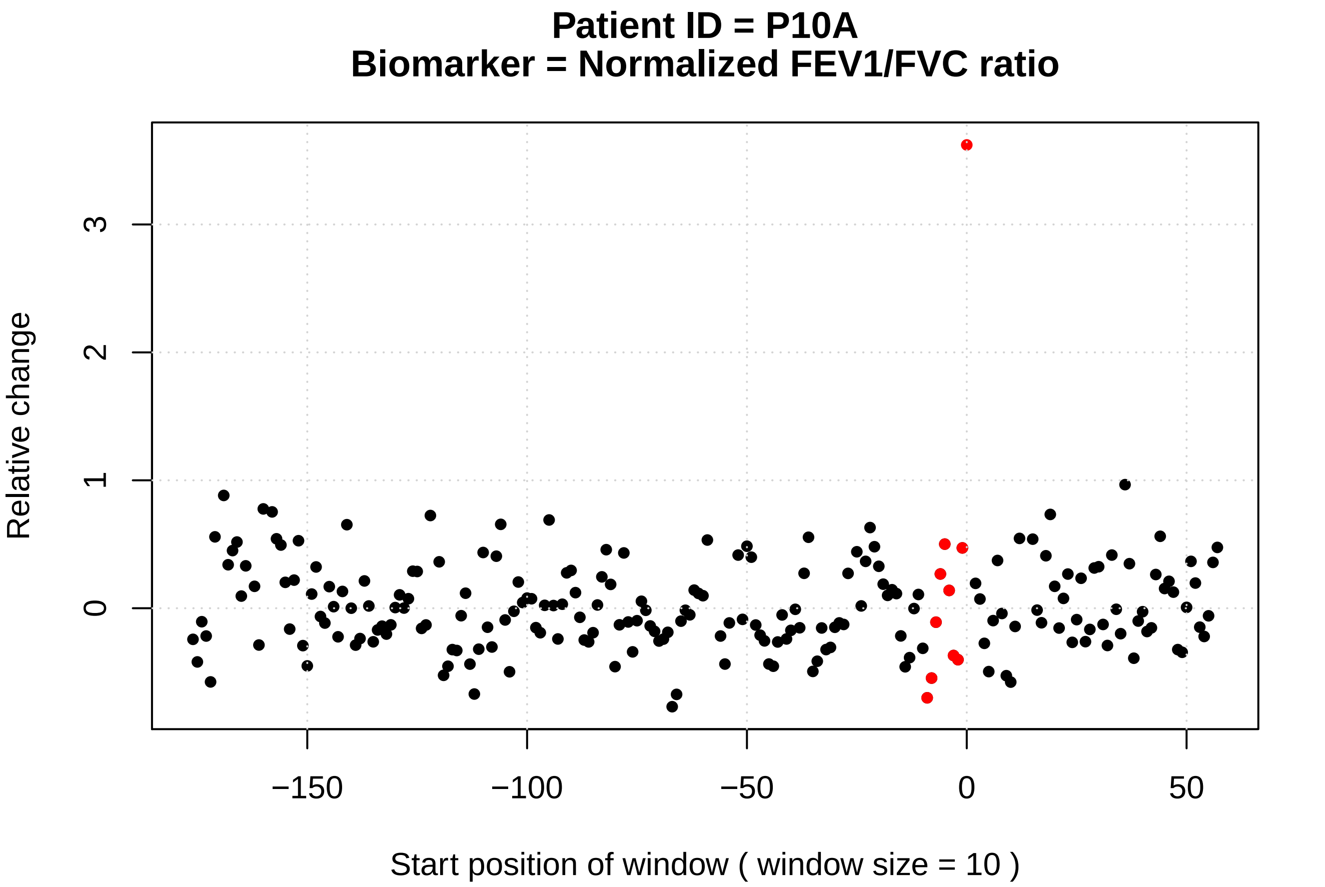
Patient ID = P10A
Biomarker = Normalized FEV1/FVC ratio
Relative change
0
1
2
3
−150
−100
−50
0
50
Start position of window (window size = 10)

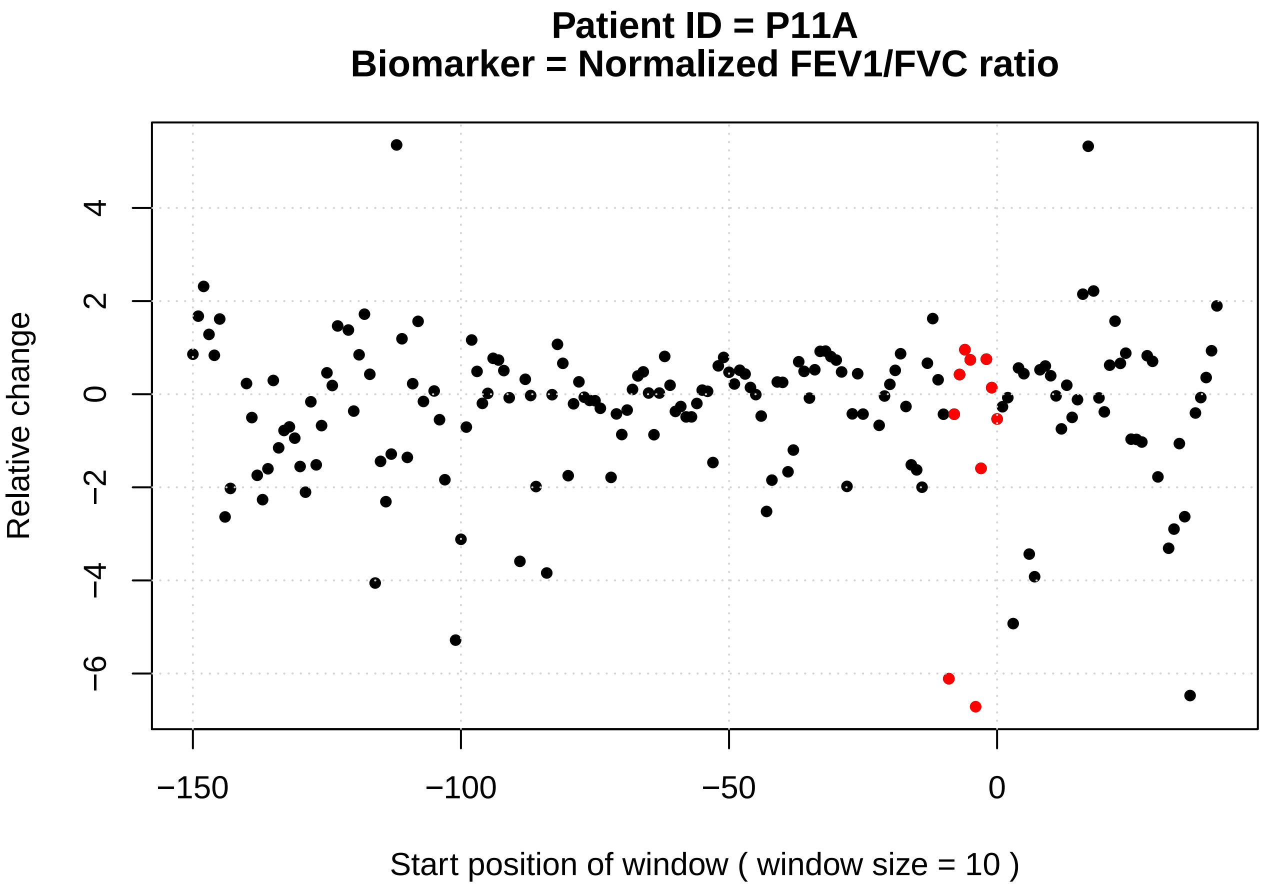
Patient ID = P11A
Biomarker = Normalized FEV1/FVC ratio
Relative change
4
2
0
−2
−4
−6
−150
−100
−50
0
Start position of window (window size = 10)

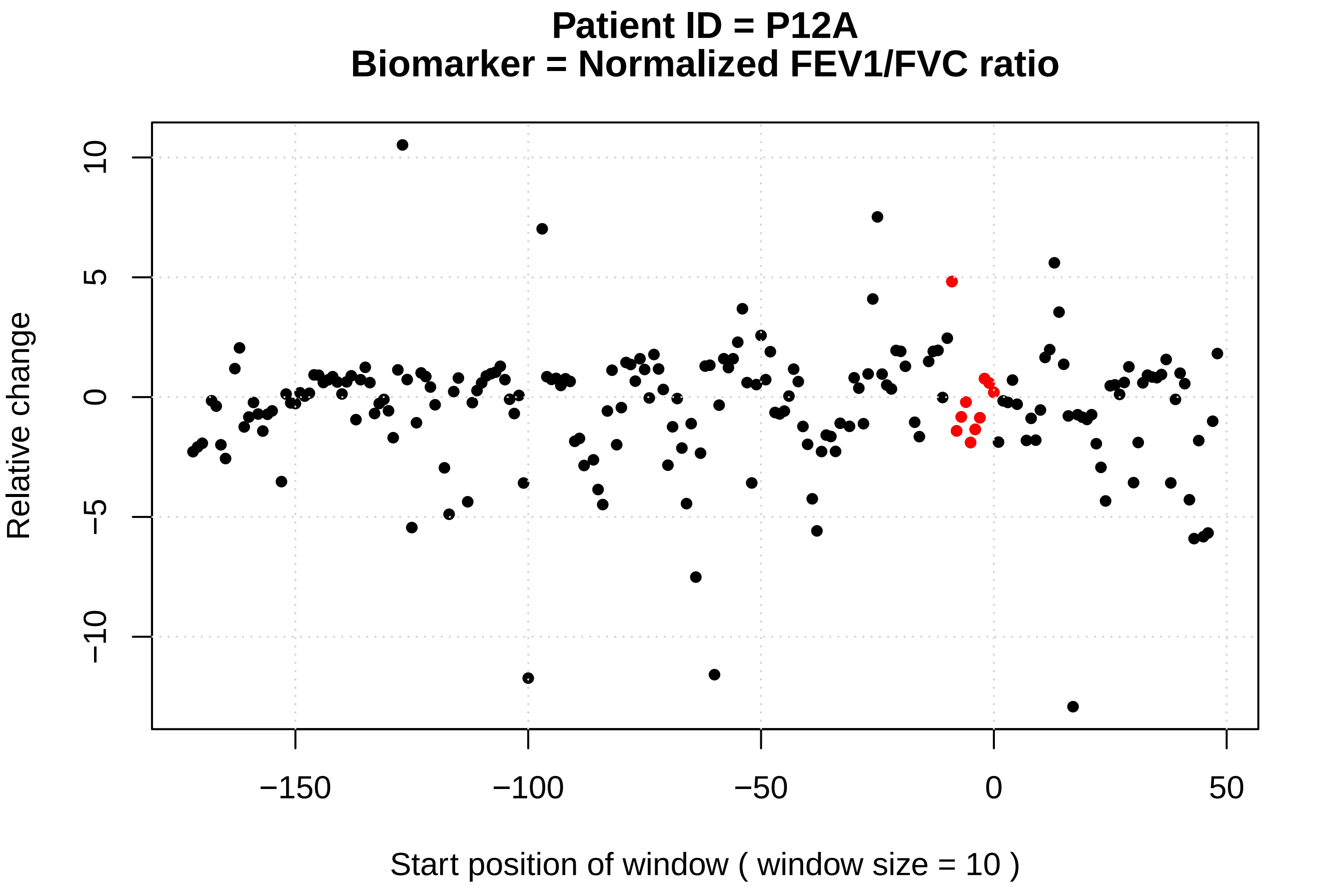
Patient ID = P12A
Biomarker = Normalized FEV1/FVC ratio
Relative change
10
5
0
−5
−10
−150
−100
−50
0
50
Start position of window (window size = 10)

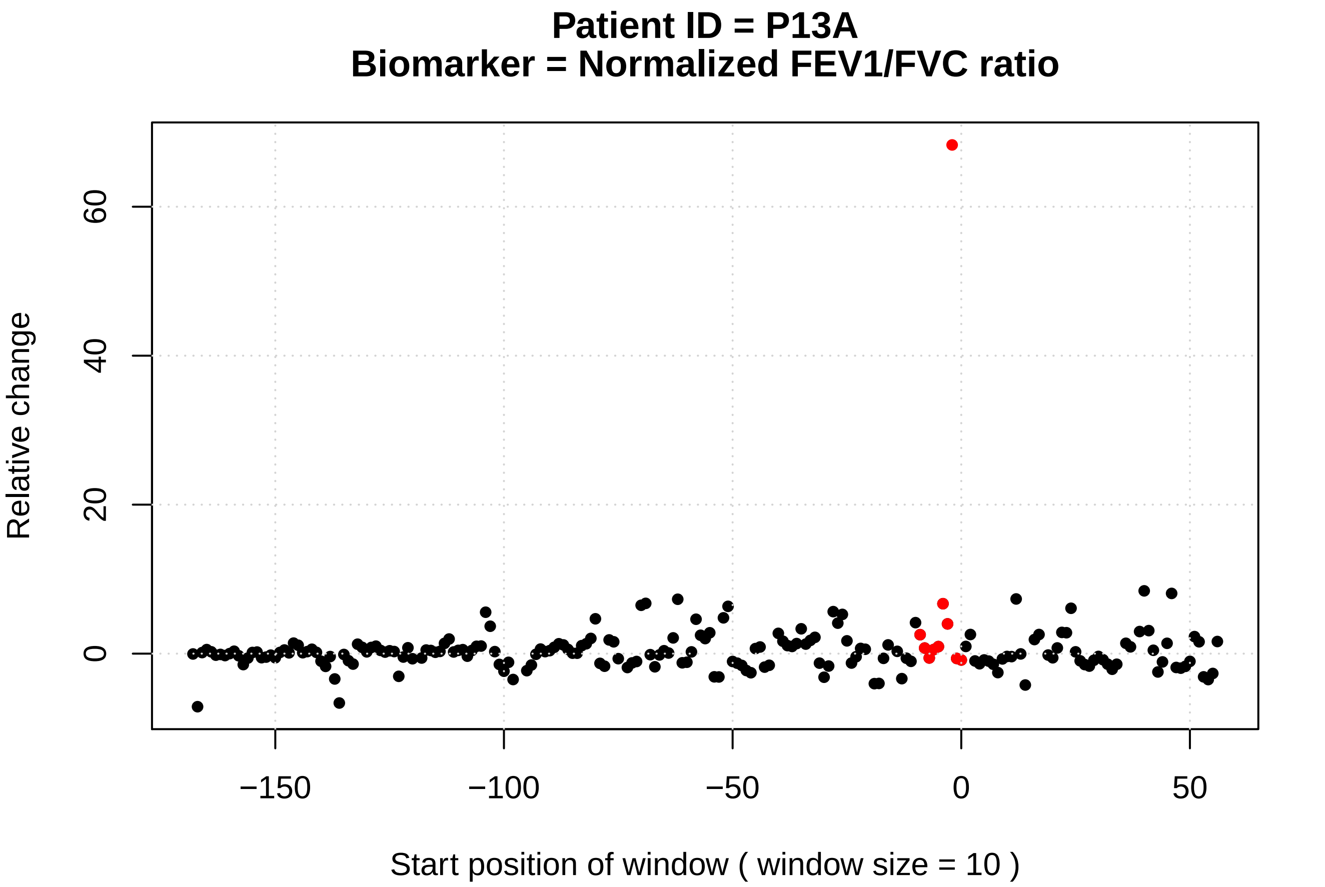
Patient ID = P13A
Biomarker = Normalized FEV1/FVC ratio
Relative change
0
20
40
60
−150
−100
−50
0
50
Start position of window (window size = 10)
