# Supplementary figures and images for: Loss of adaptive capacity in asthmatic patients revealed by biomarker fluctuation dynamics after rhinovirus challenge
Source: eLife. 2019 Nov 5;8:e47969. doi: 10.7554/eLife.47969 (PMC6877087; doi:10.7554/eLife.47969)

**P01H**

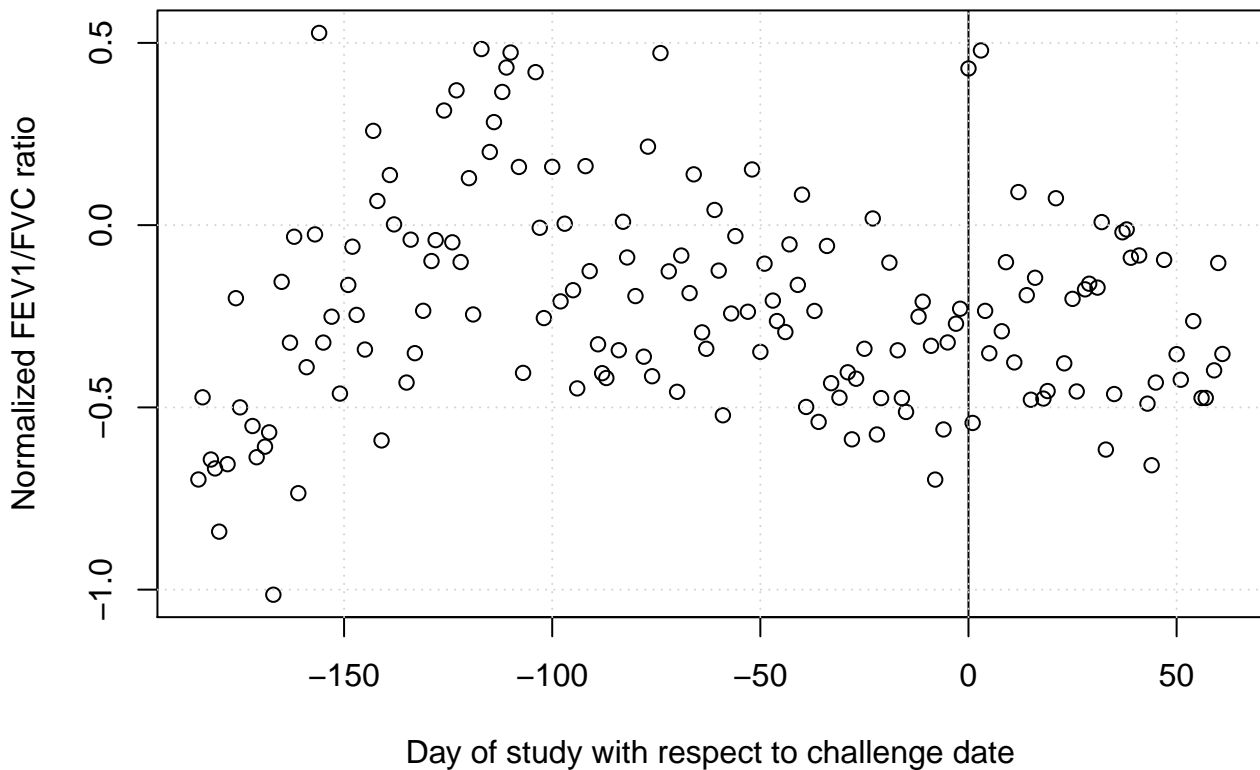

# P03H

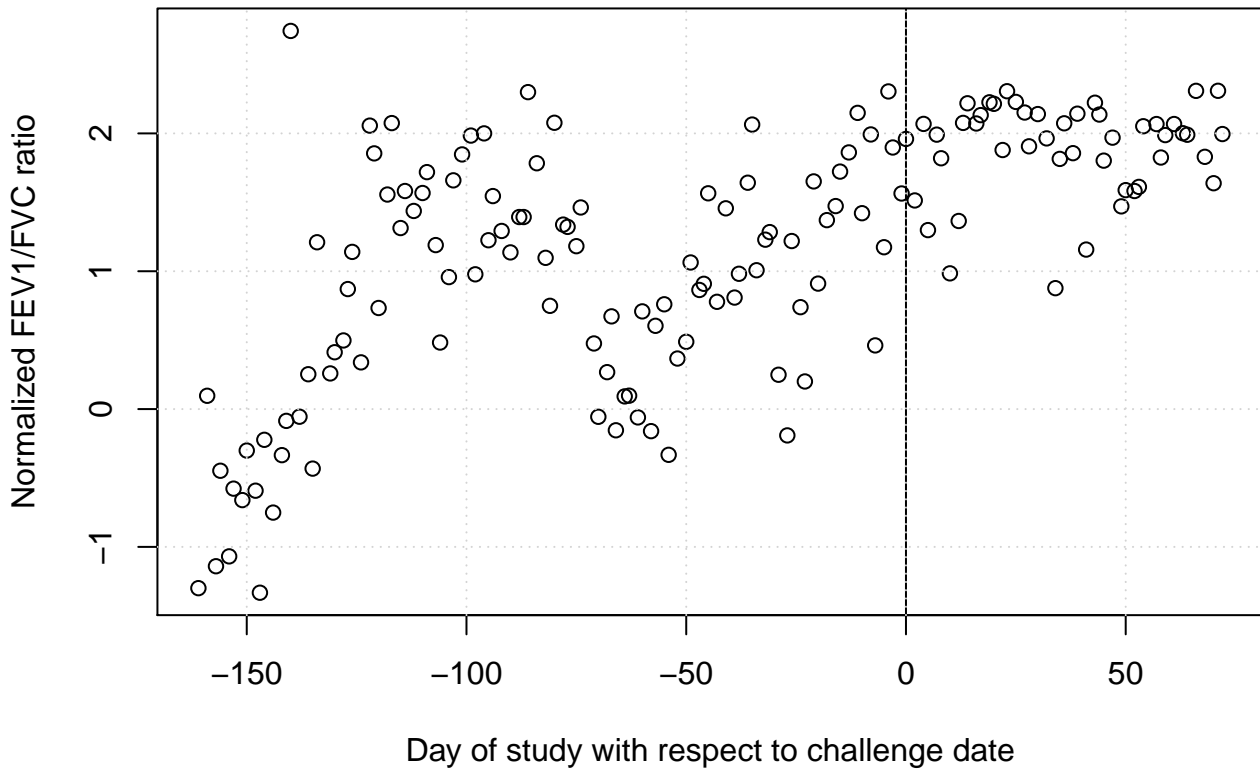

# P05H

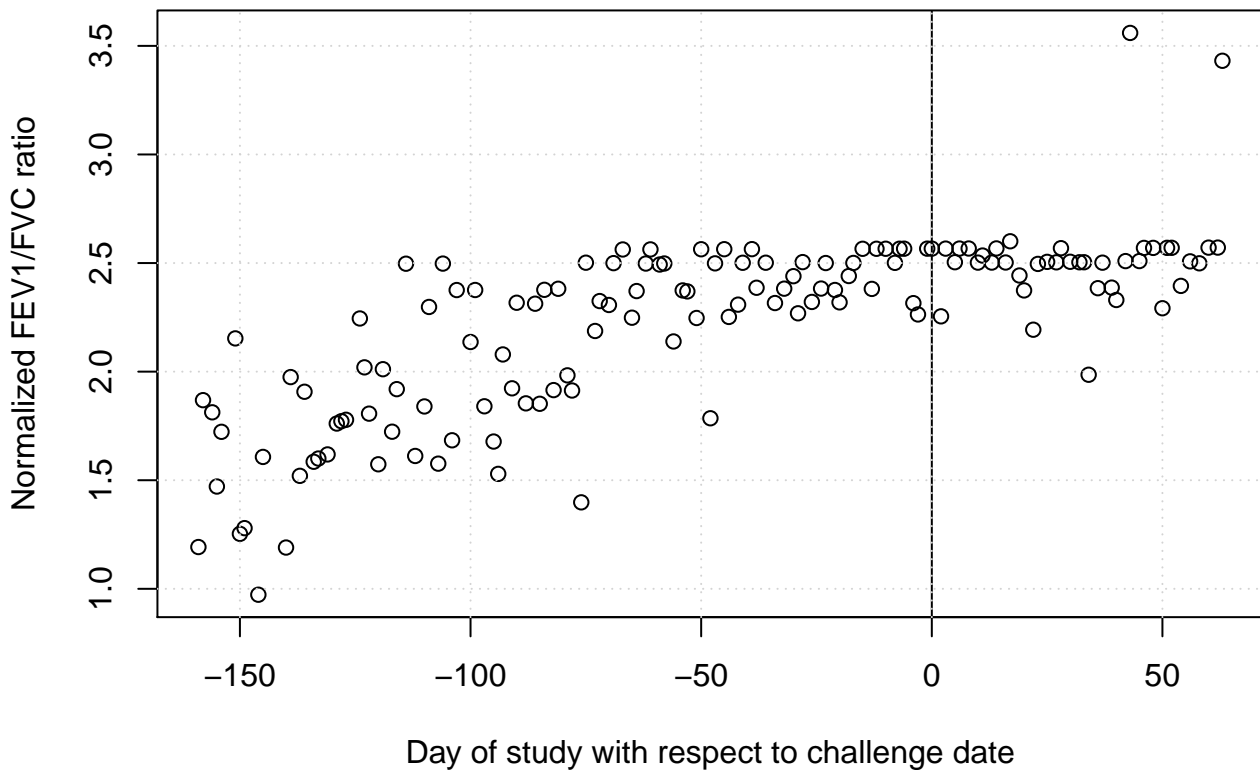

**P06H**

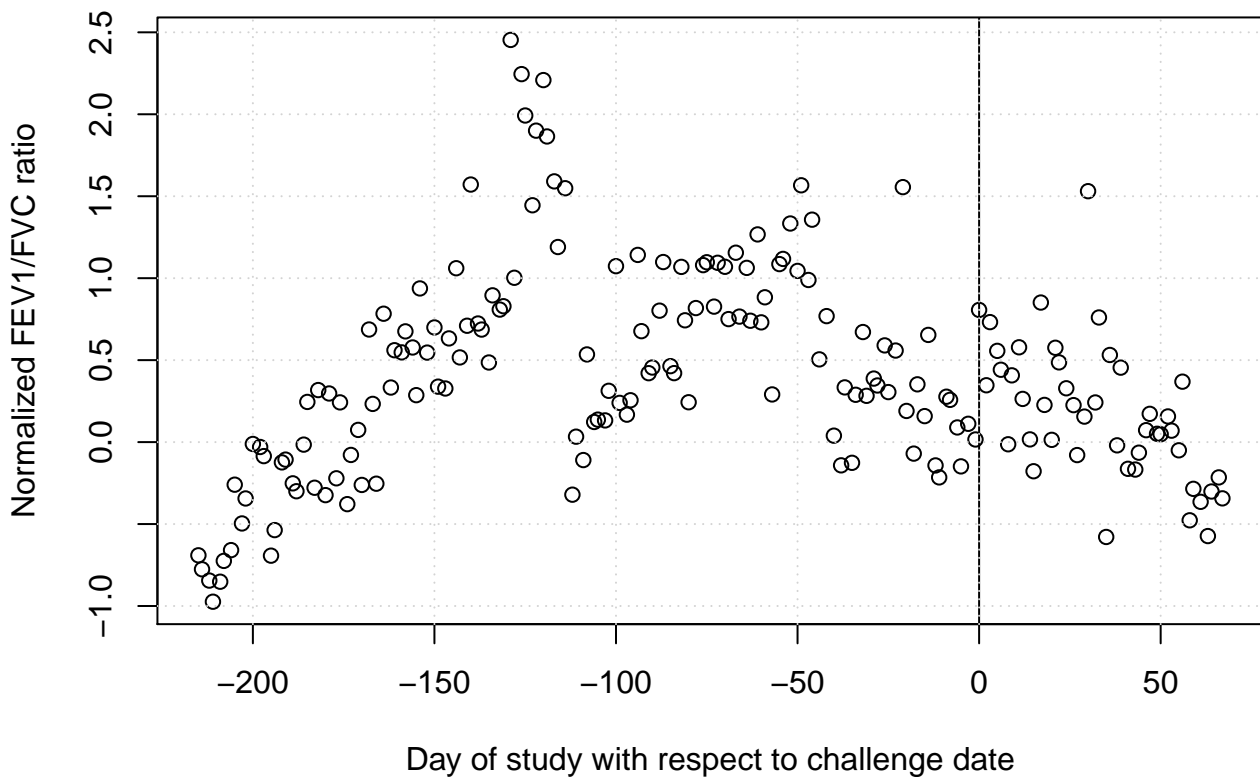

# P07H

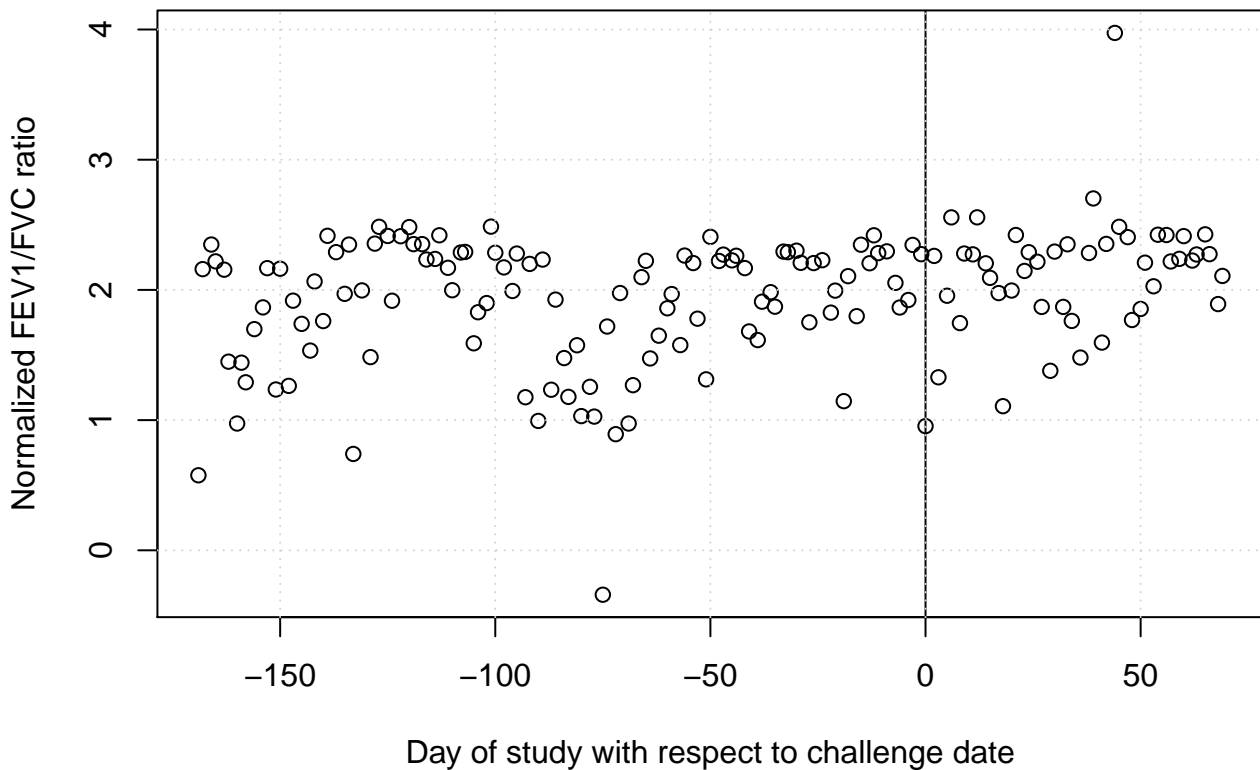

# P08H

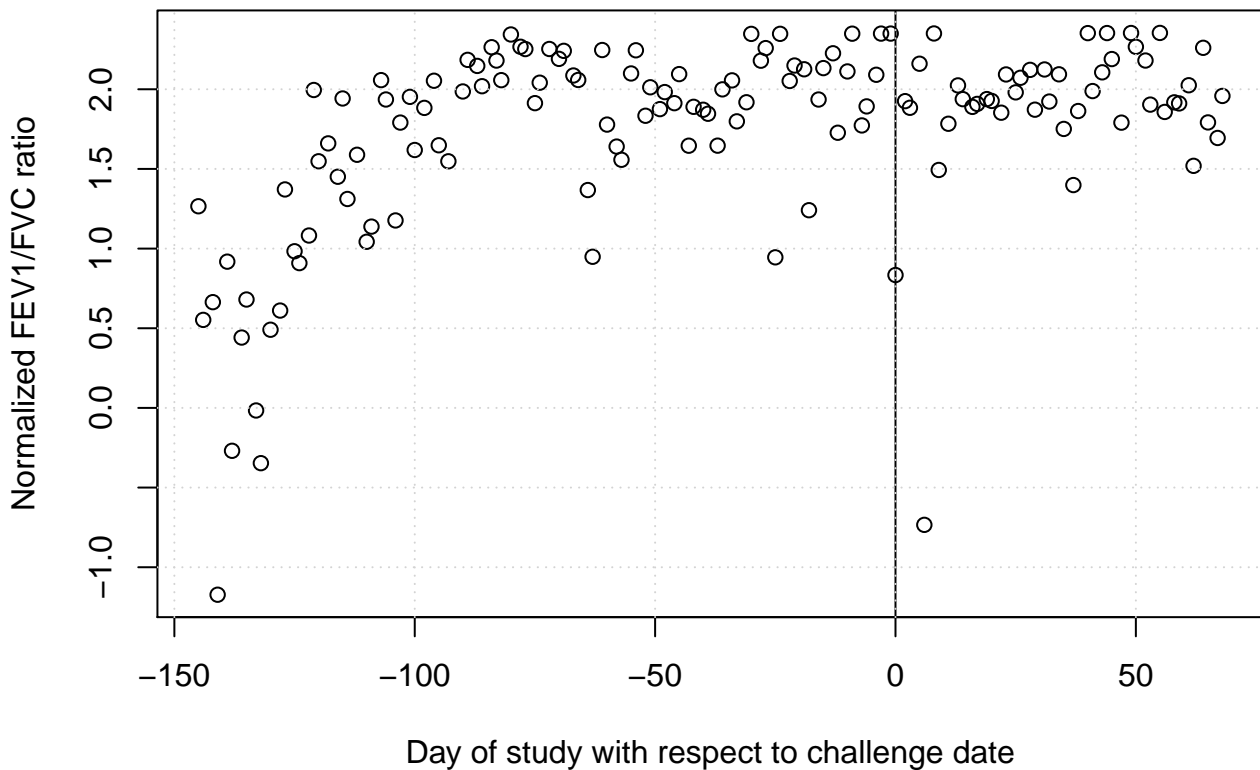

**P09H**

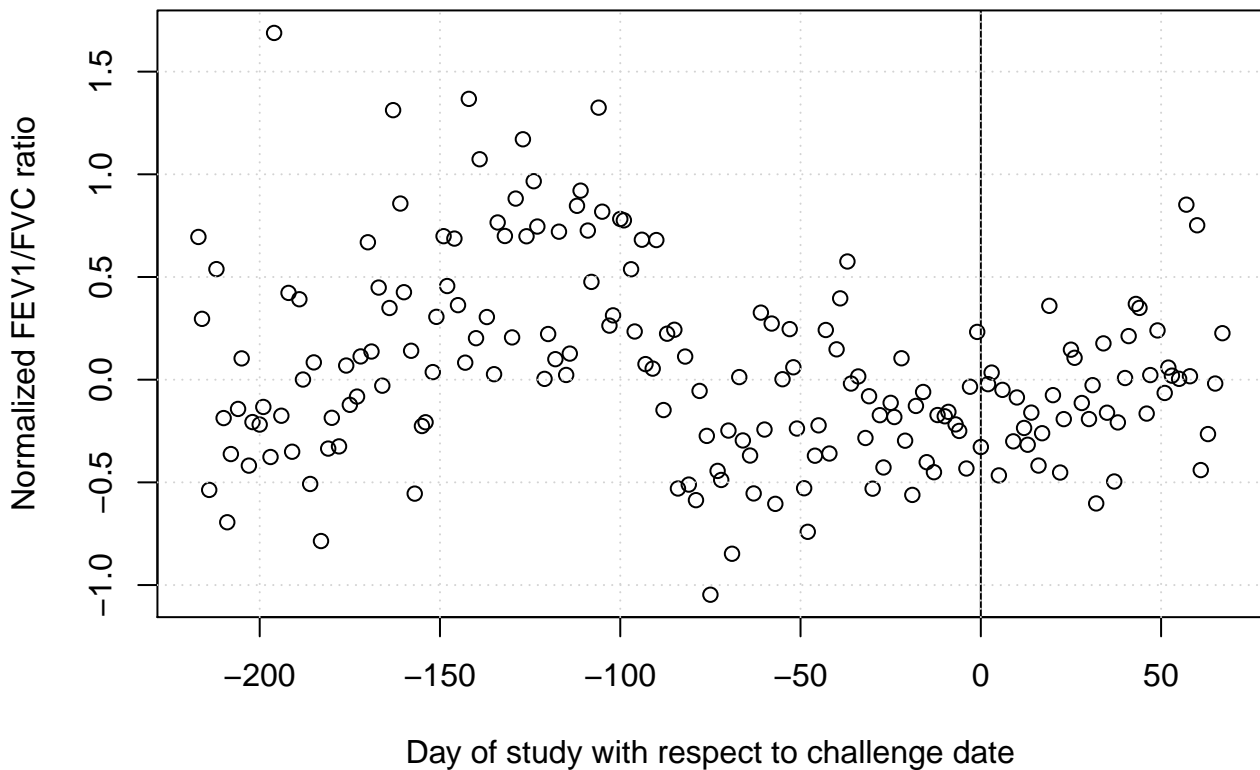

# P11H

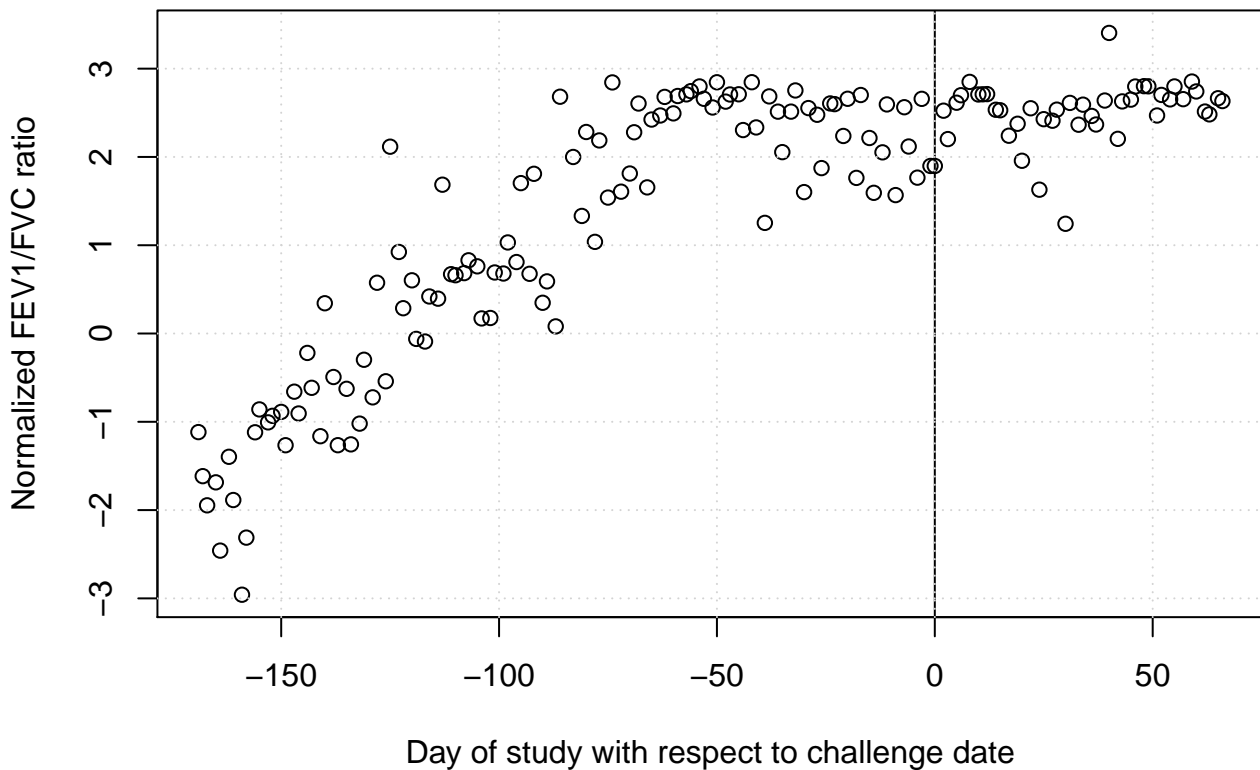

# P12H

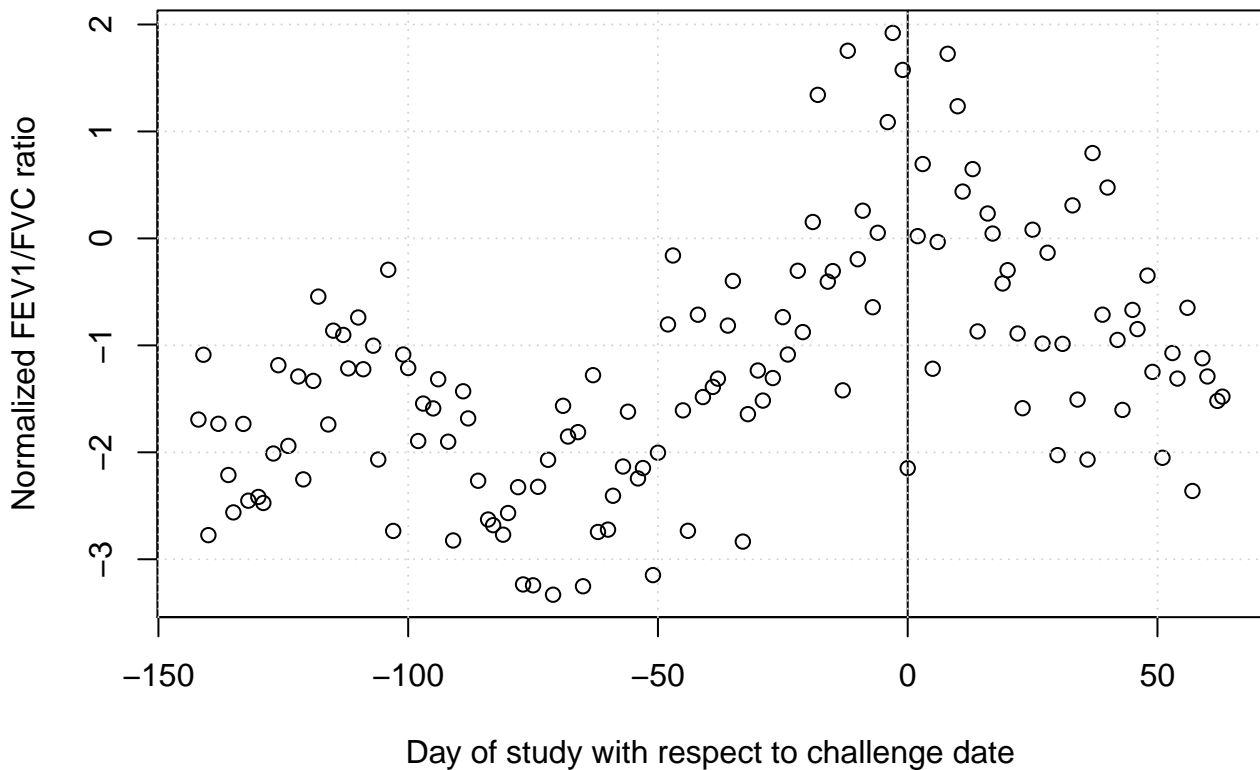

# P13H

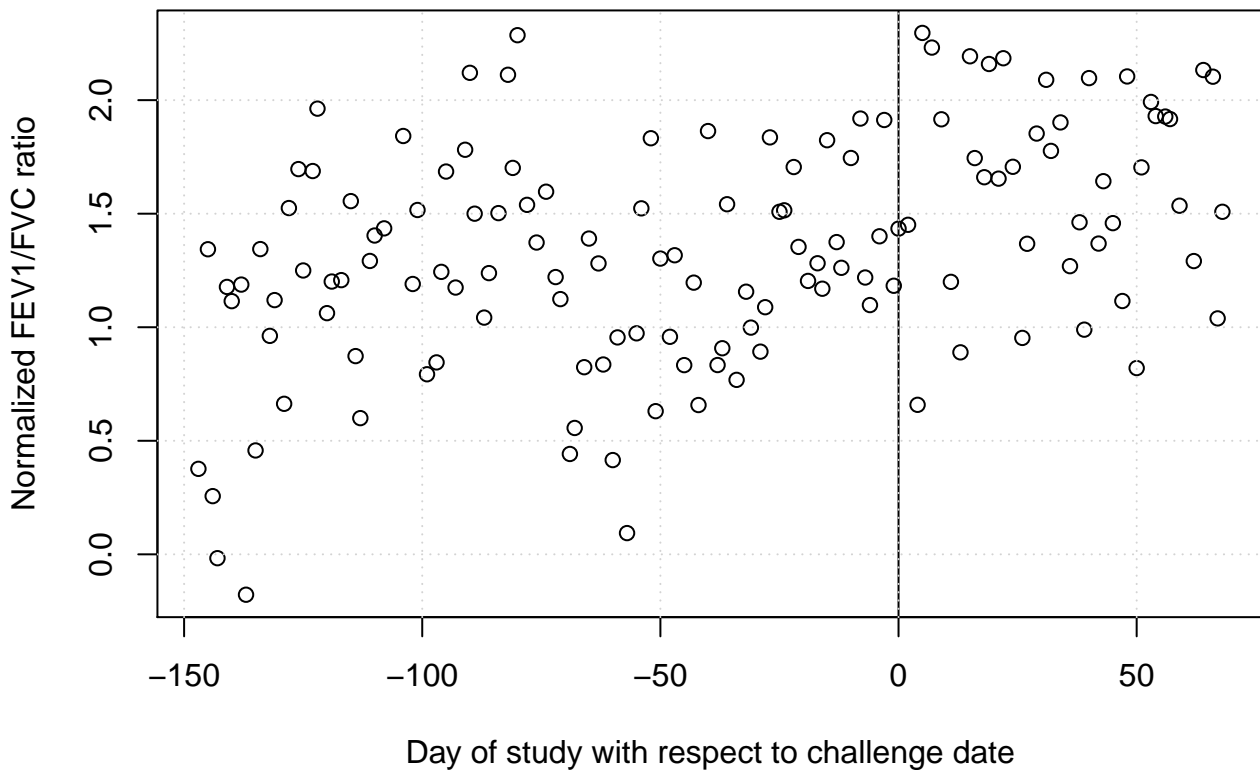

# P14H

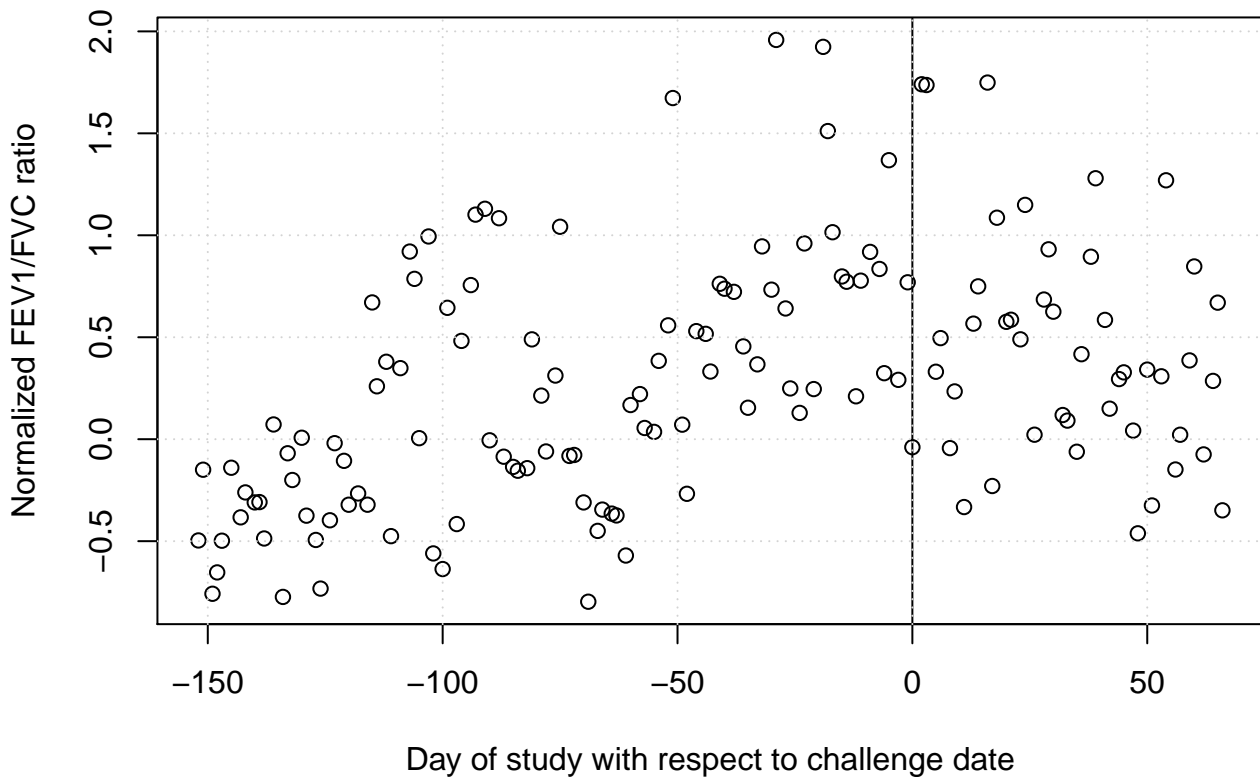

# P15H

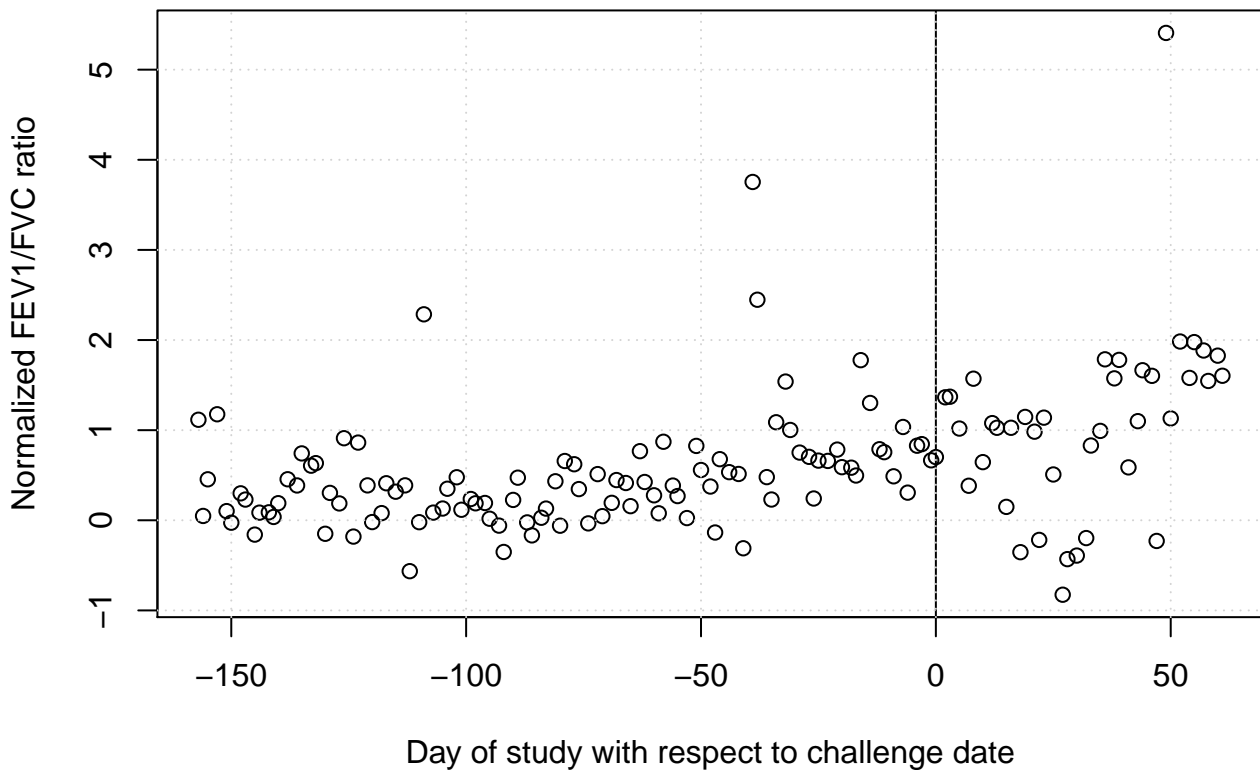

Supplement: Supplementary file 2. [file elife-47969-supp2.zip › TimeSeriesPlots_PDFs/Appendix-figure SS7_NFEV1FVC_TimeSeries_HealthyParticipants.pdf]

# P01A

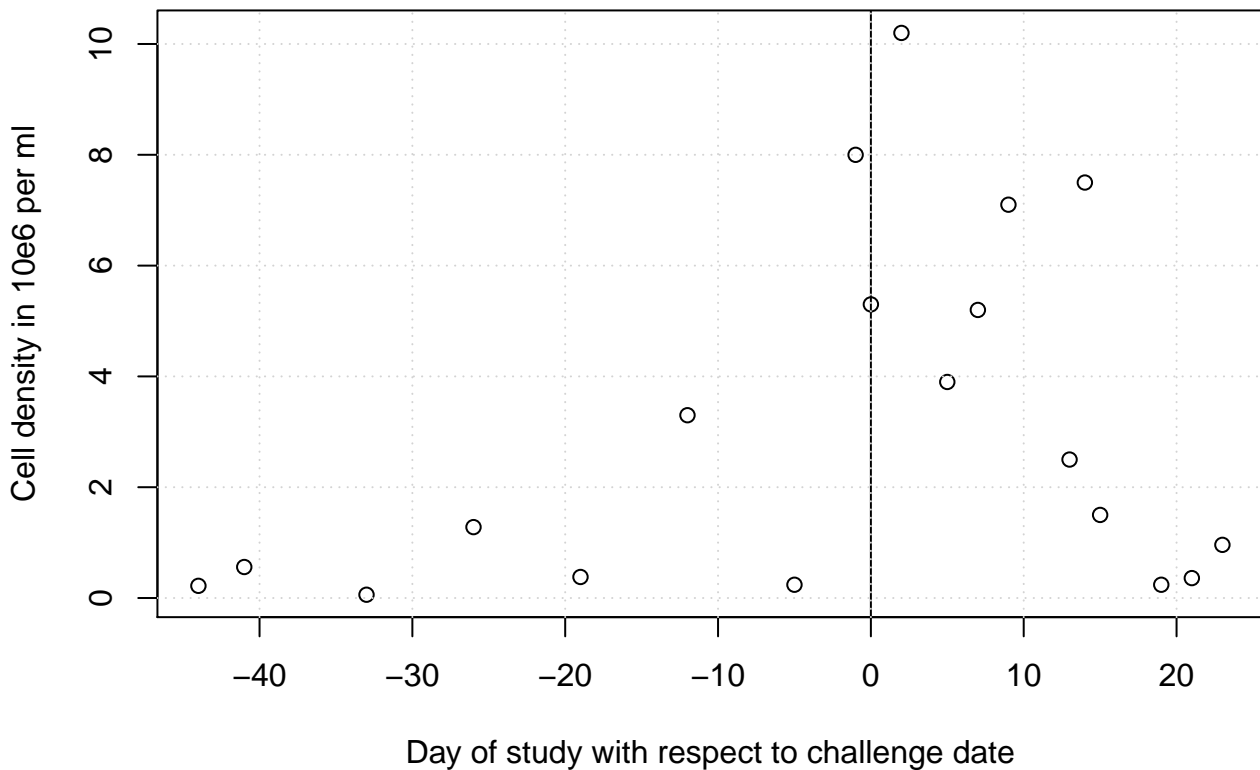

## P02A

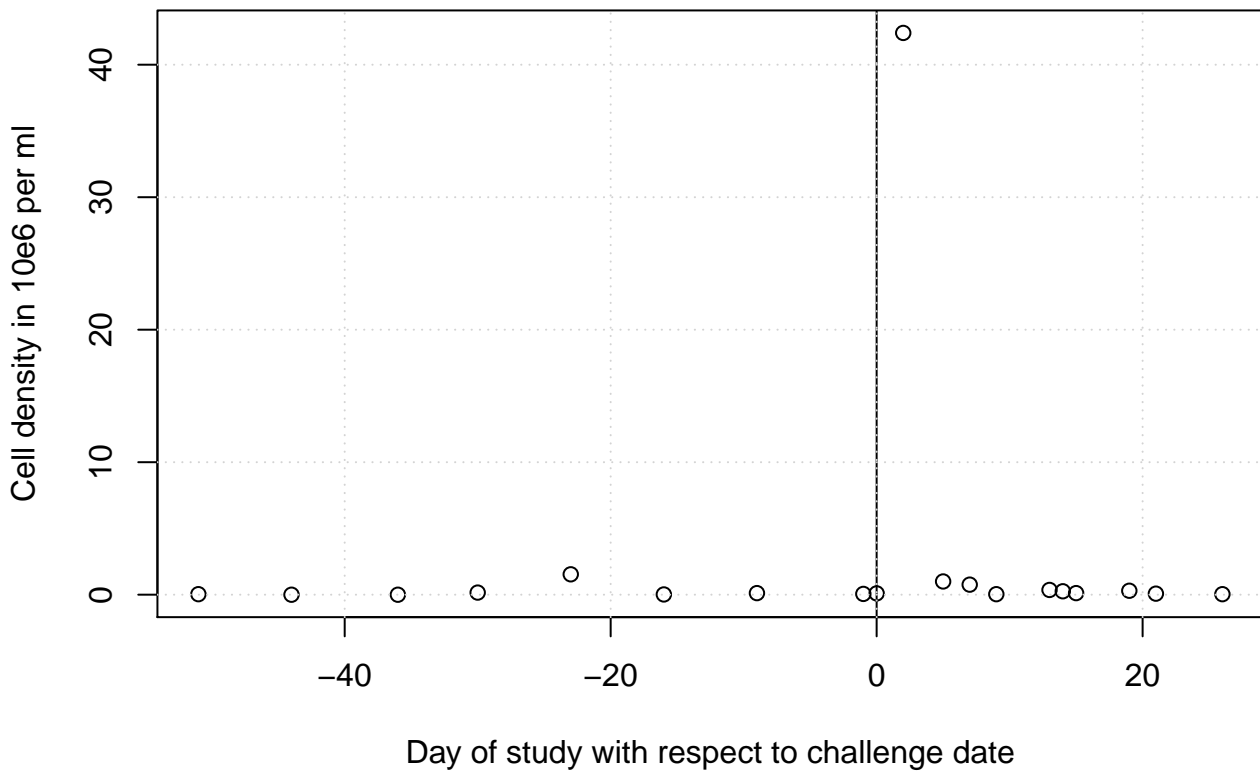

# P04A

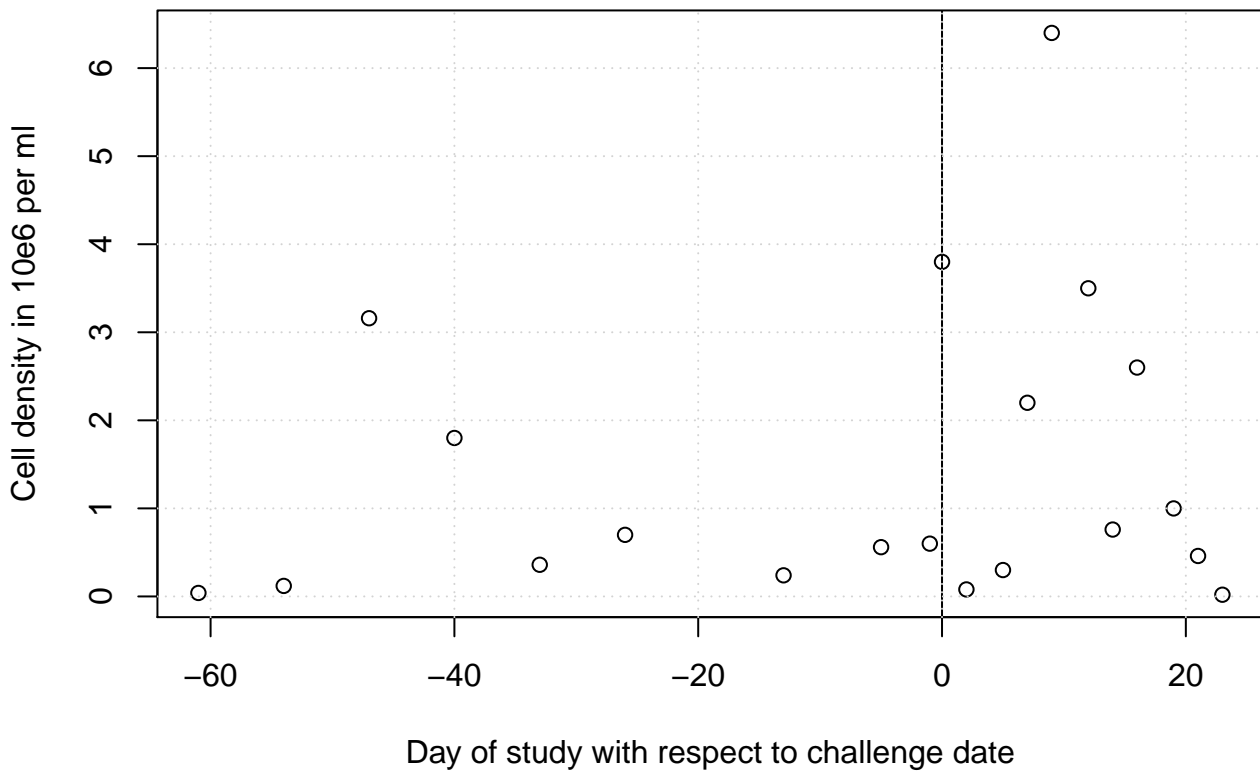

# P05A

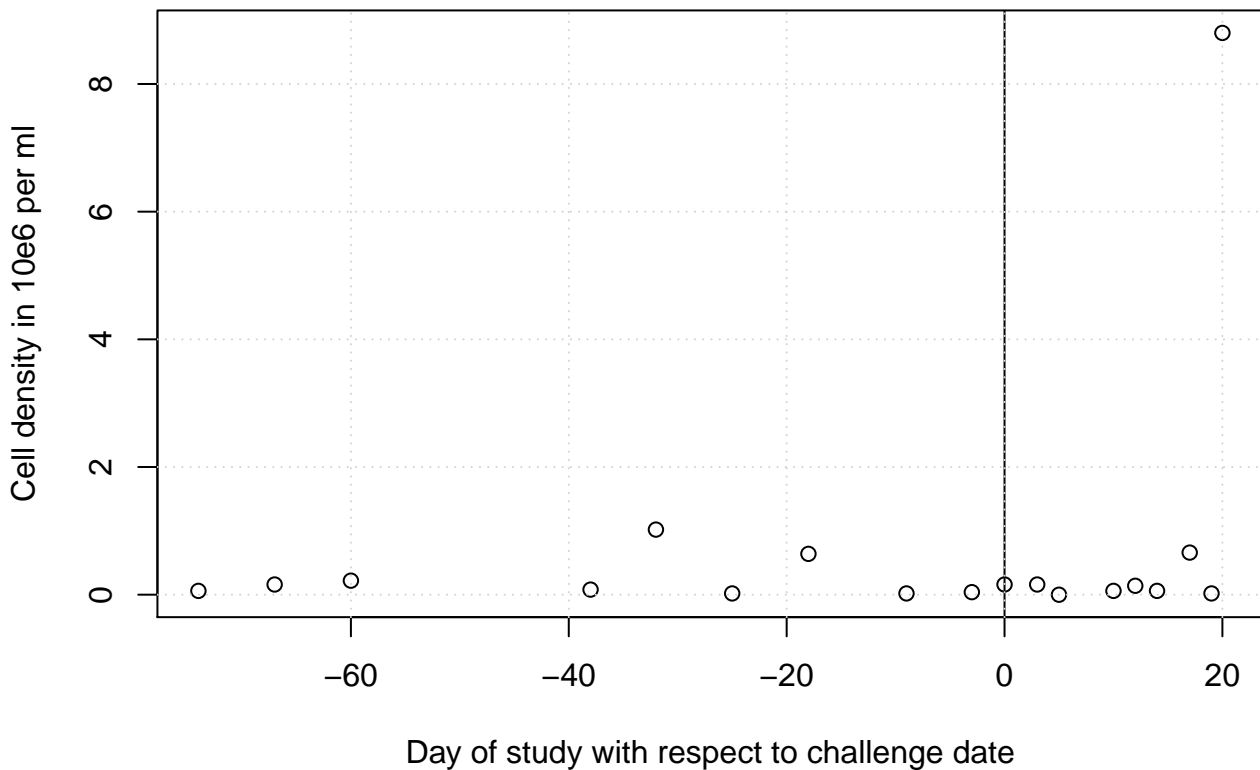

# P06A

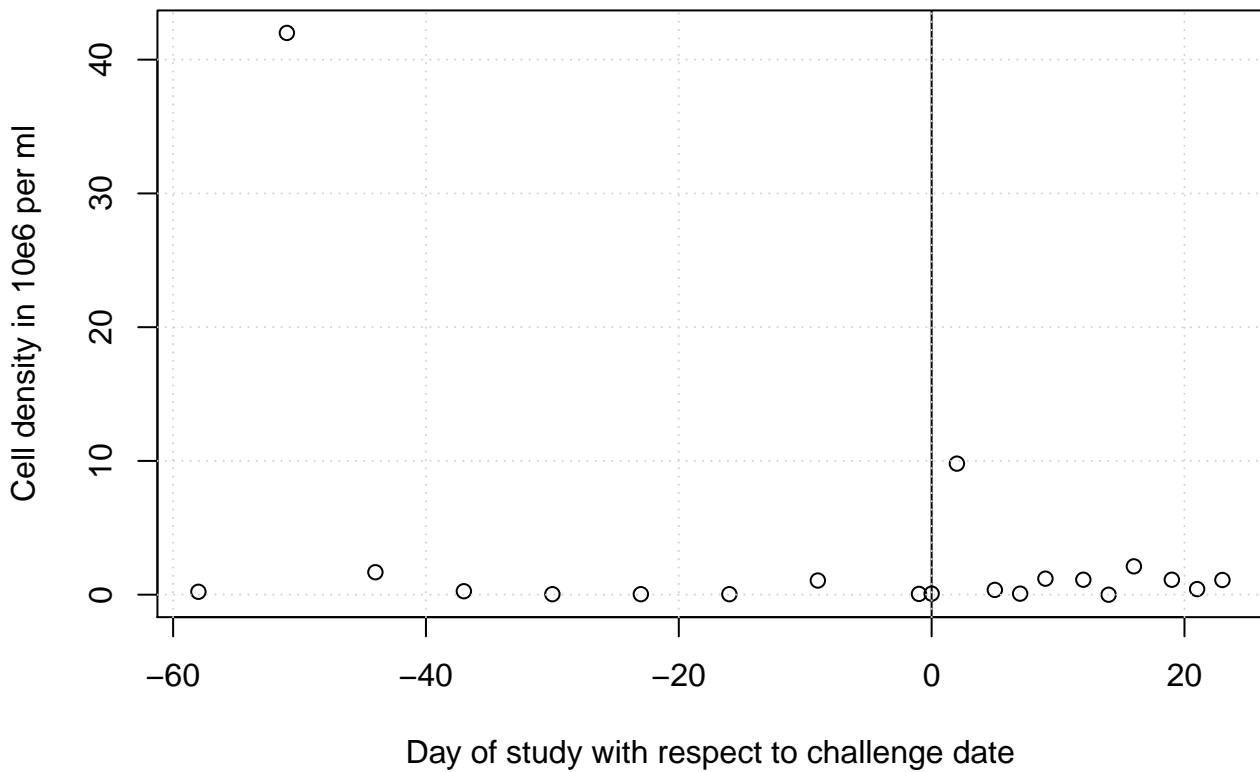

**P07A**

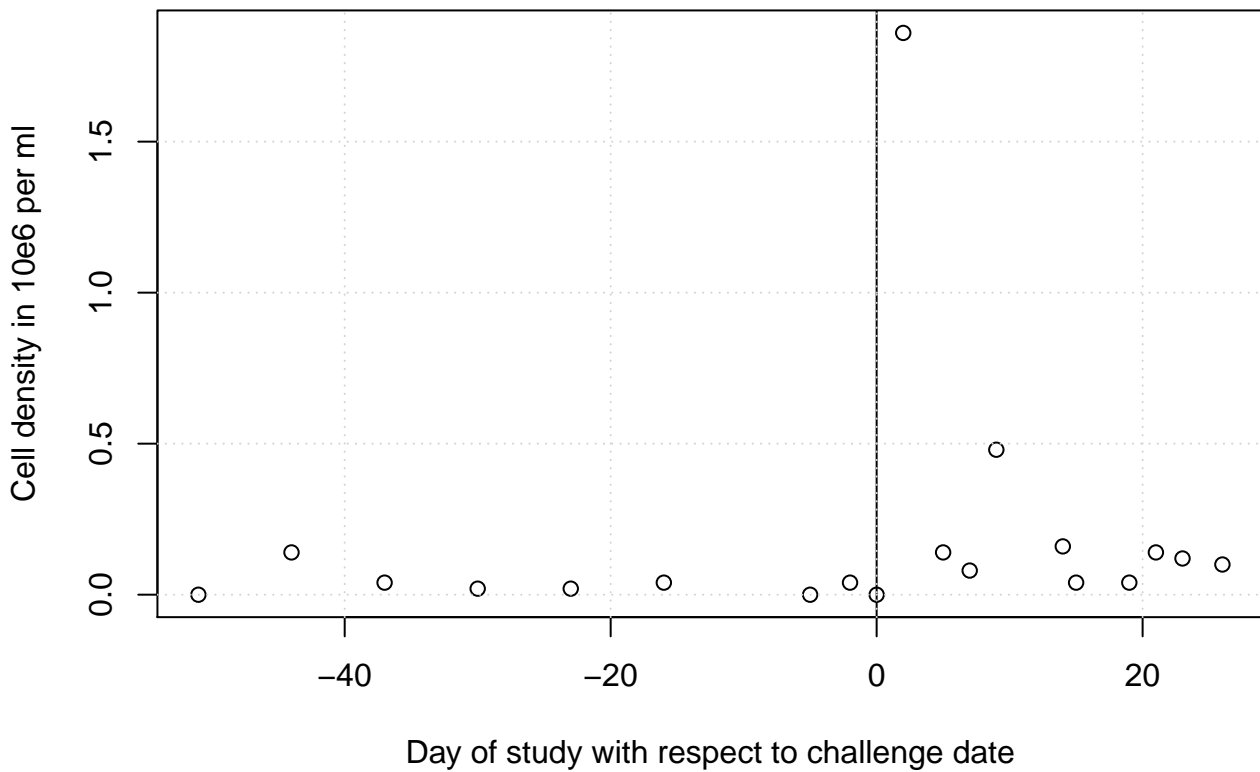

# P08A

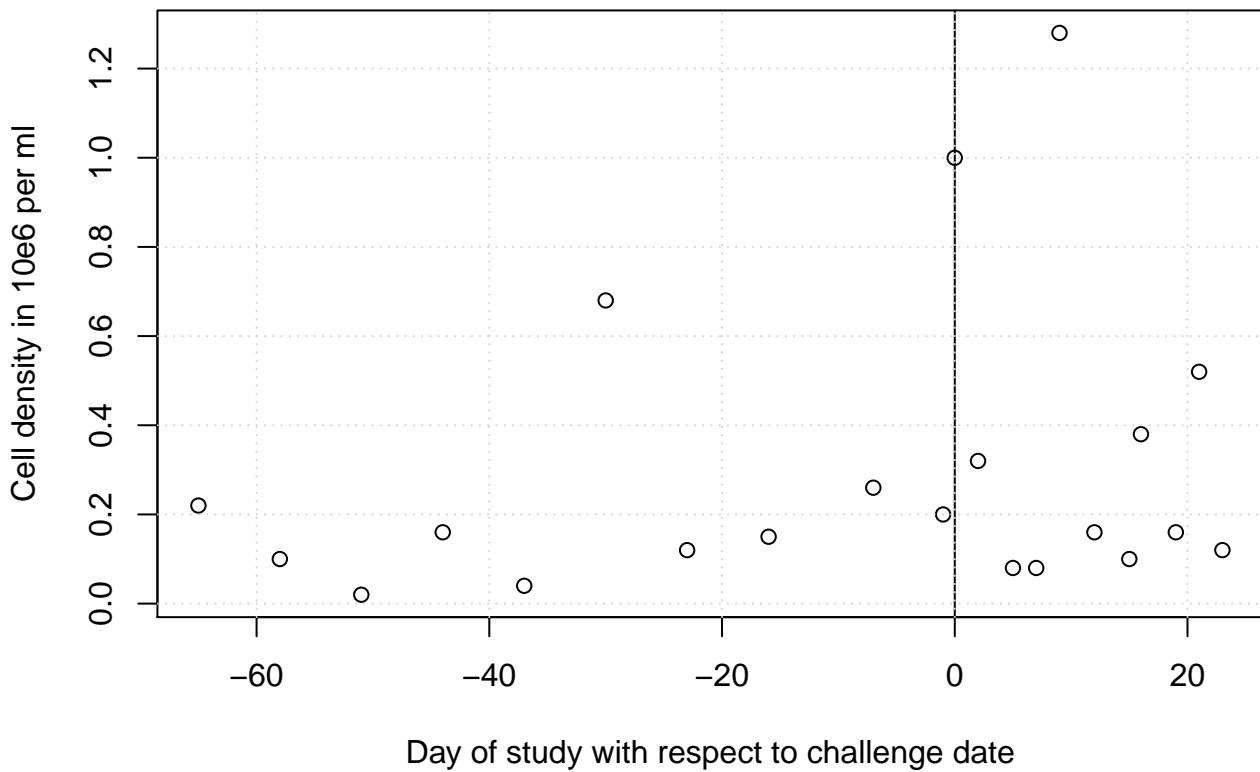

# P09A

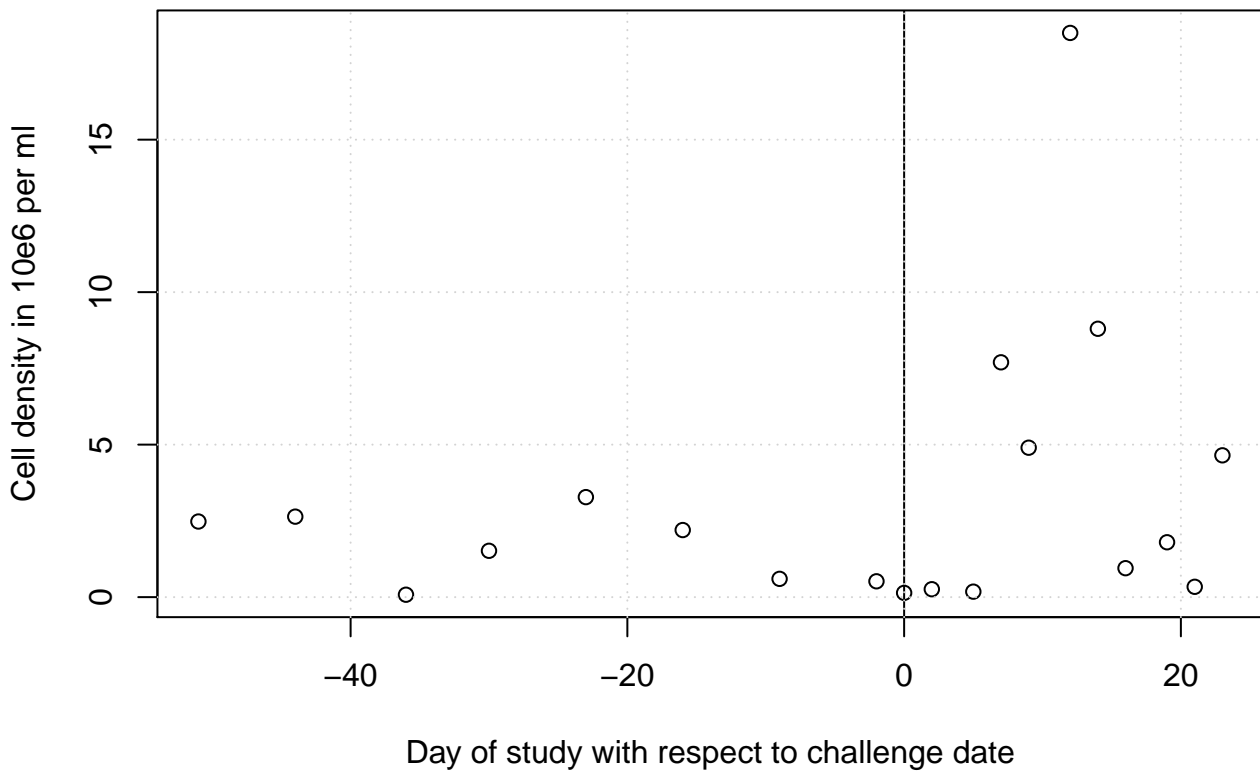

# P10A

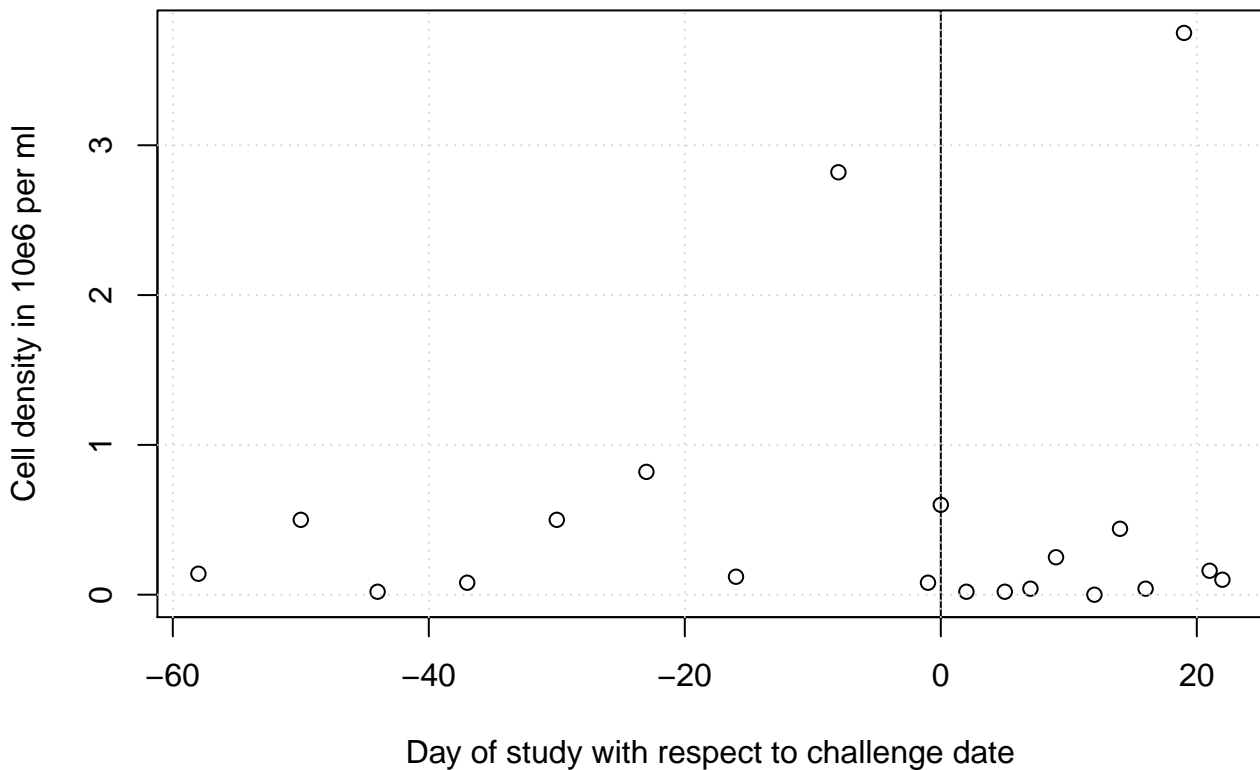

# P11A

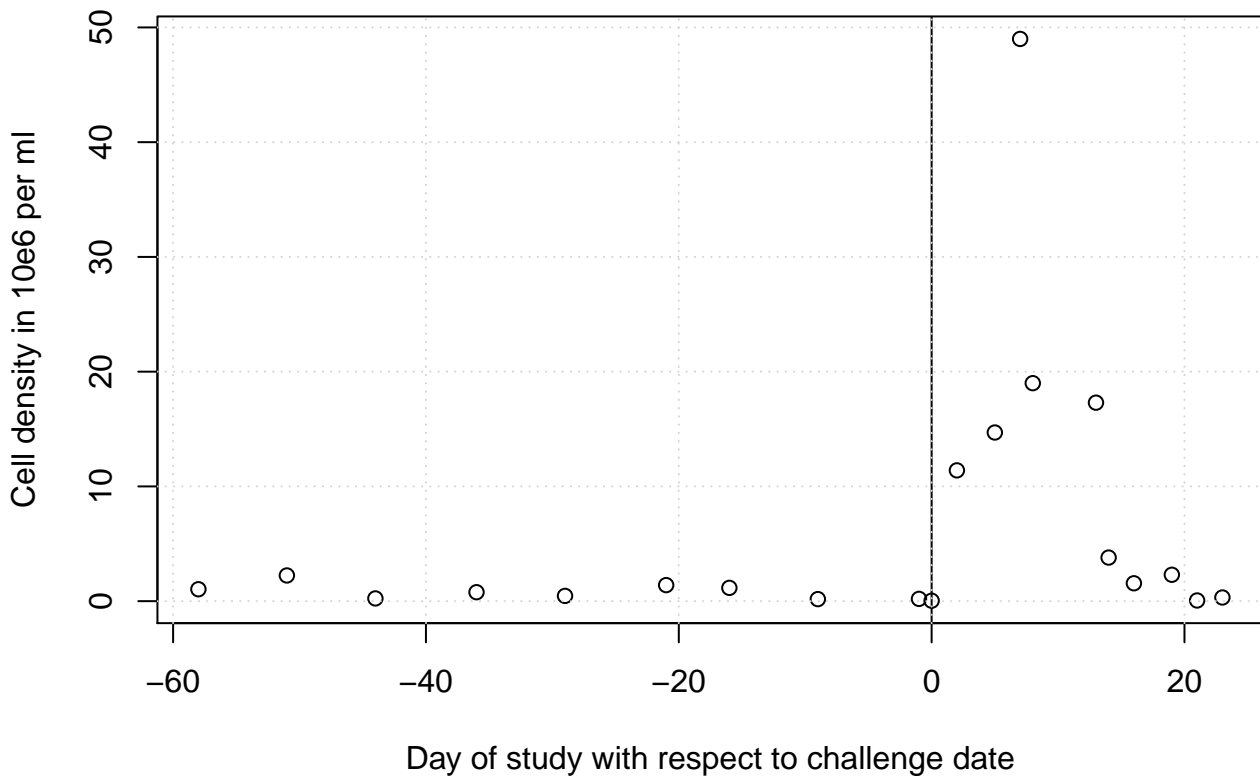

# P12A

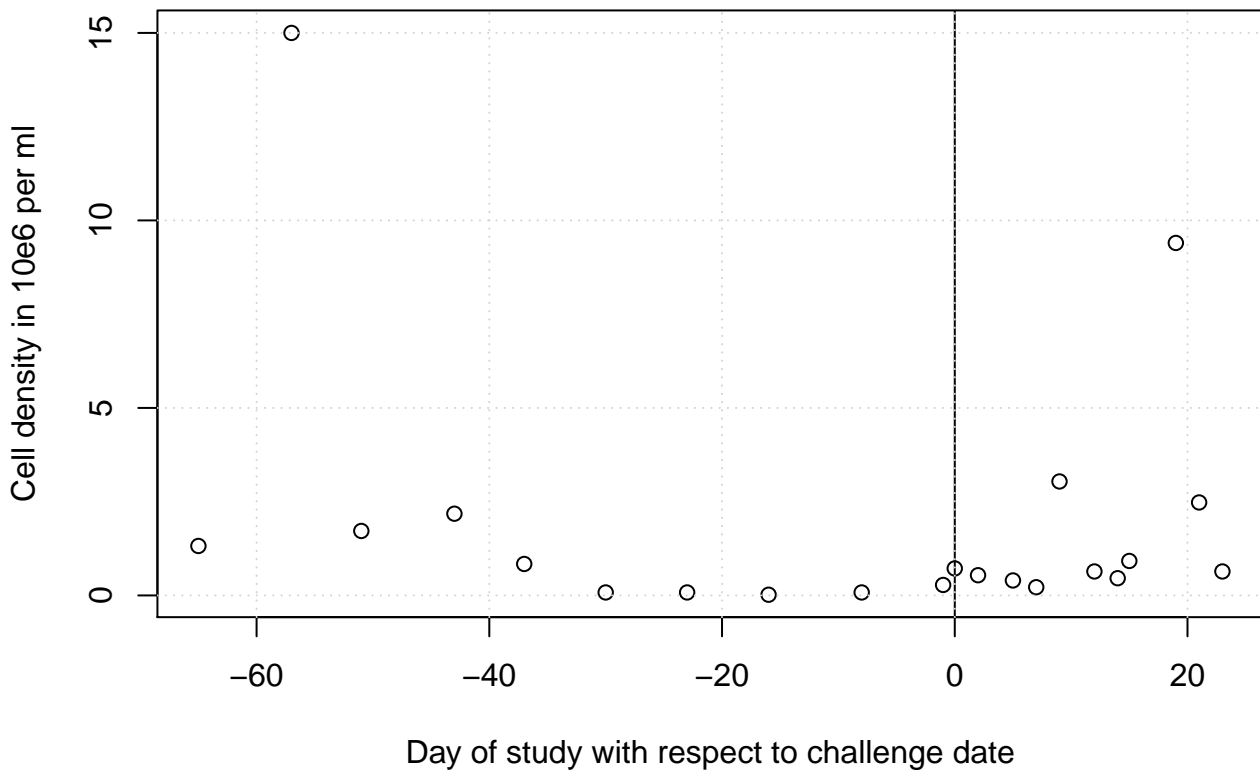

# P13A

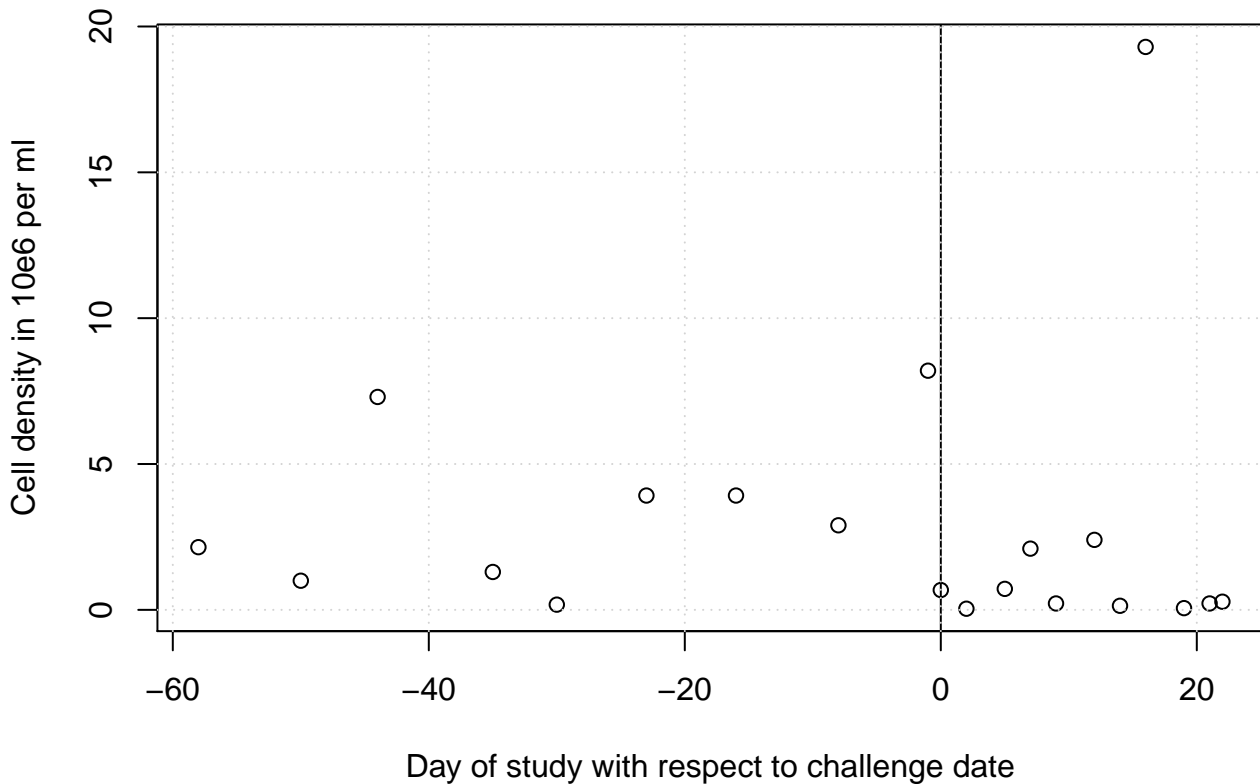

Supplement: Supplementary file 2. [file elife-47969-supp2.zip › TimeSeriesPlots_PDFs/Appendix-figure SS12_CellDensity_In10e6_Per_ml_TimeSeries_AsthmaticParticipants.pdf]

# P01H

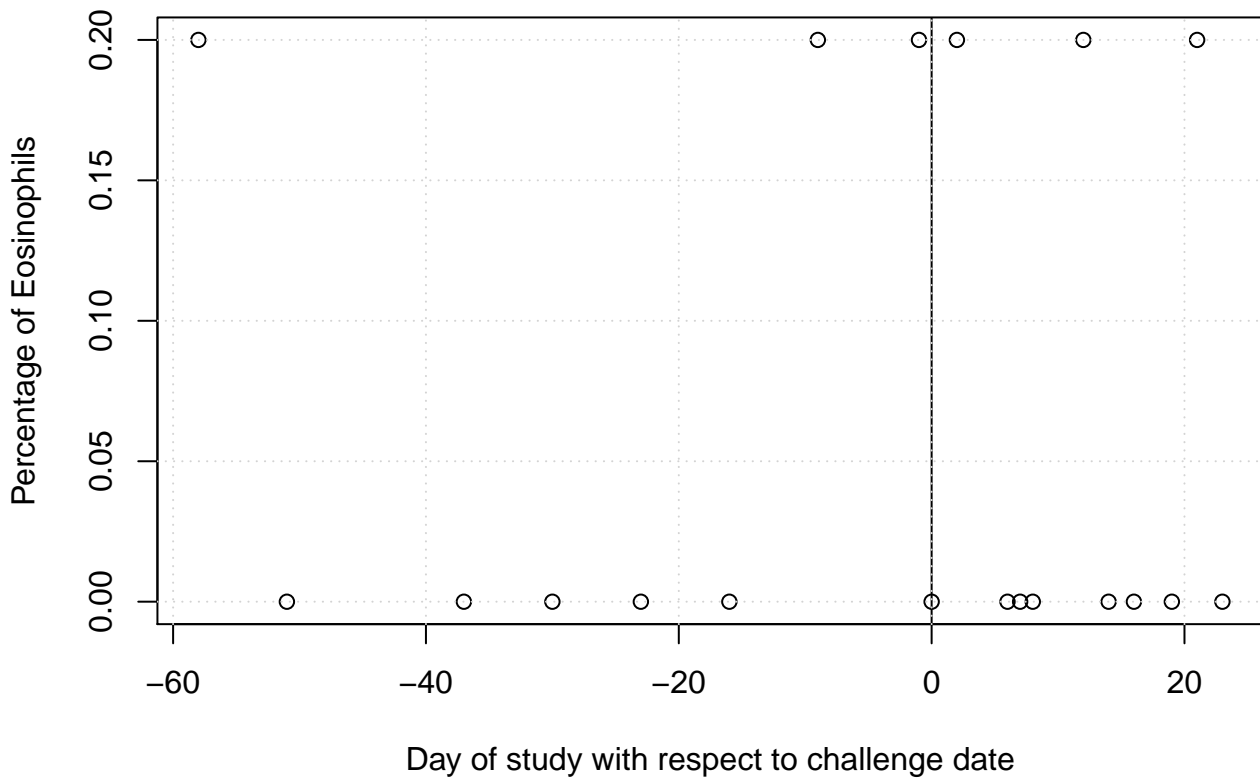

# P03H

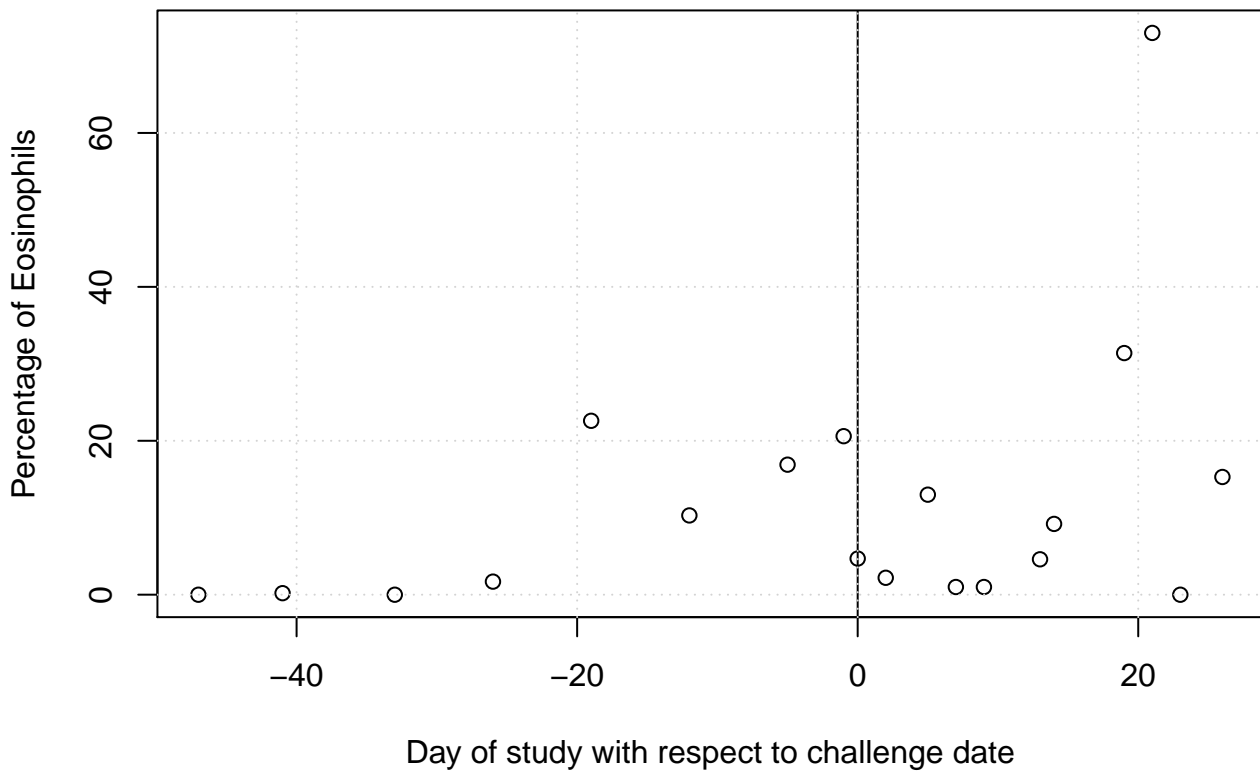

# P05H

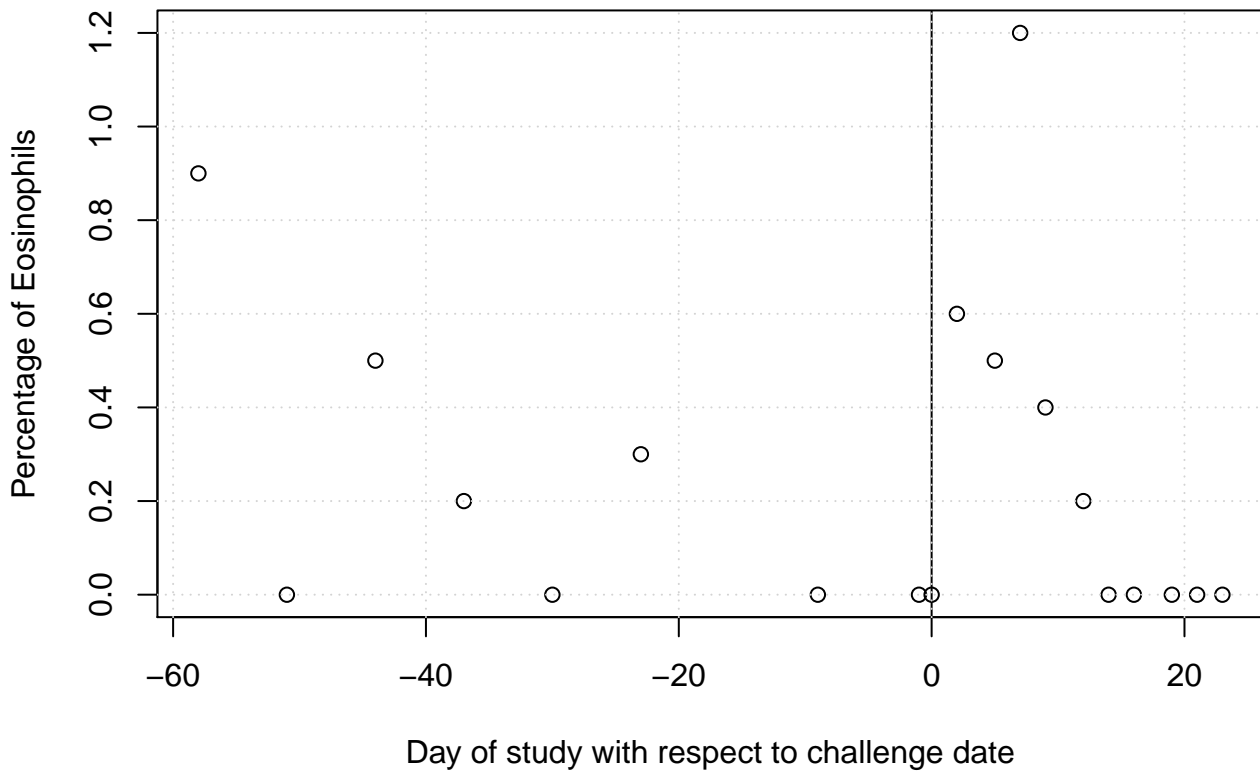

**P06H**

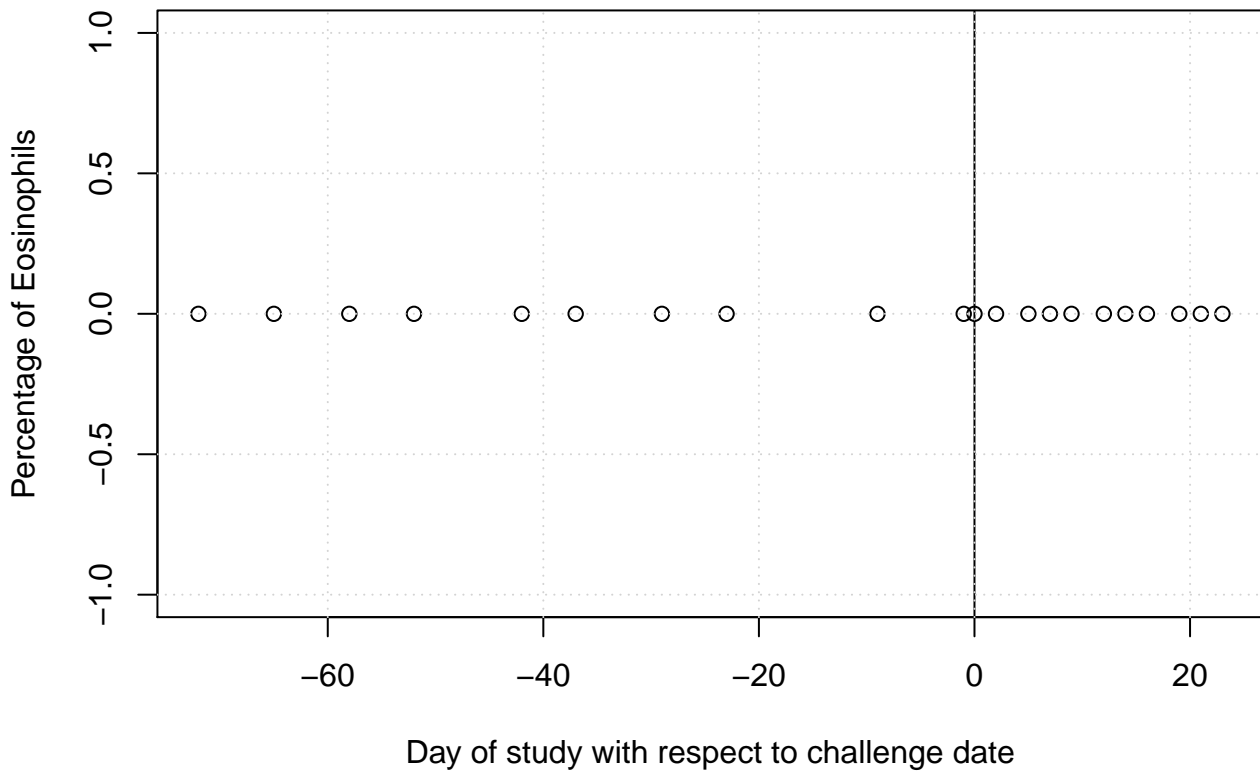

# P07H

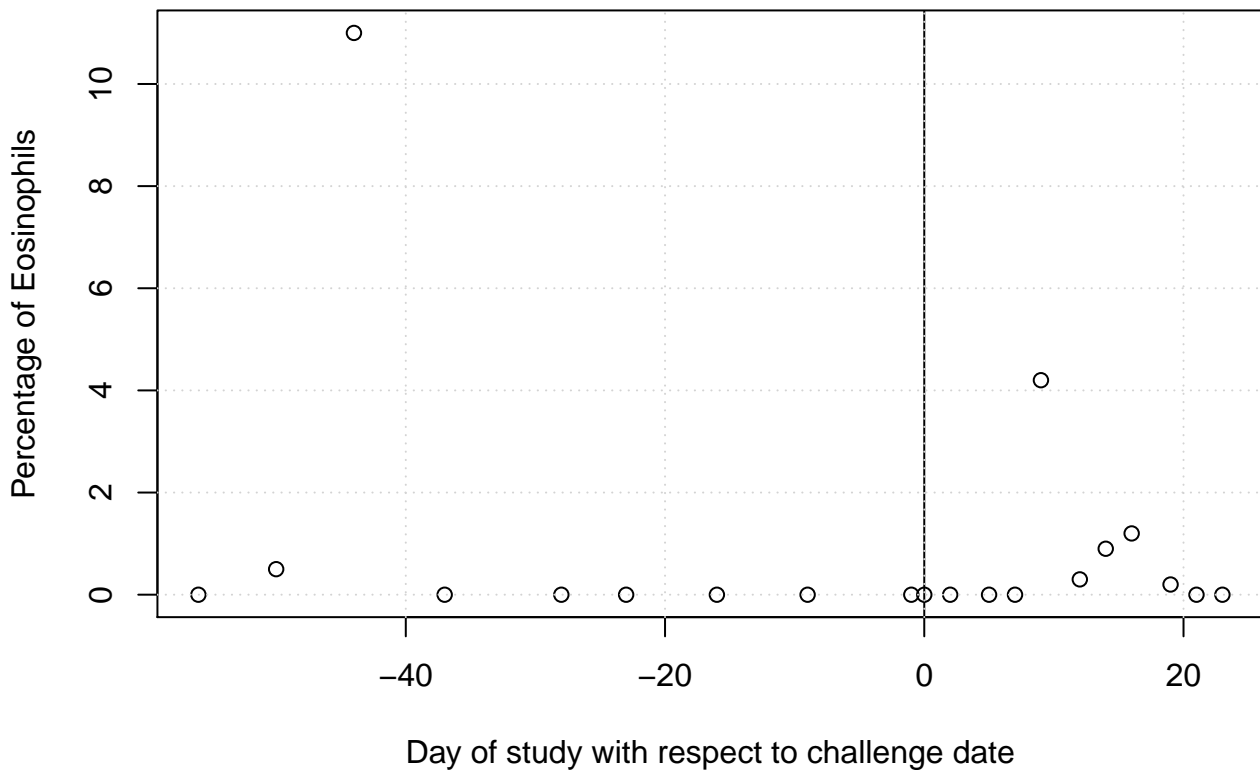

**P08H**

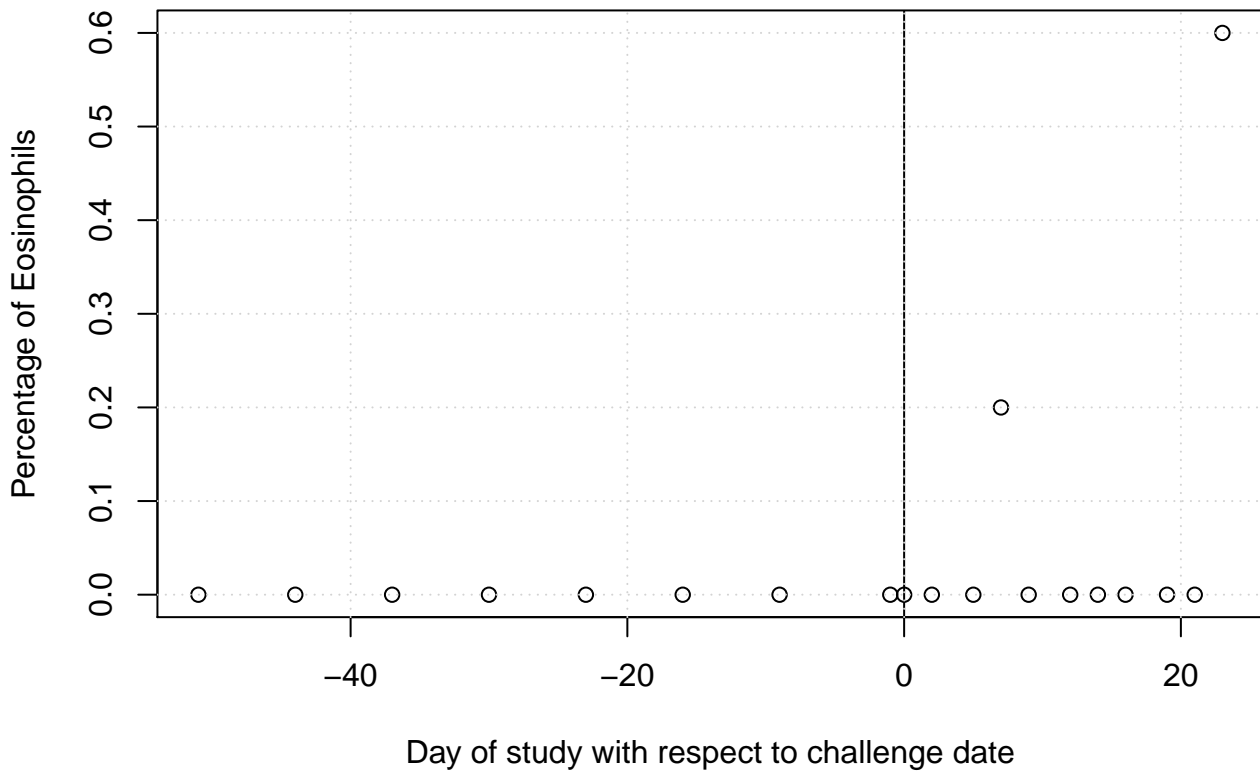

**P09H**

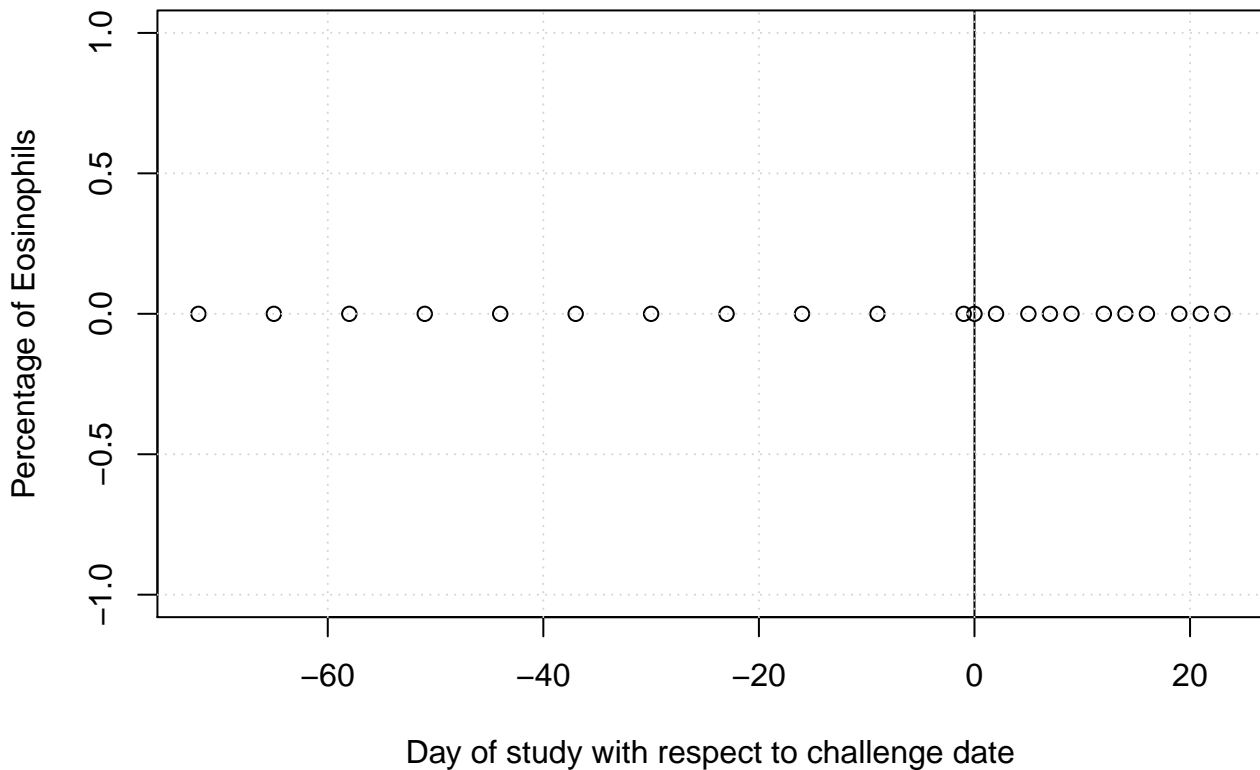

# P11H

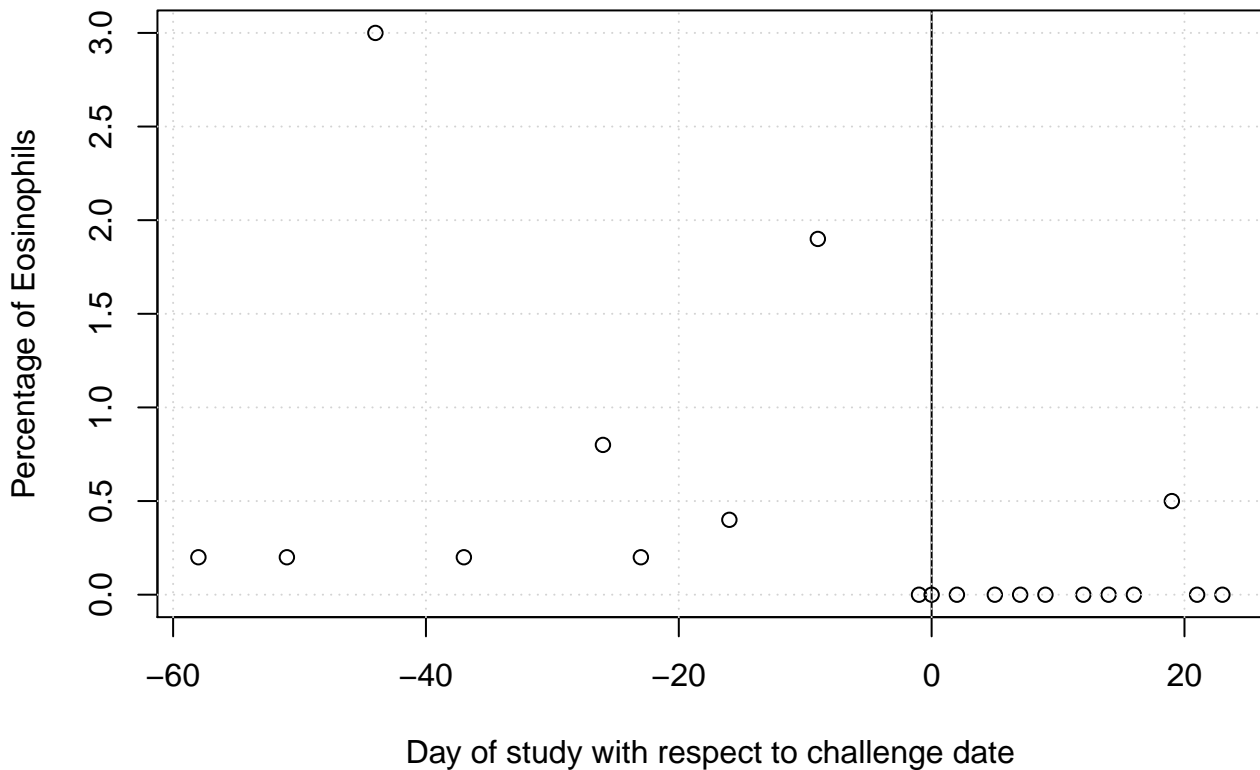

## P12H

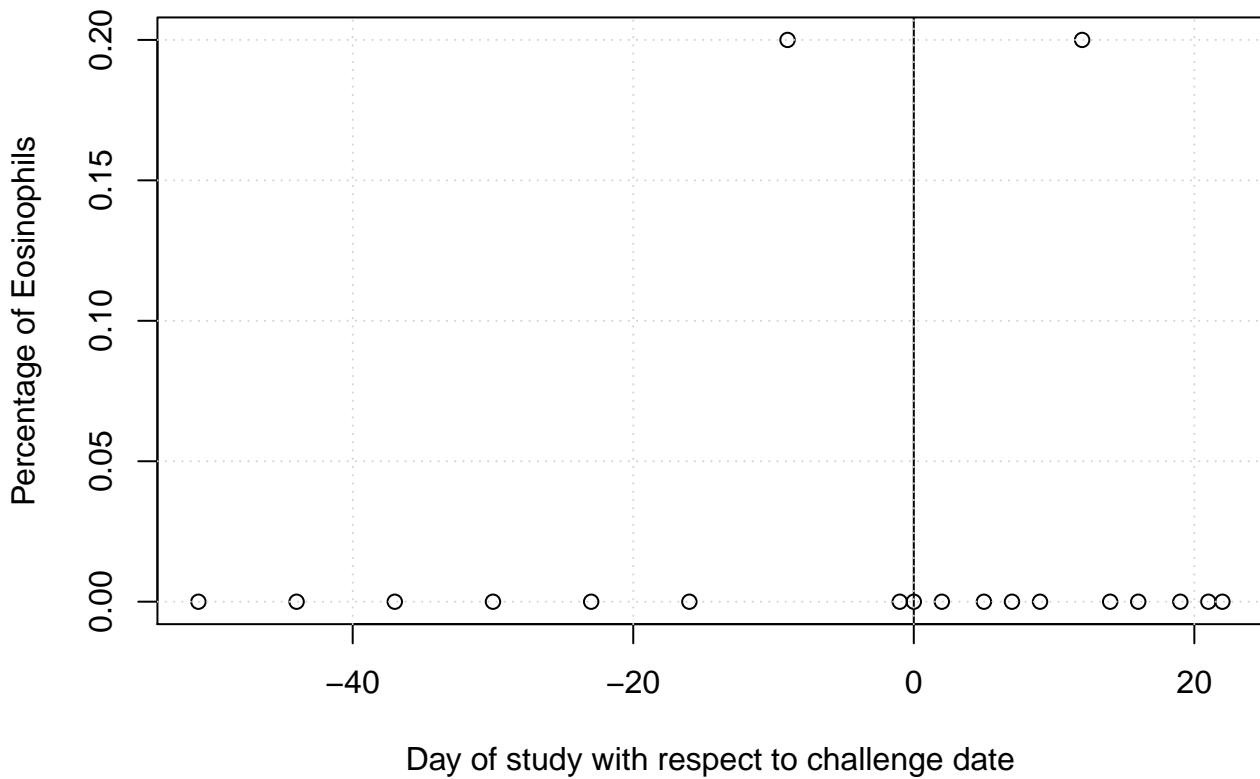

# P13H

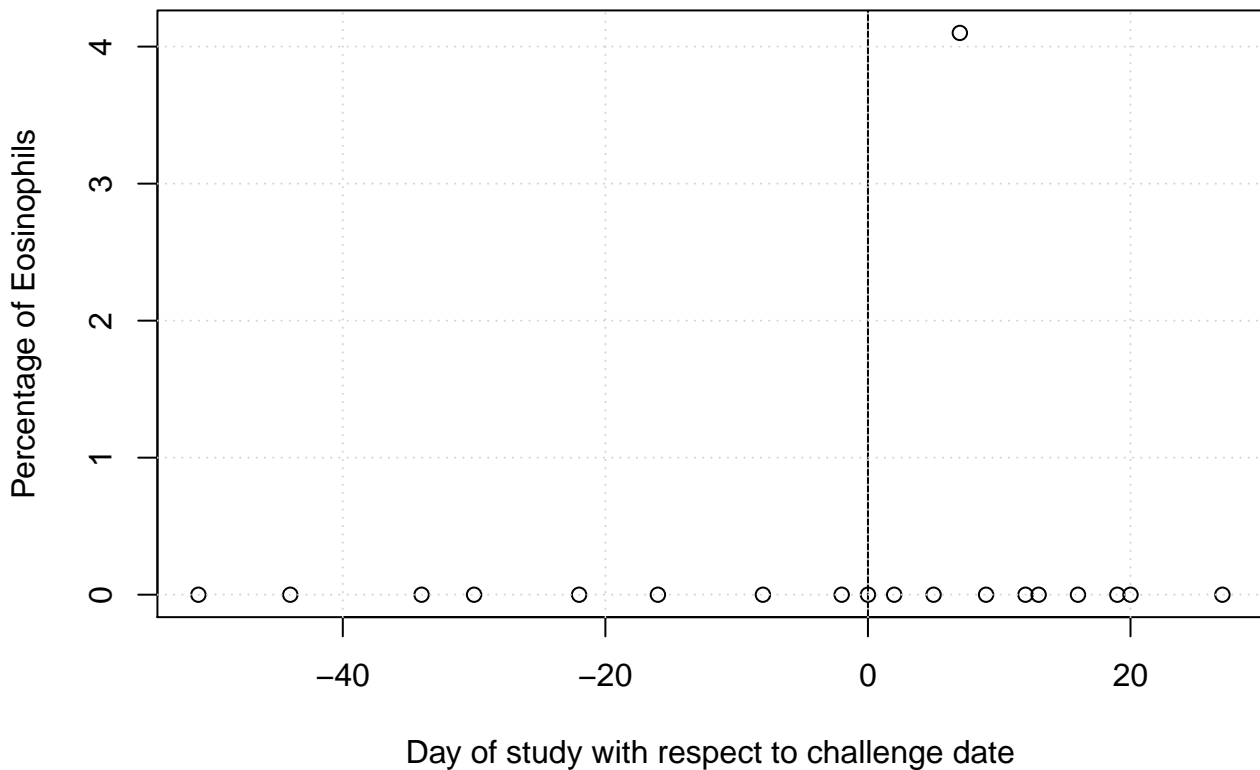

# P14H

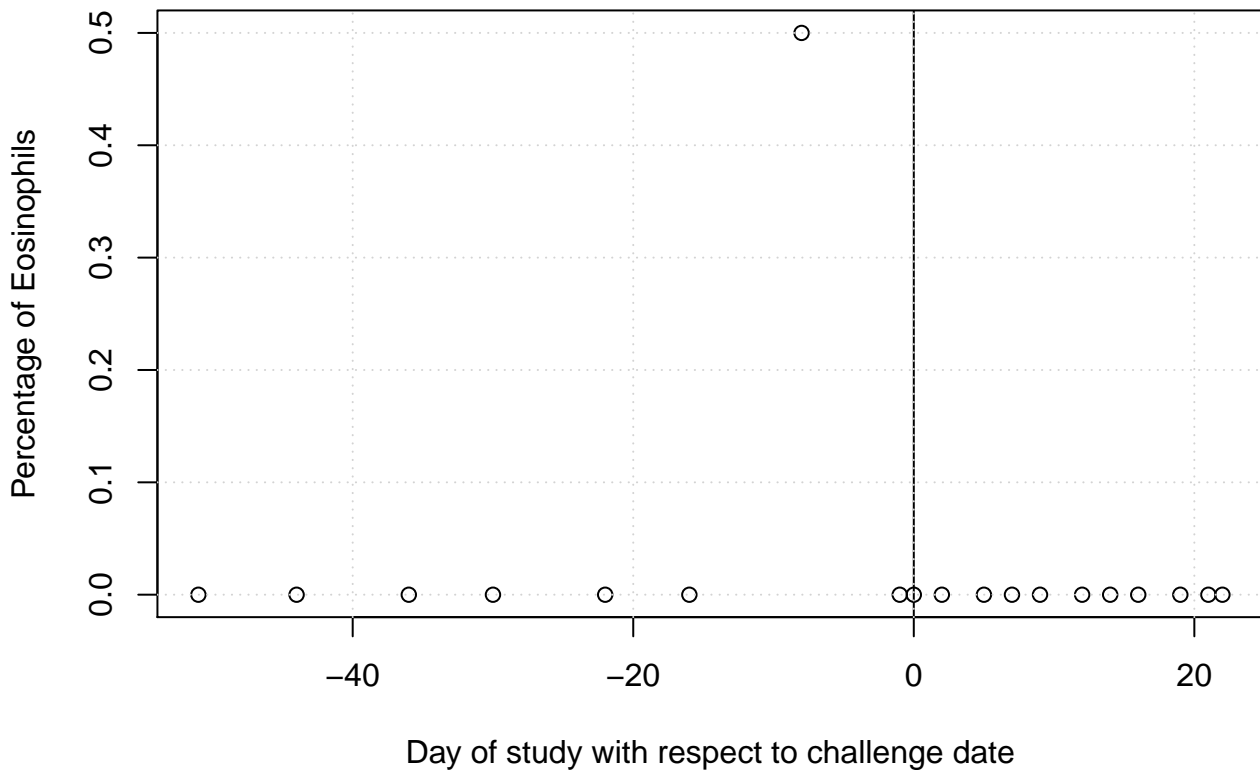

# P15H

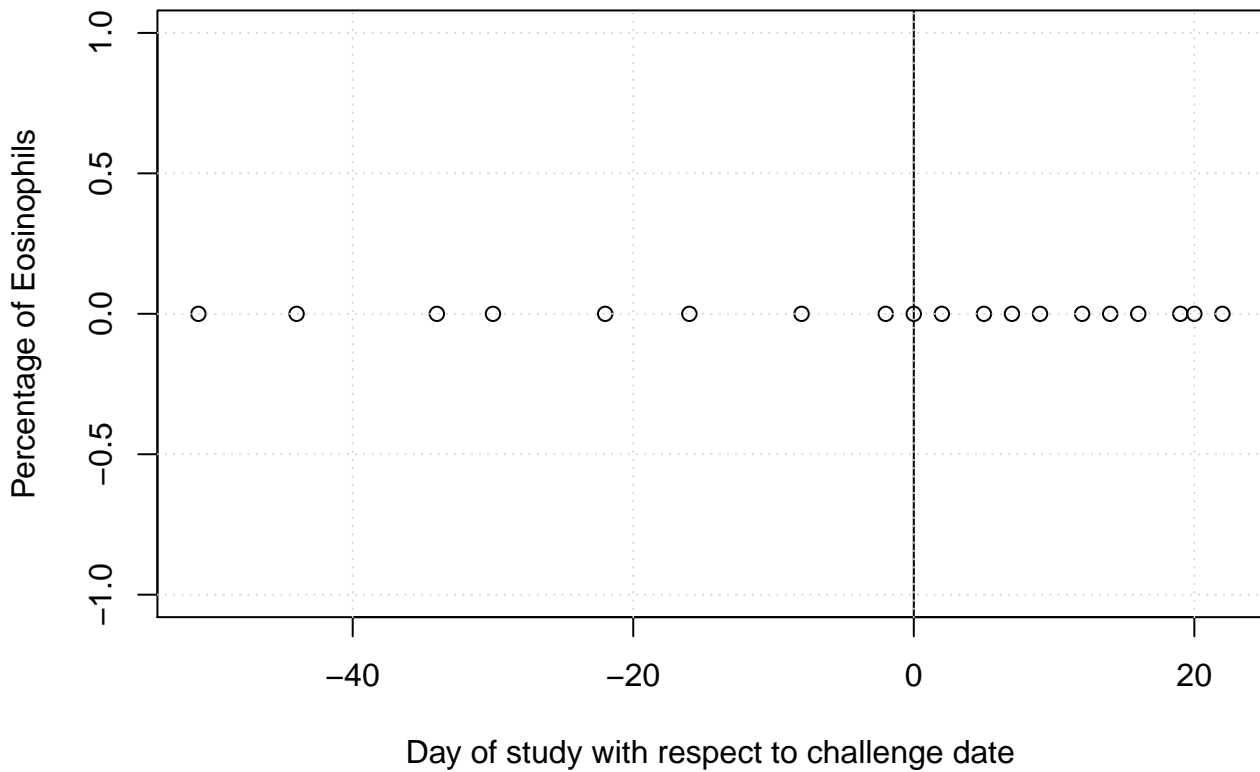

Supplement: Supplementary file 2. [file elife-47969-supp2.zip › TimeSeriesPlots_PDFs/Appendix-figure SS13_PercentageOfEosinophils_TimeSeries_HealthyParticipants.pdf]

# P01H

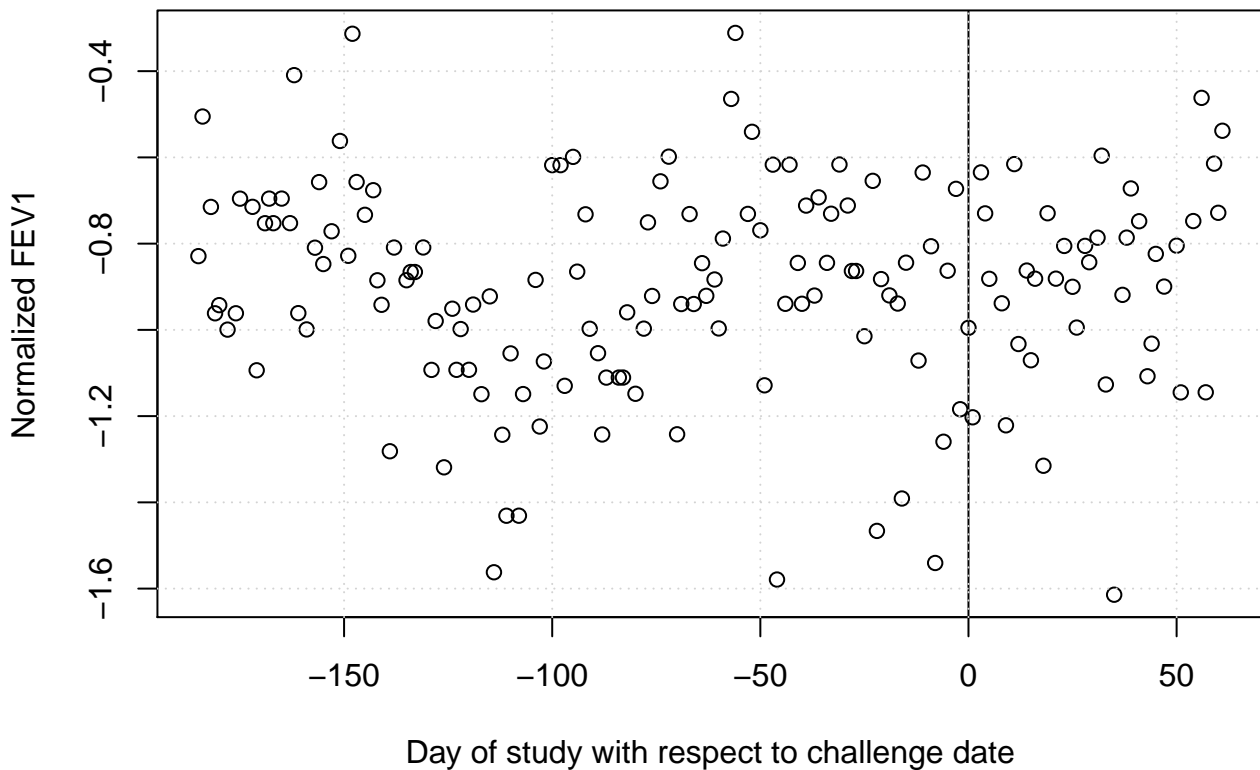

# P03H

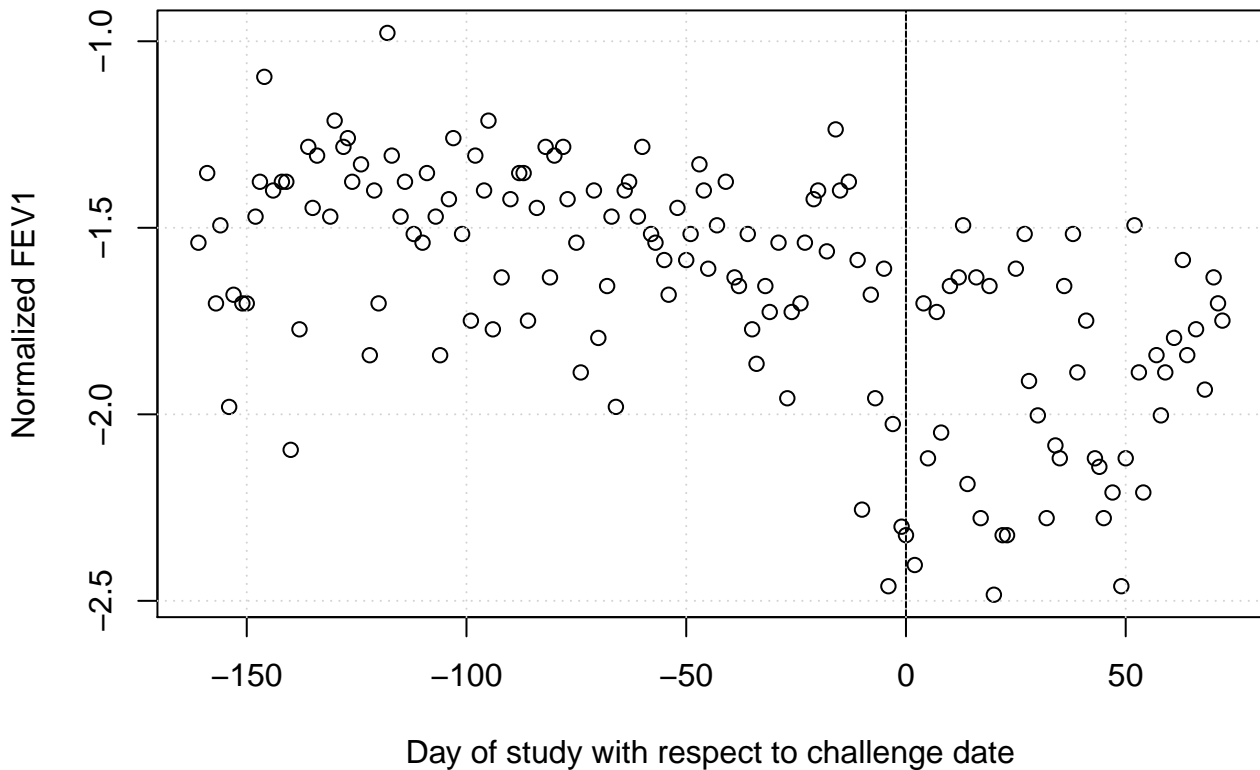

# P05H

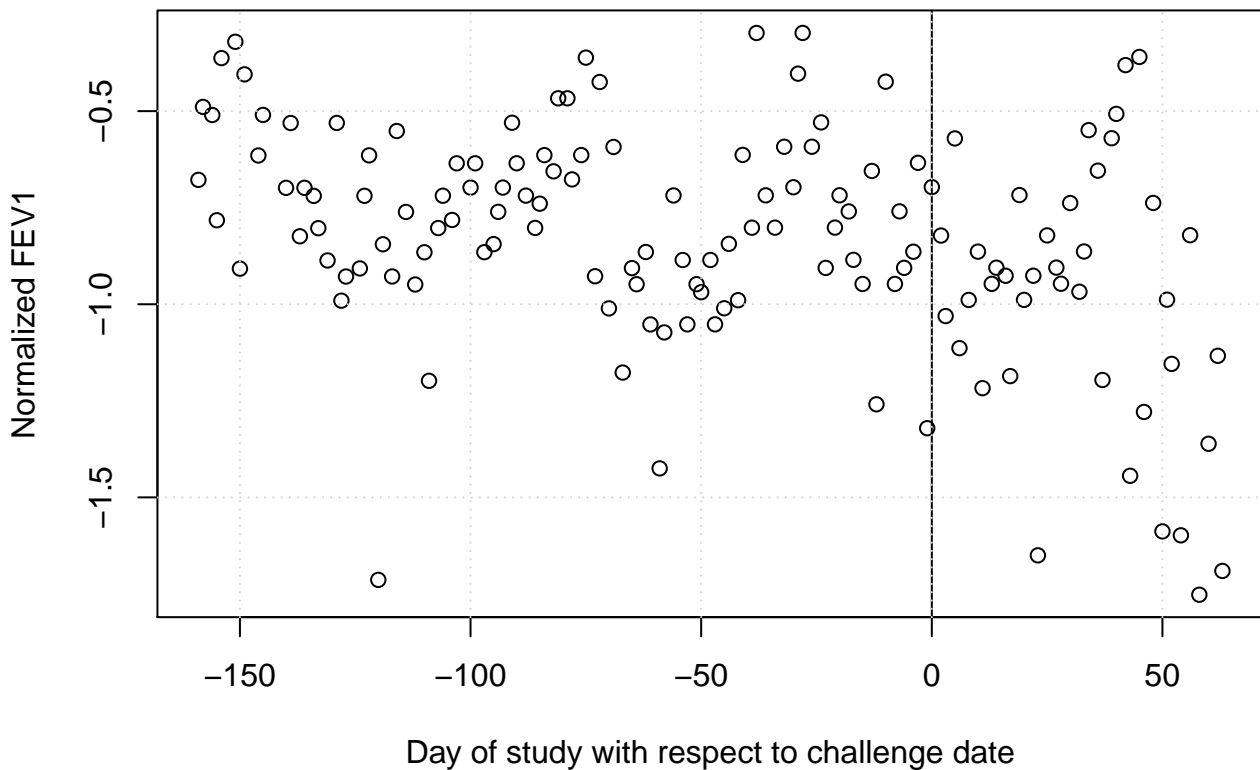

# P06H

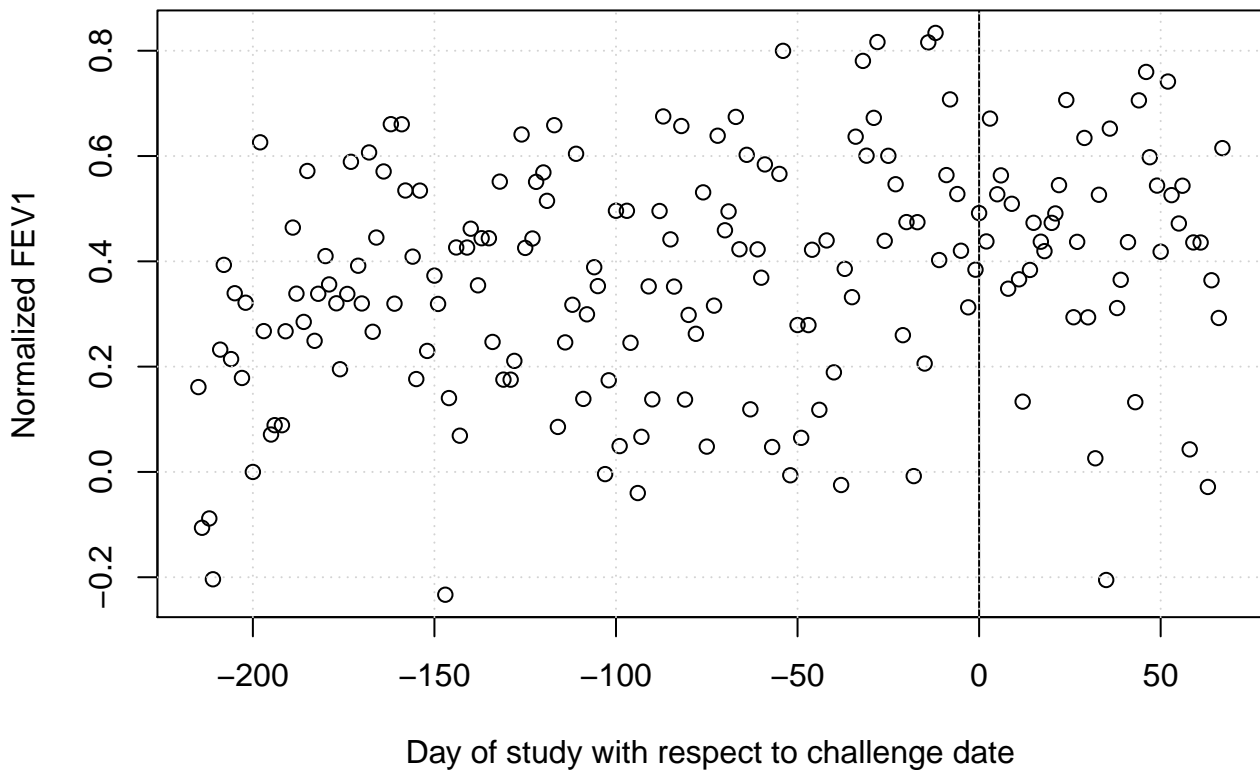

# P07H

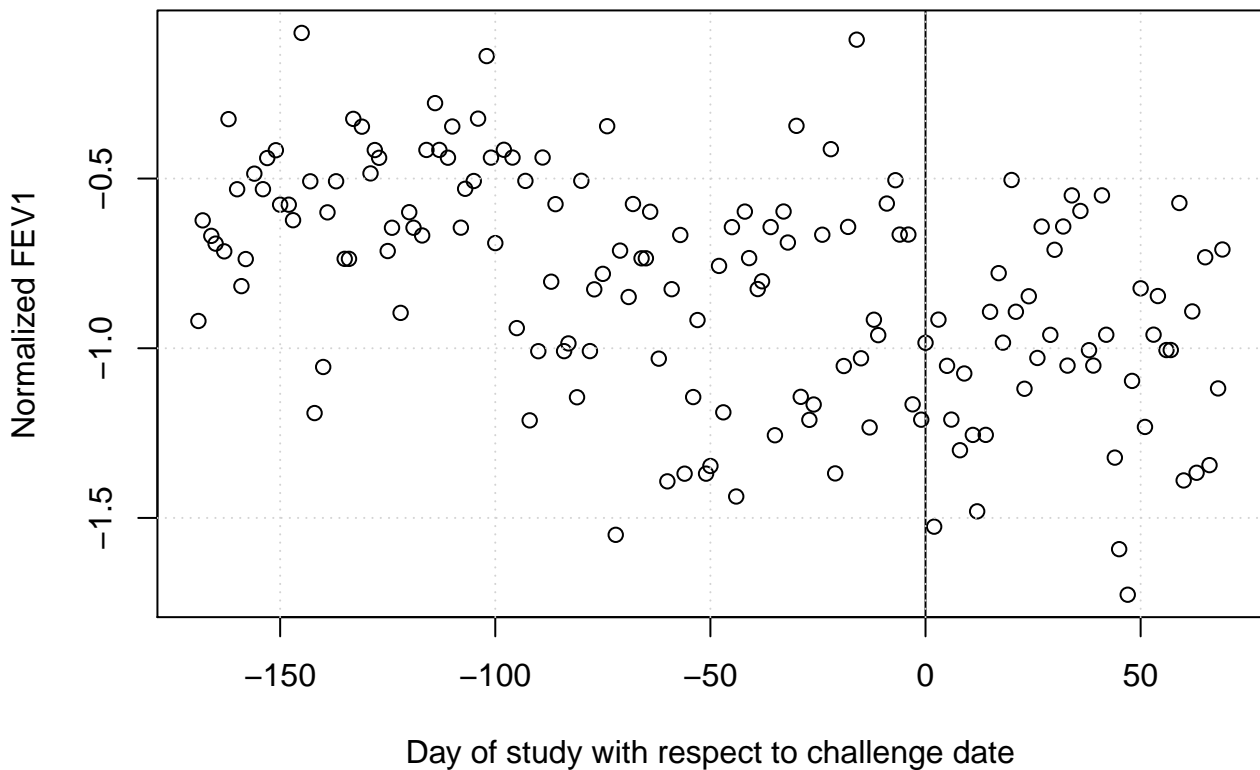

**P08H**

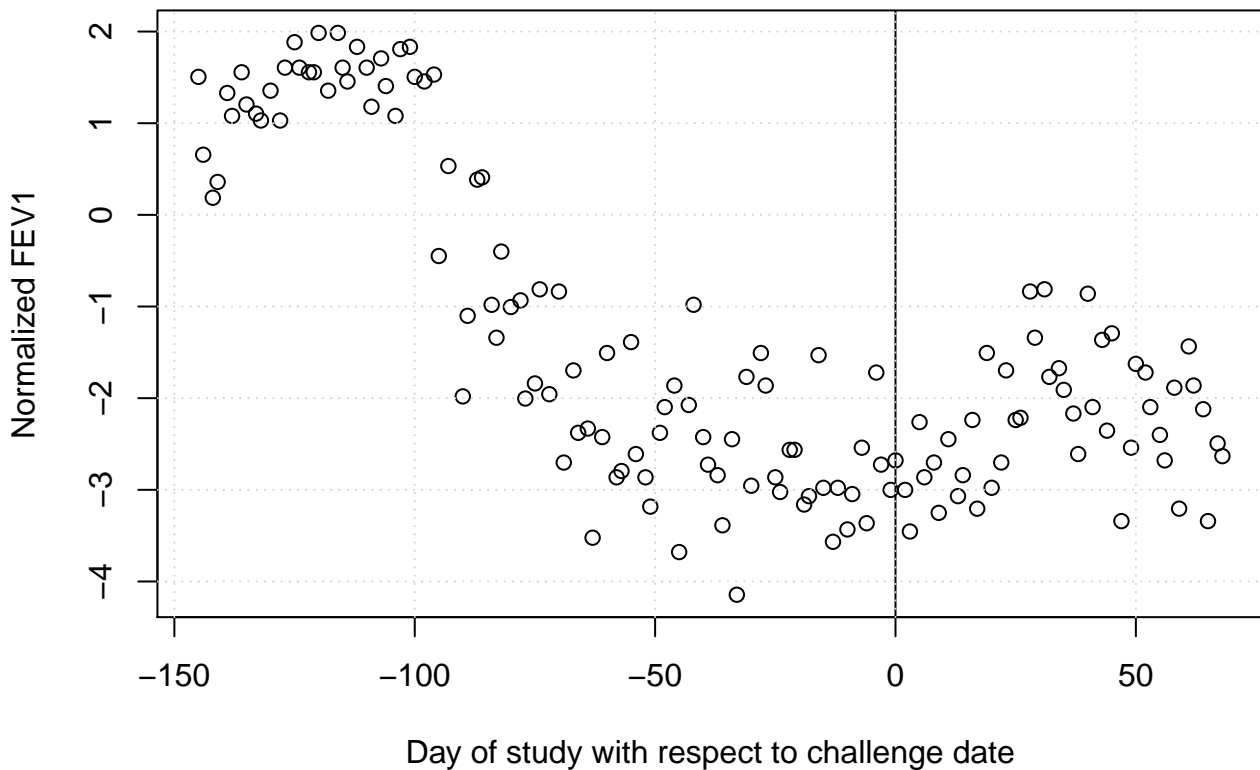

**P09H**

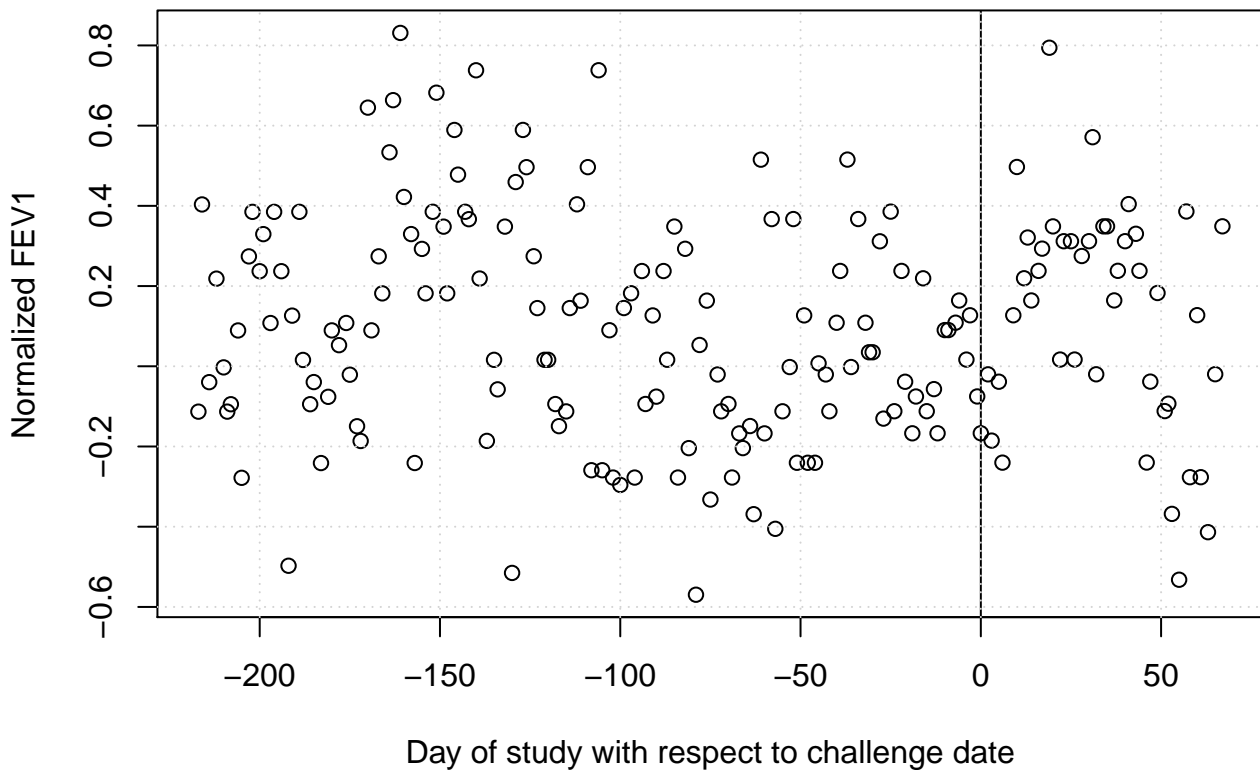

# P11H

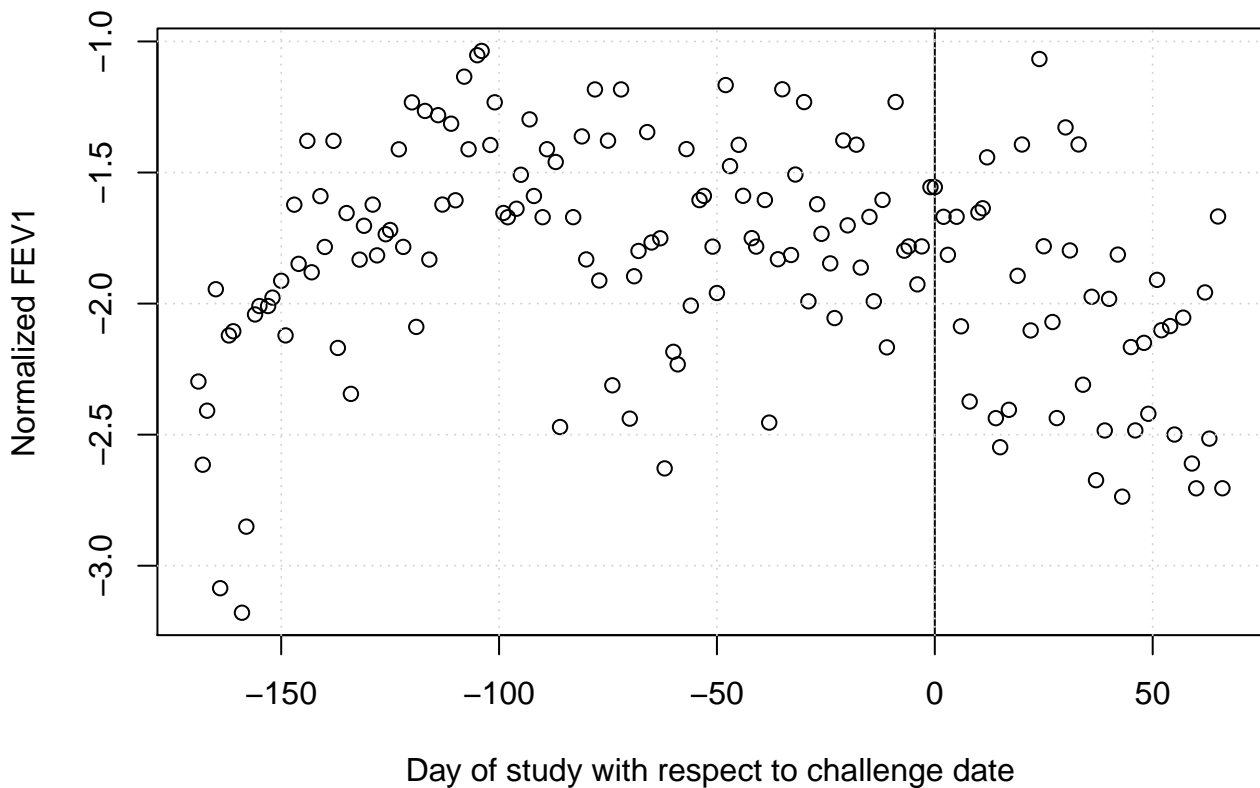

# P12H

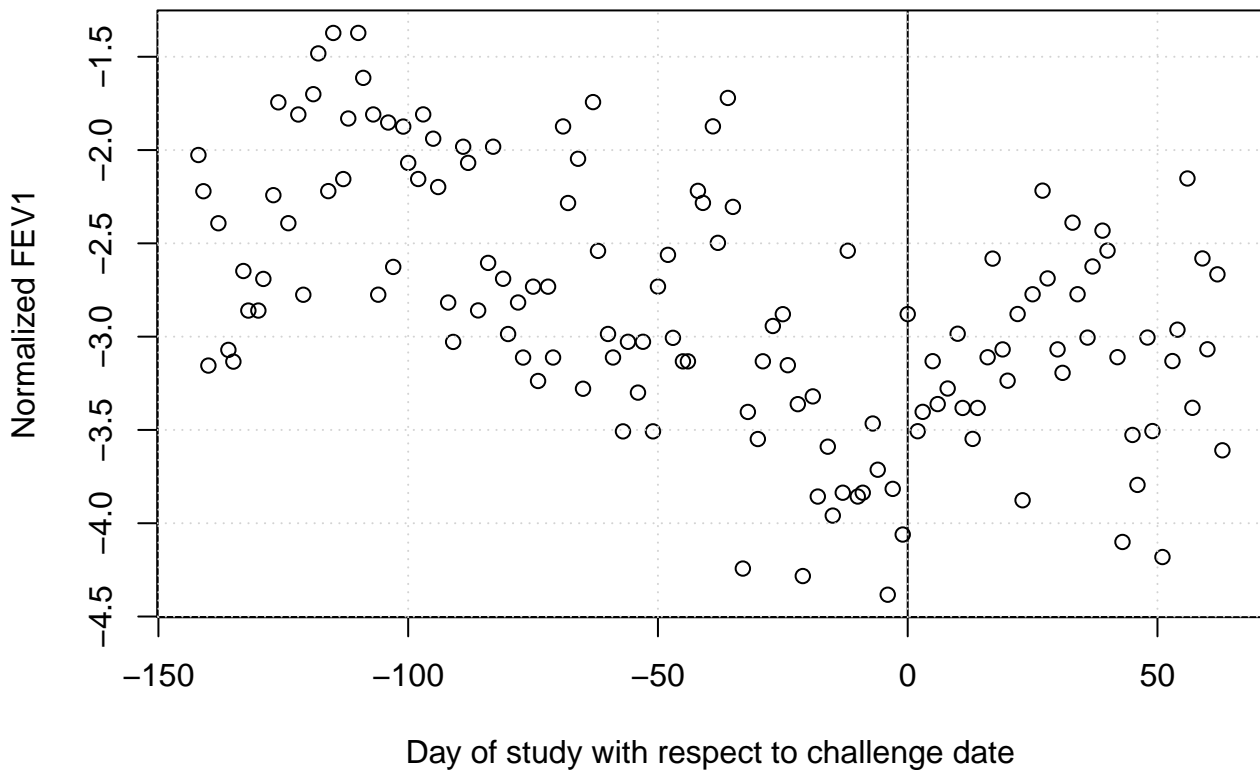

# P13H

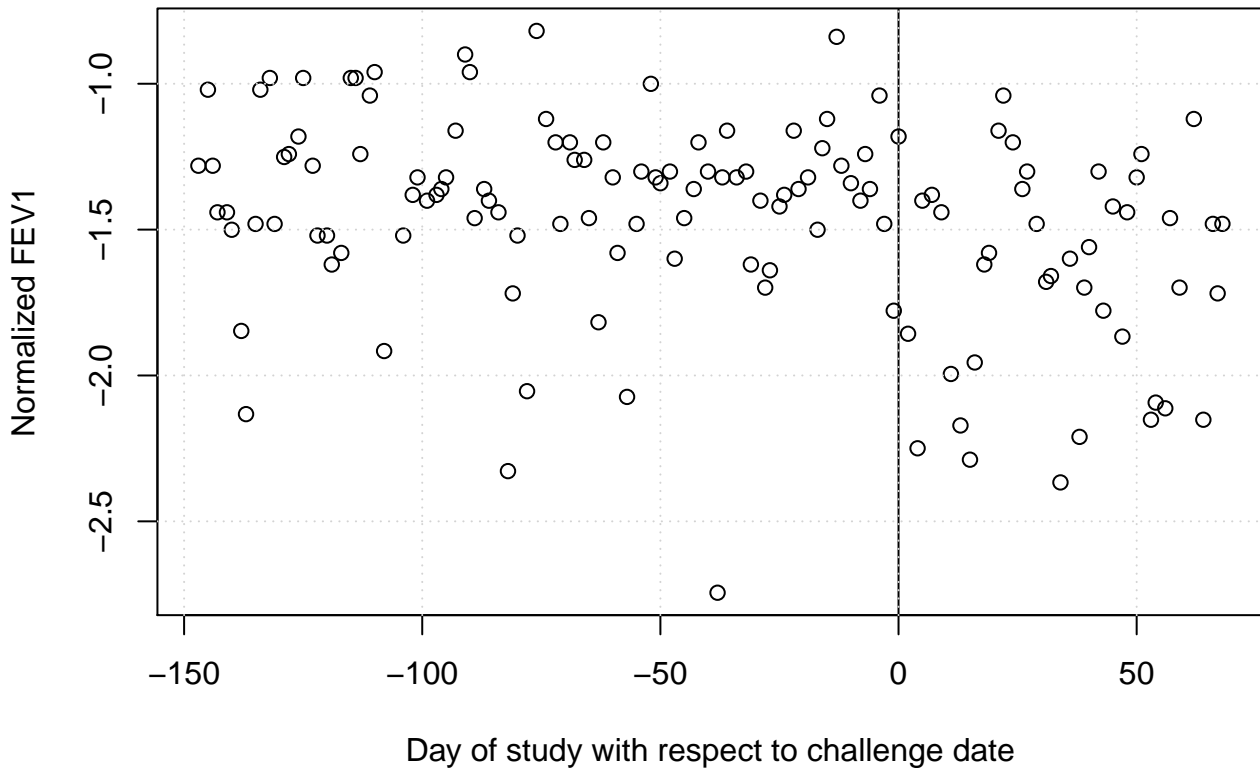

# P14H

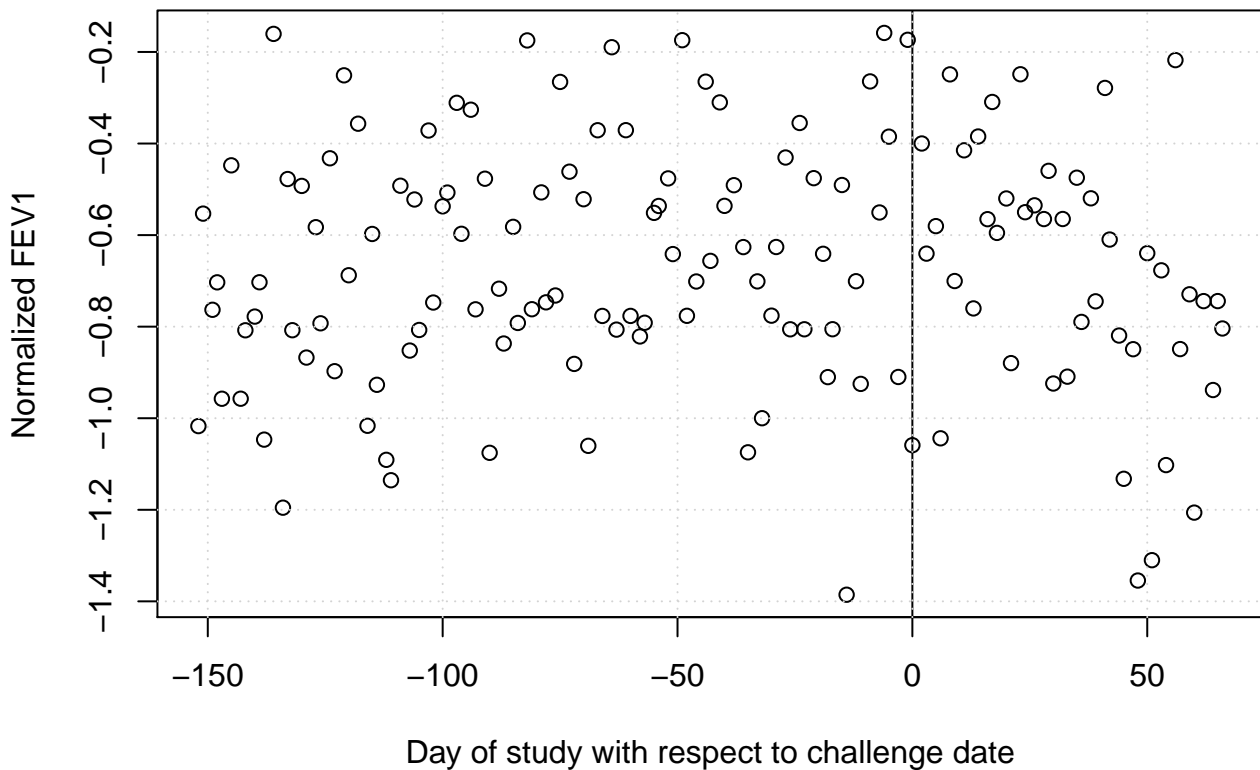

# P15H

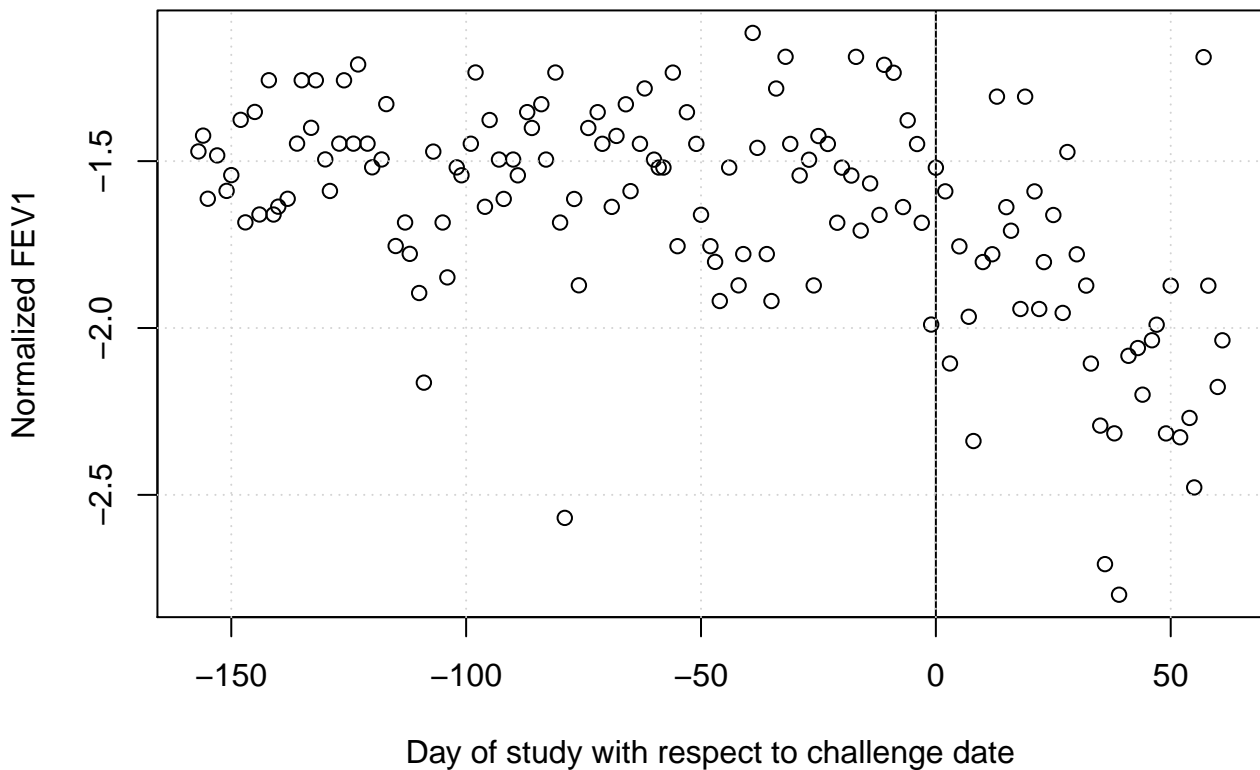

Supplement: Supplementary file 2. [file elife-47969-supp2.zip › TimeSeriesPlots_PDFs/Appendix-figure SS3_NFEV1_TimeSeries_HealthyParticipants.pdf]

# P01A

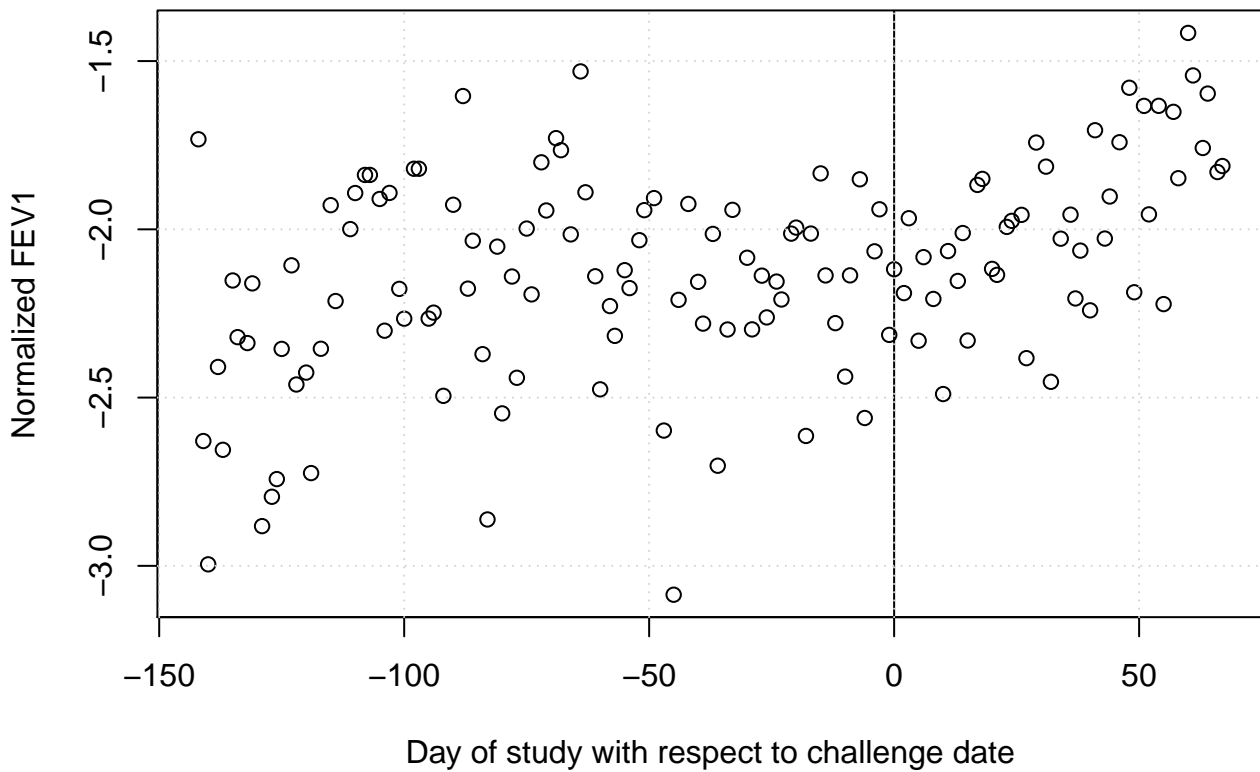

## P02A

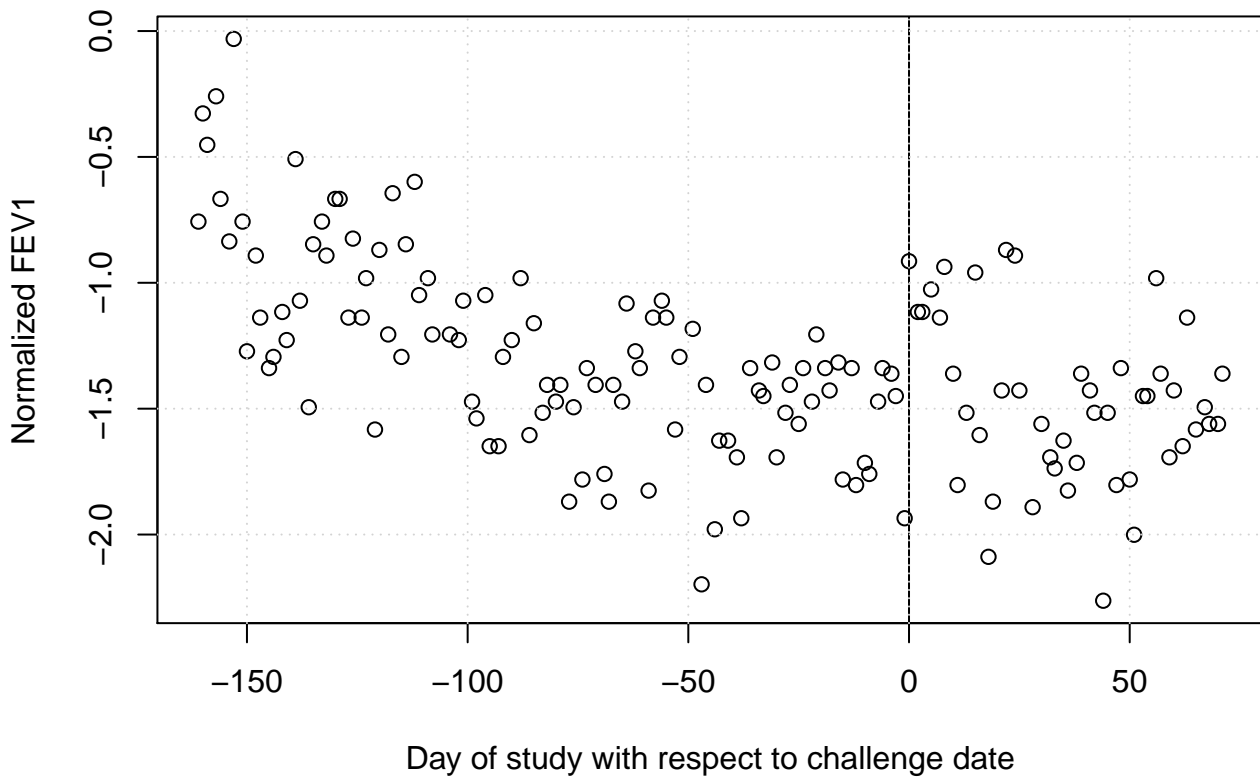

**P04A**

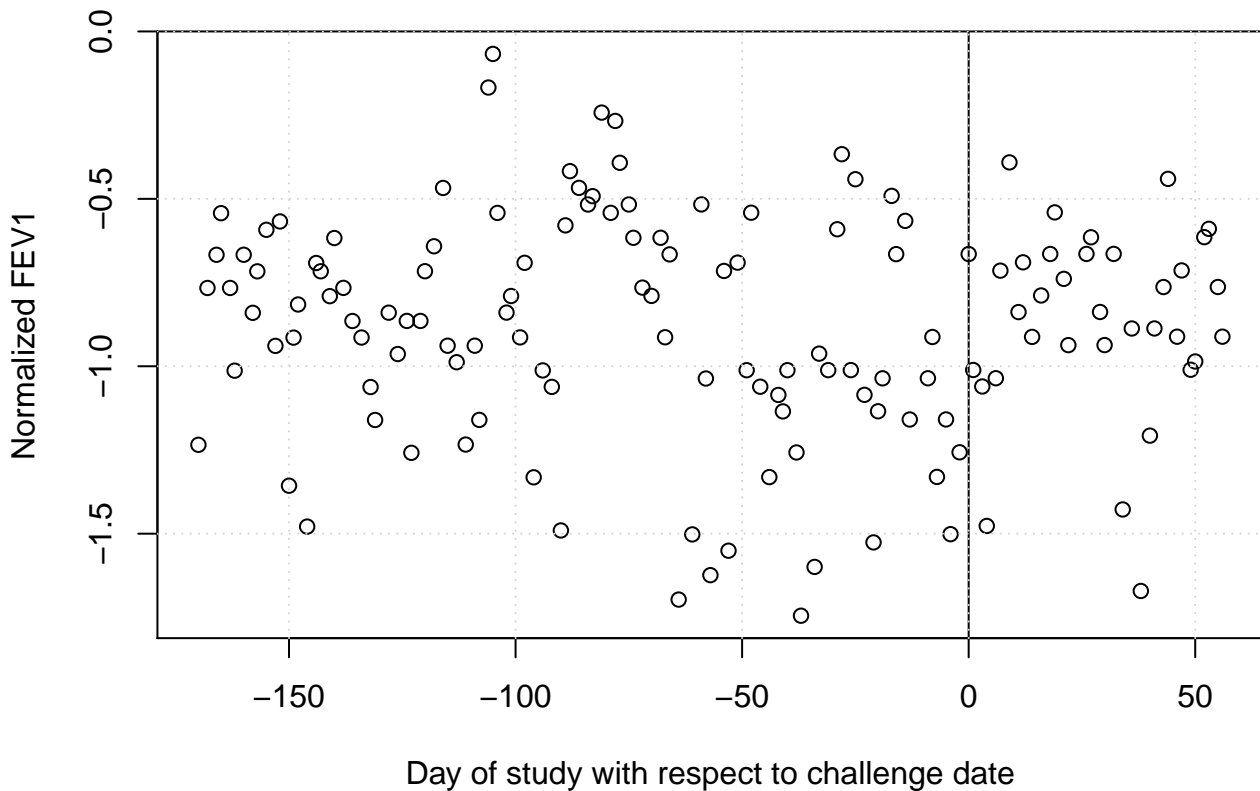

# P05A

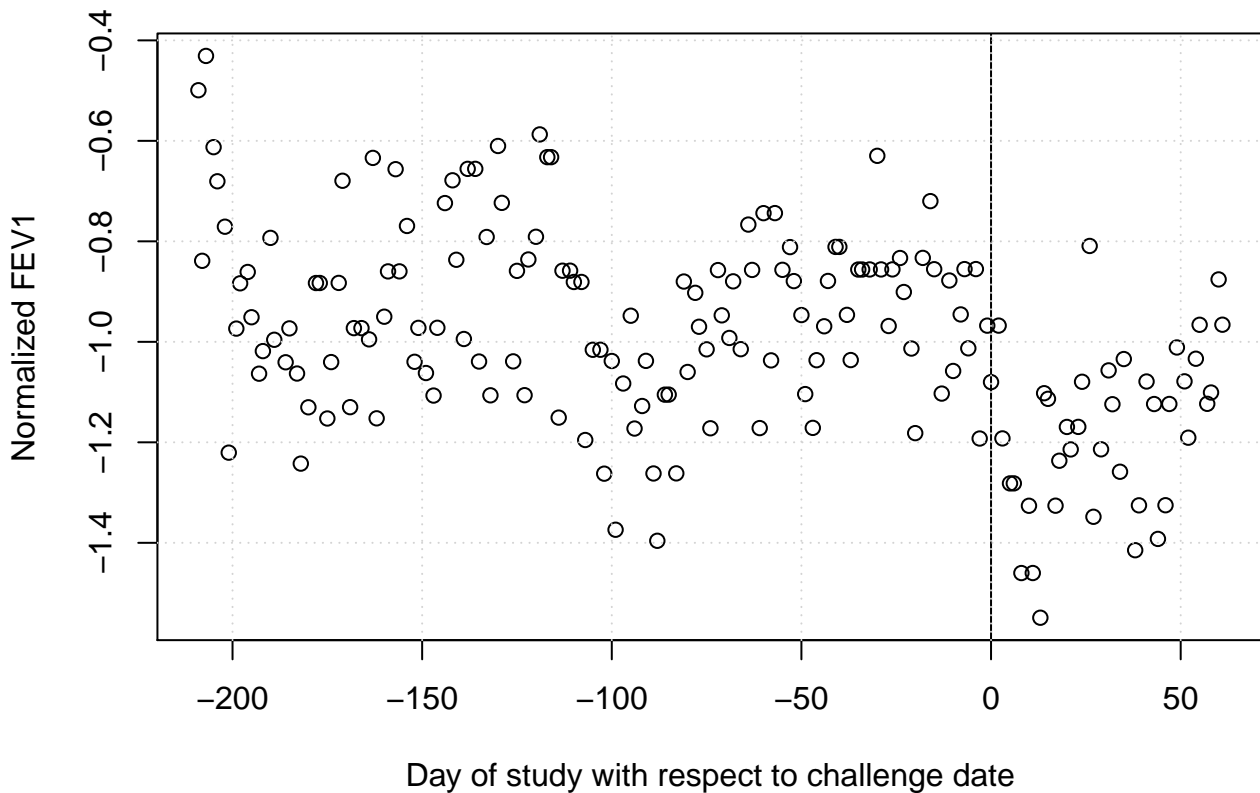

# P06A

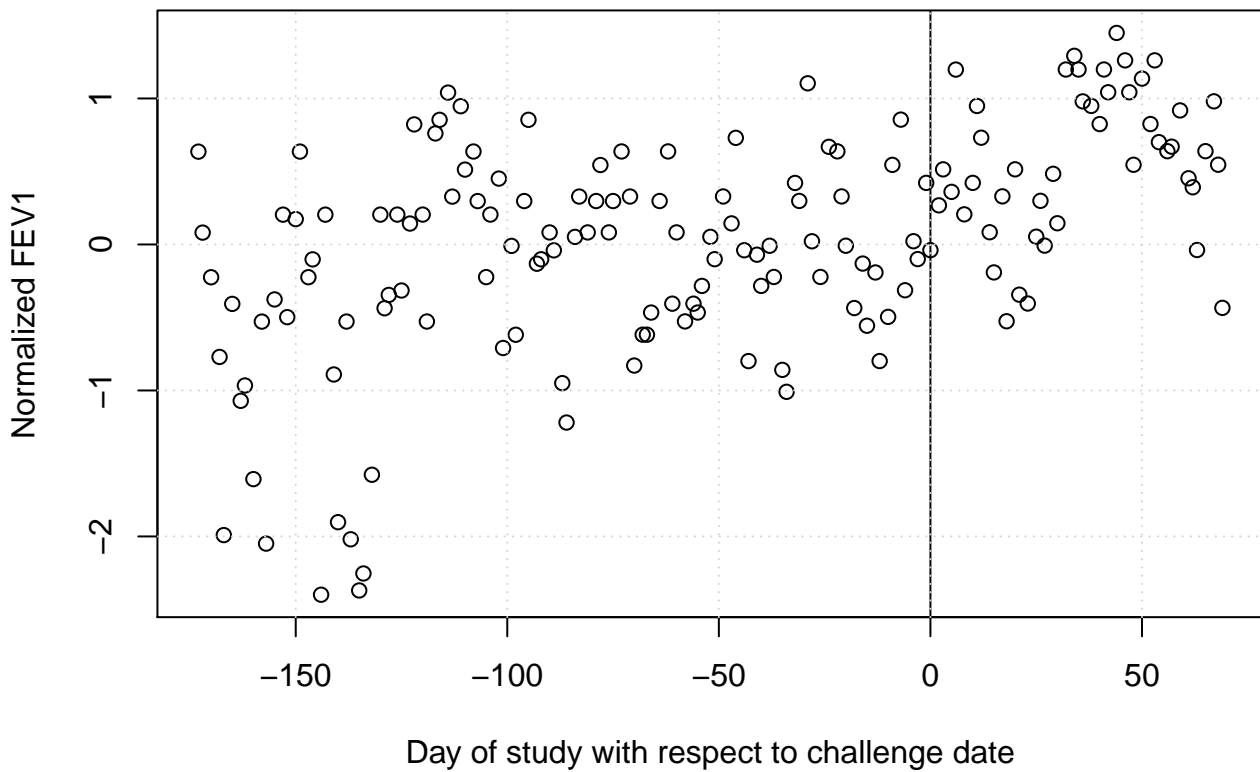

**P07A**

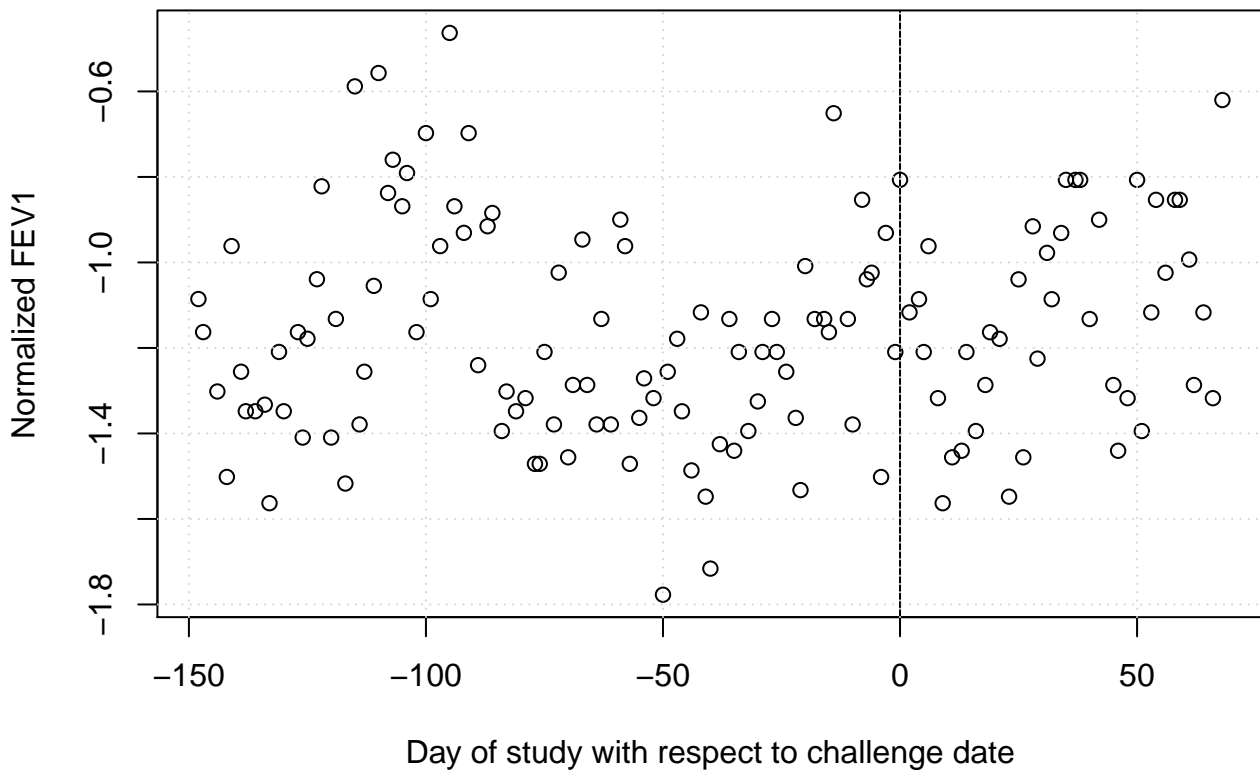

# P08A

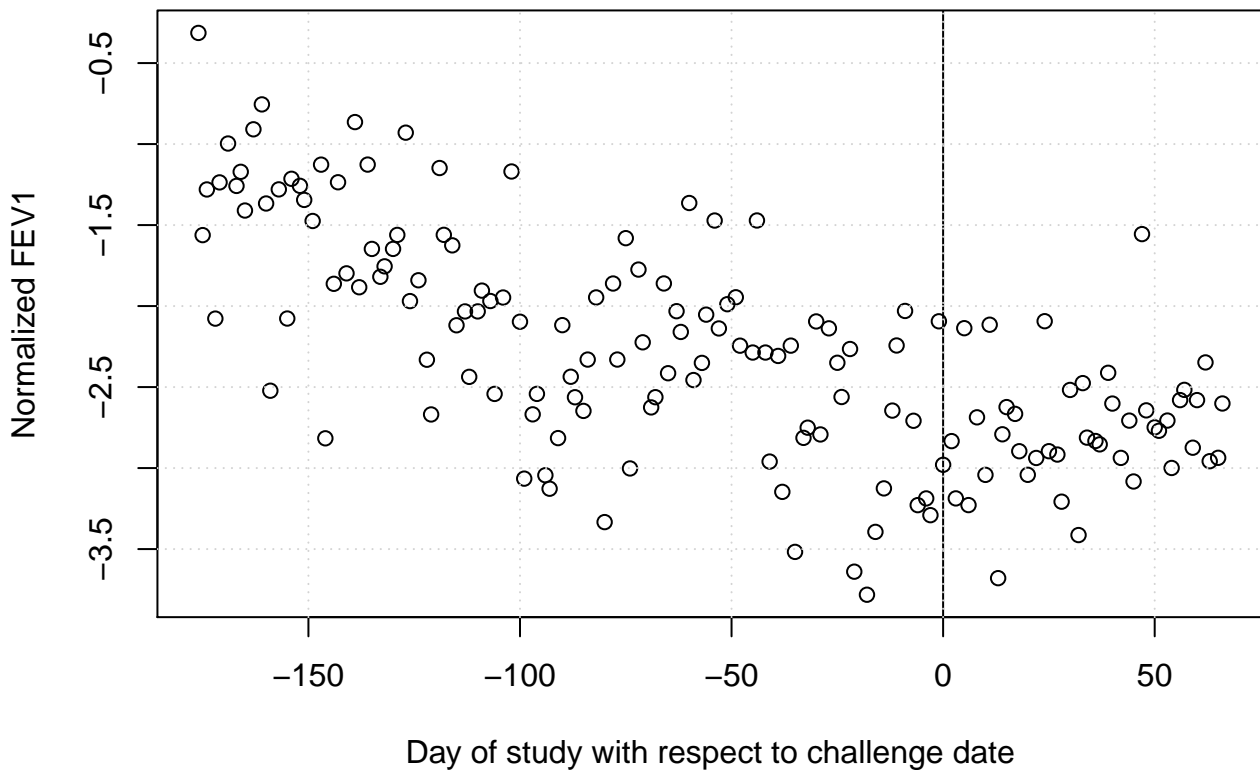

# P09A

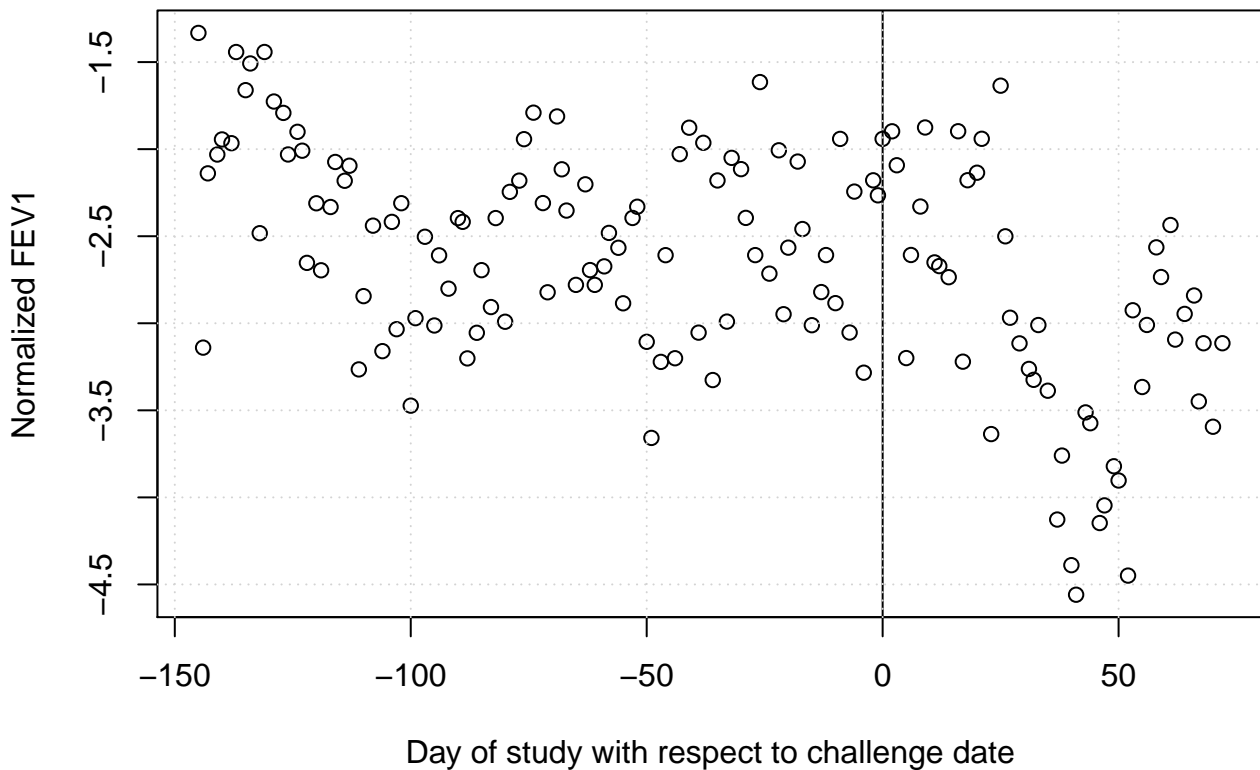

# P10A

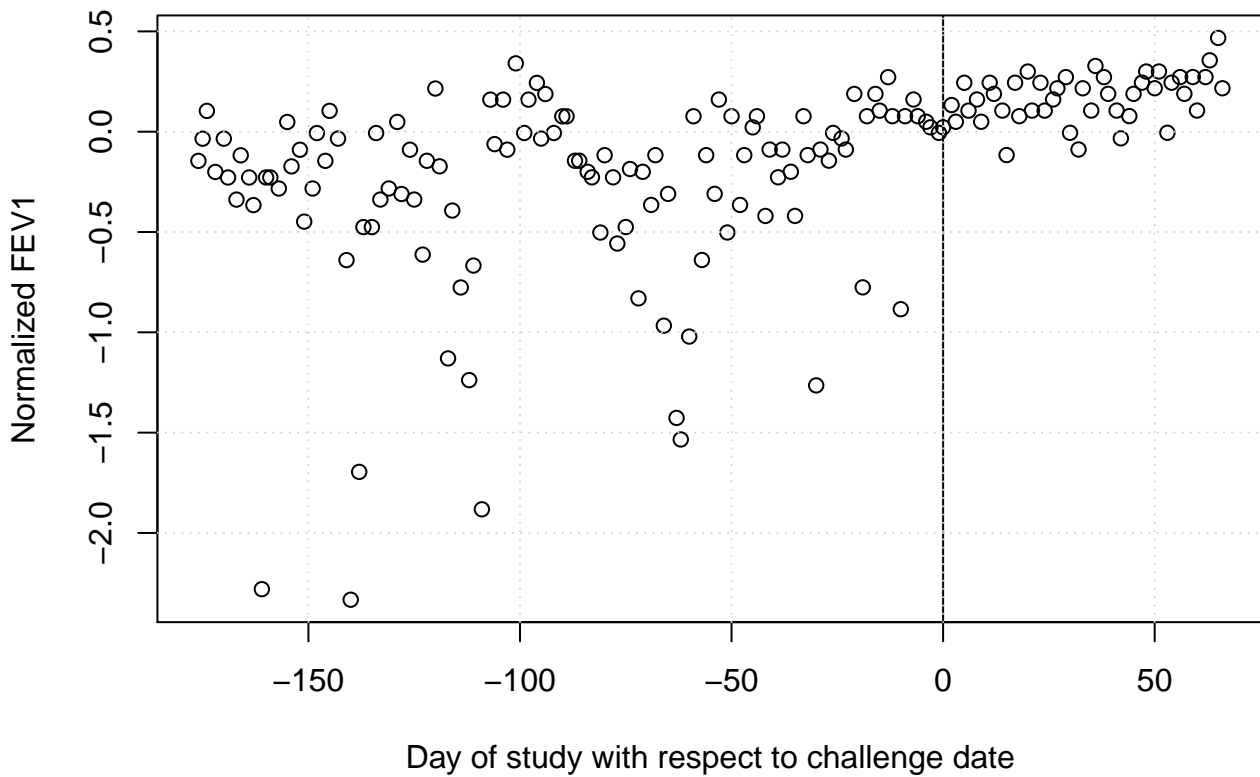

# P11A

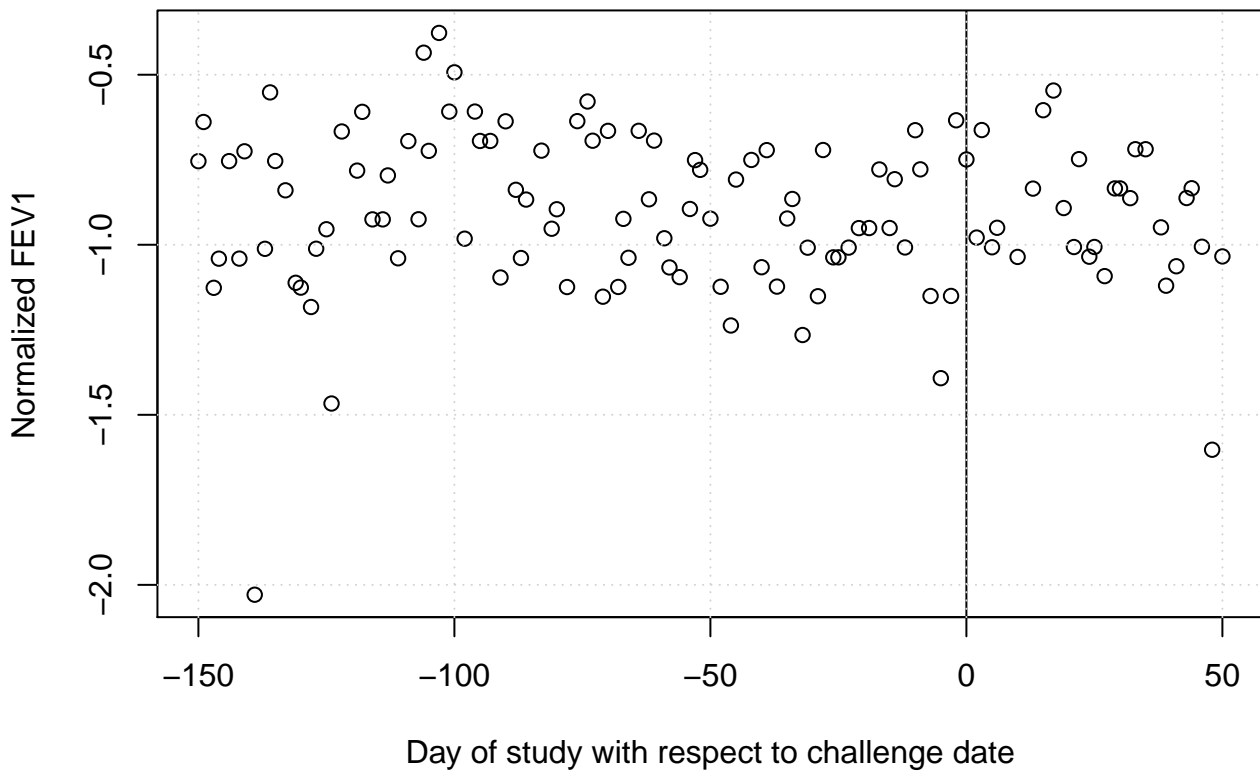

## P12A

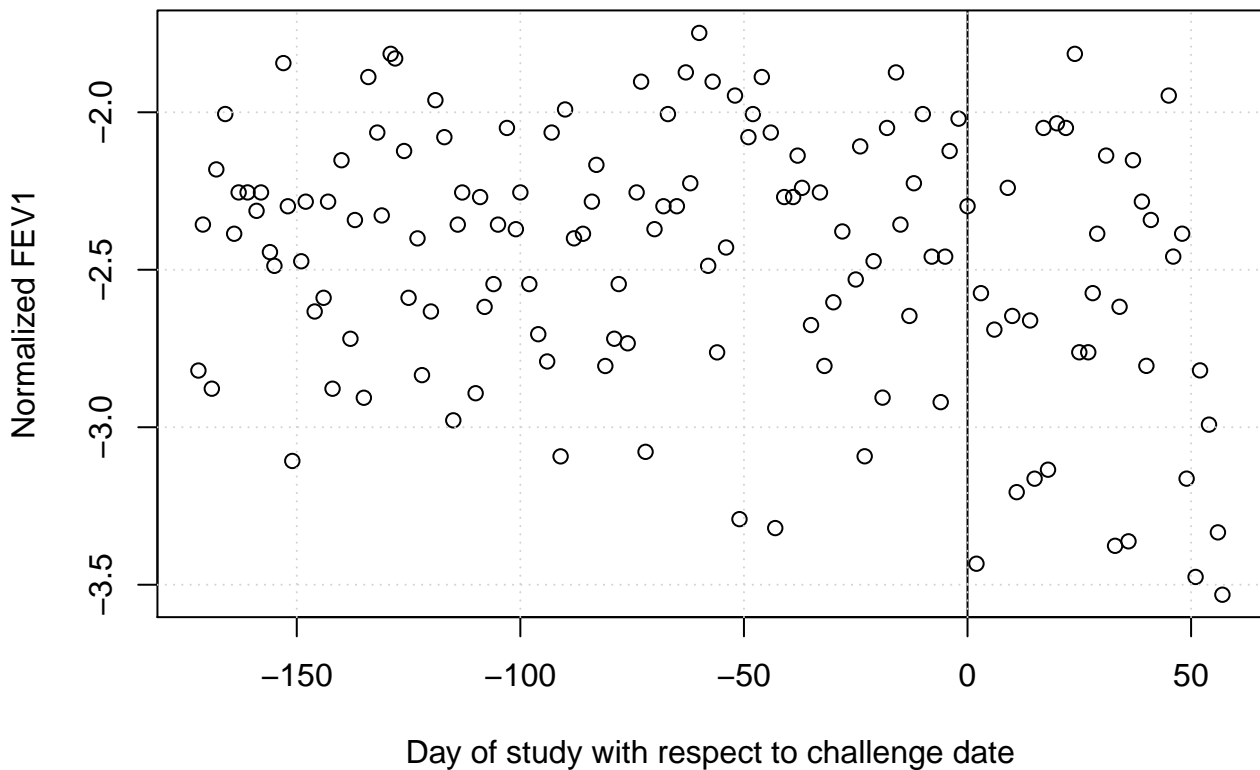

# P13A

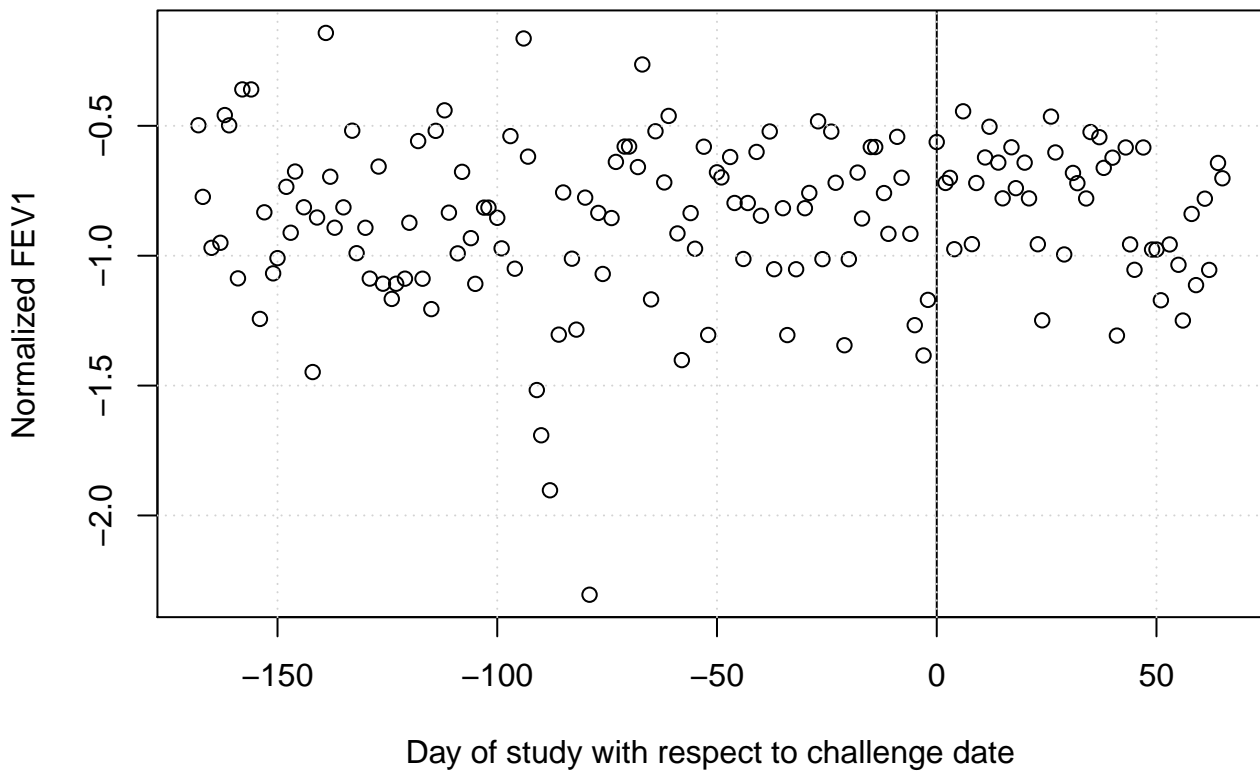

Supplement: Supplementary file 2. [file elife-47969-supp2.zip › TimeSeriesPlots_PDFs/Appendix-figure SS4_NFEV1_TimeSeries_AsthmaticParticipants.pdf]

# P01A

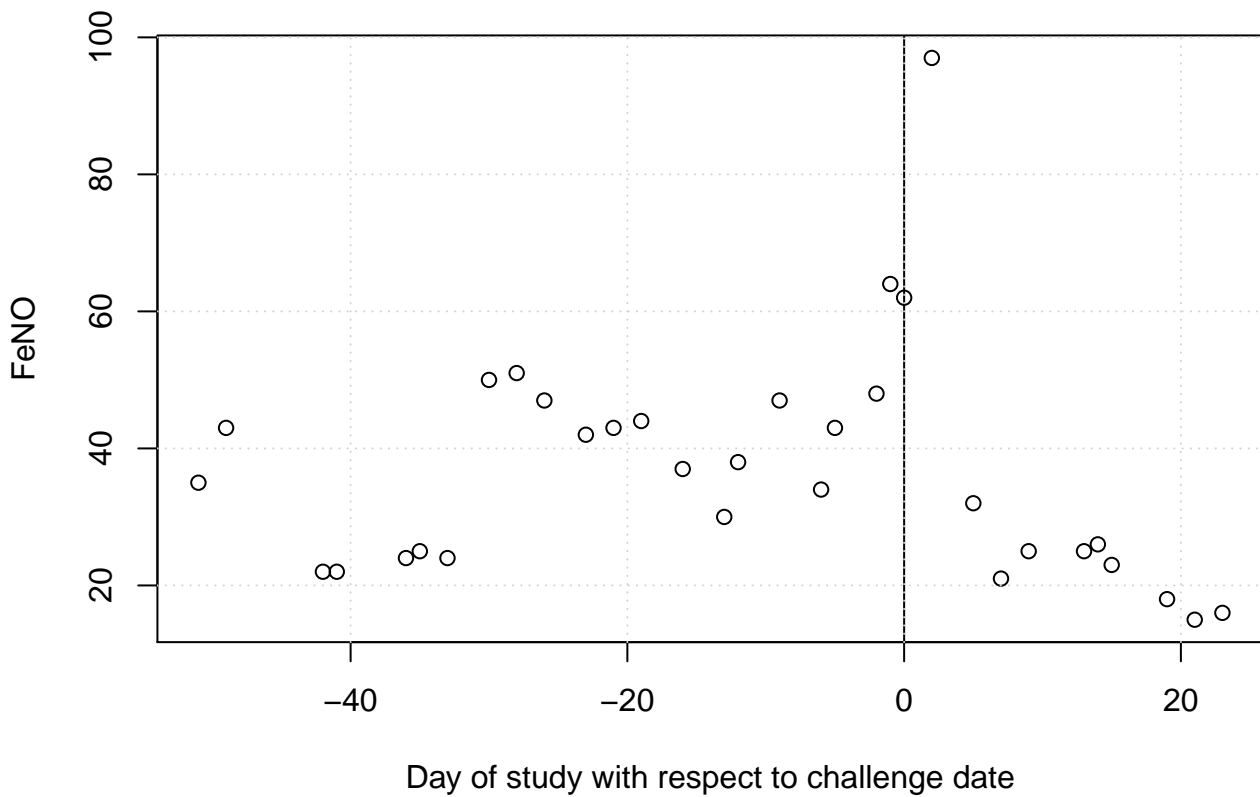

# P02A

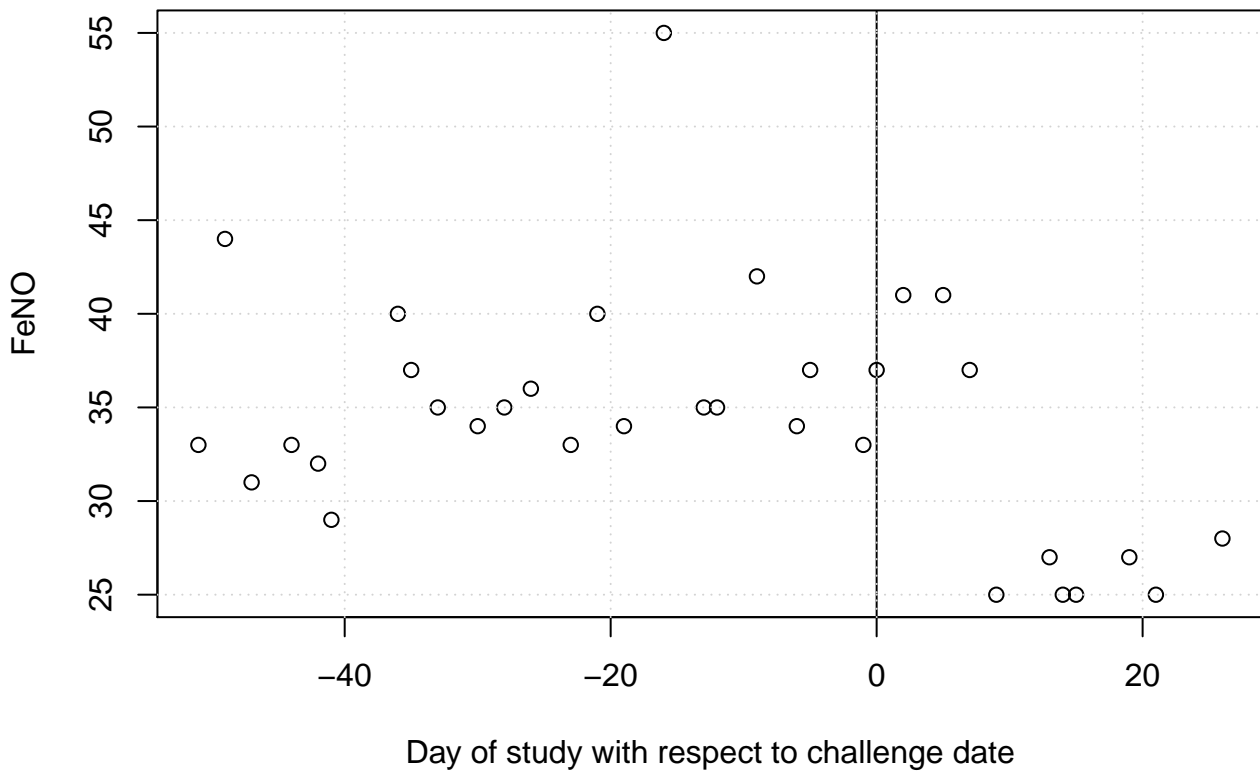

# P04A

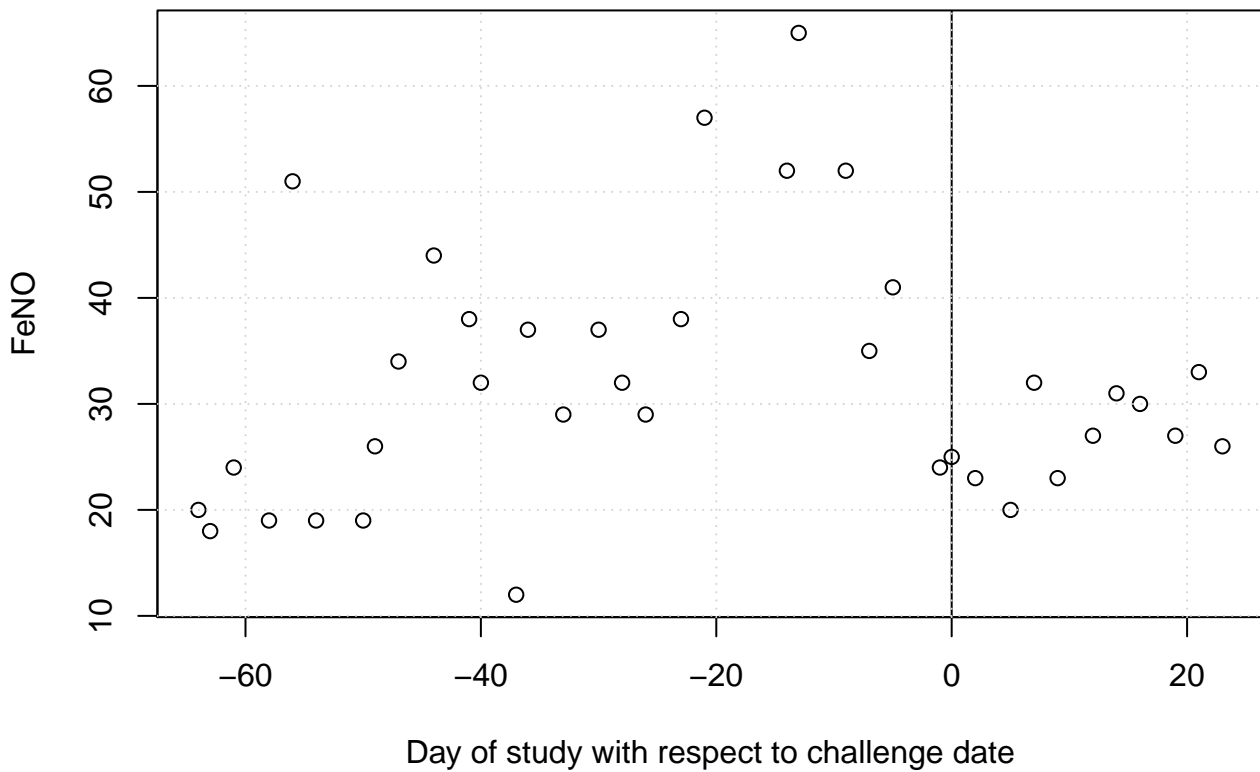

# P05A

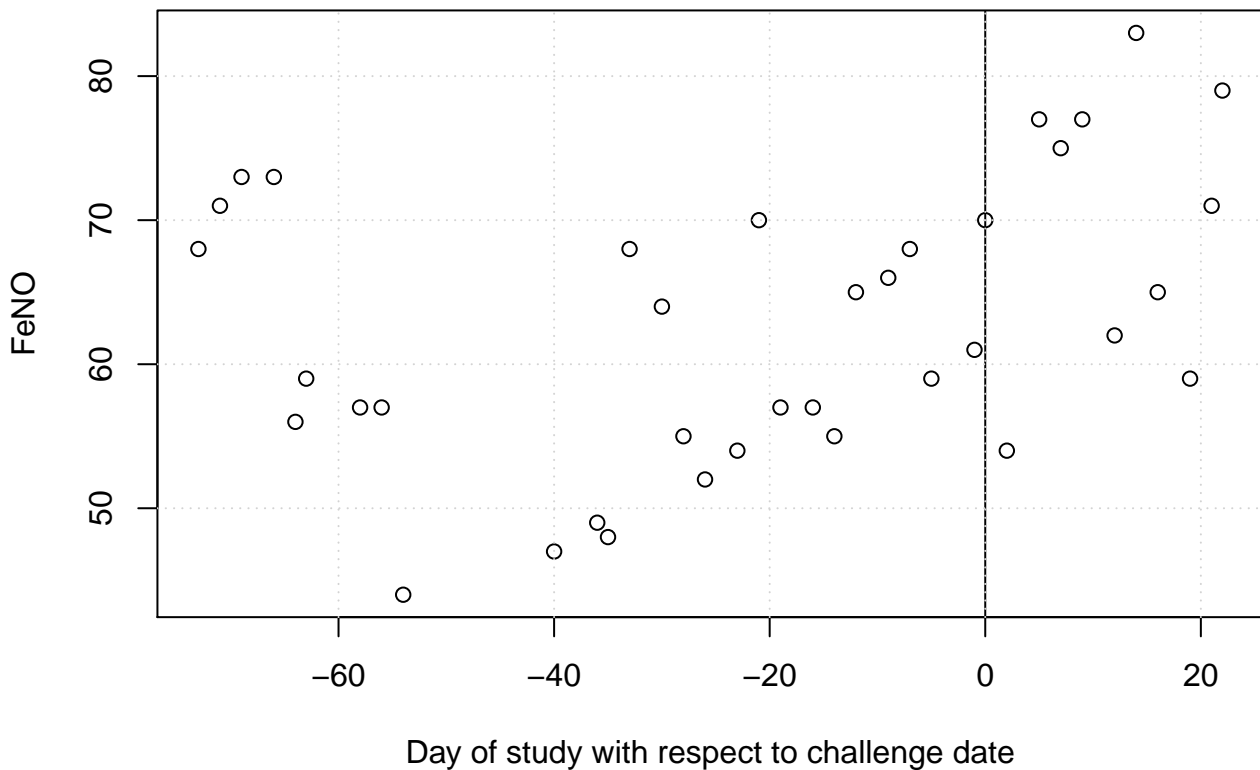

# P06A

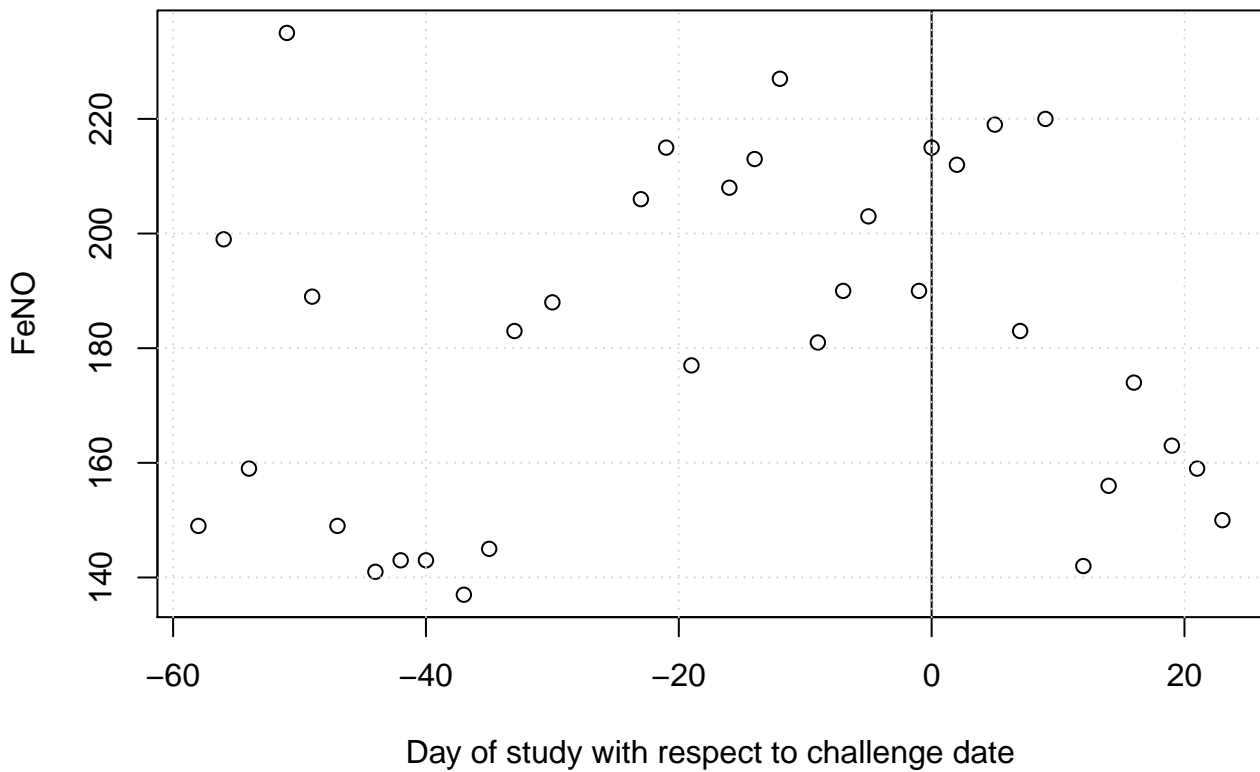

**P07A**

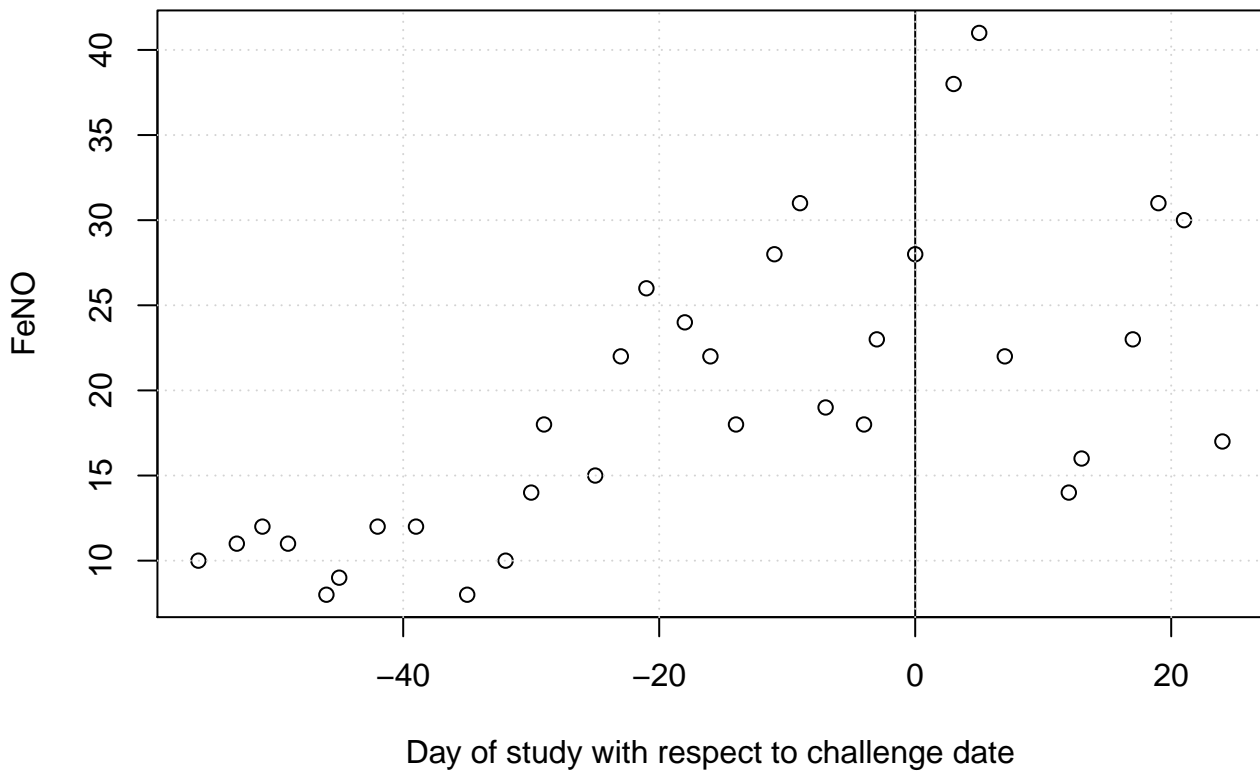

# P08A

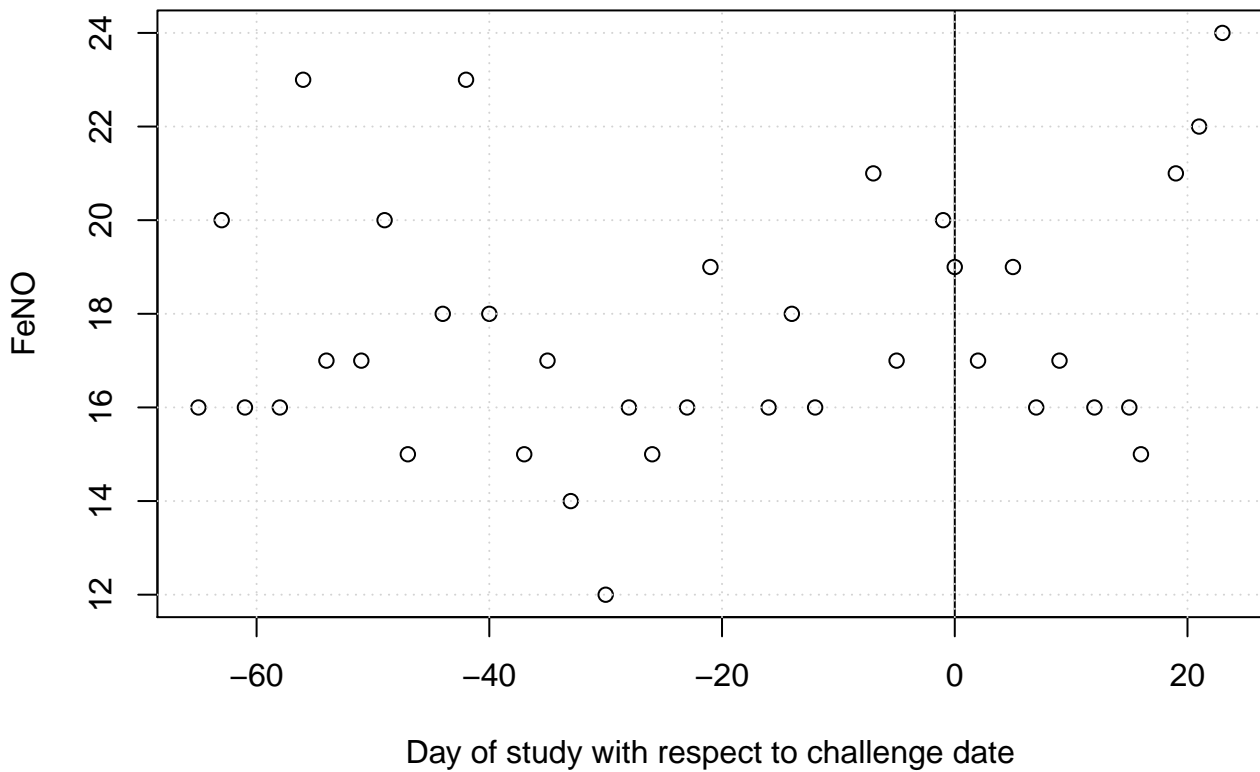

# P09A

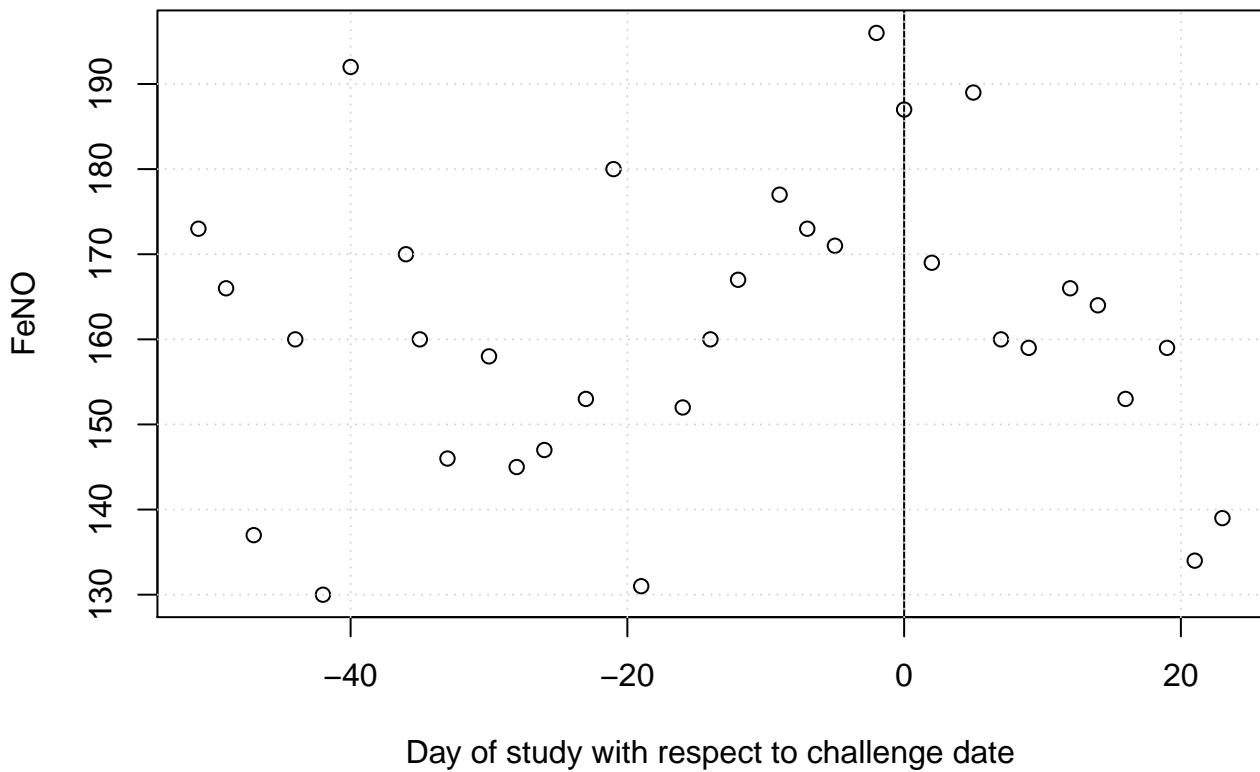

# P10A

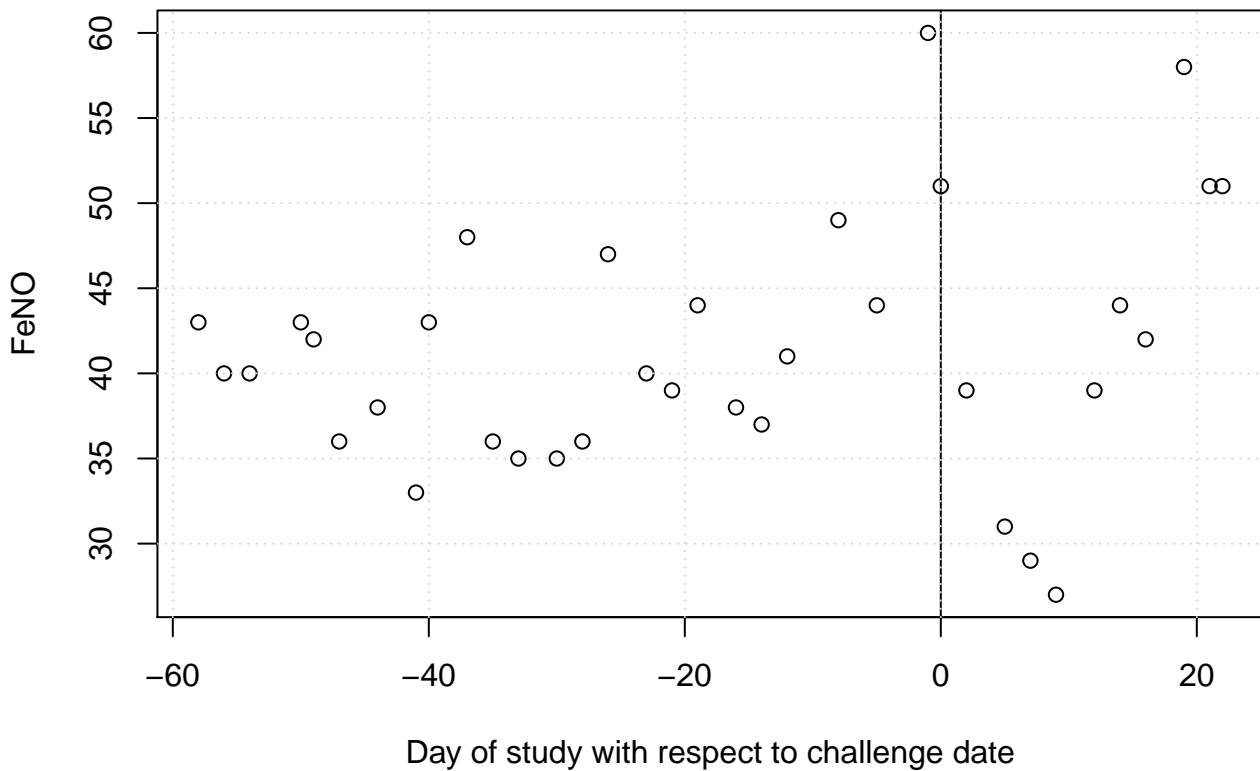

# P11A

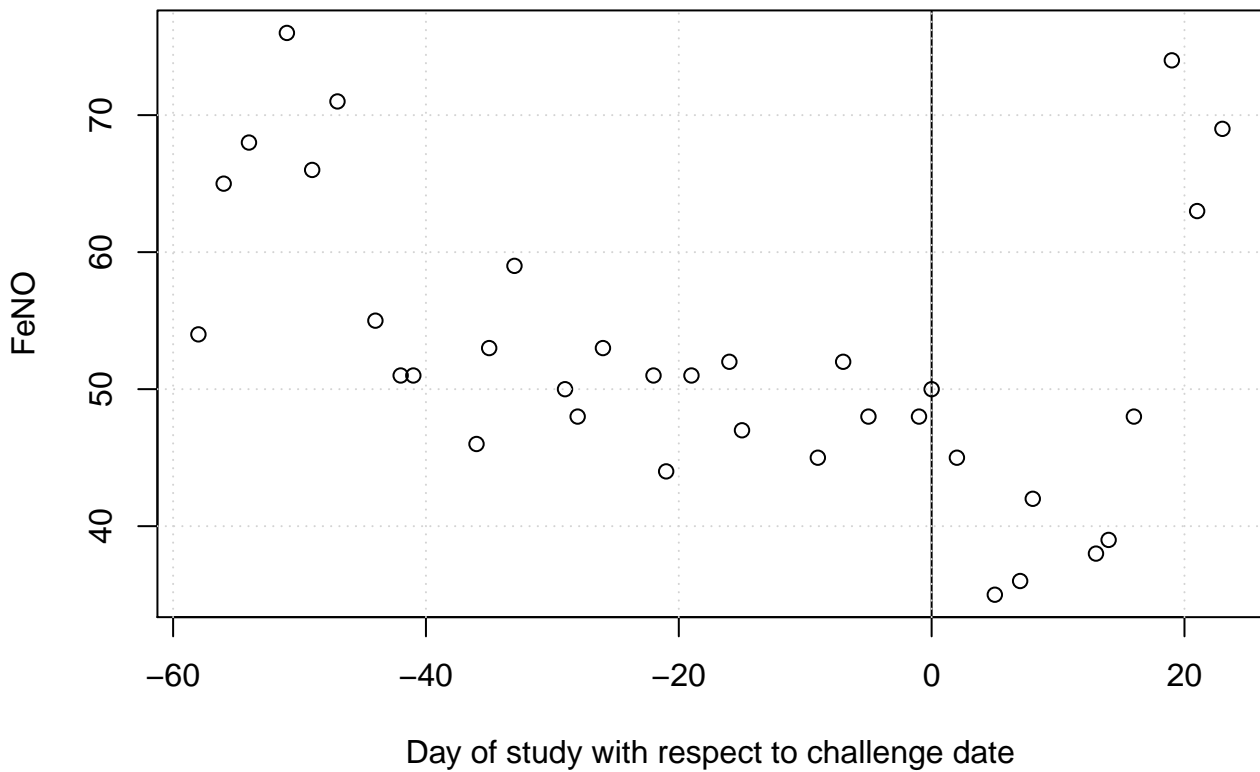

## P12A

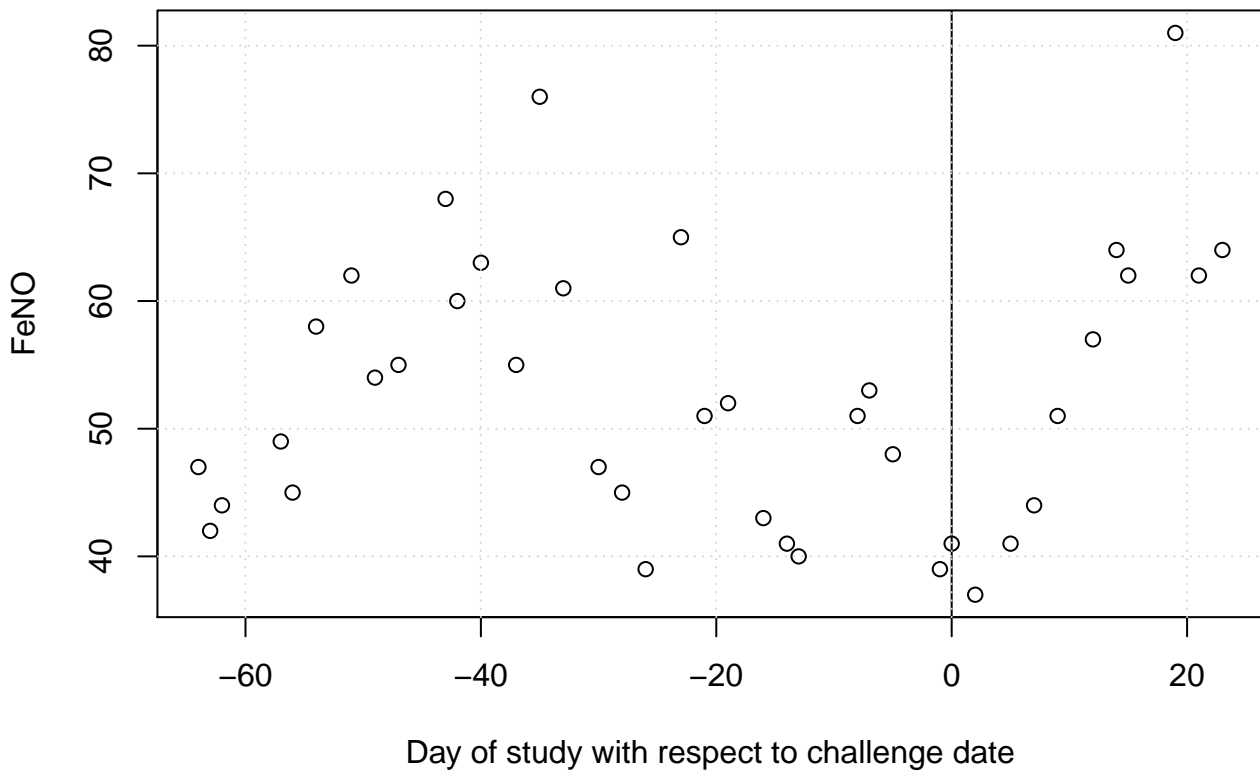

# P13A

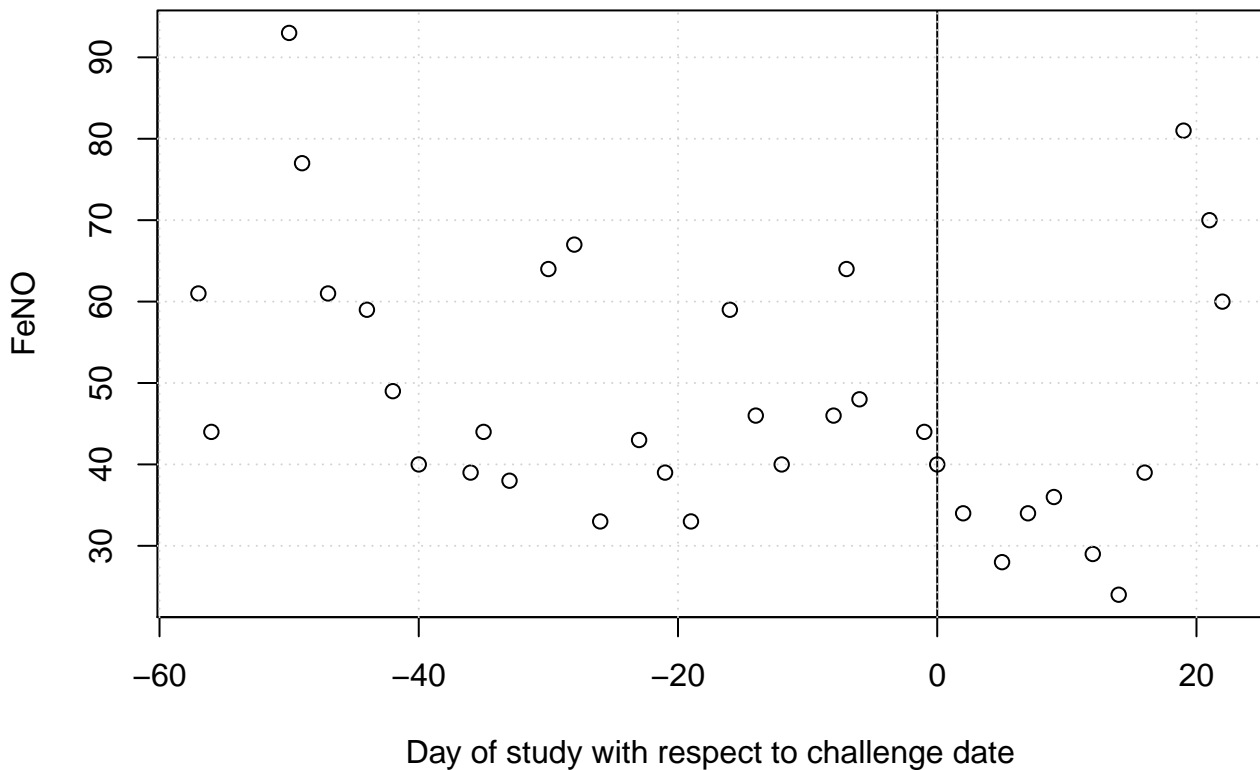

Supplement: Supplementary file 2. [file elife-47969-supp2.zip › TimeSeriesPlots_PDFs/Appendix-figure SS10_FeNO_TimeSeries_AsthmaticParticipants.pdf]

# P01A

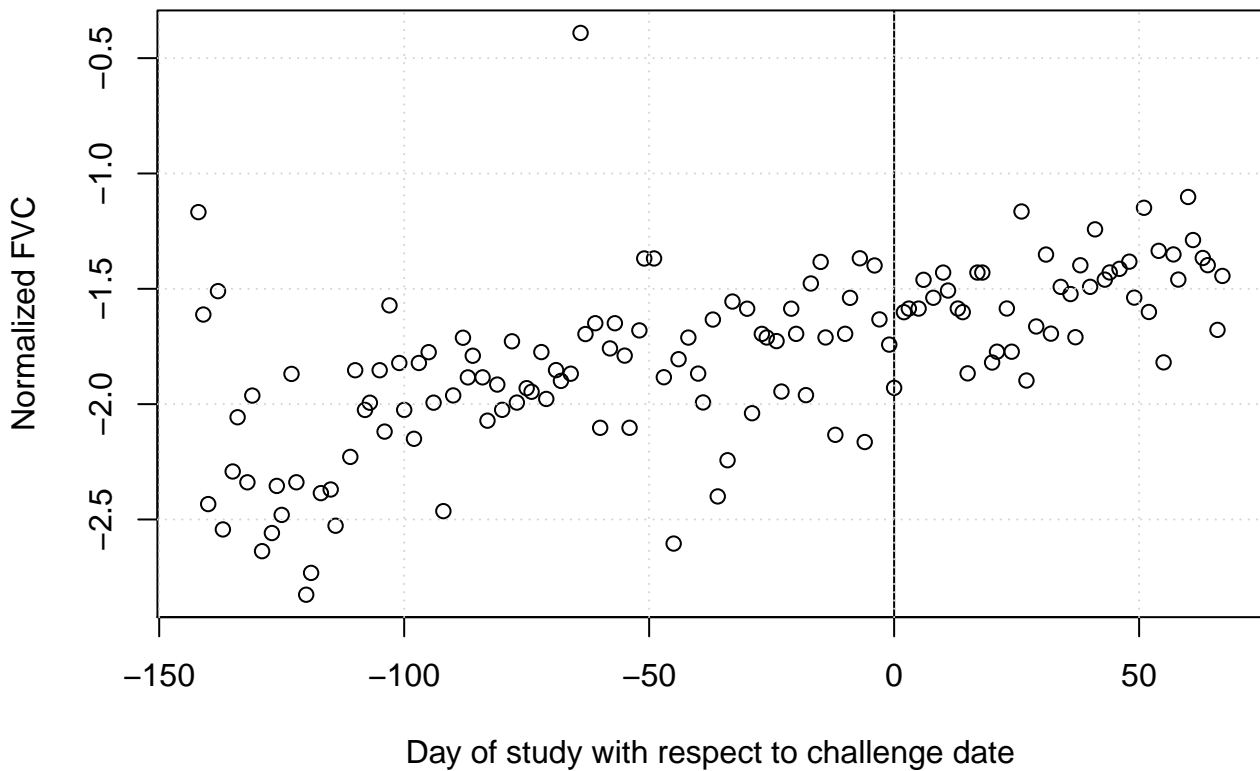

## P02A

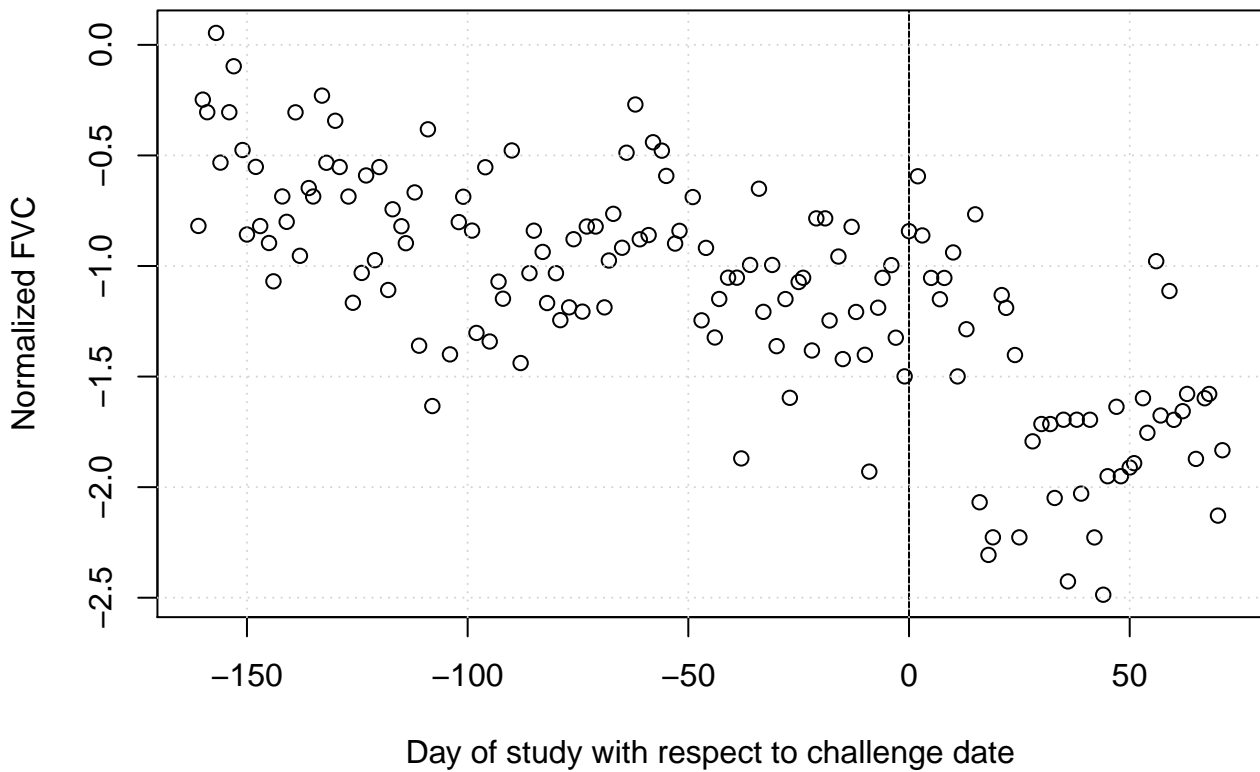

# P04A

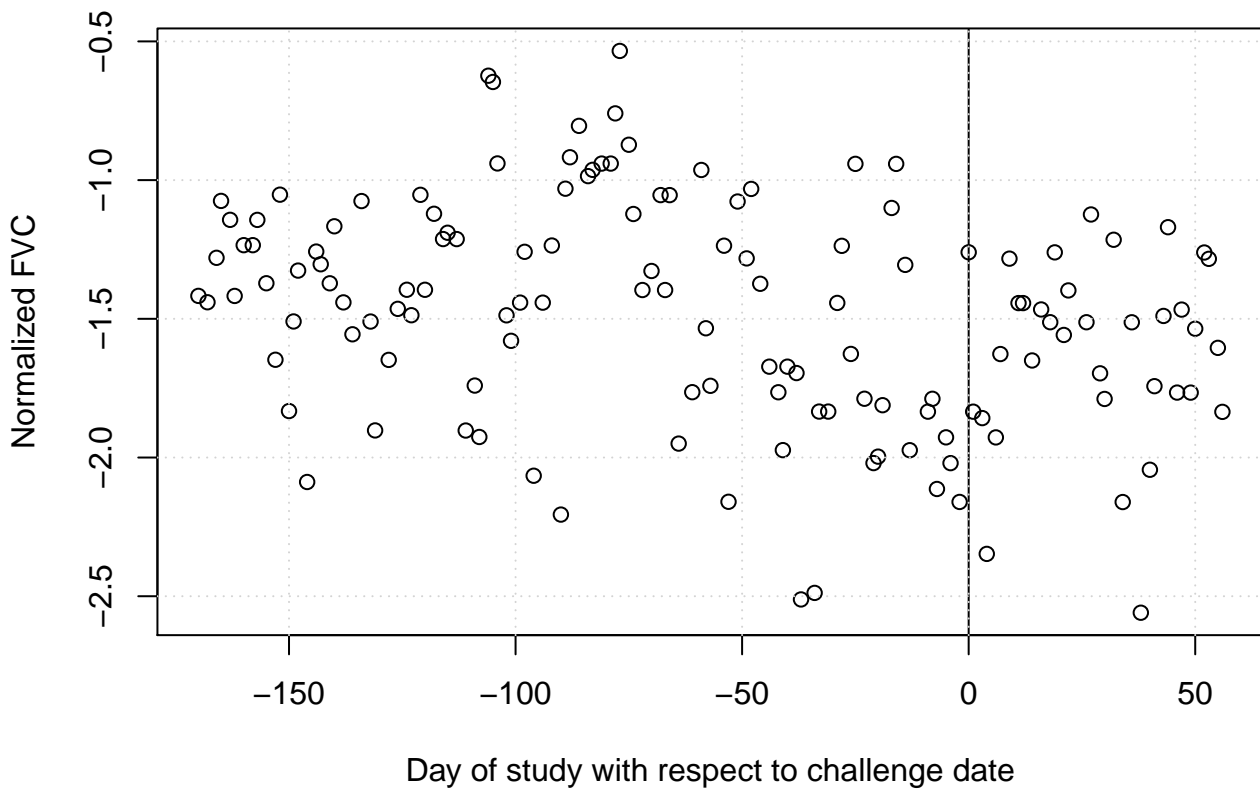

# P05A

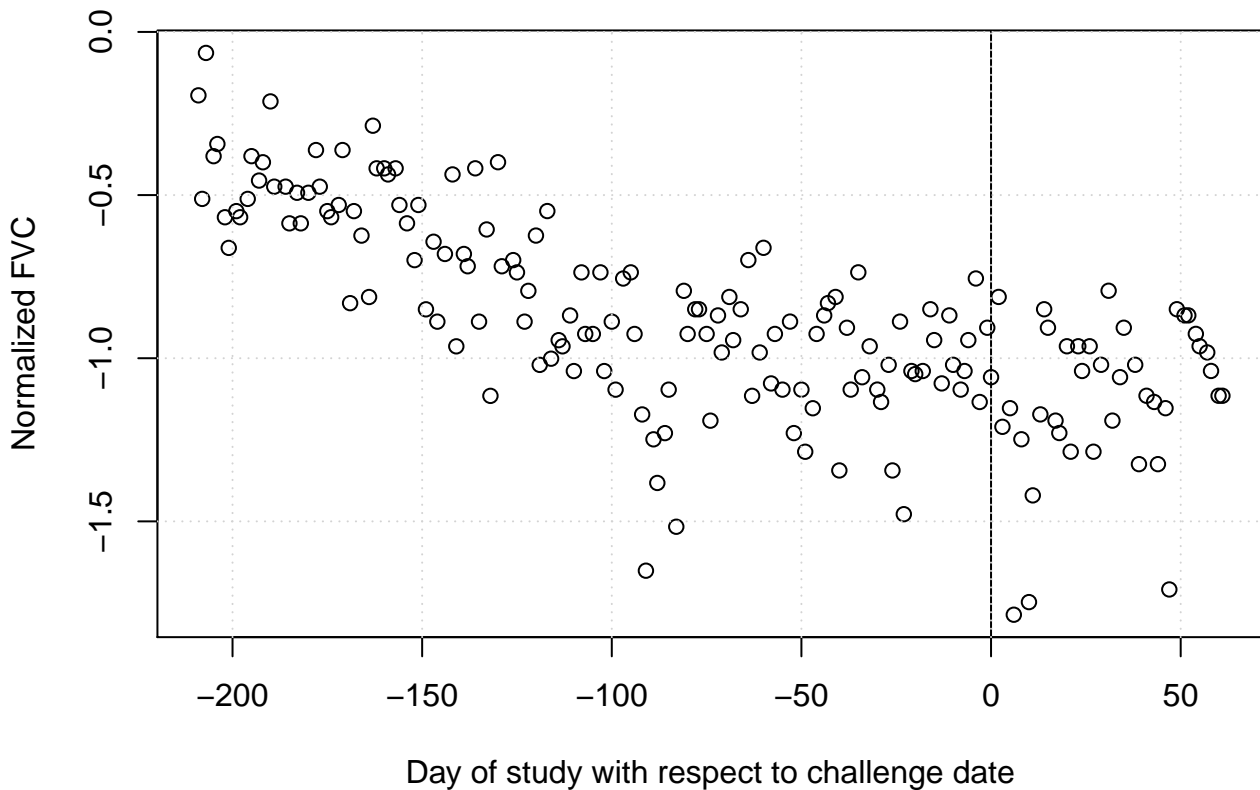

# P06A

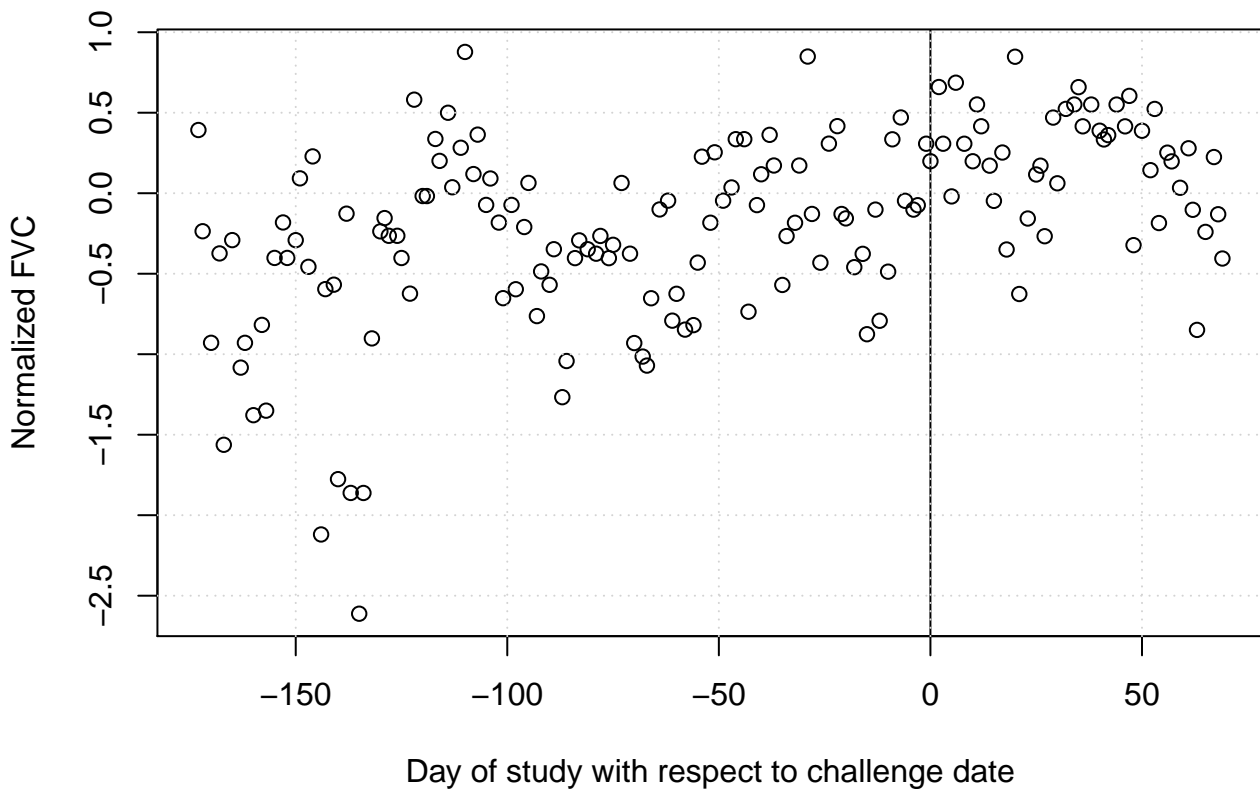

**P07A**

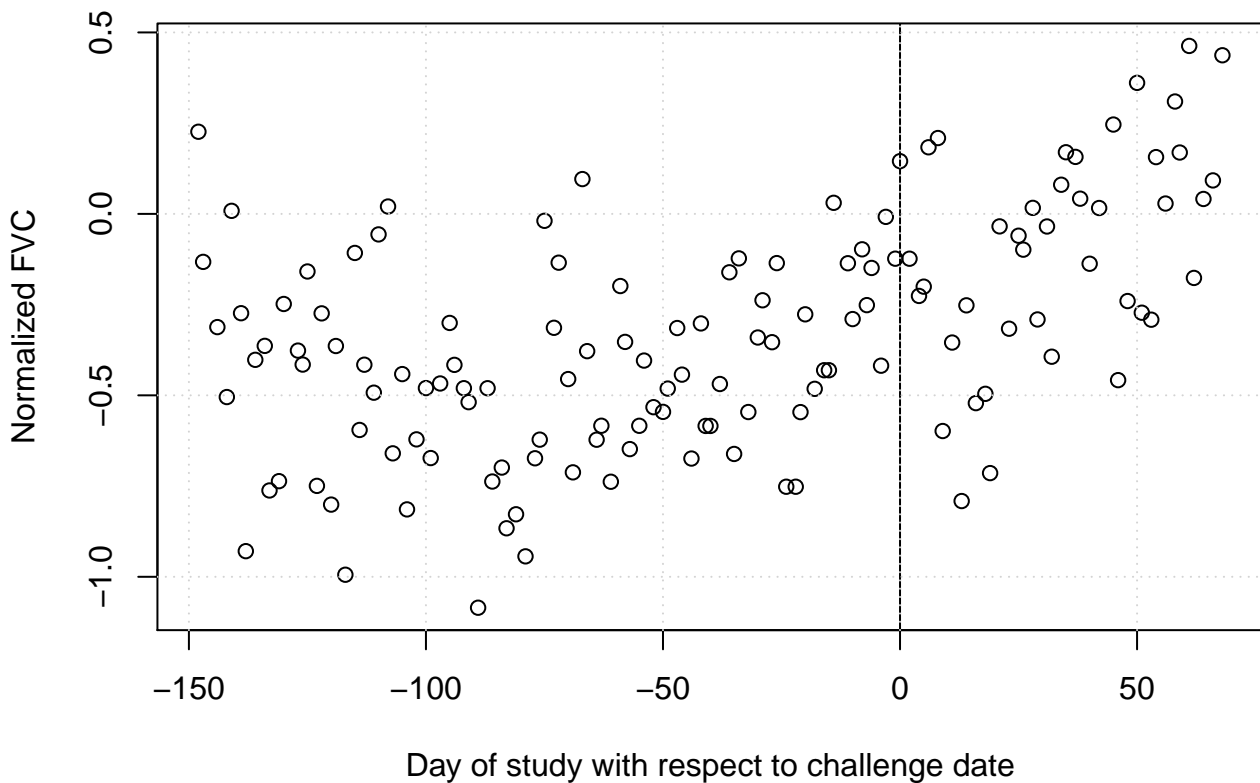

**P08A**

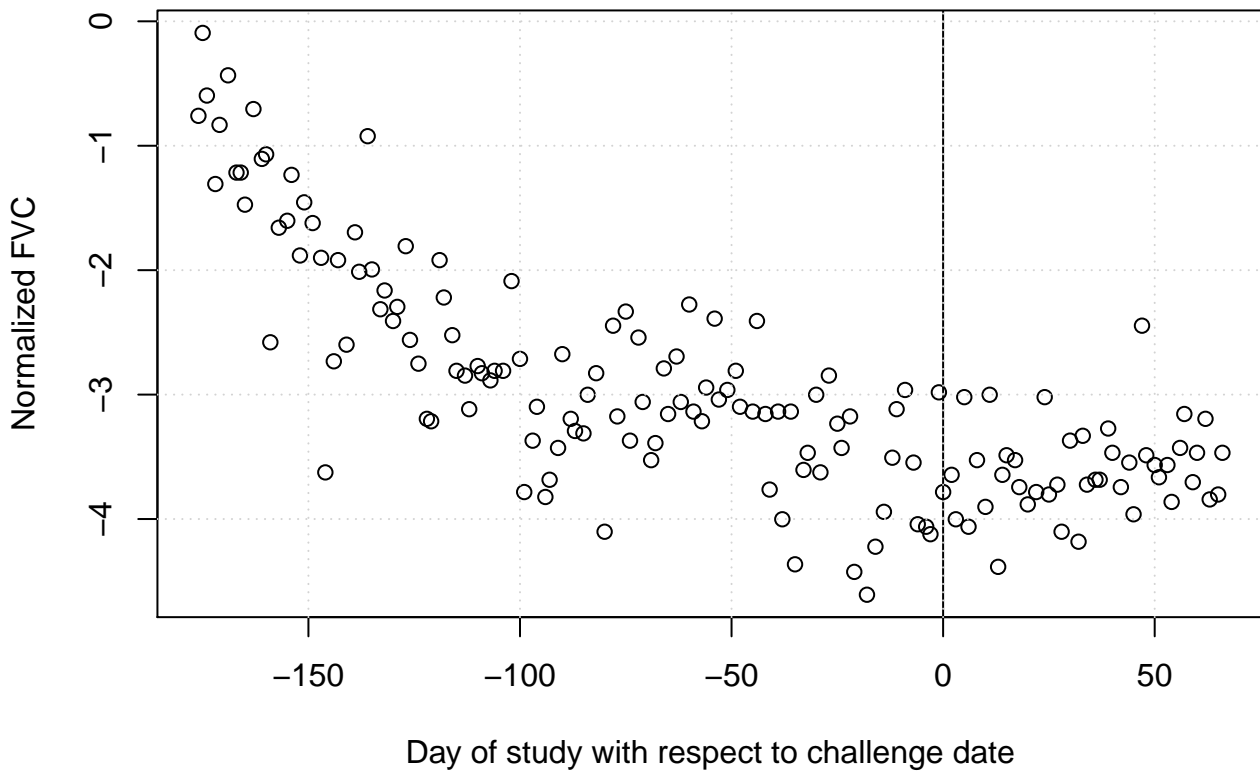

**P09A**

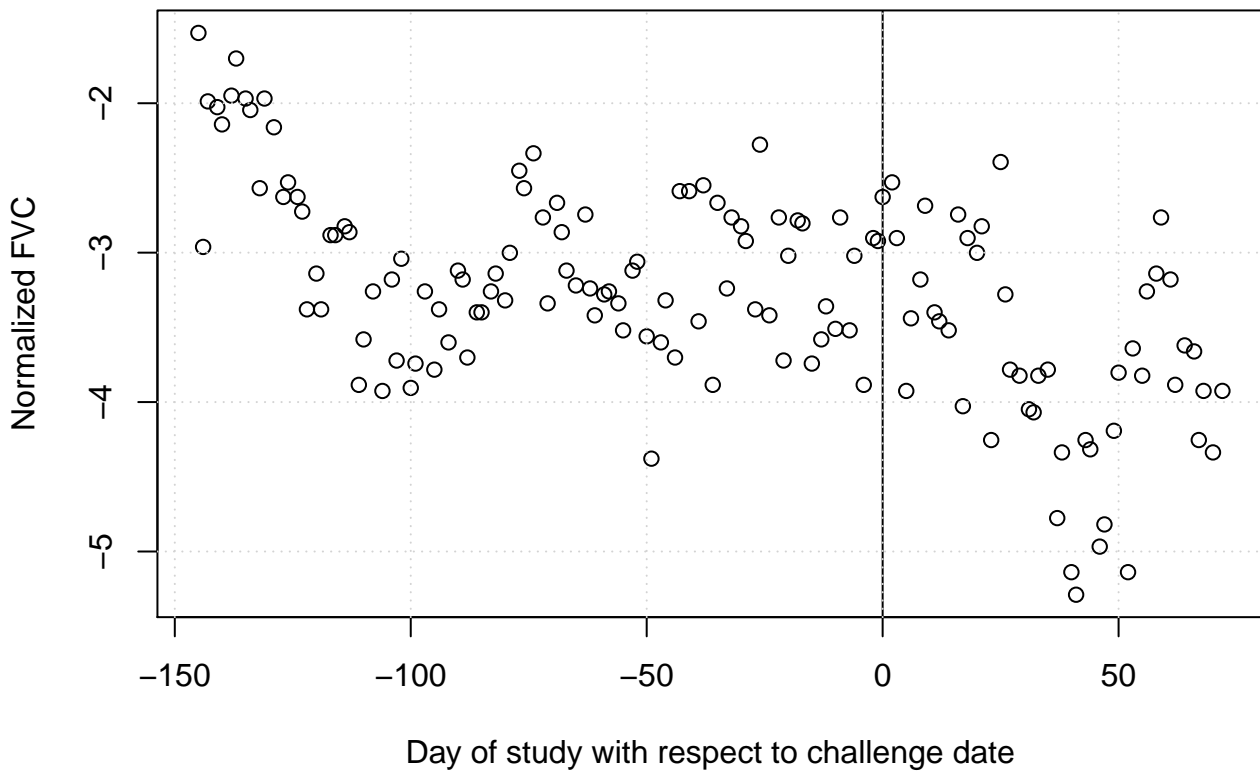

# P10A

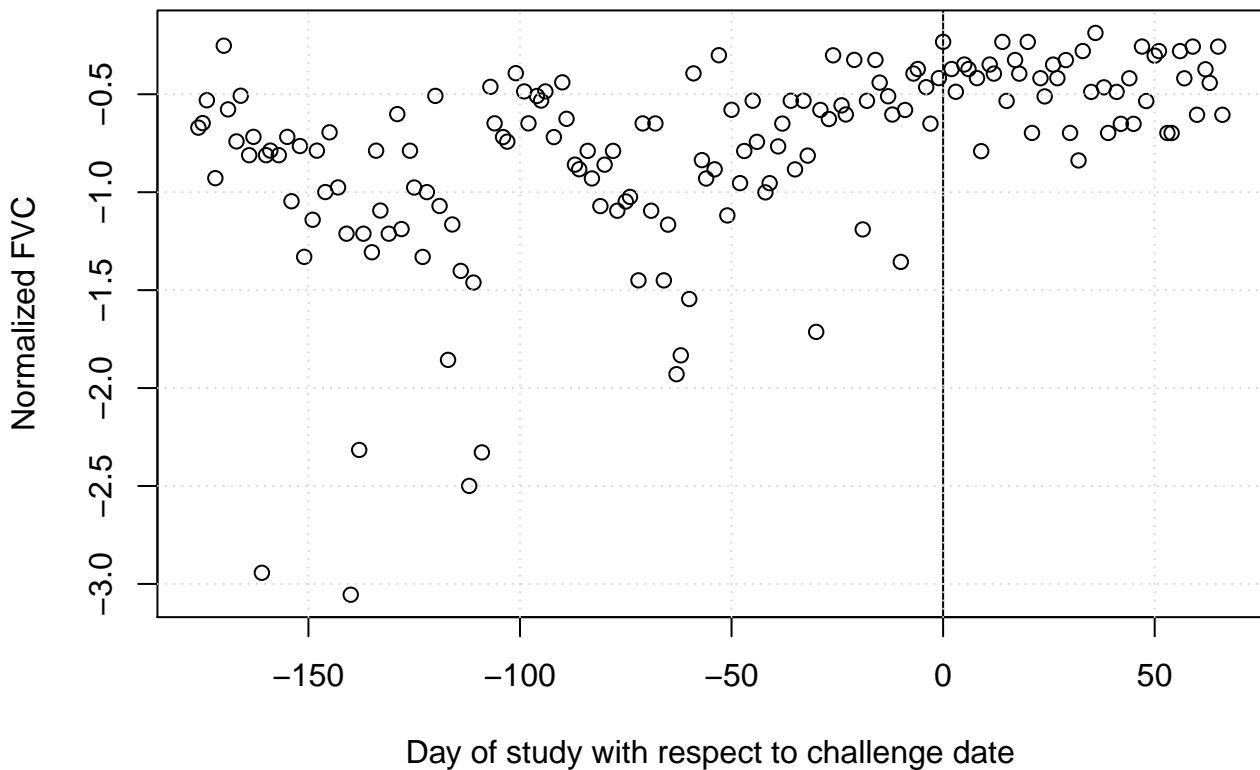

# P11A

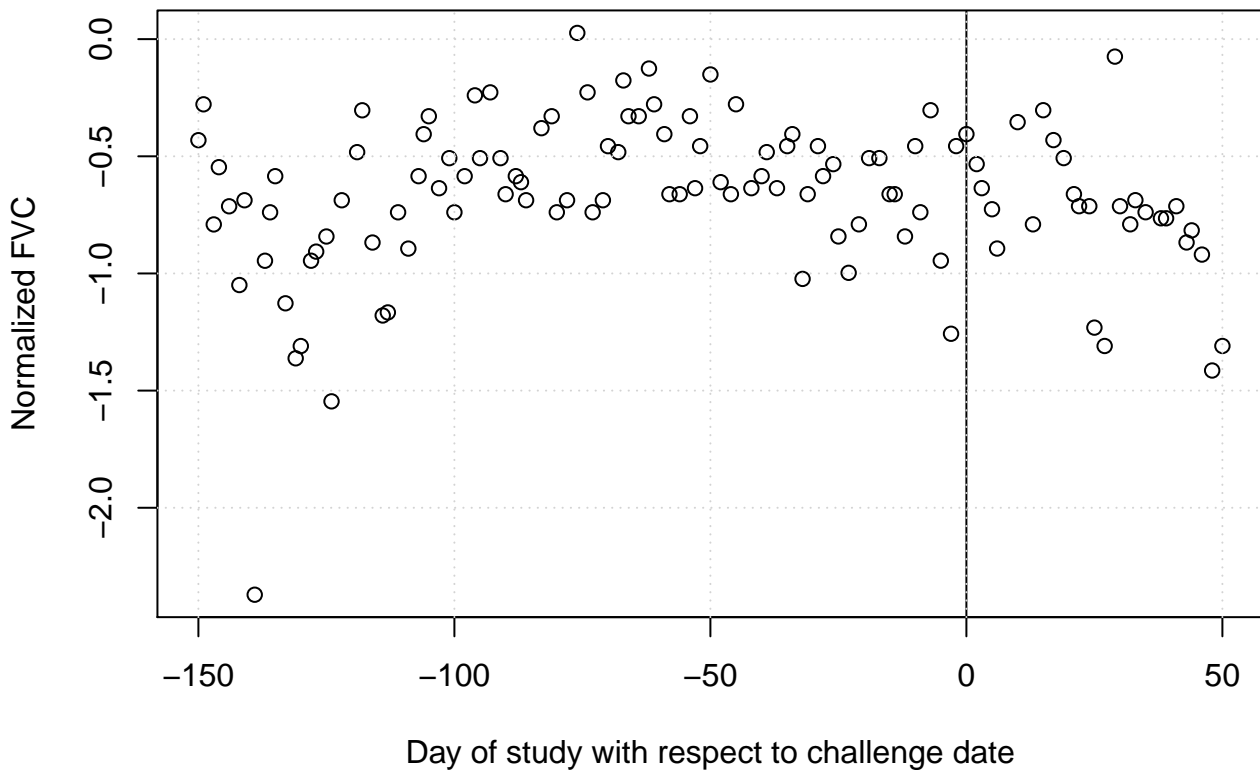

## P12A

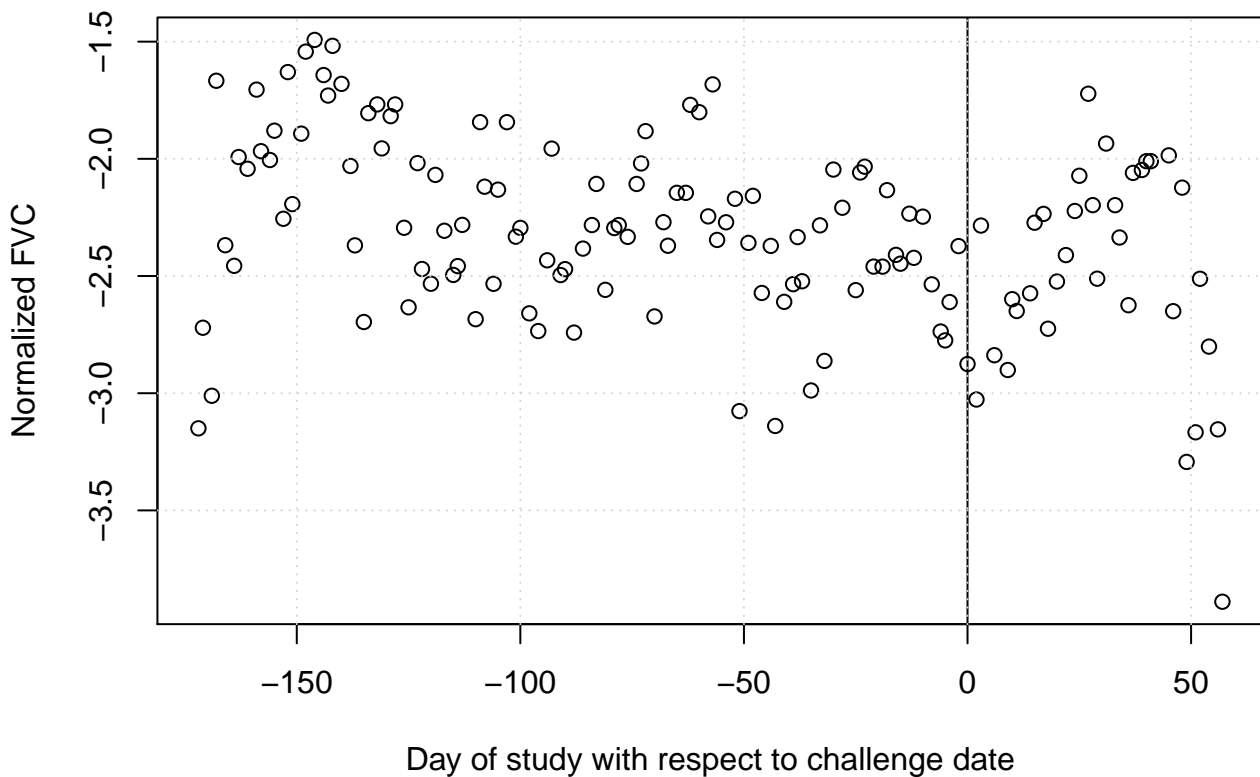

# P13A

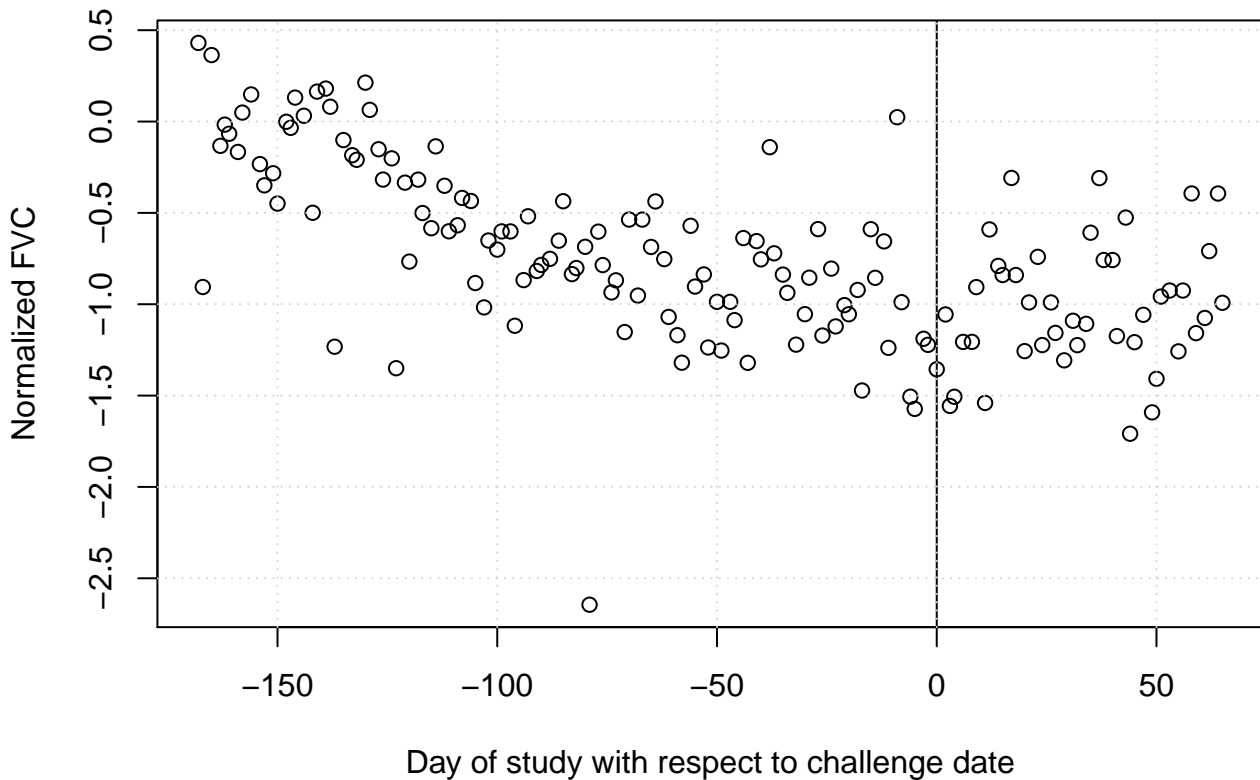

Supplement: Supplementary file 2. [file elife-47969-supp2.zip › TimeSeriesPlots_PDFs/Appendix-figure SS6_NFVC_TimeSeries_AsthmaticParticipants.pdf]

# P01A

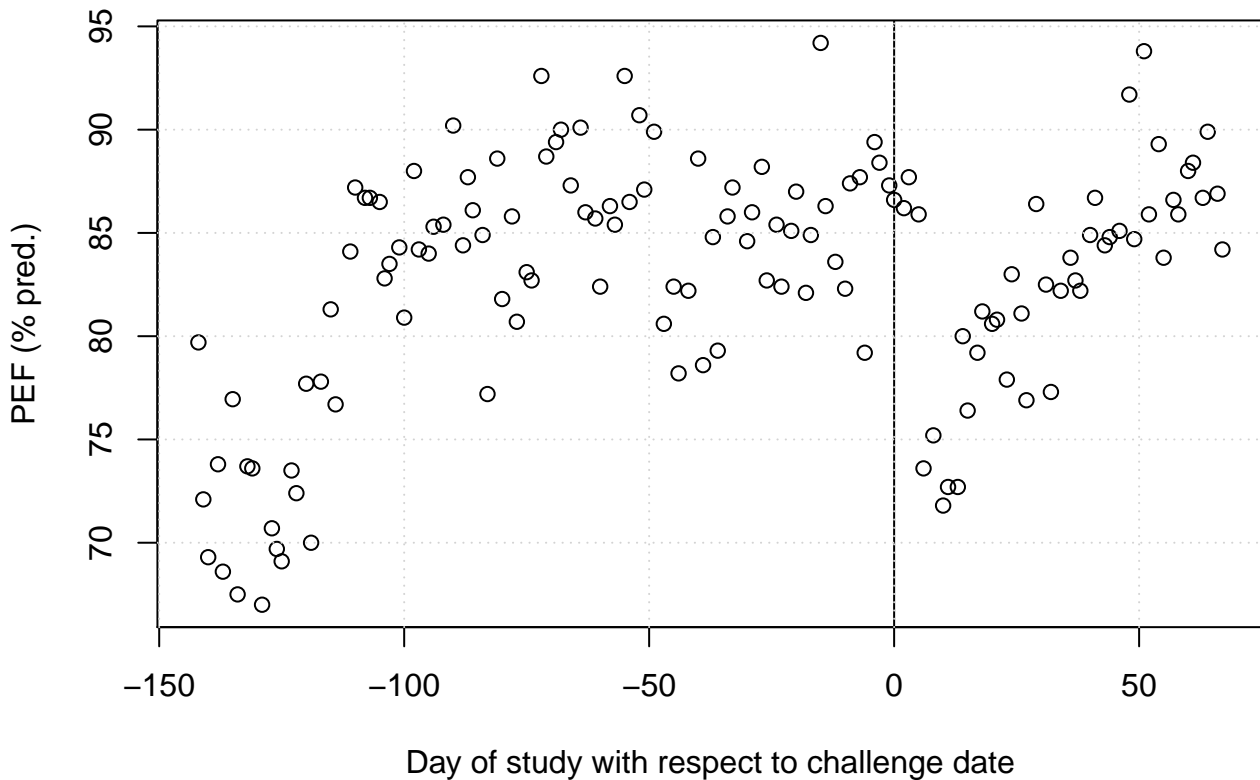

# P02A

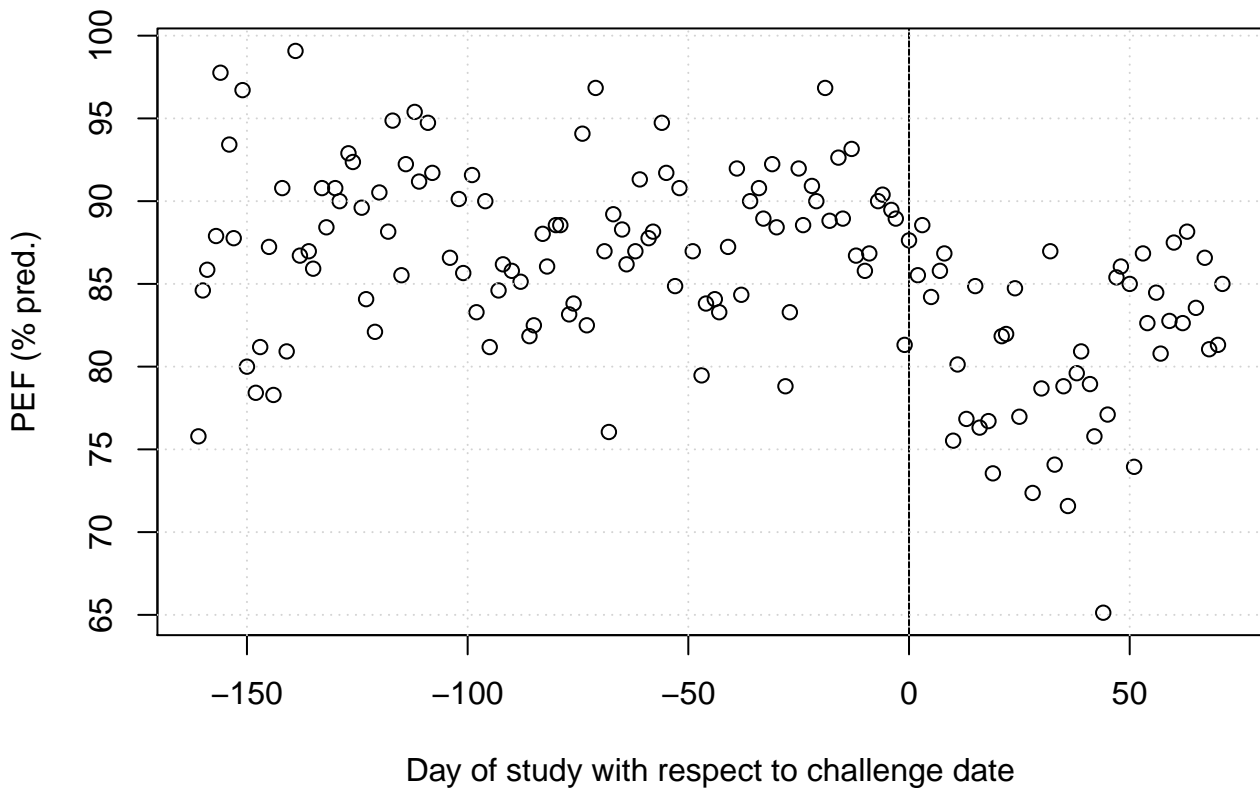

# P04A

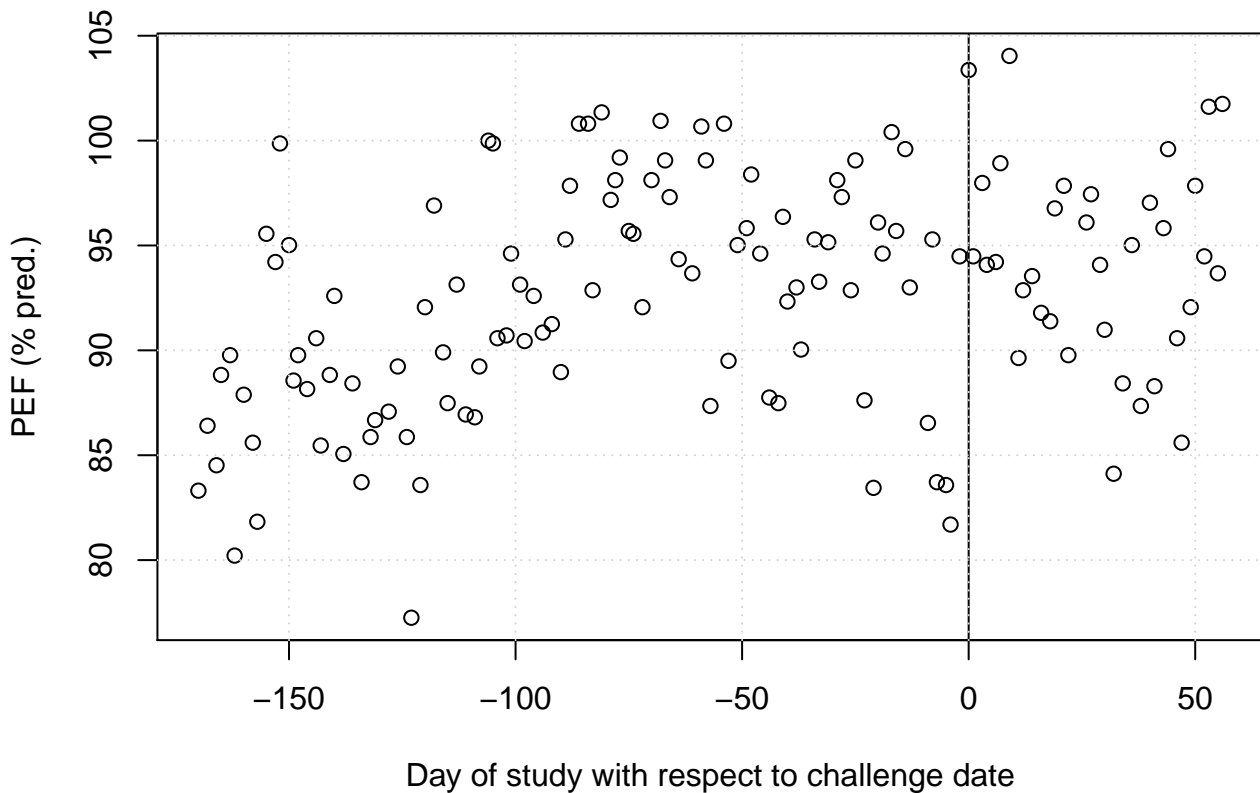

# P05A

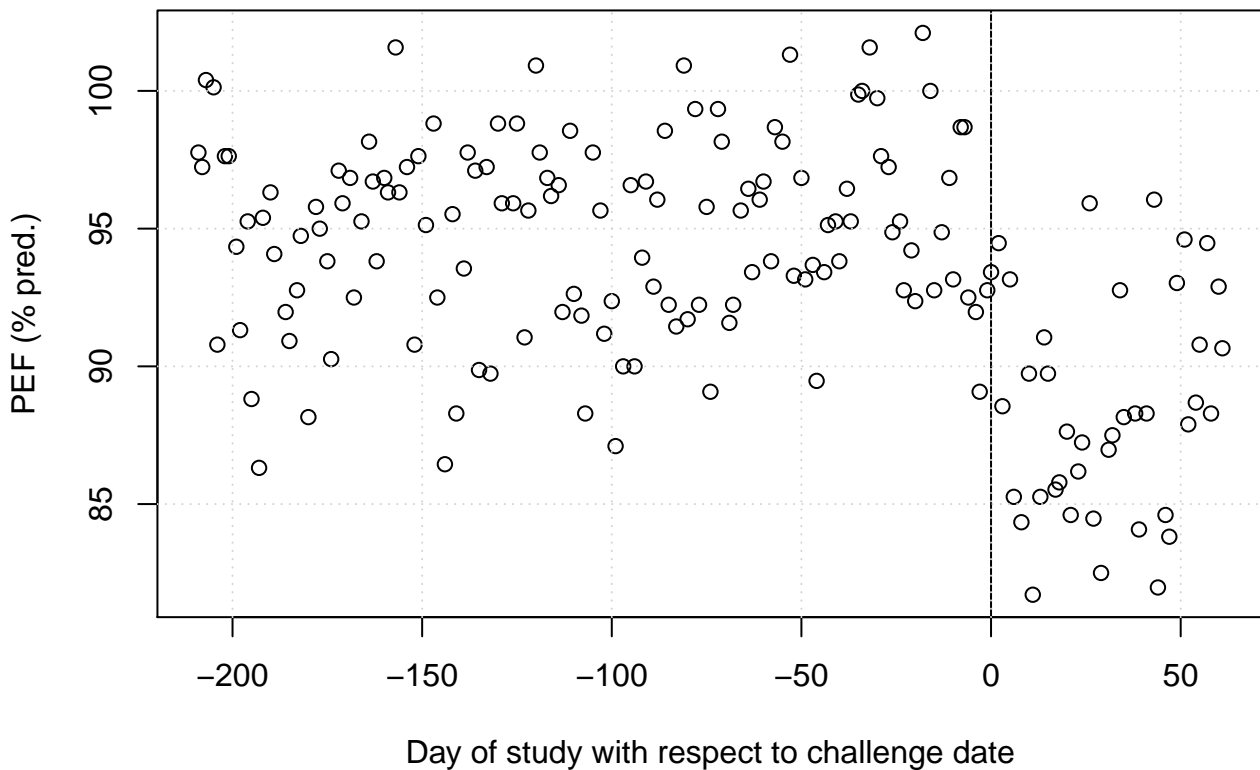

# P06A

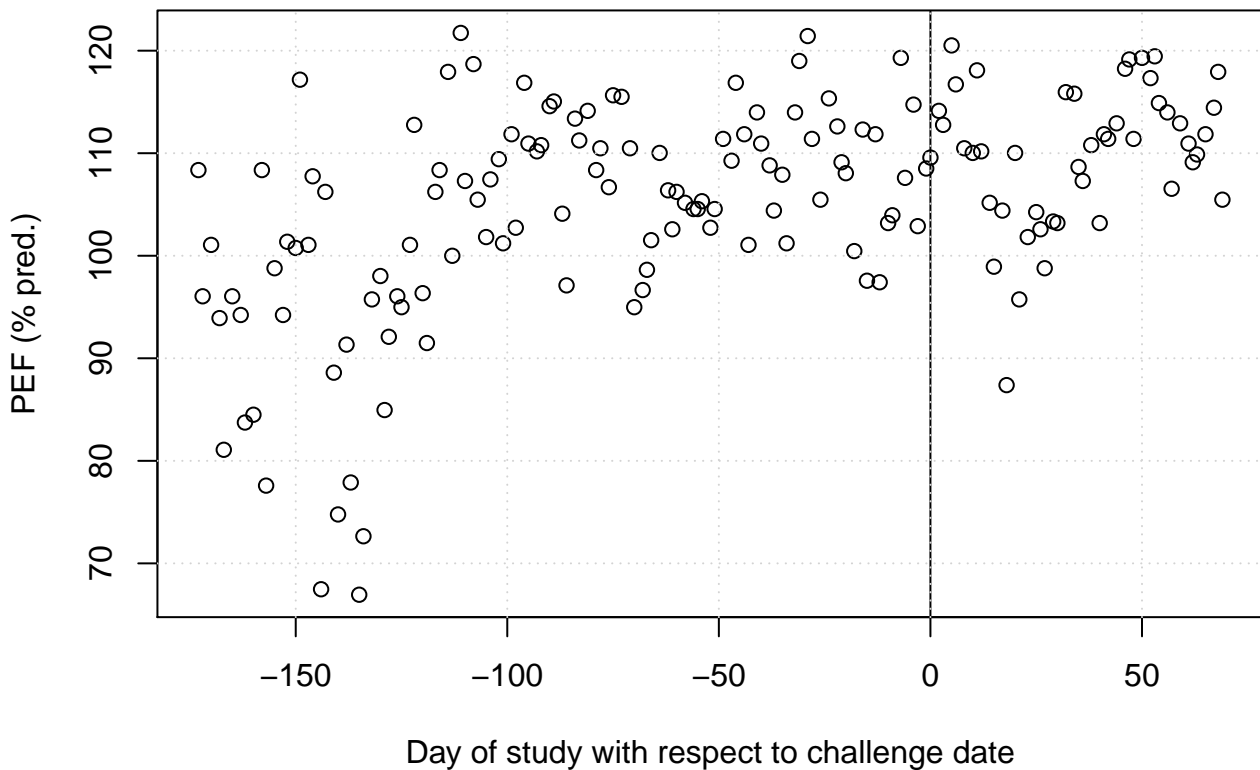

# P07A

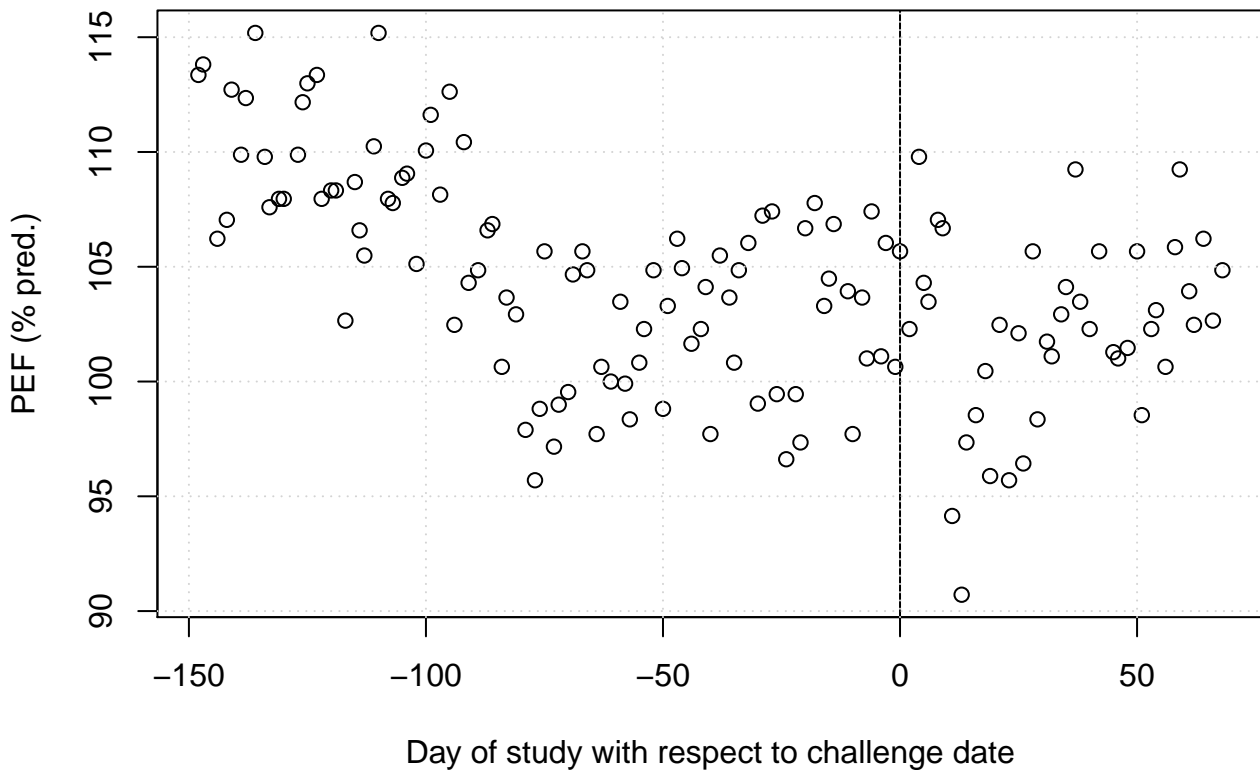

**P08A**

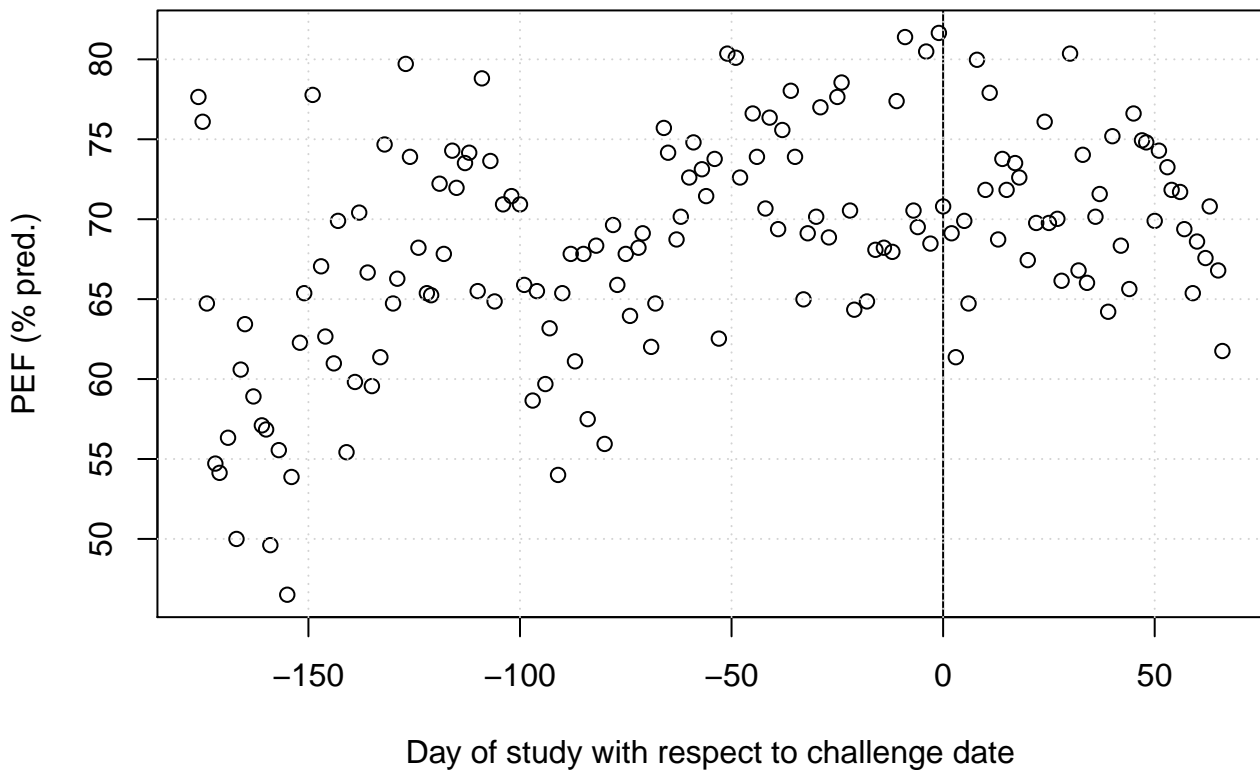

# P09A

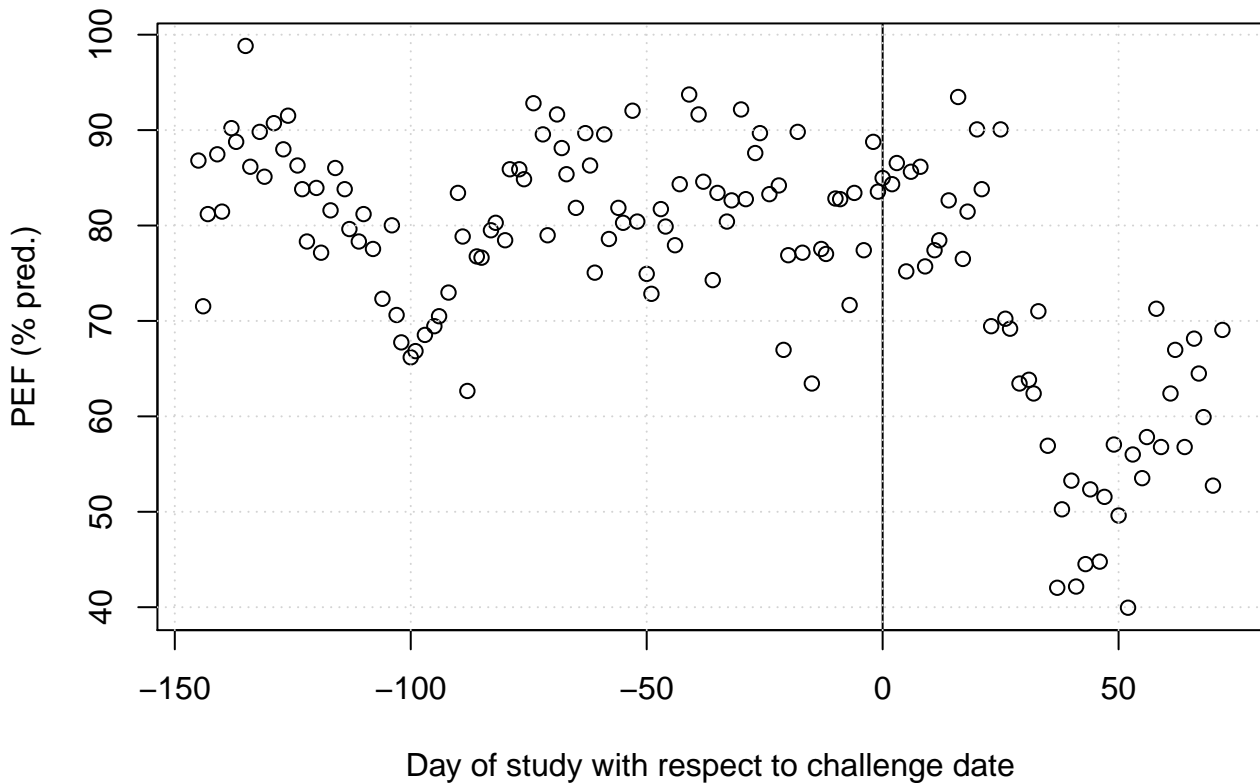

# P10A

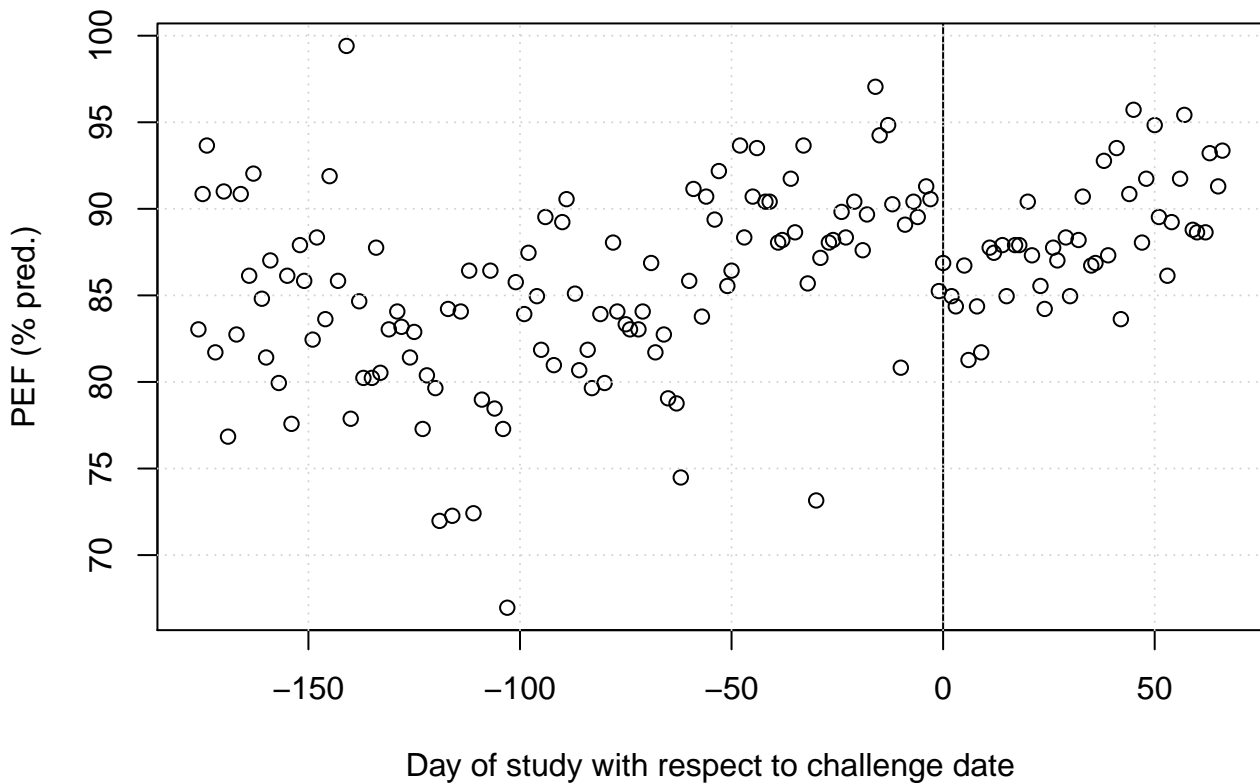

# P11A

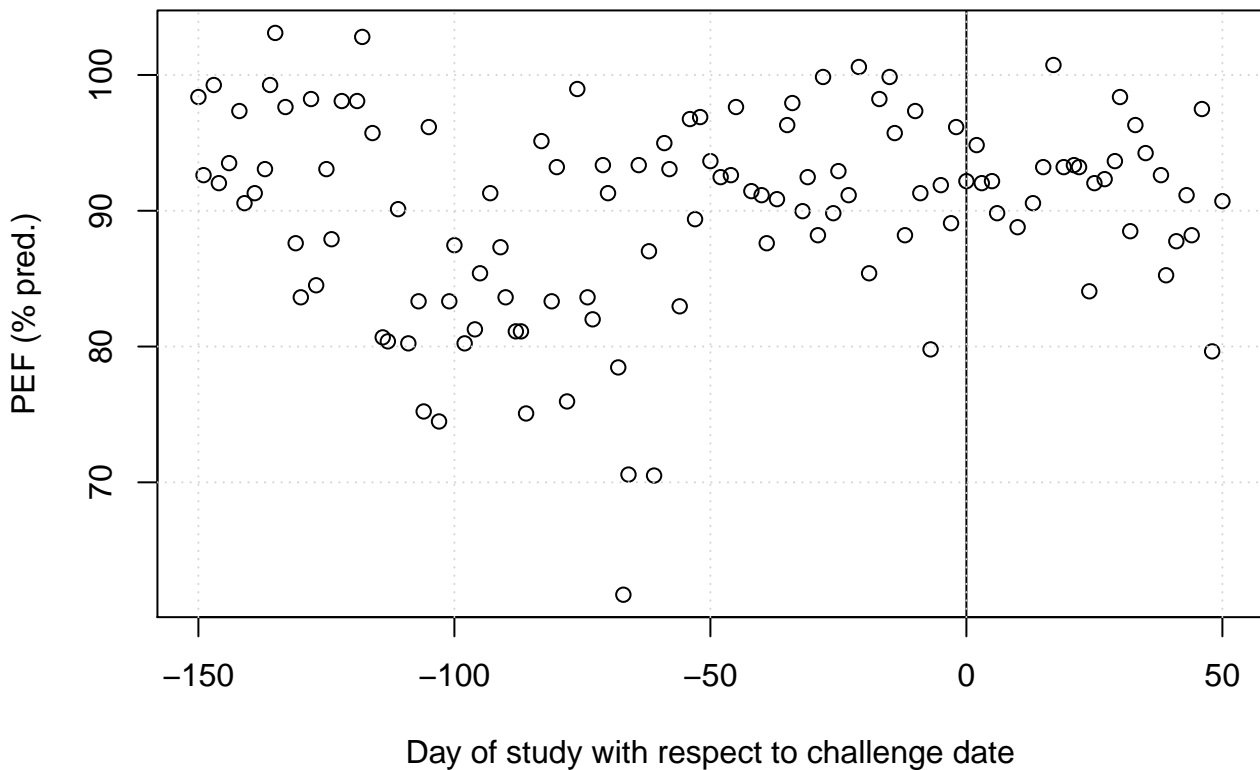

## P12A

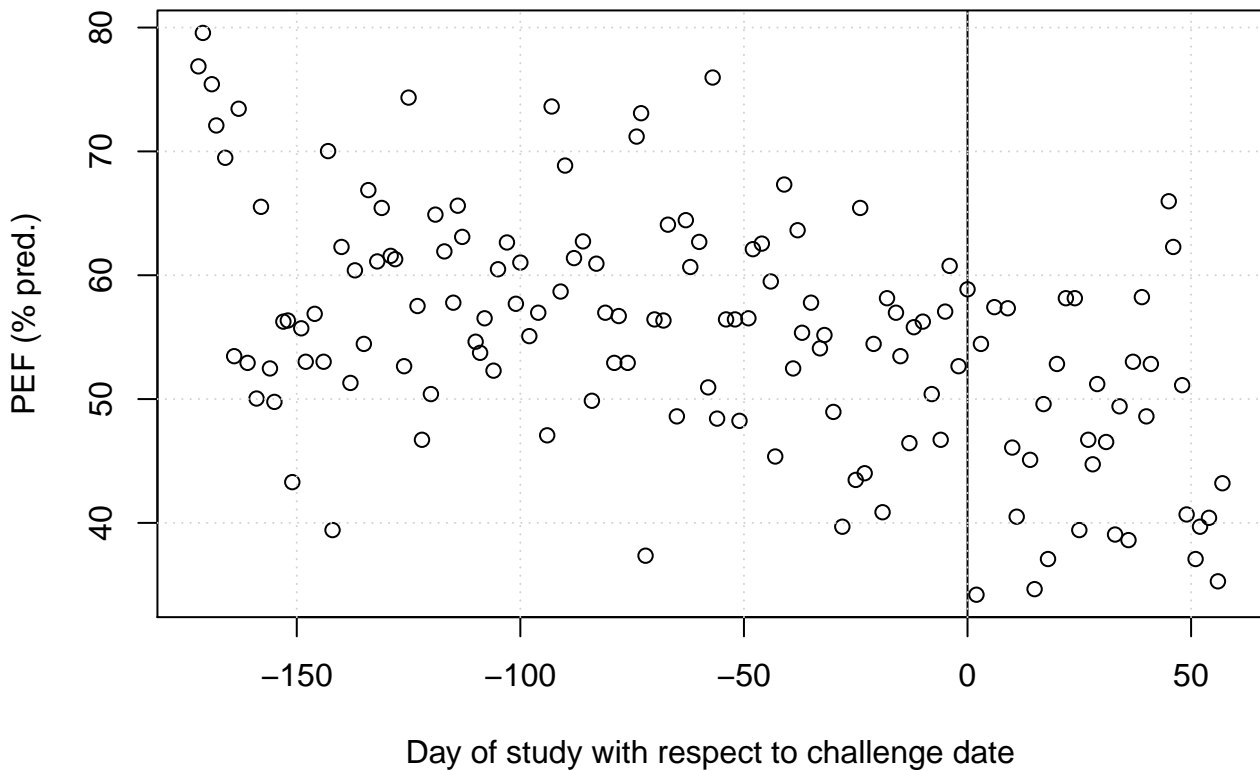

# P13A

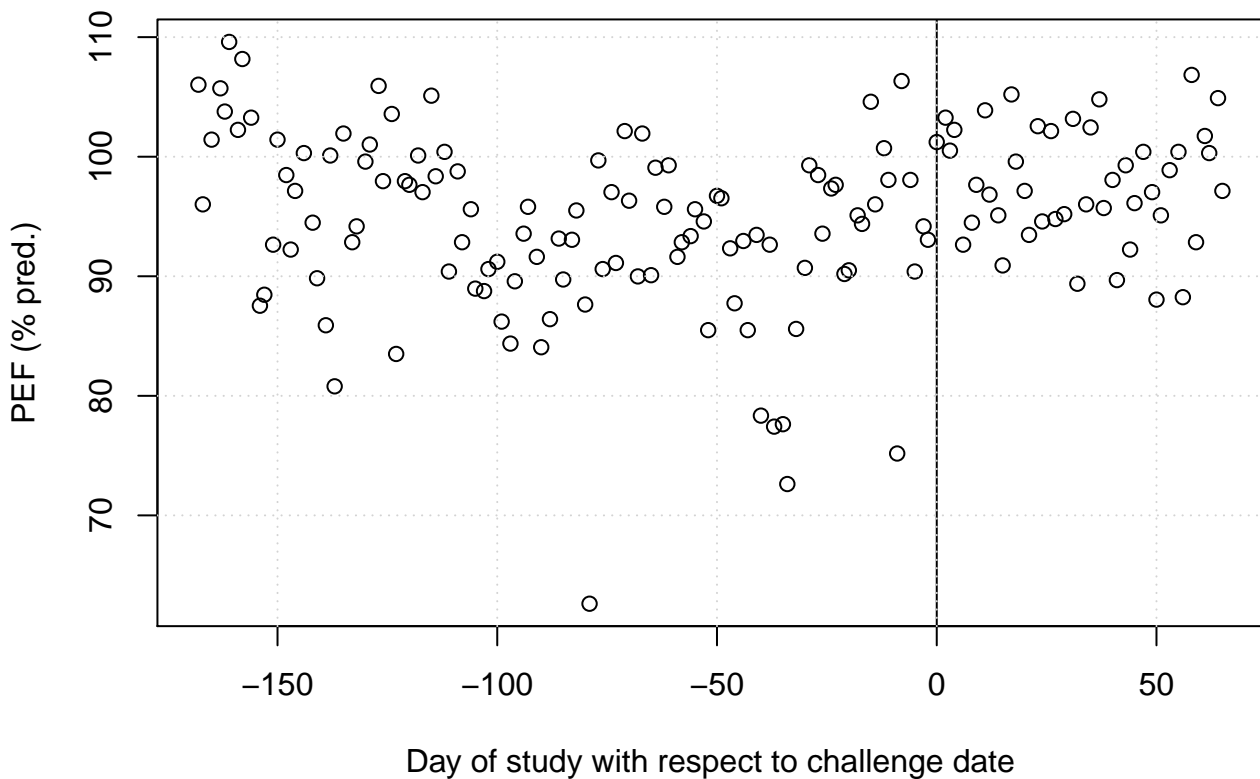

Supplement: Supplementary file 2. [file elife-47969-supp2.zip › TimeSeriesPlots_PDFs/Appendix-figure SS2_PEFpercentPred_TimeSeries_AsthmaticParticipants.pdf]

# P01H

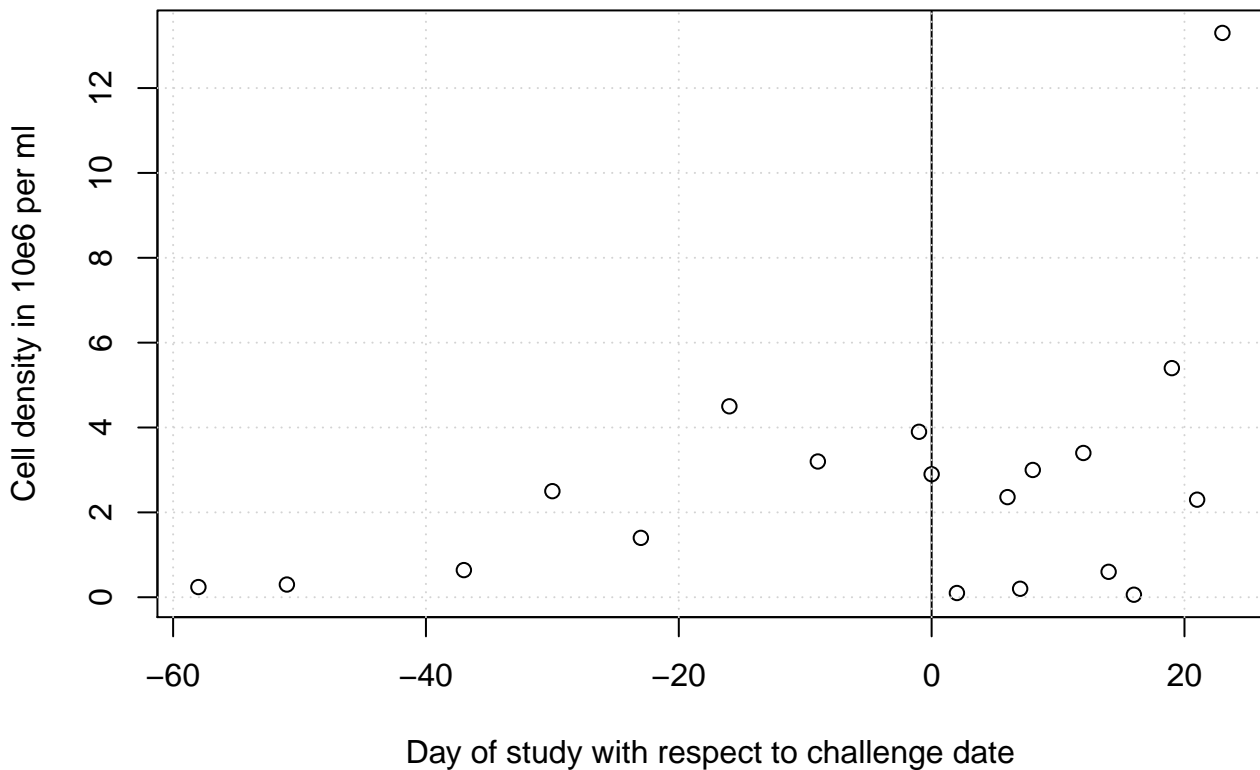

# P03H

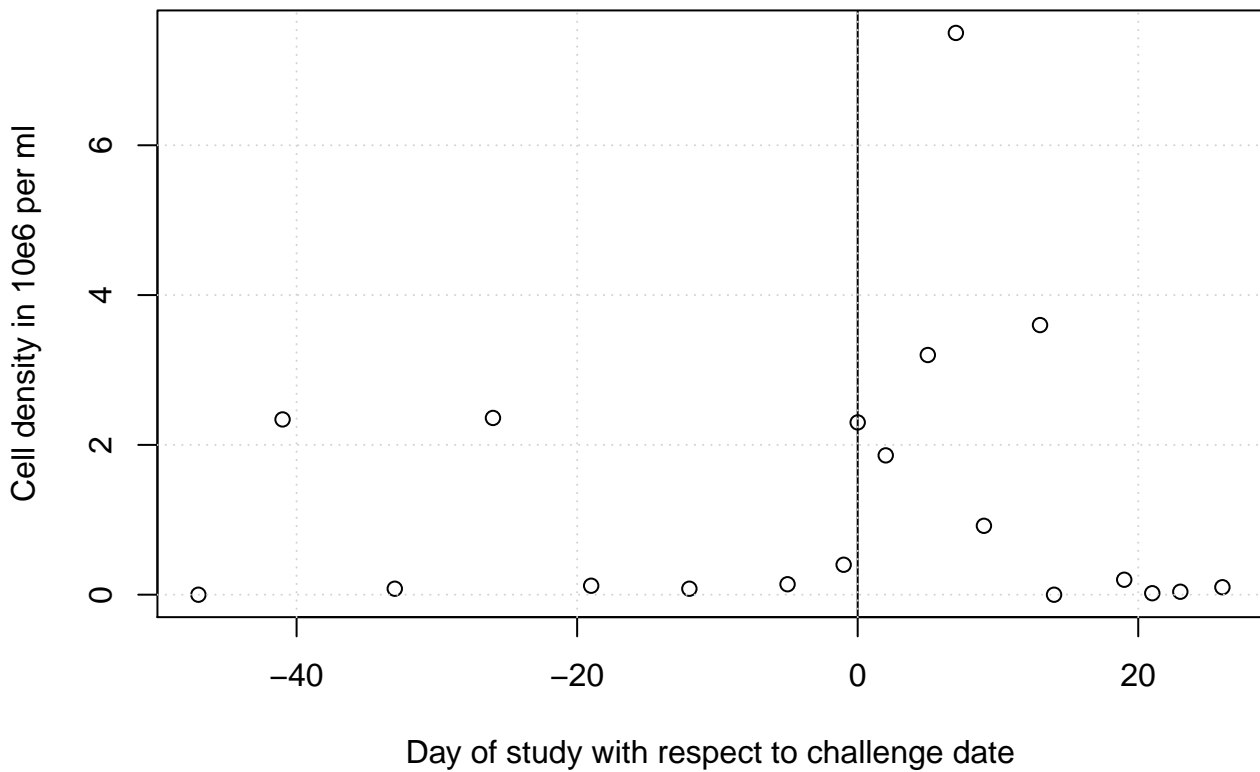

# P05H

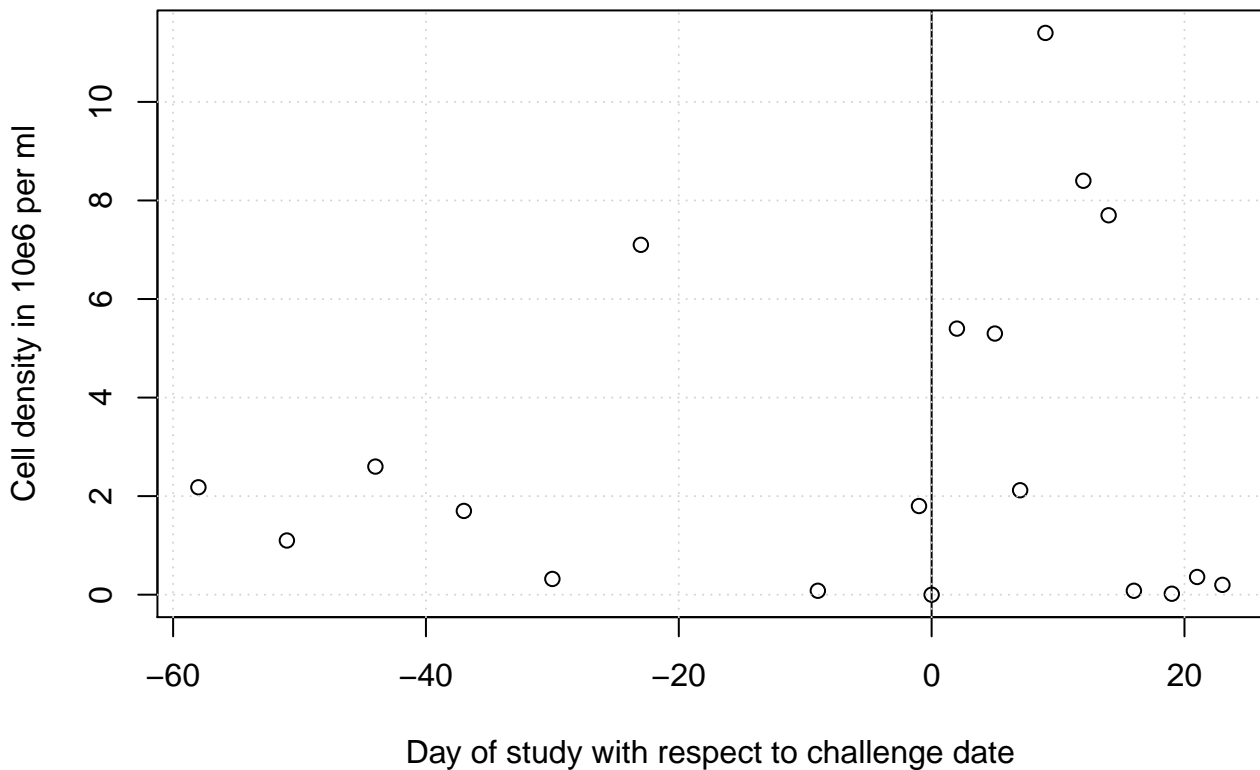

# P06H

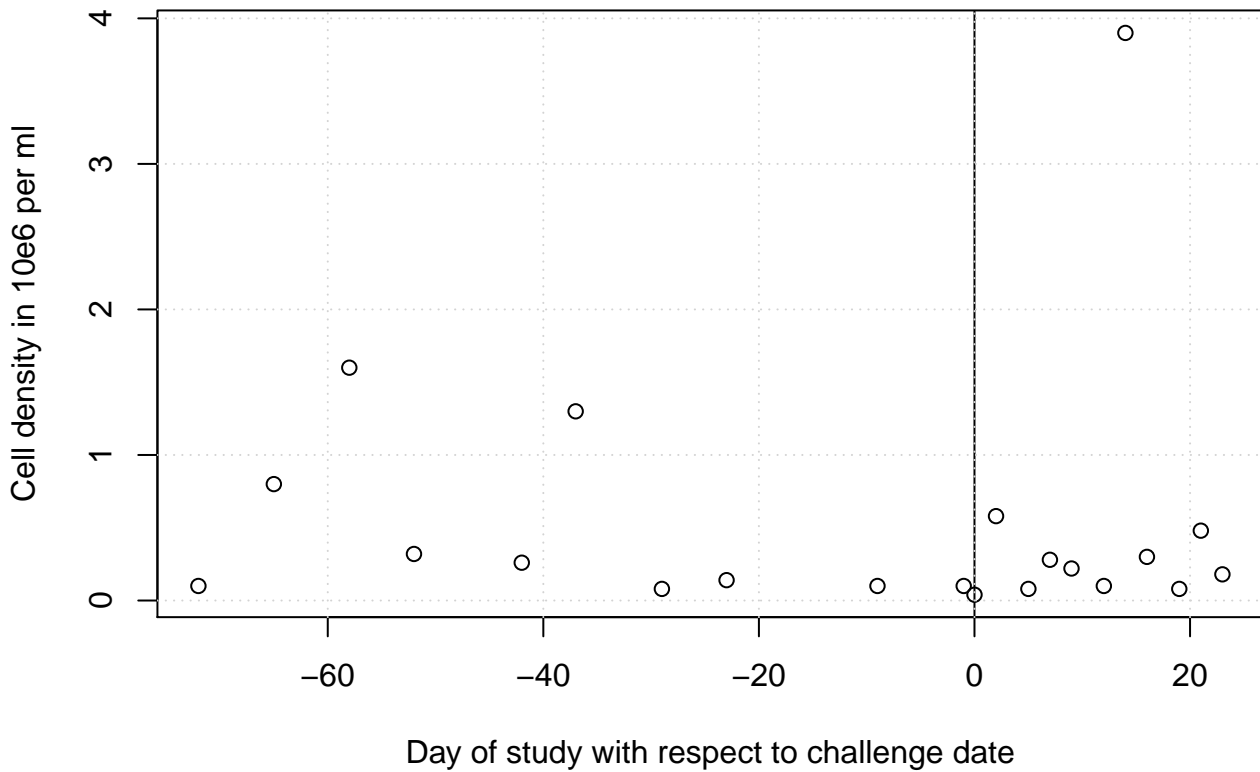

# P07H

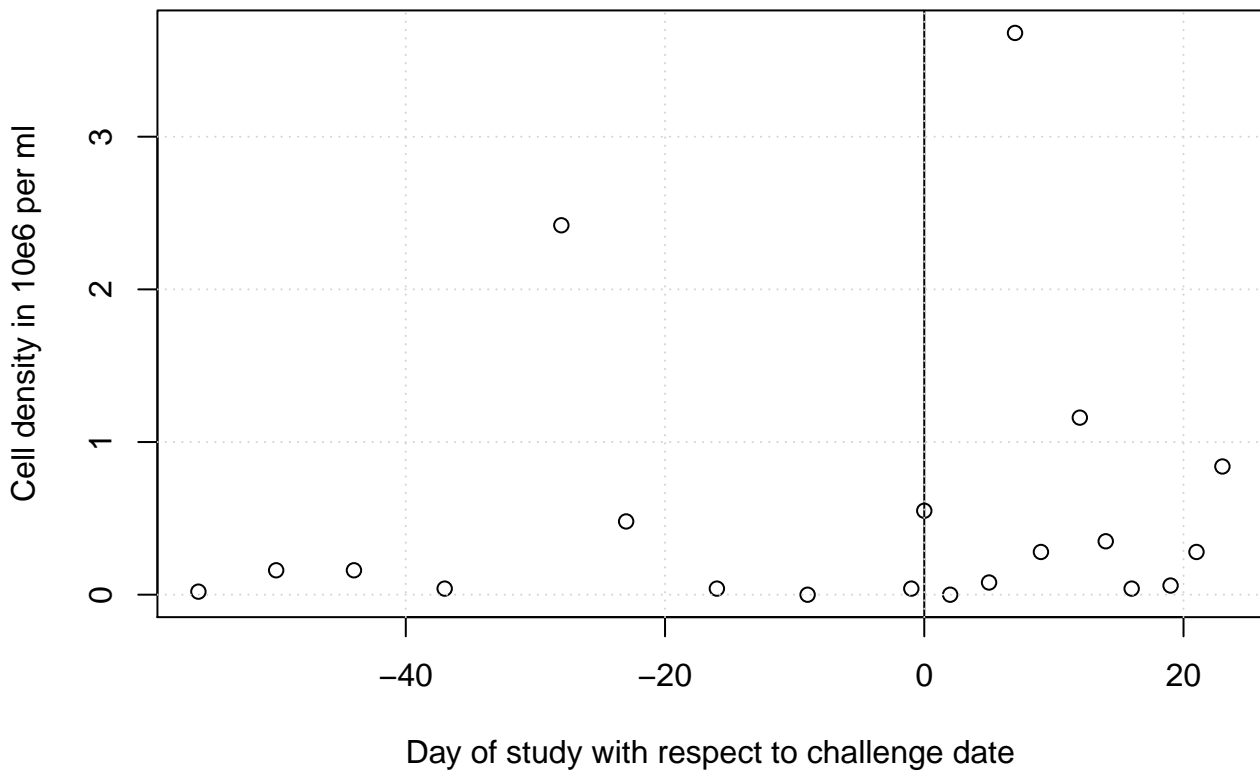

# P08H

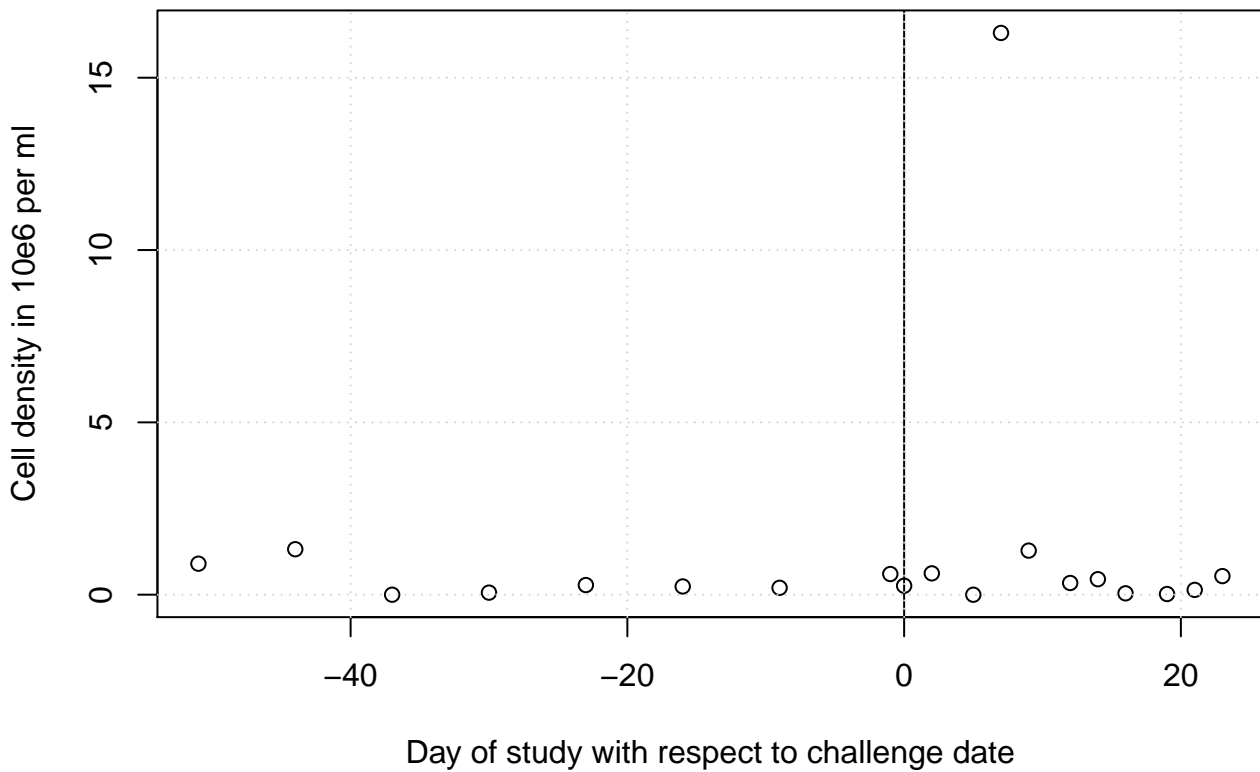

# P09H

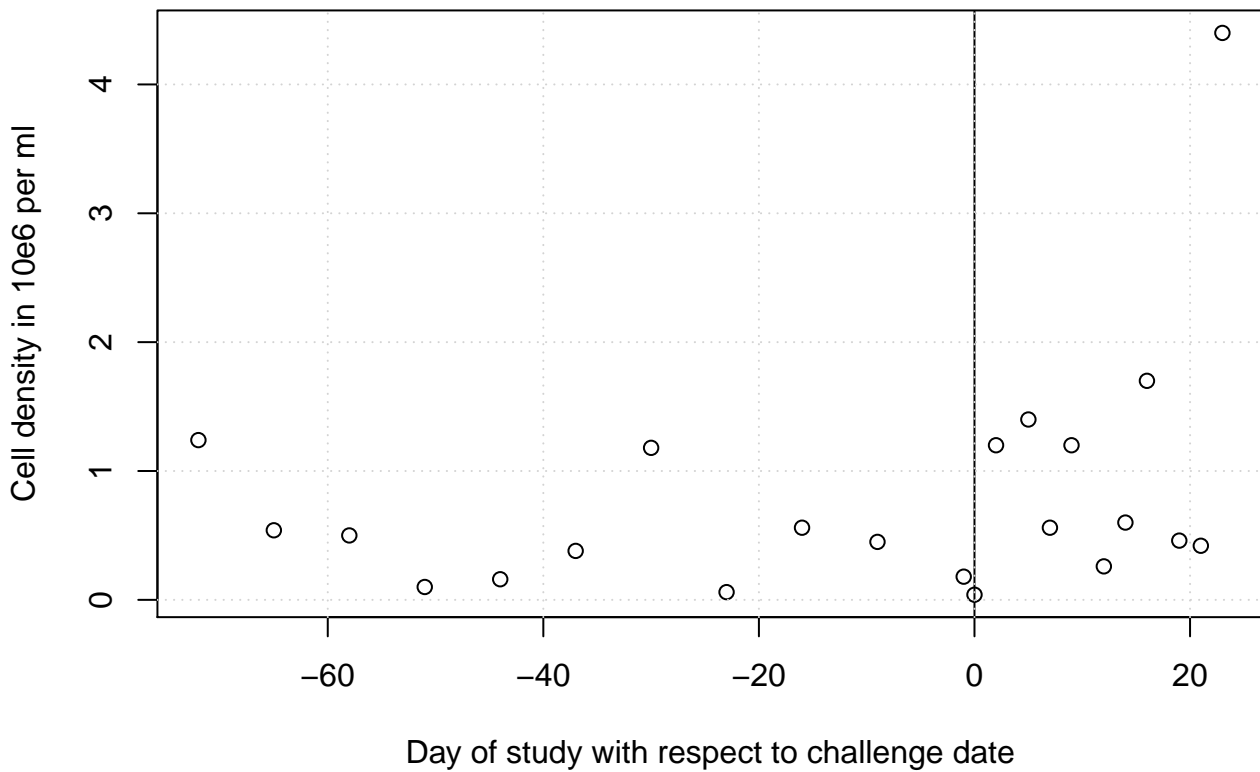

# P11H

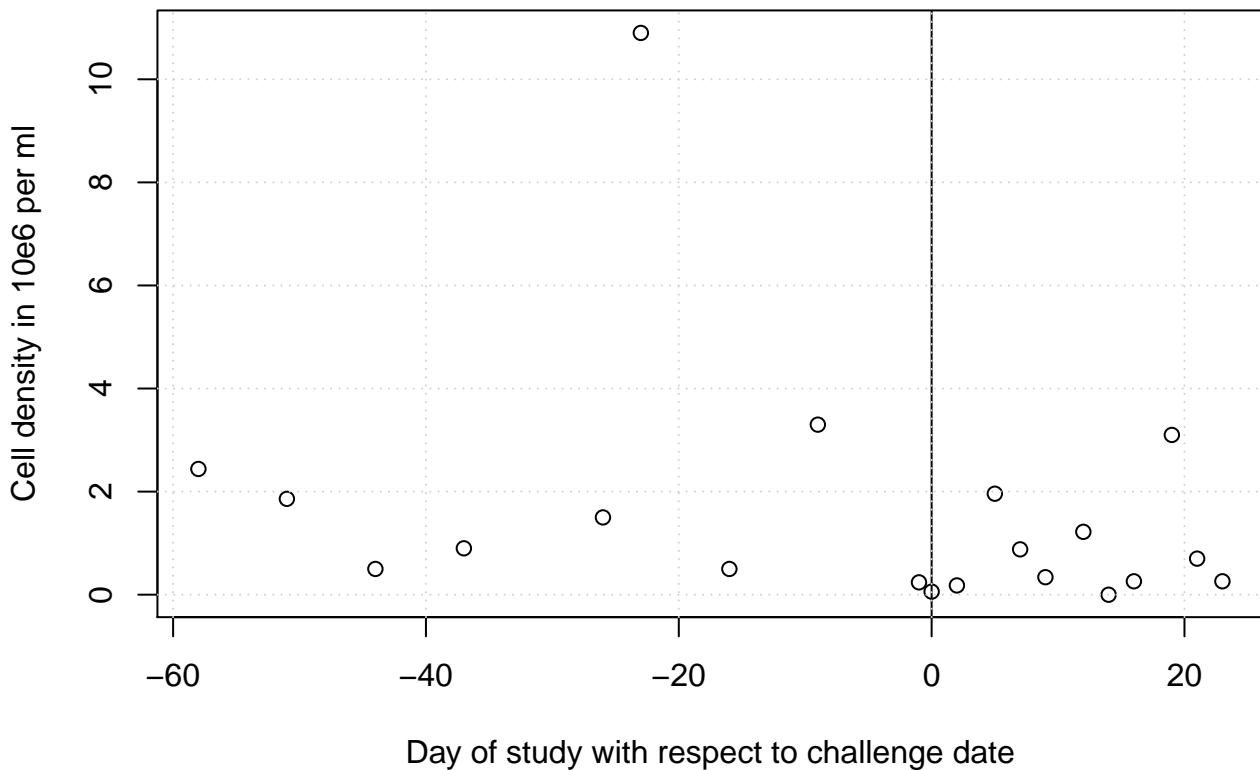

# P12H

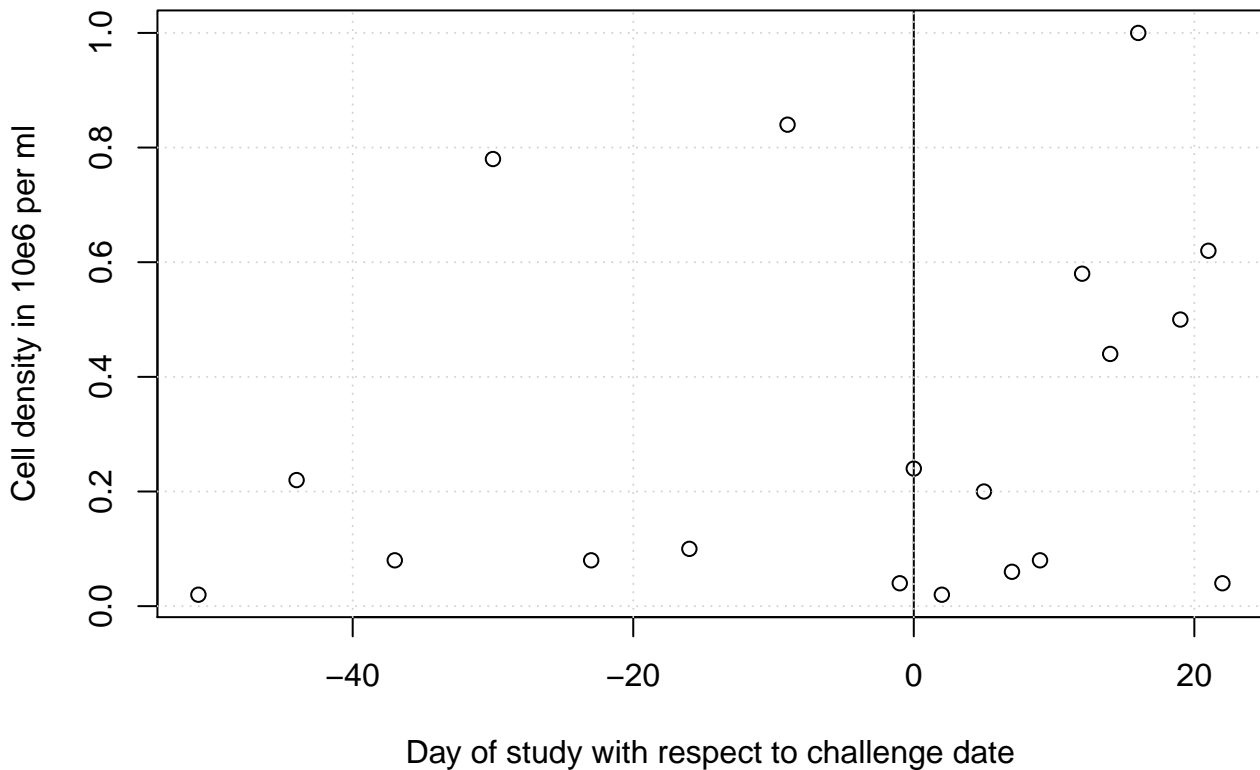

# P13H

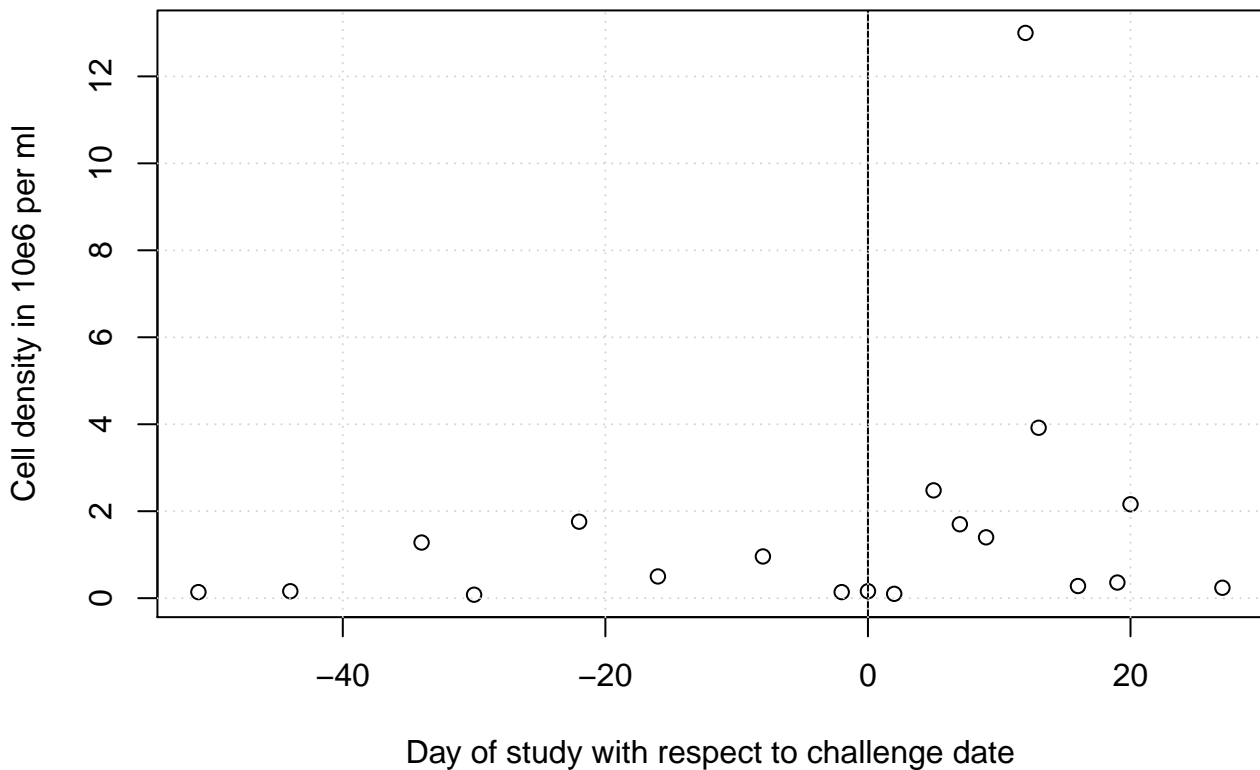

# P14H

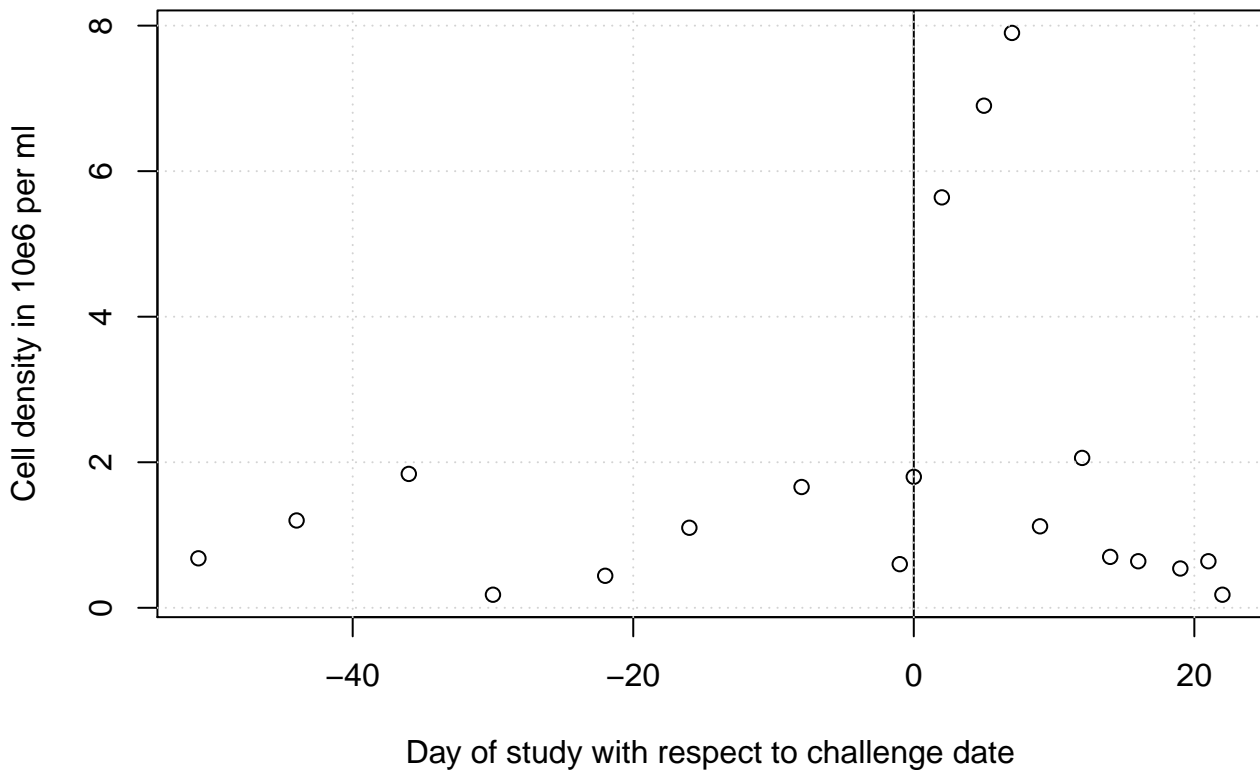

# P15H

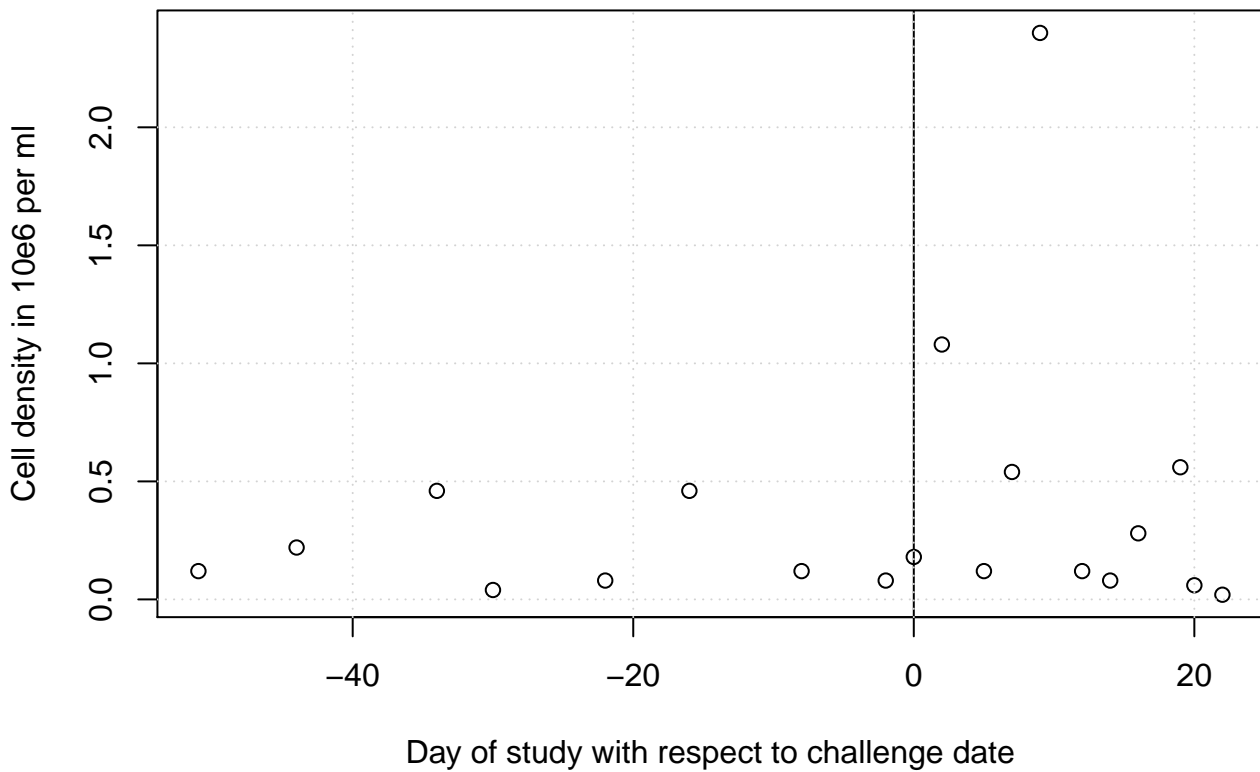

Supplement: Supplementary file 2. [file elife-47969-supp2.zip › TimeSeriesPlots_PDFs/Appendix-figure SS11_CellDensity_In10e6_Per_ml_TimeSeries_HealthyParticipants.pdf]

# P01H

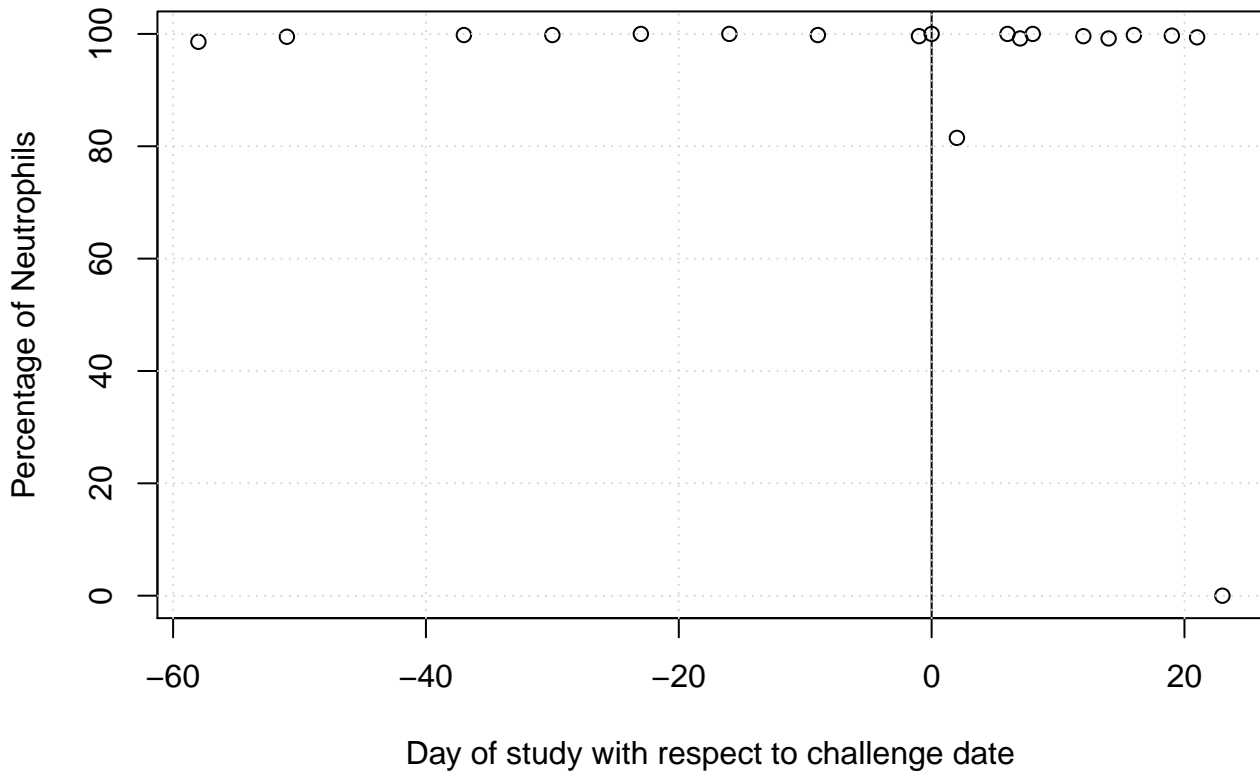

# P03H

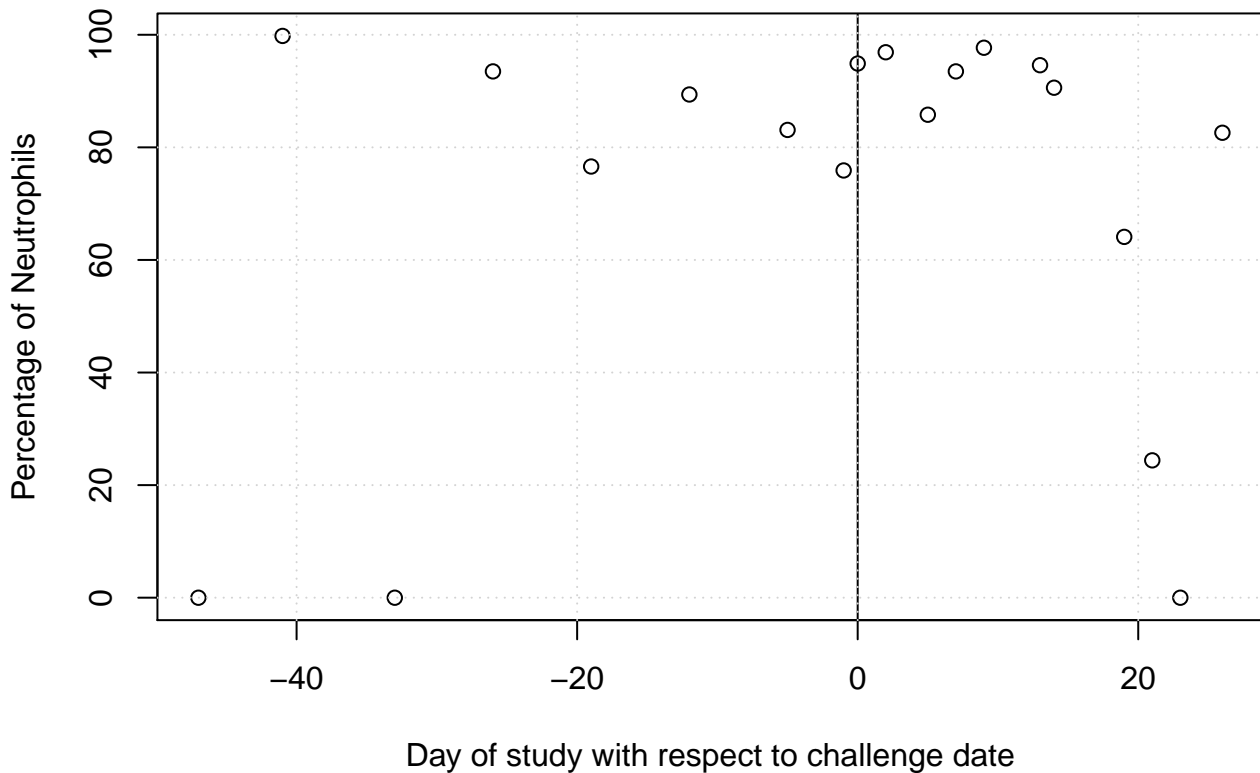

# P05H

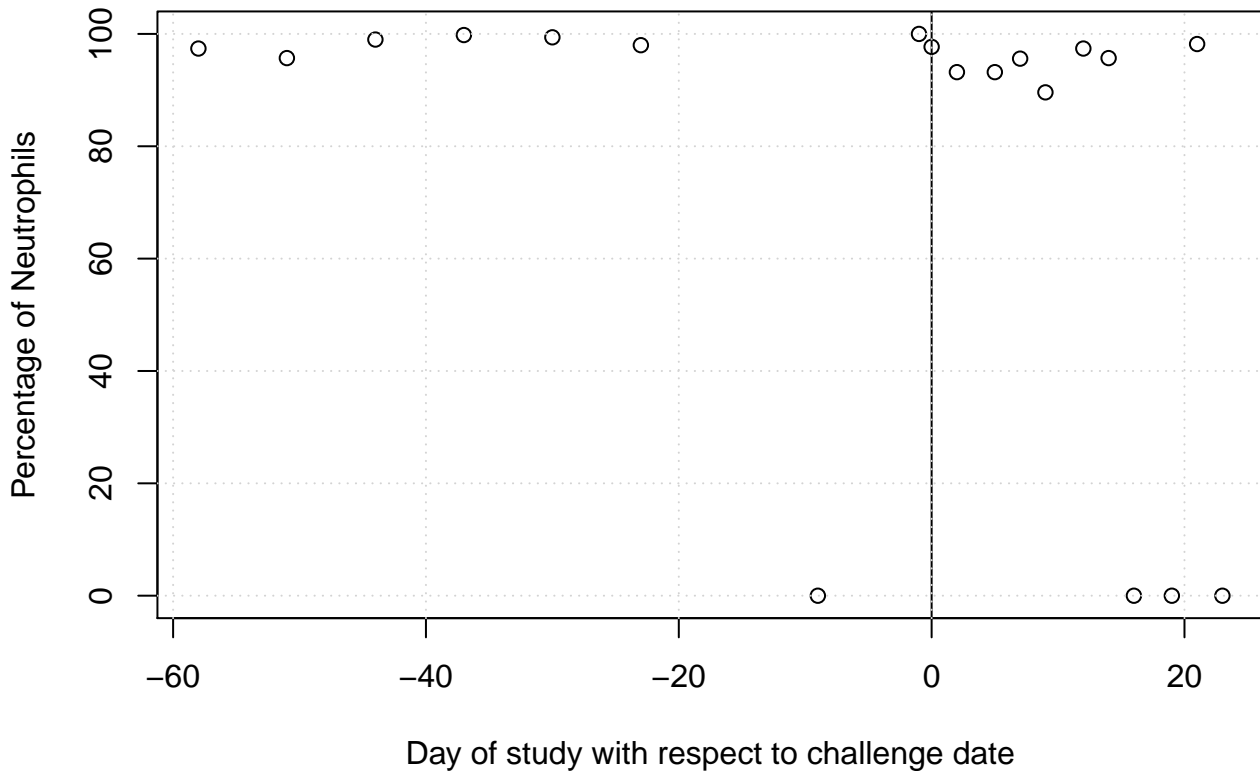

# P06H

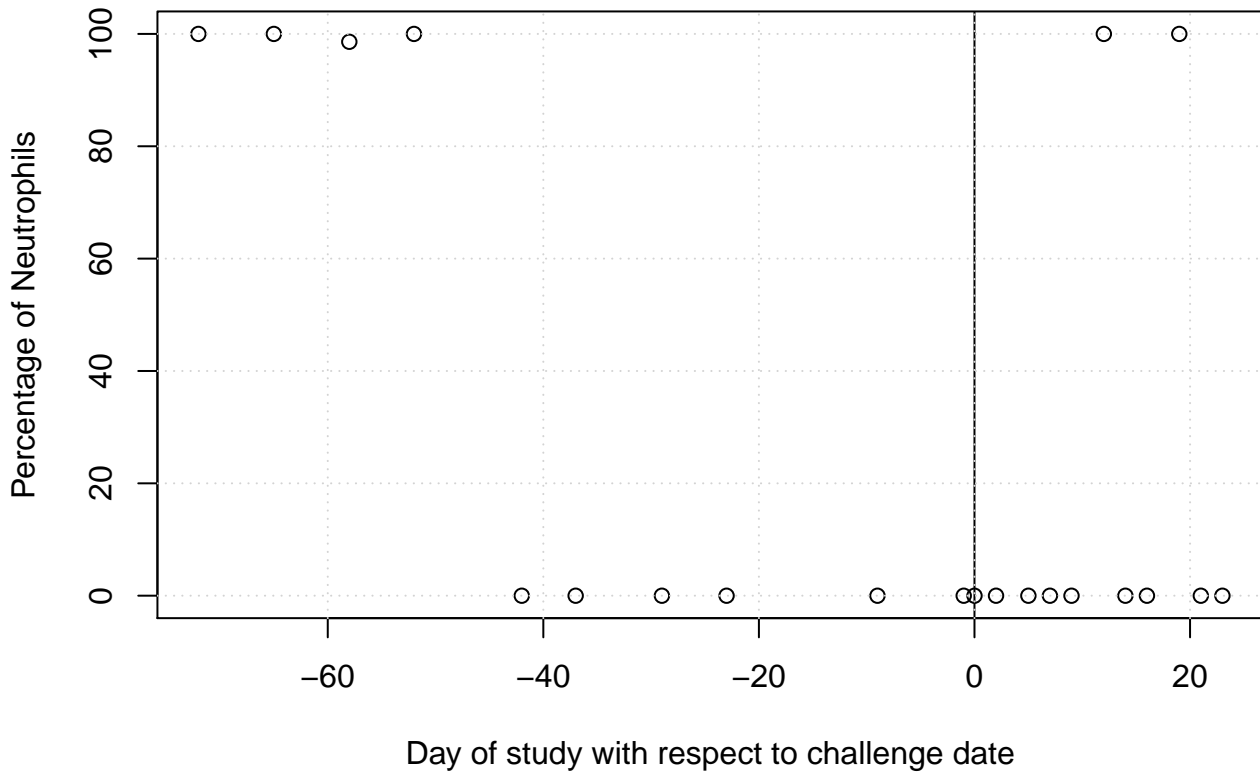

# P07H

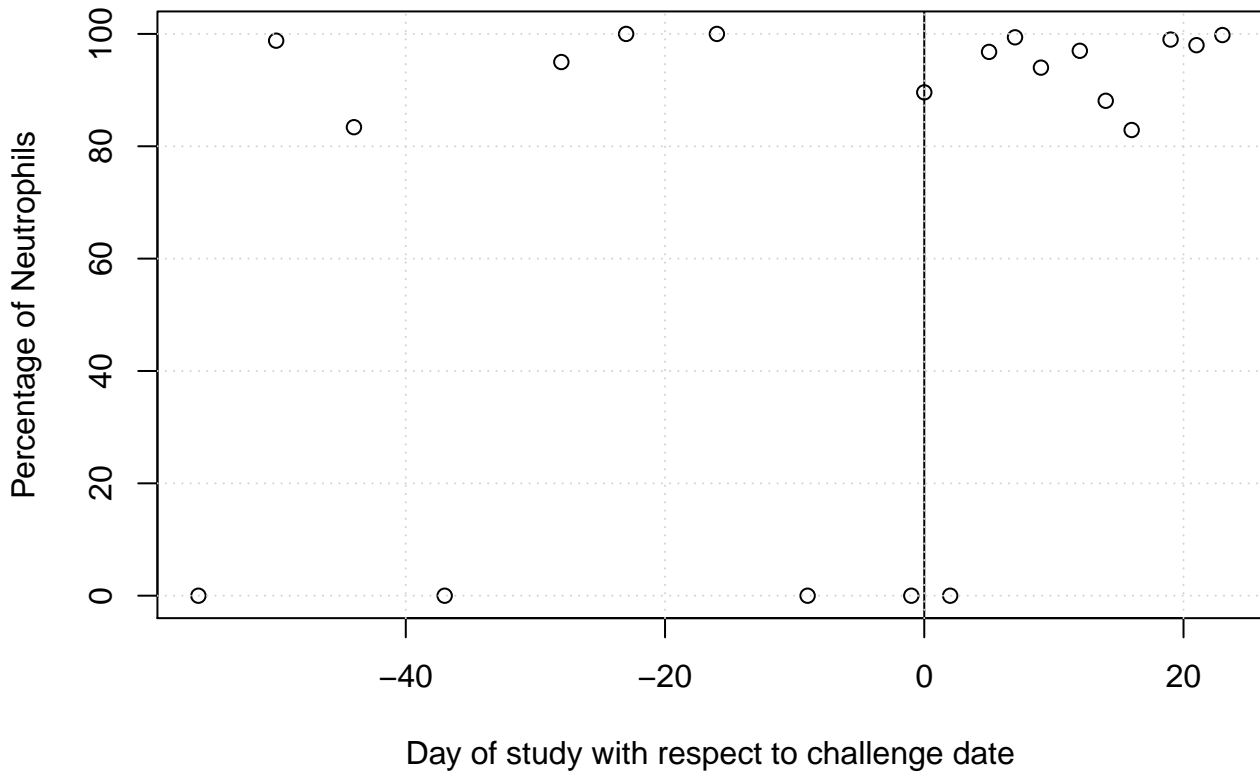

# P08H

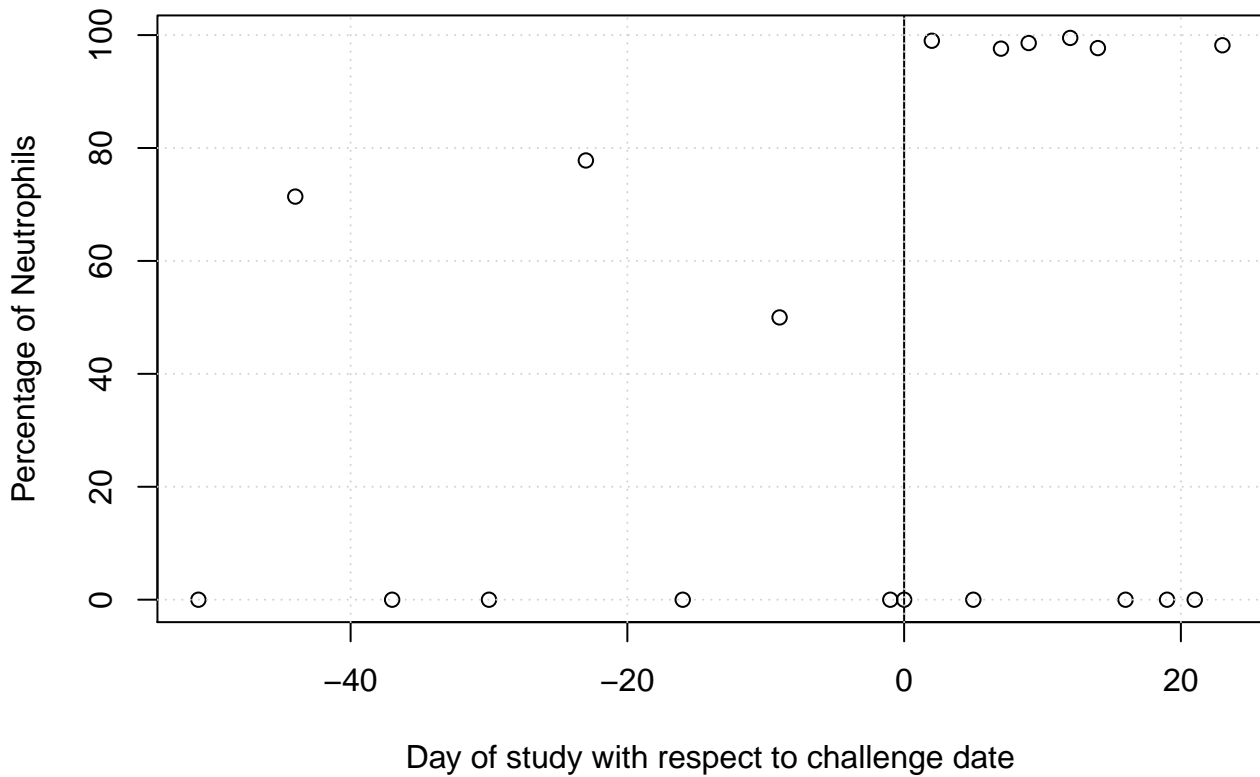

# P09H

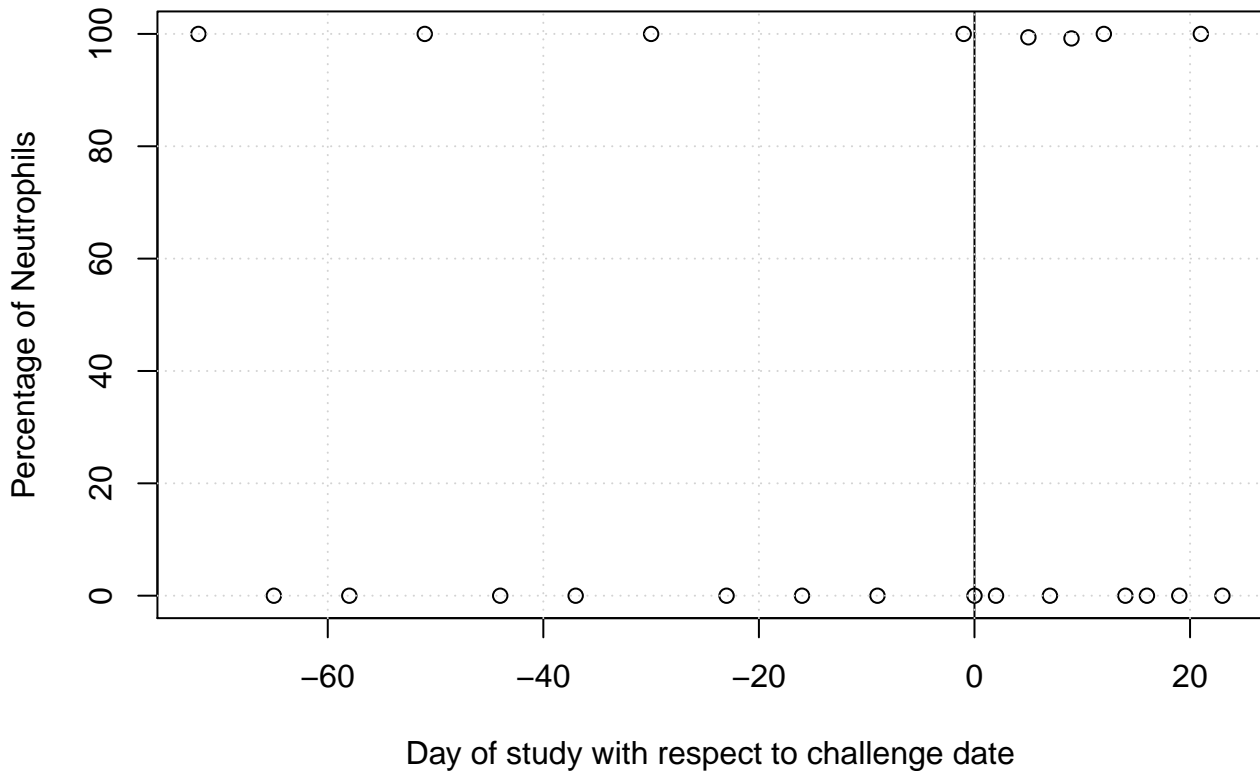

# P11H

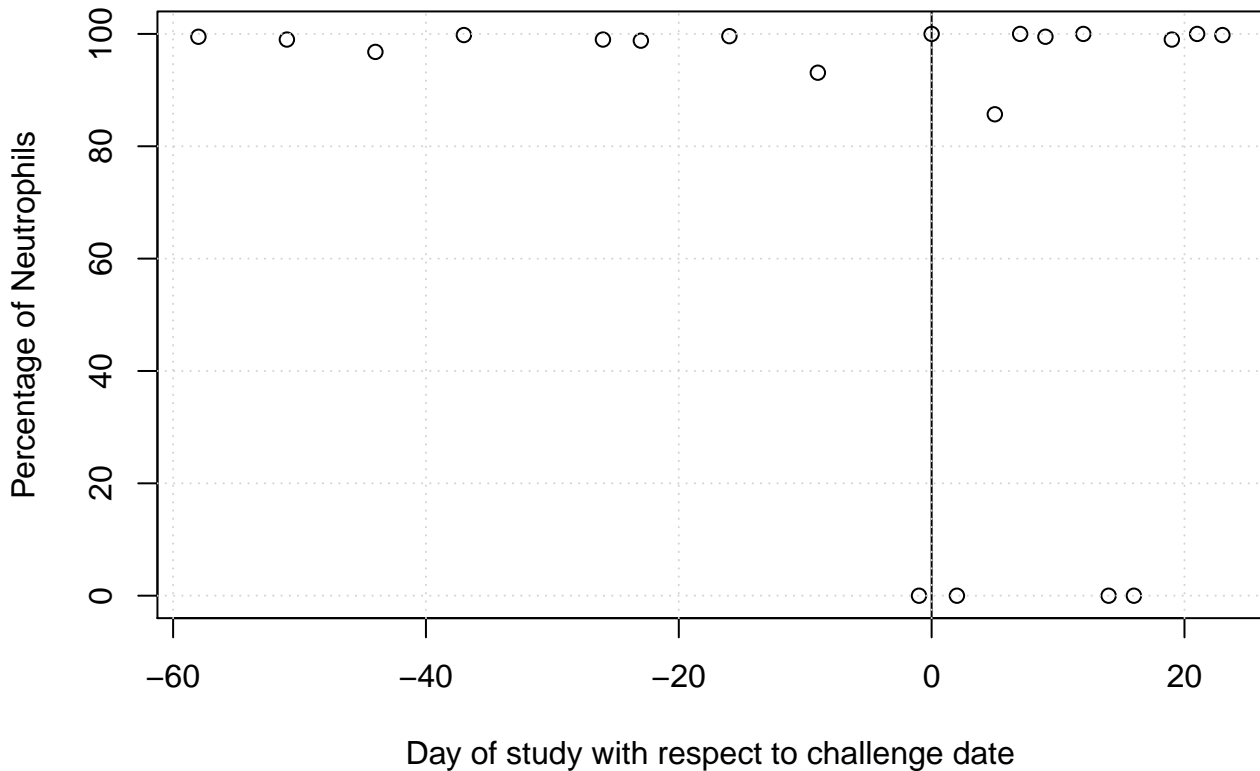

# P12H

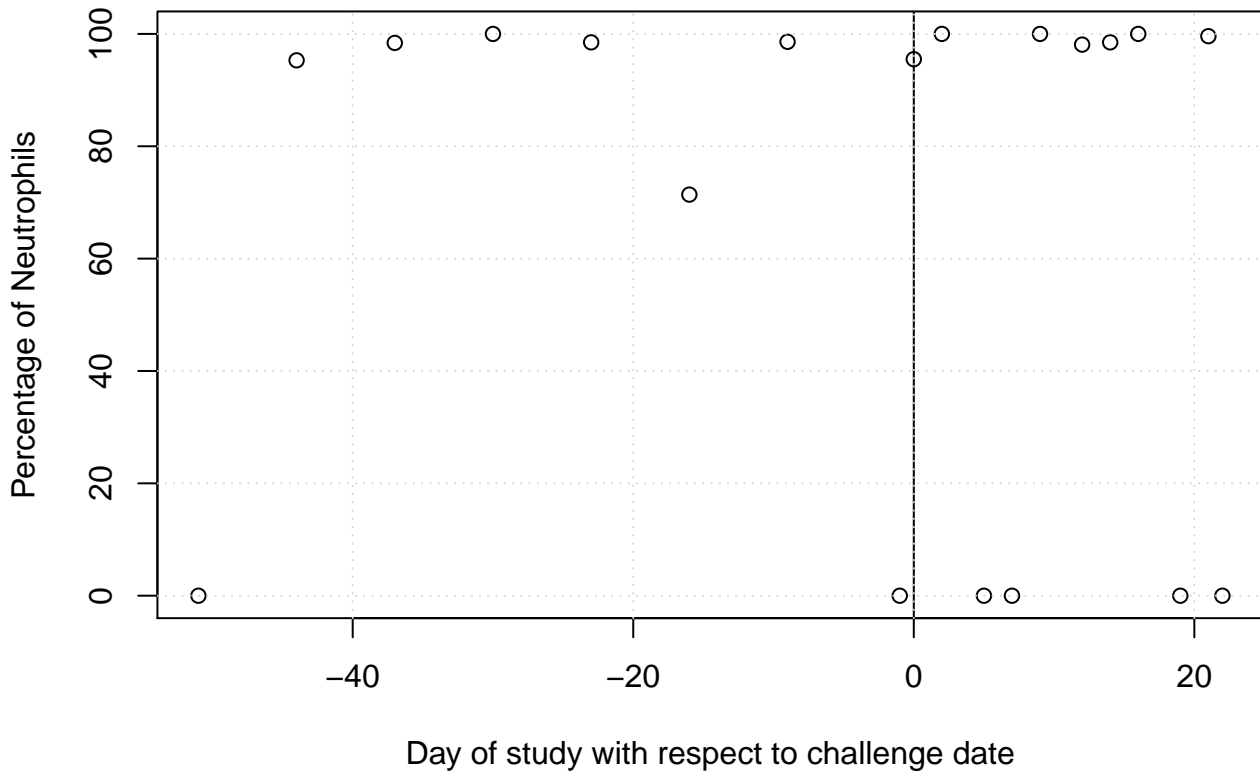

# P13H

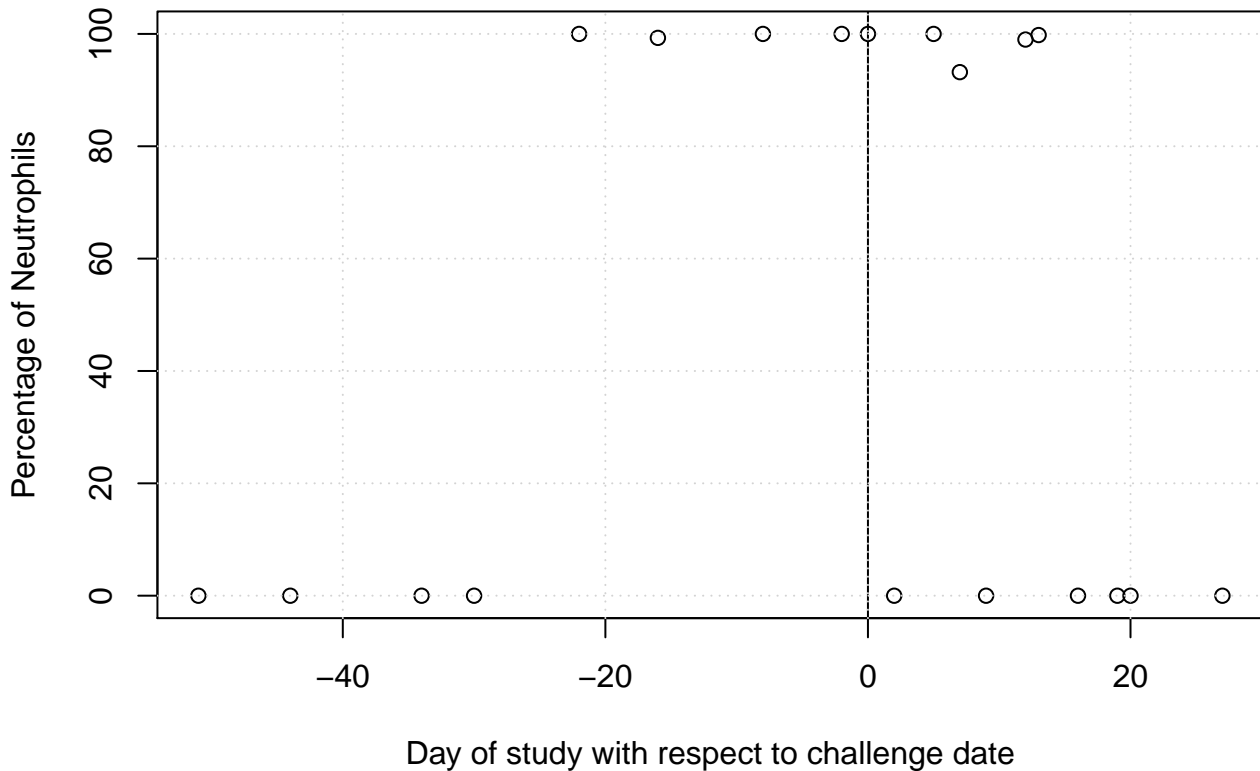

# P14H

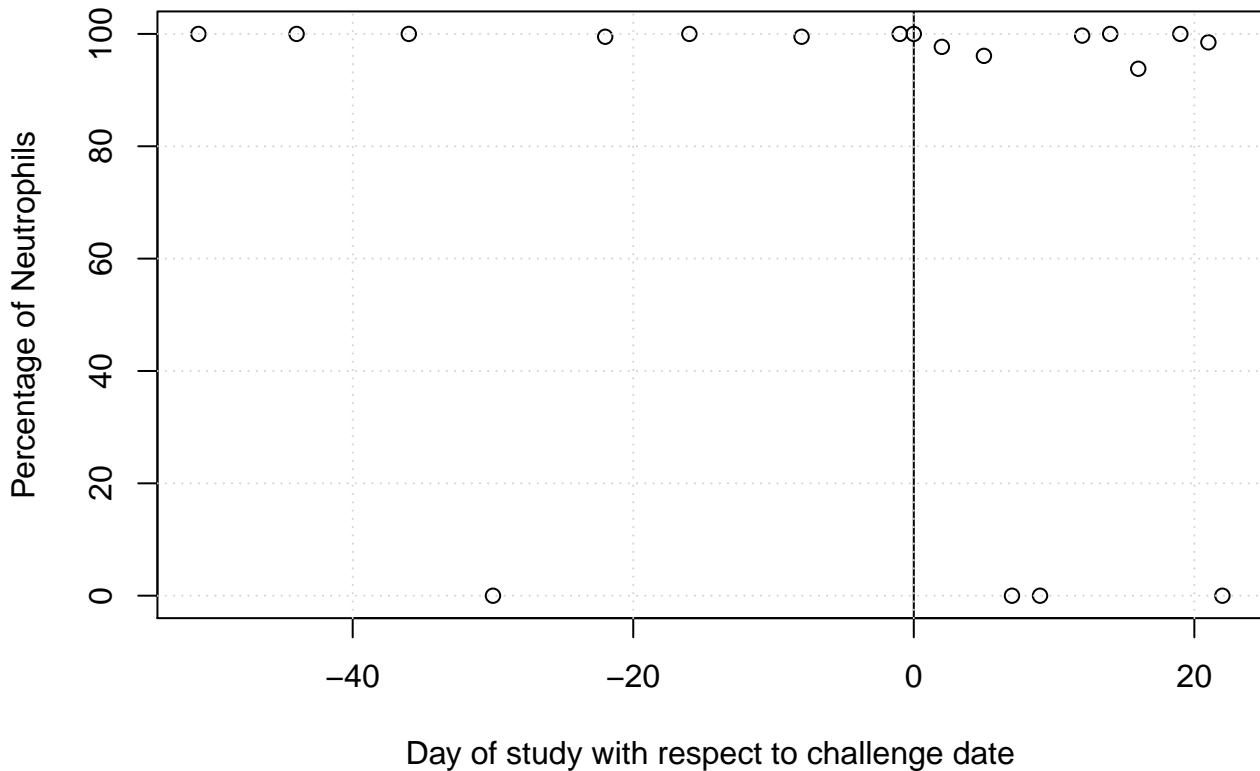

# P15H

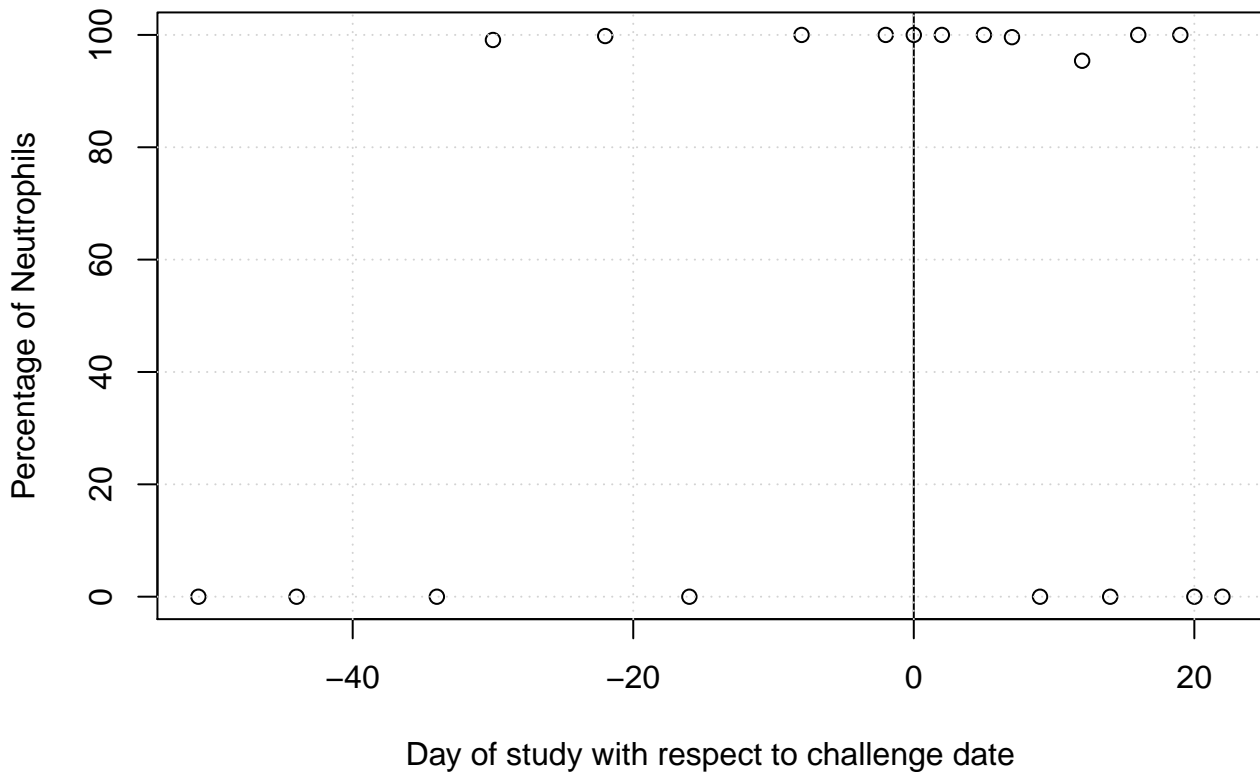

Supplement: Supplementary file 2. [file elife-47969-supp2.zip › TimeSeriesPlots_PDFs/Appendix-figure SS15_PercentageOfNeutrophils_TimeSeries_HealthyParticipants.pdf]

# P01H

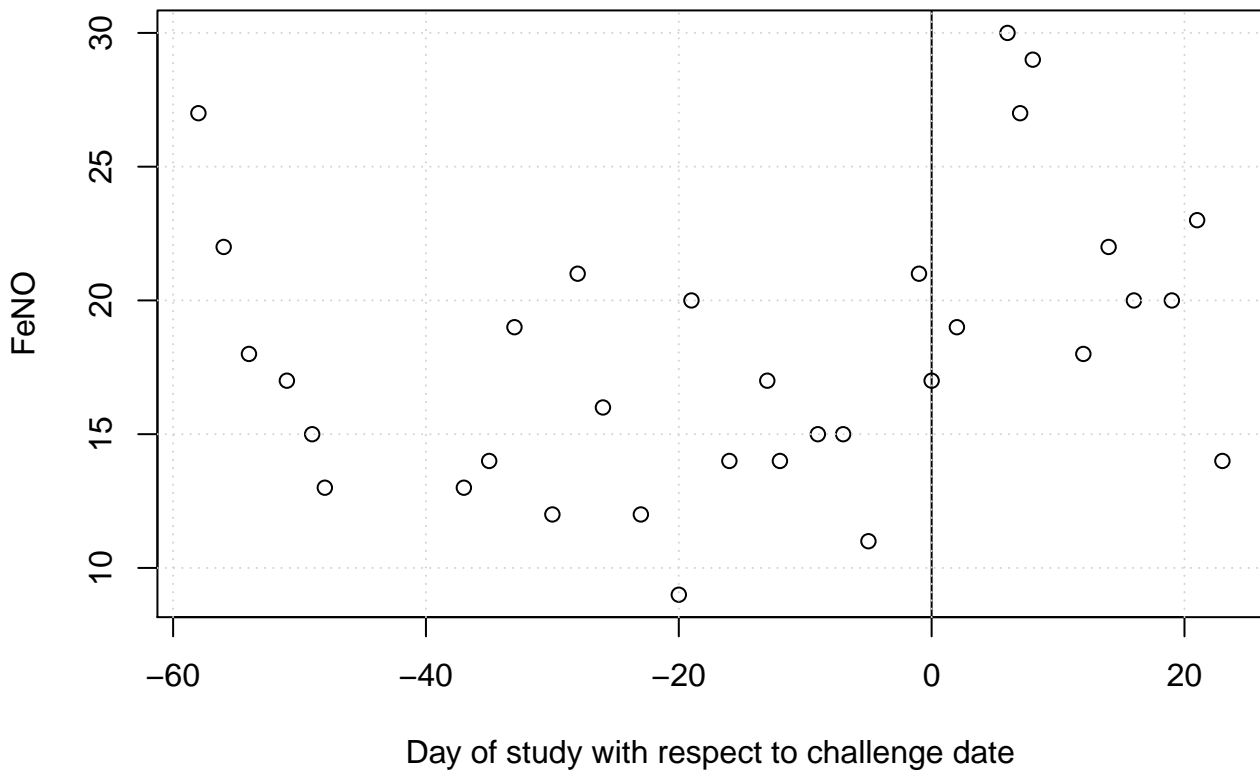

# P03H

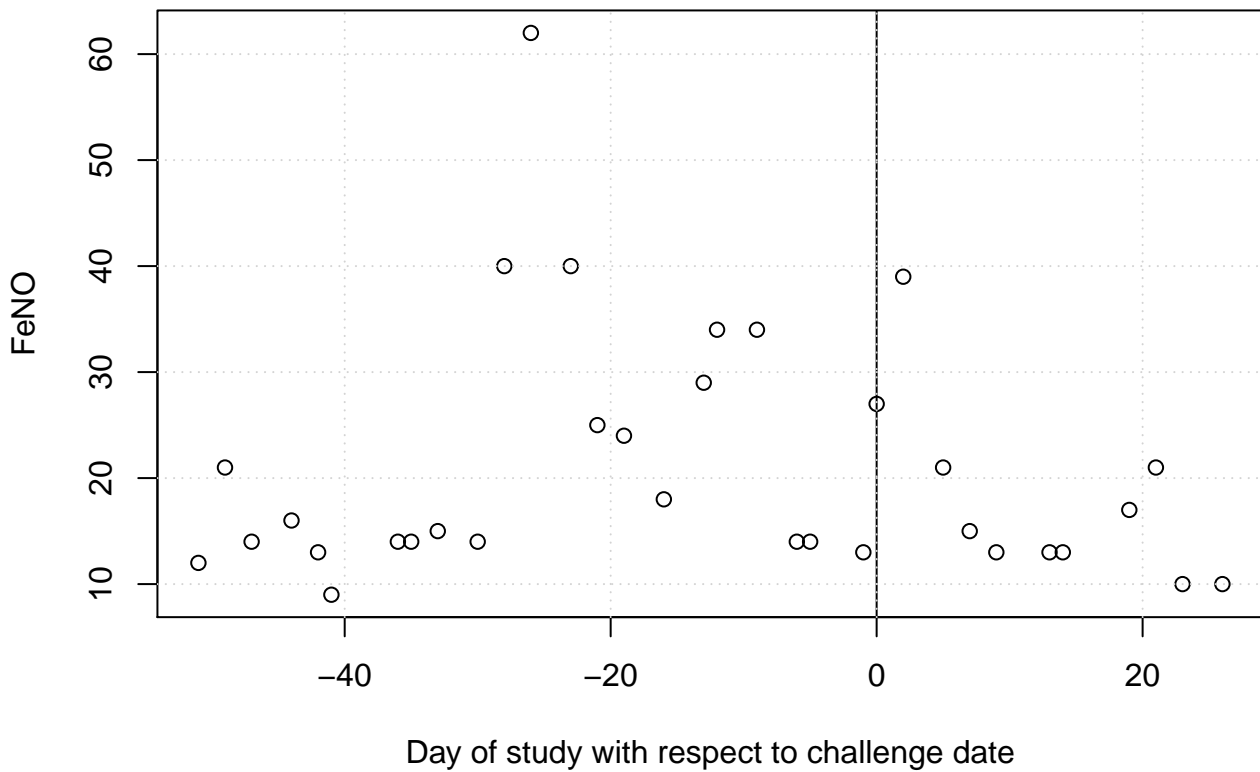

# P05H

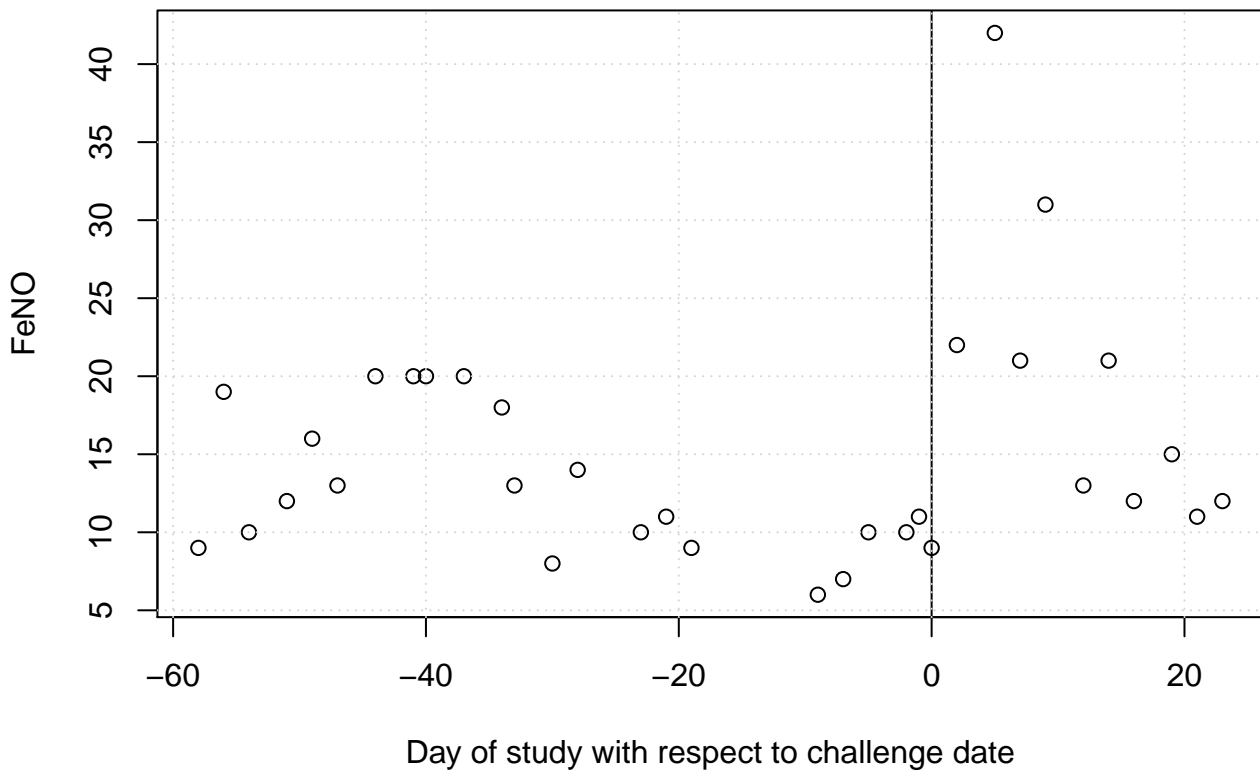

# P06H

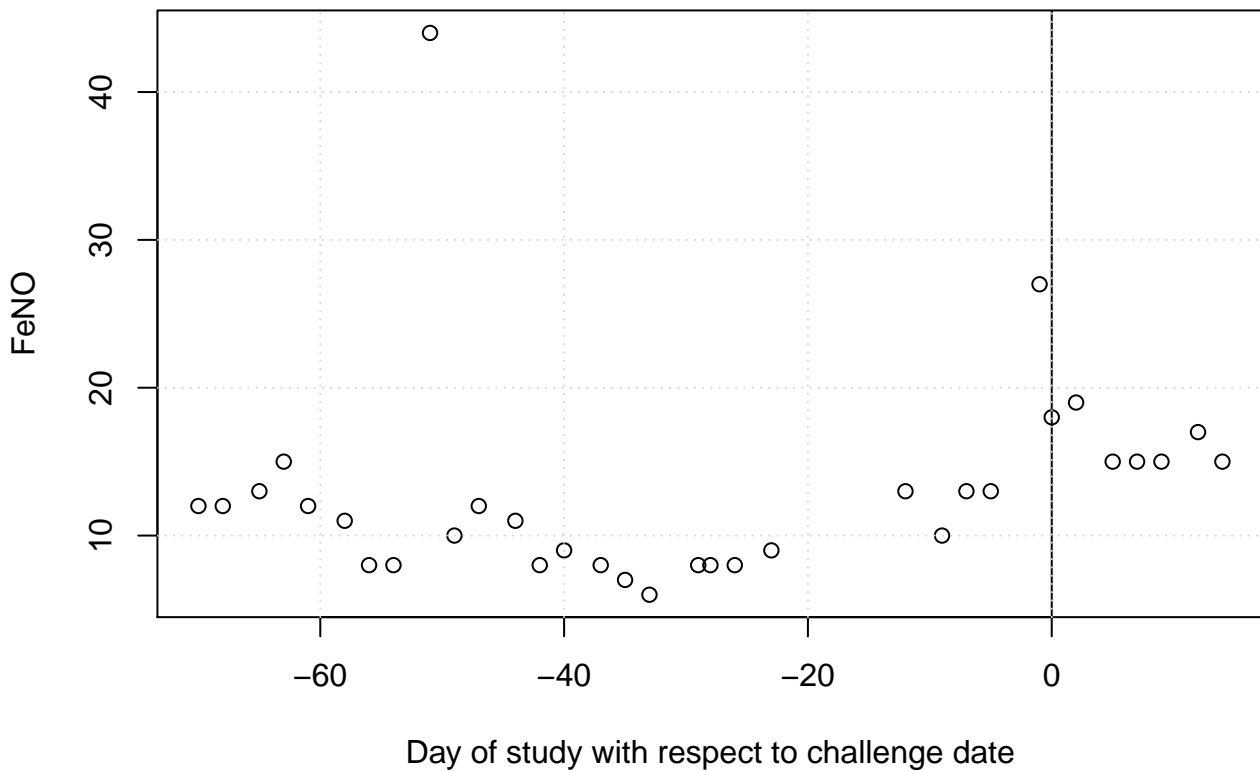

# P07H

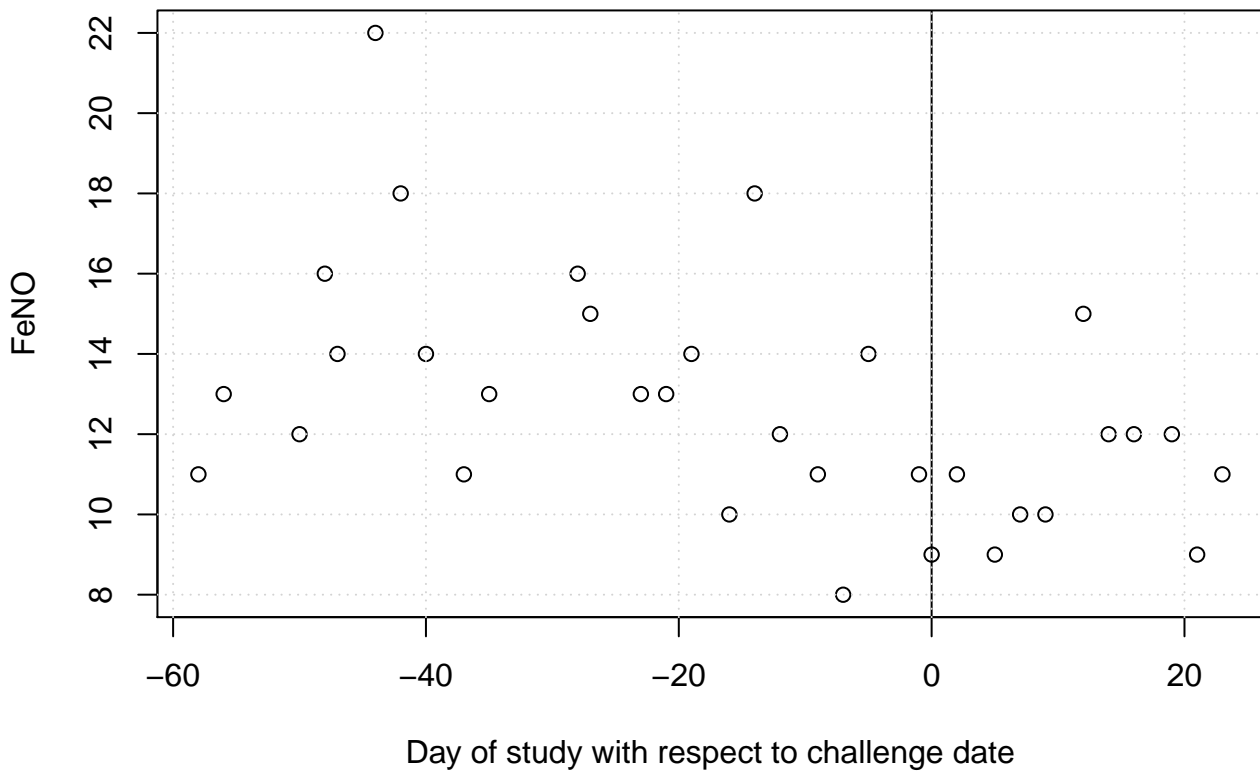

# P08H

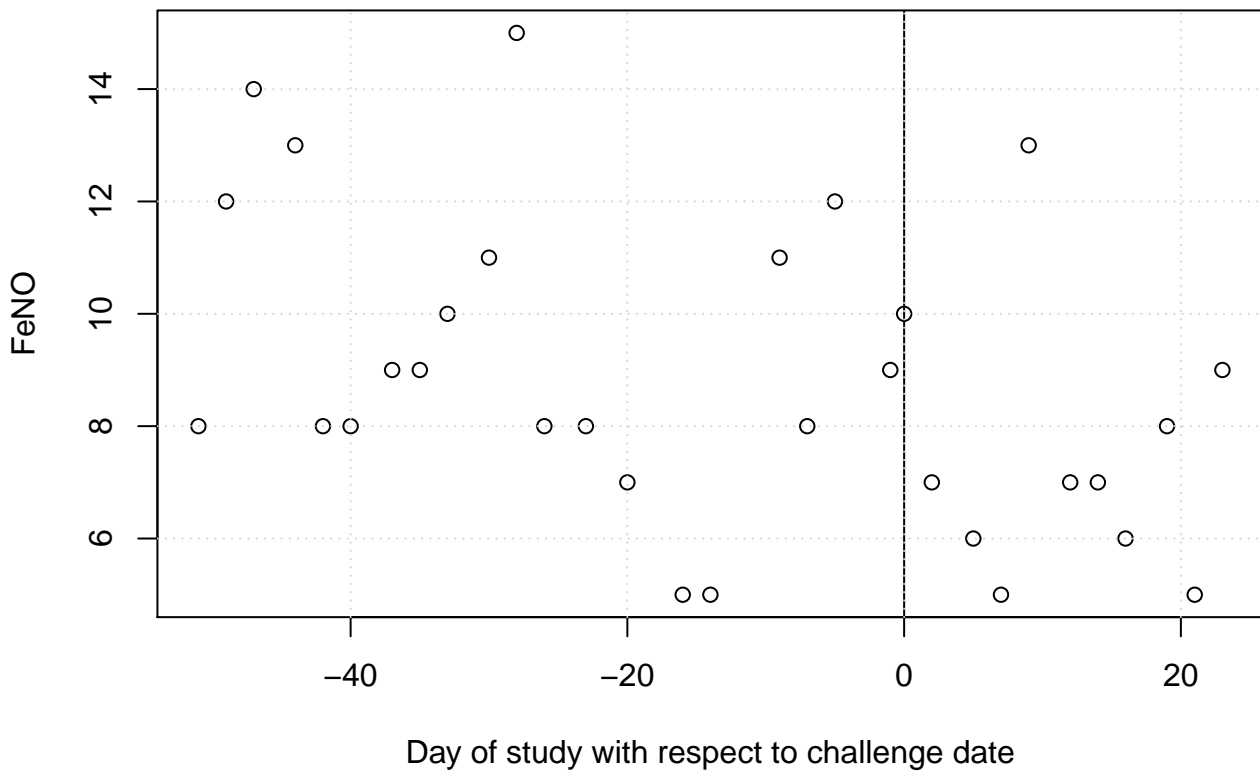

**P09H**

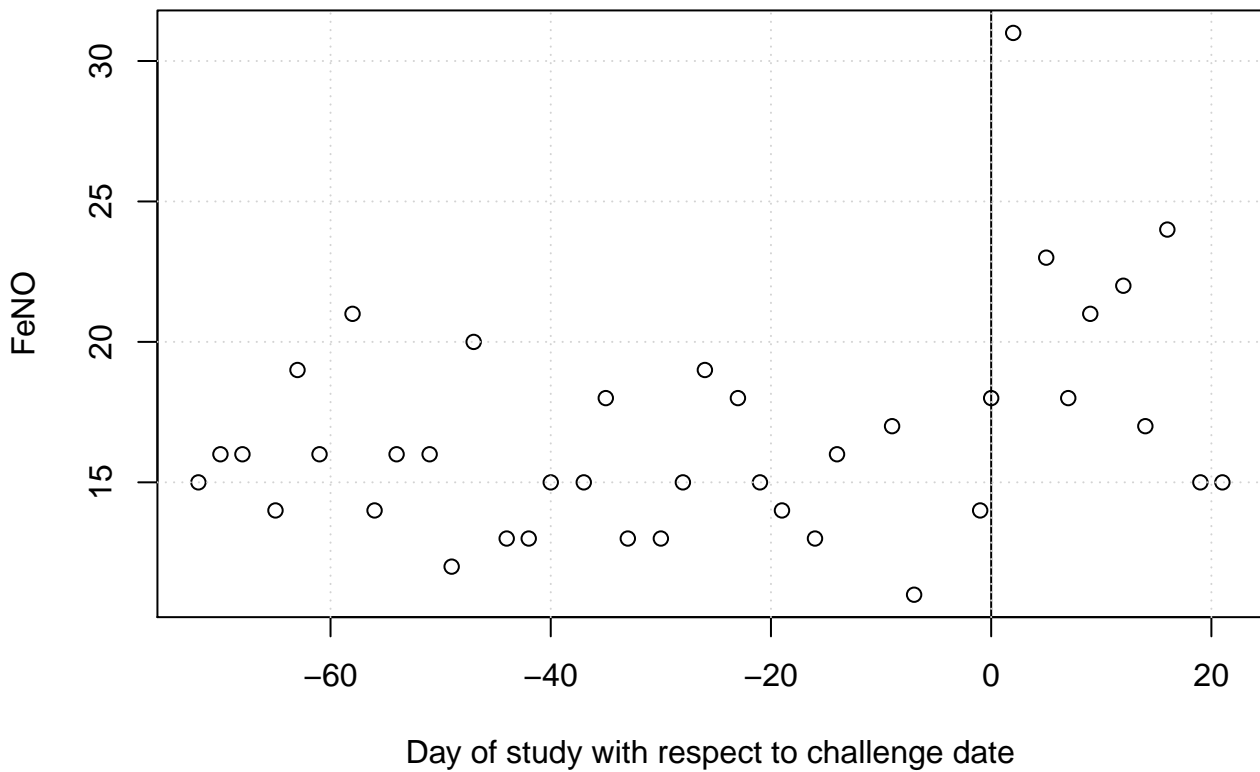

# P11H

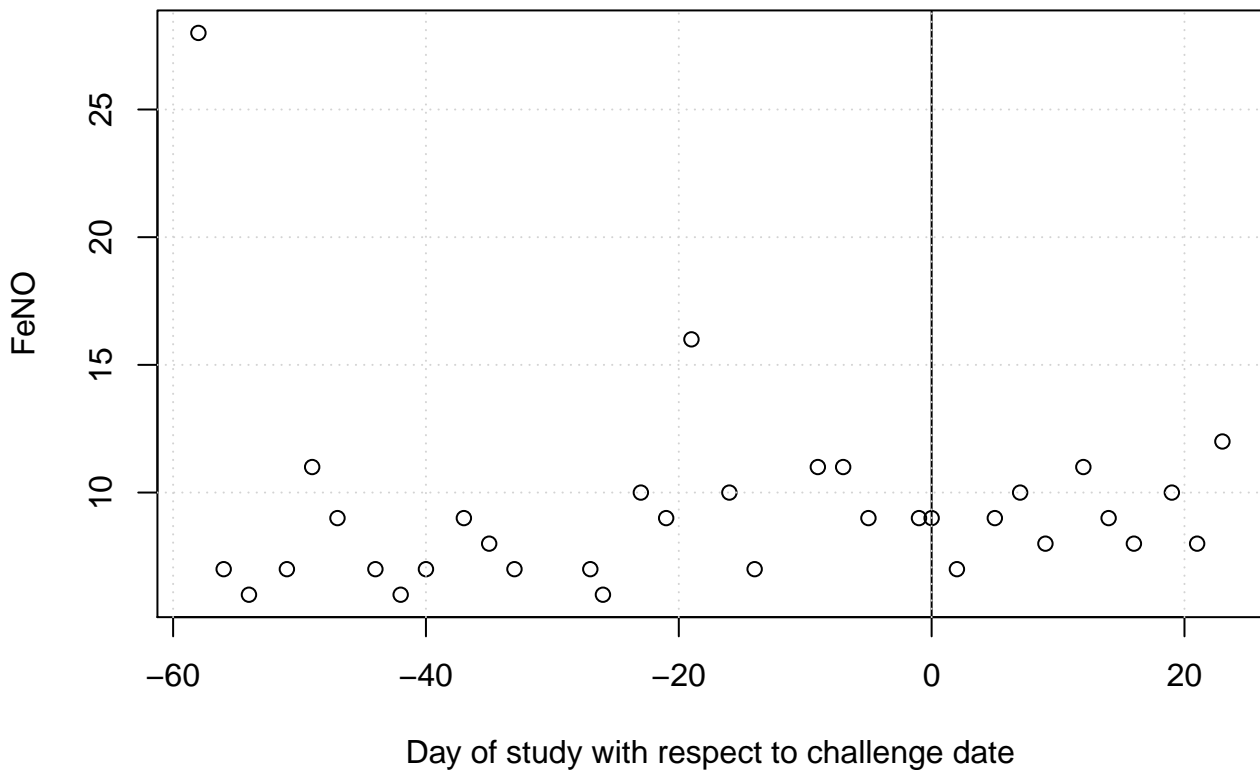

# P12H

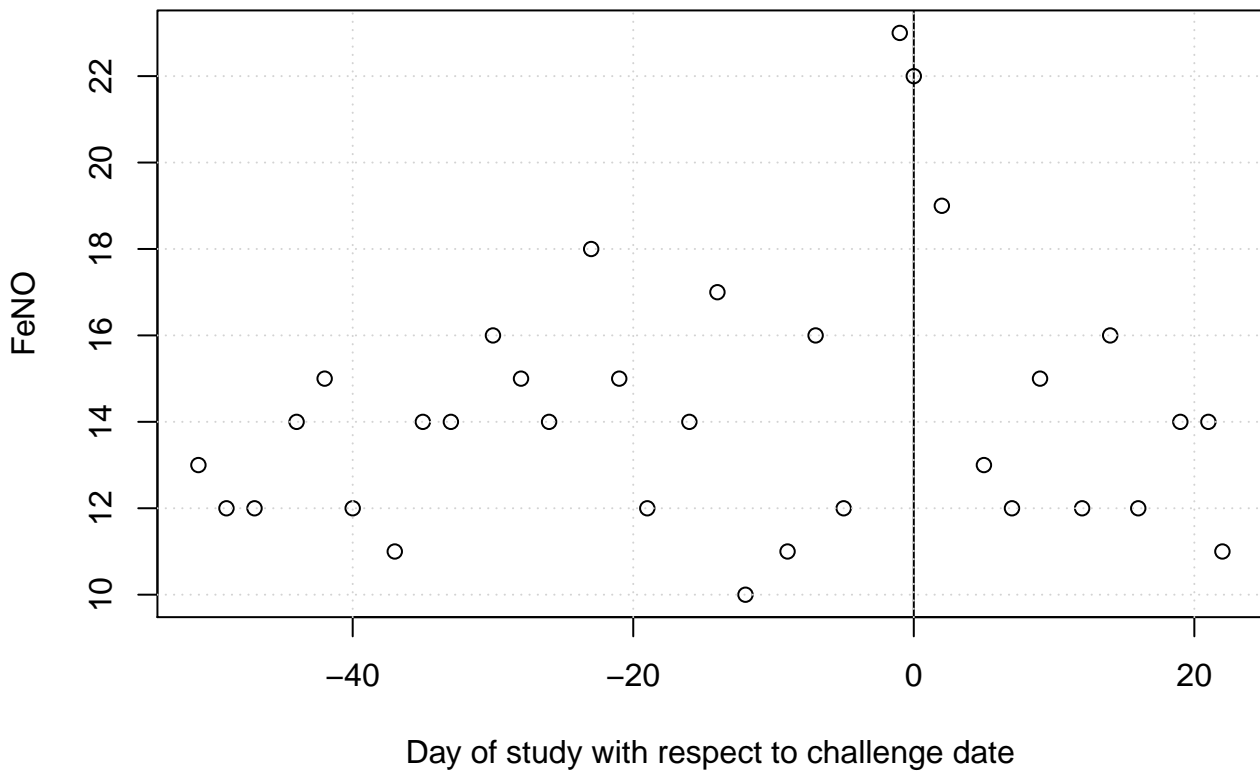

# P13H

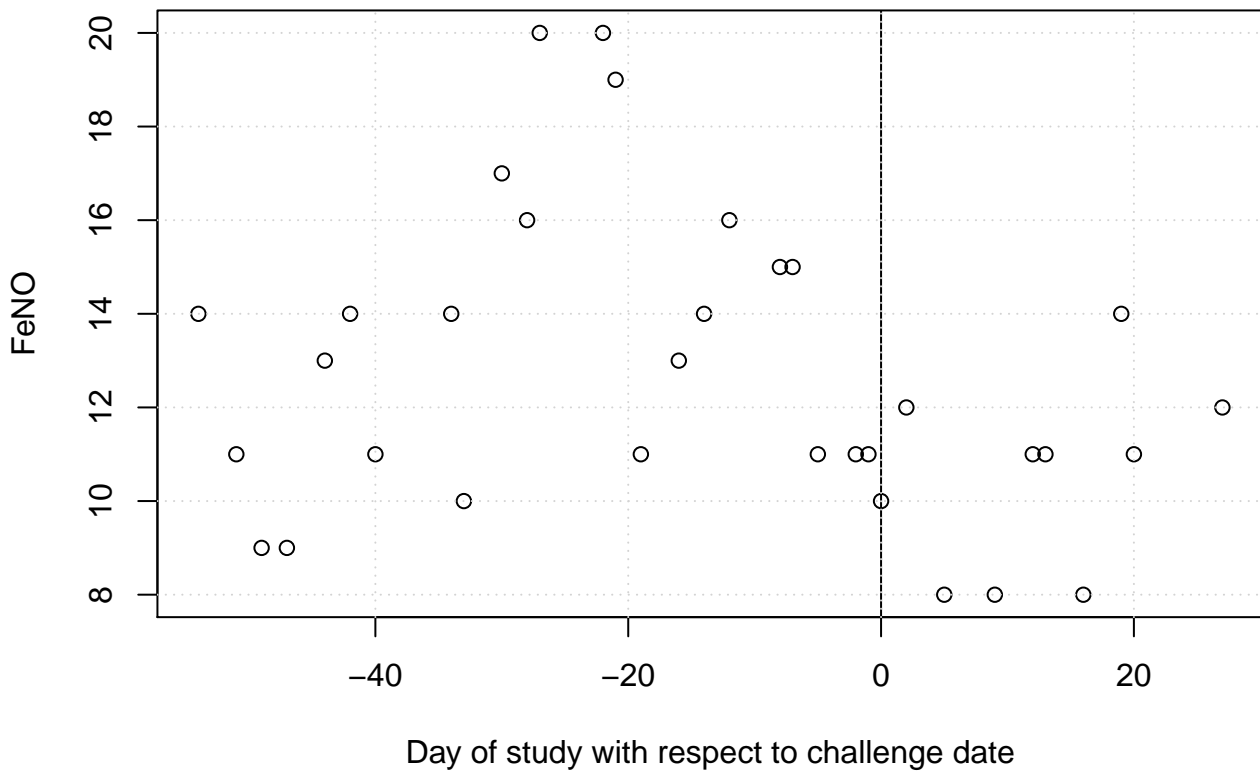

# P14H

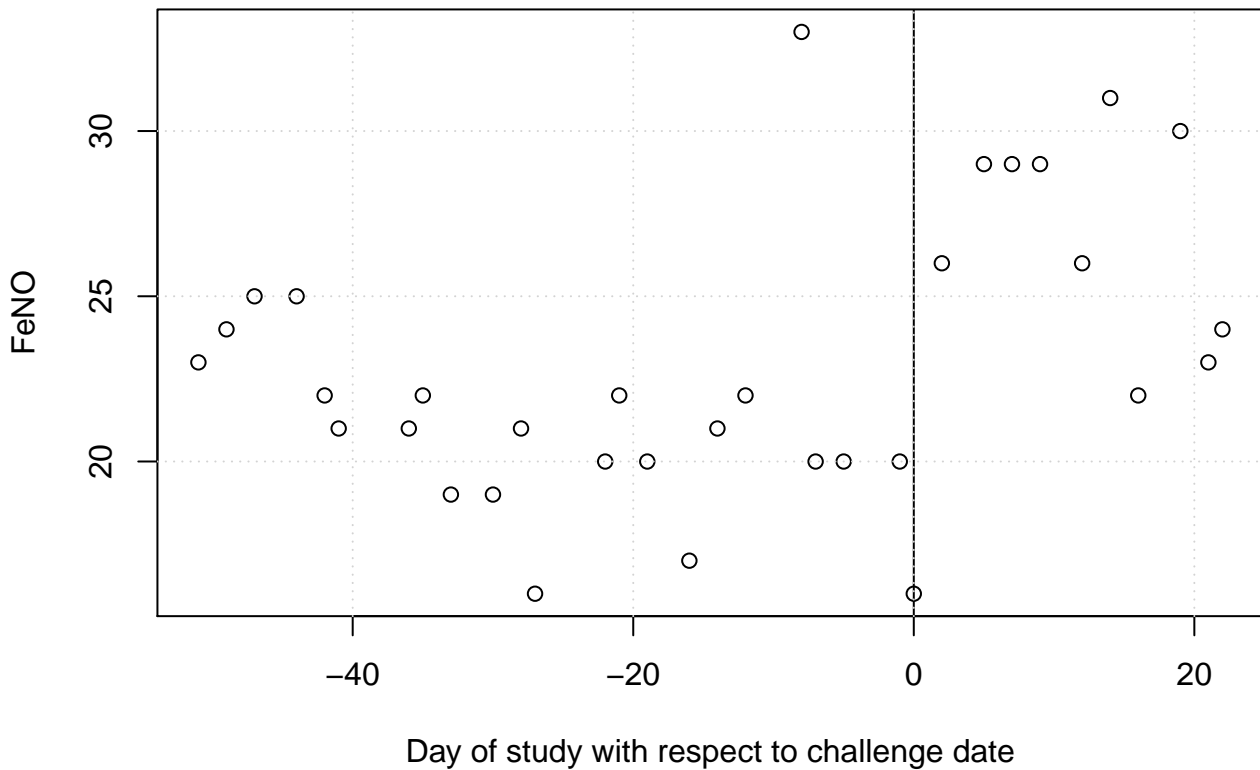

# P15H

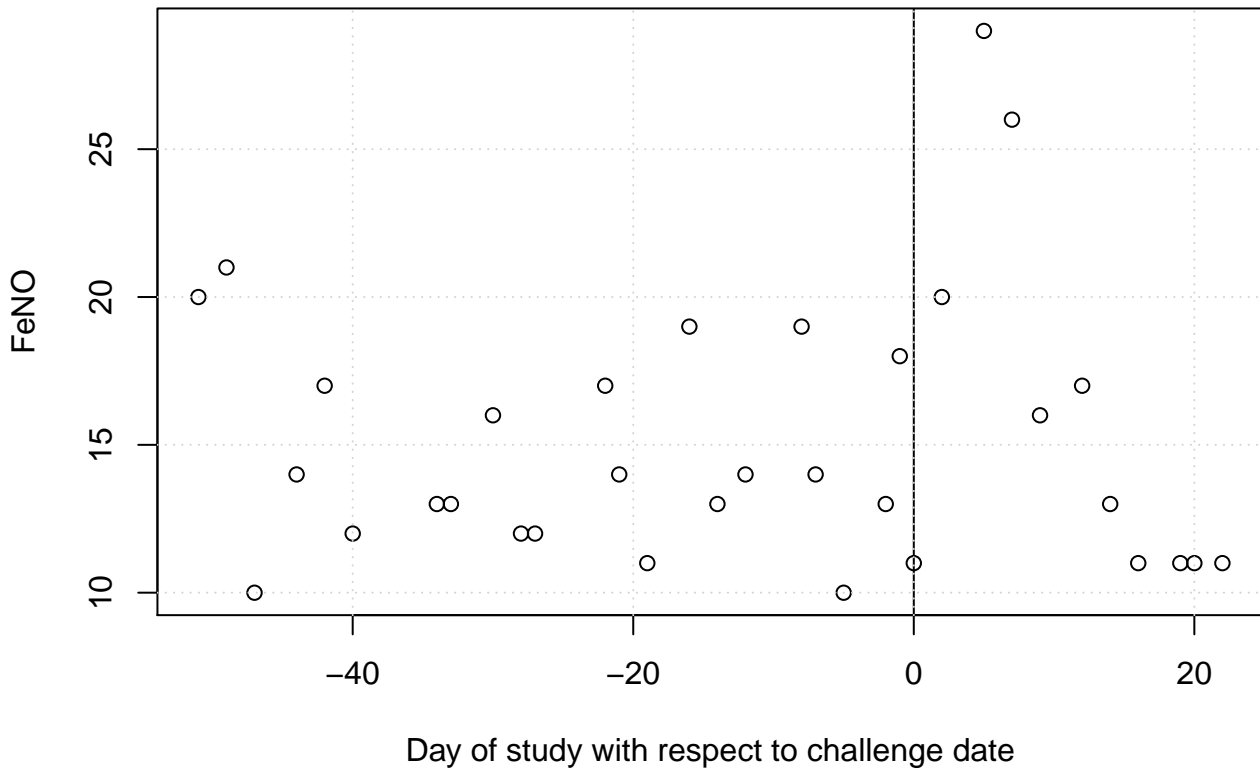

Supplement: Supplementary file 2. [file elife-47969-supp2.zip › TimeSeriesPlots_PDFs/Appendix-figure SS9_FeNO_TimeSeries_HealthyParticipants.pdf]

# P01H

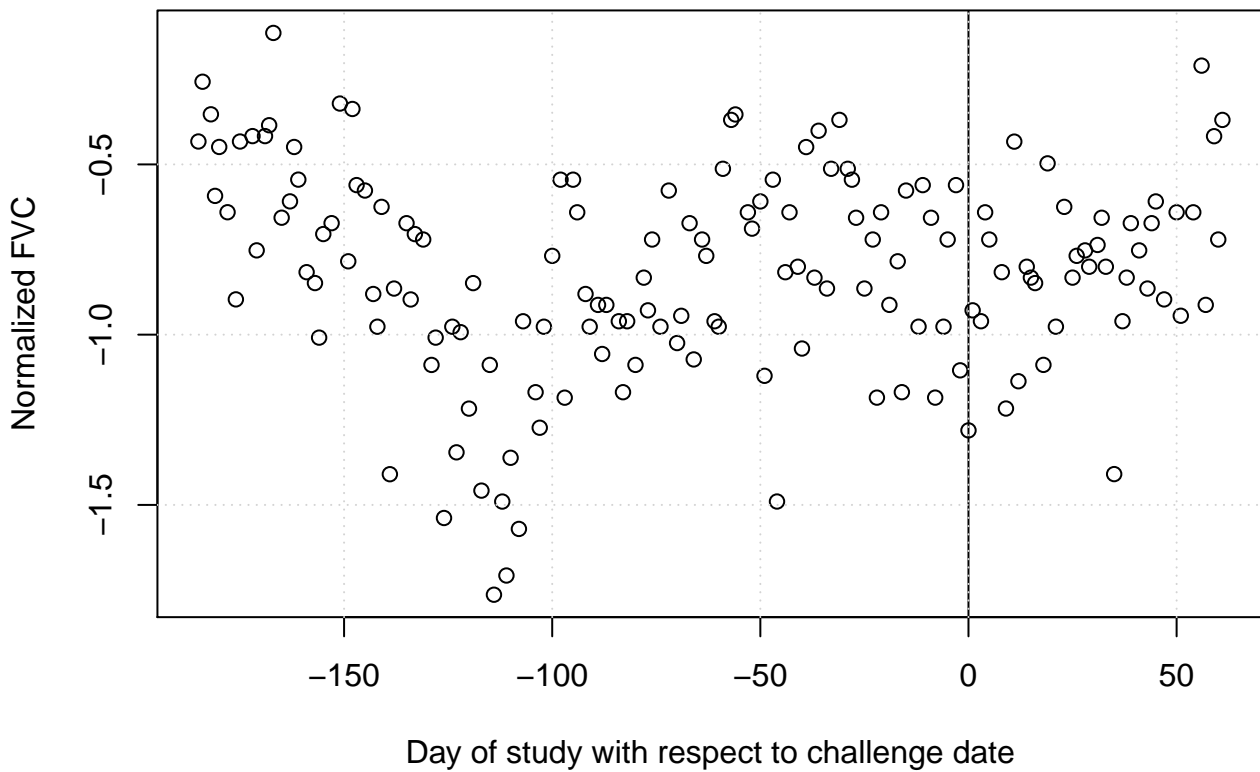

# P03H

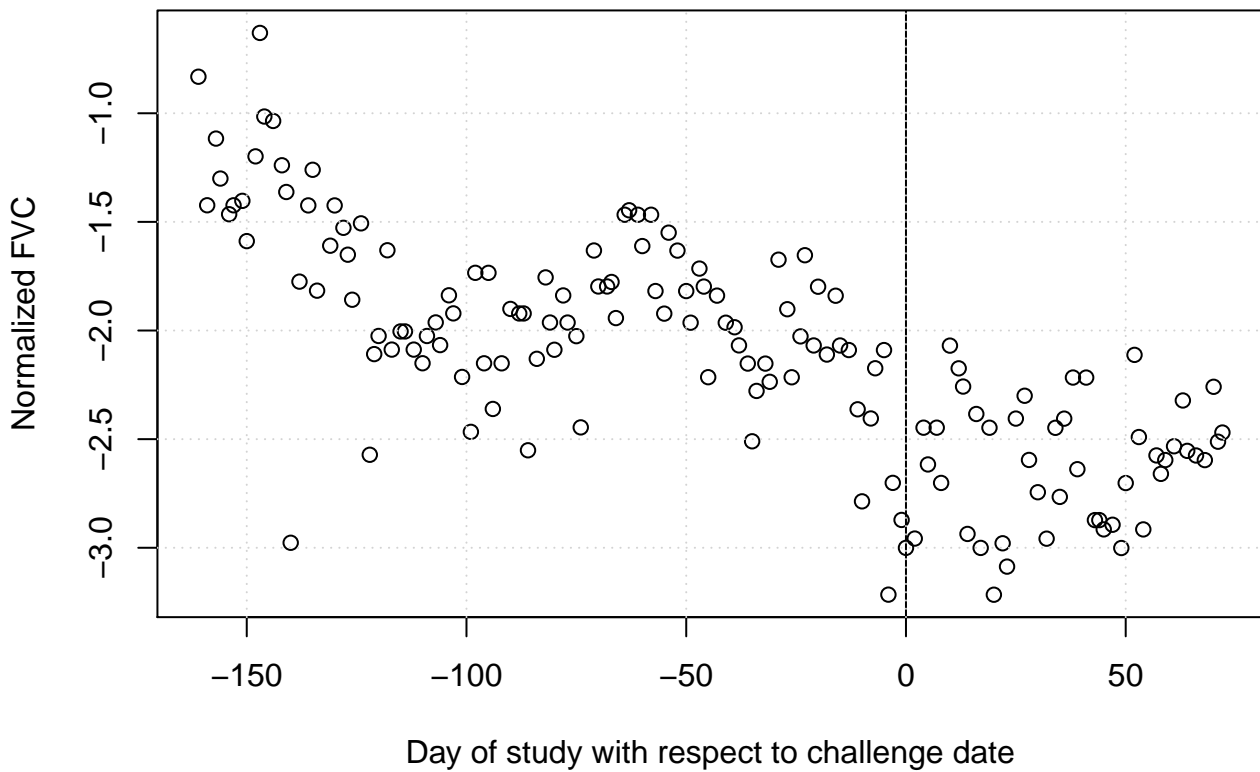

# P05H

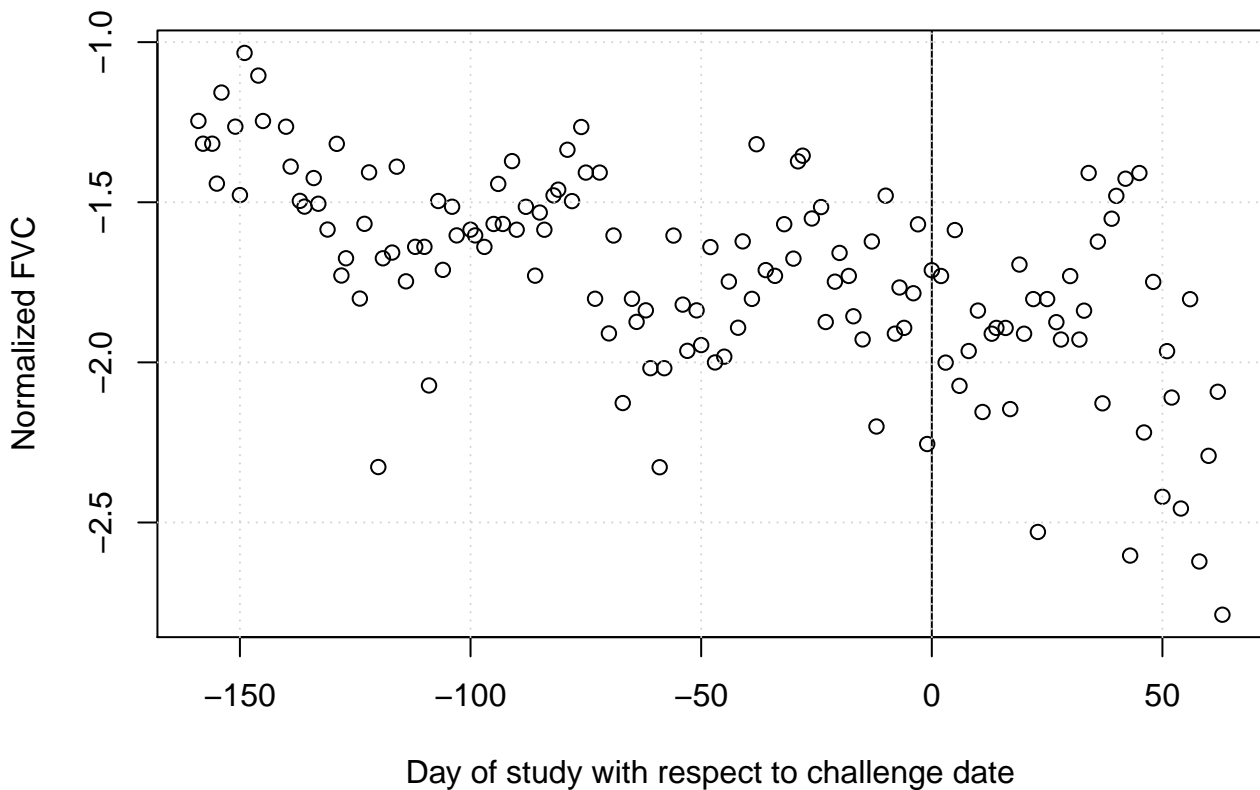

**P06H**

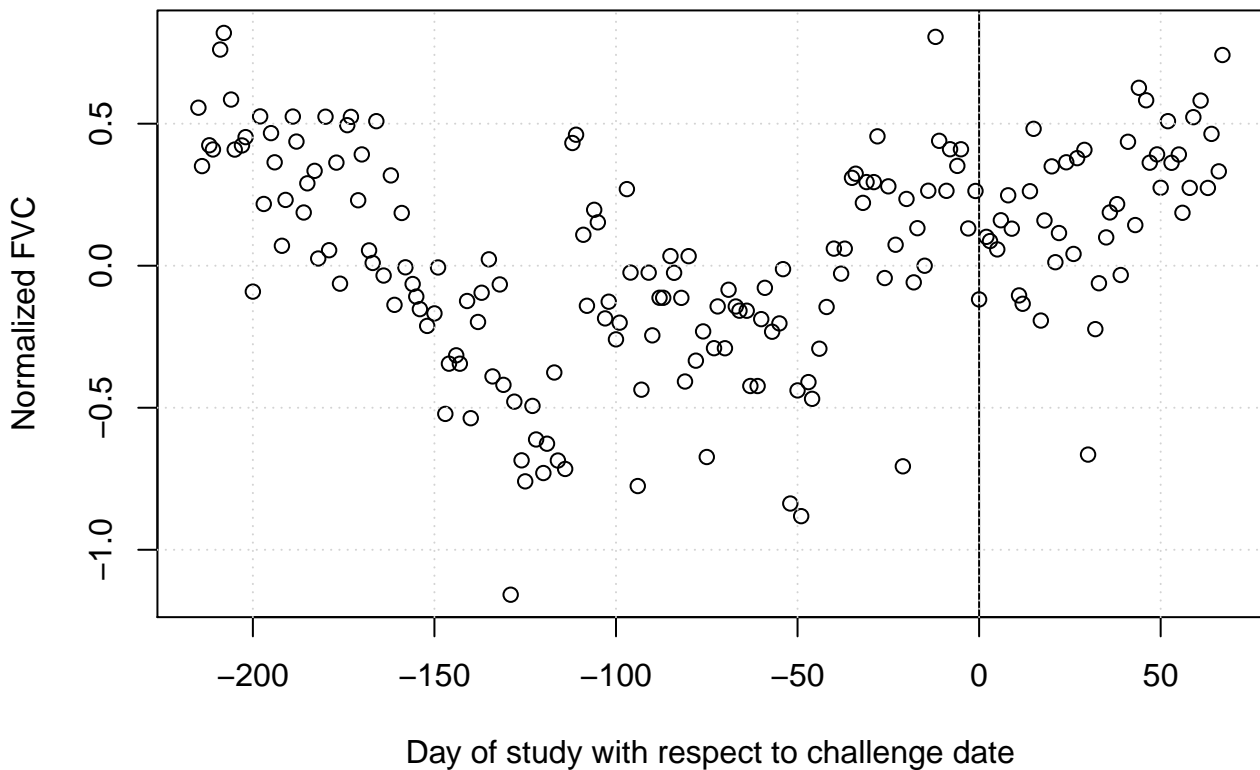

# P07H

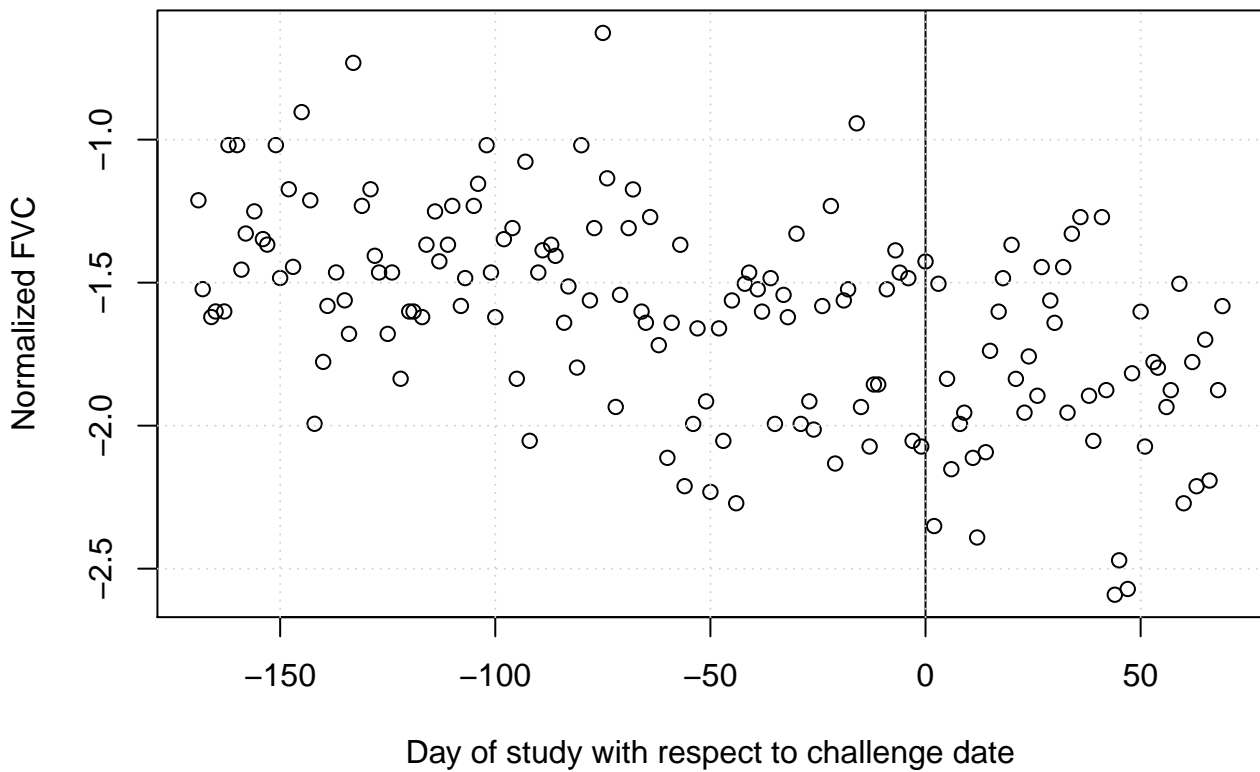

**P08H**

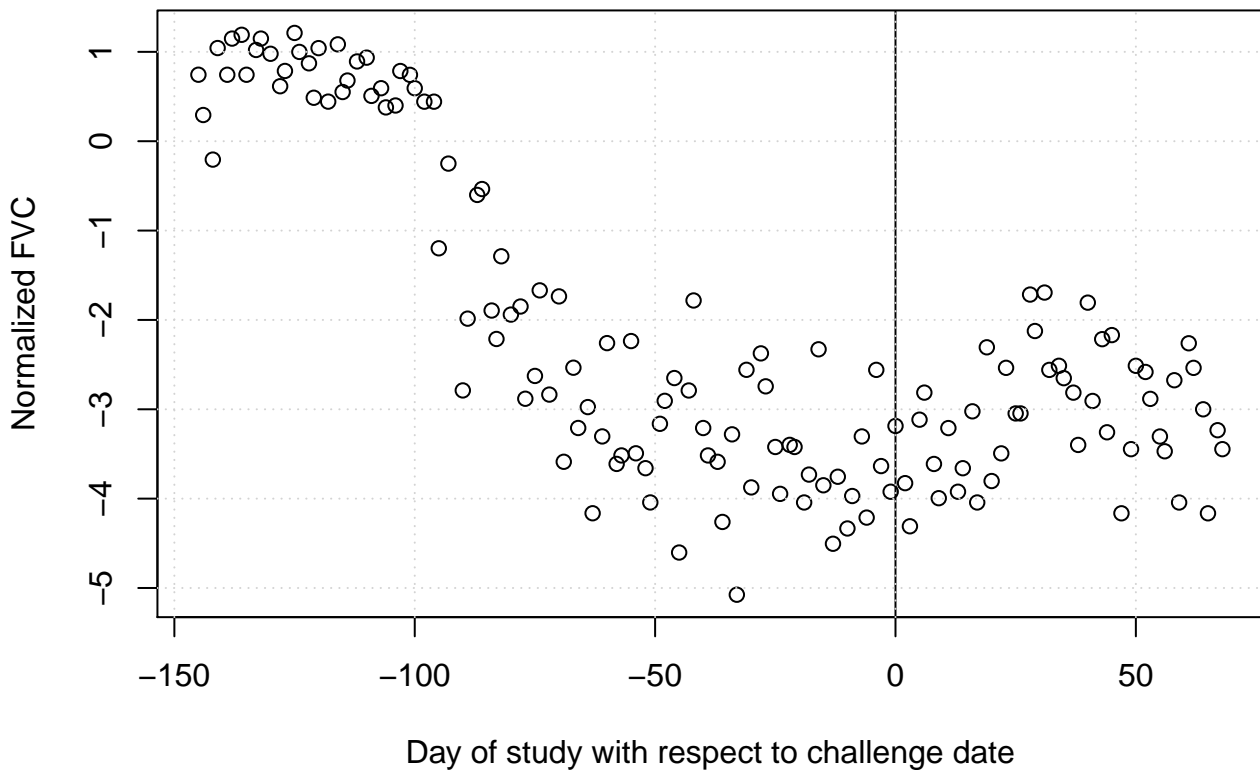

# P09H

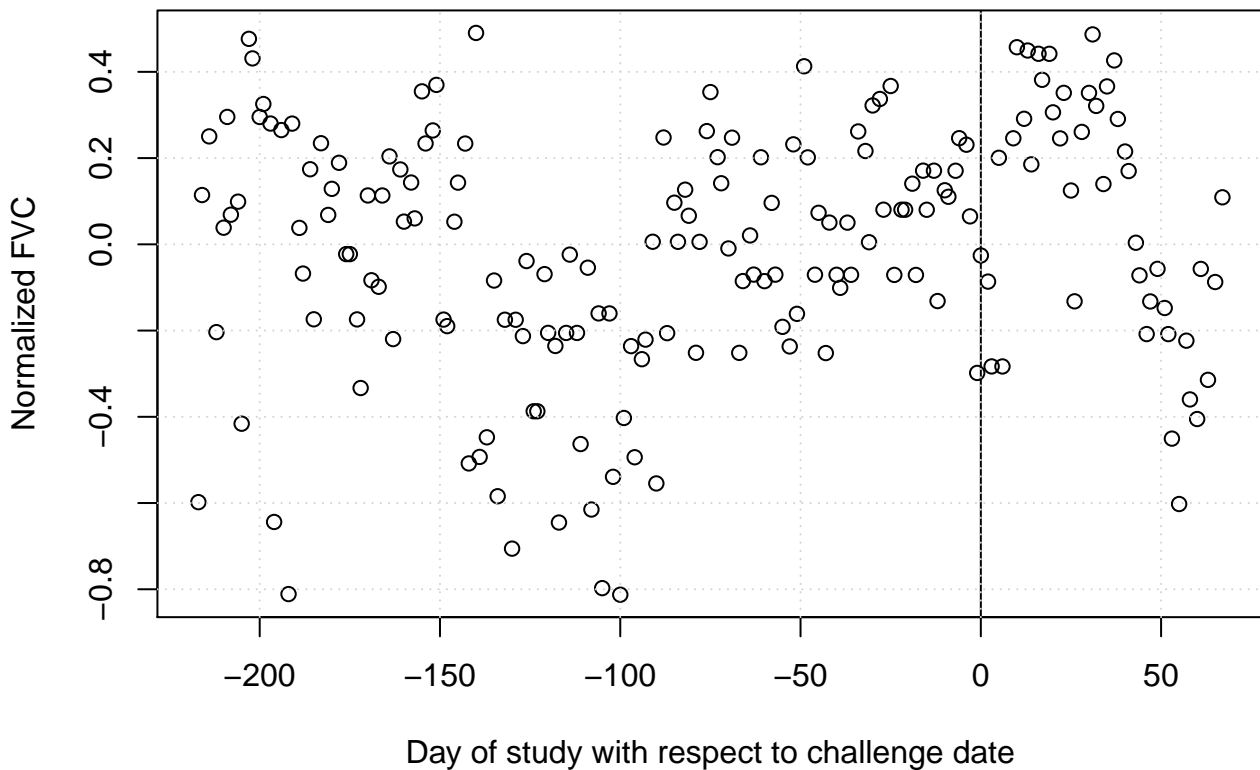

# P11H

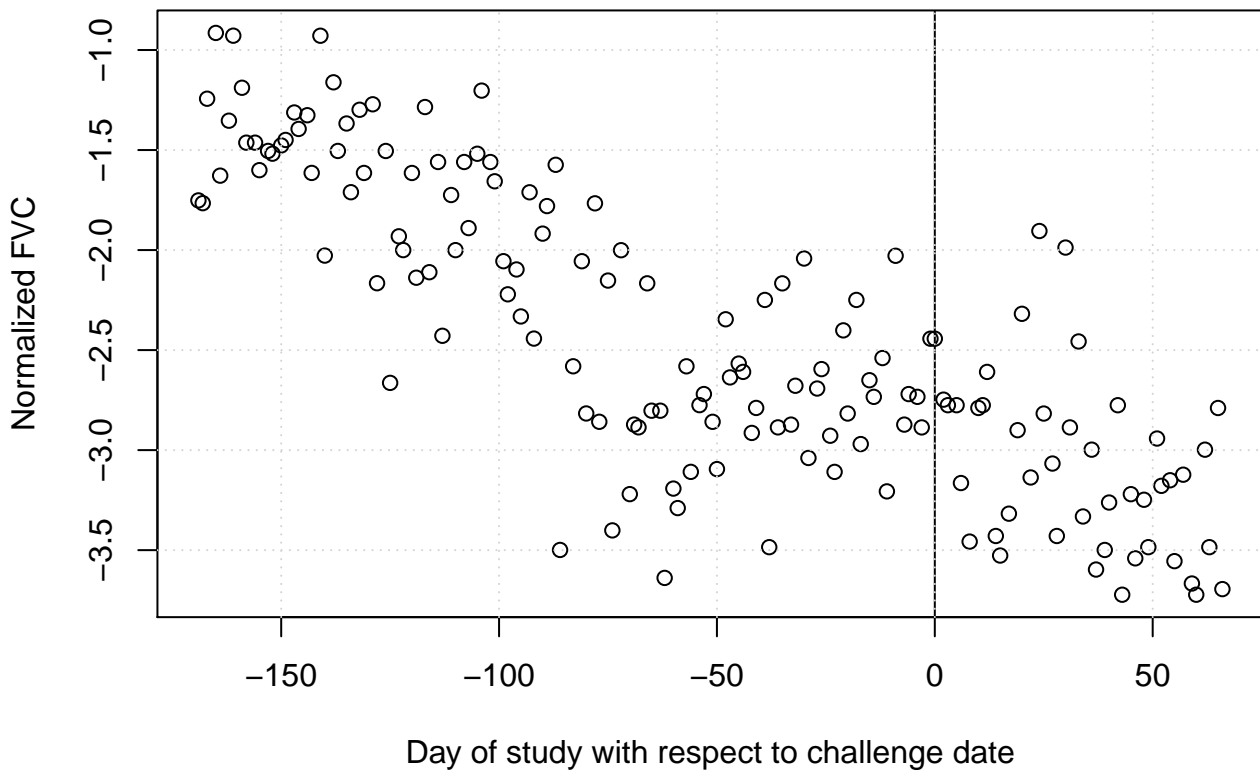

# P12H

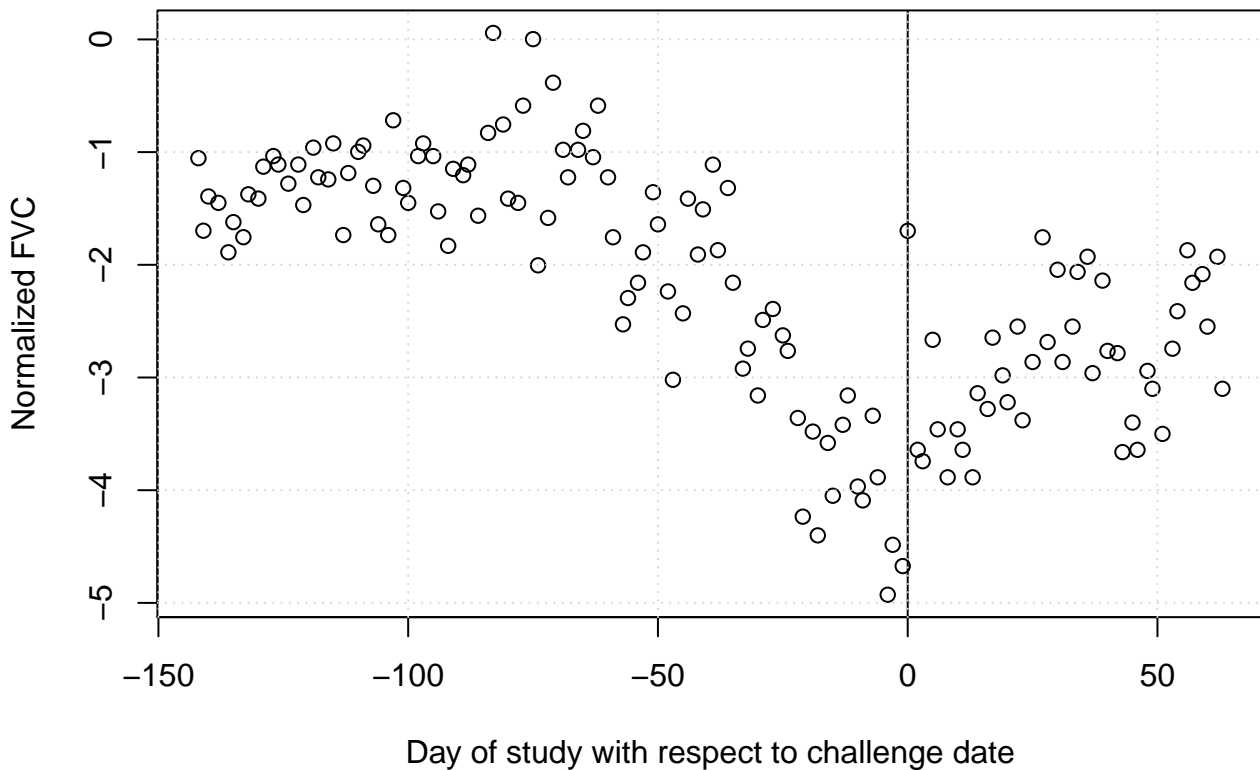

# P13H

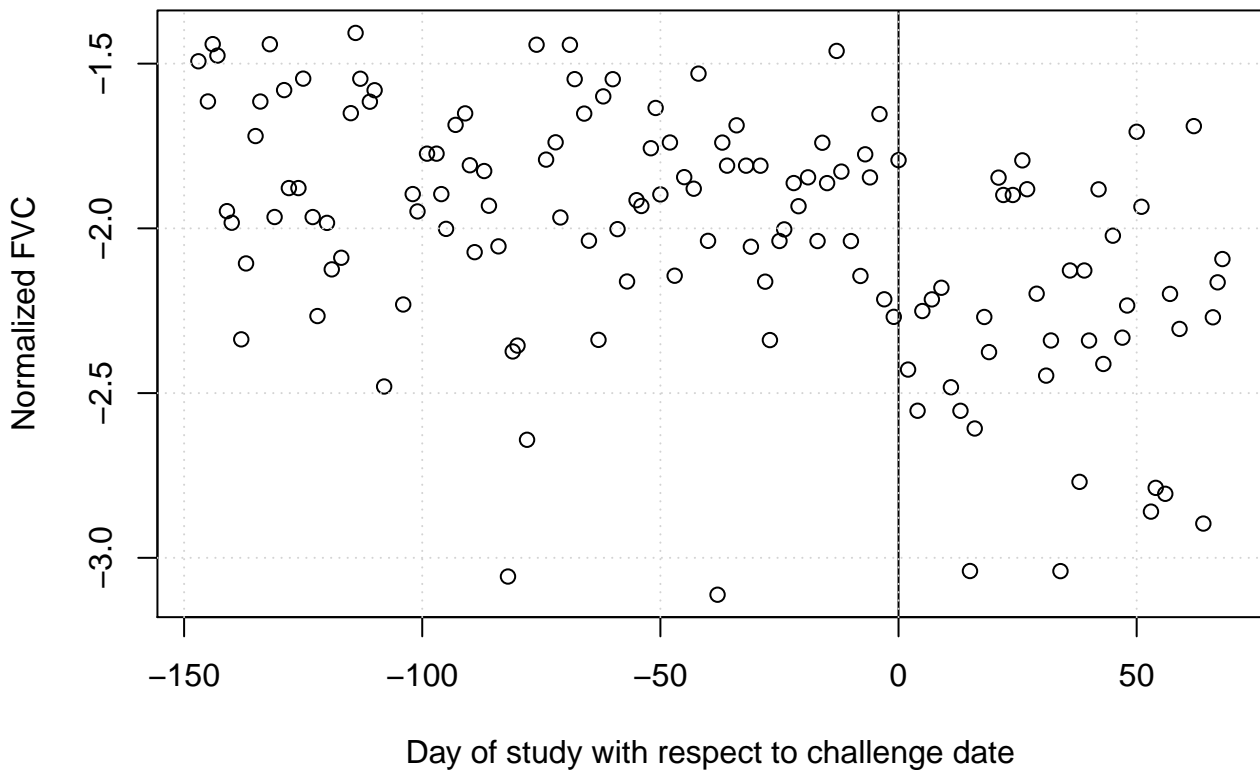

# P14H

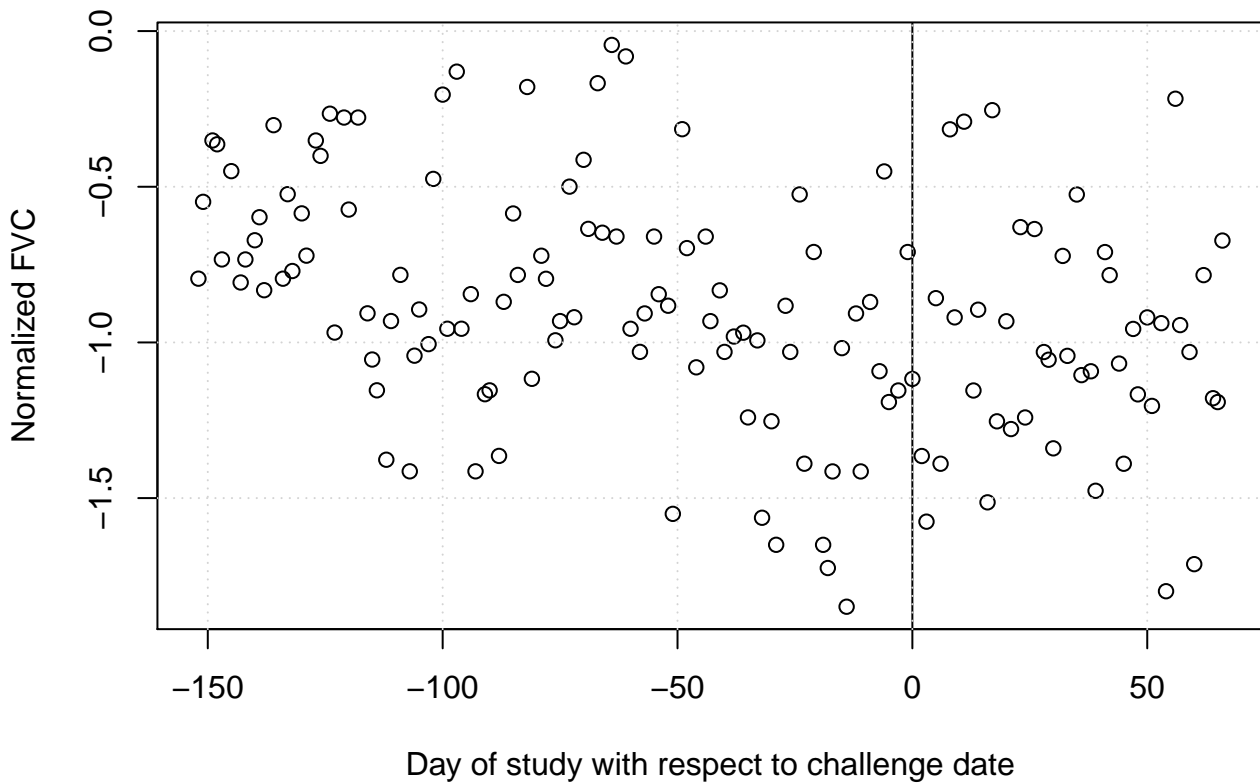

# P15H

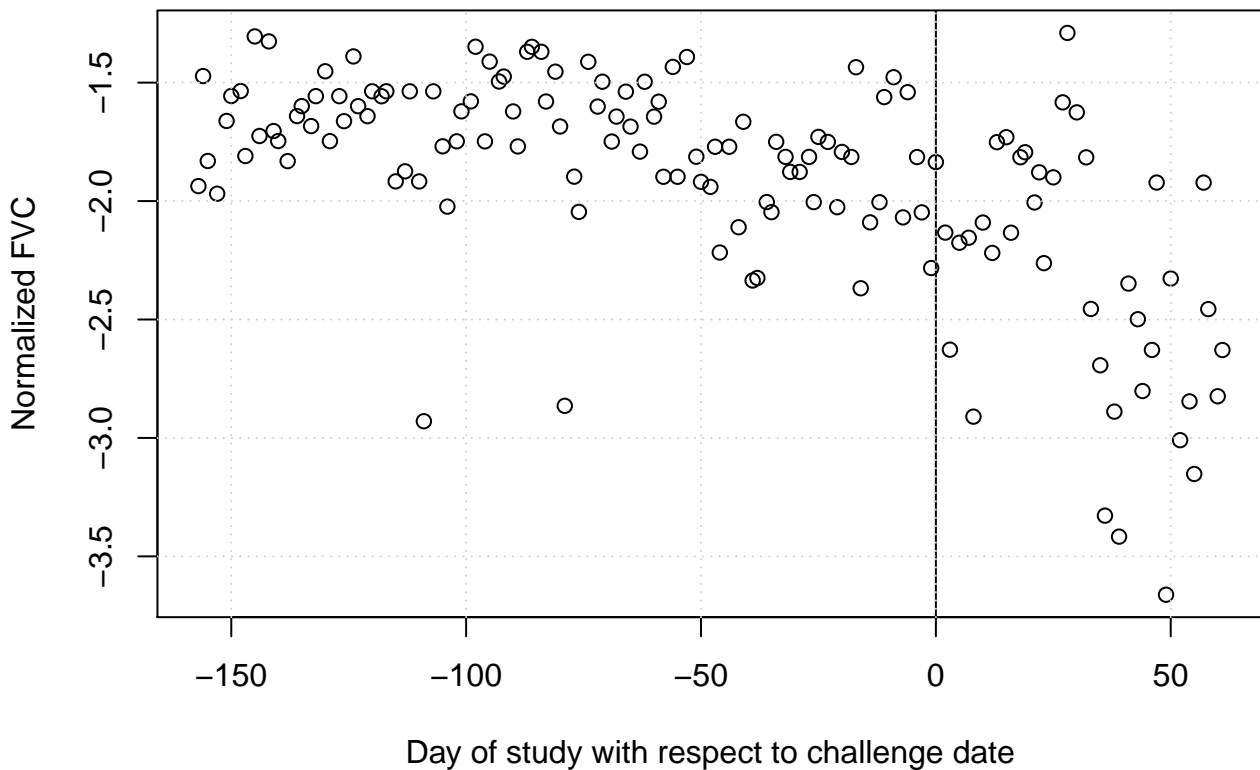

Supplement: Supplementary file 2. [file elife-47969-supp2.zip › TimeSeriesPlots_PDFs/Appendix-figure SS5_NFVC_TimeSeries_HealthyParticipants.pdf]

# P01A

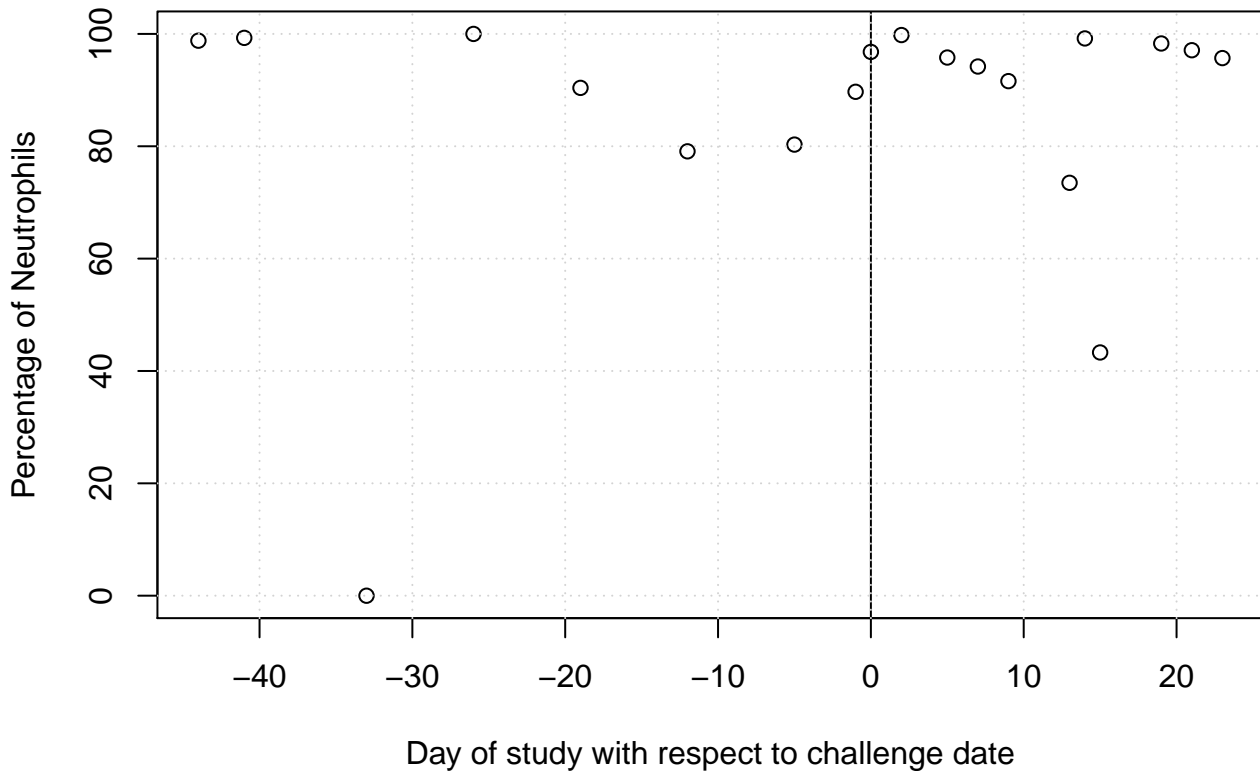

## P02A

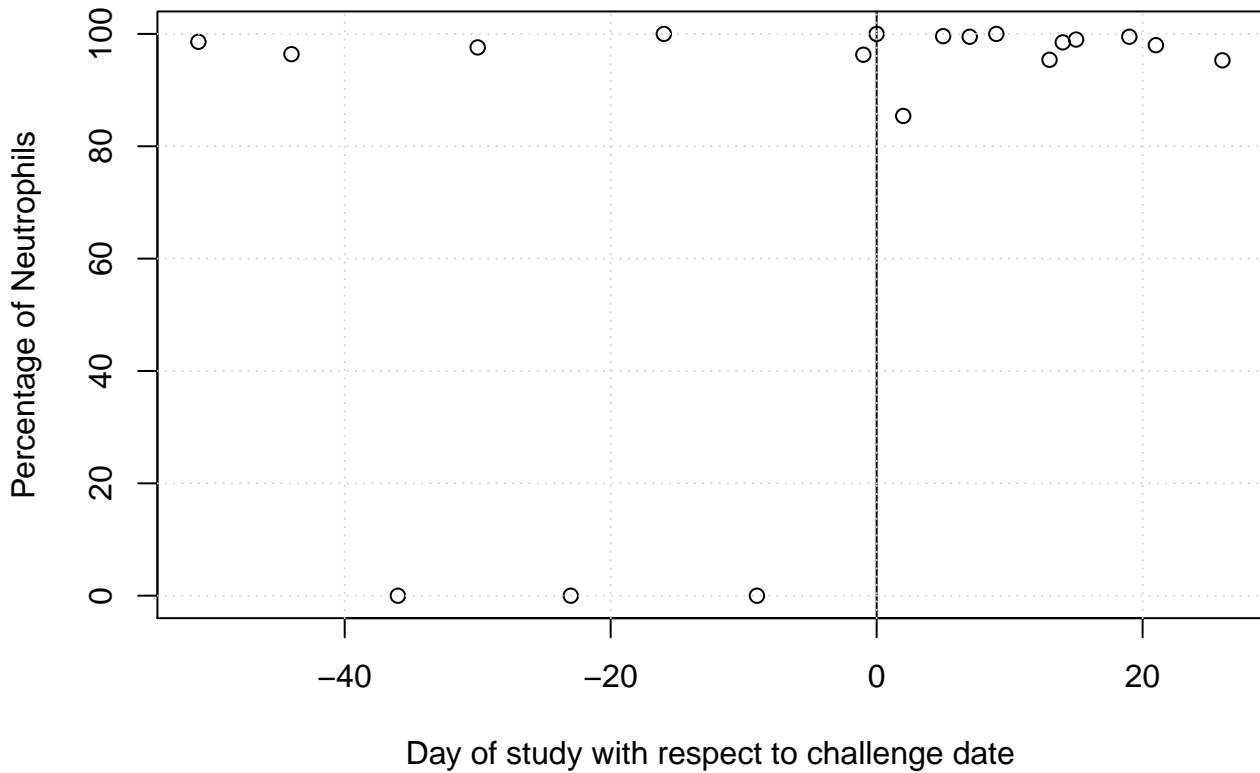

**P04A**

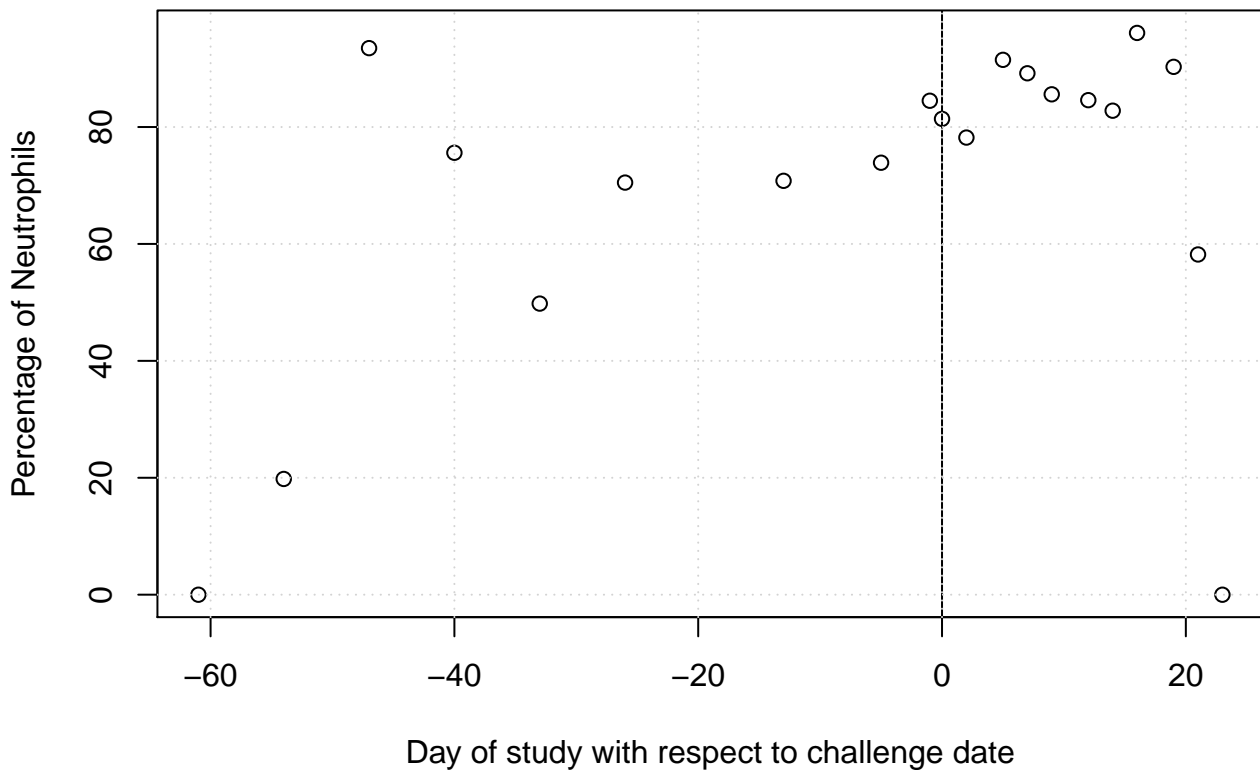

# P05A

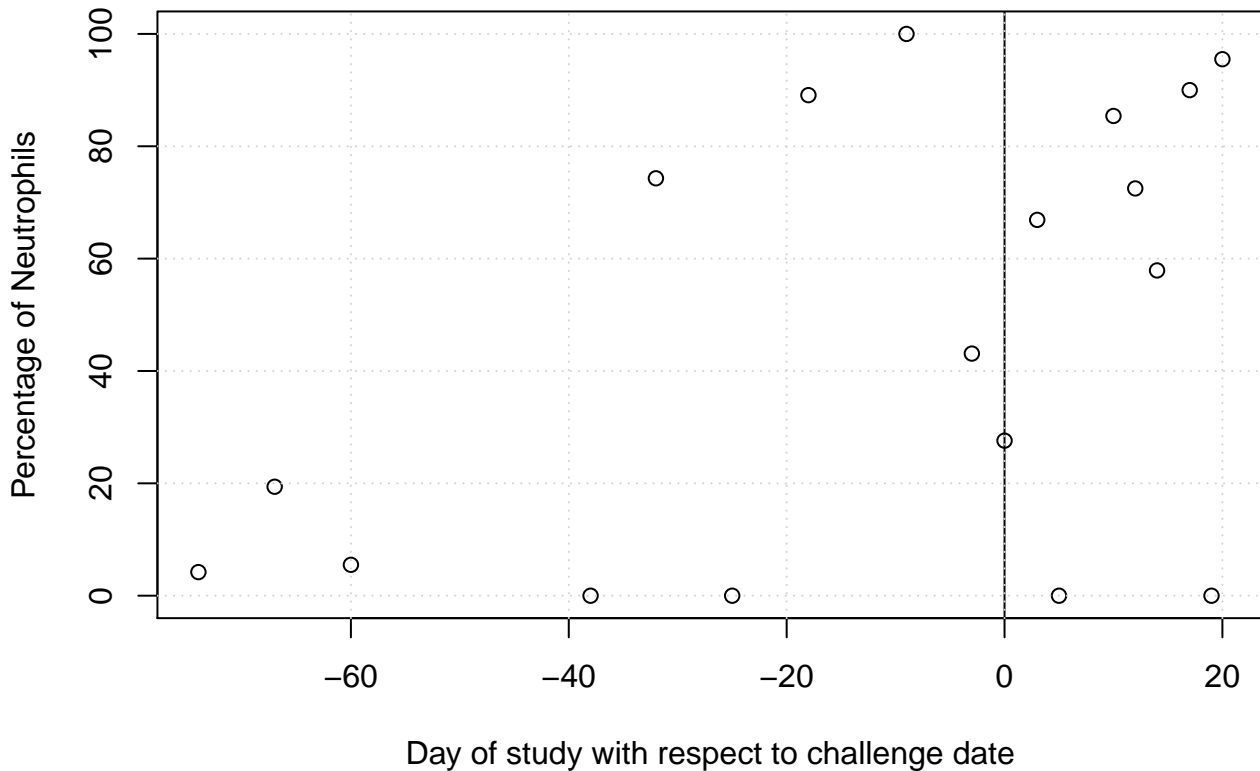

# P06A

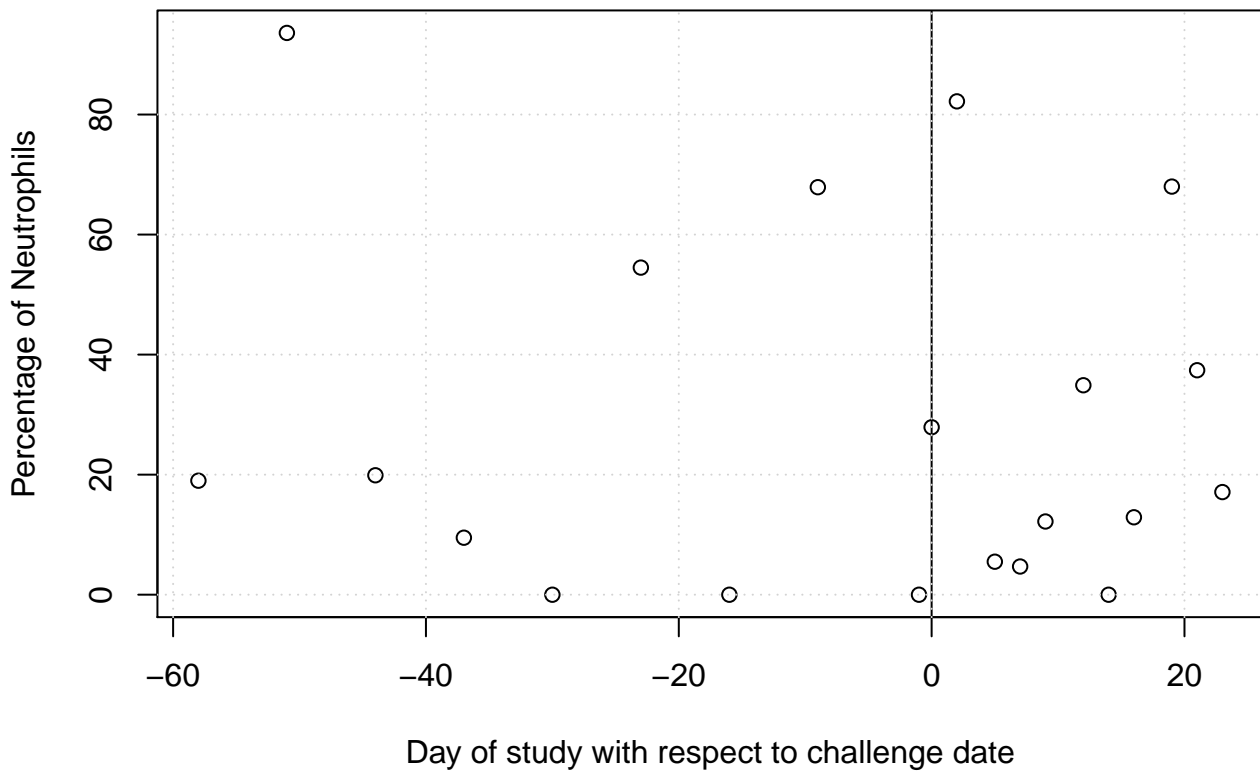

# P07A

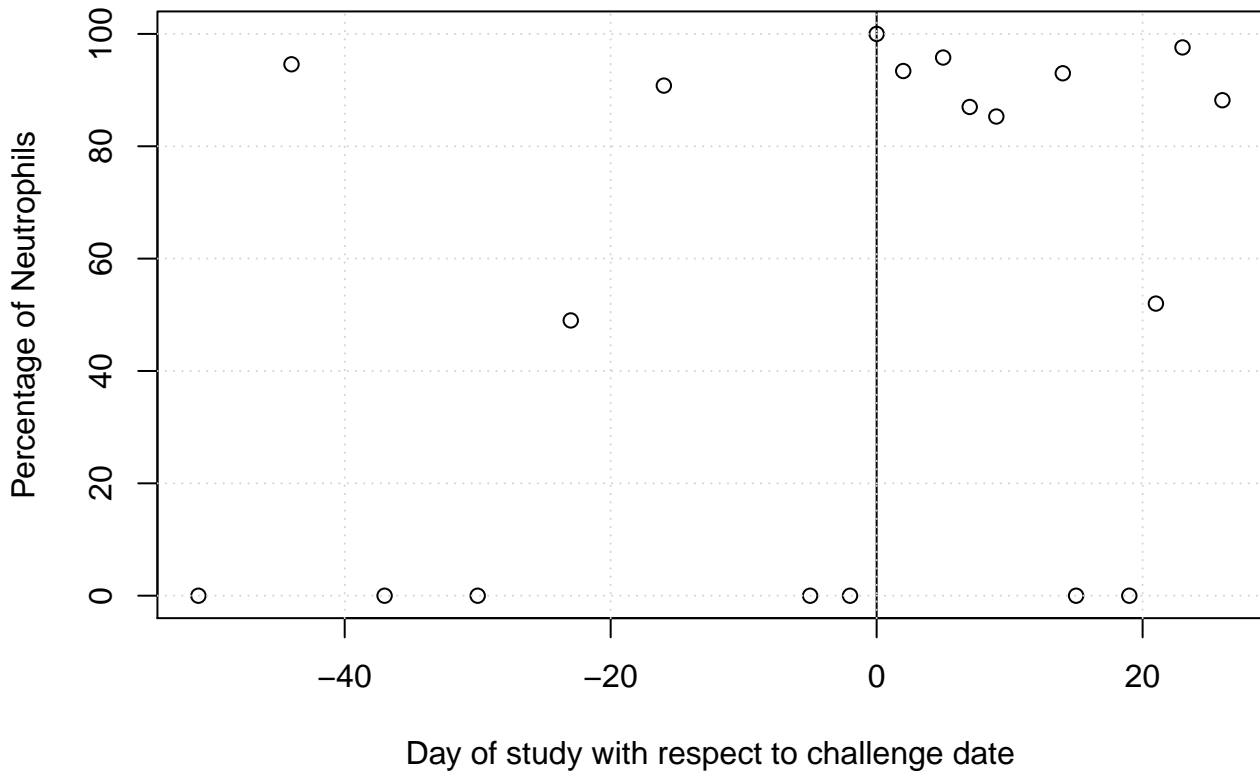

# P08A

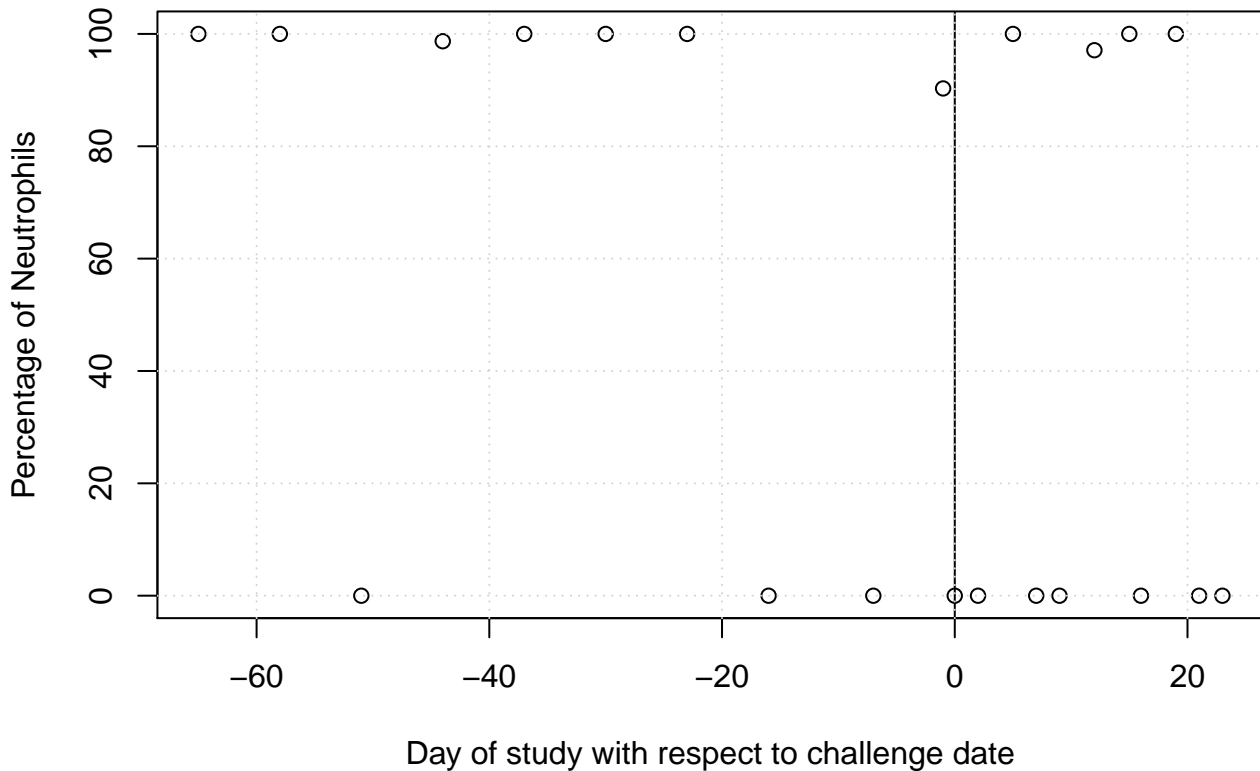

# P09A

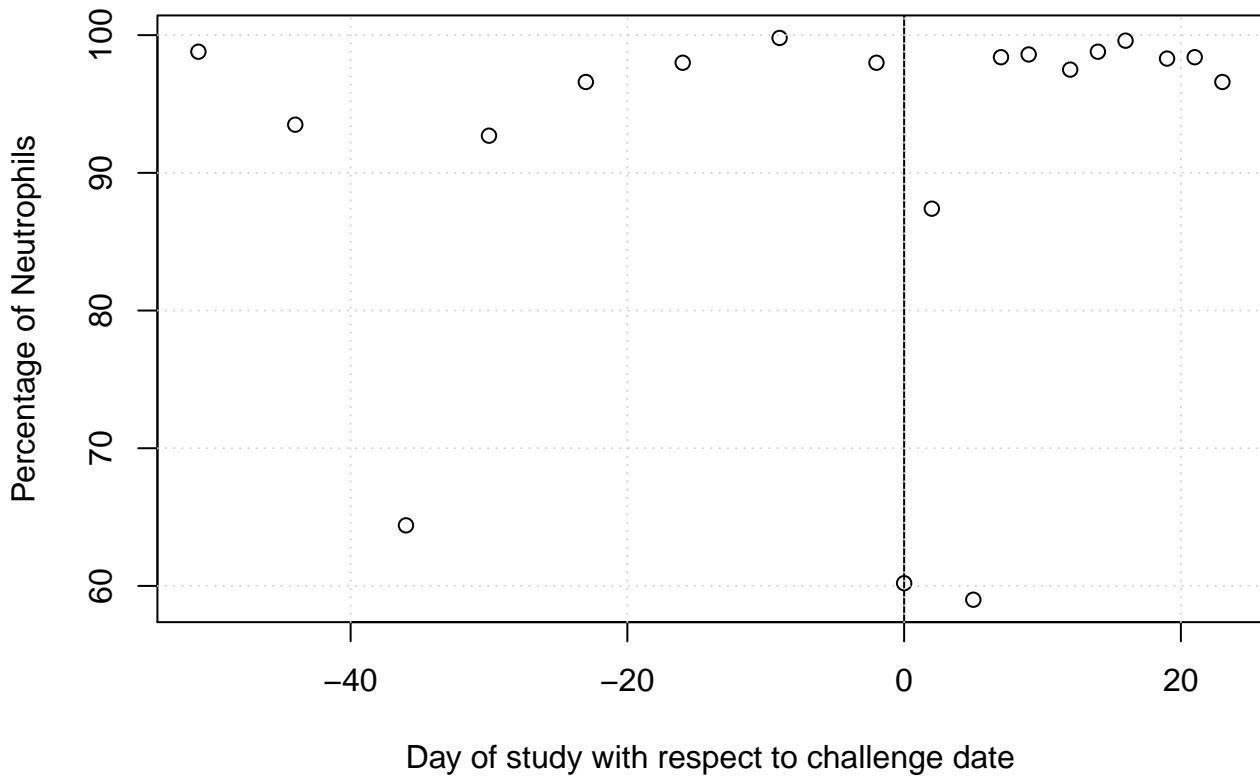

# P10A

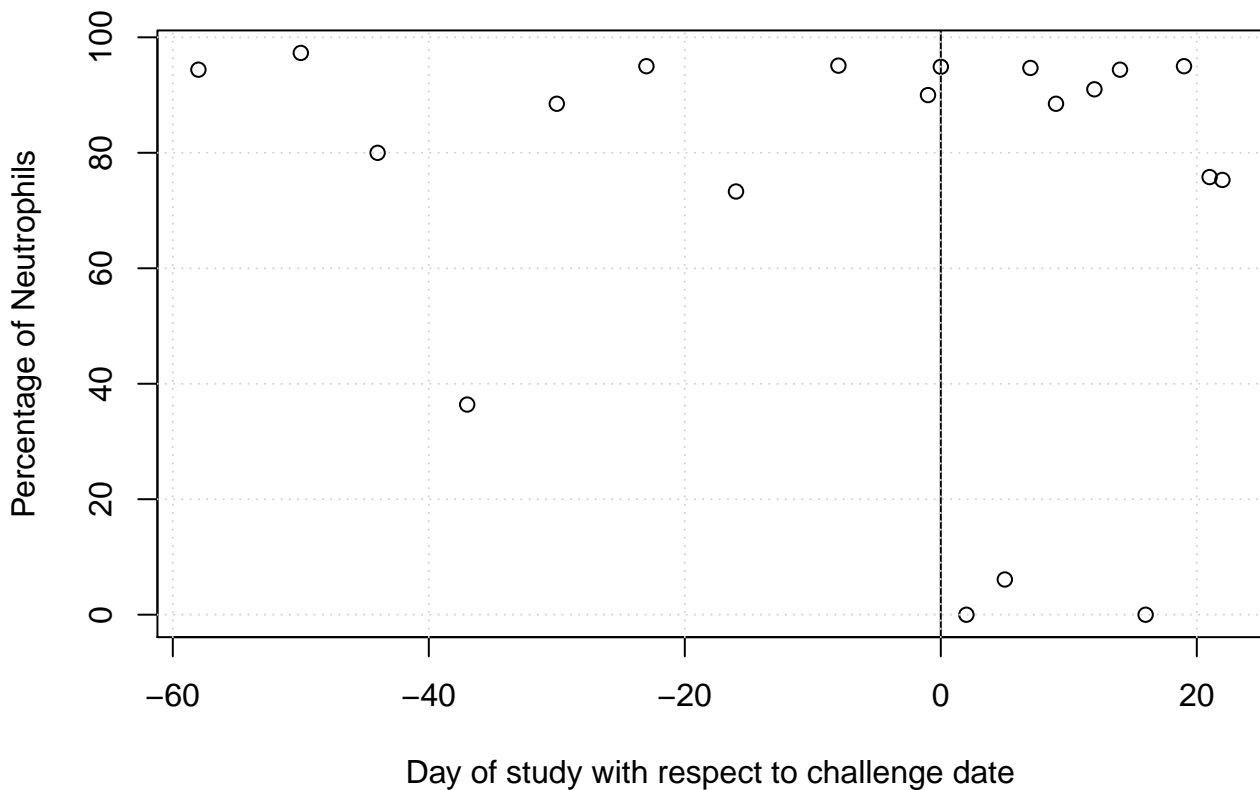

# P11A

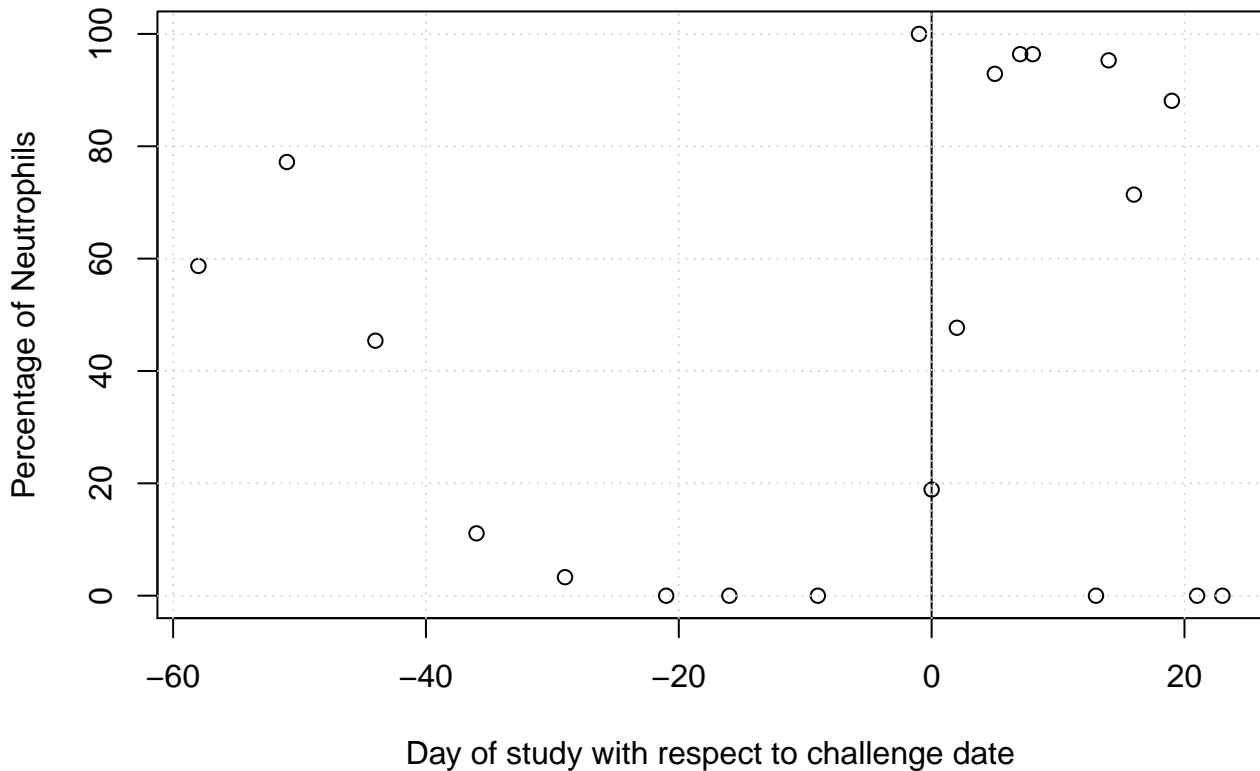

## P12A

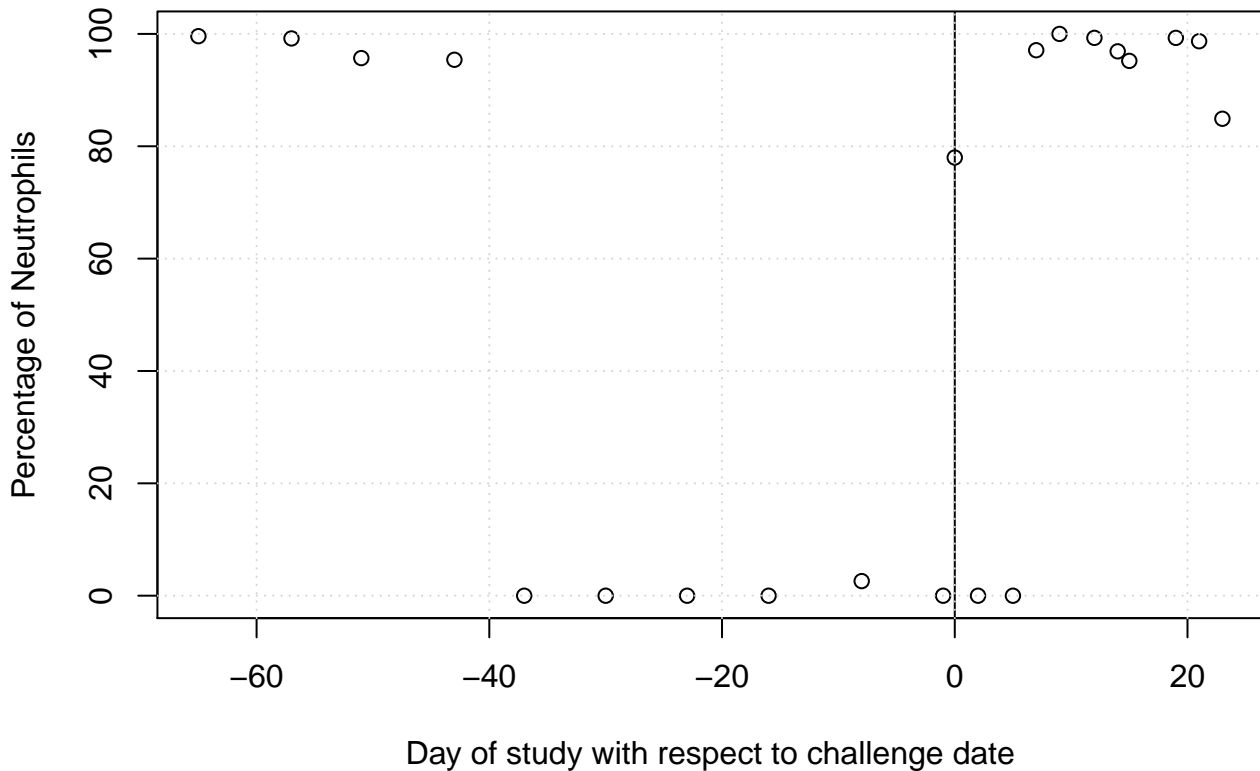

# P13A

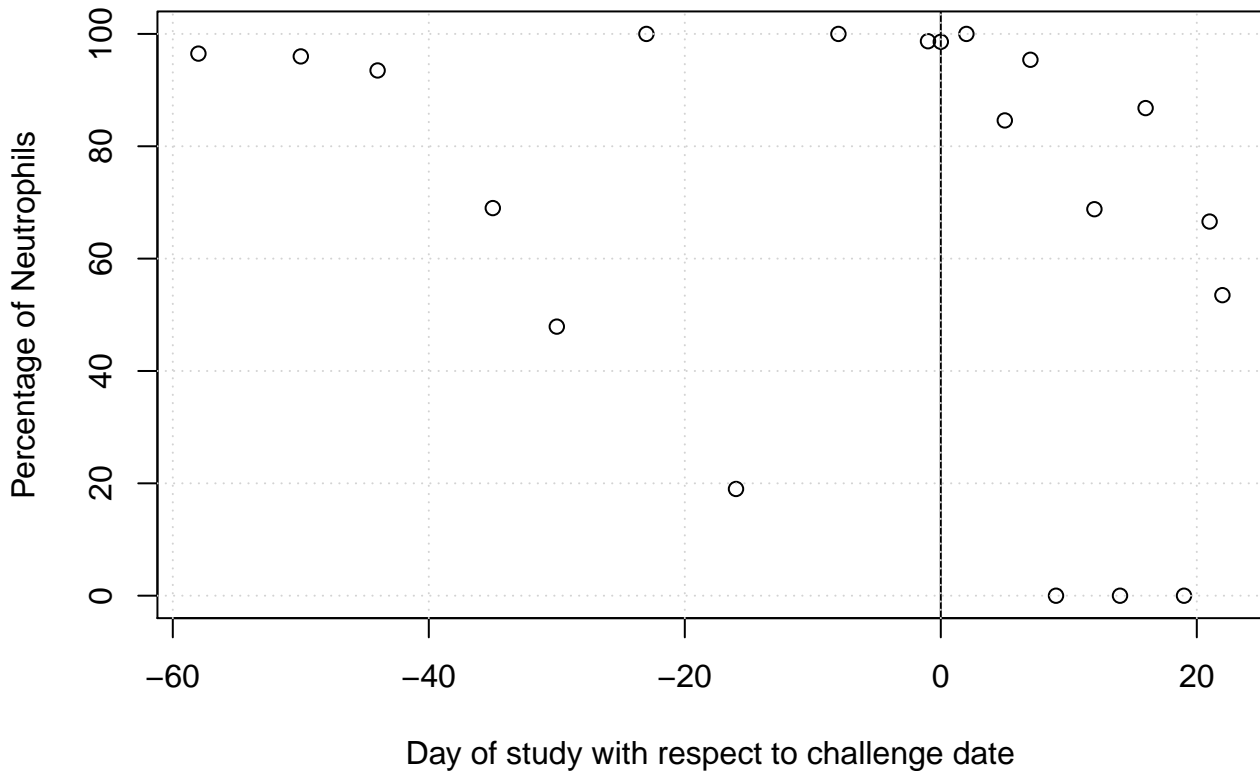

Supplement: Supplementary file 2. [file elife-47969-supp2.zip › TimeSeriesPlots_PDFs/Appendix-figure SS16_PercentageOfNeutrophils_TimeSeries_AsthmaticParticipants.pdf]

# P01A

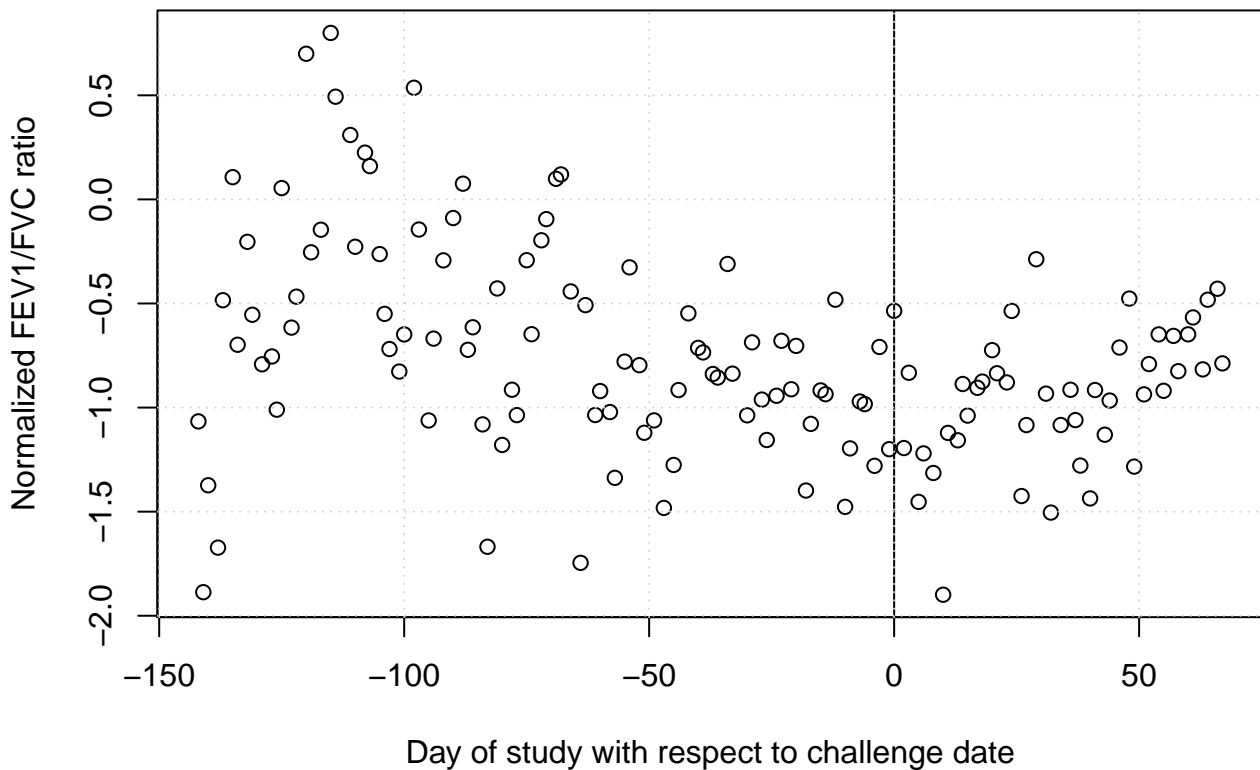

## P02A

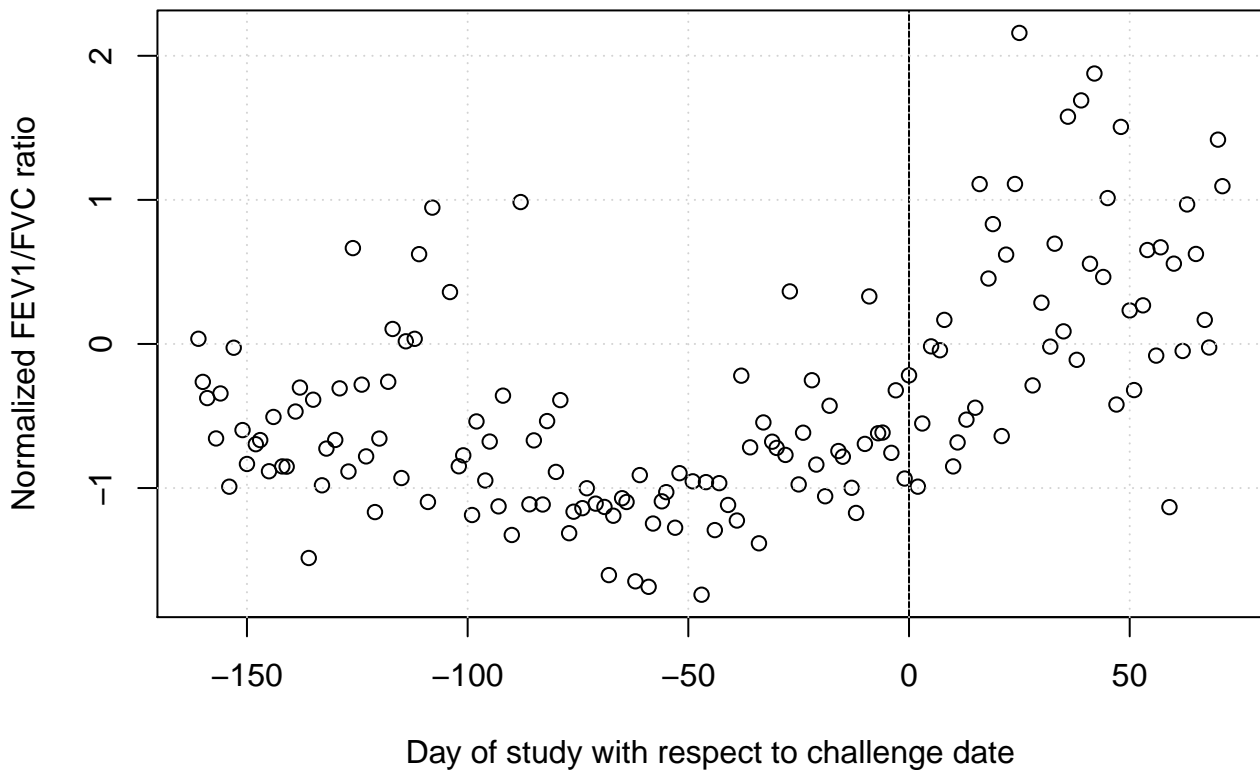

**P04A**

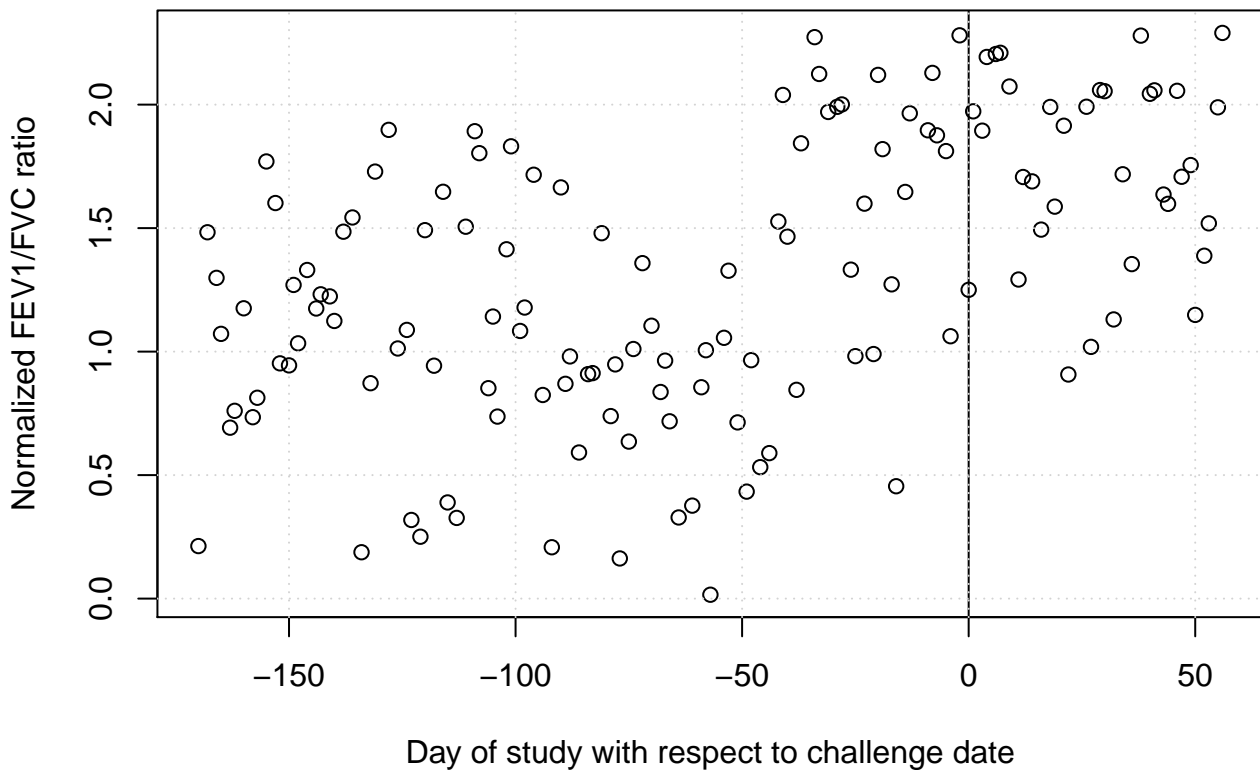

**P05A**

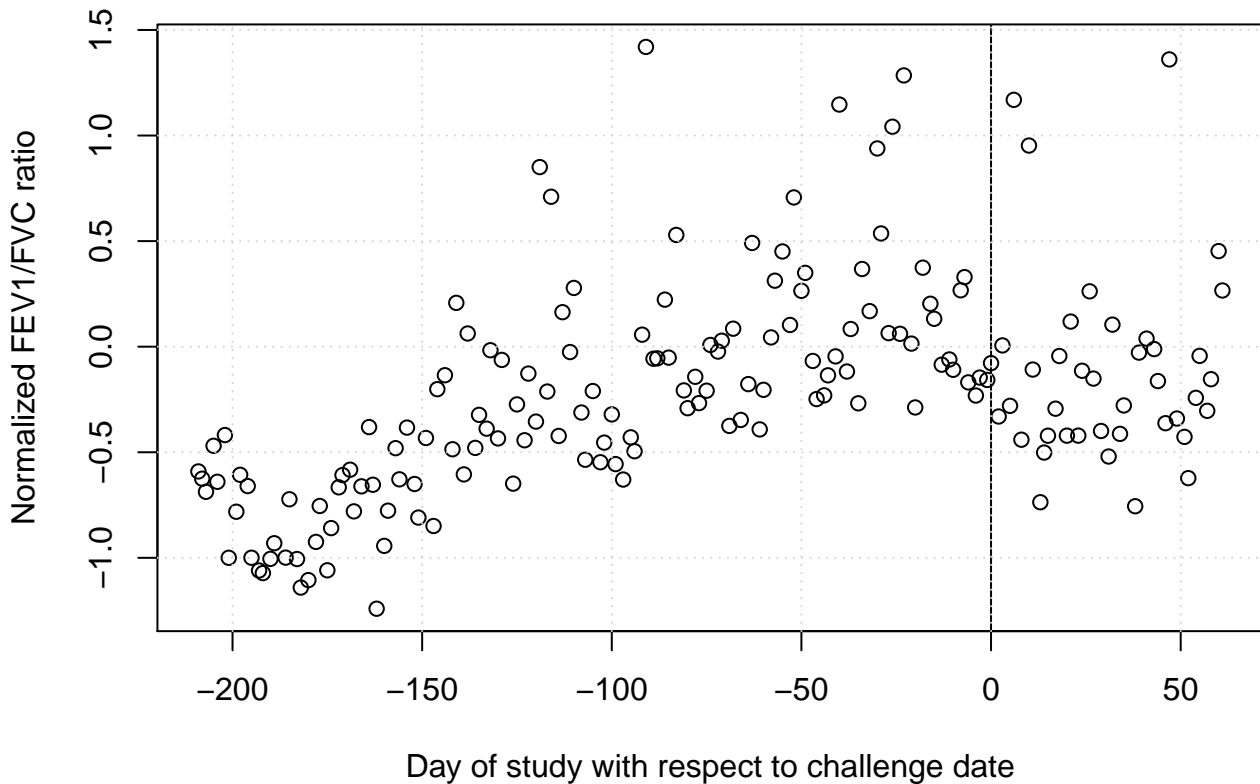

**P06A**

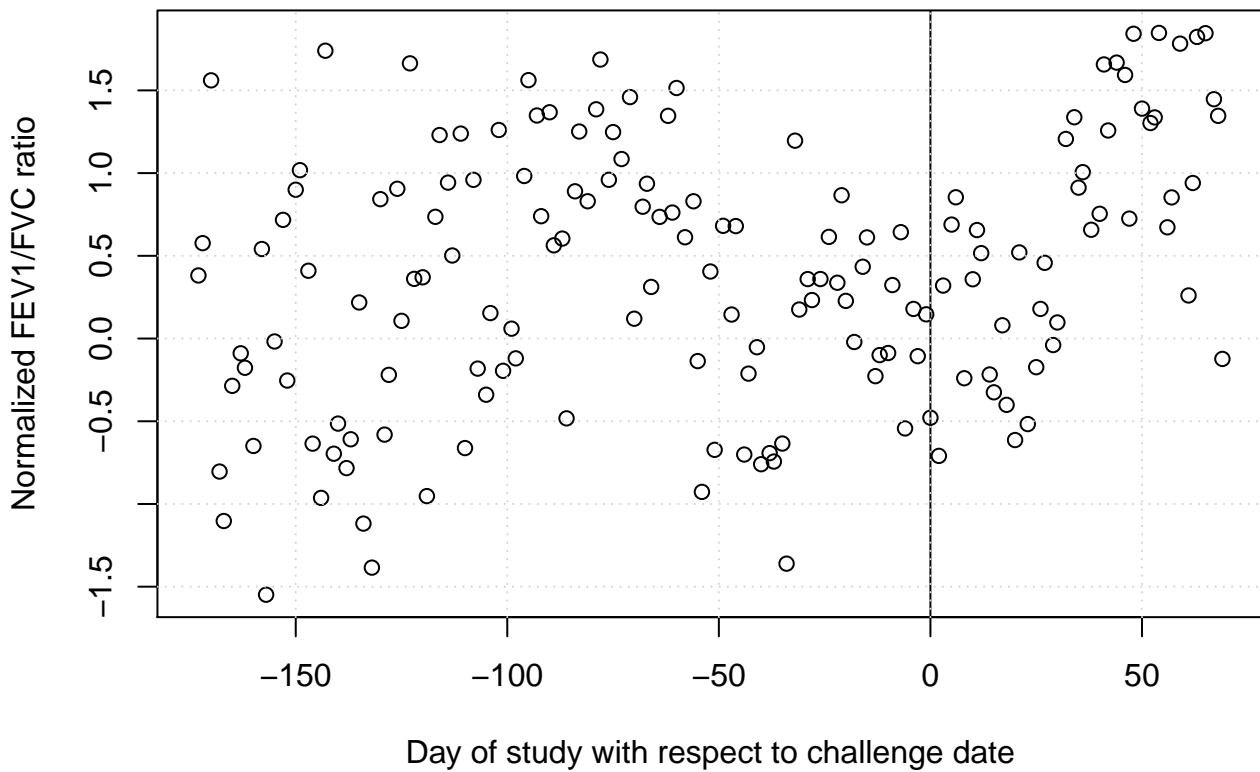

**P07A**

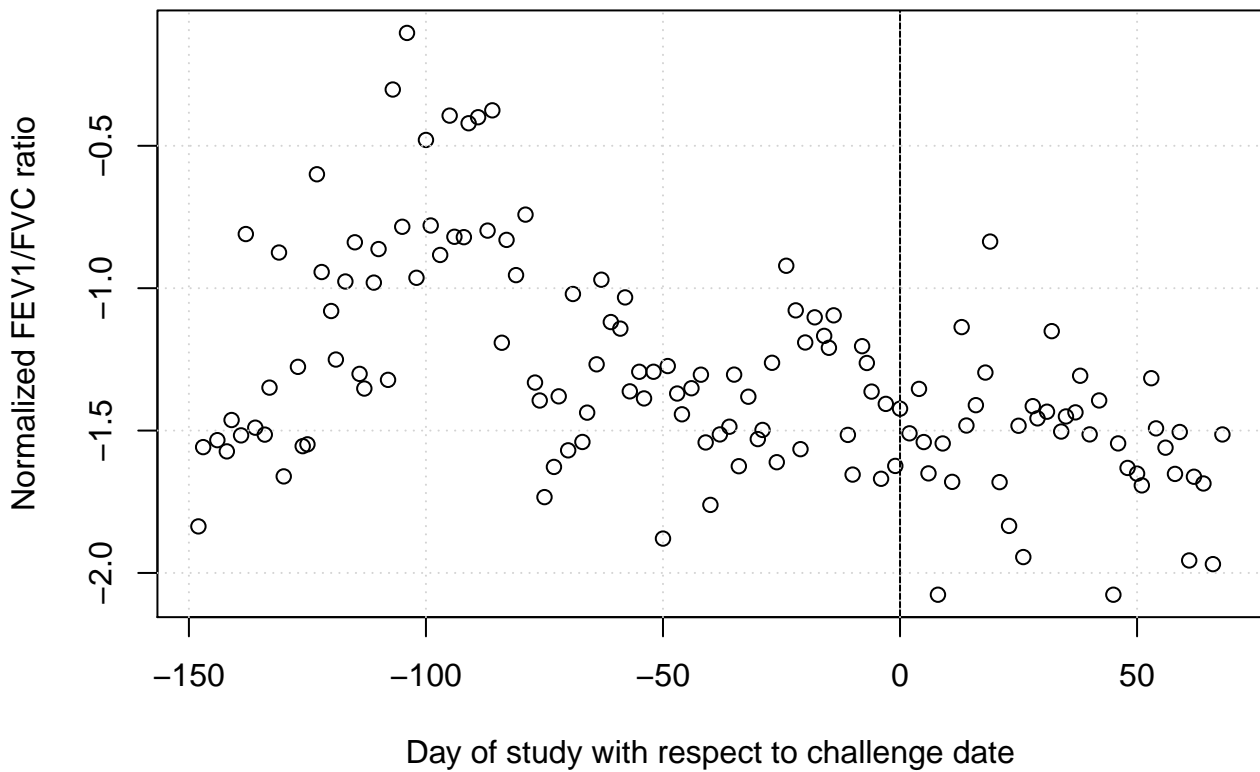

**P08A**

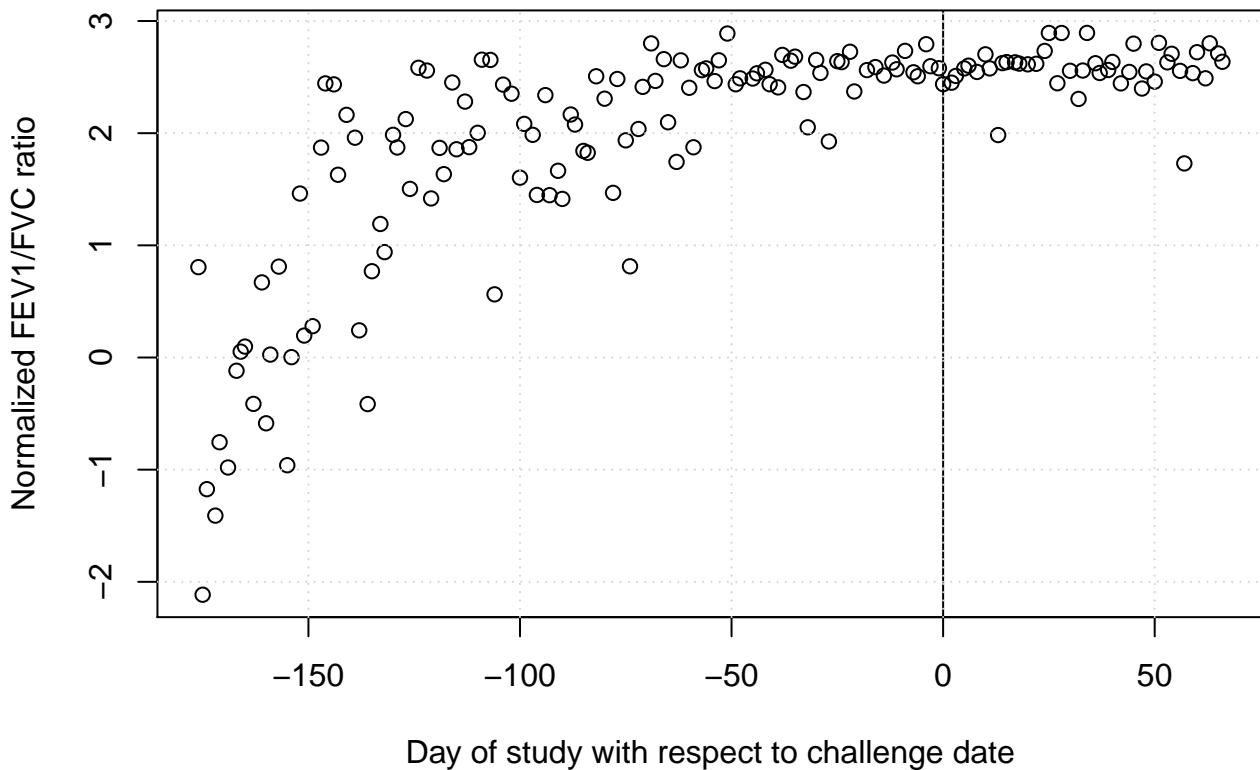

**P09A**

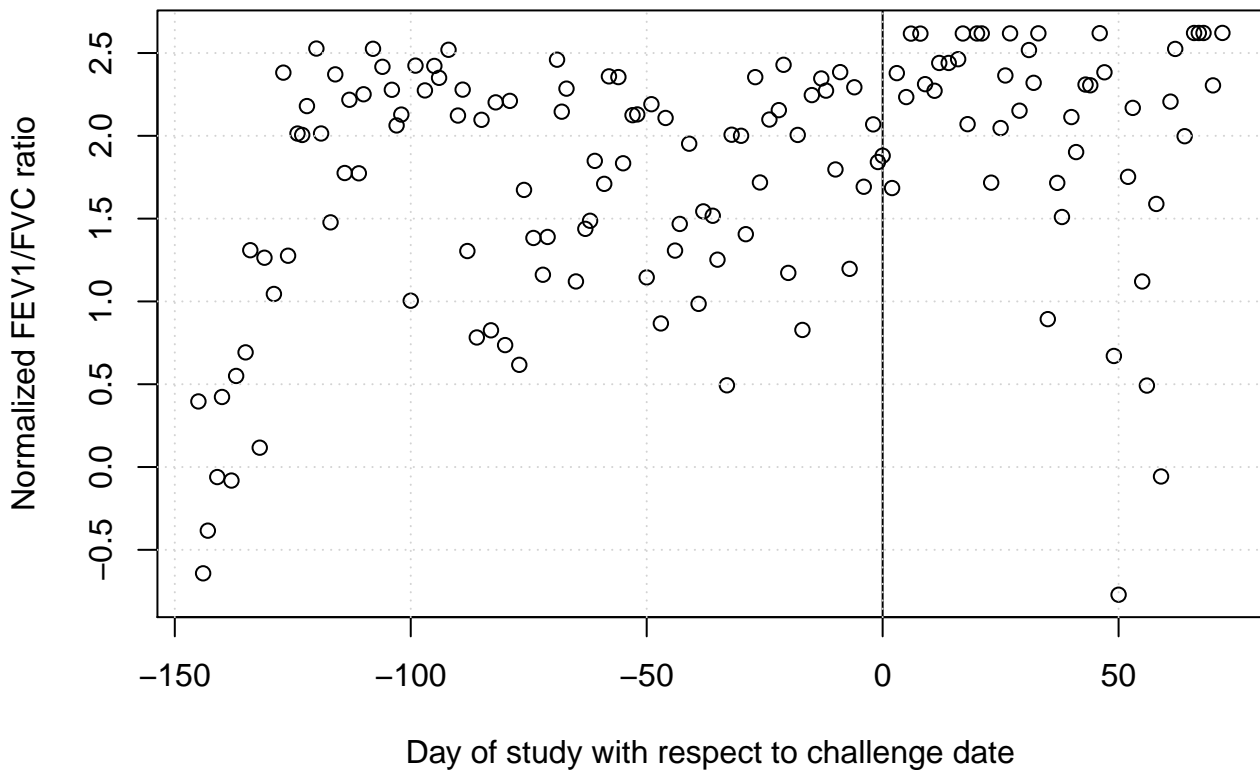

# P10A

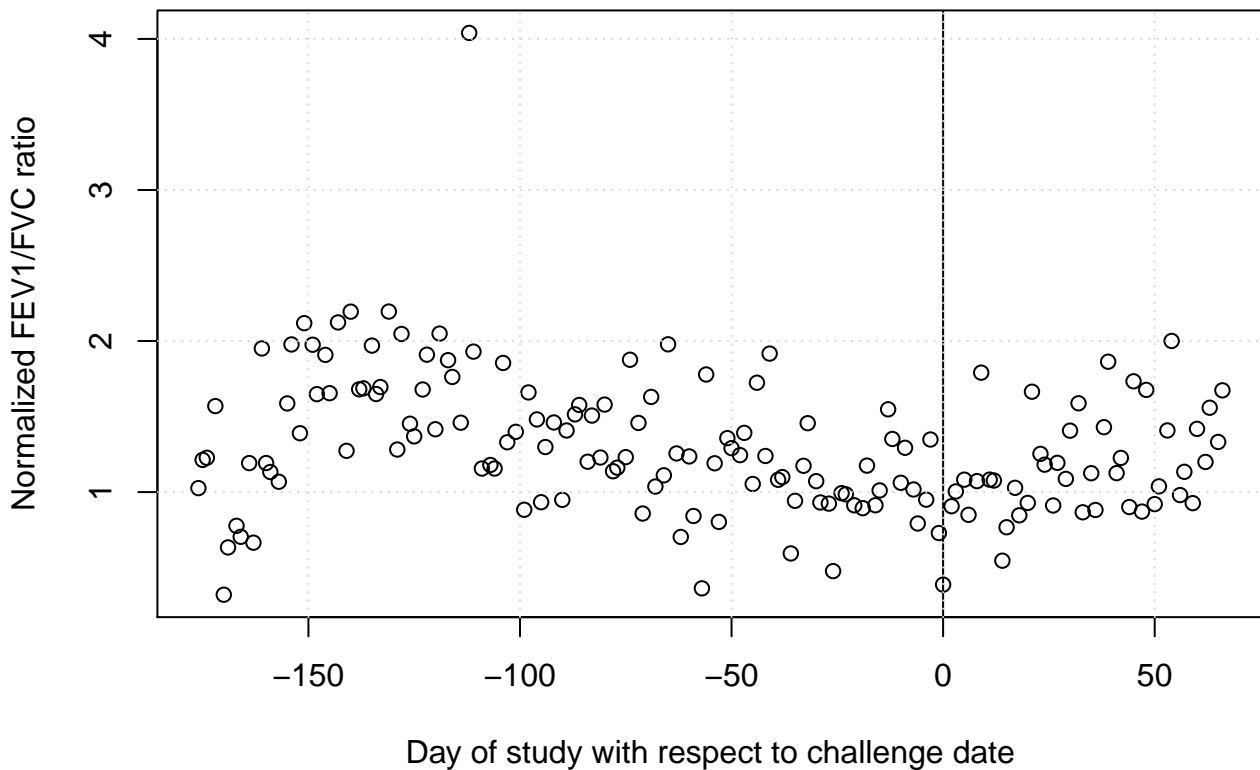

# P11A

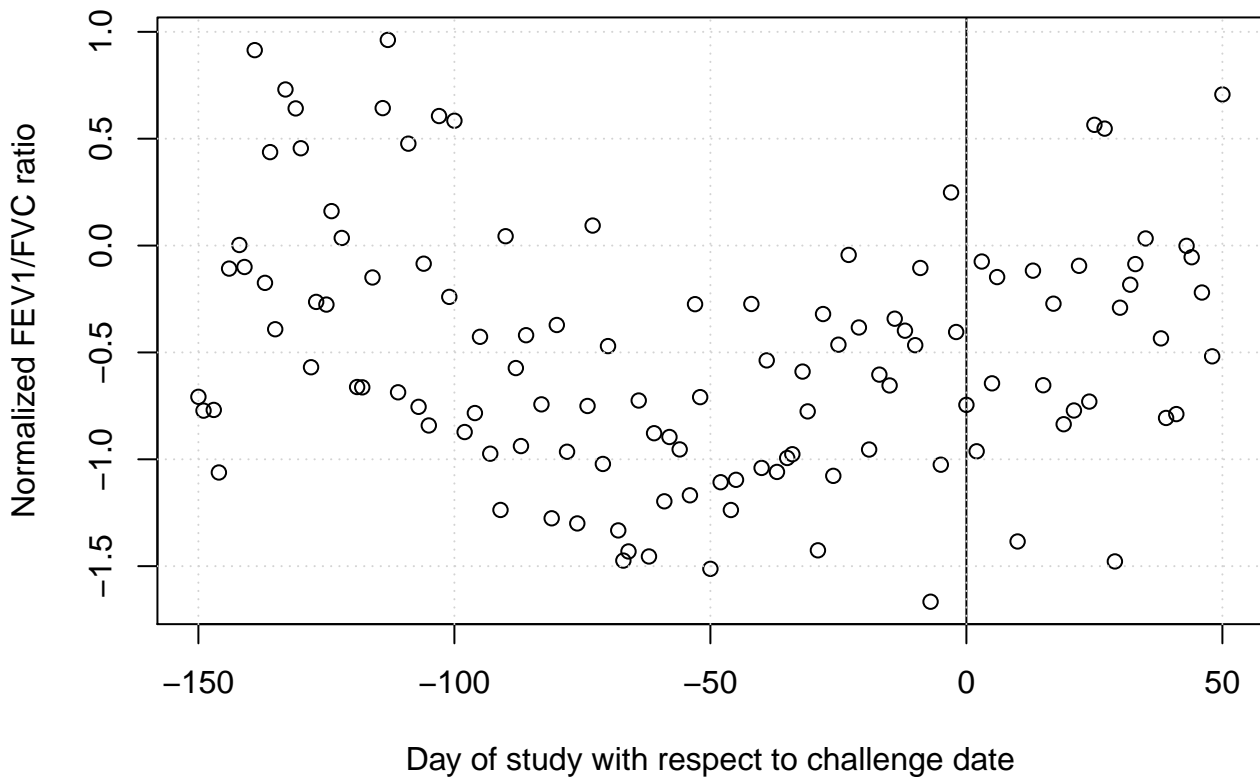

## P12A

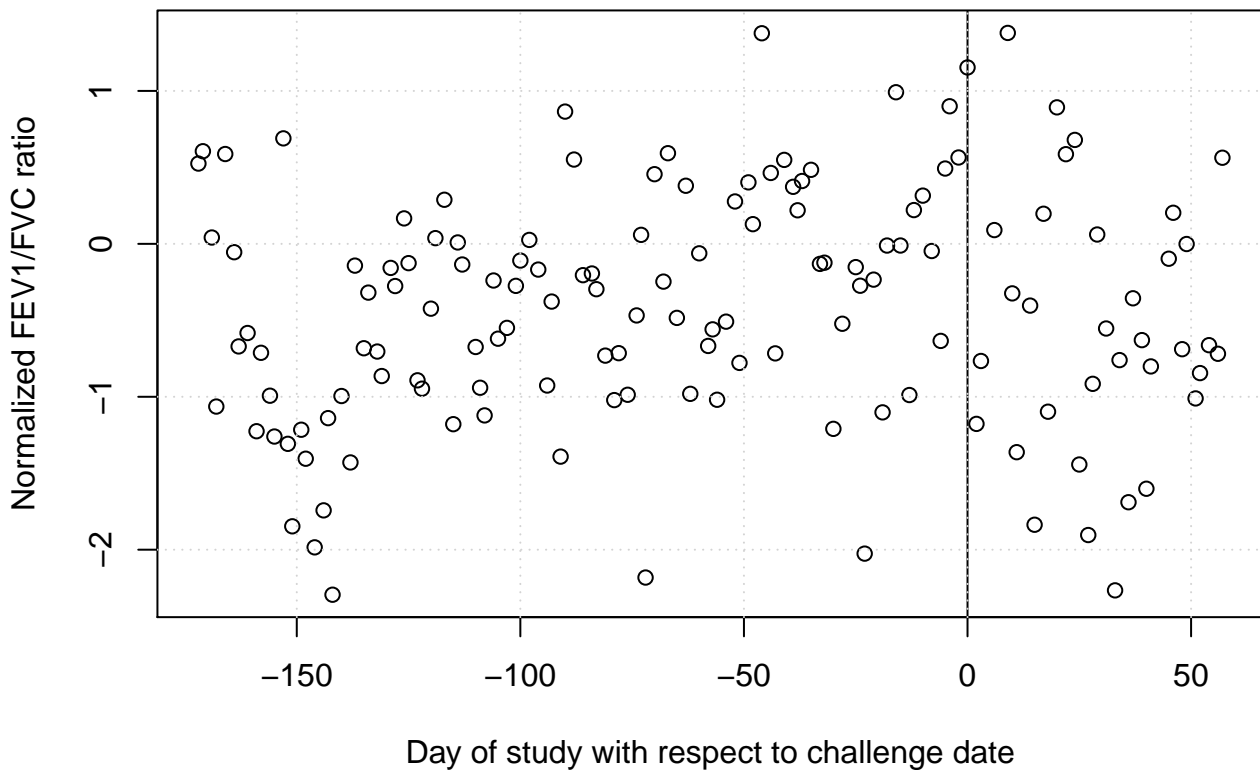

# P13A

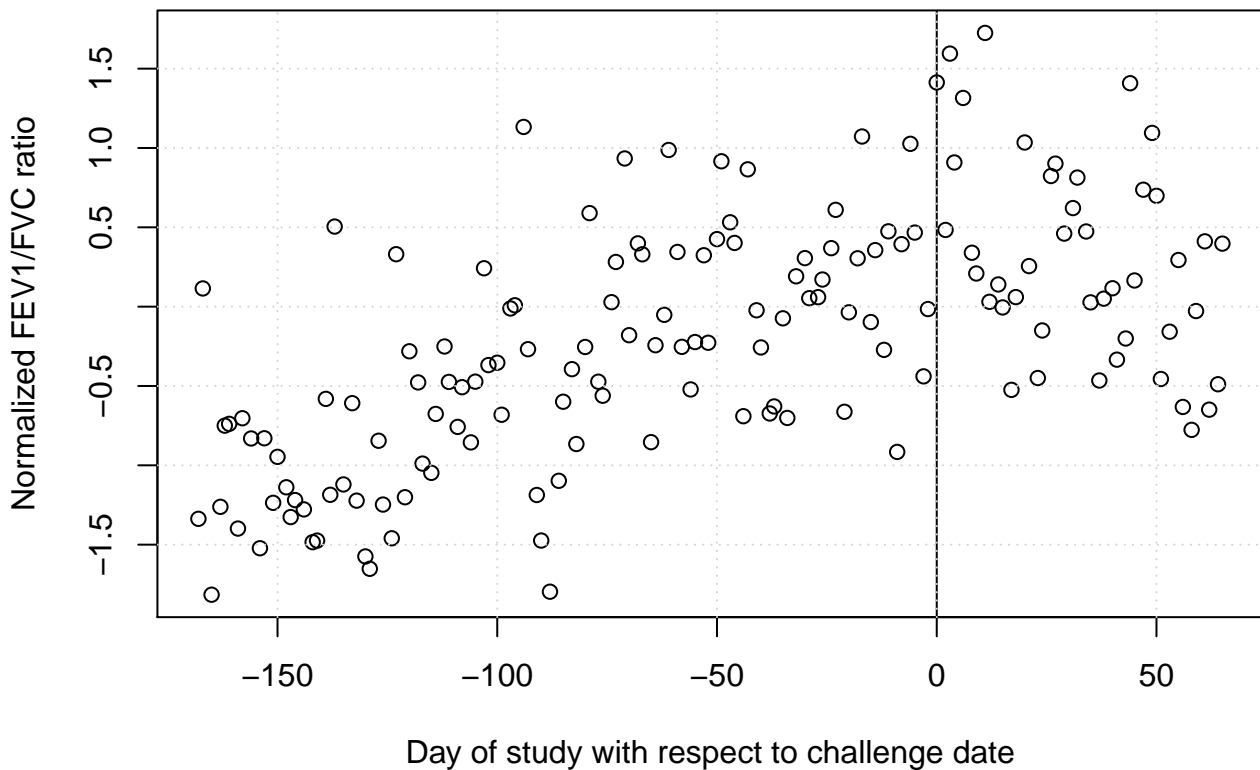

Supplement: Supplementary file 2. [file elife-47969-supp2.zip › TimeSeriesPlots_PDFs/Appendix-figure SS8_NFEV1FVC_TimeSeries_AsthmaticParticipants.pdf]

# P01A

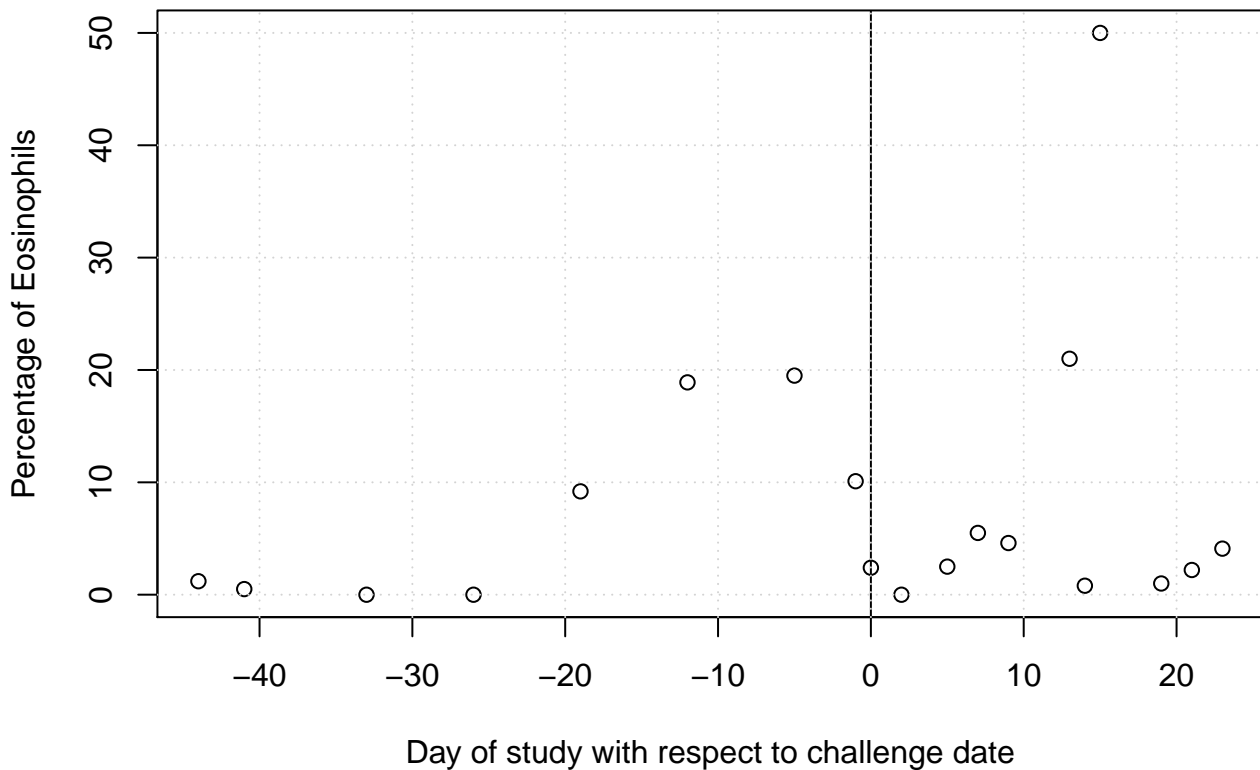

## P02A

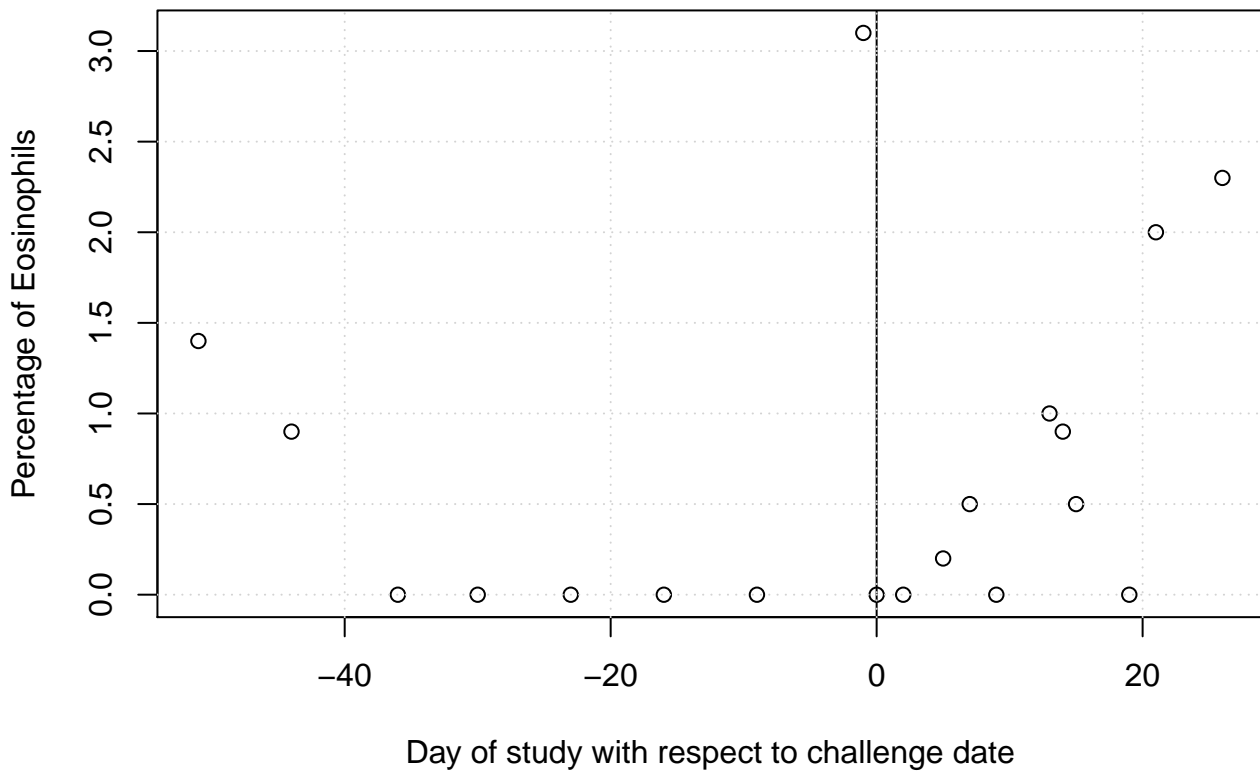

# P04A

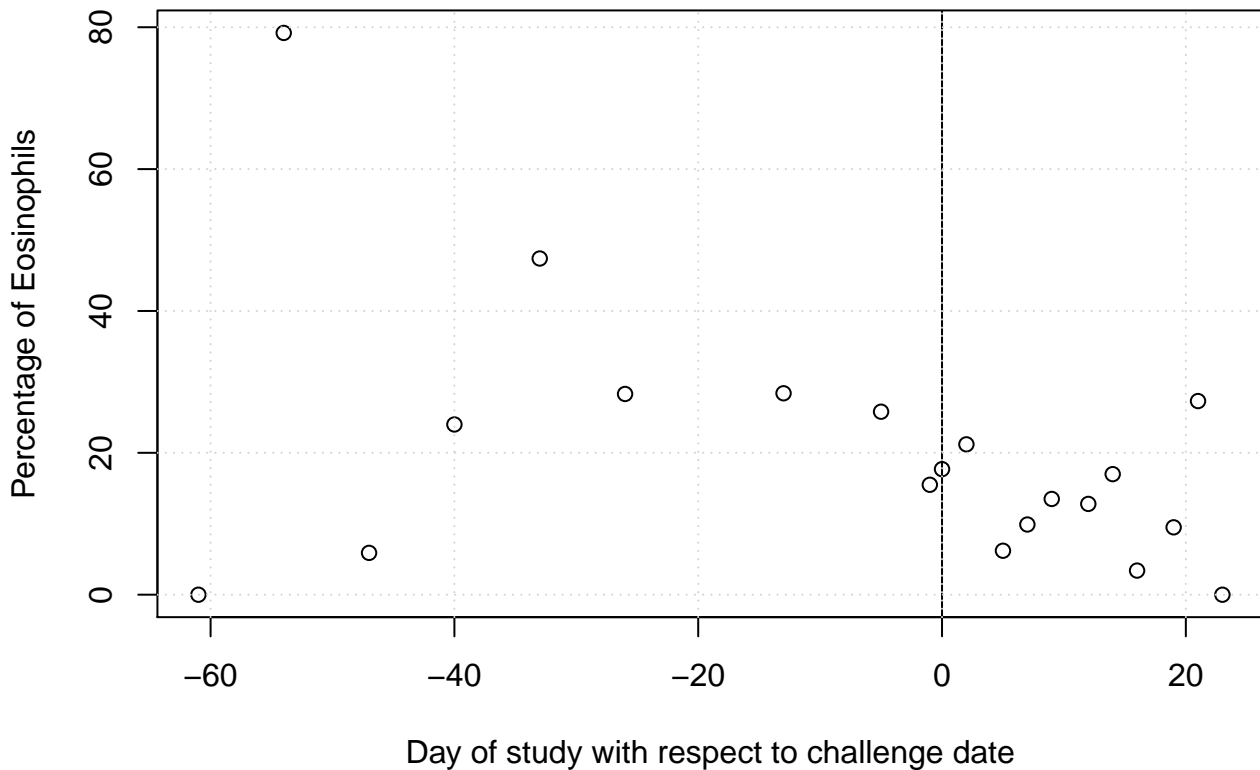

# P05A

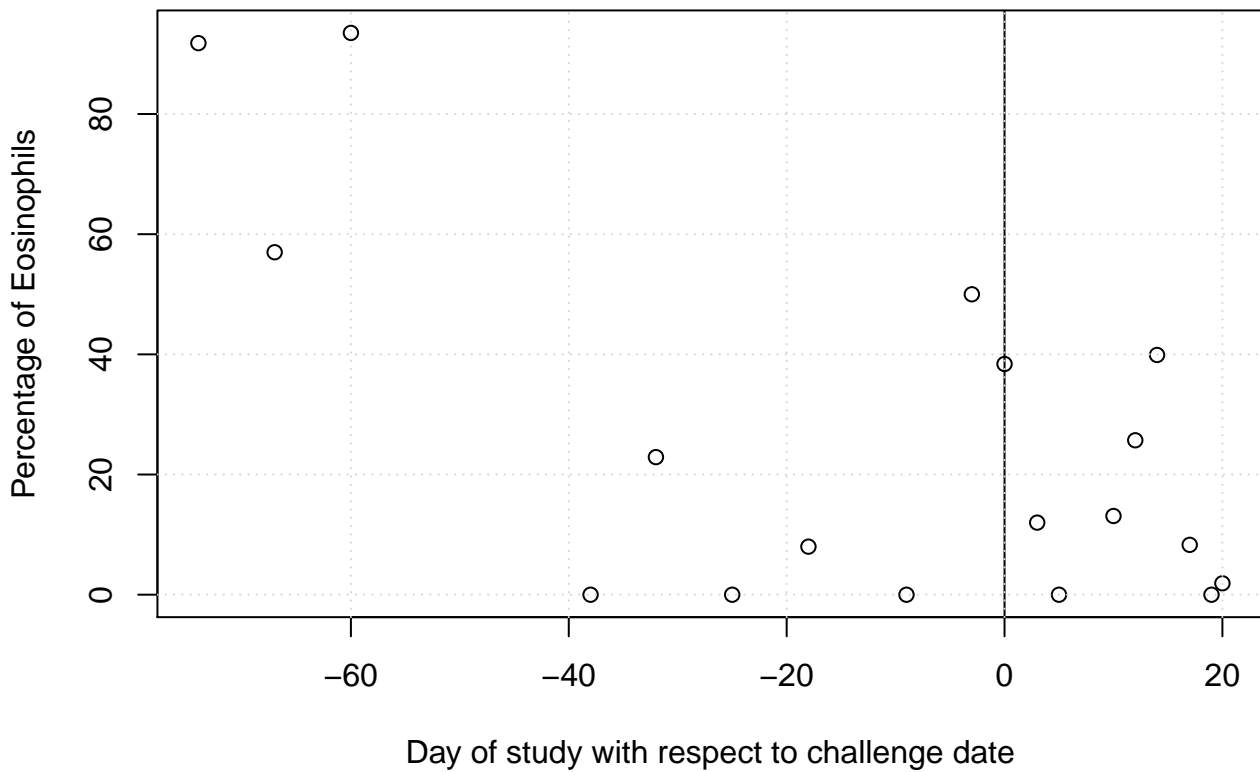

# P06A

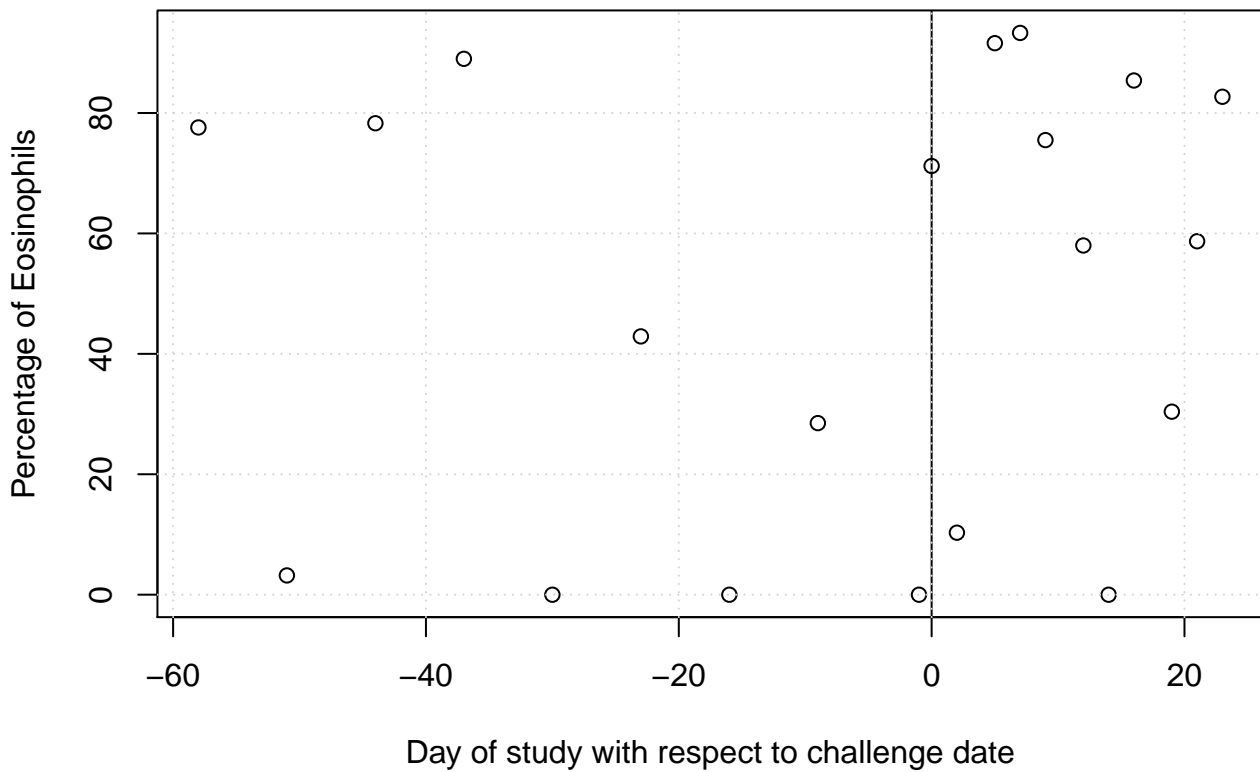

**P07A**

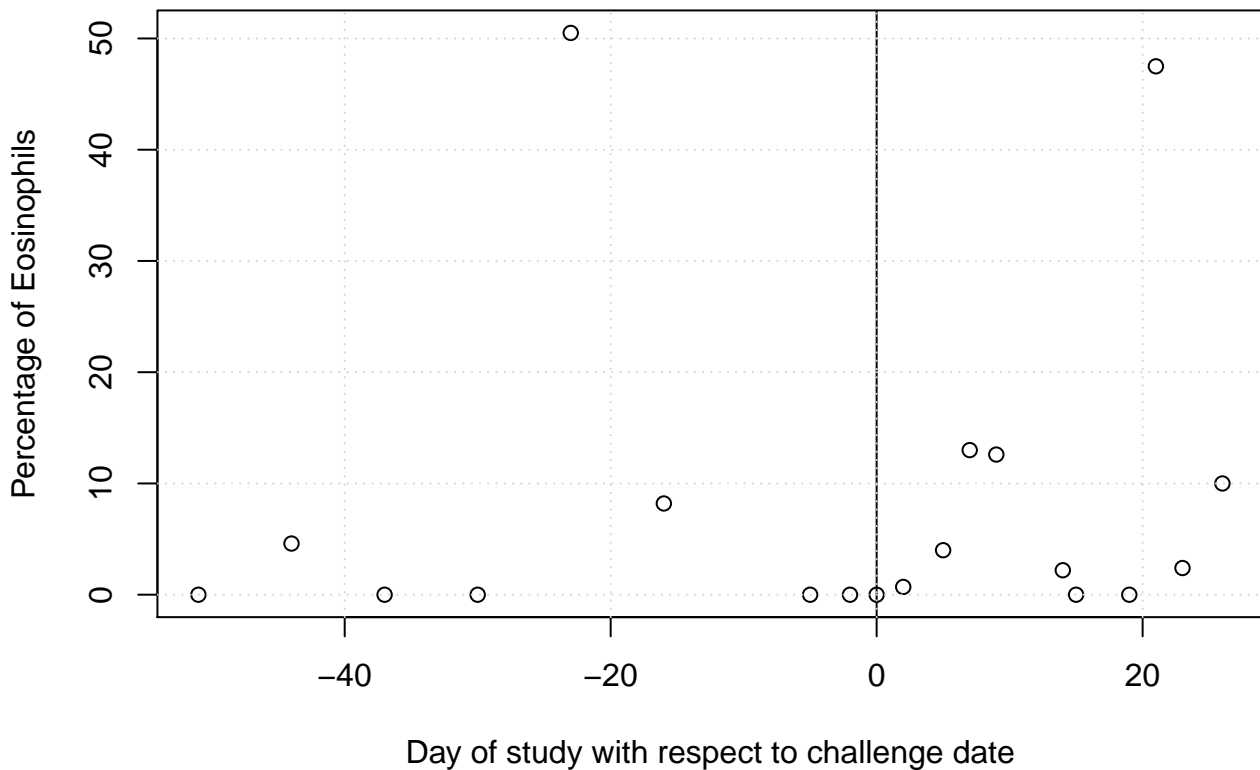

# P08A

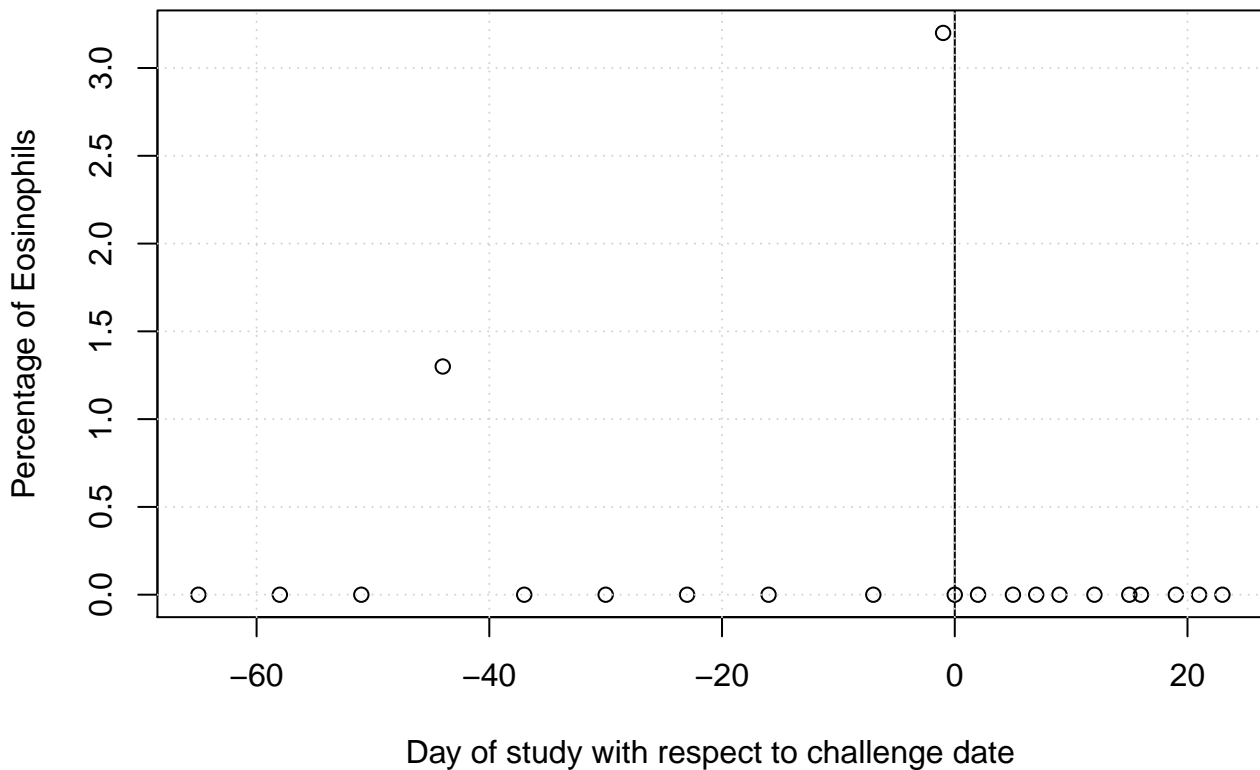

# P09A

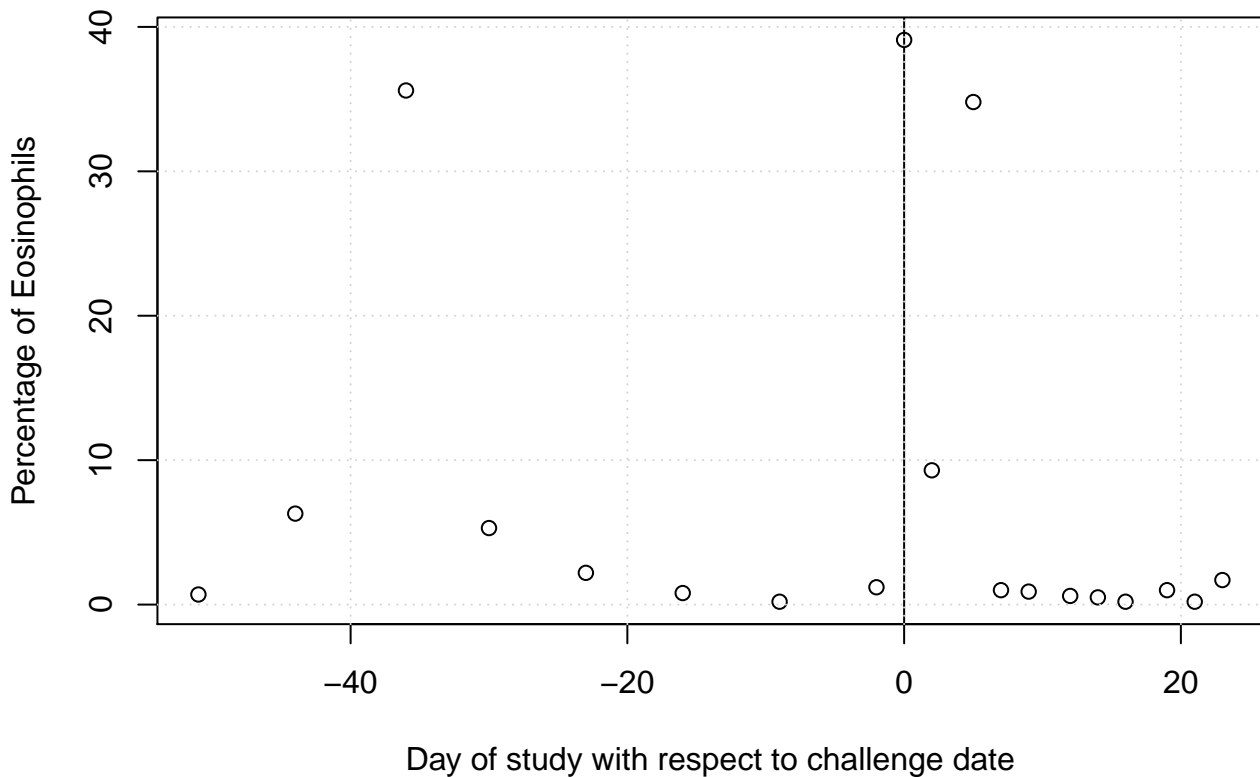

# P10A

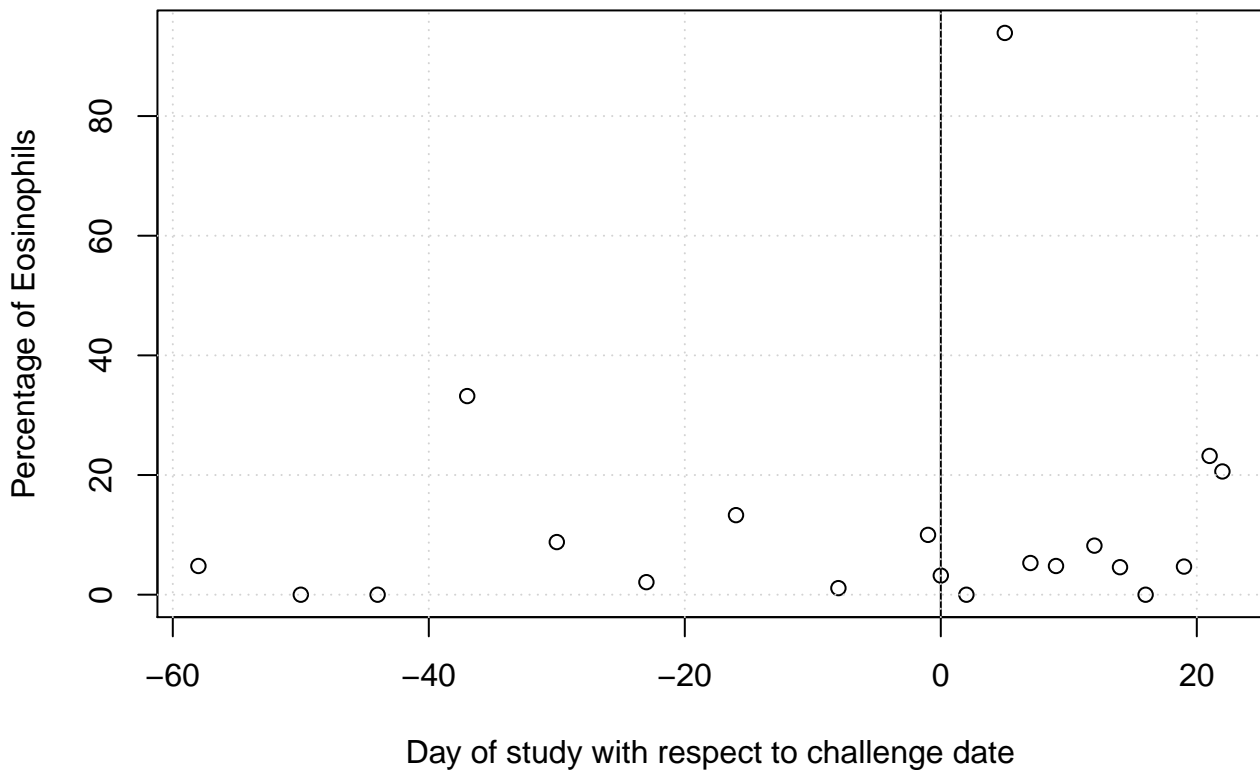

# P11A

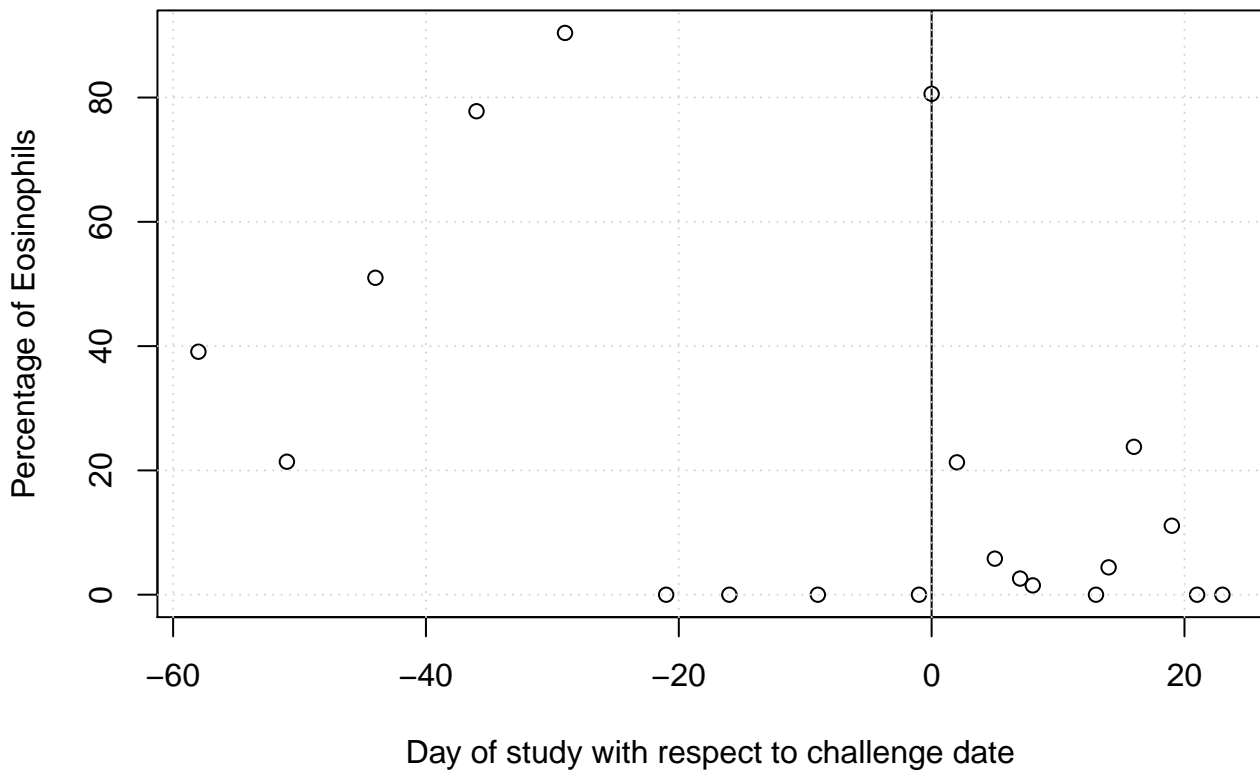

## P12A

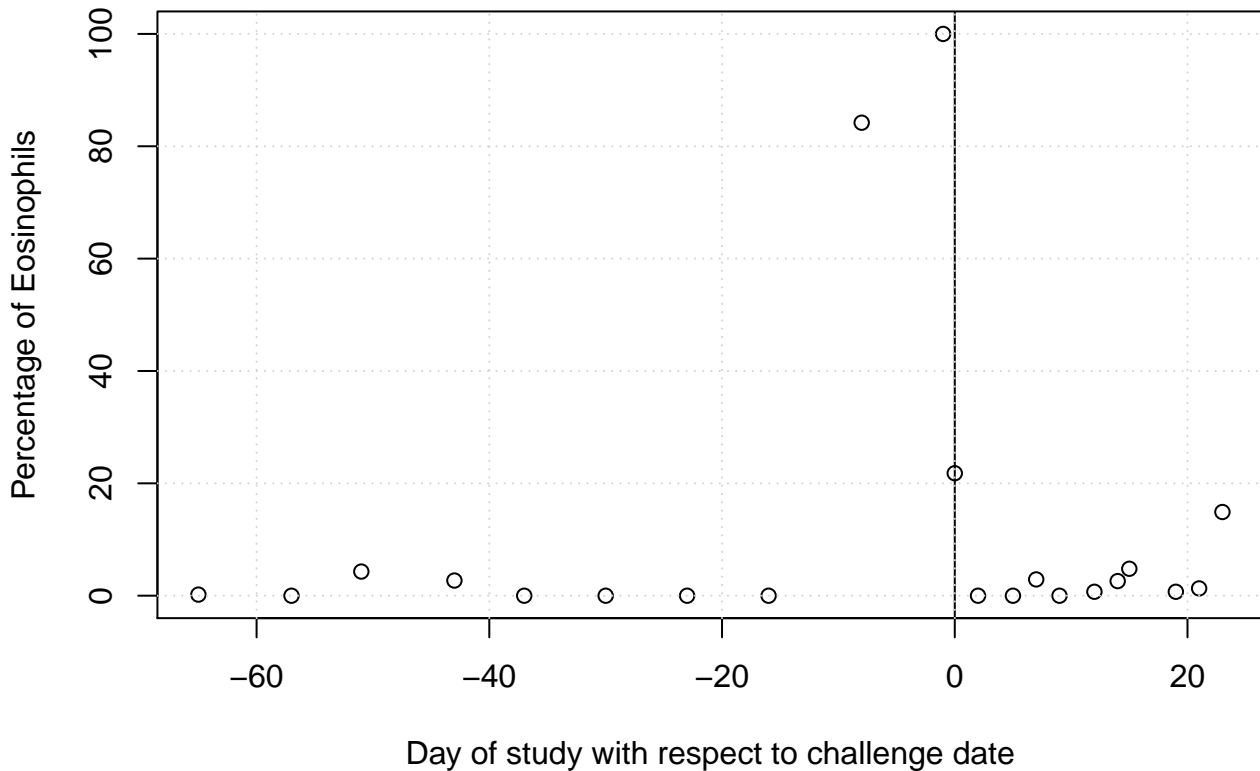

# P13A

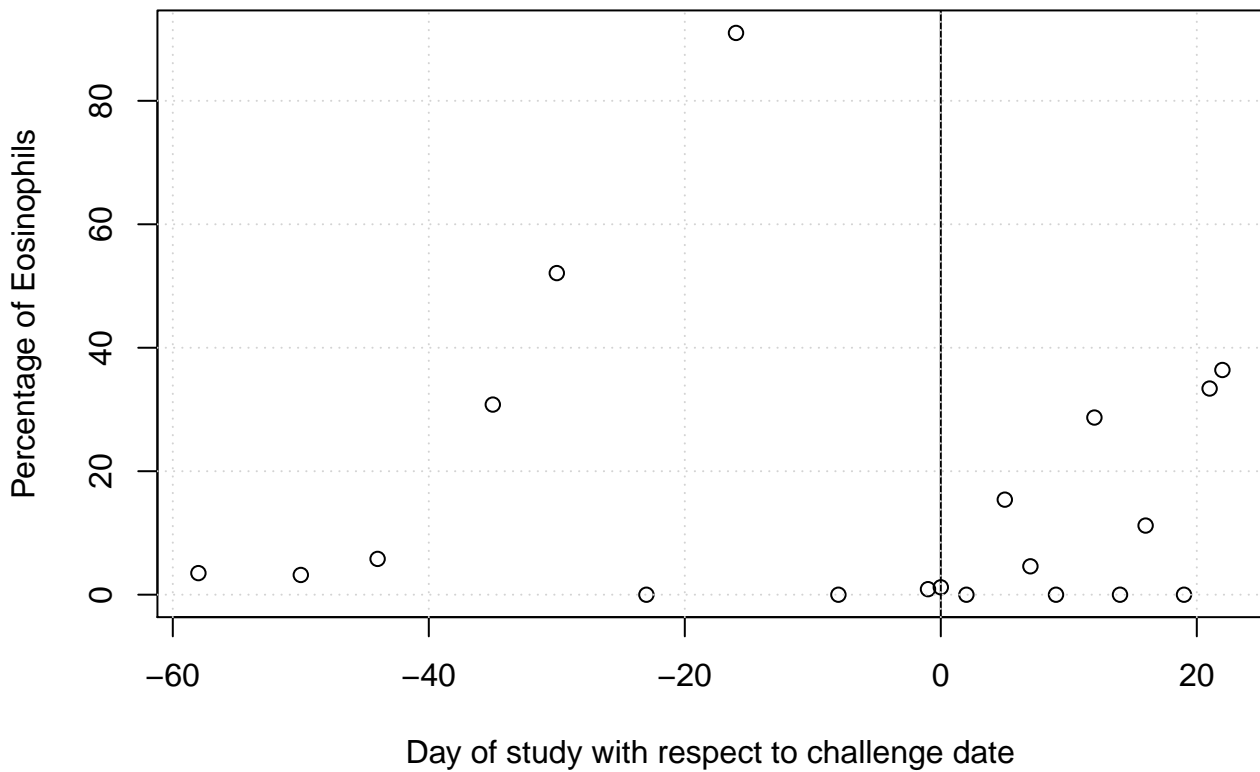

Supplement: Supplementary file 2. [file elife-47969-supp2.zip › TimeSeriesPlots_PDFs/Appendix-figure SS14_PercentageOfEosinophils_TimeSeries_AsthmaticParticipants.pdf]

# P01H

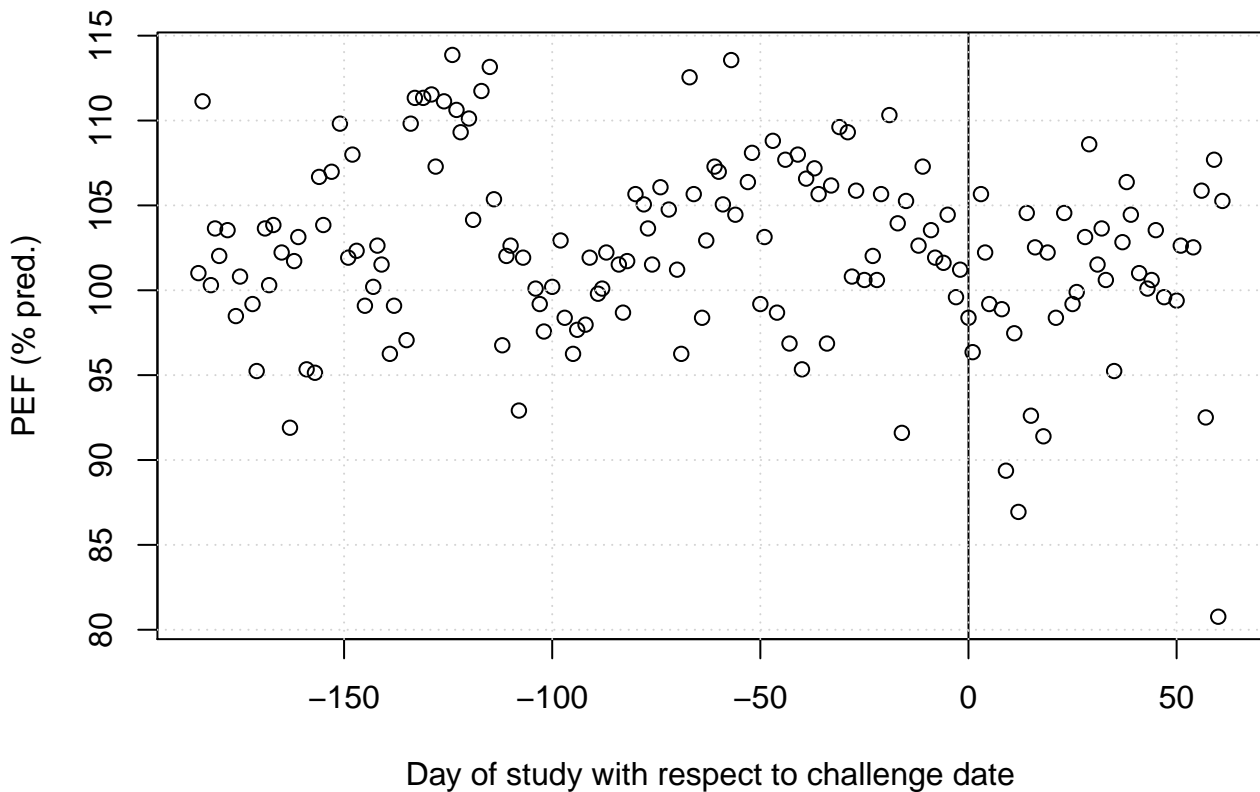

# P03H

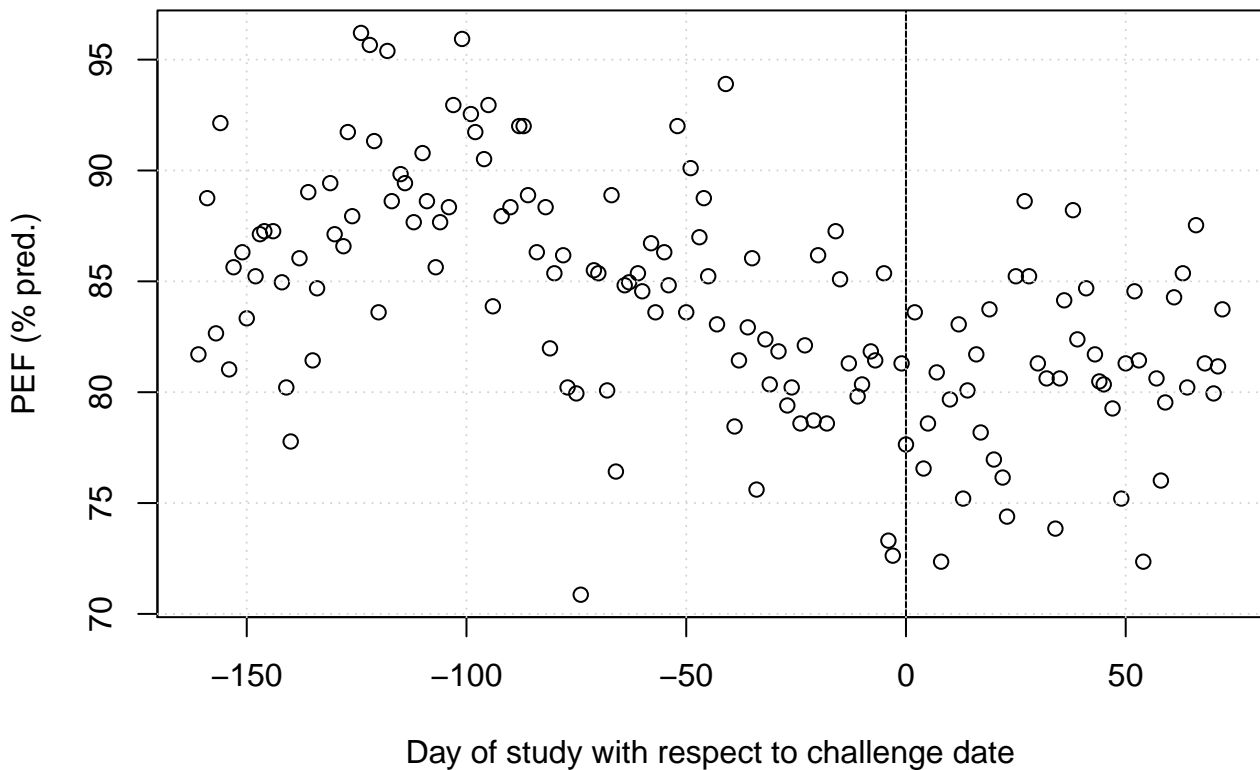

# P05H

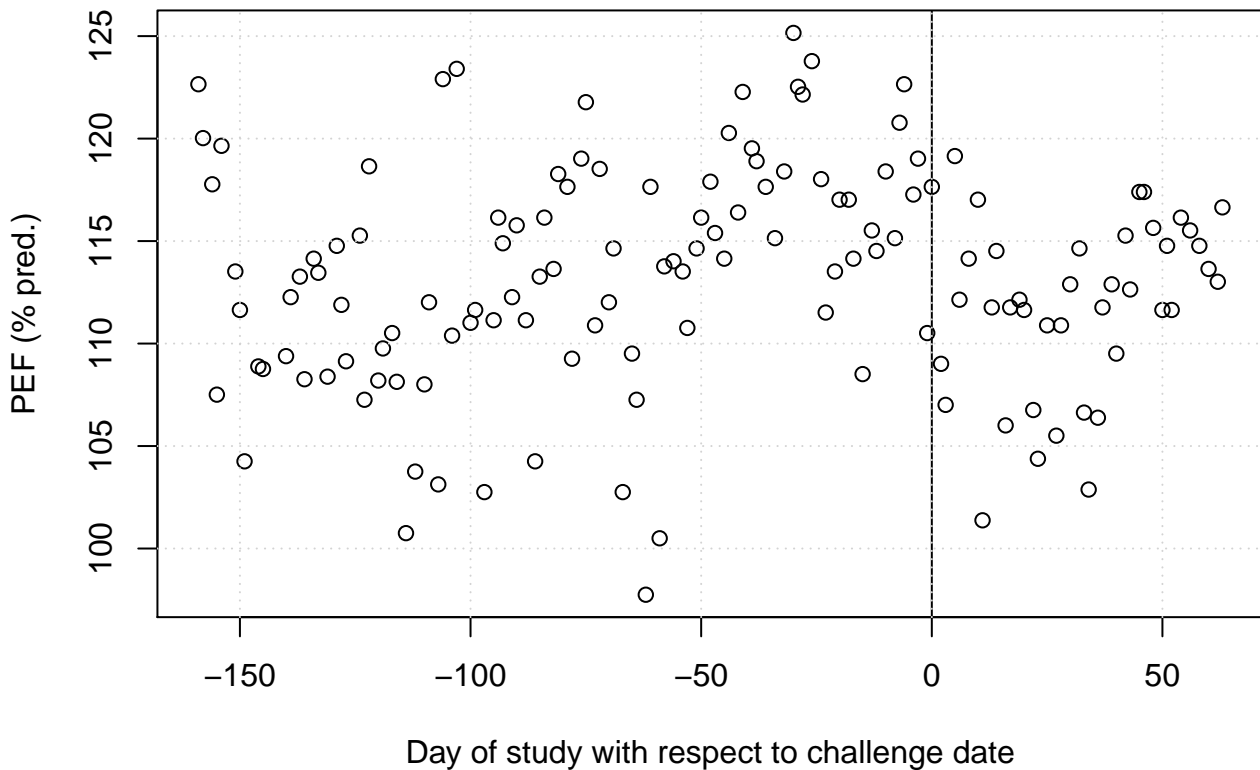

# P06H

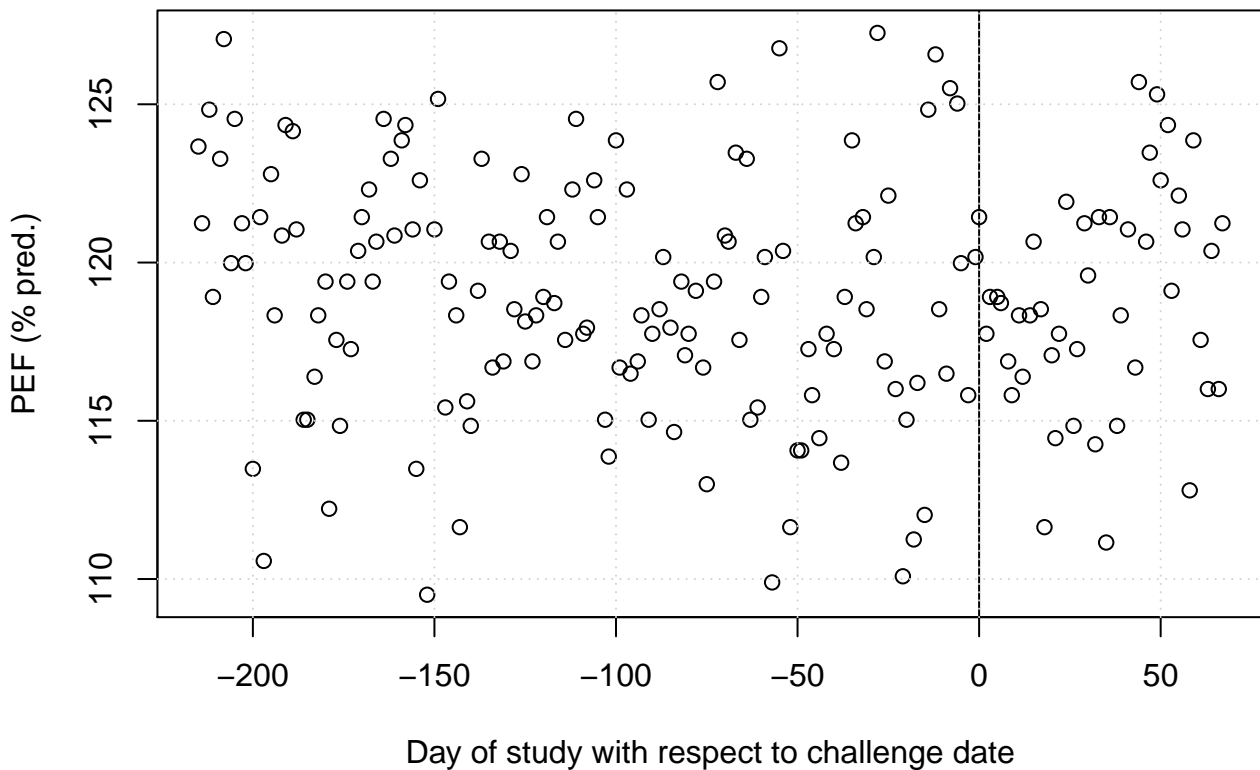

# P07H

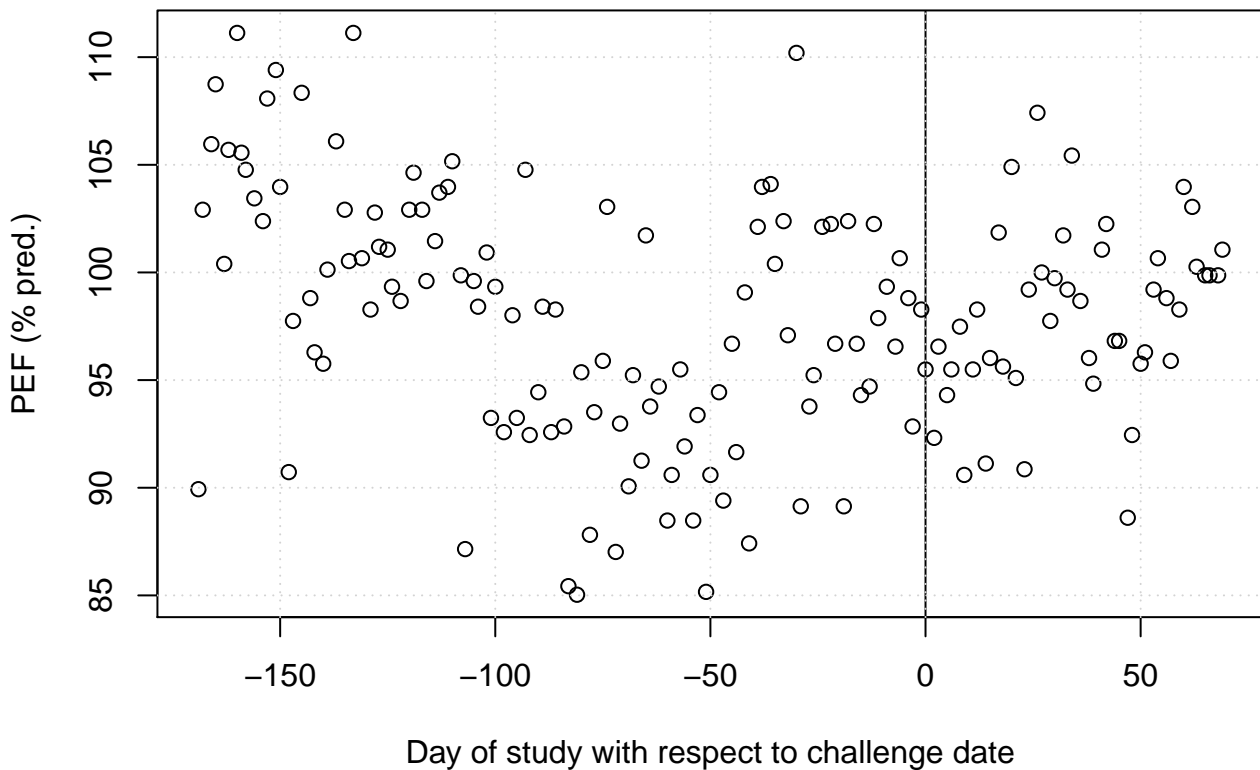

# P08H

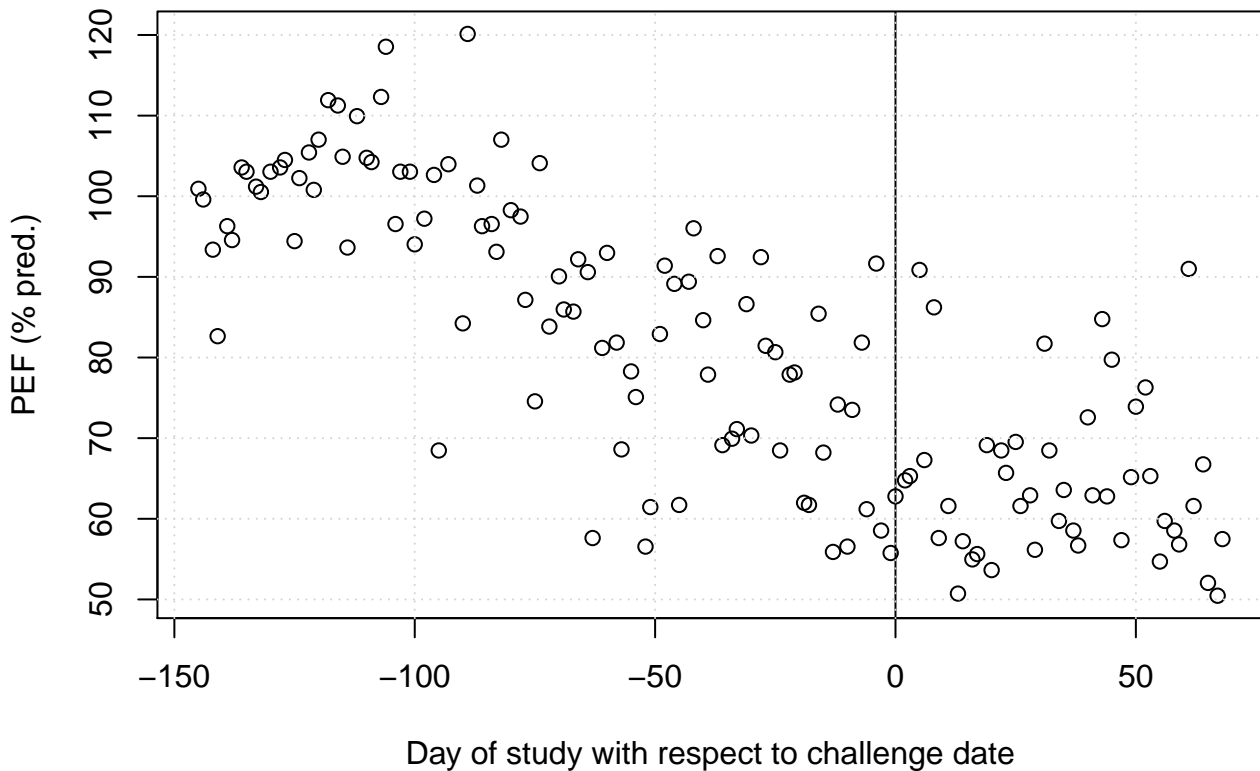

# P09H

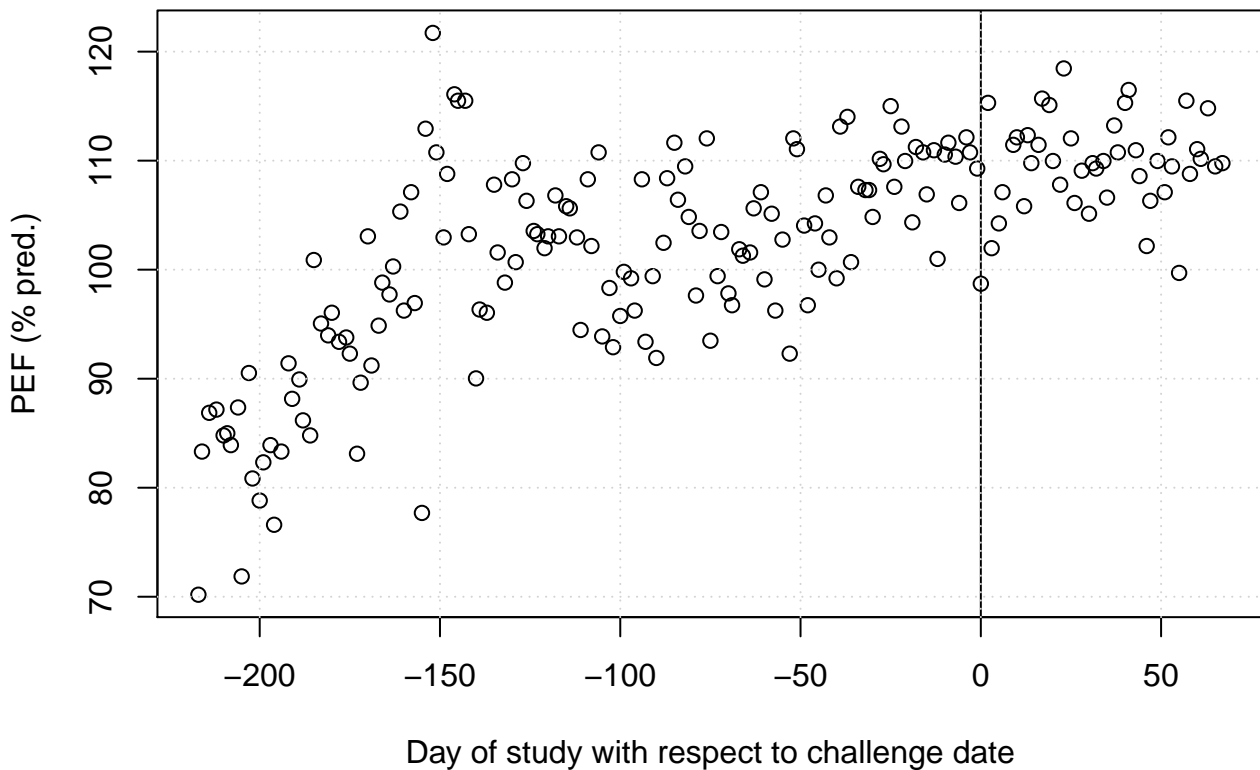

# P11H

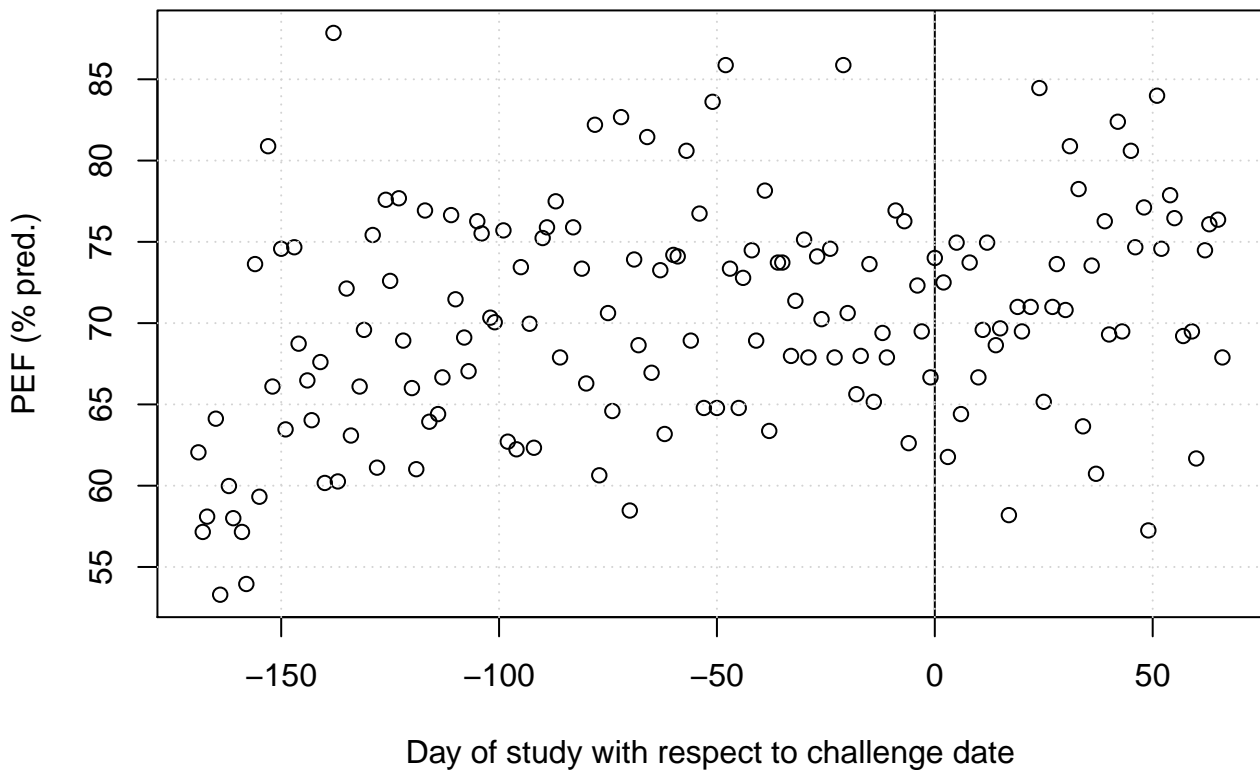

## P12H

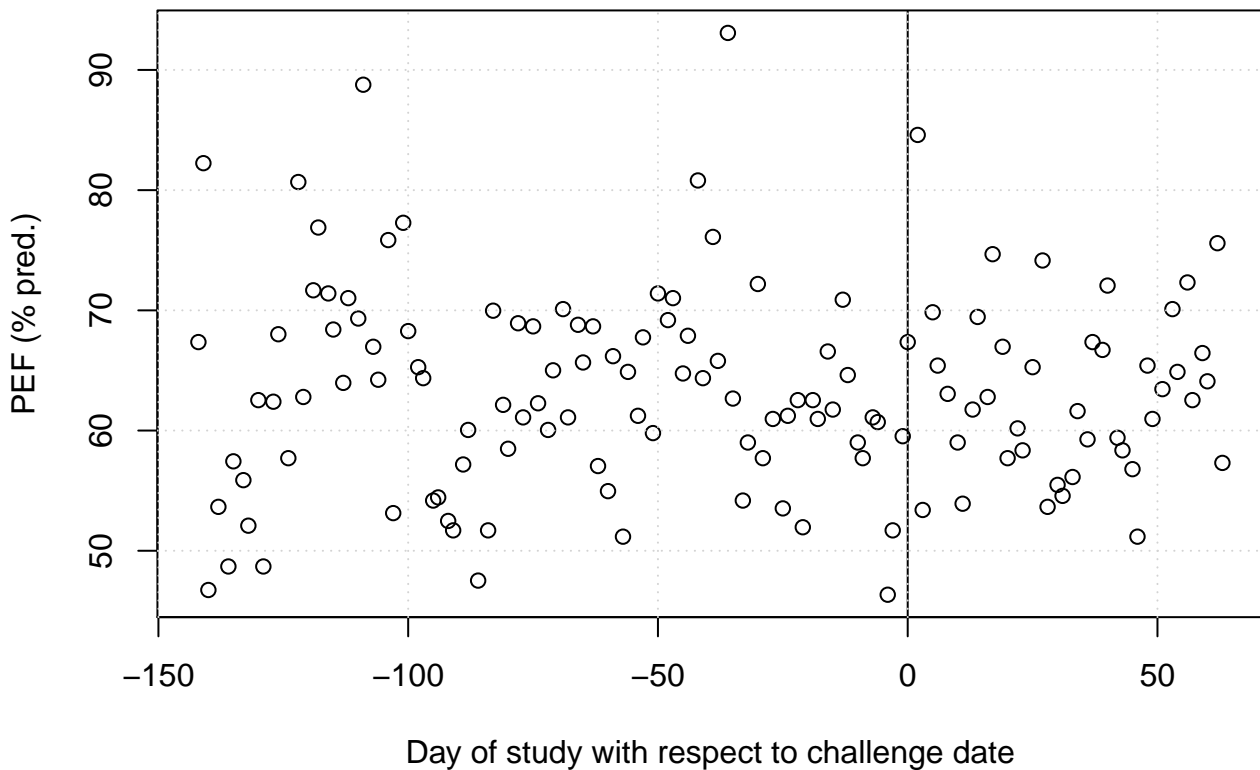

# P13H

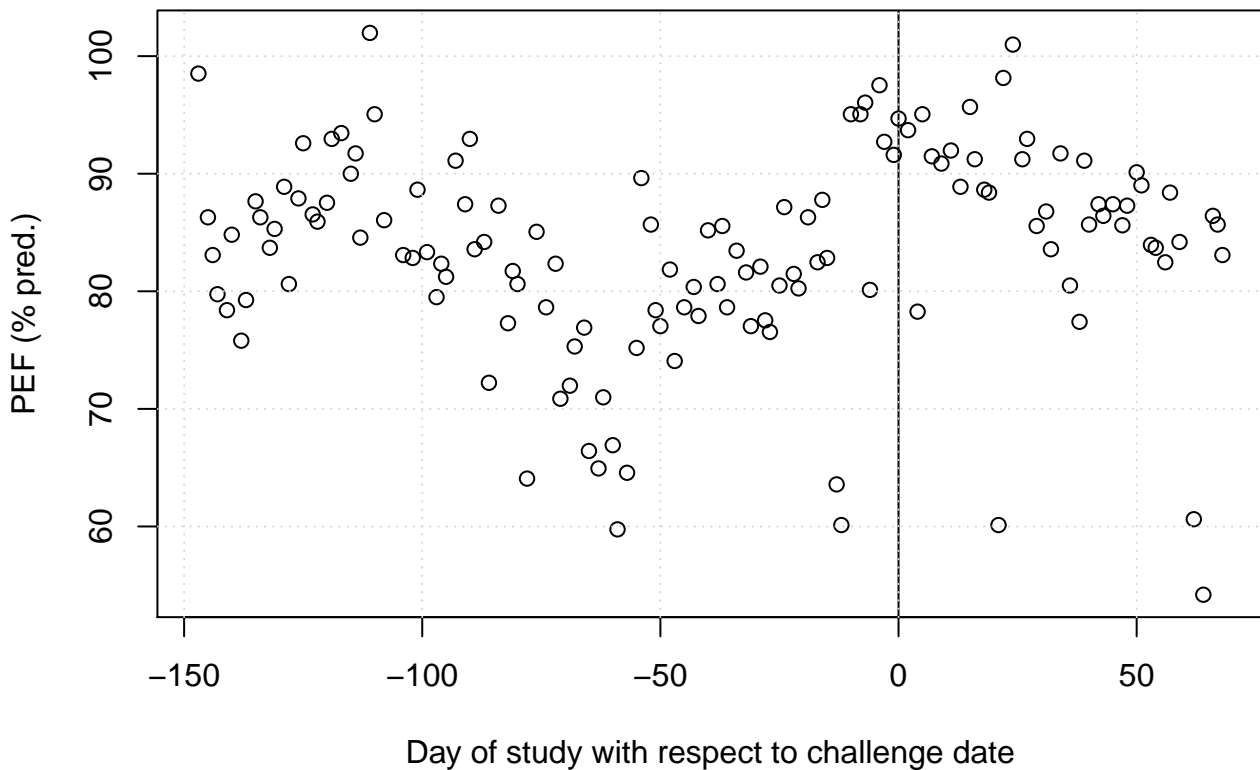

# P14H

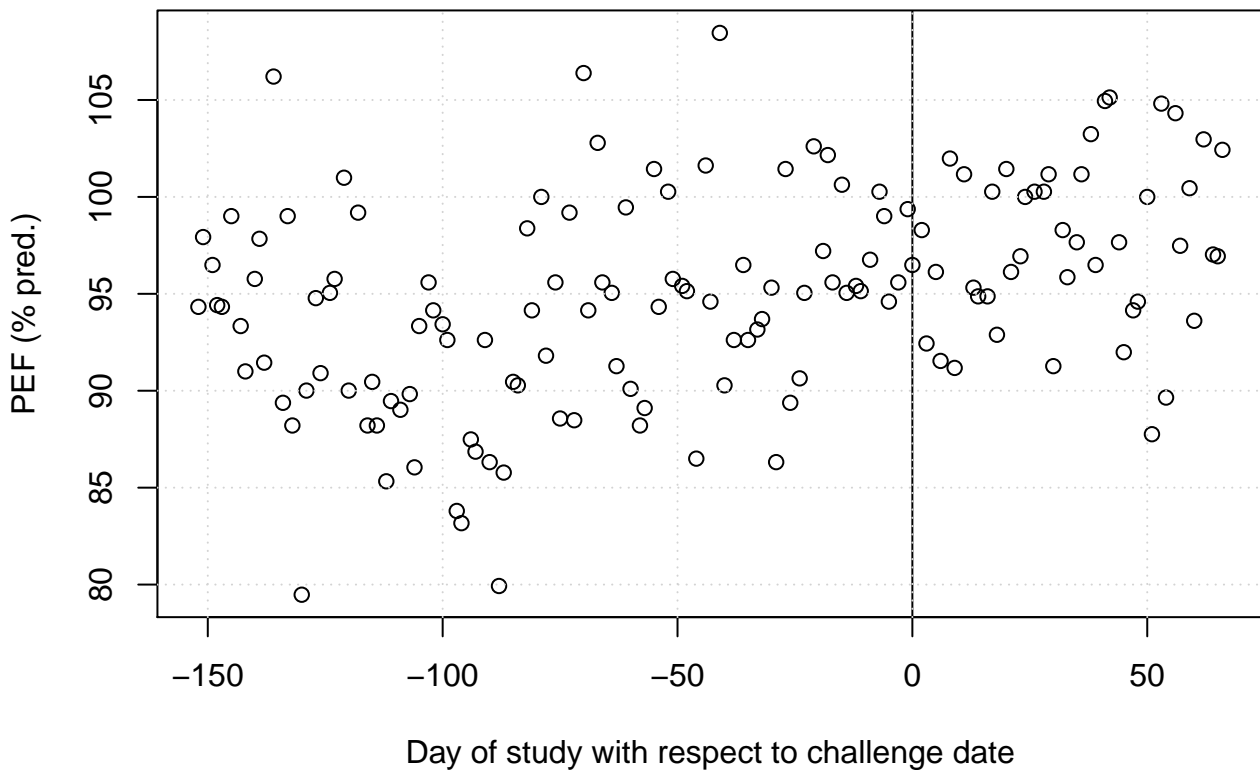

# P15H

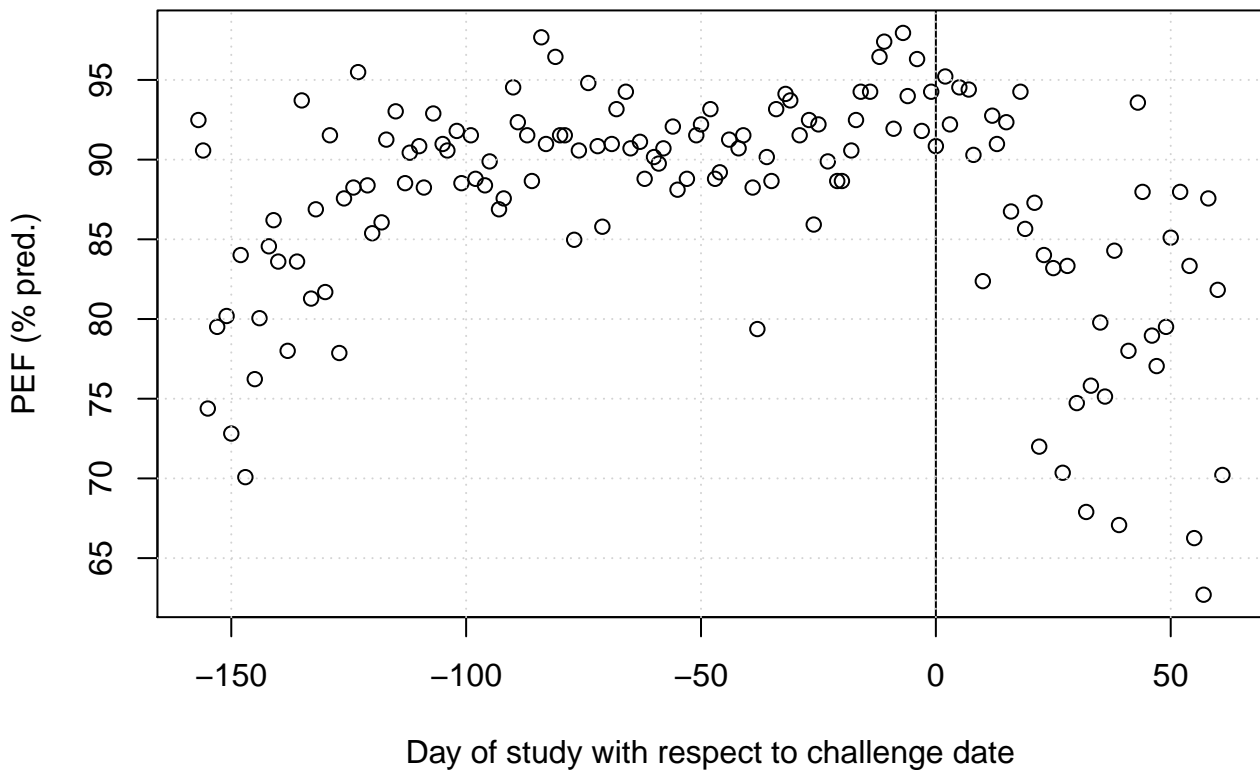

Supplement: Supplementary file 2. [file elife-47969-supp2.zip › TimeSeriesPlots_PDFs/Appendix-figure SS1_PEFpercentPred_TimeSeries_HealthyParticipants.pdf]
